# Supplementary material for: Chiral dinitrogen ligand enabled asymmetric Pd/norbornene cooperative catalysis toward the assembly of C–N axially chiral scaffolds
Source: Nat Commun. 2024 Jun 8;15:4908. doi: 10.1038/s41467-024-48582-w (PMC11162495; doi:10.1038/s41467-024-48582-w)
Supplement: Supplementary file 1 — Supplementary Information [file 41467_2024_48582_MOESM1_ESM.pdf]

Supplementary Information

Chiral dinitrogen ligand enabled asymmetric Pd/norbornene cooperative catalysis toward the assembly of C–N axially chiral scaffolds

Liang Jin[1,2,3,4], Ya Li[1,4], Yihui Mao[1,4], Xiao-Bao He[2,3], Zhan Lu[1], Qi Zhang[1,2]*, Bing-Feng Shi[1]*

[1]*Department of Chemistry, Zhejiang University, Hangzhou 310027, China.*

[2]*ZJU-Hangzhou Global Scientific and Technological Innovation Center, Zhejiang University, Hangzhou 311215, China.*

[3]*College of Chemical and Biological Engineering, Zhejiang University, Hangzhou, China.*

[4]*These authors contributed equally: Liang Jin, Ya Li, Yihui Mao.*

e-mail: qiqiyape@zju.edu.cn; bfshi@zju.edu.cn.

Table of Contents

1. General Information 1
2. Experimental Section 2
    2.1 Preparation of Substrates 2
    2.2 Optimization of Reaction Conditions 19
    2.3 Atroposelective Synthesis of C–N axially chiral scaffolds via chiral BiIM ligand enabled asymmetric Catellani reaction 22
    2.4 Scale-up Preparation, Derivatization, and Application. 85
    2.5 X-ray Crystallographic Data 94
    2.6 Enantiomerization Barrier Determination of 3 95
    2.7 Optical properties 97
    2.8 Unsuccessful substrates 100
3. NMR Spectra 103
4. References 213

1. General Information

Unless otherwise specified, all reagents were purchased from commercial suppliers (Bide Pharmatech, Energy Chemical, TCI, Aldrich, Alfa and J&K) and directly used without further purification. NMR spectra were recorded on a Bruker AV-400 or a Bruker AV-600 for ^{1}H NMR at 400 MHz, ^{13}C NMR at 101 MHz, ^{19}F NMR at 376 MHz or ^{1}H NMR at 600 MHz, ^{13}C NMR at 151 MHz, ^{19}F NMR at 565 MHz using TMS as internal standard ($\delta = 0.00$). The following abbreviations (or combinations thereof) were used to explain multiplicities: s = singlet, d = doublet, t = triplet, q = quartet, hept = septet, m = multiplet, br = broad singlet. Mass spectroscopy data of the products were collected on an HRMS-TOF instrument. The ee and dr values were determined on Shimadzu LC-20A HPLC system using CHIRALPAK column unless notified. Emission spectra were measured with a Shimadzu RF-6000 spectrometer. Circular polarized luminescence (CPL) spectra were measured on a JASCO CPL-300 spectrometer. Absorption spectra and circular dichroism (CD) spectra were measured on a Jasco J-1500-150ST CD spectrometer. Optical rotation data were obtained on a PerkinElmer Model 341 Polarimeter. Chiral Imidazoline Ligands **L20-L36** were synthesized by known literature [1] with corresponding adjustment.

2. Experimental Section

2.1 Preparation of Substrates

Synthesis of aryl iodide S4 (General Procedure A)

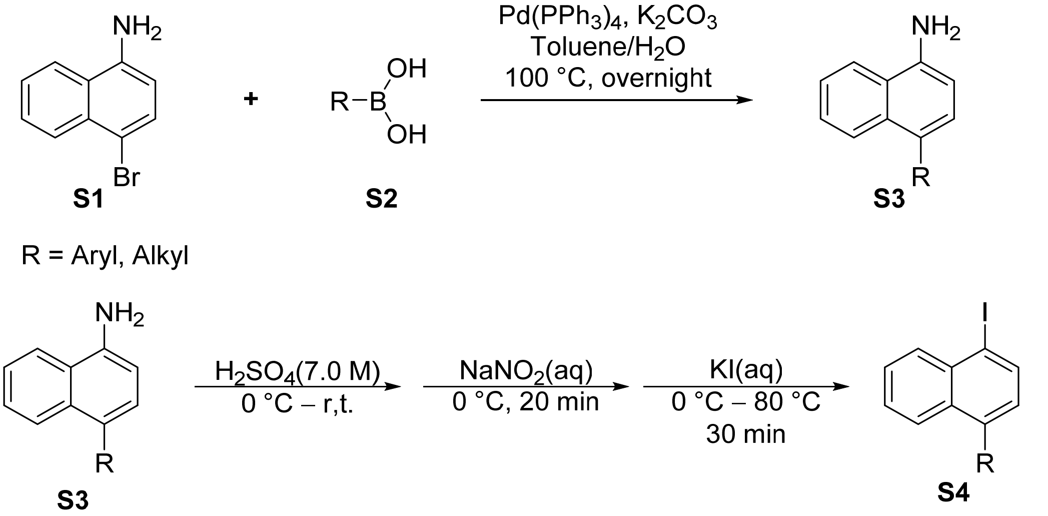

Supplementary Fig. 1 | Synthesis of aryl iodide S4

Under the protection of nitrogen atmosphere, a 100 ml round bottom flask was charged with 4-bromonaphthalen-1-amine **S1** (10 mmol), $Pd(PPh_3)_4$ (5 mol%), boronic acid **S2** (11 mmol, 1.1 eq), K_2CO_3 (3.0 eq.) in H_2O (10 mL), toluene (30 mL). The reaction mixture was stirred at reflux for 12 h. After cooling to room temperature, the reaction mixture was diluted with H_2O (30 mL) and extracted with EtOAc (3 × 40 mL). Organic layer was dried over Na_2SO_4, filtered, concentrated, and the residue was purified by silica gel column chromatography.

Aryl iodide **S4** was synthesized by corresponding 1-aminonaphthalene **S3** through the known literature procedure. [2]

Sulfuric acid solution (10 mL, 7.0 M) was added to 50 mL round-bottom flask with crude product 1-aminonaphthalene **S3** (2 mmol), and the mixture was stirred magnetically at ambient temperature until the ammonium salt formed. After the mixture was cooled to 0 °C, solution of $NaNO_2$ (3 mmol) in water (1 mL) was added dropwise while the mixture changed color gradually from pink to dark brown. After stirring about 20 minutes, water solution (1 mL) of potassium iodide (6 mmol) was added dropwise. After that, the reaction mixture was heated at 80 °C for about 30 minutes until gas stopped evolving. Appropriate amount of sodium hydroxide was added until the reaction mixture was neutral. The mixture was filtered and extracted with ethyl acetate. Organic extract was evaporated and the crude product was purified by column chromatography using petroleum ether as an eluent.

Characterization data are reported as follows:

1-cyclopropyl-4-iodonaphthalene(**1b**)

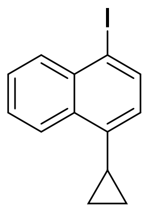

General Procedure A, colorless liquid (615 mg, 26% yield for 8 mmol scale).

<u>**^{1}H NMR (400 MHz, Chloroform-*d*)**</u> δ 8.42 – 8.35 (m, 1H), 8.16 – 8.08 (m, 1H), 7.99 (d, *J* = 7.5 Hz, 1H), 7.59 (dt, *J* = 7.4, 3.7 Hz, 2H), 6.99 (d, *J* = 7.5 Hz, 1H), 2.31 (ddd, *J* = 14.0, 8.6, 5.5 Hz, 1H), 1.13 – 1.00 (m, 2H), 0.84 – 0.67 (m, 2H).

<u>**^{13}C NMR (101 MHz, Chloroform-*d*)**</u> δ 140.6, 137.2, 134.5, 134.1, 132.9, 127.5, 126.7, 125.4, 125.2, 97.6, 13.4, 6.7.

<u>HRMS (EI-TOF)</u> calcd for $C_{13}H_{11}I$ ([M]): 293.9905, found: 293.9907.

1-(4-(4-iodonaphthalen-1-yl)phenyl)ethan-1-one(**1c**)

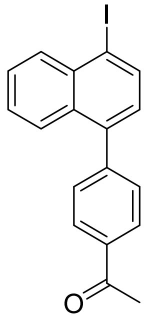

General Procedure A, yellow solid (1.42 g, 48% yield for 8 mmol scale). mp: 153.8-155 ºC.

<u>**^{1}H NMR (400 MHz, Chloroform-*d*)**</u> δ 8.18 (dd, *J* = 16.6, 8.0 Hz, 2H), 8.09 (d, *J* = 8.0 Hz, 2H), 7.76 (d, *J* = 8.4 Hz, 1H), 7.59 (m, 3H), 7.53 – 7.43 (m, 1H), 7.12 (d, *J* = 7.5 Hz, 1H), 2.69 (s, 3H).

<u>**^{13}C NMR (101 MHz, Chloroform-*d*)**</u> δ 197.9, 145.0, 140.2, 137.1, 136.3, 134.5, 132.9, 132.1, 130.4, 128.6, 128.0, 127.9, 127.3, 126.5, 100.1, 26.9.

<u>HRMS (ESI-TOF)</u> calcd for $C_{18}H_{13}INaO^+$ ([M+Na]$^+$): 394.9903, found: 394.9906.

4-(4-iodonaphthalen-1-yl)benzaldehyde(**1d**)

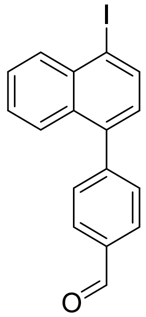

General Procedure A, yellow solid (200 mg, 7% yield for 8 mmol scale). mp: 124-125 ºC.

^{1}H NMR (400 MHz, Chloroform-*d*) δ 10.12 (s, 1H), 8.19 (dd, *J* = 15.8, 8.0 Hz, 2H), 8.02 (d, *J* = 7.9 Hz, 2H), 7.75 (d, *J* = 8.4 Hz, 1H), 7.67 – 7.57 (m, 3H), 7.49 (ddd, *J* = 8.2, 6.8, 1.2 Hz, 1H), 7.13 (d, *J* = 7.5 Hz, 1H).
^{13}C NMR (101 MHz, Chloroform-*d*) δ 192.1, 146.4, 140.0, 137.1, 135.6, 134.5, 132.9, 132.0, 130.8, 129.9, 128.1, 127.9, 127.5, 126.4, 100.3.
HRMS (ESI-TOF) calcd for $C_{17}H_{11}INaO^{+}$ ($[M+Na]^{+}$): 380.9747, found: 380.9751.

4-(4-iodonaphthalen-1-yl)benzonitrile(**1e**)

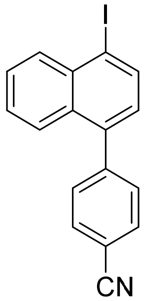

General Procedure A, yellow solid (997 mg, 35% yield for 8 mmol scale). mp: 164-165 ºC.
^{1}H NMR (400 MHz, Chloroform-*d*) δ 8.21 (d, *J* = 8.5 Hz, 1H), 8.16 (d, *J* = 7.5 Hz, 1H), 7.79 (d, *J* = 8.1 Hz, 2H), 7.69 (d, *J* = 8.4 Hz, 1H), 7.64 – 7.55 (m, 3H), 7.50 (ddd, *J* = 8.2, 6.7, 1.2 Hz, 1H), 7.09 (d, *J* = 7.5 Hz, 1H).
^{13}C NMR (101 MHz, Chloroform-*d*) δ 144.9, 139.4, 137.1, 134.6, 133.0, 132.4, 131.8, 130.9, 128.1, 128.0, 127.6, 126.1, 118.9, 111.6, 100.6.
HRMS (EI-TOF) calcd for $C_{17}H_{10}IN$ ([M]): 354.9858, found: 354.9859.

1-iodo-4-(4-nitrophenyl)naphthalene(**1f**)

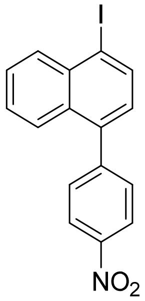

General Procedure A, yellow foam (1.0 g, 33% yield for 8 mmol scale).
^{1}H NMR (400 MHz, Chloroform-*d*) δ 8.36 (d, *J* = 8.7 Hz, 2H), 8.20 (dd, *J* = 16.1, 8.0 Hz, 2H), 7.70 (d, *J* = 8.4 Hz, 1H), 7.66 – 7.59 (m, 3H), 7.51 (ddd, *J* = 8.3, 6.8, 1.3 Hz, 1H), 7.12 (d, *J* = 7.5 Hz, 1H).
^{13}C NMR (101 MHz, Chloroform-*d*) δ 147.5, 146.9, 138.9, 137.1, 134.6, 133.1, 131.7, 131.0, 128.2, 128.0, 127.7, 126.1, 123.8, 100.8.
HRMS (ESI-TOF) calcd for $C_{16}H_{10}INNaO_2^{+}$ ($[M+Na]^{+}$): 397.9648, found: 397.9645.

1-iodo-4-(4-(trifluoromethyl)phenyl)naphthalene(**1g**)

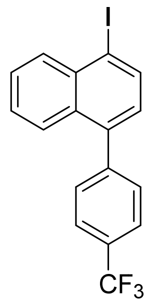

General Procedure A, yellow foam (2.19 g, 69% yield for 8 mmol scale).

^{1}H NMR (400 MHz, Chloroform-*d*) δ 8.19 (dd, *J* = 18.0, 8.0 Hz, 2H), 7.75 (dd, *J* = 11.1, 8.3 Hz, 3H), 7.67 – 7.56 (m, 3H), 7.53 – 7.43 (m, 1H), 7.11 (d, *J* = 7.5 Hz, 1H).

^{13}C NMR (101 MHz, Chloroform-*d*) δ 143.7, 139.9, 137.1, 134.5, 132.9, 132.1, 130.5, 129.9 (q, J_{CF} = 32.7 Hz), 128.0, 128.0, 127.4, 126.4, 125.5 (q, J_{CF} = 3.8 Hz), 124.3 (q, J_{CF} = 272.1 Hz), 100.1.

^{19}F NMR (376 MHz, Chloroform-*d*) δ -62.4.

HRMS (EI-TOF) calcd for $C_{17}H_{10}F_3I$ ([M]): 397.9779, found: 397.9777.

1-iodo-4-(4-(trifluoromethoxy)phenyl)naphthalene(**1h**)

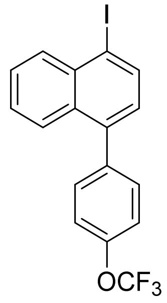

General Procedure A, white solid (360 mg, 11% yield for 8 mmol scale). mp: 68-70 °C.

^{1}H NMR (400 MHz, Chloroform-*d*) δ 8.20 (d, *J* = 8.5 Hz, 1H), 8.15 (d, *J* = 7.5 Hz, 1H), 7.77 (d, *J* = 8.4 Hz, 1H), 7.61 (ddd, *J* = 8.4, 6.9, 1.3 Hz, 1H), 7.49 (m, 3H), 7.35 (d, *J* = 8.2 Hz, 2H), 7.11 (d, *J* = 7.5 Hz, 1H).

^{13}C NMR (101 MHz, Chloroform-*d*) δ 148.9 (q, J_{CF} = 2.0 Hz), 134.0, 138.7, 137.1, 134.5, 132.8, 132.4, 131.5, 128.1, 128.0, 127.3, 126.5, 121.0, 120.7 (q, J_{CF} = 257.3 Hz), 99.7.

^{19}F NMR (376 MHz, Chloroform-*d*) δ -57.7.

HRMS (EI-TOF) calcd for $C_{17}H_{10}F_3IO$ ([M]): 413.9728, found: 413.9729.

1-iodo-4-(3-(trifluoromethyl)phenyl)naphthalene(**1i**)

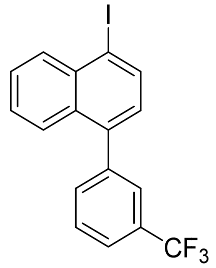

General Procedure A, yellow liquid (1.42 g, 45% yield for 8 mmol scale).
^{1}H NMR (400 MHz, Chloroform-*d*) δ 8.21 (dd, J = 8.6, 1.2 Hz, 1H), 8.16 (d, J = 7.5 Hz, 1H), 7.72 (d, J = 8.9 Hz, 3H), 7.63 (m, 3H), 7.50 (ddd, J = 8.3, 6.9, 1.3 Hz, 1H), 7.12 (d, J = 7.5 Hz, 1H).
^{13}C NMR (101 MHz, Chloroform-*d*) δ 140.8, 139.8, 137.1, 134.5, 133.5, 132.9, 132.2, 131.0 (q, J_{CF} = 32.2 Hz), 129.0, 128.1, 128.0, 127.5, 126.8 (q, J_{CF} = 3.8 Hz), 126.3, 124.6 (q, J_{CF} = 3.8 Hz), 124.2 (q, J_{CF} = 272.4 Hz), 100.0.
^{19}F NMR (376 MHz, Chloroform-*d*) δ -62.5.
HRMS (EI-TOF) calcd for $C_{17}H_{10}F_3I$ ([M]): 397.9779, found: 397.9774.

1-(3-(4-iodonaphthalen-1-yl)phenyl)ethan-1-one(**1j**)

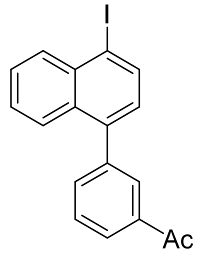

General Procedure A, yellow foam (1.06 g, 36% yield for 8 mmol scale).
^{1}H NMR (400 MHz, Chloroform-*d*) δ 8.20 (d, J = 8.5 Hz, 1H), 8.16 (d, J = 7.5 Hz, 1H), 8.07 – 8.01 (m, 2H), 7.73 (d, J = 8.4 Hz, 1H), 7.67 – 7.64 (m, 1H), 7.60 (t, J = 7.9 Hz, 2H), 7.48 (ddd, J = 8.2, 6.8, 1.2 Hz, 1H), 7.13 (d, J = 7.5 Hz, 1H), 2.65 (s, 3H).
^{13}C NMR (101 MHz, Chloroform-*d*) δ 198.1, 140.5, 140.3, 137.4, 137.1, 134.7, 134.5, 132.9, 132.3, 129.9, 128.8, 128.1, 127.9, 127.6, 127.3, 126.5, 99.8, 26.9.
HRMS (ESI-TOF) calcd for $C_{18}H_{13}INaO^+$ ($[M+Na]^+$): 394.9903, found: 394.9903.

1-(3-fluorophenyl)-4-iodonaphthalene(**1k**)

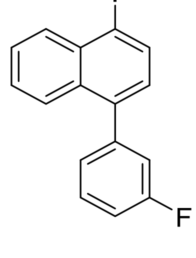

General Procedure A, yellow solid (2.07 g, 74% yield for 8 mmol scale). mp: 40-42 ºC.

^{1}H NMR (400 MHz, Chloroform-*d*) δ 8.19 (d, *J* = 9.0 Hz, 1H), 8.14 (d, *J* = 7.5 Hz, 1H), 7.80 (d, *J* = 8.9 Hz, 1H), 7.60 (ddd, *J* = 8.4, 6.8, 1.3 Hz, 1H), 7.52 – 7.40 (m, 2H), 7.27 – 7.21 (m, 1H), 7.17 (ddd, *J* = 10.9, 5.0, 2.3 Hz, 2H), 7.12 (d, *J* = 7.5 Hz, 1H).
^{13}C NMR (101 MHz, Chloroform-*d*) δ 162.8 (d, J_{CF} = 246.8 Hz), 142.1 (d, J_{CF} = 7.8 Hz), 140.1, 137.1, 134.5, 132.8, 132.2, 130.0 (d, J_{CF} = 8.4 Hz), 127.9, 127.9, 127.3, 126.6, 125.9 (d, J_{CF} = 3.0 Hz), 117.1 (d, J_{CF} = 21.6 Hz), 114.6 (d, J_{CF} = 20.9 Hz), 99.7.
^{19}F NMR (376 MHz, Chloroform-*d*) δ -113.1.
HRMS (EI-TOF) calcd for $C_{16}H_{10}FI$ ([M]): 347.9811, found: 347.9813.

4-(4-(4-iodonaphthalen-1-yl)phenyl)morpholine(**1l**)

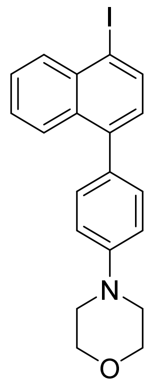

General Procedure A, orange solid (230 mg, 7% yield for 8 mmol scale). mp: 183-185 °C.
^{1}H NMR (400 MHz, Chloroform-*d*) δ 8.17 (d, *J* = 8.5 Hz, 1H), 8.12 (d, *J* = 7.5 Hz, 1H), 7.90 (d, *J* = 8.4 Hz, 1H), 7.58 (ddd, *J* = 8.4, 6.8, 1.3 Hz, 1H), 7.46 (ddd, *J* = 8.2, 6.8, 1.3 Hz, 1H), 7.38 (d, *J* = 8.6 Hz, 2H), 7.11 (d, *J* = 7.5 Hz, 1H), 7.03 (d, *J* = 8.3 Hz, 2H), 4.01 – 3.86 （t, *J* = 4.8 Hz, 4H), 3.26 (t, *J* = 4.8 Hz, 4H).
^{13}C NMR (101 MHz, Chloroform-*d*) δ 150.7, 141.3, 137.2, 134.5, 132.8, 132.6, 131.3, 131.0, 128.0, 127.7, 127.0, 126.8, 115.3, 98.5, 67.1, 49.2.
HRMS (ESI-TOF) calcd for $C_{20}H_{19}INO^{+}$ ($[M+H]^{+}$): 416.0506, found: 416.0506.

2-fluoro-5-(4-iodonaphthalen-1-yl)pyridine(**1m**)

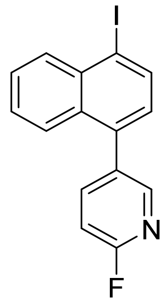

General Procedure A, brown solid (678 mg, 24% yield for 8 mmol scale). mp: 90-91.5 °C.
^{1}H NMR (400 MHz, Chloroform-*d*) δ 8.31 (d, *J* = 2.4 Hz, 1H), 8.21 (d, *J* = 8.5 Hz, 1H), 8.16 (d, *J* = 7.5 Hz, 1H), 7.88 (td, *J* = 8.0, 2.5 Hz, 1H), 7.68 (d, *J* = 8.4 Hz, 1H), 7.65 – 7.59 (m, 1H), 7.55 – 7.48 (m, 1H), 7.13 – 7.06 (m, 2H).

<u>^{13}C NMR (101 MHz, Chloroform-*d*)</u> δ 163.3 (d, J_{CF} = 240.2 Hz), 148.2 (d, J_{CF} = 14.8 Hz), 142.6 (d, J_{CF} = 8.0 Hz), 137.1, 136.2, 134.6, 133.6 (d, J_{CF} = 4.7 Hz), 133.1, 132.3, 128.4, 128.2, 127.7, 125.9, 109.4 (d, J_{CF} = 37.5 Hz), 100.5.
<u>^{19}F NMR (376 MHz, Chloroform-*d*)</u> δ -69.2.
<u>HRMS (ESI-TOF)</u> calcd for $C_{15}H_{10}FIN^+$ ($[M+H]^+$): 349.9836, found: 349.9836.

3-(4-iodonaphthalen-1-yl)thiophene(**1n**)

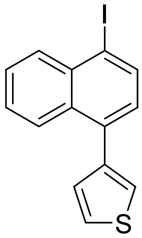

General Procedure A, white solid (324 mg, 20% yield for 5 mmol scale). mp: 77-79 ºC.
<u>^{1}H NMR (400 MHz, Chloroform-*d*)</u> δ 8.18 (dd, *J* = 8.5, 1.1 Hz, 1H), 8.12 (d, *J* = 7.5 Hz, 1H), 7.97 (dd, *J* = 8.5, 1.2 Hz, 1H), 7.59 (ddd, *J* = 8.3, 6.8, 1.3 Hz, 1H), 7.53 – 7.44 (m, 2H), 7.39 (dd, *J* = 3.1, 1.2 Hz, 1H), 7.27 (dd, *J* = 4.9, 1.2 Hz, 1H), 7.19 (d, *J* = 7.6 Hz, 1H).
<u>^{13}C NMR (101 MHz, Chloroform-*d*)</u> δ 140.3, 137.2, 136.2, 134.5, 132.7, 129.6, 128.0, 127.8, 127.1, 126.7, 125.8, 124.0, 99.2.
<u>HRMS (EI-TOF)</u> calcd for $C_{14}H_9IS$ ([M]): 335.9470, found: 335.9463.

2-(4-iodonaphthalen-1-yl)thiophene(**1o**)

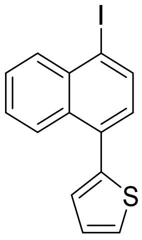

General Procedure A, yellow solid (160 mg, 10% yield for 4.8 mmol scale). mp: 49-50 ºC.
<u>^{1}H NMR (400 MHz, Chloroform-*d*)</u> δ 8.17 (ddd, *J* = 7.7, 6.0, 1.3 Hz, 2H), 8.12 (d, *J* = 7.5 Hz, 1H), 7.61 (ddd, *J* = 8.3, 6.8, 1.3 Hz, 1H), 7.53 (ddd, *J* = 8.3, 6.8, 1.3 Hz, 1H), 7.46 (dd, *J* = 5.1, 1.3 Hz, 1H), 7.27 (d, *J* = 6.1 Hz, 1H), 7.24 (dd, *J* = 3.5, 1.2 Hz, 1H), 7.20 (dd, *J* = 5.1, 3.5 Hz, 1H).
<u>^{13}C NMR (101 MHz, Chloroform-*d*)</u> δ 140.8, 137.0, 134.6, 133.7, 132.8, 132.7, 129.1, 128.0, 128.0, 127.5, 127.4, 126.7, 126.2, 100.1.
<u>HRMS (EI-TOF)</u> calcd for $C_{14}H_9IS$ ([M]): 335.9470, found: 335.9465.

3-(4-iodonaphthalen-1-yl)benzo[*b*]thiophene(**1p**)

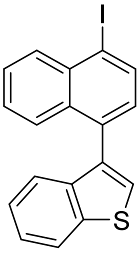

General Procedure A, yellow solid (171 mg, 9% yield for 8 mmol scale). mp: 113-115 ºC.
^{1}H NMR (400 MHz, Chloroform-*d*) δ 8.10 (t, *J* = 8.5 Hz, 2H), 7.88 (d, *J* = 8.1 Hz, 1H), 7.56 (d, *J* = 8.4 Hz, 1H), 7.50 (ddd, *J* = 8.3, 6.8, 1.2 Hz, 1H), 7.39 (s, 1H), 7.35 – 7.28 (m, 3H), 7.22 – 7.10 (m, 2H).
^{13}C NMR (101 MHz, Chloroform-*d*) δ 140.1, 139.4, 137.2, 135.5, 135.0, 134.6, 133.3, 132.7, 128.9, 128.0, 127.1, 127.0, 125.5, 124.7, 124.5, 123.4, 122.9, 100.0.
HRMS (EI-TOF) calcd for $C_{18}H_{11}IS$ ([M]): 385.9626, found: 385.9623.

2-(4-iodonaphthalen-1-yl)benzofuran(**1q**)

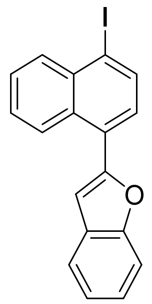

General Procedure A, yellow solid (258 mg, 14% yield for 5 mmol scale). mp: 114-115 ºC.
^{1}H NMR (400 MHz, Chloroform-*d*) δ 8.43 (dd, *J* = 7.6, 1.8 Hz, 1H), 8.29 – 8.14 (m, 2H), 7.72 – 7.52 (m, 5H), 7.39 – 7.34 (m, 1H), 7.31 (t, *J* = 7.4 Hz, 1H), 7.10 (s, 1H).
^{13}C NMR (101 MHz, Chloroform-*d*) δ 155.1, 154.7, 137.2, 134.7, 133.1, 131.4, 129.5, 129.0, 128.1, 128.1, 127.8, 126.3, 124.8, 123.2, 121.2, 111.5, 106.8, 101.6.
HRMS (ESI-TOF) calcd for $C_{18}H_{12}OI^+$ ($[M+H]^+$): 370.9924, found: 370.9924.

2-(4-iodonaphthalen-1-yl)furan(**1r**)

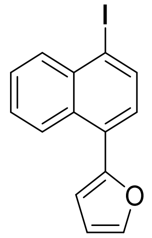

General Procedure A, orange solid (307 mg, 20% yield for 8 mmol scale). mp: 64.8-66 ºC.
^{1}H NMR (400 MHz, Chloroform-*d*) δ 8.17 (d, *J* = 8.4 Hz, 1H), 8.09 (dd, *J* = 8.2, 6.6 Hz, 2H), 7.65 (s, 1H), 7.63 – 7.57 (m, 2H), 7.52 (td, *J* = 7.5, 6.8, 1.2 Hz, 1H), 7.17 (d, *J* = 7.5 Hz, 1H), 6.68 (s, 1H).
^{13}C NMR (101 MHz, Chloroform-*d*) δ 143.2, 140.7, 137.2, 134.6, 132.8, 132.7, 132.1, 127.9, 127.9, 127.2, 126.5, 124.2, 112.5, 99.2.
HRMS (ESI-TOF) calcd for $C_{14}H_{10}IO^+$ ($[M+H]^+$): 320.9771, found: 320.9772.

tert-butyl 2-(4-iodonaphthalen-1-yl)-1H-pyrrole-1-carboxylate(**1s**)

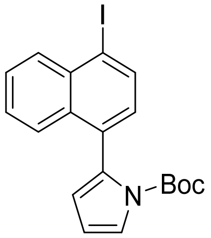

General Procedure A, yellow solid (246 mg, 12% yield for 5 mmol scale). mp: 121.4-122.2 ºC.
^{1}H NMR (400 MHz, Chloroform-*d*) δ 8.11 (dd, *J* = 10.2, 7.9 Hz, 2H), 7.60 – 7.37 (m, 4H), 7.14 (d, *J* = 7.7 Hz, 1H), 6.35 (t, *J* = 3.3 Hz, 1H), 6.28 (dd, *J* = 3.4, 1.8 Hz, 1H), 0.88 (s, 9H).
^{13}C NMR (101 MHz, Chloroform-*d*) δ 149.2, 136.9, 134.7, 134.4, 134.0, 132.4, 131.4, 128.4, 127.7, 127.1, 126.6, 122.3, 115.5, 111.0, 99.4, 83.5, 27.1.
HRMS (ESI-TOF) calcd for $C_{19}H_{18}INaO_2^+$ ($[M+Na]^+$): 442.0274, found: 442.0275.

9-(4-(4-iodonaphthalen-1-yl)phenyl)-9H-carbazole(**1t**)

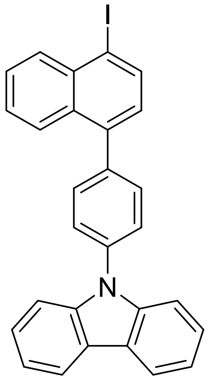

General Procedure A, yellow solid (520 mg, 35% yield for 3 mmol scale). mp: 178-179.1 ºC.
^{1}H NMR (400 MHz, Chloroform-*d*) δ 8.30 – 8.17 (m, 4H), 7.99 (d, *J* = 8.4 Hz, 1H), 7.71 (m, 4H), 7.67 – 7.62 (m, 1H), 7.58 (d, *J* = 8.1 Hz, 3H), 7.51 – 7.43 (m, 2H), 7.34 (t, *J* = 7.4 Hz, 2H), 7.25 (d, *J* = 5.8 Hz, 1H).
^{13}C NMR (101 MHz, Chloroform-*d*) δ 140.9, 140.6, 139.0, 137.3, 137.2, 134.6, 132.9, 132.5, 131.6, 128.2, 128.0, 127.3, 127.0, 126.7, 126.2, 123.6, 120.5, 120.2, 110.0, 99.6.
HRMS (ESI-TOF) calcd for $C_{28}H_{19}IN^+$ ($[M+H]^+$): 496.0557, found: 496.0553.

1-(4-chlorophenyl)-4-iodonaphthalene(**1u**)

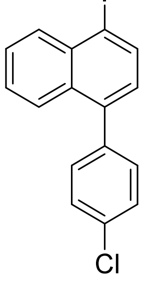

General Procedure A, off white solid (1.61 g, 55% yield for 8 mmol scale). mp: 122-124 ºC.

^{1}H NMR (400 MHz, Chloroform-*d*) δ 8.19 (d, *J* = 8.5 Hz, 1H), 8.14 (d, *J* = 7.5 Hz, 1H), 7.77 (d, *J* = 8.4 Hz, 1H), 7.60 (dd, *J* = 8.4, 6.9 Hz, 1H), 7.48 (dd, *J* = 7.8, 5.8 Hz, 3H), 7.39 (d, *J* = 8.2 Hz, 2H), 7.09 (d, *J* = 7.5 Hz, 1H).
^{13}C NMR (101 MHz, Chloroform-*d*) δ 140.2, 138.4, 137.1, 134.5, 133.8, 132.8, 132.3, 131.4, 128.7, 128.0, 127.9, 127.2, 126.6, 99.6.
HRMS (EI-TOF) calcd for $C_{16}H_{10}ClI$ ([M]): 363.9516, found: 363.9513.

4-(4-iodonaphthalen-1-yl)-*N*, *N*-diphenylaniline(**1v**)

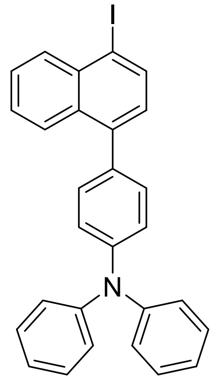

General Procedure A, white foam (0.96 g, 24% yield for 8 mmol scale).
^{1}H NMR (400 MHz, Chloroform-*d*) δ 8.18 (d, *J* = 8.5 Hz, 1H), 8.13 (d, *J* = 7.5 Hz, 1H), 7.97 (d, *J* = 8.5 Hz, 1H), 7.63 – 7.55 (m, 1H), 7.50 (t, *J* = 7.6 Hz, 1H), 7.36 – 7.28 (m, 6H), 7.19 (m, 7H), 7.07 (t, *J* = 7.3 Hz, 2H).
^{13}C NMR (101 MHz, Chloroform-*d*) δ 147.8, 147.5, 141.2, 138.3, 137.2, 134.5, 133.6, 132.7, 130.9, 129.5, 128.0, 127.7, 127.0, 126.9, 124.8, 123.3, 123.2, 98.8.
HRMS (ESI-TOF) calcd for $C_{28}H_{20}IN^{+}$ ($[M]^{+}$): 497.0635, found: 497.0630.

Synthesis of amide substrates S7 (General Procedure B)

Amide substrates **S7** was synthesized by benzoic acid derivative **S5** through the known literature procedure. [3]

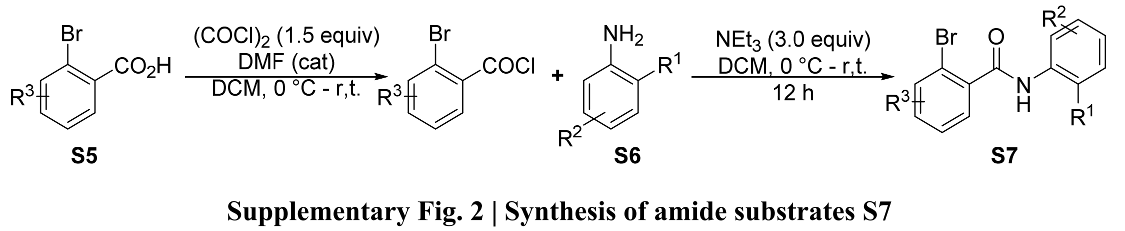

Supplementary Fig. 2 | Synthesis of amide substrates S7

To a solution of benzoic acid derivative (1.1 equiv) and DMF (one drop) in dry DCM was added oxalyl chloride (1.5 equiv) dropwise at 0 ºC. Then the reaction mixture was stirred at r.t. for another 1 h until the bubbling stopped, concentrated *in vacuo* to give benzoyl chloride, which was used in next step without further purification. The obtained benzoyl chloride was dissolved in dry DCM, then aniline derivative **S6** (1.0 equiv) and NEt_3 (3.0 equiv) were added dropwise at 0 ºC. The reaction mixture was warmed to r.t. and stirred for 12 h. H_2O was added to the mixture and the

organic layer was separated, the aqueous layer was extracted with DCM and the combined organic layers were washed with brine, dried over Na_2SO_4, filtered and concentrated *in vacuo*. The residue was purified by column chromatography on silica gel to give the amide **S7**.

Characterization data are reported as follows:

2-bromo-*N*-(2-(tert-butyl)phenyl)benzamide(**2a**)

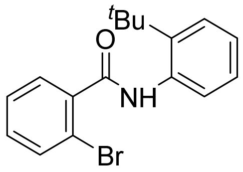

General Procedure B, white solid (4.2 g, 63% yield after recrystallization for 20 mmol scale). mp: 130-131 ºC.

^{1}H NMR (400 MHz, Chloroform-*d*) δ 7.77 (dd, *J* = 7.8, 1.5 Hz, 1H), 7.65 (t, *J* = 8.0 Hz, 2H), 7.58 (s, 1H), 7.49 – 7.41 (m, 2H), 7.33 (dtd, *J* = 13.1, 7.8, 1.7 Hz, 2H), 7.24 (td, *J* = 7.7, 1.7 Hz, 1H), 1.42 (s, 9H).

^{13}C NMR (101 MHz, Chloroform-*d*) δ 166.3, 143.5, 138.4, 134.8, 133.7, 131.7, 129.3, 128.4, 128.0, 127.2, 127.0, 126.9, 119.5, 34.9, 30.9.

HRMS (ESI-TOF) calcd for $C_{17}H_{18}BrNNaO^+$ ($[M+Na]^+$): 354.0464, found: 354.0465.

2-bromo-*N*-(2-(tert-butyl)phenyl)-4-fluorobenzamide(**2b**)

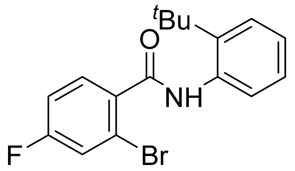

General Procedure B, white solid (1.7 g, 49% yield for 10 mmol scale). mp: 127-129 ºC.

^{1}H NMR (400 MHz, Chloroform-*d*) δ 7.74 – 7.65 (m, 2H), 7.62 (s, 1H), 7.48 – 7.36 (m, 2H), 7.30 (t, *J* = 7.5 Hz, 1H), 7.24 (t, *J* = 7.2 Hz, 1H), 7.18 – 7.11 (m, 1H), 1.42 (s, 9H).

^{13}C NMR (101 MHz, Chloroform-*d*) δ 165.4, 163.2 (d, J_{CF} = 254.9 Hz), 143.5, 134.7, 134.5 (d, J_{CF} = 3.3 Hz), 131.1 (d, J_{CF} = 9.0 Hz), 128.4, 127.2, 127.0 (d, J_{CF} = 15.9 Hz), 121.3, 121.0, 120.2 (d, J_{CF} = 9.9 Hz), 115.4 (d, J_{CF} = 21.4 Hz), 34.9, 31.0.

^{19}F NMR (376 MHz, Chloroform-*d*) δ -107.5.

HRMS (ESI-TOF) calcd for $C_{17}H_{17}BrFNNaO^+$ ($[M+Na]^+$): 372.0370, found: 372.0372.

2-bromo-*N*-(2-(tert-butyl)phenyl)-4-methylbenzamide(**2c**)

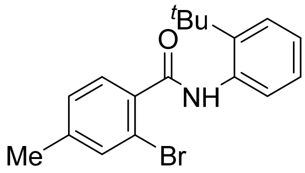

General Procedure B, white solid (2.4 g, 69% yield after recrystallization for 10 mmol scale). mp: 94-95 ºC.

^{1}H NMR (400 MHz, Chloroform-*d*) δ 7.76 (d, *J* = 7.9 Hz, 1H), 7.63 (s, 1H), 7.57 (d, *J* = 7.8 Hz, 1H), 7.49 (s, 1H), 7.44 (d, *J* = 8.0 Hz, 1H), 7.27 (m, 3H), 2.39 (s, 3H), 1.42 (s, 9H).

^{13}C NMR (101 MHz, Chloroform-*d*) δ 166.2, 143.4, 142.4, 135.3, 134.9, 134.2, 129.4, 128.7, 128.4, 127.1, 126.8, 119.3, 34.8, 30.9, 21.2.

HRMS (ESI-TOF) calcd for $C_{18}H_{20}BrNNaO^+$ ($[M+Na]^+$): 368.0620, found: 368.0623.

2-bromo-*N*-(2-(tert-butyl)phenyl)-5-nitrobenzamide(**2d**)

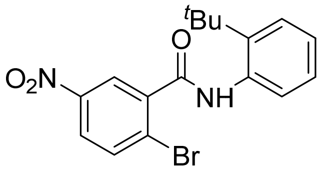

General Procedure B, white solid (1.1 g, 29% yield for 10 mmol scale). mp: 182-183 ºC.

^{1}H NMR (400 MHz, Chloroform-*d*) δ 8.45 (d, *J* = 2.8 Hz, 1H), 8.17 (dd, *J* = 8.8, 2.7 Hz, 1H), 7.87 (d, *J* = 8.7 Hz, 1H), 7.69 (d, *J* = 7.7 Hz, 1H), 7.64 (s, 1H), 7.46 (d, *J* = 7.7 Hz, 1H), 7.34 – 7.25 (m, 2H), 1.43 (s, 9H).

^{13}C NMR (101 MHz, Chloroform-*d*) δ 164.2, 147.3, 143.8, 139.6, 135.1, 134.1, 128.5, 127.6, 127.3, 127.1, 127.0, 125.9, 124.2, 34.9, 31.0.

HRMS (ESI-TOF) calcd for $C_{17}H_{17}BrN_2NaO_3^+$ ($[M+Na]^+$): 399.0315, found: 399.0316.

2-bromo-*N*-(2-(tert-butyl)phenyl)-5-methoxybenzamide(**2e**)

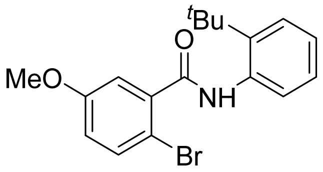

General Procedure B, white foam (1.7 g, 47% yield after recrystallization for 10 mmol scale).

^{1}H NMR (400 MHz, Chloroform-*d*) δ 7.76 (d, *J* = 7.8 Hz, 1H), 7.64 (s, 1H), 7.53 (d, *J* = 8.8 Hz, 1H), 7.44 (d, *J* = 7.9 Hz, 1H), 7.30 (t, *J* = 7.7 Hz, 1H), 7.24 (d, *J* = 7.2 Hz,1H), 7.20 (s, 1H), 6.89 (d, *J* = 8.2 Hz, 1H), 3.83 (s, 3H), 1.42 (s, 9H).

^{13}C NMR (101 MHz, Chloroform-*d*) δ 166.0, 159.2, 143.5, 138.8, 134.8, 134.6, 128.4, 127.1, 127.0, 126.9, 118.0, 114.8, 109.5, 55.8, 34.9, 30.9.

HRMS (ESI-TOF) calcd for $C_{18}H_{20}BrNNaO_2^+$ ($[M+Na]^+$): 384.0570, found: 384.0573.

2-bromo-*N*-(2-(tert-butyl)phenyl)-4,5-difluorobenzamide(**2f**)

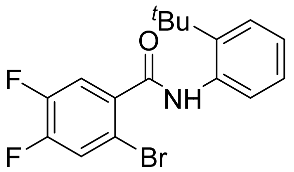

General Procedure B, off white solid (1.8 g, 49% yield after recrystallization for 10 mmol scale). mp: 142-143 ºC.

^{1}H NMR (400 MHz, Chloroform-*d*) δ 7.63 (d, *J* = 6.8 Hz, 2H), 7.49 (dt, *J* = 17.1, 8.6 Hz, 2H), 7.41 (d, *J* = 7.8 Hz, 1H), 7.23 (m, 2H), 1.38 (s, 9H).

^{13}C NMR (101 MHz, Chloroform-*d*) δ 164.1, 151.8 (dd, J_{CF} = 136.8, 12.1 Hz), 149.3 (dd, J_{CF} = 131.8, 12.8 Hz), 143.7, 134.7 (dd, J_{CF} = 9.1, 4.1 Hz), 134.4, 128.5, 127.3, 127.2, 127.0, 122.9 (d, J_{CF} = 20.3 Hz), 118.9 (d, J_{CF} = 19.8 Hz), 113.5 (dd, J_{CF} = 7.4, 4.1 Hz), 34.9, 31.0.
^{19}F NMR (376 MHz, Chloroform-*d*) δ -131.0 (d, J_{FF} = 21.0 Hz), -136.2 (d, J_{FF} = 21.1 Hz).
HRMS (ESI-TOF) calcd for $C_{17}H_{16}BrF_2NNaO^+$ ([M+Na]$^+$): 390.0276, found: 390.0278.

2-bromo-*N*-(2-(tert-butyl)phenyl)-5-fluoro-4-methylbenzamide(**2g**)

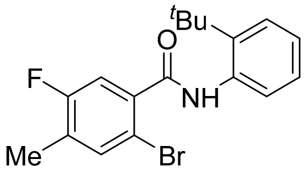

General Procedure B, white solid (2.32 g, 64% yield after recrystallization for 10 mmol scale). mp: 119-7-121.3 ºC.
^{1}H NMR (400 MHz, Chloroform-*d*) δ 7.66 (d, *J* = 6.7 Hz, 2H), 7.45 (d, *J* = 6.7 Hz, 1H), 7.41 (d, *J* = 7.9 Hz, 1H), 7.33 (d, *J* = 9.3 Hz, 1H), 7.29 – 7.17 (m, 2H), 2.28 (s, 3H), 1.38 (s, 9H).
^{13}C NMR (101 MHz, Chloroform-*d*) δ 164.9, 160.5 (d, J_{CF} = 248.1 Hz), 143.6, 136.8 (d, J_{CF} = 6.8 Hz), 136.3 (d, J_{CF} = 5.0 Hz), 134.6, 129.7 (d, J_{CF} = 18.6 Hz), 128.5, 127.2, 127.1, 126.9, 116.6 (d, J_{CF} = 25.4 Hz), 113.1 (d, J_{CF} = 3.4 Hz), 34.9, 30.9, 14.6 (d, J_{CF} = 3.1 Hz).
^{19}F NMR (376 MHz, Chloroform-*d*) δ -117.3.
HRMS (ESI-TOF) calcd for $C_{18}H_{19}BrFNNaO^+$ ([M+Na]$^+$): 386.0526, found: 386.0527.

2-bromo-*N*-(2-(tert-butyl)phenyl)-4,5-dimethoxybenzamide(**2h**)

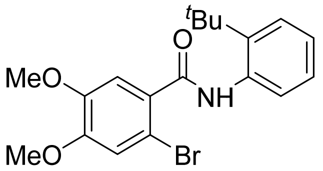

General Procedure B, white solid (1.14 g, 29% yield after recrystallization for 10 mmol scale). mp: 142-143.8 ºC.
^{1}H NMR (400 MHz, Chloroform-*d*) δ 7.91 (s, 1H), 7.70 (d, *J* = 7.8 Hz, 1H), 7.44 (d, *J* = 7.5 Hz, 1H), 7.35 – 7.27 (m, 2H), 7.22 (t, *J* = 7.6 Hz, 1H), 7.07 (s, 1H), 3.93 (s, 3H), 3.91 (s, 3H), 1.42 (s, 9H).
^{13}C NMR (101 MHz, Chloroform-*d*) δ 165.5, 151.2, 148.7, 143.5, 135.0, 129.4, 128.5, 127.1, 126.8, 126.8, 116.1, 113.0, 110.0, 56.5, 56.3, 34.8, 31.0.
HRMS (ESI-TOF) calcd for $C_{19}H_{22}BrNNaO_3^+$ ([M+Na]$^+$): 414.0675, found: 414.0676.

2-bromo-*N*-(4-bromo-2-(tert-butyl)phenyl)benzamide(**2i**)

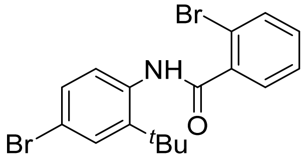

General Procedure B, yellow solid (2.4 g, 60% yield for 10 mmol scale). mp: 112.5-114 ºC.

^{1}H NMR (400 MHz, Chloroform-*d*) δ 7.66 (d, *J* = 8.2 Hz, 2H), 7.62 (d, *J* = 7.4 Hz, 1H), 7.54 (s, 2H), 7.46 – 7.40 (m, 2H), 7.35 (t, *J* = 7.5 Hz, 1H), 1.40 (s, 9H).
^{13}C NMR (101 MHz, Chloroform-*d*) δ 166.2, 145.6, 138.0, 133.9, 133.8, 131.9, 130.2, 130.1, 129.8, 129.3, 128.0, 120.5, 119.4, 35.0, 30.7.
HRMS (ESI-TOF) calcd for $C_{17}H_{17}Br_2NNaO^+$ ([M+Na]$^+$): 431.9569, found: 431.9568.

2-bromo-*N*-(2-(tert-butyl)-4-(phenylethynyl)phenyl)benzamide(**2j**)

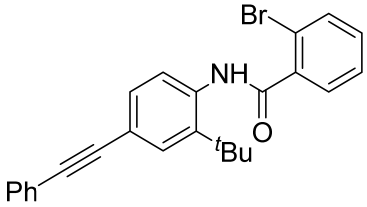

General Procedure B, yellow solid (1.37 g, 53% yield after recrystallization for 6 mmol scale). mp: 68.4-70 ºC.
^{1}H NMR (400 MHz, Chloroform-*d*) δ 7.89 (d, *J* = 8.3 Hz, 1H), 7.71 – 7.61 (m, 3H), 7.60 (s, 1H), 7.58 – 7.53 (m, 2H), 7.50 – 7.41 (m, 2H), 7.35 (m, 4H), 1.44 (s, 9H).
^{13}C NMR (101 MHz, Chloroform-*d*) δ 166.0, 142.6, 138.2, 135.1, 133.8, 131.8, 131.8, 130.4, 130.3, 129.3, 128.5, 128.4, 128.0, 127.4, 123.3, 121.3, 119.4, 89.6, 89.3, 34.8, 30.9.
HRMS (ESI-TOF) calcd for $C_{25}H_{22}BrNNaO^+$ ([M+Na]$^+$): 454.0777, found: 454.0779.

2-bromo-*N*-(2,5-di-tert-butylphenyl)benzamide(**2k**)

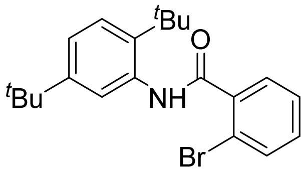

General Procedure B, white solid (3.6 g, 46% yield after recrystallization for 20 mmol scale). mp: 197-198.5 ºC.
^{1}H NMR (400 MHz, Chloroform-*d*) δ 7.78 (d, *J* = 2.2 Hz, 1H), 7.67 (dt, *J* = 8.0, 1.6 Hz, 2H), 7.60 (s, 1H), 7.43 (td, *J* = 7.5, 1.2 Hz, 1H), 7.39 – 7.31 (m, 2H), 7.24 (dd, *J* = 8.4, 2.3 Hz, 1H), 1.40 (s, 9H), 1.35 (s, 9H).
^{13}C NMR (101 MHz, Chloroform-*d*) δ 166.2, 150.0, 140.3, 138.4, 134.4, 133.8, 131.7, 129.5, 128.0, 126.5, 125.4, 123.9, 119.5, 34.5, 31.4, 31.0.
HRMS (ESI-TOF) calcd for $C_{21}H_{26}BrNNaO^+$ ([M+Na]$^+$): 410.1090, found: 410.1090.

N-(3'-acetyl-3-(tert-butyl)-[1,1'-biphenyl]-4-yl)-2-bromobenzamide(**2l**)

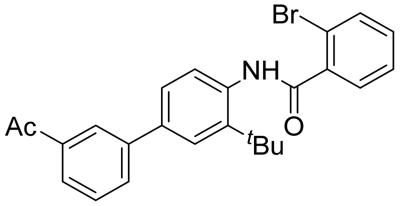

2l was synthesized by **2i** through a Suzuki coupling reaction, yellow solid (343 mg, 38% yield for 2 mmol scale). mp: 174-176 ºC.

^{1}H NMR (400 MHz, Chloroform-*d*) δ 8.16 (s, 1H), 7.92 (dd, *J* = 16.5, 8.0 Hz, 2H), 7.78 (d, *J* = 7.8 Hz, 1H), 7.70 – 7.63 (m, 4H), 7.58 – 7.51 (m, 2H), 7.45 (t, *J* = 7.5 Hz, 1H), 7.36 (td, *J* = 7.7, 2.0 Hz, 1H), 2.67 (s, 3H), 1.48 (s, 9H).

^{13}C NMR (101 MHz, Chloroform-*d*) δ 198.3, 166.4, 143.8, 141.8, 138.7, 138.2, 137.7, 134.6, 133.8, 132.8, 132.0, 131.8, 130.3, 129.3, 129.2, 128.7, 128.0, 127.4, 127.1, 126.0, 35.0, 31.0, 27.0.

HRMS (ESI-TOF) calcd for $C_{25}H_{24}BrNNaO_2^+$ ([M+Na]$^+$): 472.0883, found: 472.0884.

2-bromo-*N*-(2-(1-((tert-butyldimethylsilyl)oxy)-2-methylpropan-2-yl)phenyl)benzamide(**2m**)

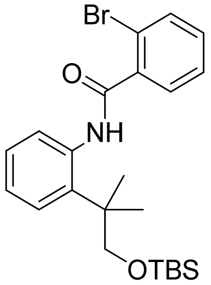

General Procedure B, off white solid (3.52 g, 76% yield after recrystallization for 10 mmol scale). mp: 68-69.8 ºC.

^{1}H NMR (400 MHz, Chloroform-*d*) δ 9.70 (s, 1H), 7.88 (dd, *J* = 8.1, 1.4 Hz, 1H), 7.61 (d, *J* = 8.0 Hz, 1H), 7.48 (dd, *J* = 7.5, 1.7 Hz, 1H), 7.41 – 7.35 (m, 2H), 7.34 – 7.27 (m, 2H), 7.17 (td, *J* = 7.7, 1.4 Hz, 1H), 3.61 (s, 2H), 1.48 (s, 6H), 0.66 (s, 9H), -0.23 (s, 6H).

^{13}C NMR (101 MHz, Chloroform-*d*) δ 166.3, 139.8, 139.3, 136.1, 133.4, 130.9, 127.7, 127.6, 127.4, 127.2, 127.1, 125.9, 120.2, 75.8, 40.4, 26.6, 26.0, 18.8, -5.7.

HRMS (ESI-TOF) calcd for $C_{23}H_{32}BrNNaO_2Si^+$ ([M+Na]$^+$): 484.1278, found: 484.1280.

2-bromo-*N*-(2-(2-((tert-butyldimethylsilyl)oxy)propan-2-yl)phenyl)benzamide(**2n**)

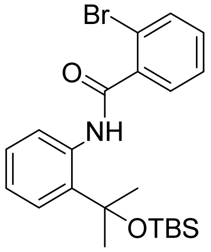

2n was prepared as described below:

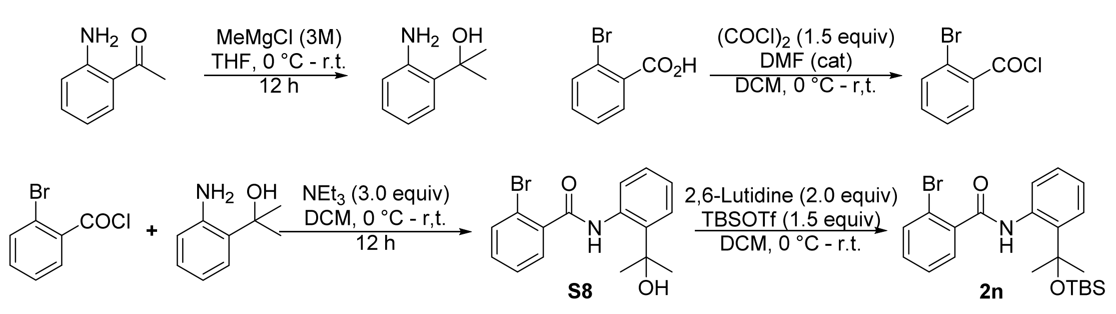

Supplementary Fig. 3 | Synthesis of amide 2n

Add a solution of methylmagnesium chloride (10 mL of 3 M solution in THF, 30 mmol, 3 equiv) to a stirred solution of 1-(2-aminophenyl)ethan-1-one (1.35 g, 10 mmol, 1 equiv.) in anhydrous THF (60 mL) under nitrogen atmosphere at 0°C. Stir the resulting mixture overnight at room temperature. Dilute it with brine and extract with EtOAc. Dry the organic layer over anhydrous Na_2SO_4, filter and concentrate under reduced pressure. Purify the crude product 2-(2-aminophenyl)propan-2-ol by flash chromatography on silica in EtOAc/DCM.
Then to a solution of 2-bromobenzoic acid (1.1 equiv) and DMF (one drop) in dry DCM was added oxalyl chloride (1.5 equiv) dropwise at 0 ºC. Then the reaction mixture was stirred at r.t. for another 1 h until the bubbling stopped, concentrated *in vacuo* to give benzoyl chloride, which was used in next step without further purification. The obtained benzoyl chloride was dissolved in dry DCM, then 2-(2-aminophenyl)propan-2-ol (6 mmol) and NEt_3 (3.0 equiv) were added dropwise at 0 ºC. The reaction mixture was warmed to r.t. and stirred for 12 h. H_2O was added to the mixture and the organic layer was separated, the aqueous layer was extracted with DCM and the combined organic layers were washed with brine, dried over Na_2SO_4, filtered and concentrated *in vacuo*. The residue was purified by column chromatography on silica gel to give the amide **S8**.
To a solution of **S8** (4 mmol, 1.0 equiv) and 2,6-Lutidine (8 mmol, 2.0 equiv) in DCM (20 mL) was added TBSOTf (6 mmol, 1.5 equiv) dropwise at 0 ºC, the mixture was warmed to r.t. and stirred for 1 h. Then the reaction was quenched with HCl (1 M), extracted with DCM, the combined organic layers were washed with brine, dried over Na_2SO_4, filtered and concentrated *in vacuo*. The residue was purified by column chromatography on silica gel gave the desired product **2n** as light yellow solid (1.69 g, 94% yield). mp: 71.8-73 ºC.
^{1}H NMR (400 MHz, Chloroform-*d*) δ 9.62 (s, 1H), 8.42 (dd, *J* = 8.2, 1.3 Hz, 1H), 7.62 (dd, *J* = 8.1, 1.1 Hz, 1H), 7.47 (dd, *J* = 7.5, 1.7 Hz, 1H), 7.41 – 7.32 (m, 2H), 7.29 (td, *J* = 7.3, 6.8, 3.3 Hz, 2H), 7.10 (td, *J* = 7.7, 1.3 Hz, 1H), 1.74 (s, 6H), 0.61 (s, 9H), -0.11 (s, 6H).
^{13}C NMR (101 MHz, Chloroform-*d*) δ 166.1, 139.7, 136.5, 136.3, 133.4, 131.0, 128.2, 127.9, 127.5, 124.8, 124.3, 123.3, 119.8, 77.1, 30.9, 25.8, 18.1, -2.2.
HRMS (ESI-TOF) calcd for $C_{22}H_{30}BrNNaO_2Si^+$ ($[M+Na]^+$): 470.1121, found: 470.1122.

2-bromo-*N*-(2-(1-hydroxy-2-methylpropan-2-yl)phenyl)benzamide(**2o**)

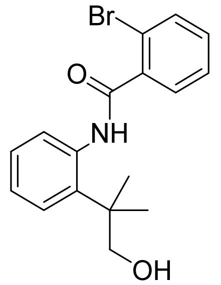

2o was prepared from **2m**.

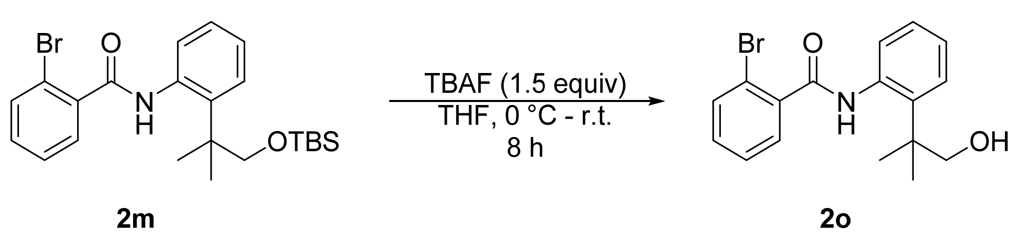

TBAF (6 mmol, 1 M in THF, 1.5 equiv) was added dropwise to a solution of **2m** (4.0 mmol, 1.0 equiv) in THF (20 mL) at 0 °C, then the mixture was warmed to r.t. and stirred for 8 h. Quenched with H_2O (10 mL), extracted with EtOAc, the combined organic layers were washed with brine, dried over Na_2SO_4, filtered and concentrated *in vacuo*. The residue was purified by column chromatography on silica gel gave the desired product **2o** as white solid (1.28 g, 93% yield). mp: 163.3-164.5 °C.

^{1}H NMR (400 MHz, Chloroform-*d*) δ 10.05 (s, 1H), 7.89 (dd, *J* = 8.0, 1.4 Hz, 1H), 7.61 (dd, *J* = 8.0, 1.1 Hz, 1H), 7.53 (dd, *J* = 7.6, 1.7 Hz, 1H), 7.42 – 7.34 (m, 2H), 7.29 (dd, *J* = 7.9, 1.7 Hz, 2H), 7.18 (td, *J* = 7.7, 1.5 Hz, 1H), 3.65 (s, 2H), 2.40 (br, 1H), 1.41 (s, 6H).

^{13}C NMR (101 MHz, Chloroform-*d*) δ 166.4, 139.2, 138.9, 136.2, 133.6, 131.2, 128.8, 127.7, 127.7, 127.4, 126.8, 126.0, 119.8, 73.9, 40.3, 26.3.

HRMS (ESI-TOF) calcd for $C_{17}H_{18}BrNNaO_2^+$ ([M+Na]$^+$): 370.0413, found: 370.0415.

2-bromo-N-(2-isopropylphenyl)benzamide (**2p**)

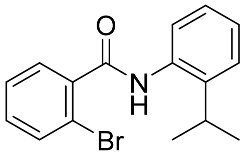

General Procedure B, white powder (4.2 g, 66% yield for 20 mmol scale).

^{1}H NMR (600 MHz, Chloroform-*d*) δ 7.85 (dd, *J* = 6.1, 6.1 Hz, 1H), 7.67 (dd, *J* = 17.5, 7.8 Hz, 1H), 7.57 (s, 1H), 7.43 (m, 1H), 7.34 (m, 2H), 7.25 (m, 2H), 3.16 (p, *J* = 6.8 Hz, 1H), 1.28 (d, *J* = 6.9 Hz, 6H).

^{13}C NMR (151 MHz, Chloroform-*d*) δ 166.2, 141.2, 138.2, 133.7, 133.7, 131.7, 130.1, 127.9, 126.7, 126.6, 125.9, 125.0, 119.3, 28.2, 23.4.

HRMS (ESI-TOF) calcd for $C_{16}H_{17}BrNO^+$ ([M+H]$^+$): 318.0488, found: 318.0489.

2-bromo-N-(naphthalen-1-yl)benzamide (**2q**)

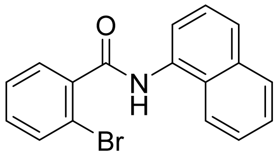

General Procedure B, white powder (2.3 g, 35% yield for 20 mmol scale).

^{1}H NMR (600 MHz, DMSO-*d*$_6$) δ 10.56 (s, 1H), 8.21 (d, *J* = 6.8 Hz, 1H), 8.04 – 7.95 (m, 1H), 7.86 (d, *J* = 8.2 Hz, 1H), 7.76 (dd, *J* = 7.7, 4.8 Hz, 2H), 7.72 (d, *J* = 7.0 Hz, 1H), 7.57 (m, 4H), 7.48 – 7.42 (m, 1H).

^{13}C NMR (151 MHz, DMSO-*d*$_6$) δ 167.3, 139.8, 134.3, 133.6, 133.2, 131.6, 129.5, 128.9, 128.5, 128.2, 126.6, 126.5, 126.0, 123.7, 123.3, 119.5.

HRMS (ESI-TOF) calcd for $C_{17}H_{13}BrNO^+$ ([M+H]$^+$): 326.0175, found: 326.0174.

2.2 Optimization of Reaction Conditions

Supplementary Table 1 | Further optimization of other reaction parameters

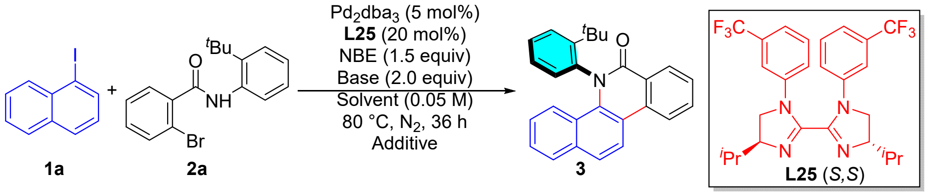

entry[a]	Additive	Solvent	Base	yield[b]	ee[c]
1	none	Toluene	Cs_2CO_3	76% (78%[d])	23% (60%[d])
2	none	Toluene + H_2O (4 μL)	Cs_2CO_3	54%	66%
3	none	Toluene + H_2O (8 μL)	Cs_2CO_3	65%	73%
4	none	Toluene + H_2O (16 μL)	Cs_2CO_3	75%	85%
5	4Å MS	Toluene + H_2O (16 μL)	Cs_2CO_3	53%	88%
6	4Å MS	Toluene + H_2O (16 μL)	Ag_2CO_3	93%	53%
7	4Å MS	Toluene + H_2O (60 μL)	Ag_2CO_3	91%	66%
8	4Å MS	Toluene + H_2O (100 μL)	Ag_2CO_3	94%	78%
9	4Å MS	Toluene + H_2O (200 μL)	Ag_2CO_3	96%	88%
10	4Å MS	Toluene + H_2O (200 μL)	AgOAc	trace	--
11	4Å MS	Toluene + H_2O (200 μL)	Ag_3PO_4	93%	82%
12	4Å MS	Toluene + H_2O (200 μL)	$AgNO_3$	45%	81%
13	4Å MS	Toluene + H_2O (200 μL)	Ag_2SO_4	95%	91%
14	4Å MS	DCE + H_2O (200 μL)	Ag_2SO_4	75%	91%
15	4Å MS	THF + H_2O (200 μL)	Ag_2SO_4	61%	74%
16	4Å MS	MeCN + H_2O (200 μL)	Ag_2SO_4	15%	51%

[a] Condition: **2a** (0.1 mmol), **1a** (0.15 mmol), $Pd_2(dba)_3$ (5 mol%), NBE (0.15 mmol), Base (0.2 mmol),Solvent (0.05 M), 4Å MS (100 mg) in 80 °C, under N_2, reaction for 36 h. [b] isolated yield. [c] ee value was determined by chiral HPLC. [d] without anhydrous operation.

Supplementary Table 2 | Further screening of Ligand for Catellani-Reaction [a]

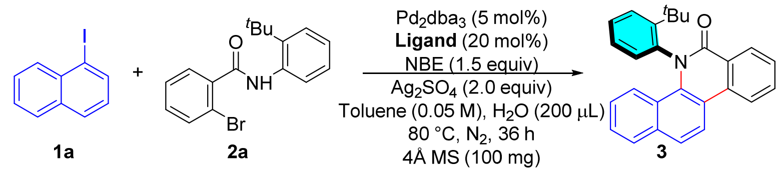

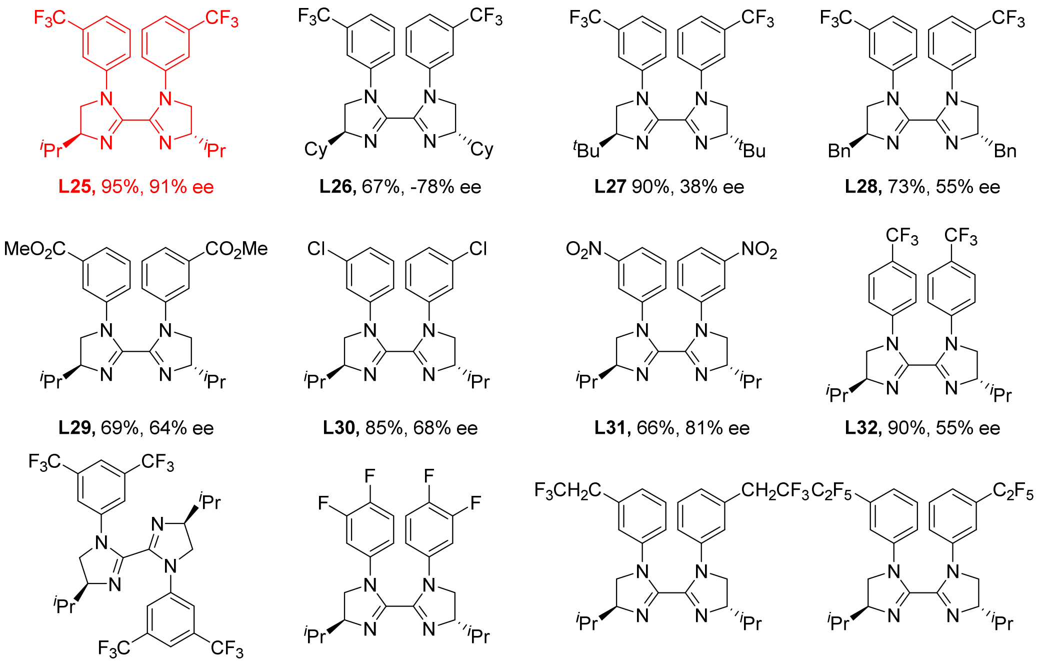

L25, 95%, 91% ee
L26, 67%, -78% ee
L27 90%, 38% ee
L28, 73%, 55% ee
L29, 69%, 64% ee
L30, 85%, 68% ee
L31, 66%, 81% ee
L32, 90%, 55% ee
L33, 70%, 5% ee
L34, 75%, 85% ee
L35, 84%, 88% ee
L36, 76%, 86% ee

[a] Condition: **2a** (0.1 mmol), **1** (0.15 mmol), $Pd_2(dba)_3$ (5 mol%), NBE (0.15 mmol), Ag_2SO_4 (0.2 mmol),Solvent (0.05 M), H_2O (200 μL) 4Å MS (100 mg) in 80 °C, reaction for 36 h.

Supplementary Table 3 | Control experiments for Catellani-Reaction

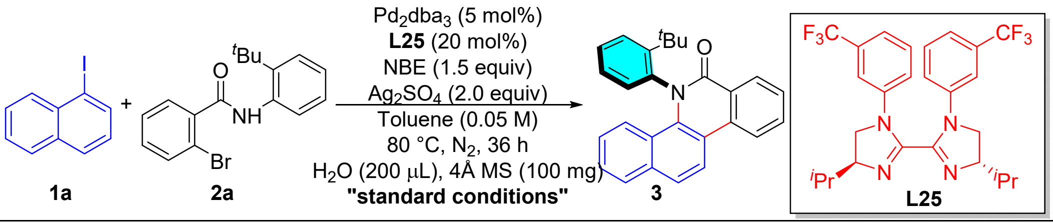

entry[a]	Change from the standard conditions	yield[b]	ee[c]
1	none	95%	91%
2	no $Pd_2(dba)_3$ and **L25**	0	--
3	no **L25**	6%	0%
4	no NBE	NR	--
5	no H_2O	29%	85%
6	no 4Å MS	32%	84%
7	no Ag_2SO_4	trace	--
8	$Pd(OAc)_2$ instead of $Pd_2(dba)_3$	92%	28%
9	Cs_2CO_3 instead of Ag_2SO_4	35%	89%
10	DMF instead of Toluene	43%	69%
11	THF instead of Toluene	61%	74%
12	DCE instead of Toluene	75%	91%
13	MeCN instead of Toluene	15%	51%
14	^{t}BuOH instead of Toluene	54%	85%

[a] Condition: **2a** (0.1 mmol), **1a** (0.15 mmol), $Pd_2(dba)_3$ (5 mol%), NBE (0.15 mmol), Ag_2SO_4 (0.2 mmol),Toluene (0.05 M), H_2O (200 µL), 4Å MS (100 mg) in 80 °C, reaction for 36 h. [b] isolated yield. [c] ee value was determined by chiral HPLC.

2.3 Atroposelective Synthesis of C−N axially chiral scaffolds via chiral BiIM ligand enabled asymmetric Catellani reaction

General procedure for BiIM ligand enabled asymmetric Catellani reaction

General Procedure C: To an oven-dried 10 mL Schlenk tube were added substrate **1** (0.15 mmol), amide **2** (0.1 mmol), $Pd_2(dba)_3$ (4.9 mg, 0.005 mmol), **L25** (10.2 mg, 0.020 mmol), Ag_2SO_4 (62.3 mg, 0.2 mmol), Toluene (2.0 mL), H_2O (0.2 mL), 4ÅMS (100 mg), NBE (15 mg, 0.15 mmol). The mixture was stirred for 36 h at 80 °C. The resulting mixture was quenched by filtered through a celite pad and concentrated in *vacuo*. The residue was purified by preparative TLC to afford the product.

General Procedure D: To an oven-dried 10 mL Schlenk tube were added substrate **1** (0.15 mmol), amide **2** (0.1 mmol), $Pd_2(dba)_3$ (4.9 mg, 0.005 mmol), **L25** (10.2 mg, 0.020 mmol), Ag_2SO_4 (62.3 mg, 0.2 mmol), DCE (2.0 mL), H_2O (0.2 mL), 4ÅMS (100 mg), NBE (15 mg, 0.15 mmol). The mixture was stirred for 36 h at 80 °C. The resulting mixture was quenched by filtered through a celite pad and concentrated in *vacuo*. The residue was purified by preparative TLC to afford the product. Characterization data for products are reported as follows:

(***S***)-5-(2-(tert-butyl)phenyl)benzo[*c*]phenanthridin-6(5H)-one(**3**)

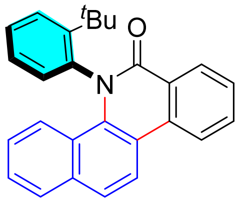

The title compound **3** was prepared according to **General Procedure C** and purified by preparative TLC in hexane/EtOAc = 10/1 as the eluent to afford a yellow oil (35 mg, 95% yield, 91% ee).

^{1}H NMR (600 MHz, Chloroform-*d*) δ 8.58 (d, *J* = 7.9 Hz, 1H), 8.41 (dd, *J* = 15.6, 8.6 Hz, 2H), 7.86 – 7.79 (m, 2H), 7.75 (d, *J* = 8.7 Hz, 2H), 7.63 (t, *J* = 7.6 Hz, 1H), 7.50 (t, *J* = 7.9 Hz, 1H), 7.36 (t, *J* = 7.5 Hz, 1H), 7.28 (t, *J* = 7.7 Hz, 1H), 7.23 (d, *J* = 8.8 Hz, 1H), 7.00 (m, 2H), 1.20 (s, 9H).

^{13}C NMR (151 MHz, Chloroform-*d*) δ 164.3, 147.5, 140.4, 135.7, 135.5, 134.8, 133.1, 131.1, 130.6, 129.2, 129.0, 129.0, 128.1, 127.8, 126.2, 126.1, 125.6, 125.3, 124.9, 124.6, 122.7, 120.8, 116.7, 36.7, 31.7.

HRMS (ESI-TOF) calcd for $C_{27}H_{23}NNaO^{+}$ ($[M+Na]^{+}$): 400.1672, found: 400.1669.

$[\alpha]_D^{20}$ = 50.4 (c = 1.02, $CHCl_3$).

The ee value was determined by HPLC analysis on a IE column (n-hexane/i-PrOH = 70/30, flow = 1.0 mL/min, 254 nm), t = 10.9 min (major), t = 9.4 min (minor). The absolute stereochemistry was assigned by analogy to compound **55**.

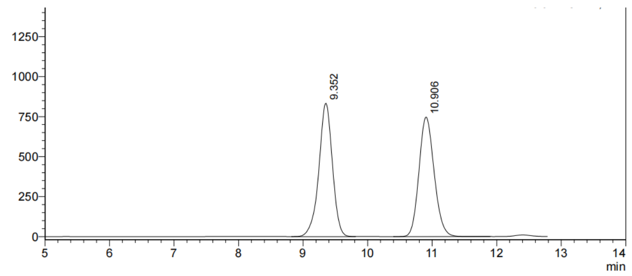

Peak	Ret. Time	Area	Height	Area%
1	9.352	12193159	833517	49.895
2	10.906	12244267	746945	50.105
Total		24437426	1580462	100.000

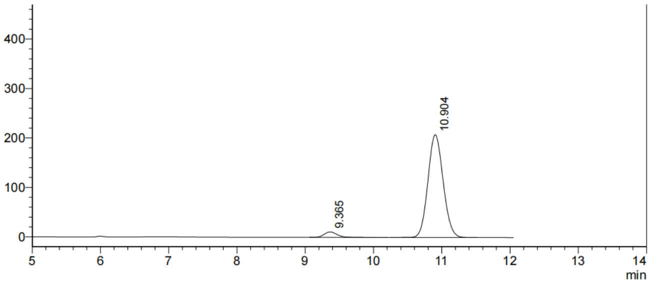

Peak	Ret. Time	Area	Height	Area%
1	9.365	148503	11054	4.405
2	10.904	3222361	207908	95.595
Total		3370864	218962	100.000

Supplementary Fig. 4 | Enantiomeric excess determination of product 3

(***S***)-5-(2-(tert-butyl)phenyl)-12-cyclopropylbenzo[*c*]phenanthridin-6(5H)-one(**4**)

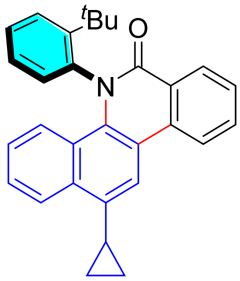

The title compound **4** was prepared according to **General Procedure C** and purified by preparative TLC in hexane/EtOAc = 15/1 as the eluent to afford a colorless oil (33 mg, 80% yield, 90% ee).

^{1}H NMR (600 MHz, Chloroform-*d*) δ 8.57 (d, *J* = 7.7 Hz, 1H), 8.50 (d, *J* = 8.3 Hz, 1H), 8.44 (d, *J* = 8.5 Hz, 1H), 8.24 (s, 1H), 7.84 (t, *J* = 7.8 Hz, 1H), 7.75 (d, *J* = 7.7 Hz, 1H), 7.61 (t, *J* = 7.6 Hz, 1H), 7.48 (t, *J* = 7.4 Hz, 1H), 7.44 (t, *J* = 7.6 Hz, 1H), 7.41 (d, *J* = 9.1 Hz, 1H), 7.25 (t, *J* = 7.7 Hz, 1H), 7.07 – 7.00 (m, 1H), 6.95 – 6.89 (m, 1H), 2.35 (td, *J* = 8.4, 4.2 Hz, 1H), 1.23 (s, 9H), 1.16 (d, *J* = 8.6 Hz, 2H), 0.89 (dd, *J* = 15.7, 5.8 Hz, 2H).

^{13}C NMR (151 MHz, Chloroform-*d*) δ 164.3, 147.5, 140.7, 135.6, 134.8, 134.8, 134.4, 133.0, 130.8, 130.5, 129.2, 128.9, 128.0, 127.8, 126.5, 126.1, 125.7, 125.2, 124.8, 124.8, 122.6, 119.7, 116.0, 36.8, 31.7, 14.2, 6.5, 6.5.

HRMS (ESI-TOF) calcd for $C_{30}H_{27}NNaO^+$ ([M+Na]$^+$): 440.1986, found: 440.1985.

$[\alpha]_D^{20}$ = 34.5 (c = 0.78, $CHCl_3$). The ee value was determined by HPLC analysis on a AD-H column (n-hexane/i-PrOH = 80/20, flow = 1.0 mL/min, 254 nm), t = 7.0 min (major), t = 6.5 min (minor). The absolute stereochemistry was assigned by analogy to compound **55**.

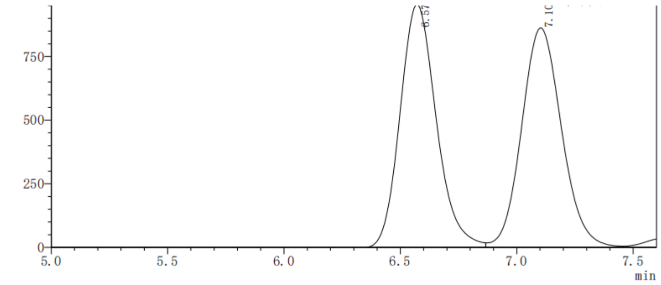

Peak	Ret. Time	Area	Height	Area%
1	6.572	10440217	958272	50.069
2	7.103	10411619	866138	49.931
Total		20851837	1824410	100.000

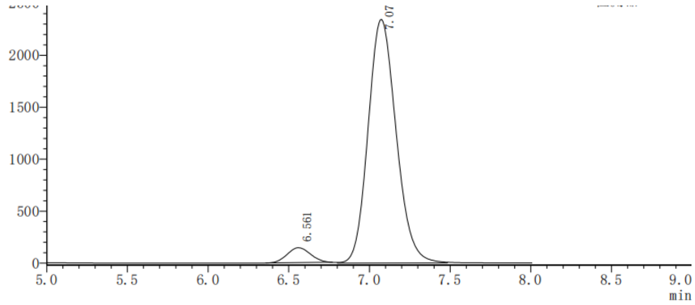

Peak	Ret. Time	Area	Height	Area%
1	6.561	1442897	143511	4.851
2	7.073	28302291	2344870	95.149
Total		29745188	2488381	100.000

Supplementary Fig. 5 | Enantiomeric excess determination of product 4

(***S***)-5-(2-(tert-butyl)phenyl)-12-methylbenzo[*c*]phenanthridin-6(5H)-one(**5**)

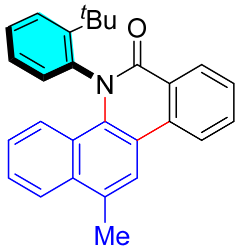

The title compound **5** was prepared according to **General Procedure C** and purified by preparative TLC in hexane/EtOAc = 10/1 as the eluent to afford a yellow oil (35 mg, 82% yield, 85% ee).
^{1}H NMR (600 MHz, Chloroform-*d*) δ 8.58 (d, *J* = 7.9 Hz, 1H), 8.43 (d, *J* = 8.3 Hz, 1H), 8.24 (s, 1H), 7.98 (d, *J* = 8.4 Hz, 1H), 7.83 (t, *J* = 7.8 Hz, 1H), 7.74 (d, *J* = 8.3 Hz, 1H), 7.61 (t, *J* = 7.6 Hz, 1H), 7.48 (t, *J* = 7.9 Hz, 1H), 7.42 (d, *J* = 7.2 Hz, 2H), 7.25 (t, *J* = 7.4 Hz, 1H), 7.05 – 6.97 (m, 1H), 6.94 (d, *J* = 7.9 Hz, 1H), 2.77 (s, 3H), 1.22 (s, 9H).

^{13}C NMR (151 MHz, Chloroform-*d*) δ 164.2, 147.5, 140.7, 134.7, 134.3, 133.0, 130.9, 130.6, 130.1, 129.2, 128.9, 128.0, 127.8, 126.6, 126.2, 125.7, 124.9, 124.8, 124.8, 122.6, 121.5, 116.2, 36.8, 31.7, 20.3.

HRMS (ESI-TOF) calcd for $C_{28}H_{25}NNaO^+$ ($[M+Na]^+$): 414.1828, found: 414.1831.

$[\alpha]_D^{20}$ = 41.2 (c = 1.08, $CHCl_3$).

The ee value was determined by HPLC analysis on a IG column (n-hexane/i-PrOH = 80/20, flow = 1.0 mL/min, 254 nm), t = 8.9 min (major), t = 7.7 min (minor). The absolute stereochemistry was assigned by analogy to compound **55**.

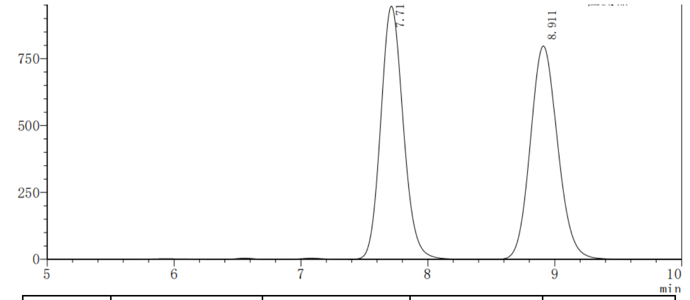

Peak	Ret. Time	Area	Height	Area%
1	7.713	11717156	946911	49.484
2	8.911	11961512	798092	50.516
Total		23678668	1745003	100.000

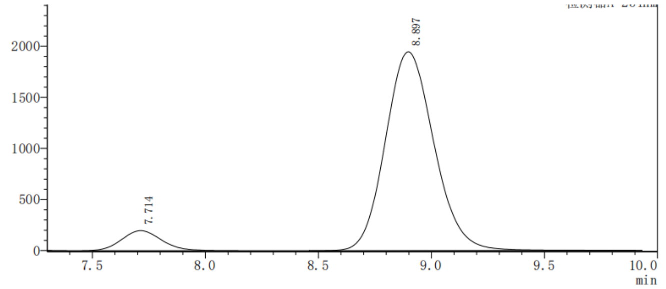

Peak	Ret. Time	Area	Height	Area%
1	7.714	2398985	196594	7.606
2	8.897	29141109	1945878	92.394
Total		31540094	214.2472	100.000

Supplementary Fig. 6 | Enantiomeric excess determination of product 5

(***S***)-5-(2-(tert-butyl)phenyl)-12-methoxybenzo[*c*]phenanthridin-6(5H)-one(**6**)

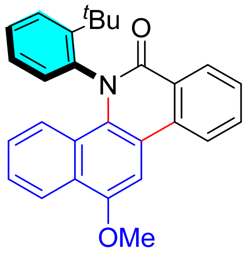

The title compound **6** was prepared according to **General Procedure D** and purified by preparative TLC in hexane/EtOAc = 10/1 as the eluent to afford a colorless oil (26 mg, 64% yield, 79% ee).

^{1}H NMR (600 MHz, Chloroform-*d*) δ 8.61 (d, *J* = 8.5 Hz, 1H), 8.37 (dd, *J* = 15.7, 8.6 Hz, 2H), 7.89 – 7.82 (m, 1H), 7.76 – 7.73 (m, 1H), 7.69 (s, 1H), 7.64 (t, *J* = 7.5 Hz, 1H), 7.52 – 7.47 (m, 1H), 7.40 (t, *J* = 7.6 Hz, 1H), 7.28 (dd, *J* = 9.0, 6.1 Hz, 2H), 7.05 (td, *J* = 7.2, 6.6, 3.7 Hz, 1H), 6.99 (d, *J* = 6.6 Hz, 1H), 4.18 (s, 3H), 1.21 (s, 9H).
^{13}C NMR (151 MHz, Chloroform-*d*) δ 163.9, 151.7, 147.5, 140.6, 134.8, 132.9, 131.1, 130.5, 130.1, 129.3, 128.9, 128.1, 127.9, 127.8, 125.9, 125.9, 125.9, 125.9, 125.7, 122.6, 122.5, 116.6, 98.1, 55.8, 36.7, 31.7.
HRMS (ESI-TOF) calcd for $C_{28}H_{25}NNaO_2^+$ ($[M+Na]^+$): 430.1778, found: 430.1778.
$[\alpha]_D^{20}$ = 23.0 (c = 0.91, $CHCl_3$).
The ee value was determined by HPLC analysis on a AS-H column (n-hexane/i-PrOH = 93/7, flow = 1.05 mL/min, 254 nm), t = 7.4 min (major), t = 6.3 min (minor). The absolute stereochemistry was assigned by analogy to compound **55**.

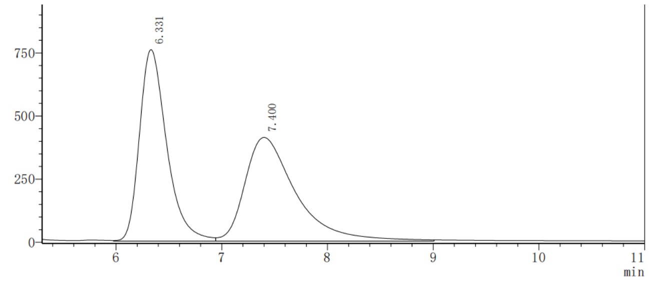

Peak	Ret. Time	Area	Height	Area%
1	6.331	13616337	758767	49.706
2	7.400	13777137	410918	50.294
Total		27393474	1169685	100.000

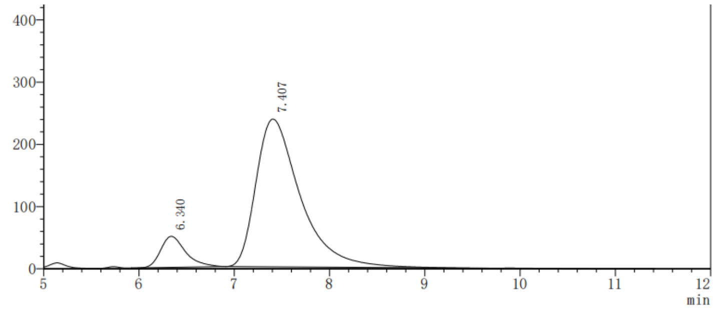

Peak	Ret. Time	Area	Height	Area%
1	6.340	915723	49983	10.631
2	7.407	7697824	237469	89.369
Total		8613548	287452	100.000

Supplementary Fig. 7 | Enantiomeric excess determination of product 6

(***S***)-5-(2-(tert-butyl)phenyl)-12-fluorobenzo[*c*]phenanthridin-6(5H)-one(**7**)

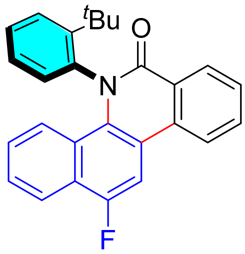

The title compound **7** was prepared according to **General Procedure D** and purified by preparative TLC in hexane/EtOAc = 10/1 as the eluent to afford a off white foam (15 mg, 38% yield, 90% ee).
<u>^{1}H NMR (600 MHz, Chloroform-*d*)</u> δ 8.59 (d, *J* = 7.7 Hz, 1H), 8.30 (d, *J* = 8.3 Hz, 1H), 8.14 (d, *J* = 8.3 Hz, 1H), 8.08 (d, *J* = 11.6 Hz, 1H), 7.90 – 7.84 (m, 1H), 7.77 – 7.75 (m, 1H), 7.66 (t, *J* = 7.5 Hz, 1H), 7.54 – 7.49 (m, 1H), 7.45 (t, *J* = 7.5 Hz, 1H), 7.30 (td, *J* = 7.6, 1.6 Hz, 1H), 7.24 (d, *J* = 11.5 Hz, 1H), 7.08 (ddd, *J* = 8.8, 6.9, 1.7 Hz, 1H), 6.99 (dd, *J* = 7.7, 1.6 Hz, 1H), 1.20 (s, 9H).
<u>^{13}C NMR (151 MHz, Chloroform-*d*)</u> δ 164.0, 155.2 (d, J_{CF} = 246.6 Hz), 147.6, 140.2, 134.2 (d, J_{CF} = 2.5 Hz), 133.3, 132.1 (d, J_{CF} = 2.6 Hz), 131.1, 130.6, 129.3, 129.1, 128.6, 127.9, 126.6, 126.3, 126.2 (d, J_{CF} = 1.7 Hz), 125.9 (d, J_{CF} = 3.9 Hz), 125.7 (d, J_{CF} = 16.2 Hz), 125.7, 122.7, 121.1 (d, J_{CF} = 6.9 Hz), 116.5 (d, J_{CF} = 8.6 Hz), 104.0 (d, J_{CF} = 23.1 Hz), 36.7, 31.7.
<u>^{19}F NMR (565 MHz, Chloroform-*d*)</u> δ -127.4.
<u>HRMS (ESI-TOF)</u> calcd for $C_{27}H_{22}NFNaO^{+}$ ($[M+Na]^{+}$): 418.1578, found: 418.1580.
$[\alpha]_D^{20}$ = 47.3 (c = 0.94, $CHCl_3$).
The ee value was determined by HPLC analysis on a AD-H column (n-hexane/i-PrOH = 80/20, flow = 1.0 mL/min, 254 nm), t = 5.3 min (major), t = 6.0 min (minor). The absolute stereochemistry was assigned by analogy to compound **55**.

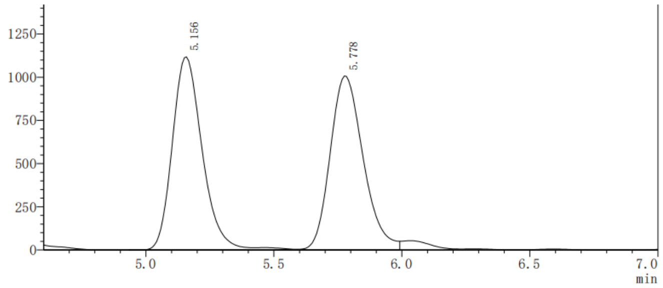

Peak	Ret. Time	Area	Height	Area%
1	5.156	9165877	1118711	50.267
2	5.778	9068386	1007784	49.733
Total		18234263	2126495	100.000

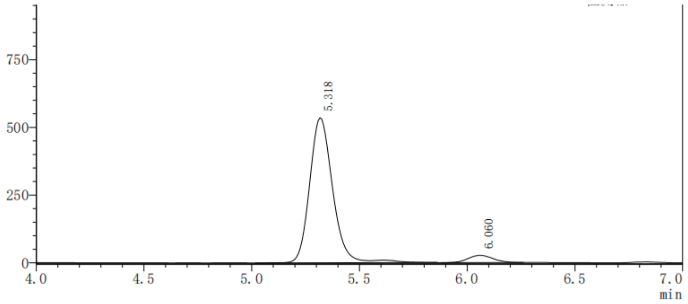

Peak	Ret. Time	Area	Height	Area%

1	5.318	3993599	535696	94.970
2	6.060	211496	26538	5.030
Total		4205095	562234	100.000

Supplementary Fig. 8 | Enantiomeric excess determination of product 7

(***S***)-12-bromo-5-(2-(tert-butyl)phenyl)benzo[*c*]phenanthridin-6(5H)-one(**8**)

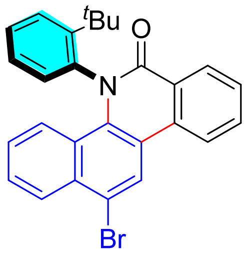

The title compound **8** was prepared according to **General Procedure D** and purified by preparative TLC in hexane/EtOAc = 10/1 as the eluent to afford a yellow foam (10 mg, 22% yield, 90% ee).
^{1}H NMR (600 MHz, Chloroform-*d*) δ 8.72 (s, 1H), 8.58 (dd, *J* = 7.8, 1.5 Hz, 1H), 8.36 (d, *J* = 8.3 Hz, 1H), 8.32 (dd, *J* = 8.4, 1.5 Hz, 1H), 7.87 (ddd, *J* = 8.4, 7.0, 1.5 Hz, 1H), 7.76 (dd, *J* = 8.3, 1.5 Hz, 1H), 7.69 – 7.62 (m, 1H), 7.49 (dddd, *J* = 15.0, 8.2, 7.0, 1.3 Hz, 2H), 7.43 (d, *J* = 9.1 Hz, 1H), 7.30 – 7.26 (m, 1H), 7.07 (ddd, *J* = 8.8, 6.8, 1.5 Hz, 1H), 6.94 (dd, *J* = 7.8, 1.6 Hz, 1H), 1.22 (s, 9H).
^{13}C NMR (151 MHz, Chloroform-*d*) δ 164.1, 147.5, 140.3, 135.3, 133.7, 133.4, 133.2, 130.9, 130.7, 129.3, 129.1, 128.7, 128.3, 127.9, 127.5, 126.5, 125.9, 125.7, 125.1, 122.6, 118.7, 117.3, 36.8, 31.7.
HRMS (ESI-TOF) calcd for $C_{27}H_{22}NBrNaO^{+}$ ($[M+Na]^{+}$): 478.0777, found: 478.0776.
$[\alpha]_D^{20}$ = 24.7 (c = 0.73, $CHCl_3$).
The ee value was determined by HPLC analysis on a IG column (n-hexane/i-PrOH = 75/25, flow = 1.1 mL/min, 254 nm), t = 7.3 min (major), t = 6.0 min (minor). The absolute stereochemistry was assigned by analogy to compound **55**.

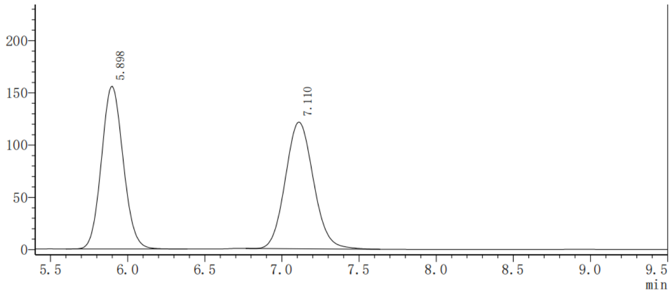

Peak	Ret. Time	Area	Height	Area%
1	5.898	1527944	155709	49.830
2	7.110	1538359	121267	50.170
Total		3066302	276975	100.000

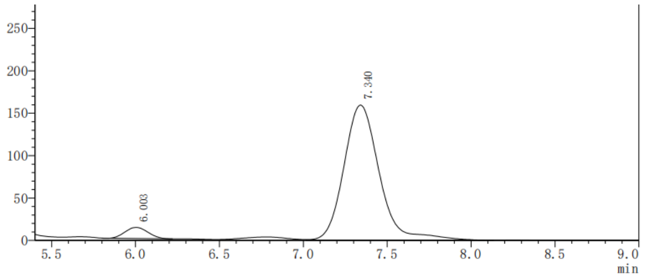

Peak	Ret. Time	Area	Height	Area%
1	6.003	124876	13137	5.014
2	7.340	2365493	159773	94.986
Total		2490369	172910	100.000

Supplementary Fig. 9 | Enantiomeric excess determination of product 8

(***S***)-5-(2-(tert-butyl)phenyl)-4-methylphenanthridin-6(5H)-one(**9**)

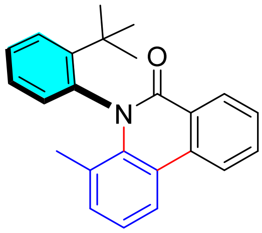

The title compound **9** was prepared according to **General Procedure C** and purified by preparative TLC in hexane/EtOAc = 10/1 as the eluent to afford a yellow oil (17 mg, 49% yield, 77% ee).

^{1}H NMR (600 MHz, Chloroform-*d*) δ 8.56 – 8.47 (m, 1H), 8.34 (dd, *J* = 8.3, 2.6 Hz, 1H), 8.27 (q, *J* = 4.3 Hz, 1H), 7.82 – 7.73 (m, 1H), 7.63 (d, *J* = 8.2 Hz, 1H), 7.59 (dt, *J* = 7.9, 3.9 Hz, 1H), 7.45 – 7.39 (m, 1H), 7.26 (td, *J* = 7.8, 3.7 Hz, 1H), 7.19 (t, *J* = 3.8 Hz, 2H), 7.12 (d, *J* = 7.8 Hz, 1H), 1.71 (s, 3H), 1.22 (s, 9H).

^{13}C NMR (151 MHz, Chloroform-*d*) δ 163.9, 148.1, 139.1, 138.2, 135.4, 134.8, 132.9, 132.7, 129.7, 129.1, 128.9, 128.1, 126.6, 126.4, 125.6, 122.8, 122.3, 120.5, 36.6, 31.7, 23.6.

HRMS (ESI-TOF) calcd for $C_{24}H_{23}NNaO^{+}$ ([M+Na]$^{+}$): 364.1672, found: 364.1674.

$[\alpha]_D^{20}$ = 52.7 (c = 0.5, $CHCl_3$).

The ee value was determined by HPLC analysis on a AD-H column (n-hexane/i-PrOH = 80/20, flow = 1.05 mL/min, 254 nm), t = 5.6 min (major), t = 5.1 min (minor). The absolute stereochemistry was assigned by analogy to compound **55**.

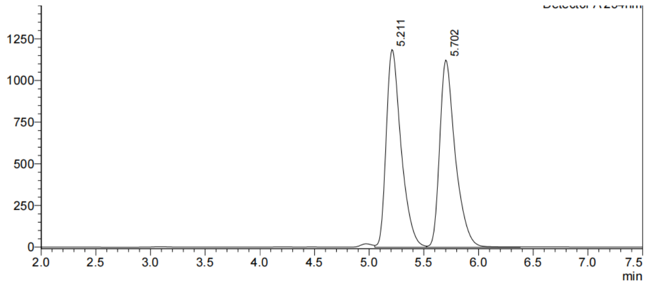

Peak	Ret. Time	Area	Height	Area%
1	5.211	11226674	1186062	50.117
2	5.702	11174159	1122839	49.883
Total		22400833	2308901	100.000

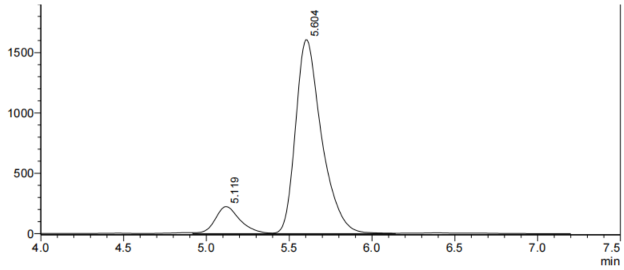

Peak	Ret. Time	Area	Height	Area%
1	5.119	2346678	224221	11.761
2	5.604	17606985	1607176	88.239
Total		19953664	1831397	100.000

Supplementary Fig. 10 | Enantiomeric excess determination of product 9

(***S***)-5-(2-(2-((tert-butyldimethylsilyl)oxy)propan-2-yl)phenyl)-4-methylphenanthridin-6(5H)-one(**10**)

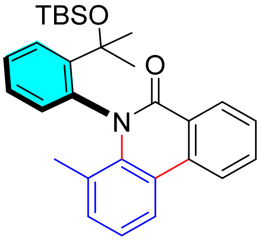

The title compound **10** was prepared according to **General Procedure C** and purified by preparative TLC in hexane/EtOAc = 10/1 as the eluent to afford a yellow oil (37 mg, 81% yield, 81% ee).

^{1}H NMR (400 MHz, Chloroform-*d*) δ 8.50 (dd, *J* = 8.0, 1.4 Hz, 1H), 8.33 (d, *J* = 8.3 Hz, 1H), 8.26 (dd, *J* = 6.3, 3.4 Hz, 1H), 7.93 (dd, *J* = 8.2, 1.6 Hz, 1H), 7.79 (ddd, *J* = 8.5, 7.1, 1.5 Hz, 1H), 7.59 (t, *J* = 7.7 Hz, 1H), 7.49 – 7.44 (m, 1H), 7.29 (td, *J* = 7.6, 1.6 Hz, 1H), 7.19 (d, *J* = 6.3 Hz, 2H), 7.11 (dd, *J* = 7.8, 1.5 Hz, 1H), 1.70 (s, 3H), 1.45 (s, 3H), 1.32 (s, 3H), 0.86 (s, 9H), 0.09 (s, 3H), -0.01 (s, 3H).

^{13}C NMR (101 MHz, Chloroform-*d*) δ 163.5, 148.0, 138.0, 137.0, 135.4, 134.7, 133.0, 131.9, 129.9, 129.1, 128.8, 128.1, 127.4, 126.7, 125.5, 122.9, 122.4, 122.2, 120.5, 76.3, 32.3, 30.2, 26.0, 23.4, 18.4, -1.9, -1.9.

HRMS (ESI-TOF) calcd for $C_{29}H_{35}NNaO_2Si^+$ ($[M+Na]^+$): 480.2329, found: 480.2325.

$[\alpha]_D^{20}$ = 29.7 (c = 0.72, $CHCl_3$).

The ee value was determined by HPLC analysis on a IG column (n-hexane/i-PrOH = 80/20, flow = 1.1 mL/min, 254 nm), t = 5.5 min (major), t = 4.5 min (minor). The absolute stereochemistry was assigned by analogy to compound **55**.

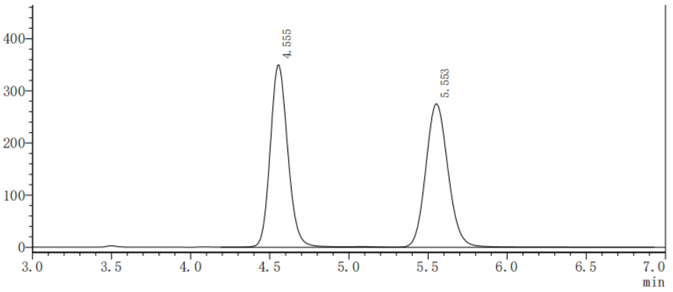

Peak	Ret. Time	Area	Height	Area%
1	4.555	2721074	349897	50.077
2	5.553	2712657	275249	49.923
Total		5433731	625146	100.000

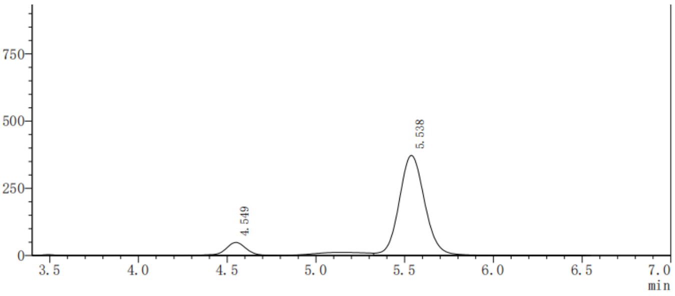

Peak	Ret. Time	Area	Height	Area%
1	4.549	392322	48916	9.495
2	5.538	3739404	372394	90.505
Total		4131726	421310	100.000

Supplementary Fig. 11 | Enantiomeric excess determination of product 10

(***S***)-5-(2-(2-((tert-butyldimethylsilyl)oxy)propan-2-yl)phenyl)-4-phenylphenanthridin-6(5H)-one(**11**)

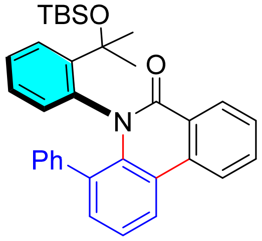

The title compound **11** was prepared according to **General Procedure C** and purified by preparative TLC in hexane/EtOAc = 10/1 as the eluent to afford a yellow oil (34 mg, 65% yield, 83% ee).
^{1}H NMR (400 MHz, Chloroform-*d*) δ 8.52 (dt, *J* = 8.1, 1.8 Hz, 1H), 8.42 – 8.32 (m, 2H), 7.86 – 7.79 (m, 1H), 7.69 (dt, *J* = 8.3, 1.9 Hz, 1H), 7.61 (td, *J* = 7.6, 2.1 Hz, 1H), 7.31 – 7.20 (m, 1H), 7.10 – 6.95 (m, 4H), 6.89 – 6.77 (m, 2H), 6.65 (dt, *J* = 7.9, 1.8 Hz, 1H), 6.58 (ddd, *J* = 9.7, 6.0, 4.2 Hz, 2H), 1.05 (dd, *J* = 5.9, 2.2 Hz, 6H), 0.88 (d, *J* = 2.3 Hz, 9H), -0.02 (d, *J* = 2.3 Hz, 3H), -0.06 (d, *J* = 2.3 Hz, 3H).

^{13}C NMR (101 MHz, Chloroform-*d*) δ 163.4, 145.4, 141.9, 137.8, 135.1, 134.8, 134.2, 133.1, 132.8, 130.3, 129.3, 129.3, 128.4, 128.2, 128.1, 128.0, 126.5, 126.1, 125.9, 122.7, 122.3, 122.2, 121.0, 75.6, 31.4, 30.8, 26.1, 18.4, -1.7, -2.2.

HRMS (ESI-TOF) calcd for $C_{34}H_{37}NNaO_2Si^+$ ($[M+Na]^+$): 542.2486, found: 542.2488.

$[\alpha]_D^{20}$ = 35.8 (c = 0.85, $CHCl_3$).

The ee value was determined by HPLC analysis on a IG column (n-hexane/i-PrOH = 90/10, flow = 0.8 mL/min, 254 nm), t = 8.8 min (major), t = 7.9 min (minor). The absolute stereochemistry was assigned by analogy to compound **55**.

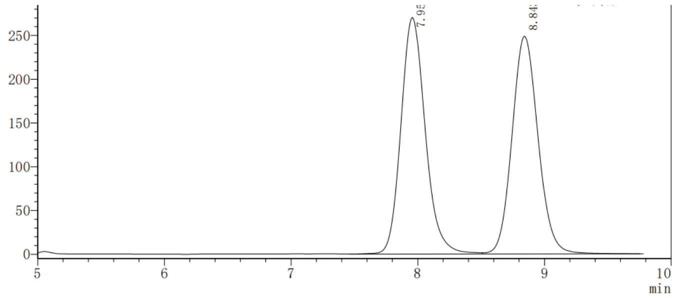

Peak	Ret. Time	Area	Height	Area%
1	7.958	3601187	270180	50.372
2	8.843	3548031	248731	49.628
Total		7149219	518911	100.000

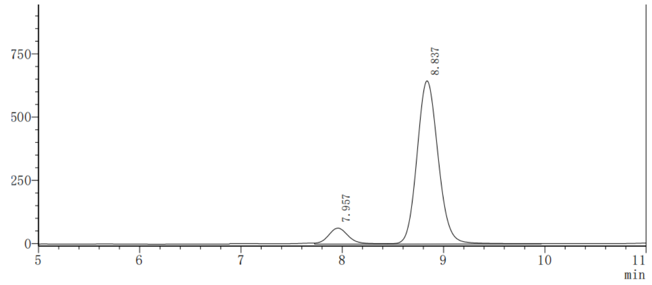

Peak	Ret. Time	Area	Height	Area%
1	7.957	886001	63234	8.600
2	8.837	9416769	644457	91.400
Total		10302769	707692	100.000

Supplementary Fig. 12 | Enantiomeric excess determination of product 11

(***S***)-12-(2-(tert-butyl)phenyl)benzofuro[2,3-*c*]phenanthridin-13(12H)-one(**12**)

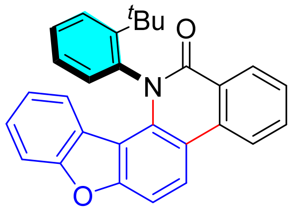

The title compound **12** was prepared according to **General Procedure C** and purified by preparative TLC in hexane/EtOAc = 10/1 as the eluent to afford a white foam (36 mg, 86% yield, 91% ee).

^{1}H NMR (400 MHz, Chloroform-*d*) δ 8.56 (d, *J* = 8.9 Hz, 1H), 8.52 (dd, *J* = 7.9, 1.5 Hz, 1H), 8.38 (d, *J* = 8.3 Hz, 1H), 7.91 – 7.75 (m, 2H), 7.68 – 7.55 (m, 3H), 7.47 (dd, *J* = 8.1, 1.2 Hz, 1H), 7.33 (td, *J* = 7.6, 1.5 Hz, 1H), 7.26 (td, *J* = 7.8, 7.1, 1.2 Hz, 1H), 7.20 (dd, *J* = 7.8, 1.5 Hz, 1H), 6.73 (ddd, *J* = 8.5, 7.1, 1.3 Hz, 1H), 4.95 (d, *J* = 8.5 Hz, 1H), 1.21 (s, 9H).
^{13}C NMR (101 MHz, Chloroform-*d*) δ 164.1, 158.4, 155.6, 150.1, 139.6, 136.9, 135.4, 133.3, 132.9, 130.6, 129.8, 129.1, 128.1, 127.6, 126.6, 125.8, 124.4, 124.1, 123.3, 122.5, 122.2, 114.8, 113.0, 110.9, 107.8, 36.7, 31.8.
HRMS (ESI-TOF) calcd for $C_{29}H_{23}NNaO_2^+$ ($[M+Na]^+$): 440.1621, found: 440.1623.
$[\alpha]_D^{20}$ = 184.3 (c = 0.91, $CHCl_3$).
The ee value was determined by HPLC analysis on a IG column (n-hexane/i-PrOH = 80/20, flow = 1.1 mL/min, 254 nm), t = 9.8 min (major), t = 10.5 min (minor). The absolute stereochemistry was assigned by analogy to compound **55**.

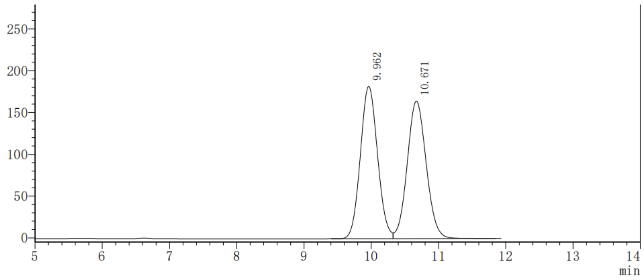

Peak	Ret. Time	Area	Height	Area%
1	9.962	3239458	182073	50.079
2	10.671	3229287	164491	49.921
Total		6468745	346564	100.000

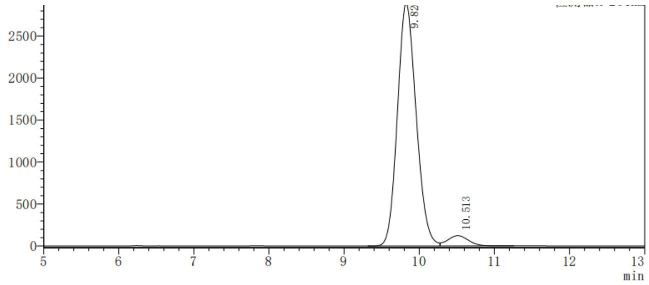

Peak	Ret. Time	Area	Height	Area%
1	9.826	50033830	2894563	95.518
2	10.513	2347589	122477	4.482
Total		52381419	3017040	100.000

Supplementary Fig. 13 | Enantiomeric excess determination of product 12

(***S***)-5-(2-(tert-butyl)phenyl)benzo[*c*][1,7]phenanthrolin-6(5H)-one(**13**)

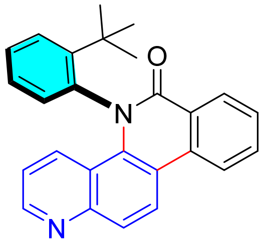

The title compound **13** was prepared according to **General Procedure D** and purified by preparative TLC in hexane/EtOAc = 4/1 as the eluent to afford a yellow solid (28 mg, 74% yield, 85% ee). mp:150.5-151.9 °C.

^{1}H NMR (600 MHz, Chloroform-*d*) δ 8.76 (d, *J* = 3.9 Hz, 1H), 8.68 (d, *J* = 9.0 Hz, 1H), 8.59 (d, *J* = 7.9 Hz, 1H), 8.45 (d, *J* = 8.3 Hz, 1H), 8.10 (d, *J* = 9.0 Hz, 1H), 7.89 (t, *J* = 7.5 Hz, 1H), 7.78 (d, *J* = 7.9 Hz, 1H), 7.68 (t, *J* = 7.5 Hz, 1H), 7.55 (t, *J* = 7.7 Hz, 1H), 7.41 (d, *J* = 9.1 Hz, 1H), 7.35 (t, *J* = 7.6 Hz, 1H), 7.05 (d, *J* = 8.1 Hz, 1H), 6.94 (dd, *J* = 9.3, 3.8 Hz, 1H), 1.16 (s, 9H).

^{13}C NMR (151 MHz, Chloroform-*d*) δ 164.1, 149.4, 147.5, 139.6, 135.1, 134.6, 134.3, 133.5, 131.2, 130.9, 129.6, 129.2, 128.8, 128.3, 125.7, 125.5, 124.9, 122.9, 122.7, 120.3, 119.7, 116.9, 36.7, 31.7.

HRMS (ESI-TOF) calcd for $C_{26}H_{22}N_2NaO^+$ ($[M+Na]^+$): 401.1624, found: 401.1626.

$[\alpha]_D^{20}$ = 62.4 (c = 0.84, $CHCl_3$).

The ee value was determined by HPLC analysis on a AD-H column (n-hexane/i-PrOH = 80/20, flow = 1.1 mL/min, 254 nm), t = 10.7 min (major), t = 12.8 min (minor). The absolute stereochemistry was assigned by analogy to compound **55**.

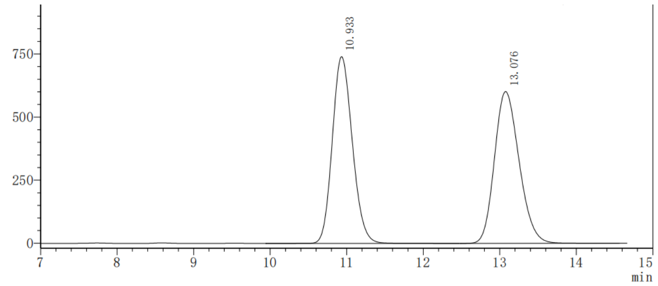

Peak	Ret. Time	Area	Height	Area%
1	10.933	13483171	740683	49.982
2	13.076	13492696	601832	50.018
Total		26975867	1342515	100.000

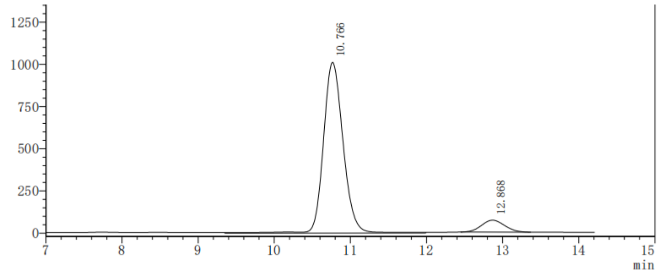

Peak	Ret. Time	Area	Height	Area%
1	10.766	18343700	1011677	92.472
2	12.868	1493420	70573	7.528
Total		19837120	1082250	100.000

Supplementary Fig. 14 | Enantiomeric excess determination of product 13

(***S***)-5-(2-(2-((tert-butyldimethylsilyl)oxy)propan-2-yl)phenyl)benzo[*c*][1,7]phenanthrolin-6(5H)-one(**14**)

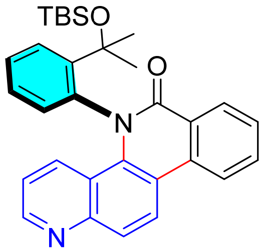

The title compound **14** was prepared according to **General Procedure C** and purified by preparative TLC in hexane/EtOAc = 10/1 as the eluent to afford a yellow foam (34 mg, 68% yield, 90% ee).
^{1}H NMR (400 MHz, Chloroform-*d*) δ 8.75 (dd, *J* = 4.1, 1.4 Hz, 1H), 8.65 (d, *J* = 9.1 Hz, 1H), 8.58 (dd, *J* = 8.0, 1.4 Hz, 1H), 8.45 (d, *J* = 8.3 Hz, 1H), 8.12 – 8.03 (m, 2H), 7.90 (ddd, *J* = 8.4, 7.1, 1.5 Hz, 1H), 7.71 – 7.62 (m, 1H), 7.57 (ddd, *J* = 8.4, 7.3, 1.4 Hz, 1H), 7.49 (dt, *J* = 9.2, 1.2 Hz, 1H), 7.34 (td, *J* = 7.6, 1.6 Hz, 1H), 6.96 (dd, *J* = 7.8, 1.4 Hz, 1H), 6.91 (dd, *J* = 9.2, 4.1 Hz, 1H), 1.54 (s, 3H), 1.22 (s, 3H), 0.84 (s, 9H), 0.06 (s, 3H), -0.08 (s, 3H).
^{13}C NMR (101 MHz, Chloroform-*d*) δ 163.7, 149.9, 149.8, 147.6, 137.8, 134.9, 134.3, 134.1, 133.5, 131.1, 130.2, 129.3, 129.2, 129.0, 128.7, 126.1, 125.6, 124.3, 122.8, 120.2, 119.9, 116.9, 32.2, 30.2, 26.0, 18.3, -1.9, -2.0.
HRMS (ESI-TOF) calcd for $C_{31}H_{34}N_2NaO_2Si^+$ ([M+Na]$^+$): 517.2282, found: 517.2281.
$[\alpha]_D^{20}$ = 38.8 (c = 0.89, $CHCl_3$).
The ee value was determined by HPLC analysis on a AD-H column (n-hexane/i-PrOH = 75/25, flow = 1.1 mL/min, 254 nm), t = 4.9 min (major), t = 5.4 min (minor). The absolute stereochemistry was assigned by analogy to compound **55**.

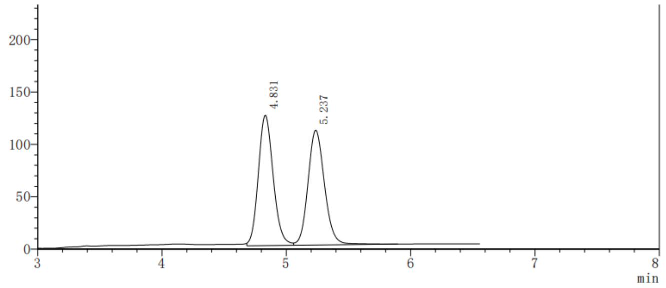

Peak	Ret. Time	Area	Height	Area%
1	4.831	1038212	124502	50.491
2	5.237	1018010	109597	49.509
Total		2056222	234099	100.000

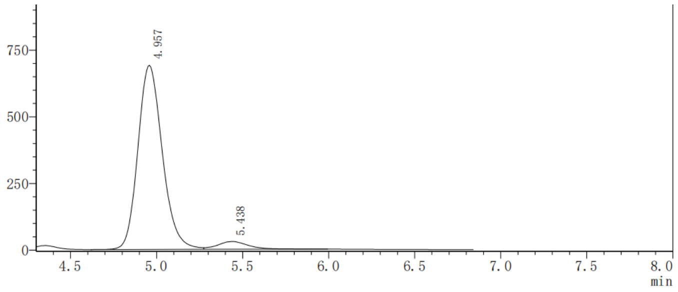

Peak	Ret. Time	Area	Height	Area%
1	4.957	6817176	690958	94.984
2	5.438	359988	29164	5.016

Total		7177164	720122	100.000

Supplementary Fig. 15 | Enantiomeric excess determination of product 14

(***S***)-5-(2-(tert-butyl)phenyl)-12-(3-(trifluoromethyl)phenyl)benzo[*c*]phenanthridin-6(5H)-one(**15**)

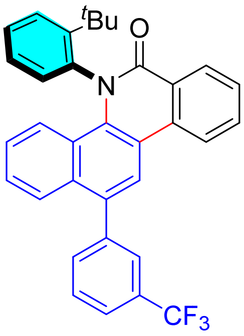

The title compound **15** was prepared according to **General Procedure C** and purified by preparative TLC in hexane/EtOAc = 10/1 as the eluent to afford a colorless oil (49 mg, 94% yield, 93% ee).

^{1}H NMR (600 MHz, Chloroform-*d*) δ 8.61 (dd, *J* = 8.0, 1.6 Hz, 1H), 8.42 (d, *J* = 8.3 Hz, 1H), 8.32 (s, 1H), 7.88 – 7.83 (m, 2H), 7.79 (dd, *J* = 8.3, 1.6 Hz, 1H), 7.77 – 7.72 (m, 3H), 7.66 (dt, *J* = 12.3, 7.6 Hz, 2H), 7.56 – 7.47 (m, 2H), 7.36 – 7.32 (m, 1H), 7.30 (td, *J* = 7.5, 1.5 Hz, 1H), 7.06 (ddd, *J* = 8.7, 6.7, 1.6 Hz, 1H), 6.99 (dd, *J* = 7.8, 1.6 Hz, 1H), 1.29 (s, 9H).

^{13}C NMR (151 MHz, Chloroform-*d*) δ 164.3, 147.6, 141.6, 140.5, 135.4, 134.9, 134.5, 133.7, 133.7, 133.3, δ 131.2 (q, J_{CF} = 31.7 Hz), 130.8, 130.7, 129.3, 129.1, 129.1, 128.4, 127.9, 127.1 (q, J_{CF} = 3.3 Hz), 126.6, 126.6, 126.5, 125.8, 125.2, 124.9, 124.5 (q, J_{CF} = 3.7 Hz), 124.3 (q, J_{CF} = 269.8 Hz), 122.7, 122.3, 116.1, 36.9, 31.8.

^{19}F NMR (565 MHz, Chloroform-*d*) δ -62.4.

HRMS (ESI-TOF) calcd for $C_{34}H_{26}F_3NNaO^+$ ($[M+Na]^+$): 544.1859, found: 544.1861.

$[\alpha]_D^{20}$ = 9.5 (c = 0.86, $CHCl_3$).

The ee value was determined by HPLC analysis on a OD-H column (n-hexane/i-PrOH = 90/10, flow = 0.8 mL/min, 254 nm), t = 12.8 min (major), t = 16.4 min (minor). The absolute stereochemistry was assigned by analogy to compound **55**.

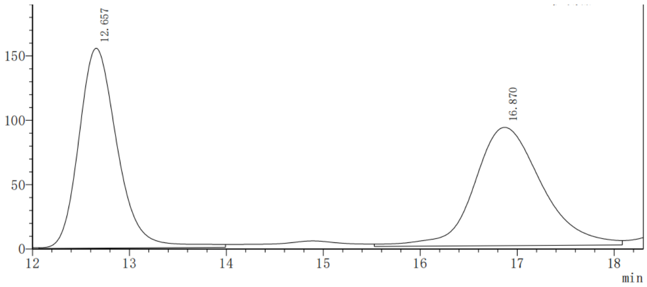

Peak	Ret. Time	Area	Height	Area%
1	12.657	4299655	155321	49.067
2	16.870	4463192	91888	50.933
Total		8762847	247209	100.000

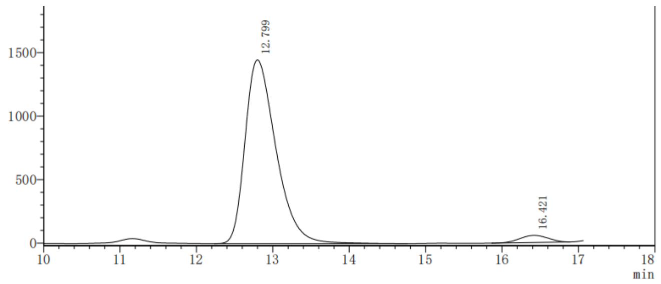

Peak	Ret. Time	Area	Height	Area%
1	12.799	40653894	1445828	96.599
2	16.421	1431471	55826	3.401
Total		42085365	1501654	100.000

Supplementary Fig. 16 | Enantiomeric excess determination of product 15

(***S***)-12-(3-acetylphenyl)-5-(2-(tert-butyl)phenyl)benzo[*c*]phenanthridin-6(5H)-one(**16**)

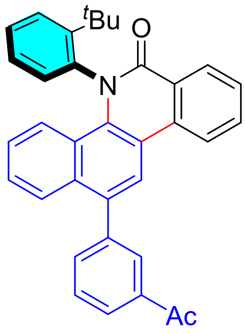

The title compound **16** was prepared according to **General Procedure C** and purified by preparative TLC in hexane/EtOAc = 5/1 as the eluent to afford a yellow oil (40 mg, 81% yield, 90% ee).
^{1}H NMR (600 MHz, Chloroform-*d*) δ 8.60 (dd, *J* = 8.0, 1.6 Hz, 1H), 8.41 (d, *J* = 8.3 Hz, 1H), 8.33 (s, 1H), 8.15 (s, 1H), 8.09 (dt, *J* = 8.0, 1.6 Hz, 1H), 7.84 (ddd, *J* = 8.4, 6.9, 1.6 Hz, 1H), 7.80 – 7.74 (m, 3H), 7.65 (td, *J* = 7.6, 4.3 Hz, 2H), 7.54 – 7.47 (m, 2H), 7.34 – 7.27 (m, 2H), 7.05 (ddd, *J* = 8.8, 6.8, 1.6 Hz, 1H), 6.99 (dd, *J* = 7.9, 1.6 Hz, 1H), 2.68 (s, 3H), 1.29 (s, 9H).
^{13}C NMR (151 MHz, Chloroform-*d*) δ 198.2, 164.3, 147.6, 141.4, 140.6, 137.6, 135.4, 135.3, 135.0, 134.6, 133.8, 133.2, 130.8, 130.7, 130.1, 129.3, 129.0, 128.9, 128.4, 127.9, 127.6, 126.7, 126.5, 125.8, 125.1, 124.9, 122.7, 122.2, 116.1, 36.9, 31.8, 27.0.
HRMS (ESI-TOF) calcd for $C_{35}H_{29}NNaO_2^+$ ([M+Na]$^+$): 518.2091, found: 518.2092.
$[\alpha]_D^{20}$ = 5.7 (c = 0.88, $CHCl_3$).
The ee value was determined by HPLC analysis on a IG column (n-hexane/i-PrOH = 80/20, flow = 1.05 mL/min, 254 nm), t = 20.6 min (major), t = 13.9 min (minor). The absolute stereochemistry was assigned by analogy to compound **55**.

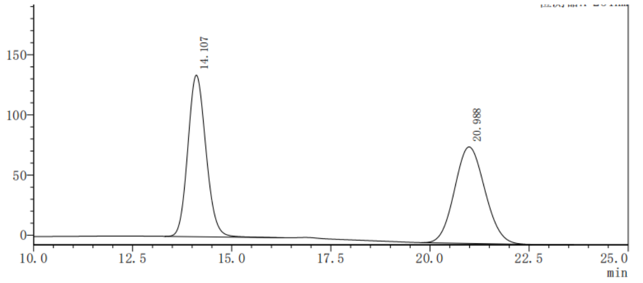

Peak	Ret. Time	Area	Height	Area%
1	14.107	4287695	134241	50.033
2	20.988	4282026	80129	49.967
Total		8569721	214371	100.000

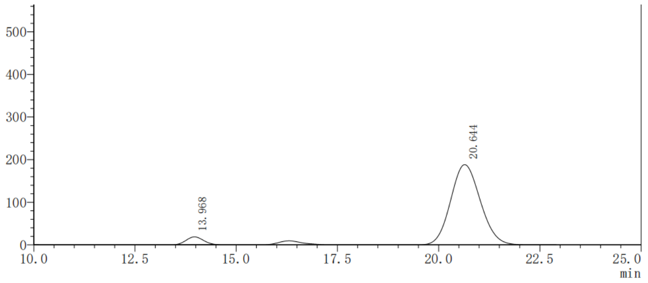

Peak	Ret. Time	Area	Height	Area%
1	13.968	543537	18502	5.279
2	20.644	9752956	188992	94.721
Total		10296493	207493	100.000

Supplementary Fig. 17 | Enantiomeric excess determination of product 16

(***S***)-5-(2-(tert-butyl)phenyl)-12-(3-fluorophenyl)benzo[*c*]phenanthridin-6(5H)-one(**17**)

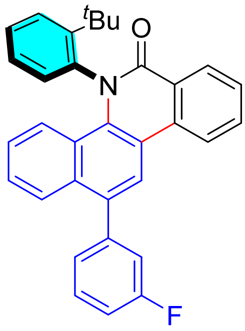

The title compound **17** was prepared according to **General Procedure C** and purified by preparative TLC in hexane/EtOAc = 10/1 as the eluent to afford a yellow foam (43 mg, 92% yield, 91% ee).
^{1}H NMR (600 MHz, Chloroform-*d*) δ 8.60 (dd, *J* = 8.0, 1.6 Hz, 1H), 8.41 (d, *J* = 8.3 Hz, 1H), 8.32 (s, 1H), 7.84 (ddd, *J* = 8.6, 6.8, 1.7 Hz, 2H), 7.79 (dd, *J* = 8.4, 1.7 Hz, 1H), 7.65 (t, *J* = 7.5 Hz, 1H), 7.55 – 7.47 (m, 3H), 7.36 – 7.32 (m, 2H), 7.29 (tt, *J* = 7.5, 1.6 Hz, 2H), 7.19 (td, *J* = 8.6, 2.4 Hz, 1H), 7.04 (ddd, *J* = 8.8, 6.7, 1.7 Hz, 1H), 6.99 (dd, *J* = 7.9, 1.7 Hz, 1H), 1.29 (s, 9H).
^{13}C NMR (151 MHz, Chloroform-*d*) δ 164.3, 162.9 (d, J_{CF} = 247.0 Hz), 147.6, 143.0 (d, J_{CF} = 7.6 Hz), 140.6, 135.3, 135.2, 134.6, 133.7, 133.2, 130.8, 130.6, 130.1, 130.1, 129.3, 129.0, 128.3, 127.9,

126.8, 126.4, 126.2 (d, J_{CF} = 3.1 Hz), 125.8, 125.1, 124.9, 122.7, 122.0, 117.4 (d, J_{CF} = 21.7 Hz), 116.0, 114.6 (d, J_{CF} = 20.8 Hz), 36.9, 31.8.

^{19}F NMR (565 MHz, Chloroform-*d*) δ -113.1.

HRMS (ESI-TOF) calcd for $C_{33}H_{26}NFNaO^+$ ($[M+Na]^+$): 494.1891, found: 494.1892.

$[\alpha]_D^{20}$ = 10.3 (c = 0.69, $CHCl_3$).

The ee value was determined by HPLC analysis on a IG column (n-hexane/i-PrOH = 80/20, flow = 1.1 mL/min, 254 nm), t = 8.1 min (major), t = 6.5 min (minor). The absolute stereochemistry was assigned by analogy to compound **55**.

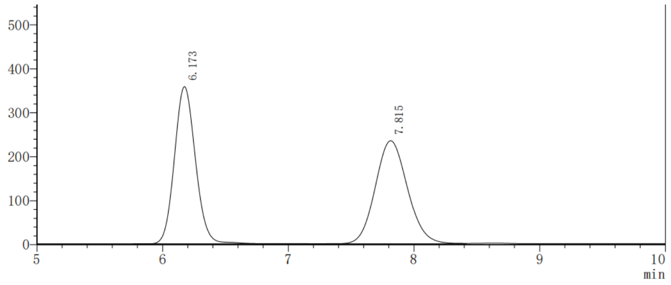

Peak	Ret. Time	Area	Height	Area%
1	6.173	4169416	357529	49.961
2	7.815	4175978	234370	50.039
Total		8345393	591899	100.000

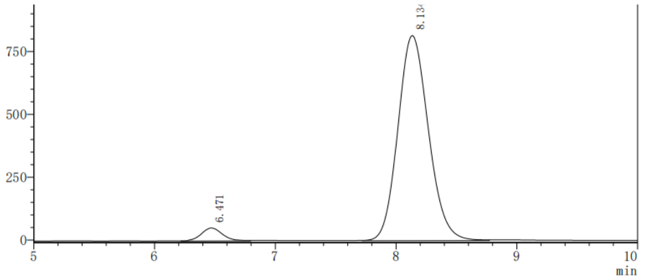

Peak	Ret. Time	Area	Height	Area%
1	6.471	639931	51435	4.278
2	8.134	14320283	815279	95.722
Total		14960214	866713	100.000

Supplementary Fig. 18 | Enantiomeric excess determination of product 17

(***S***)-5-(2-(tert-butyl)phenyl)-12-phenylbenzo[*c*]phenanthridin-6(5H)-one(**18**)

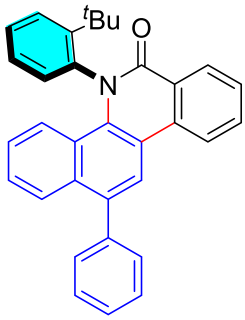

The title compound **18** was prepared according to **General Procedure C** and purified by preparative TLC in hexane/EtOAc = 10/1 as the eluent to afford a yellow oil (39 mg, 88% yield, 92% ee).

^{1}H NMR (600 MHz, Chloroform-*d*) δ 8.62 (d, *J* = 7.9 Hz, 1H), 8.42 (d, *J* = 8.3 Hz, 1H), 8.34 (s, 1H), 7.89 (d, *J* = 8.4 Hz, 1H), 7.83 (t, *J* = 7.7 Hz, 1H), 7.80 (d, *J* = 8.2 Hz, 1H), 7.64 (t, *J* = 7.5 Hz, 1H), 7.59 – 7.47 (m, 7H), 7.30 (q, *J* = 7.8 Hz, 2H), 7.04 (t, *J* = 8.0 Hz, 1H), 7.00 (d, *J* = 7.8 Hz, 1H), 1.31 (s, 9H).
^{13}C NMR (151 MHz, Chloroform-*d*) δ 164.3, 147.6, 140.8, 140.6, 136.6, 134.9, 134.7, 134.0, 133.1, 130.8, 130.6, 130.3, 129.2, 128.9, 128.6, 128.2, 127.8, 127.6, 127.1, 126.3, 126.2, 125.7, 124.9, 124.8, 122.7, 121.9, 116.1, 36.9, 31.8.
HRMS (ESI-TOF) calcd for $C_{33}H_{27}NNaO^+$ ($[M+Na]^+$): 476.1985, found: 476.1987.
$[\alpha]_D^{20}$ = 7.0 (c = 1.04, $CHCl_3$).
The ee value was determined by HPLC analysis on a AD-H column (n-hexane/i-PrOH = 80/20, flow = 1.0 mL/min, 254 nm), t = 5.4 min (major), t = 4.6 min (minor). The absolute stereochemistry was assigned by analogy to compound **55**.

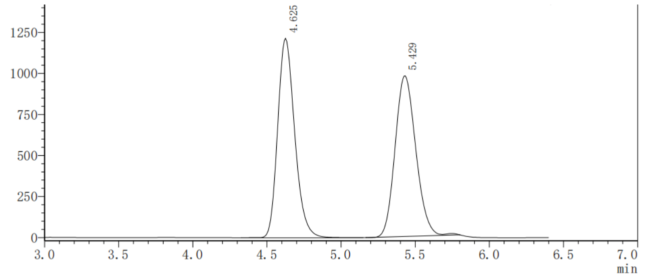

Peak	Ret. Time	Area	Height	Area%
1	4.625	9683440	1214655	50.459
2	5.429	9507241	977752	49.541
Total		19190681	2192407	100.000

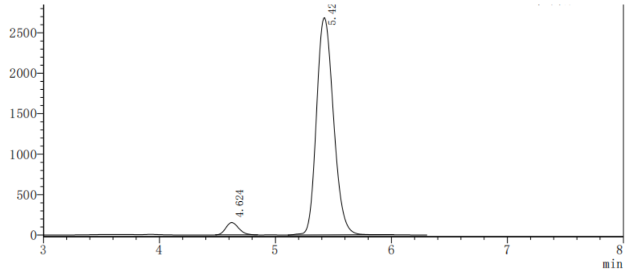

Peak	Ret. Time	Area	Height	Area%
1	4.624	1198269	153436	4.187
2	5.422	27421654	2683909	95.813
Total		28619923	2837345	100.000

Supplementary Fig. 19 | Enantiomeric excess determination of product 18

(***S***)-12-(4-acetylphenyl)-5-(2-(tert-butyl)phenyl)benzo[*c*]phenanthridin-6(5H)-one(**19**)

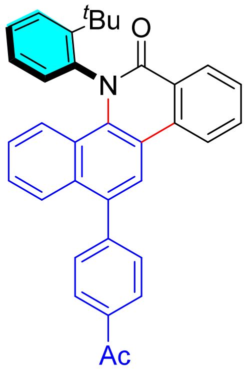

The title compound **19** was prepared according to **General Procedure C** and purified by preparative TLC in hexane/EtOAc = 4/1 as the eluent to afford a yellow foam (40 mg, 81% yield, 93% ee).

^{1}H NMR (600 MHz, Chloroform-*d*) δ 8.60 (d, *J* = 7.9 Hz, 1H), 8.40 (d, *J* = 8.3 Hz, 1H), 8.33 (s, 1H), 8.14 (d, *J* = 8.4 Hz, 2H), 7.86 – 7.75 (m, 3H), 7.68 (d, *J* = 7.8 Hz, 2H), 7.64 (t, *J* = 7.5 Hz, 1H), 7.52 (t, *J* = 8.7 Hz, 2H), 7.35 – 7.26 (m, 2H), 7.05 (ddd, *J* = 8.6, 6.8, 1.6 Hz, 1H), 6.99 (d, *J* = 7.8 Hz, 1H), 2.71 (s, 3H), 1.29 (s, 9H).

^{13}C NMR (151 MHz, Chloroform-*d*) δ 197.9, 164.3, 147.6, 145.8, 140.5, 136.3, 135.4, 135.3, 134.5, 133.5, 133.2, 130.7, 130.6, 129.2, 129.0, 128.7, 128.4, 127.8, 126.7, 126.5, 126.4, 125.7, 125.2, 124.9, 122.6, 122.0, 116.0, 36.9, 31.8, 26.9.

HRMS (ESI-TOF) calcd for $C_{35}H_{29}NNaO_2^+$ ([M+Na]$^+$): 518.2091, found: 518.2092.

$[\alpha]_D^{20}$ = 6.9 (c = 0.91, $CHCl_3$).

The ee value was determined by HPLC analysis on a AD-H column (n-hexane/i-PrOH = 80/20, flow = 1.0 mL/min, 254 nm), t = 8.6 min (major), t = 7.8 min (minor). The absolute stereochemistry was assigned by analogy to compound **55**.

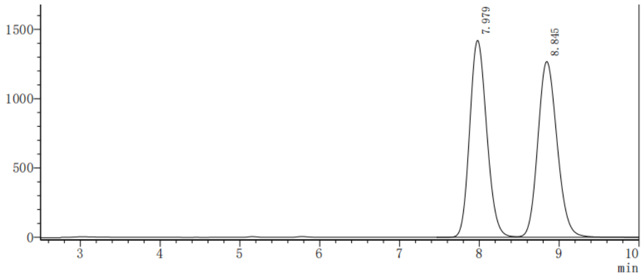

Peak	Ret. Time	Area	Height	Area%
1	7.979	21148961	1421049	49.790
2	8.845	21327703	1268288	50.210
Total		42476665	2689337	100.000

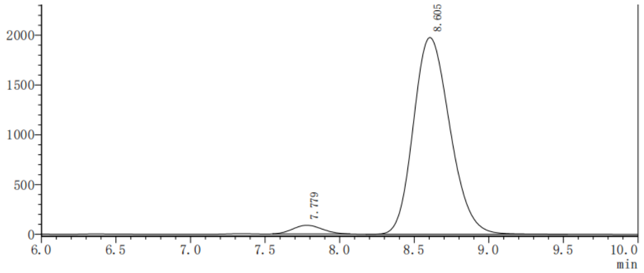

Peak	Ret. Time	Area	Height	Area%

1	7.779	1202373	87243	3.445
2	8.605	33701355	1975769	96.555
Total		34903728	2063012	100.000

Supplementary Fig. 20 | Enantiomeric excess determination of product 19

(***S***)-5-(2-(tert-butyl)phenyl)-12-(4-chlorophenyl)benzo[*c*]phenanthridin-6(5H)-one(**20**)

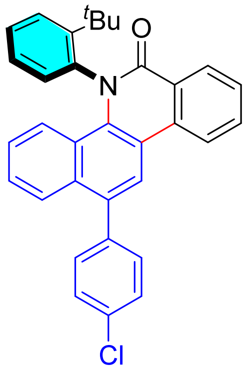

The title compound **20** was prepared according to **General Procedure C** and purified by preparative TLC in hexane/EtOAc = 5/1 as the eluent to afford a yellow foam (44 mg, 90% yield, 91% ee).
^{1}**H NMR (600 MHz, Chloroform-*d*)** δ 8.60 (dd, *J* = 8.0, 1.5 Hz, 1H), 8.40 (d, *J* = 8.3 Hz, 1H), 8.30 (s, 1H), 7.87 – 7.75 (m, 3H), 7.67 – 7.61 (m, 1H), 7.51 (qd, *J* = 7.8, 6.9, 1.7 Hz, 6H), 7.35 – 7.26 (m, 2H), 7.04 (ddd, *J* = 8.9, 6.8, 1.6 Hz, 1H), 6.98 (dd, *J* = 7.8, 1.5 Hz, 1H), 1.29 (s, 9H).
^{13}**C NMR (151 MHz, Chloroform-*d*)** δ 164.3, 147.6, 140.6, 139.3, 135.2, 135.2, 134.6, 133.8, 133.8, 133.2, 131.7, 130.8, 130.6, 129.3, 129.0, 128.8, 128.3, 127.8, 126.8, 126.4, 126.4, 125.8, 125.1, 124.9, 122.6, 122.0, 116.1, 36.9, 31.8.
HRMS (ESI-TOF) calcd for $C_{33}H_{26}NClNaO^{+}$ ($[M+Na]^{+}$): 510.1595, found: 510.1596.
$[\alpha]_D^{20}$ = 5.5 (c = 0.94, $CHCl_3$).
The ee value was determined by HPLC analysis on a IG column (n-hexane/i-PrOH = 75/25, flow = 1.1 mL/min, 254 nm), t = 7.2 min (major), t = 6.0 min (minor). The absolute stereochemistry was assigned by analogy to compound **55**.

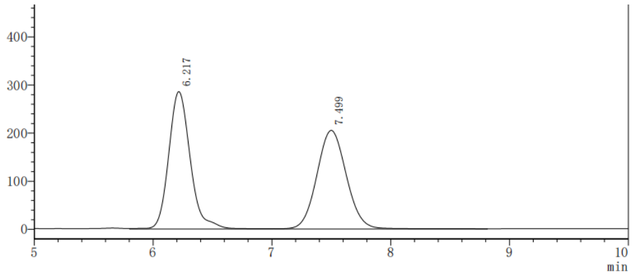

Peak	Ret. Time	Area	Height	Area%
1	6.217	3615932	285884	50.395
2	7.499	3559257	204958	49.605
Total		7175189	490842	100.000

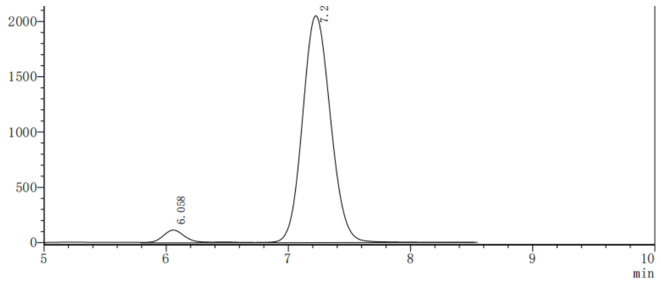

Peak	Ret. Time	Area	Height	Area%
1	6.058	1524417	115258	4.449
2	7.225	32737770	2052054	95.551
Total		34262186	2167312	100.000

Supplementary Fig. 21 | Enantiomeric excess determination of product 20

(***S***)-4-(5-(2-(tert-butyl)phenyl)-6-oxo-5,6-dihydrobenzo[*c*]phenanthridin-12-yl)benzaldehyde(**21**)

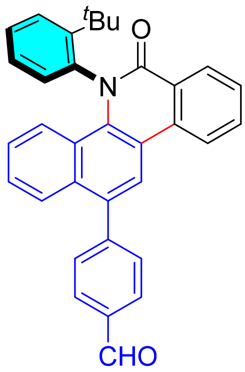

The title compound **21** was prepared according to **General Procedure C** and purified by preparative TLC in hexane/EtOAc = 5/1 as the eluent to afford a white foam (44.5 mg, 93% yield, 91% ee).
^{1}H NMR (600 MHz, Chloroform-*d*) δ 10.16 (s, 1H), 8.60 (dd, *J* = 8.0, 1.5 Hz, 1H), 8.41 (d, *J* = 8.2 Hz, 1H), 8.34 (s, 1H), 8.07 (d, *J* = 7.8 Hz, 2H), 7.84 (ddd, *J* = 8.4, 7.0, 1.5 Hz, 1H), 7.79 (ddd, *J* = 8.2, 6.7, 1.5 Hz, 2H), 7.75 (d, *J* = 7.8 Hz, 2H), 7.67 – 7.62 (m, 1H), 7.52 (t, *J* = 8.6 Hz, 2H), 7.33 (ddd, *J* = 8.1, 6.6, 1.1 Hz, 1H), 7.29 (td, *J* = 7.5, 1.5 Hz, 1H), 7.06 (ddd, *J* = 8.9, 6.8, 1.5 Hz, 1H), 6.99 (dd, *J* = 7.8, 1.5 Hz, 1H), 1.29 (s, 9H).
^{13}C NMR (151 MHz, Chloroform-*d*) δ 192.0, 164.3, 147.6, 147.3, 140.5, 135.7, 135.6, 135.1, 134.5, 133.4, 133.3, 131.1, 130.8, 130.7, 130.0, 129.3, 129.1, 128.4, 127.9, 126.6, 126.6, 126.5, 125.8, 125.2, 124.9, 122.6, 122.1, 116.1, 36.9, 31.8.
HRMS (ESI-TOF) calcd for $C_{34}H_{27}NNaO_2^+$ ([M+Na]$^+$): 504.1935, found: 504.1934.
$[\alpha]_D^{20}$ = 8.8 (c = 0.87, $CHCl_3$).
The ee value was determined by HPLC analysis on a AD-H column (n-hexane/i-PrOH = 75/25, flow = 1.1 mL/min, 254 nm), t = 7.0 min (major), t = 5.9 min (minor). The absolute stereochemistry was assigned by analogy to compound **55**.

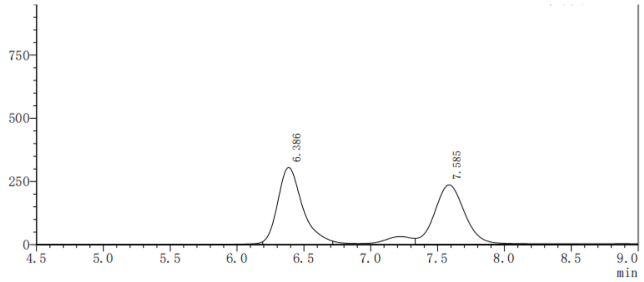

Peak	Ret. Time	Area	Height	Area%
1	6.386	4041792	307508	51.521
2	7.585	3803173	236606	48.479
Total		7844965	544115	100.000

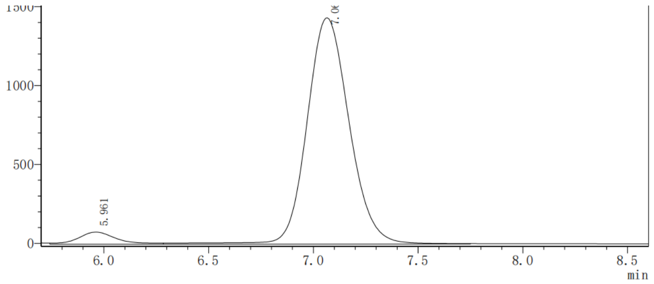

Peak	Ret. Time	Area	Height	Area%
1	5.961	988851	77453	4.666
2	7.065	20204685	1434656	95.334
Total		21193535	1512109	100.000

Supplementary Fig. 22 | Enantiomeric excess determination of product 21

(***S***)-4-(5-(2-(tert-butyl)phenyl)-6-oxo-5,6-dihydrobenzo[*c*]phenanthridin-12-yl)benzonitrile(**22**)

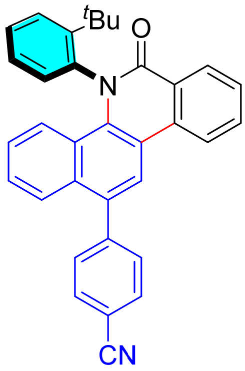

The title compound **22** was prepared according to **General Procedure C** and purified by preparative TLC in hexane/EtOAc = 4/1 as the eluent to afford a yellow oil (40 mg, 84% yield, 90% ee).
^{1}H NMR (600 MHz, Chloroform-*d*) δ 8.60 (dd, *J* = 8.0, 1.5 Hz, 1H), 8.39 (d, *J* = 8.4 Hz, 1H), 8.29 (s, 1H), 7.85 (dd, *J* = 8.3, 6.6 Hz, 3H), 7.79 (dd, *J* = 8.2, 1.5 Hz, 1H), 7.73 (dd, *J* = 8.4, 1.5 Hz, 1H), 7.69 (d, *J* = 7.9 Hz, 2H), 7.67 – 7.62 (m, 1H), 7.55 – 7.48 (m, 2H), 7.34 (ddd, *J* = 8.2, 6.7, 1.1 Hz, 1H), 7.29 (td, *J* = 7.6, 1.5 Hz, 1H), 7.10 – 7.03 (m, 1H), 6.98 (dd, *J* = 7.7, 1.5 Hz, 1H), 1.28 (s, 9H).

^{13}C NMR (151 MHz, Chloroform-*d*) δ 164.3, 147.6, 145.8, 140.4, 135.7, 134.5, 134.4, 133.3, 133.3, 132.4, 131.2, 130.8, 130.7, 129.3, 129.1, 128.5, 127.9, 126.7, 126.6, 126.3, 125.8, 125.3, 124.9, 122.6, 122.2, 118.9, 116.0, 111.6, 36.9, 31.8.

HRMS (ESI-TOF) calcd for $C_{34}H_{26}N_2NaO^+$ ($[M+Na]^+$): 501.1939, found: 501.1937.

$[\alpha]_D^{20}$ = 9.5 (c = 0.93, $CHCl_3$).

The ee value was determined by HPLC analysis on a IG column (n-hexane/i-PrOH = 75/25, flow = 1.1 mL/min, 254 nm), t = 16.4 min (major), t = 12.5 min (minor). The absolute stereochemistry was assigned by analogy to compound **55**.

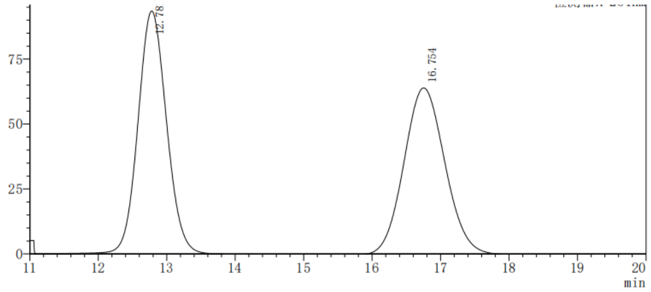

Peak	Ret. Time	Area	Height	Area%
1	12.781	2736663	93632	50.359
2	16.754	2697667	64310	49.641
Total		5434329	157942	100.000

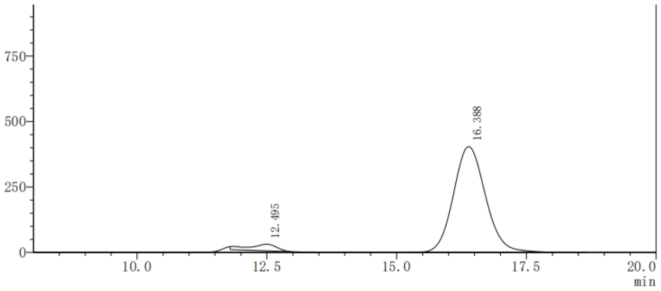

Peak	Ret. Time	Area	Height	Area%
1	12.495	950016	25504	5.173
2	16.388	17413983	404478	94.827
Total		18363999	429982	100.000

Supplementary Fig. 23 | Enantiomeric excess determination of product 22

(***S***)-5-(2-(tert-butyl)phenyl)-12-(4-nitrophenyl)benzo[*c*]phenanthridin-6(5H)-one(**23**)

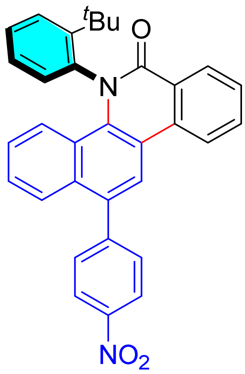

The title compound **23** was prepared according to **General Procedure C** and purified by preparative TLC in hexane/EtOAc = 4/1 as the eluent to afford a yellow solid (49 mg, 99% yield, 92% ee). mp: 118.6-120.2 °C.

^{1}H NMR (600 MHz, Chloroform-*d*) δ 8.60 (dd, *J* = 8.0, 1.5 Hz, 1H), 8.41 (t, *J* = 8.3 Hz, 3H), 8.33 (s, 1H), 7.85 (ddd, *J* = 8.3, 6.9, 1.6 Hz, 1H), 7.79 (dd, *J* = 8.2, 1.6 Hz, 1H), 7.77 – 7.72 (m, 3H), 7.66 (t, *J* = 7.5 Hz, 1H), 7.56 – 7.49 (m, 2H), 7.38 – 7.32 (m, 1H), 7.29 (td, *J* = 7.5, 1.5 Hz, 1H), 7.07 (ddd, *J* = 8.7, 6.7, 1.6 Hz, 1H), 6.98 (dd, *J* = 7.9, 1.6 Hz, 1H), 1.29 (s, 9H).

^{13}C NMR (151 MHz, Chloroform-*d*) δ 164.3, 147.7, 147.6, 147.5, 140.4, 135.8, 134.4, 134.0, 133.3, 133.2, 131.3, 130.8, 130.7, 129.3, 129.1, 128.5, 127.9, 126.8, 126.7, 126.3, 125.8, 125.4, 124.9, 123.9, 122.6, 122.3, 116.0, 36.9, 31.8.

HRMS (ESI-TOF) calcd for $C_{33}H_{26}N_2NaO_3^+$ ([M+Na]$^+$): 521.1836, found: 521.1837.

$[\alpha]_D^{20}$ = 15.3 (c = 0.75, $CHCl_3$).

The ee value was determined by HPLC analysis on a IG column (n-hexane/i-PrOH = 75/25, flow = 1.0 mL/min, 254 nm), t = 19.9 min (major), t = 14.1 min (minor). The absolute stereochemistry was assigned by analogy to compound **55**.

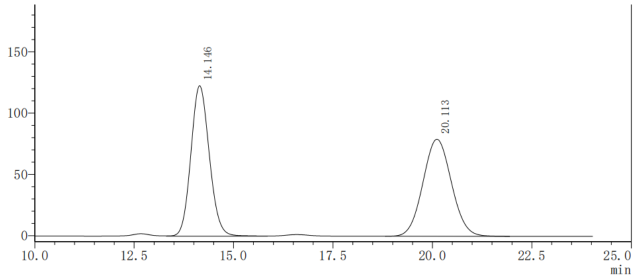

Peak	Ret. Time	Area	Height	Area%
1	14.146	3964794	122739	50.045
2	20.113	3957602	79188	49.955
Total		7922396	201927	100.000

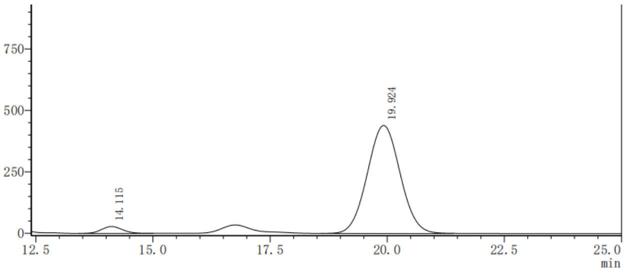

Peak	Ret. Time	Area	Height	Area%
1	14.115	886312	28015	3.952
2	19.924	21541004	439285	96.048
Total		22427316	467300	100.000

Supplementary Fig. 24 | Enantiomeric excess determination of product 23

(***S***)-5-(2-(tert-butyl)phenyl)-12-(4-(trifluoromethyl)phenyl)benzo[*c*]phenanthridin-6(5H)-one(**24**)

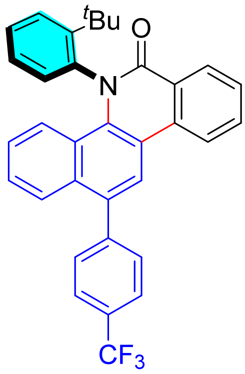

The title compound **24** was prepared according to **General Procedure C** and purified by preparative TLC in hexane/EtOAc = 10/1 as the eluent to afford a colorless oil (45 mg, 88% yield, 91% ee).

^{1}H NMR (600 MHz, Chloroform-*d*) δ 8.60 (dd, *J* = 7.9, 1.5 Hz, 1H), 8.40 (d, *J* = 8.3 Hz, 1H), 8.32 (s, 1H), 7.84 (ddd, *J* = 8.4, 7.0, 1.5 Hz, 1H), 7.82 – 7.76 (m, 4H), 7.69 (d, *J* = 7.9 Hz, 2H), 7.65 (t, *J* = 7.6 Hz, 1H), 7.55 – 7.49 (m, 2H), 7.35 – 7.31 (m, 1H), 7.29 (td, *J* = 7.5, 1.5 Hz, 1H), 7.05 (ddd, *J* = 8.8, 6.7, 1.5 Hz, 1H), 6.98 (dd, *J* = 7.8, 1.5 Hz, 1H), 1.29 (s, 9H).

^{13}C NMR (151 MHz, Chloroform-*d*) δ 164.3, 147.6, 144.6, 140.5, 135.4, 135.0, 134.5, 133.6, 133.3, 130.7, 130.7, 129.9 (q, J_{CF} = 32.4 Hz), 129.3, 129.1, 128.4, 127.9, 126.6, 126.5, 126.5, 125.8, 125.6 (q, J_{CF} = 3.5 Hz), 125.2, 124.9, 124.4 (q, J_{CF} = 272.1 Hz), 122.6, 122.1, 116.0, 36.9, 31.8.

^{19}F NMR (565 MHz, Chloroform-*d*) δ -62.4.

HRMS (ESI-TOF) calcd for $C_{34}H_{26}F_3NNaO^+$ ([M+Na]$^+$): 544.1859, found: 544.1862.

$[\alpha]_D^{20}$ = 9.4 (c = 0.96, $CHCl_3$).

The ee value was determined by HPLC analysis on a OD-H column (n-hexane/i-PrOH = 92/8, flow = 0.9 mL/min, 254 nm), t = 11.7 min (major), t = 13.4 min (minor). The absolute stereochemistry was assigned by analogy to compound **55**.

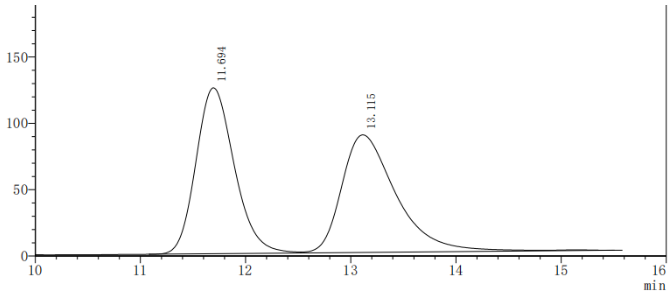

Peak	Ret. Time	Area	Height	Area%
1	11.694	3179450	125063	49.820
2	13.115	3202372	88626	50.180
Total		6381822	213689	100.000

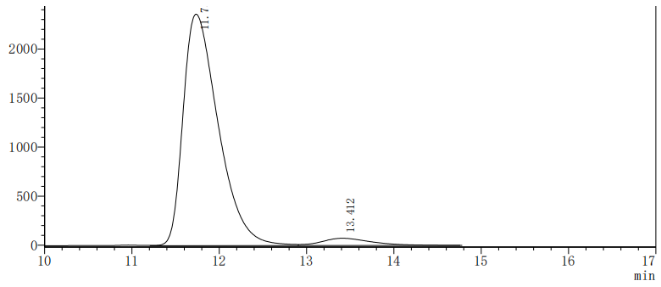

Peak	Ret. Time	Area	Height	Area%
1	11.736	66049934	2360380	95.762
2	13.412	2923164	74024	4.238
Total		68973098	2434404	100.000

Supplementary Fig. 25 | Enantiomeric excess determination of product 24

(***S***)-5-(2-(tert-butyl)phenyl)-12-(4-(trifluoromethoxy)phenyl)benzo[*c*]phenanthridin-6(5H)-one(**25**)

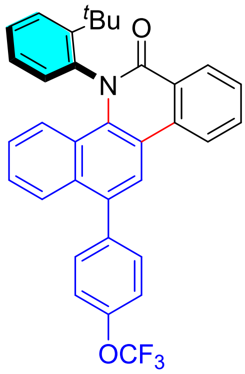

The title compound **25** was prepared according to **General Procedure C** and purified by preparative TLC in hexane/EtOAc = 10/1 as the eluent to afford a off white foam (45 mg, 84% yield, 92% ee).

^{1}H NMR (600 MHz, Chloroform-*d*) δ 8.60 (dd, *J* = 8.0, 1.5 Hz, 1H), 8.41 (d, *J* = 8.3 Hz, 1H), 8.31 (s, 1H), 7.84 (ddd, *J* = 8.4, 7.1, 1.5 Hz, 1H), 7.79 (td, *J* = 8.5, 1.6 Hz, 2H), 7.66 – 7.62 (m, 1H), 7.59 (d, *J* = 8.5 Hz, 2H), 7.52 (ddd, *J* = 8.3, 5.1, 1.9 Hz, 2H), 7.40 (d, *J* = 8.1 Hz, 2 H), 7.33 (ddd, *J* = 8.0, 6.7, 1.1 Hz, 1H), 7.29 (td, *J* = 7.5, 1.5 Hz, 1H), 7.08 – 7.01 (m, 1H), 6.98 (dd, *J* = 7.8, 1.6 Hz, 1H), 1.29 (s, 9H).

^{13}C NMR (151 MHz, Chloroform-*d*) δ 164.3, 148.9, 147.7, 140.6, 139.5, 135.3, 135.0, 134.6, 133.8, 133.2, 131.8, 130.8, 130.7, 129.3, 129.0, 128.4, 127.9, 126.8, 126.5, 126.4, 125.8, 125.1, 124.9, 122.7, 122.2, 121.1, 120.8 (q, J_{CF} = 258.2 Hz), 116.1, 36.9, 31.8.

^{19}F NMR (565 MHz, Chloroform-*d*) δ -57.7.

HRMS (ESI-TOF) calcd for $C_{34}H_{26}F_3NNaO_2^+$ ([M+Na]$^+$): 560.1808, found: 560.1807.

$[\alpha]_D^{20}$ = 9.1 (c = 0.89, $CHCl_3$).

The ee value was determined by HPLC analysis on a AD-H column (n-hexane/i-PrOH = 95/5, flow = 0.5 mL/min, 254 nm), t = 15.6 min (major), t = 14.2 min (minor). The absolute stereochemistry was assigned by analogy to compound **55**.

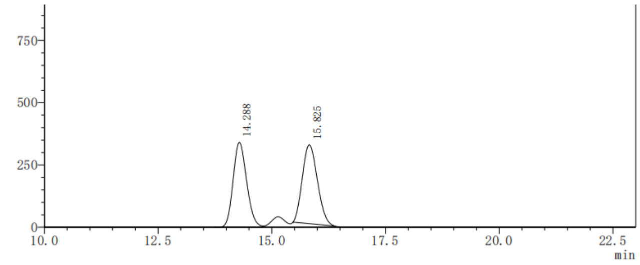

Peak	Ret. Time	Area	Height	Area%
1	14.288	7081217	340738	49.208
2	15.825	7309279	316960	50.792
Total		14390496	657698	100.000

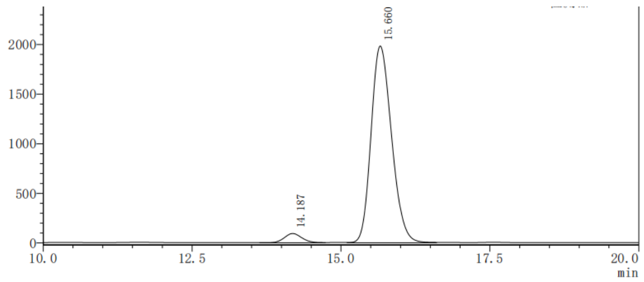

Peak	Ret. Time	Area	Height	Area%
1	14.187	1959543	93209	3.960
2	15.660	47523774	1982445	96.040
Total		49483318	2075654	100.000

Supplementary Fig. 26 | Enantiomeric excess determination of product 25

(***S***)-5-(2-(tert-butyl)phenyl)-12-(4-morpholinophenyl)benzo[*c*]phenanthridin-6(5H)-one(**26**)

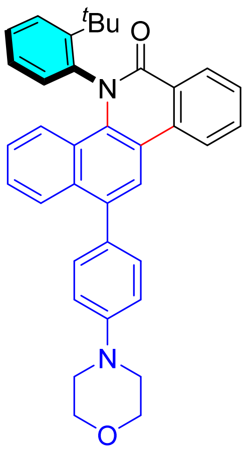

The title compound **26** was prepared according to **General Procedure C** and purified by preparative TLC in hexane/EtOAc = 4/1 as the eluent to afford a yellow solid (48 mg, 89% yield, 90% ee). mp:148.7-150 °C.

^{1}H NMR (600 MHz, Chloroform-*d*) δ 8.59 (dd, *J* = 8.0, 1.5 Hz, 1H), 8.41 (d, *J* = 8.3 Hz, 1H), 8.31 (s, 1H), 7.93 (dd, *J* = 8.4, 1.5 Hz, 1H), 7.82 (ddd, *J* = 8.4, 7.0, 1.5 Hz, 1H), 7.78 (dd, *J* = 8.2, 1.5 Hz, 1H), 7.63 (t, *J* = 7.4 Hz, 1H), 7.53 – 7.45 (m, 4H), 7.33 – 7.25 (m, 2H), 7.10 – 7.06 (m, 2H), 7.02

(ddd, J = 8.6, 6.7, 1.6 Hz, 1H), 6.98 (dd, J = 7.8, 1.6 Hz, 1H), 4.09 – 3.86 (m, 4H), 3.47 – 3.13 (m, 4H), 1.29 (s, 9H).

^{13}C NMR (151 MHz, Chloroform-*d*) δ 164.3, 150.8, 147.7, 140.7, 136.4, 134.8, 134.7, 134.3, 133.1, 132.2, 131.2, 130.8, 130.6, 129.2, 128.9, 128.2, 127.8, 127.2, 126.3, 126.1, 125.8, 124.9, 124.9, 122.7, 121.8, 116.2, 115.5, 67.1, 49.4, 36.9, 31.8.

HRMS (ESI-TOF) calcd for $C_{37}H_{34}N_2NaO_2^+$ ($[M+Na]^+$): 561.2512, found: 561.2511.

$[\alpha]_D^{20}$ = 6.1 (c = 0.98, $CHCl_3$).

The ee value was determined by HPLC analysis on a AD-H column (n-hexane/i-PrOH = 75/25, flow = 1.1 mL/min, 254 nm), t = 6.2 min (major), t = 6.8 min (minor). The absolute stereochemistry was assigned by analogy to compound **55**.

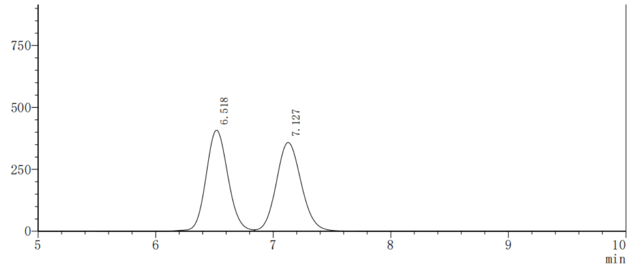

Peak	Ret. Time	Area	Height	Area%
1	6.518	5538439	413601	50.115
2	7.127	5513003	363659	49.885
Total		11051442	777261	100.000

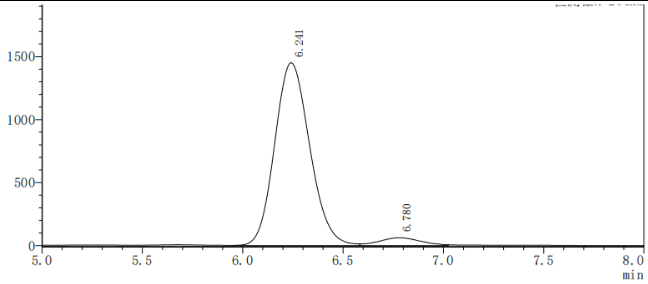

Peak	Ret. Time	Area	Height	Area%
1	6.241	17639623	1453152	95.087
2	6.780	911342	62578	4.913
Total		18550965	1515729	100.000

Supplementary Fig. 27 | Enantiomeric excess determination of product 26

(***S***)-12-(4-(9H-carbazol-9-yl)phenyl)-5-(2-(tert-butyl)phenyl)benzo[*c*]phenanthridin-6(5H)-one(**27**)

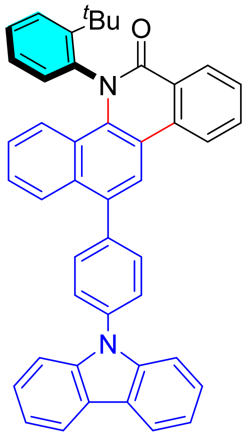

The title compound **27** was prepared according to **General Procedure C** and purified by preparative TLC in hexane/EtOAc = 5/1 as the eluent to afford a yellow foam (58 mg, 94% yield, 90% ee).

^{1}H NMR (400 MHz, Chloroform-*d*) δ 8.64 (d, *J* = 7.3 Hz, 1H), 8.50 (d, *J* = 8.3 Hz, 1H), 8.47 (s, 1H), 8.21 (d, *J* = 7.7 Hz, 2H), 8.04 (d, *J* = 8.3 Hz, 1H), 7.87 (t, *J* = 7.3 Hz, 1H), 7.84 – 7.75 (m, 5H), 7.67 (t, *J* = 7.5 Hz, 1H), 7.64 – 7.54 (m, 4H), 7.49 (t, *J* = 7.7 Hz, 2H), 7.42 (t, *J* = 7.6 Hz, 1H), 7.38 – 7.29 (m, 3H), 7.10 (ddd, *J* = 8.7, 6.7, 1.4 Hz, 1H), 7.03 (dd, *J* = 7.8, 1.4 Hz, 1H), 1.33 (s, 9H).

^{13}C NMR (101 MHz, Chloroform-*d*) δ 164.3, 147.6, 141.0, 140.6, 139.9, 137.2, 135.6, 135.2, 134.7, 133.9, 133.2, 131.8, 130.8, 130.7, 129.3, 129.0, 128.4, 127.9, 127.2, 127.1, 126.9, 126.5, 126.1, 125.8, 125.1, 125.0, 123.7, 122.7, 122.2, 120.6, 120.3, 116.2, 110.0, 36.9, 31.8.

HRMS (ESI-TOF) calcd for $C_{45}H_{34}N_2NaO^+$ ($[M+Na]^+$): 641.2563, found: 641.2566.

$[\alpha]_D^{20}$ = 15.0 (c = 1.1, $CHCl_3$).

The ee value was determined by HPLC analysis on a IG column (n-hexane/i-PrOH = 80/20, flow = 1.1 mL/min, 254 nm), t = 24.0 min (major), t = 26.9 min (minor). The absolute stereochemistry was assigned by analogy to compound **55**.

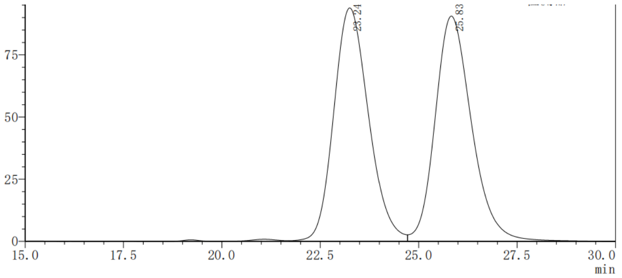

Peak	Ret. Time	Area	Height	Area%
1	23.249	5780123	93819	49.740
2	25.831	5840529	90549	50.260
Total		11620652	184369	100.000

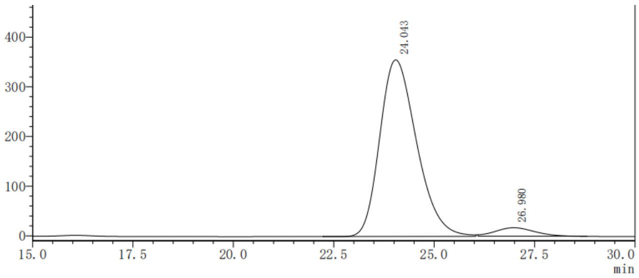

Peak	Ret. Time	Area	Height	Area%
1	24.043	22677041	355634	95.219
2	26.980	1138751	16953	4.781
Total		23815792	372586	100.000

Supplementary Fig. 28 | Enantiomeric excess determination of product 27

(***S***)-5-(2-(tert-butyl)phenyl)-12-(4-(diphenylamino)phenyl)benzo[*c*]phenanthridin-6(5H)-one(**28**)

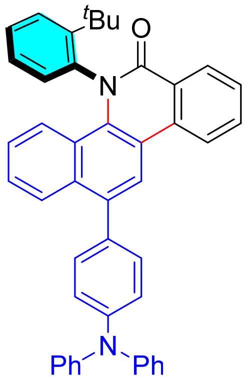

The title compound **28** was prepared according to **General Procedure C** and purified by preparative TLC in hexane/EtOAc = 4/1 as the eluent to afford a yellow foam (47 mg, 76% yield, 75% ee).
^{1}H NMR (400 MHz, Chloroform-*d*) δ 8.60 (dd, *J* = 8.0, 1.4 Hz, 1H), 8.45 (d, *J* = 8.3 Hz, 1H), 8.35 (s, 1H), 8.01 (dd, *J* = 8.5, 1.5 Hz, 1H), 7.84 (ddd, *J* = 8.4, 7.1, 1.5 Hz, 1H), 7.78 (dd, *J* = 8.2, 1.5 Hz, 1H), 7.67 – 7.61 (m, 1H), 7.55 – 7.47 (m, 2H), 7.45 – 7.40 (m, 2H), 7.37 – 7.28 (m, 6H), 7.26 – 7.21 (m, 6H), 7.11 – 7.01 (m, 3H), 6.98 (dd, *J* = 7.9, 1.5 Hz, 1H), 1.30 (s, 9H).
^{13}C NMR (101 MHz, Chloroform-*d*) δ 164.3, 147.8, 147.6, 147.4, 140.6, 138.4, 136.3, 134.7, 134.7, 134.5, 134.1, 133.1, 131.1, 130.8, 130.6, 129.5, 129.2, 128.9, 128.2, 127.8, 127.2, 126.3, 126.1, 125.8, 124.9, 124.7, 123.4, 123.2, 122.8, 121.9, 116.2, 36.9, 31.8.
HRMS (ESI-TOF) calcd for $C_{45}H_{36}N_2NaO^+$ ([M+Na]$^+$): 643.2720, found: 643.2723.
$[\alpha]_D^{20}$ = 1.5 (c = 0.76, $CHCl_3$).
The ee value was determined by HPLC analysis on a IG column (n-hexane/i-PrOH = 80/20, flow = 1.1 mL/min, 254 nm), t = 5.7 min (major), t = 6.7 min (minor). The absolute stereochemistry was assigned by analogy to compound **55**.

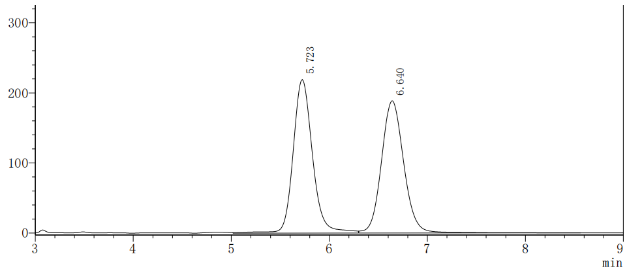

Peak	Ret. Time	Area	Height	Area%
1	5.723	3024014	219150	50.346
2	6.640	2982459	188760	49.654
Total		6006473	407910	100.000

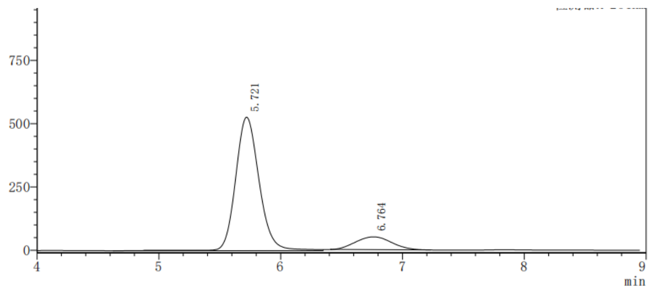

Peak	Ret. Time	Area	Height	Area%
1	5.721	7092795	526558	87.134
2	6.764	1047328	50646	12.866
Total		8140123	577205	100.000

Supplementary Fig. 29 | Enantiomeric excess determination of product 28

(***S***)-5-(2-(tert-butyl)phenyl)-12-(6-fluoropyridin-3-yl)benzo[*c*]phenanthridin-6(5H)-one(**29**)

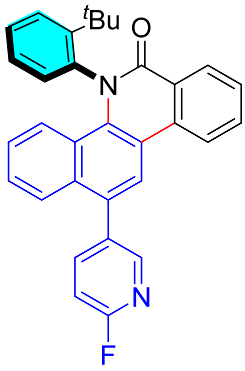

The title compound **29** was prepared according to **General Procedure C** and purified by preparative TLC in hexane/EtOAc = 5/1 as the eluent to afford a white foam (45 mg, 95% yield, 92% ee).

^{1}H NMR (600 MHz, Chloroform-*d*) δ 8.63 – 8.58 (m, 1H), 8.43 (d, *J* = 2.4 Hz, 1H), 8.40 (d, *J* = 8.3 Hz, 1H), 8.31 (s, 1H), 7.98 (td, *J* = 8.0, 2.5 Hz, 1H), 7.89 – 7.82 (m, 1H), 7.79 (dd, *J* = 8.3, 1.6 Hz, 1H), 7.72 – 7.69 (m, 1H), 7.66 (t, *J* = 7.5 Hz, 1H), 7.52 (t, *J* = 7.4 Hz, 2H), 7.39 – 7.32 (m, 1H), 7.31 – 7.27 (m, 1H), 7.13 (dd, *J* = 8.4, 2.8 Hz, 1H), 7.07 (ddd, *J* = 9.0, 6.8, 1.6 Hz, 1H), 6.97 (dd, *J* = 7.9, 1.7 Hz, 1H), 1.29 (s,9H).

^{13}C NMR (151 MHz, Chloroform-*d*) δ 164.3, 163.3 (d, J_{CF} = 239.9 Hz), 148.6, 148.5, 147.6, 143.0 (d, J_{CF} = 7.7 Hz), 140.4, 135.7, 134.4, 133.8, 133.3, 131.3, 130.8, 130.7, 129.3, 129.1, 128.5, 127.9, 126.8, 126.7, 126.2, 125.8, 125.3, 125.0, 122.7 (d, J_{CF} = 20.8 Hz), 116.1, 109.5, 109.3, 36.9, 31.8.

^{19}F NMR (565 MHz, Chloroform-*d*) δ -69.3.

HRMS (ESI-TOF) calcd for $C_{32}H_{25}FN_2NaO^+$ ($[M+Na]^+$): 495.1843, found: 495.1843.

$[\alpha]_D^{20}$ = 8.2 (c = 0.74, $CHCl_3$).

The ee value was determined by HPLC analysis on a IG column (n-hexane/i-PrOH = 80/20, flow = 1.1 mL/min, 254 nm), t = 23.2 min (major), t = 12.9 min (minor). The absolute stereochemistry was assigned by analogy to compound **55**.

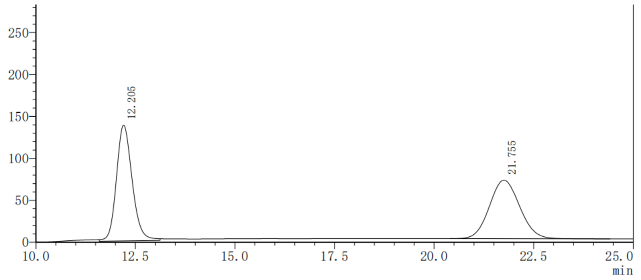

Peak	Ret. Time	Area	Height	Area%
1	12.205	3801445	137961	50.728
2	21.755	3692397	69845	49.272
Total		7493842	207806	100.000

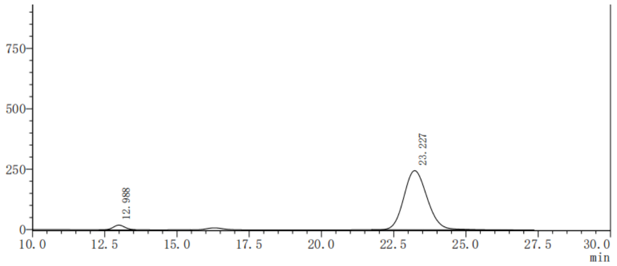

Peak	Ret. Time	Area	Height	Area%
1	12.988	566330	19683	3.950
2	23.227	13772864	244924	96.050
Total		14339195	264607	100.000

Supplementary Fig. 30 | Enantiomeric excess determination of product 29

(***S***)-5-(2-(tert-butyl)phenyl)-12-(thiophen-3-yl)benzo[*c*]phenanthridin-6(5H)-one(**30**)

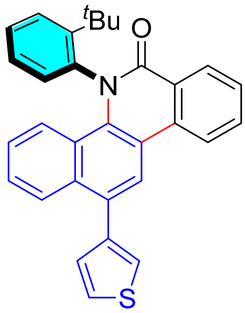

The title compound **30** was prepared according to **General Procedure C** and purified by preparative TLC in hexane/EtOAc = 10/1 as the eluent to afford a colorless oil (41 mg, 90% yield, 91% ee).
^{1}H NMR (600 MHz, Chloroform-*d*) δ 8.59 (dd, *J* = 8.0, 1.6 Hz, 1H), 8.42 (d, *J* = 8.3 Hz, 1H), 8.39 (s, 1H), 7.99 (dd, *J* = 8.5, 1.7 Hz, 1H), 7.84 (ddd, *J* = 8.4, 6.9, 1.6 Hz, 1H), 7.78 (dd, *J* = 8.3, 1.6 Hz, 1H), 7.69 – 7.58 (m, 1H), 7.53 – 7.47 (m, 4H), 7.37 – 7.32 (m, 2H), 7.28 (td, *J* = 7.5, 1.6 Hz, 1H), 7.04 (ddd, *J* = 8.7, 6.7, 1.6 Hz, 1H), 6.97 (dd, *J* = 7.8, 1.7 Hz, 1H), 1.29 (s, 9H).
^{13}C NMR (151 MHz, Chloroform-*d*) δ 164.3, 147.6, 141.1, 140.6, 135.1, 134.7, 134.2, 133.2, 131.4, 130.8, 130.6, 129.9, 129.2, 129.0, 128.3, 127.8, 126.9, 126.3, 126.3, 125.8, 125.0, 124.9, 124.0, 122.7, 122.1, 116.1, 36.9, 31.8.
HRMS (ESI-TOF) calcd for $C_{31}H_{25}SNNaO^+$ ([M+Na]$^+$): 482.1549, found: 482.1548.

$[\alpha]_D^{20}$ = 5.3 (c = 1.27, $CHCl_3$).

The ee value was determined by HPLC analysis on a OD-H column (n-hexane/i-PrOH = 80/20, flow = 1.0 mL/min, 254 nm), t = 8.8 min (major), t = 11.4 min (minor). The absolute stereochemistry was assigned by analogy to compound **55**.

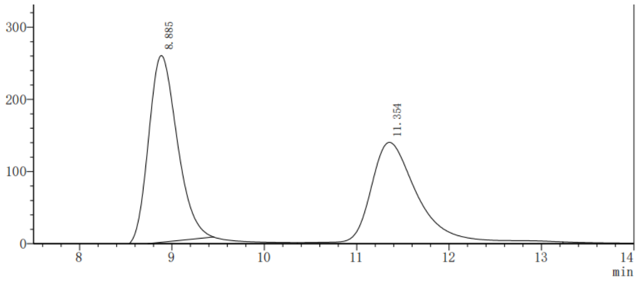

Peak	Ret. Time	Area	Height	Area%
1	8.885	5603637	258955	50.359
2	11.354	5523846	143087	49.641
Total		11127483	402042	100.000

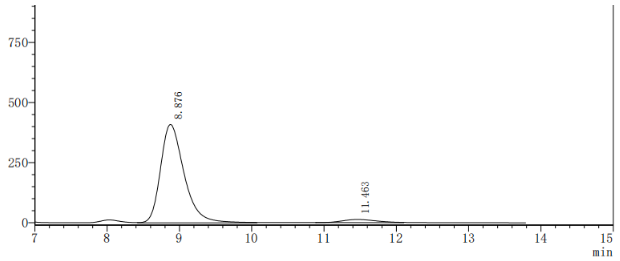

Peak	Ret. Time	Area	Height	Area%
1	8.876	9274693	409101	95.479
2	11.463	439186	13146	4.521
Total		9713879	422247	100.000

Supplementary Fig. 31 | Enantiomeric excess determination of product 30

(***S***)-5-(2-(tert-butyl)phenyl)-12-(thiophen-2-yl)benzo[*c*]phenanthridin-6(5H)-one(**31**)

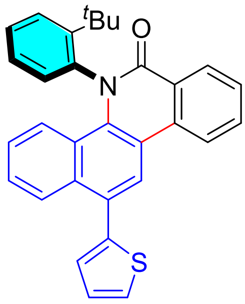

The title compound **31** was prepared according to **General Procedure C** and purified by preparative TLC in hexane/EtOAc = 10/1 as the eluent to afford a yellow oil (30 mg, 65% yield, 90% ee).

^{1}H NMR (600 MHz, Chloroform-*d*) δ 8.59 (d, *J* = 7.8 Hz, 1H), 8.48 (s,1H), 8.42 (d, *J* = 8.3 Hz, 1H), 8.18 (d, *J* = 8.4 Hz, 1H), 7.87 – 7.82 (m, 1H), 7.78 (dd, *J* = 8.2, 1.4 Hz, 1H), 7.65 (t, *J* = 7.6 Hz, 1H), 7.51 (q, *J* = 6.7, 5.0 Hz, 3H), 7.40 – 7.33 (m, 1H), 7.31 – 7.26 (m, 2H), 7.24 (dd, *J* = 5.2, 3.5 Hz, 1H), 7.05 (ddd, *J* = 8.6, 6.7, 1.5 Hz, 1H), 6.96 (dd, *J* = 7.9, 1.5 Hz, 1H), 1.29 (s, 9H).

^{13}C NMR (151 MHz, Chloroform-*d*) δ 164.3, 147.6, 141.7, 140.5, 135.4, 134.5, 134.3, 133.3, 130.7, 130.6, 129.2, 129.0, 128.7, 128.4, 128.0, 127.9, 127.5, 126.8, 126.5, 126.3, 126.0, 125.7, 125.2, 124.9, 123.5, 122.7, 116.0, 36.9, 31.8.

HRMS (ESI-TOF) calcd for $C_{31}H_{25}SNNaO^{+}$ ($[M+Na]^{+}$): 482.1549, found: 482.1550.

$[\alpha]_D^{20}$ = 8.5 (c = 0.69, $CHCl_3$).

The ee value was determined by HPLC analysis on a IG column (n-hexane/i-PrOH = 80/20, flow = 1.1 mL/min, 254 nm), t = 9.5 min (major), t = 6.6 min (minor). The absolute stereochemistry was assigned by analogy to compound **55**.

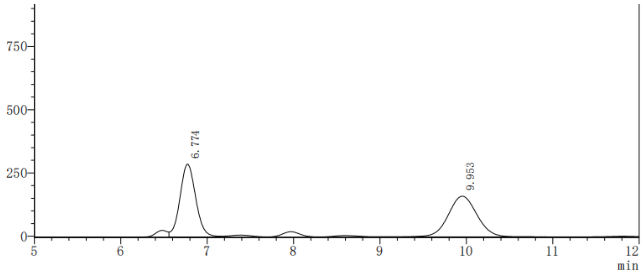

Peak	Ret. Time	Area	Height	Area%
1	6.774	3666176	288360	49.682
2	9.953	3713083	160737	50.318
Total		7379259	449097	100.000

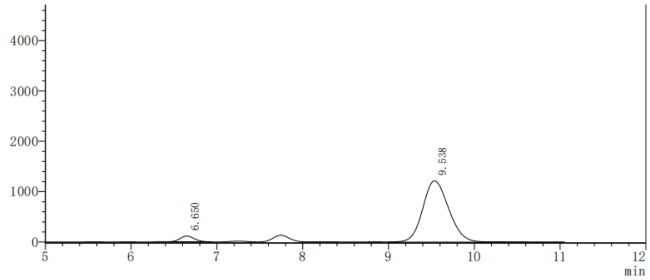

Peak	Ret. Time	Area	Height	Area%
1	6.650	1303190	113543	4.808
2	9.538	25798785	1215954	95.192
Total		27101975	1329497	100.000

Supplementary Fig. 32 | Enantiomeric excess determination of product 31

(***S***)-12-(benzo[*b*]thiophen-3-yl)-5-(2-(tert-butyl)phenyl)benzo[*c*]phenanthridin-6(5H)-one(**32**)

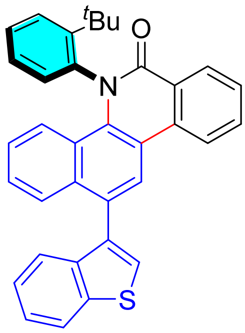

The title compound **32** was prepared according to **General Procedure C** and purified by preparative TLC in hexane/EtOAc = 10/1 as the eluent to afford a orange solid (46 mg, 90% yield, 90% ee). mp:228.3-229.6 °C.

^{1}H NMR (400 MHz, Chloroform-*d*) δ 8.61 (dd, *J* = 7.9, 1.4 Hz, 1H), 8.47 (s, 1H), 8.38 (t, *J* = 6.9 Hz, 1H), 8.01 (d, *J* = 8.3 Hz, 1H), 7.81 (q, *J* = 5.8, 4.1 Hz, 2H), 7.70 (d, *J* = 8.5 Hz, 1H), 7.64 (t, *J* = 7.5 Hz, 1H), 7.61 – 7.48 (m, 3H), 7.46 – 7.39 (m, 2H), 7.31 (p, *J* = 7.3 Hz, 2H), 7.26 – 7.22 (m, 1H), 7.04 (ddd, *J* = 9.7, 7.6, 1.7 Hz, 2H), 1.30 (s, 9H).

^{13}C NMR (101 MHz, Chloroform-*d*) δ 164.3, 147.6, 140.5, 140.1, 139.7, 135.5, 134.6, 133.2, 131.0, 130.8, 130.6, 129.2, 129.0, 128.3, 127.9, 127.8, 127.3, 127.1, 126.3, 125.7, 125.6, 125.2, 124.9, 124.8, 124.5, 123.6, 123.5, 123.1, 123.0, 122.7, 116.2, 36.9, 31.8.

HRMS (ESI-TOF) calcd for $C_{35}H_{27}SNNaO^{+}$ ([M+Na]$^{+}$): 532.1706, found: 532.1707.

$[\alpha]_D^{20}$ = -2.7 (c = 0.6, $CHCl_3$).

The ee value was determined by HPLC analysis on a IG column (n-hexane/i-PrOH = 80/20, flow = 1.1 mL/min, 254 nm), t = 9.5 min (major), t = 6.7 min (minor). The absolute stereochemistry was assigned by analogy to compound **55**.

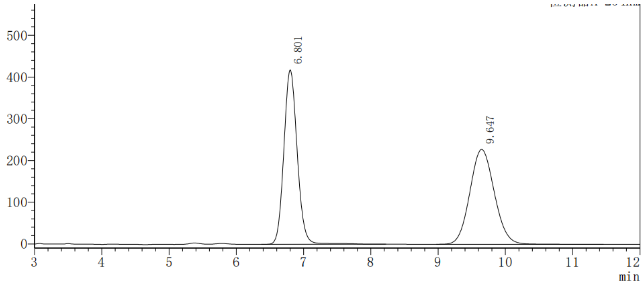

Peak	Ret. Time	Area	Height	Area%
1	6.801	5772342	417821	50.184
2	9.647	5729954	227380	49.816
Total		11502296	645200	100.000

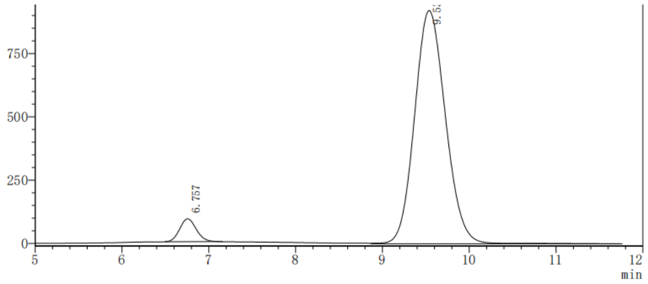

Peak	Ret. Time	Area	Height	Area%
1	6.757	1204841	90273	5.022
2	9.539	22785738	920219	94.978
Total		23990580	1010492	100.000

Supplementary Fig. 33 | Enantiomeric excess determination of product 32

(***S***)-12-(benzofuran-2-yl)-5-(2-(tert-butyl)phenyl)benzo[*c*]phenanthridin-6(5H)-one(**33**)

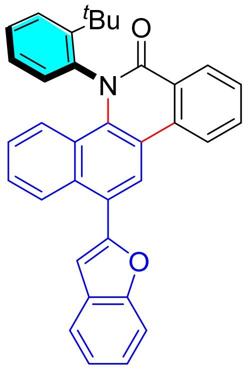

The title compound **33** was prepared according to **General Procedure C** and purified by preparative TLC in hexane/EtOAc = 10/1 as the eluent to afford a orange foam (38 mg, 78% yield, 91% ee).
^{1}H NMR (600 MHz, Chloroform-*d*) δ 8.79 (s, 1H), 8.60 (dd, *J* = 8.0, 1.4 Hz, 1H), 8.50 (d, *J* = 8.3 Hz, 1H), 8.42 (dd, *J* = 8.4, 1.4 Hz, 1H), 7.89 (ddd, *J* = 8.5, 7.0, 1.5 Hz, 1H), 7.79 (dd, *J* = 8.3, 1.5 Hz, 1H), 7.71 (d, *J* = 8.2 Hz, 1H), 7.66 (t, *J* = 8.0 Hz, 2H), 7.57 – 7.50 (m, 2H), 7.46 – 7.41 (m, 1H), 7.41 – 7.37 (m, 1H), 7.34 (t, *J* = 7.4 Hz, 1H), 7.29 (td, *J* = 7.5, 1.6 Hz, 1H), 7.14 (s, 1H), 7.09 (ddd, *J* = 8.8, 6.7, 1.5 Hz, 1H), 6.98 (dd, *J* = 7.6, 1.6 Hz, 1H), 1.28 (s, 9H).
^{13}C NMR (151 MHz, Chloroform-*d*) δ 164.3, 155.3, 155.1, 147.5, 140.4, 136.1, 134.5, 133.4, 133.0, 130.8, 130.7, 129.2, 129.1, 129.1, 128.5, 127.8, 127.0, 126.5, 126.3, 125.7, 125.3, 125.0, 124.8, 124.6, 123.2, 122.9, 122.7, 121.2, 116.1, 111.5, 106.6, 36.9, 31.8.
HRMS (ESI-TOF) calcd for $C_{35}H_{27}NNaO_2^+$ ([M+Na]$^+$): 516.1934, found: 516.1936.
$[\alpha]_D^{20}$ = -11.9 (c = 0.83, $CHCl_3$).
The ee value was determined by HPLC analysis on a IG column (n-hexane/i-PrOH = 80/20, flow = 1.1 mL/min, 254 nm), t = 7.4 min (major), t = 8.2 min (minor). The absolute stereochemistry was assigned by analogy to compound **55**.

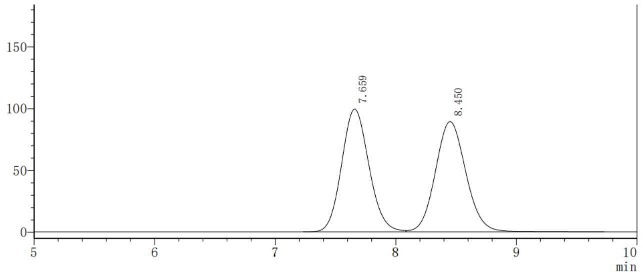

Peak	Ret. Time	Area	Height	Area%
1	7.659	1566618	99376	49.945
2	8.450	1570049	89265	50.055
Total		3136667	188641	100.000

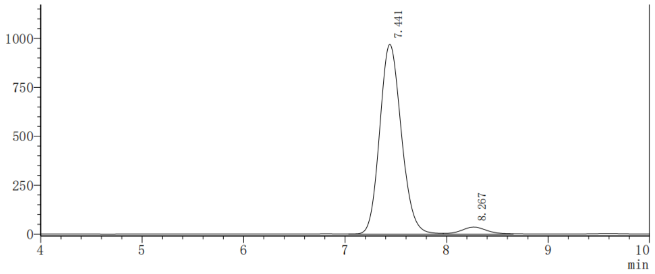

Peak	Ret. Time	Area	Height	Area%
1	7.441	14350242	969646	95.799
2	8.267	629291	35669	4.201
Total		14979533	1005314	100.000

Supplementary Fig. 34 | Enantiomeric excess determination of product 33

(***S***)-5-(2-(tert-butyl)phenyl)-12-(furan-2-yl)benzo[*c*]phenanthridin-6(5H)-one(**34**)

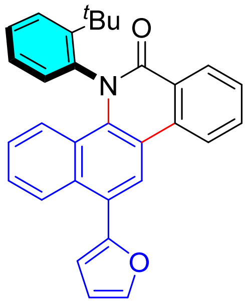

The title compound **34** was prepared according to **General Procedure C** and purified by preparative TLC in hexane/EtOAc = 10/1 as the eluent to afford a yellow foam (42 mg, 94% yield, 90% ee).

^{1}**H NMR (600 MHz, Chloroform-*d*)** δ 8.60 (dd, *J* = 8.0, 1.5 Hz, 1H), 8.43 (d, *J* = 8.3 Hz, 1H), 8.38 (s,1H), 8.12 (dd, *J* = 8.3, 1.5 Hz, 1H), 7.85 (ddd, *J* = 8.4, 7.0, 1.5 Hz, 1H), 7.78 (dd, *J* = 8.2, 1.5 Hz, 1H), 7.74 (s, 1H), 7.68 – 7.61 (m, 1H), 7.54 – 7.46 (m, 2H), 7.37 (ddd, *J* = 8.1, 6.7, 1.1 Hz, 2H), 7.28 (td, *J* = 7.5, 1.5 Hz, 1H), 7.09 – 7.02 (m, 1H), 6.97 (dd, *J* = 7.8, 1.5 Hz, 1H), 6.75 (s, 1H), 1.28 (s, 9H).

^{13}**C NMR (151 MHz, Chloroform-*d*)** δ 164.3, 147.6, 143.2, 140.8, 140.6, 135.1, 134.6, 134.1, 133.2, 130.8, 130.6, 129.2, 129.0, 128.3, 127.8, 127.2, 126.7, 126.4, 126.4, 125.7, 125.1, 124.9, 124.9, 122.7, 122.1, 116.2, 112.8, 36.9, 31.8.

HRMS (ESI-TOF) calcd for $C_{31}H_{25}NNaO_2^+$ ([M+Na]$^+$): 466.1778, found: 466.1777.

$[\alpha]_D^{20}$ = 19.1 (c = 0.76, $CHCl_3$).

The ee value was determined by HPLC analysis on a IG column (n-hexane/i-PrOH = 80/20, flow = 1.1 mL/min, 254 nm), t = 9.9 min (major), t = 7.0 min (minor). The absolute stereochemistry was assigned by analogy to compound **55**.

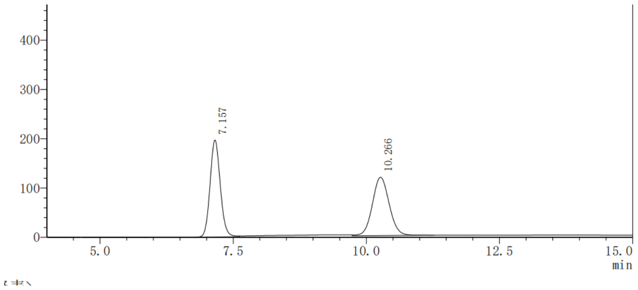

Peak	Ret. Time	Area	Height	Area%
1	7.157	2625391	196671	50.320
2	10.266	2591950	118459	49.680
Total		5217341	315130	100.000

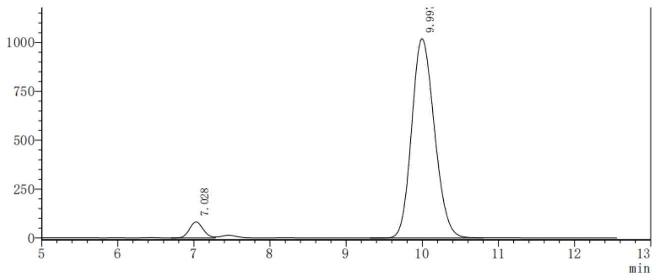

Peak	Ret. Time	Area	Height	Area%
1	7.028	1040734	82253	4.674
2	9.997	21228099	1019421	95.326
Total		22268833	1101674	100.000

Supplementary Fig. 35 | Enantiomeric excess determination of product 34

(***S***)-tert-butyl-2-(5-(2-(tert-butyl)phenyl)-6-oxo-5,6-dihydrobenzo[*c*]phenanthridin-12-yl)-1H-pyrrole-1-carboxylate(**35**)

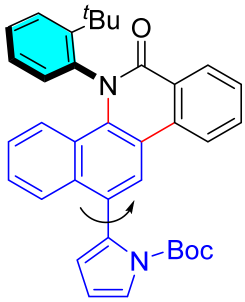

The title compound **35** was prepared according to **General Procedure C** and purified by preparative TLC in hexane/EtOAc = 10/1 as the eluent to afford a yellow foam (24 mg, 45% yield, 1:1.2 dr, 90% ee).

^{1}H NMR (400 MHz, Chloroform-*d*) δ 8.59 (dd, *J* = 8.0, 1.4 Hz, 1.79H), 8.42 (d, *J* = 8.3 Hz, 1.76H), 8.38 (d, *J* = 3.9 Hz, 1.86H), 7.85 (ddd, *J* = 8.4, 7.1, 1.5 Hz, 1.87H), 7.79 (dd, *J* = 8.1, 1.5 Hz, 1.83H), 7.68 – 7.62 (m, 1.9H), 7.58 (td, *J* = 4.2, 1.4 Hz, 2.13H), 7.56 – 7.51 (m, 2.89H), 7.50 – 7.38 (m, 2.51H), 7.33 – 7.28 (m, 1.91H), 7.26 – 7.22 (m, 1H), 7.01 (ddd, *J* = 8.3, 6.8, 1.4 Hz, 1.83H), 6.89 (dd, *J* = 7.9, 1.4 Hz, 0.87H), 6.82 (dd, *J* = 7.9, 1.5 Hz, 1H), 6.44 – 6.36 (m, 3.51H), 1.33 (s, 7.86H), 1.31 (s, 9H), 0.89 (s, 9H), 0.82 (s, 7.7H).

^{13}C NMR (101 MHz, Chloroform-*d*) δ 164.4, 149.4, 149.3, 147.8, 147.7, 140.7, 140.6, 136.1, 136.1, 135.2, 135.2, 134.7, 134.7, 133.2, 132.1, 132.0, 130.7, 130.5, 130.4, 130.2, 129.6, 129.2, 129.2, 129.1, 129.0, 128.2, 128.0, 127.8, 127.1, 126.9, 126.4, 126.3, 126.2, 126.1, 125.6, 124.9, 124.9, 124.5, 124.4, 122.8, 122.7, 122.7, 122.5, 122.4, 122.3, 115.9, 115.9, 115.8, 115.7, 111.0, 111.0, 83.5, 83.4, 37.0, 36.9, 31.8, 27.3, 27.2.

HRMS (ESI-TOF) calcd for $C_{36}H_{34}N_2NaO_3^+$ ([M+Na]$^+$): 565.2462, found: 565.2463.

$[\alpha]_D^{20}$ = -2.1 (c = 0.94, $CHCl_3$).

The ee value was determined by HPLC analysis on a IG column (n-hexane/i-PrOH = 80/20, flow = 1.1 mL/min, 254 nm), t = 7.3 min (major), t = 5.3 min (minor). The absolute stereochemistry was assigned by analogy to compound **55**.

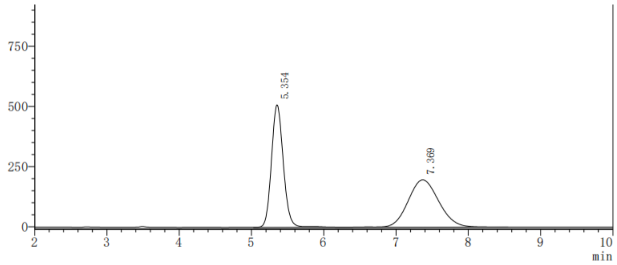

Peak	Ret. Time	Area	Height	Area%
1	5.354	5822536	507878	50.219
2	7.369	5771848	196292	49.781
Total		11594384	704170	100.000

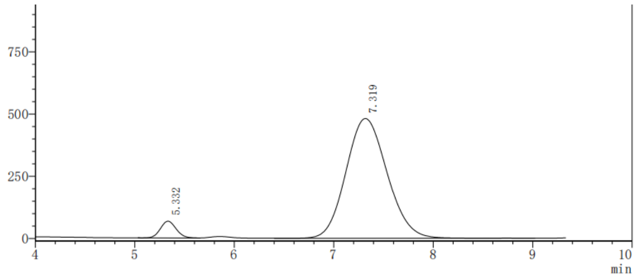

Peak	Ret. Time	Area	Height	Area%
1	5.332	739112	67375	5.043
2	7.319	13917443	481050	94.957
Total		14656555	548425	100.000

Supplementary Fig. 36 | Enantiomeric excess determination of product 35

(***S***)-5-(2-(tert-butyl)phenyl)-12-(1H-pyrrol-2-yl)benzo[*c*]phenanthridin-6(5H)-one(**35'**)

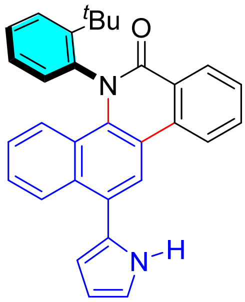

The title compound **35'** was prepared from **35** stirring in TFA then purified by preparative TLC in hexane/EtOAc = 5/1 as the eluent to afford a yellow foam.

^{1}H NMR (400 MHz, Chloroform-*d*) δ 8.82 (s, 1H), 8.53 (dd, *J* = 8.0, 1.4 Hz, 1H), 8.30 (t, *J* = 4.2 Hz, 2H), 8.20 (dd, *J* = 8.5, 1.5 Hz, 1H), 7.84 – 7.76 (m, 1H), 7.74 (dd, *J* = 8.2, 1.5 Hz, 1H), 7.60 (t, *J* = 7.5 Hz, 1H), 7.48 – 7.42 (m, 1H), 7.40 (d, *J* = 9.1 Hz, 1H), 7.35 (dd, *J* = 8.3, 6.7 Hz, 1H), 7.10 (td, *J* = 7.6, 1.4 Hz, 1H), 7.05 – 6.96 (m, 2H), 6.65 (dd, *J* = 7.8, 1.5 Hz, 1H), 6.51 (q, *J* = 2.5, 1.7 Hz, 1H), 6.45 (q, *J* = 2.9 Hz, 1H), 1.24 (s, 9H).

^{13}C NMR (101 MHz, Chloroform-*d*) δ 164.3, 147.4, 140.4, 134.7, 134.5, 134.2, 133.2, 130.7, 130.5, 130.3, 129.1, 128.9, 128.2, 127.8, 126.9, 126.5, 126.3, 125.6, 125.1, 124.9, 122.7, 122.0, 118.7, 116.2, 109.8, 109.4, 100.1, 36.8, 31.7.

HRMS (ESI-TOF) calcd for $C_{31}H_{26}N_2NaO^+$ ($[M+Na]^+$): 465.1937, found: 465.1938.

$[\alpha]_D^{20}$ = 1.7 (c = 0.6, $CHCl_3$).

The ee value was determined by HPLC analysis on a OD-H column (n-hexane/i-PrOH = 75/25, flow = 1.1 mL/min, 254 nm), t = 7.0 min (major), t = 12.3 min (minor). The absolute stereochemistry was assigned by analogy to compound **55**.

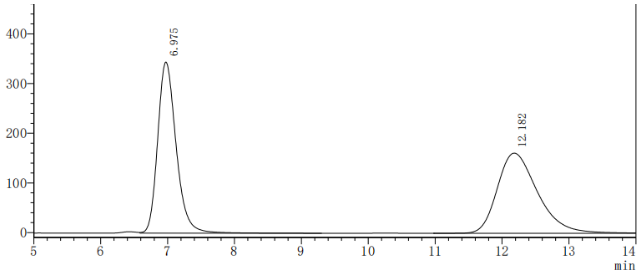

Peak	Ret. Time	Area	Height	Area%
1	6.975	6622412	344530	49.516
2	12.182	6751875	161271	50.484
Total		13374287	505801	100.000

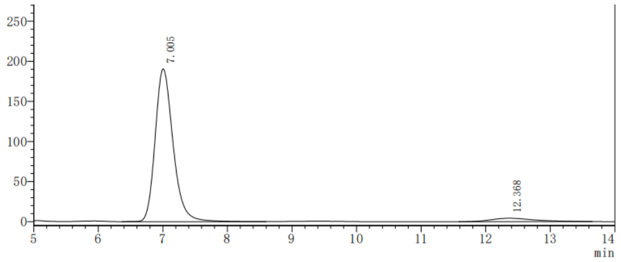

Peak	Ret. Time	Area	Height	Area%
1	7.005	3697676	190577	94.844
2	12.368	201024	4295	5.156
Total		3898700	194871	100.000

Supplementary Fig. 37 | Enantiomeric excess determination of product 35'

(***S***)-5-(2-(tert-butyl)phenyl)-9-fluorobenzo[*c*]phenanthridin-6(5H)-one(**36**)

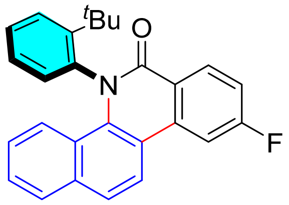

The title compound **36** was prepared according to **General Procedure C** and purified by preparative TLC in hexane/EtOAc = 10/1 as the eluent to afford a yellow oil (30 mg, 76% yield, 91% ee).

^{1}H NMR (400 MHz, Chloroform-*d*) δ 8.60 (dd, *J* = 8.8, 6.1 Hz, 1H), 8.24 (d, *J* = 8.8 Hz, 1H), 8.04 (dd, *J* = 10.8, 2.3 Hz, 1H), 7.83 (d, *J* = 8.1 Hz, 1H), 7.77 (dt, *J* = 8.4, 2.2 Hz, 2H), 7.52 (t, *J* = 7.7 Hz, 1H), 7.39 (t, *J* = 7.5 Hz, 1H), 7.36 – 7.27 (m, 2H), 7.23 (d, *J* = 9.2 Hz, 1H), 7.06 – 6.94 (m, 2H), 1.20 (s, 9H).

^{13}C NMR (101 MHz, Chloroform-*d*) δ 166.2 (d, J_{CF} = 252.1 Hz), 163.6, 147.5, 140.2, 137.4 (d, J_{CF} = 9.5 Hz), 136.2, 135.9, 132.5 (d, J_{CF} = 9.9 Hz), 131.1, 130.6, 129.1, 129.1, 127.9, 126.6, 126.2, 125.5, 125.1, 124.5, 122.1, 120.7, 116.6 (d, J_{CF} = 23.2 Hz), 115.8, 108.5 (d, J_{CF} = 23.5 Hz), 36.7, 31.7.

^{19}F NMR (376 MHz, Chloroform-*d*) δ -104.9.

HRMS (ESI-TOF) calcd for $C_{27}H_{22}NFNaO^+$ ($[M+Na]^+$): 418.1578, found: 418.1579.

$[\alpha]_D^{20}$ = 41.4 (c = 0.8, $CHCl_3$).

The ee value was determined by HPLC analysis on a IE column (n-hexane/i-PrOH = 70/30, flow = 1.1 mL/min, 254 nm), t = 8.9 min (major), t = 7.6 min (minor). The absolute stereochemistry was assigned by analogy to compound **55**.

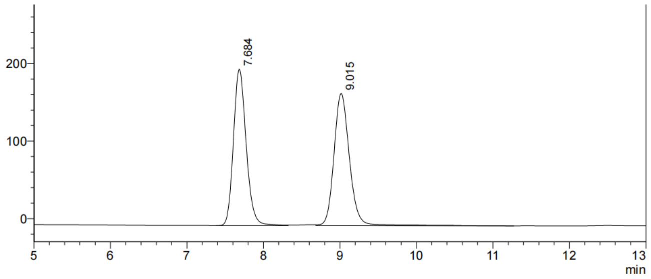

Peak	Ret. Time	Area	Height	Area%
1	7.684	2336094	201666	49.039
2	9.015	2427677	170493	50.961
Total		4763771	372159	100.000

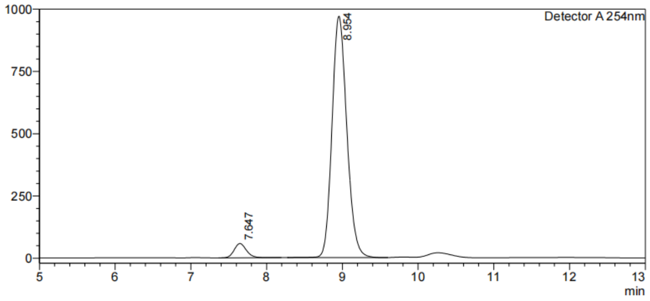

Peak	Ret. Time	Area	Height	Area%
1	7.647	646045	57089	4.677
2	8.954	13166380	968310	95.323
Total		13812425	1025399	100.000

Supplementary Fig. 38 | Enantiomeric excess determination of product 36

(***S***)-5-(2-(tert-butyl)phenyl)-9-methylbenzo[*c*]phenanthridin-6(5H)-one(**37**)

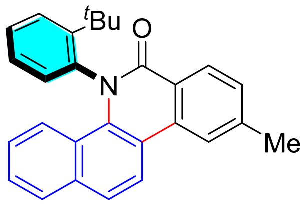

The title compound **37** was prepared according to **General Procedure D** and purified by preparative TLC in hexane/EtOAc = 10/1 as the eluent to afford a white foam (20 mg, 52% yield, 90% ee).

^{1}H NMR (400 MHz, Chloroform-*d*) δ 8.48 (d, *J* = 8.1 Hz, 1H), 8.41 (d, *J* = 8.8 Hz, 1H), 8.22 (s, 1H), 7.82 (dd, *J* = 8.1, 1.5 Hz, 1H), 7.76 (d, *J* = 8.5 Hz, 2H), 7.54 – 7.48 (m, 1H), 7.46 (d, *J* = 8.1 Hz, 1H), 7.36 (t, *J* = 7.4 Hz, 1H), 7.29 (td, *J* = 7.5, 1.5 Hz, 1H), 7.23 (d, *J* = 9.1 Hz, 1H), 7.01 (ddt, *J* = 8.9, 6.1, 3.0 Hz, 2H), 2.63 (s, 3H), 1.20 (s, 9H).

^{13}C NMR (101 MHz, Chloroform-*d*) δ 164.2, 147.5, 143.7, 140.5, 135.6, 134.8, 131.2, 130.5, 129.6, 129.2, 129.0, 128.9, 127.7, 126.1, 126.1, 125.2, 124.7, 124.6, 123.4, 122.7, 120.8, 116.6, 36.7, 31.7, 22.5.

HRMS (ESI-TOF) calcd for $C_{28}H_{25}NNaO^{+}$ ($[M+Na]^{+}$): 414.1828, found: 414.1830.

$[\alpha]_D^{20}$ = 33.6 (c = 0.74, $CHCl_3$).

The ee value was determined by HPLC analysis on a IG column (n-hexane/i-PrOH = 80/20, flow = 1.1 mL/min, 254 nm), t = 16.7 min (major), t = 11.5 min (minor). The absolute stereochemistry was assigned by analogy to compound **55**.

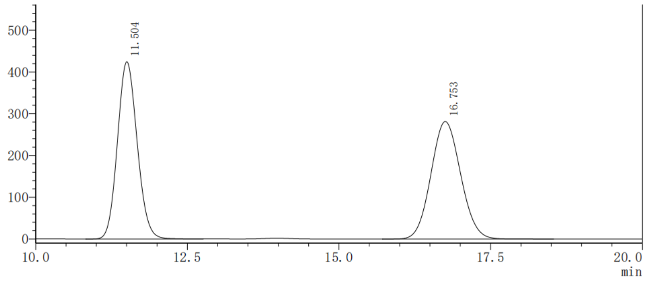

Peak	Ret. Time	Area	Height	Area%
1	11.504	9623536	424329	50.089
2	16.753	9589369	281277	49.911
Total		19212905	705607	100.000

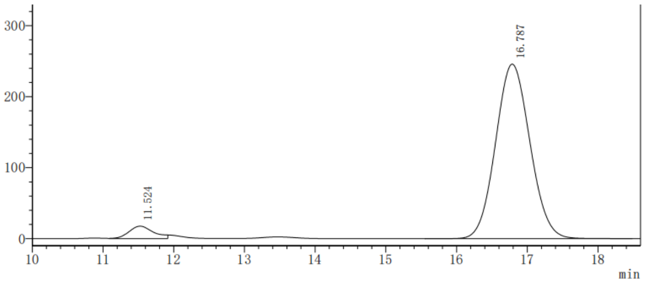

Peak	Ret. Time	Area	Height	Area%
1	11.524	428723	17586	4.900
2	16.787	8320577	245750	95.100
Total		8749300	263336	100.000

Supplementary Fig. 39 | Enantiomeric excess determination of product 37

(***S***)-5-(2-(tert-butyl)phenyl)-8-nitrobenzo[*c*]phenanthridin-6(5H)-one(**38**)

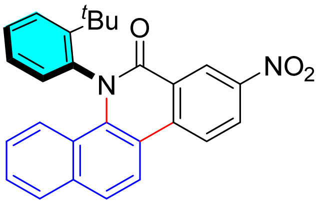

The title compound **38** was prepared according to **General Procedure C** and purified by preparative TLC in hexane/EtOAc = 5/1 as the eluent to afford a yellow solid (30 mg, 72% yield, 90% ee). mp:227.2-228 °C.

^{1}H NMR (400 MHz, Chloroform-*d*) δ 9.40 (d, *J* = 2.4 Hz, 1H), 8.63 (dd, *J* = 9.0, 2.5 Hz, 1H), 8.57 (d, *J* = 9.1 Hz, 1H), 8.37 (d, *J* = 8.9 Hz, 1H), 7.86 (d, *J* = 8.1 Hz, 1H), 7.82 (d, *J* = 8.9 Hz, 1H), 7.79 (dd, *J* = 8.2, 1.4 Hz, 1H), 7.58 – 7.51 (m, 1H), 7.44 (t, *J* = 7.4 Hz, 1H), 7.32 (td, *J* = 7.5, 1.4 Hz, 1H), 7.28 – 7.23 (m, 1H), 7.06 (ddd, *J* = 8.7, 6.7, 1.5 Hz, 1H), 6.98 (dd, *J* = 7.8, 1.4 Hz, 1H), 1.21 (s, 9H).

^{13}C NMR (101 MHz, Chloroform-*d*) δ 163.1, 147.4, 147.1, 139.7, 137.5, 136.6, 130.9, 130.7, 129.5, 129.2, 128.1, 127.5, 127.0, 126.5, 125.9, 125.8, 125.7, 125.4, 124.5, 124.4, 120.8, 115.2, 36.8, 31.7.

HRMS (ESI-TOF) calcd for $C_{27}H_{22}N_2NaO_3^+$ ([M+Na]$^+$): 445.1523, found: 445.1524.

$[\alpha]_D^{20}$ = 37.9 (c = 0.82, $CHCl_3$).

The ee value was determined by HPLC analysis on a IE column (n-hexane/i-PrOH = 70/30, flow = 1.1 mL/min, 254 nm), t = 19.4 min (major), t = 23.5 min (minor). The absolute stereochemistry was assigned by analogy to compound **55**.

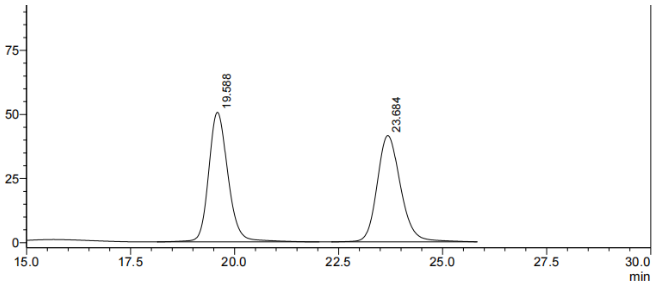

Peak	Ret. Time	Area	Height	Area%
1	19.588	1641568	50550	50.186
2	23.684	1629376	41454	49.814
Total		3270944	92004	100.000

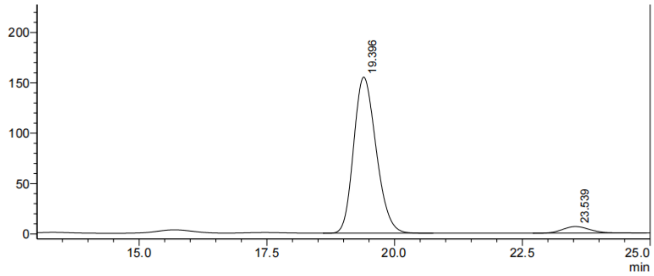

Peak	Ret. Time	Area	Height	Area%
1	19.396	4742726	154889	95.072
2	23.539	245850	6525	4.928

Total		4988576	161414	100.000

Supplementary Fig. 40 | Enantiomeric excess determination of product 38

(***S***)-5-(2-(tert-butyl)phenyl)-8-methoxybenzo[*c*]phenanthridin-6(5H)-one(**39**)

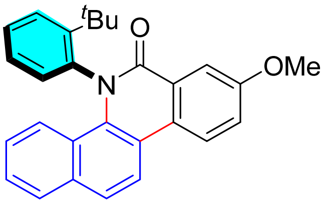

The title compound **39** was prepared according to **General Procedure C** and purified by preparative TLC in hexane/EtOAc = 10/1 as the eluent to afford a white foam (21 mg, 51% yield, 89% ee).

^{1}H NMR (600 MHz, Chloroform-*d*) δ 8.36 (t, J = 8.3 Hz, 2H), 7.99 (m, 1H), 7.82 (d, J = 8.0 Hz, 1H), 7.76 (d, J = 9.2 Hz, 2H), 7.51 (t, J = 7.8 Hz, 1H), 7.45 (dd, J = 9.0, 2.7 Hz, 1H), 7.35 (t, J = 7.4 Hz, 1H), 7.29 (t, J = 7.6 Hz, 1H), 7.20 (d, J = 9.1 Hz, 1H), 7.00 (m, 2H), 3.98 (s, 3H), 1.20 (s, 9H).

^{13}C NMR (151 MHz, Chloroform-*d*) δ 164.0, 159.8, 147.6, 140.6, 135.1, 134.1, 131.1, 130.6, 129.1, 129.0, 128.5, 127.8, 126.9, 125.8, 125.3, 125.0, 124.7, 124.6, 123.4, 120.7, 116.9, 109.1, 55.9, 36.8, 31.7.

HRMS (ESI-TOF) calcd for $C_{28}H_{25}NNaO_2^+$ ([M+Na]$^+$): 430.1778, found: 430.1777.

$[\alpha]_D^{20}$ = 42.8 (c = 0.95, $CHCl_3$).

The ee value was determined by HPLC analysis on a AD-H column (n-hexane/i-PrOH = 92/8, flow = 1.1 mL/min, 254 nm), t = 8.4 min (major), t = 7.6 min (minor). The absolute stereochemistry was assigned by analogy to compound **55**.

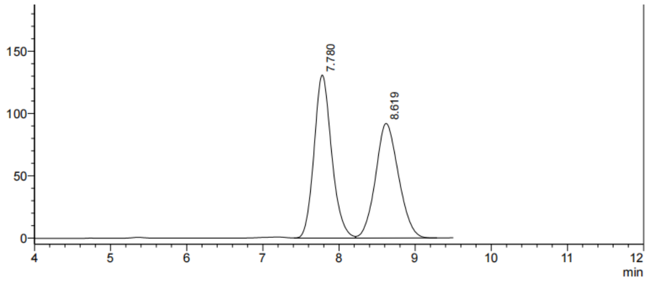

Peak	Ret. Time	Area	Height	Area%
1	7.780	2106659	130905	52.126
2	8.619	1934843	92038	47.874
Total		4041503	222943	100.000

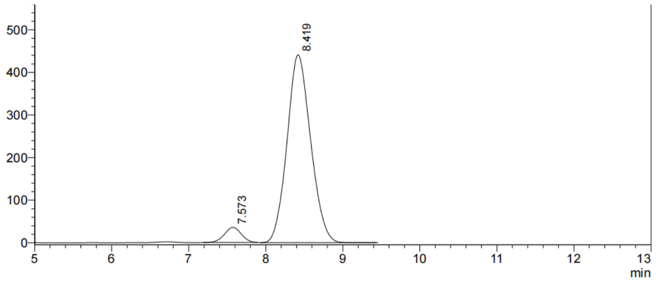

Peak	Ret. Time	Area	Height	Area%

1	7.573	538308	35336	5.413
2	8.419	9406754	441122	94.587
Total		9945063	476458	100.000

Supplementary Fig. 41 | Enantiomeric excess determination of product 39

(***S***)-5-(2-(tert-butyl)phenyl)-8,9-difluorobenzo[*c*]phenanthridin-6(5H)-one(**40**)

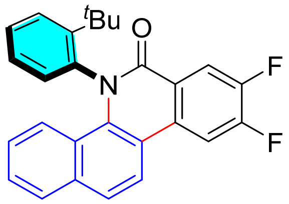

The title compound **40** was prepared according to **General Procedure C** and purified by preparative TLC in hexane/EtOAc = 10/1 as the eluent to afford a yellow foam (33 mg, 79% yield, 89% ee).
^{1}H NMR (400 MHz, Chloroform-*d*) δ 8.34 (dd, *J* = 10.4, 8.4 Hz, 1H), 8.20 – 8.12 (m, 2H), 7.84 (dd, *J* = 8.1, 1.5 Hz, 1H), 7.80 – 7.74 (m, 2H), 7.57 – 7.49 (m, 1H), 7.42 – 7.37 (m, 1H), 7.30 (td, *J* = 7.5, 1.5 Hz, 1H), 7.21 (d, *J* = 9.2 Hz, 1H), 7.06 – 6.95 (m, 2H), 1.19 (s, 9H).
^{13}C NMR (101 MHz, Chloroform-*d*) δ 162.7, 154.6 (dd, J_{CF} = 255.5, 14.1 Hz), 150.7 (dd, J_{CF} = 254.1, 13.6 Hz), 147.4, 143.5, 140.0, 135.8, 132.9 (dd, J_{CF} = 7.8, 2.3 Hz), 130.9, 130.7, 129.3, 129.1, 129.1, 128.5, 127.9, 126.7, 126.1, 125.7, 125.4, 122.8 (d, J_{CF} = 6.1 Hz), 120.6, 117.3 (d, J_{CF} = 18.6 Hz), 115.3, 111.3 (d, J_{CF} = 19.2 Hz), 36.8, 31.7.
^{19}F NMR (376 MHz, Chloroform-*d*) δ -128.69, -135.8.
HRMS (ESI-TOF) calcd for $C_{27}H_{21}NF_2NaO^+$ ([M+Na]$^+$): 436.1483, found: 436.1482.
$[\alpha]_D^{20}$ = 37.4 (c = 1.0, $CHCl_3$).
The ee value was determined by HPLC analysis on a IE column (n-hexane/i-PrOH = 70/30, flow = 1.1 mL/min, 254 nm), t = 7.9 min (major), t = 7.2 min (minor). The absolute stereochemistry was assigned by analogy to compound **55**.

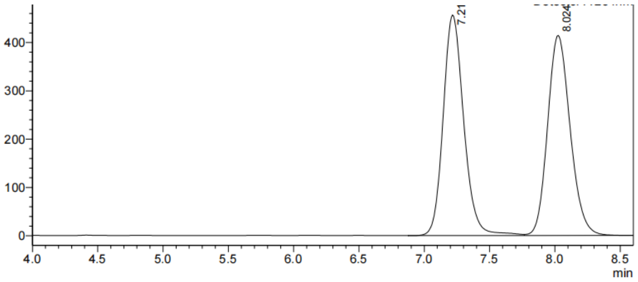

Peak	Ret. Time	Area	Height	Area%
1	7.217	5010697	456635	50.143
2	8.024	4982085	414339	49.857
Total		9992782	870974	100.000

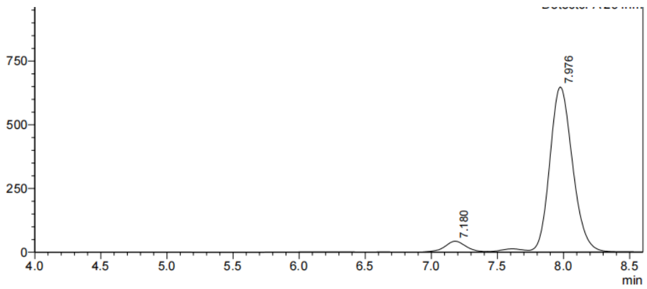

Peak	Ret. Time	Area	Height	Area%
1	7.180	468174	42315	5.609
2	7.976	7879224	647294	94.391
Total		8347398	689609	100.000

Supplementary Fig. 42 | Enantiomeric excess determination of product 40

(***S***)-5-(2-(tert-butyl)phenyl)-8-fluoro-9-methylbenzo[*c*]phenanthridin-6(5H)-one(**41**)

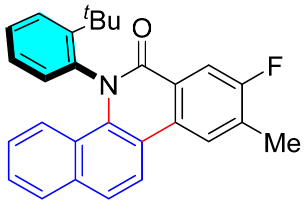

The title compound **41** was prepared according to **General Procedure C** and purified by preparative TLC in hexane/EtOAc = 10/1 as the eluent to afford a yellow foam (31 mg, 77% yield, 89% ee).

^{1}H NMR (600 MHz, Chloroform-*d*) δ 8.33 (d, J = 8.8 Hz, 1H), 8.25 (d, J = 6.7 Hz, 1H), 8.16 (d, J = 9.6 Hz, 1H), 7.82 (d, J = 8.1 Hz, 1H), 7.76 (d, J = 8.5 Hz, 2H), 7.52 (t, J = 7.7 Hz, 1H), 7.37 (t, J = 7.4 Hz, 1H), 7.30 (t, J = 7.4 Hz, 1H), 7.21 (d, J = 9.1 Hz, 1H), 7.01 (dd, J = 12.8, 7.9 Hz, 2H), 2.56 (s, 3H), 1.20 (s, 9H).

^{13}C NMR (151 MHz, Chloroform-*d*) δ 163.4, 161.5 (d, J_{CF} = 248.6 Hz), 147.5, 140.6, 135.4, 135.0, 132.2 (d, J_{CF} = 19.3 Hz), 131.2, 131.1, 130.6, 129.1 (d, J_{CF} = 8.9 Hz), 128.5, 127.8, 126.2, 126.0 (d, J_{CF} = 4.7 Hz), 126.0, 125.5 (d, J_{CF} = 8.4 Hz), 125.4, 125.0, 124.6, 120.7, 116.1, 113.8 (d, J_{CF} = 23.8 Hz), 36.7, 31.7, 15.9 (d, J_{CF} = 3.2 Hz).

^{19}F NMR (565 MHz, Chloroform-*d*) δ -116.3.

HRMS (ESI-TOF) calcd for $C_{28}H_{24}NFNaO^{+}$ ([M+Na]$^{+}$): 432.1734, found: 432.1736.

$[\alpha]_D^{20}$ = 41.3 (c = 0.89, $CHCl_3$).

The ee value was determined by HPLC analysis on a AD-H column (n-hexane/i-PrOH = 80/20, flow = 1.1 mL/min, 254 nm), t = 7.6 min (major), t = 6.1 min (minor). The absolute stereochemistry was assigned by analogy to compound **55**.

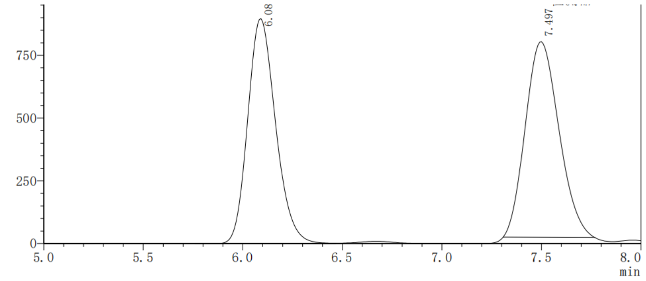

Peak	Ret. Time	Area	Height	Area%

1	6.089	8736754	896365	48.864
2	7.497	9143071	778755	51.136
Total		17879826	1675120	100.000

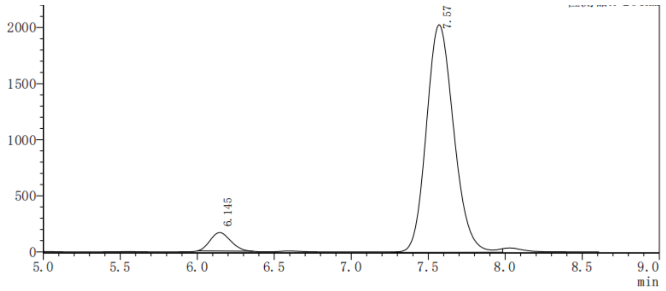

Peak	Ret. Time	Area	Height	Area%
1	6.145	1526890	164444	5.730
2	7.571	25122510	2027671	94.270
Total		26649400	2192114	100.000

Supplementary Fig. 43 | Enantiomeric excess determination of product 41

(***S***)-5-(2-(tert-butyl)phenyl)-8,9-dimethoxybenzo[*c*]phenanthridin-6(5H)-one(**42**)

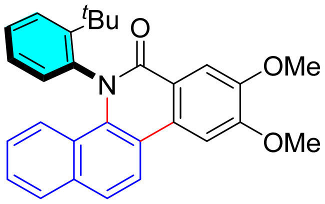

The title compound **42** was prepared according to **General Procedure D** and purified by preparative TLC in hexane/EtOAc = 4/1 as the eluent to afford a white foam (16 mg, 37% yield, 88% ee).
^{1}H NMR (600 MHz, Chloroform-*d*) δ 8.29 (d, J = 8.7 Hz, 1H), 7.97 (s, 1H), 7.82 (d, J = 8.1 Hz, 1H), 7.75 (d, J = 8.0 Hz, 3H), 7.51 (t, J = 7.8 Hz, 1H), 7.36 (t, J = 7.4 Hz, 1H), 7.29 (t, J = 7.6 Hz, 1H), 7.22 (d, J = 9.2 Hz, 1H), 7.00 (d, J = 7.9 Hz, 2H), 4.16 (s, 3H), 4.05 (s, 3H), 1.20 (s, 9H).
^{13}C NMR (151 MHz, Chloroform-*d*) δ 163.6, 154.0, 150.3, 147.6, 140.6, 135.2, 134.8, 131.2, 130.5, 129.8, 129.0, 128.9, 127.7, 126.0, 125.9, 125.3, 124.8, 124.7, 120.6, 119.8, 116.5, 109.1, 103.5, 56.4, 56.4, 36.7, 31.7.
HRMS (ESI-TOF) calcd for $C_{29}H_{27}NNaO_3^+$ ([M+Na]$^+$): 460.1883, found: 460.1884.
$[\alpha]_D^{20}$ = 57.5 (c = 0.67, $CHCl_3$).
The ee value was determined by HPLC analysis on a OD-H column (n-hexane/i-PrOH = 70/30, flow = 1.1 mL/min, 254 nm), t = 6.6 min (major), t = 7.7 min (minor). The absolute stereochemistry was assigned by analogy to compound **55**.

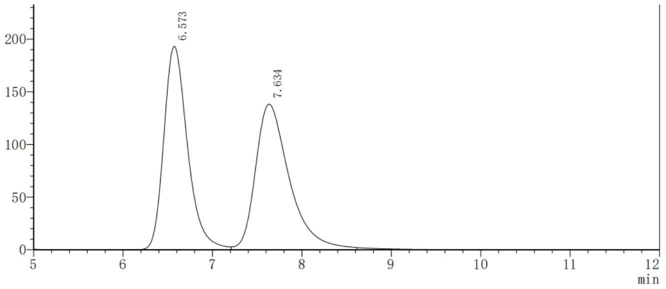

Peak	Ret. Time	Area	Height	Area%
1	6.573	3575372	193287	49.453
2	7.634	3654441	138388	50.547
Total		7229812	331675	100.000

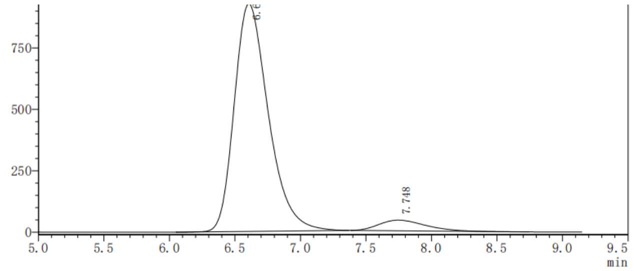

Peak	Ret. Time	Area	Height	Area%
1	6.606	16769181	926819	93.951
2	7.748	1079716	43418	6.049
Total		17848897	970237	100.000

Supplementary Fig. 44 | Enantiomeric excess determination of product 42

(***S***)-5-(4-bromo-2-(tert-butyl)phenyl)benzo[*c*]phenanthridin-6(5H)-one(**43**)

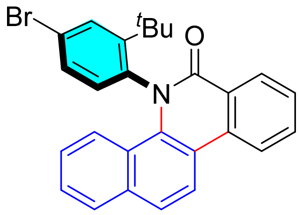

The title compound **43** was prepared according to **General Procedure C** and purified by preparative TLC in hexane/EtOAc = 10/1 as the eluent to afford a yellow solid (32 mg, 70% yield, 79% ee).

^{1}H NMR (600 MHz, Chloroform-*d*) δ 8.57 (dd, *J* = 7.9, 1.5 Hz, 1H), 8.41 (dd, *J* = 15.4, 8.6 Hz, 2H), 7.88 (d, *J* = 2.3 Hz, 1H), 7.87 – 7.83 (m, 2H), 7.78 (d, *J* = 8.8 Hz, 1H), 7.64 (t, *J* = 7.4 Hz, 1H), 7.44 – 7.38 (m, 2H), 7.35 (d, *J* = 9.1 Hz, 1H), 7.11 (ddd, *J* = 8.8, 6.8, 1.6 Hz, 1H), 6.87 (d, *J* = 8.4 Hz, 1H), 1.21 (s, 9H).

^{13}C NMR (151 MHz, Chloroform-*d*) δ 164.2, 150.1, 139.8, 135.7, 135.1, 134.8, 133.8, 133.4, 132.8, 131.0, 129.2, 128.5, 128.3, 126.4, 125.8, 125.6, 125.5, 125.1, 124.4, 123.0, 122.8, 120.8, 116.9, 37.0, 31.5.

HRMS (ESI-TOF) calcd for $C_{27}H_{22}NBrNaO^{+}$ ($[M+Na]^{+}$): 478.0777, found: 478.0776.

$[\alpha]_D^{20}$ = 26.2 (c = 0.88, $CHCl_3$).

The ee value was determined by HPLC analysis on a IG column (n-hexane/i-PrOH = 80/20, flow = 1.1 mL/min, 254 nm), t = 11.6 min (major), t = 10.5 min (minor). The absolute stereochemistry was assigned by analogy to compound **55**.

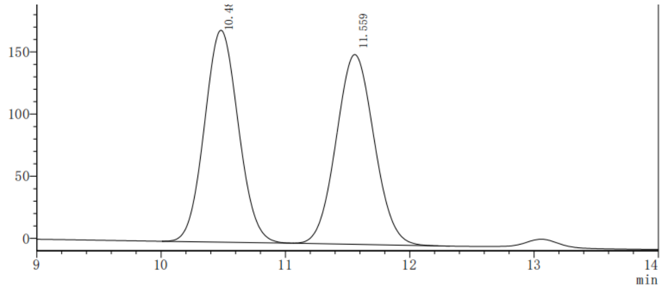

Peak	Ret. Time	Area	Height	Area%
1	10.482	3241881	170513	49.912
2	11.559	3253314	152691	50.088
Total		6495194	323203	100.000

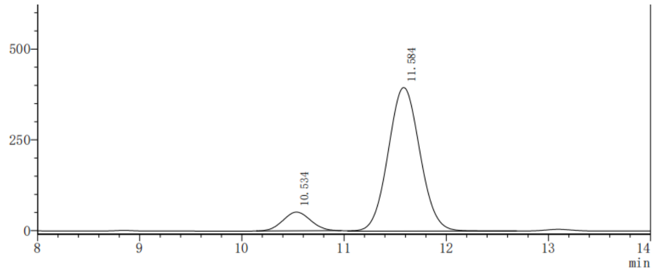

Peak	Ret. Time	Area	Height	Area%
1	10.534	974936	51621	10.274
2	11.584	8514387	395251	89.726
Total		9489323	446871	100.000

Supplementary Fig. 45 | Enantiomeric excess determination of product 43

(***S***)-5-(2-(tert-butyl)-4-(phenylethynyl)phenyl)benzo[*c*]phenanthridin-6(5H)-one(**44**)

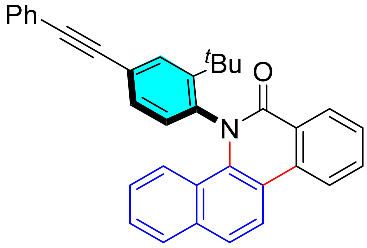

The title compound **44** was prepared according to **General Procedure C** and purified by preparative TLC in hexane/EtOAc = 10/1 as the eluent to afford a yellow foam (31 mg, 65% yield, 88% ee).

^{1}H NMR (600 MHz, Chloroform-*d*) δ 8.59 (dd, *J* = 8.0, 1.5 Hz, 1H), 8.42 (dd, *J* = 15.4, 8.6 Hz, 2H), 7.94 (d, *J* = 2.0 Hz, 1H), 7.89 – 7.83 (m, 2H), 7.79 (d, *J* = 8.9 Hz, 1H), 7.65 (t, *J* = 7.5 Hz, 1H), 7.61 (dd, *J* = 7.7, 1.8 Hz, 2H), 7.46 (dd, *J* = 8.0, 1.9 Hz, 1H), 7.39 (dq, *J* = 7.9, 5.9 Hz, 5H), 7.13 – 7.06 (m, 1H), 7.00 (d, *J* = 7.9 Hz, 1H), 1.25 (s, 9H).

^{13}C NMR (151 MHz, Chloroform-*d*) δ 164.2, 148.0, 140.7, 135.7, 135.3, 134.9, 134.1, 133.3, 131.9, 131.4, 130.8, 129.2, 129.1, 128.6, 128.2, 126.4, 126.0, 125.6, 125.5, 125.1, 124.5, 124.1, 123.3, 122.7, 120.8, 116.8, 90.1, 89.5, 36.9, 31.7.

HRMS (ESI-TOF) calcd for $C_{35}H_{27}NNaO^+$ ($[M+Na]^+$): 500.1985, found: 500.1987.
$[\alpha]_D^{20}$ = 3.1 (c = 0.59, $CHCl_3$).
The ee value was determined by HPLC analysis on a IG column (n-hexane/i-PrOH = 80/20, flow = 1.1 mL/min, 254 nm), t = 18.3 min (major), t = 12.4 min (minor). The absolute stereochemistry was assigned by analogy to compound **55**.

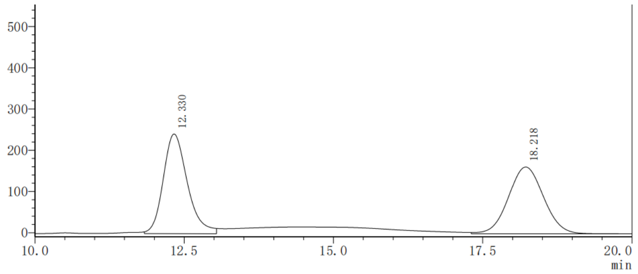

Peak	Ret. Time	Area	Height	Area%
1	12.330	6807774	241718	50.636
2	18.218	6636804	161997	49.364
Total		13444578	403715	100.000

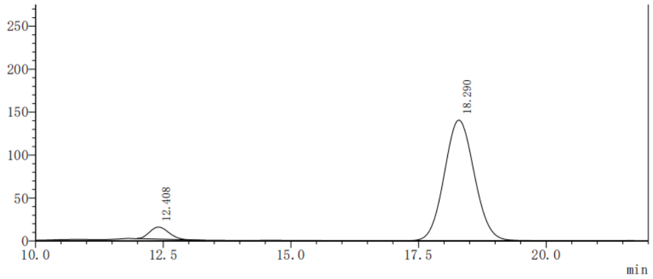

Peak	Ret. Time	Area	Height	Area%
1	12.408	364685	14013	5.982
2	18.290	5731251	140280	94.018
Total		6095936	154294	100.000

Supplementary Fig. 46 | Enantiomeric excess determination of product 44

(***S***)-5-(2,5-di-tert-butylphenyl)benzo[*c*]phenanthridin-6(5H)-one(**45**)

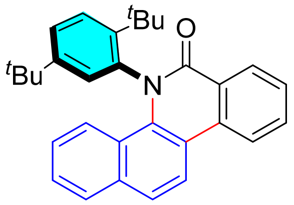

The title compound **45** was prepared according to **General Procedure D** and purified by preparative TLC in hexane/EtOAc = 10/1 as the eluent to afford a yellow foam (24 mg, 55% yield, 90% ee).
^{1}H NMR (600 MHz, Chloroform-*d*) δ 8.60 (d, *J* = 7.8 Hz, 1H), 8.43 (dd, *J* = 15.8, 8.5 Hz, 2H), 7.86 (t, *J* = 7.8 Hz, 1H), 7.82 (d, *J* = 8.0 Hz, 1H), 7.77 (d, *J* = 8.6 Hz, 1H), 7.70 – 7.61 (m, 2H), 7.56 – 7.52 (m, 1H), 7.35 (t, *J* = 7.4 Hz, 1H), 7.06 – 7.00 (m, 2H), 6.96 (t, *J* = 8.1 Hz, 1H), 1.22 (s, 9H), 1.15 (s, 9H).

^{13}C NMR (151 MHz, Chloroform-*d*) δ 164.2, 150.8, 144.1, 139.7, 135.7, 135.7, 134.9, 133.1, 130.2, 129.2, 128.9, 128.5, 128.3, 128.1, 126.3, 126.2, 126.0, 125.7, 125.1, 124.8, 122.7, 120.8, 116.6, 36.2, 34.3, 31.7, 31.2.

HRMS (ESI-TOF) calcd for $C_{31}H_{31}NNaO^+$ ($[M+Na]^+$): 456.2298, found: 456.2299.

$[\alpha]_D^{20}$ = 132.9 (c = 0.86, $CHCl_3$).

The ee value was determined by HPLC analysis on a OD-H column (n-hexane/i-PrOH = 75/25, flow = 1.1 mL/min, 254 nm), t = 3.5 min (major), t = 4.0 min (minor). The absolute stereochemistry was assigned by analogy to compound **55**.

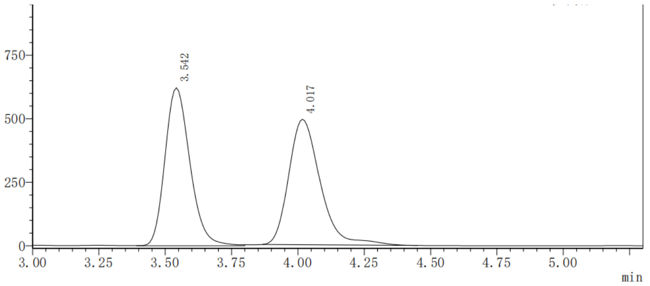

Peak	Ret. Time	Area	Height	Area%
1	3.542	4075350	621761	49.991
2	4.017	4076816	493448	50.009
Total		8152165	1115209	100.000

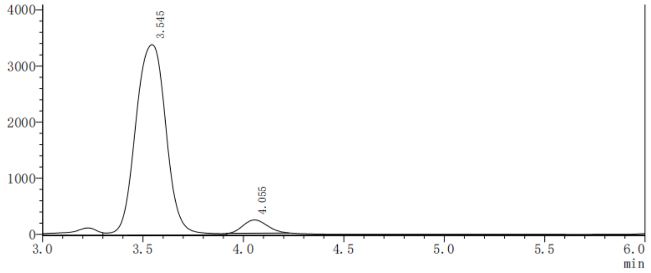

Peak	Ret. Time	Area	Height	Area%
1	3.545	35704653	3397783	94.734
2	4.055	1984631	236547	5.266
Total		37689283	3634330	100.000

Supplementary Fig. 47 | Enantiomeric excess determination of product 45

(***S***)-5-(3'-acetyl-3-(tert-butyl)-[1,1'-biphenyl]-4-yl)benzo[*c*]phenanthridin-6(5H)-one(**46**)

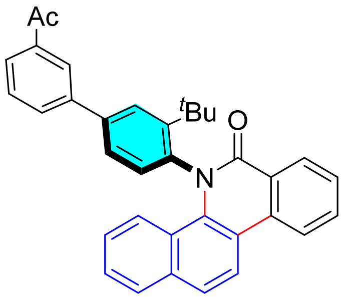

The title compound **46** was prepared according to **General Procedure C** and purified by preparative TLC in hexane/EtOAc = 10/1 as the eluent to afford a white foam (36 mg, 73% yield, 86% ee).

^{1}H NMR (600 MHz, Chloroform-*d*) δ 8.60 (d, *J* = 6.7 Hz, 1H), 8.44 (dd, *J* = 13.9, 8.6 Hz, 2H), 8.29 (s, 1H), 8.02 – 7.97 (m, 2H), 7.92 – 7.84 (m, 3H), 7.80 (d, *J* = 8.8 Hz, 1H), 7.65 (t, *J* = 7.5 Hz, 1H), 7.60 (t, *J* = 7.7 Hz, 1H), 7.54 (dd, *J* = 8.1, 2.2 Hz, 1H), 7.44 (d, *J* = 9.1 Hz, 1H), 7.39 (t, *J* = 7.4 Hz, 1H), 7.10 (d, *J* = 8.0 Hz, 1H), 7.06 (td, *J* = 6.9, 3.3 Hz, 1H), 2.70 (s, 3H), 1.28 (s, 9H).
^{13}C NMR (151 MHz, Chloroform-*d*) δ 164.3, 148.2, 141.6, 140.8, 140.4, 137.9, 135.7, 135.3, 134.9, 133.3, 132.1, 131.7, 129.6, 129.3, 129.2, 129.1, 128.2, 127.6, 127.2, 126.7, 126.3, 126.0, 125.5, 125.4, 125.1, 124.6, 122.7, 120.8, 116.8, 37.0, 31.8, 27.0.
HRMS (ESI-TOF) calcd for $C_{35}H_{29}NNaO_2^{+}$ ($[M+Na]^{+}$): 518.2091, found: 518.2090.
$[\alpha]_D^{20}$ = 4.9 (c = 0.96, $CHCl_3$).
The ee value was determined by HPLC analysis on a OD-H column (n-hexane/i-PrOH = 70/30, flow = 1.1 mL/min, 254 nm), t = 7.2 min (major), t = 10.3 min (minor). The absolute stereochemistry was assigned by analogy to compound **55**.

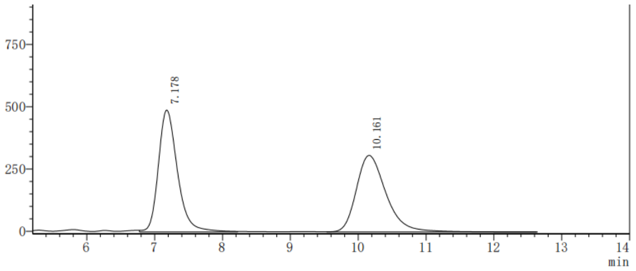

Peak	Ret. Time	Area	Height	Area%
1	7.178	9599921	489071	50.548
2	10.161	9391737	307110	49.452
Total		18991658	796180	100.000

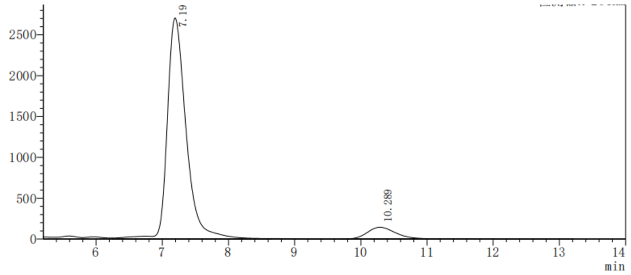

Peak	Ret. Time	Area	Height	Area%
1	7.193	53798955	2705379	92.798
2	10.289	4175515	142285	7.202
Total		57974471	2847664	100.000

Supplementary Fig. 48 | Enantiomeric excess determination of product 46

(***S***)-12-(2-(tert-butyl)phenyl)-2,3-difluorobenzofuro[2,3-*c*]phenanthridin-13(12H)-one(**47**)

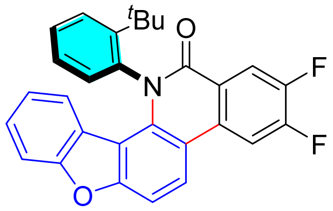

The title compound **47** was prepared according to **General Procedure C** and purified by preparative TLC in hexane/EtOAc = 10/1 as the eluent to afford a yellow foam (29 mg, 64% yield, 90% ee).

^{1}H NMR (400 MHz, Chloroform-*d*) δ 8.42 – 8.22 (m, 2H), 8.11 (dd, J = 12.0, 7.0 Hz, 1H), 7.87 (dd, J = 8.3, 1.5 Hz, 1H), 7.72 – 7.58 (m, 2H), 7.47 (dd, J = 8.1, 1.2 Hz, 1H), 7.34 (td, J = 7.5, 1.5 Hz, 1H), 7.30 – 7.25 (m, 1H), 7.17 (dd, J = 7.8, 1.5 Hz, 1H), 6.74 (ddd, J = 8.5, 7.1, 1.3 Hz, 1H), 4.93 (dd, J = 8.6, 1.1 Hz, 1H), 1.20 (s, 9H).

^{13}C NMR (101 MHz, Chloroform-*d*) δ 162.5, 158.6, 155.6, 154.2 (dd, J_{CF} = 240.9, 15.7 Hz), 150.1, 149.9 (dd, J_{CF} = 253.1, 13.6 Hz), 139.2, 137.0, 133.5 (dd, J_{CF} = 7.8, 2.6 Hz), 132.8, 130.7, 130.0, 128.1, 126.9, 125.8, 124.1, 123.0, 122.4, 121.5 (d, J_{CF} = 5.9 Hz), 117.2 (d, J_{CF} = 16.6 Hz), 113.5, 113.2, 111.2, 111.0, 108.3, 36.7, 31.8.

^{19}F NMR (376 MHz, Chloroform-*d*) δ -128.5 (d, J = 22.2 Hz), -136.8 (d, J = 21.4 Hz).

HRMS (ESI-TOF) calcd for $C_{29}H_{21}NF_2NaO_2^+$ ($[M+Na]^+$): 476.1433, found: 476.1434.

$[\alpha]_D^{20}$ = 153.2 (c = 0.693, $CHCl_3$).

The ee value was determined by HPLC analysis on a IG column (n-hexane/i-PrOH = 80/20, flow = 1.1 mL/min, 254 nm), t = 8.4 min (major), t = 7.7 min (minor). The absolute stereochemistry was assigned by analogy to compound **55**.

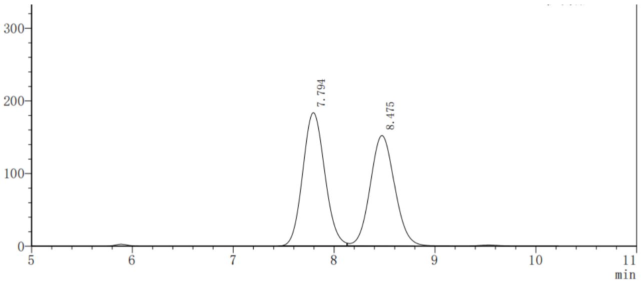

Peak	Ret. Time	Area	Height	Area%
1	7.794	2784642	183277	51.891
2	8.475	2581707	152129	48.109
Total		5366350	335407	100.000

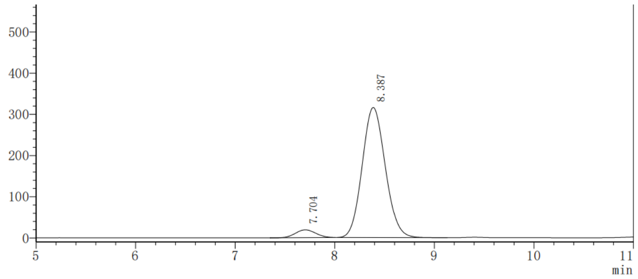

Peak	Ret. Time	Area	Height	Area%
1	7.704	271528	19089	5.051
2	8.387	5104483	315671	94.949
Total		5376011	334760	100.000

Supplementary Fig. 49 | Enantiomeric excess determination of product 47

(***S***)-12-(2-(tert-butyl)phenyl)-2,3-dimethoxybenzofuro[2,3-c]phenanthridin-13(12H)-one(**48**)

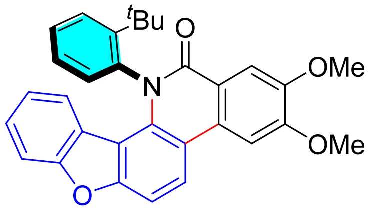

The title compound **48** was prepared according to **General Procedure C** and purified by preparative TLC in hexane/EtOAc = 4/1 as the eluent to afford a white foam (42 mg, 89% yield, 90% ee).

^{1}H NMR (400 MHz, Chloroform-*d*) δ 8.43 (d, *J* = 8.9 Hz, 1H), 7.89 (s, 1H), 7.85 (dd, *J* = 8.3, 1.4 Hz, 1H), 7.70 (s, 1H), 7.66 – 7.56 (m, 2H), 7.46 (dd, *J* = 8.2, 1.2 Hz, 1H), 7.33 (td, *J* = 7.5, 1.4 Hz, 1H), 7.25 (td, *J* = 7.6, 7.1, 1.1 Hz, 1H), 7.19 (dd, *J* = 7.8, 1.5 Hz, 1H), 6.72 (ddd, *J* = 8.5, 7.2, 1.2 Hz, 1H), 4.92 (d, *J* = 8.5 Hz, 1H), 4.15 (s, 3H), 4.03 (s, 3H), 1.19 (s, 9H).

^{13}C NMR (101 MHz, Chloroform-*d*) δ 163.5, 157.8, 155.5, 154.0, 150.2, 149.7, 139.7, 136.3, 133.0, 130.5, 130.4, 129.7, 127.9, 126.5, 125.8, 123.6, 123.4, 122.1, 118.3, 114.7, 113.0, 110.9, 108.8, 107.6, 103.3, 56.3, 36.7, 31.8.

HRMS (ESI-TOF) calcd for $C_{31}H_{27}NNaO_4^+$ ([M+Na]$^+$): 500.1832, found:500.1830.

$[\alpha]_D^{20}$ = 157.8 (c = 0.875, $CHCl_3$).

The ee value was determined by HPLC analysis on a IG column (n-hexane/i-PrOH = 80/20, flow = 1.1 mL/min, 254 nm), t = 10.1 min (major), t = 13.8 min (minor). The absolute stereochemistry was assigned by analogy to compound **55**.

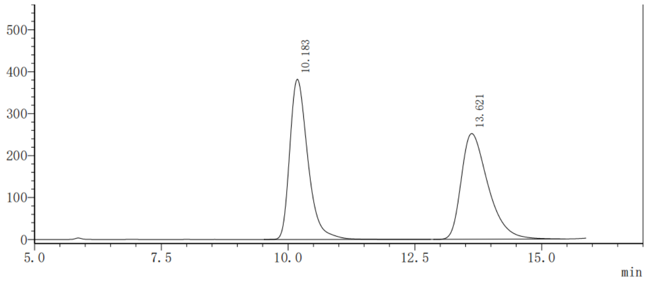

Peak	Ret. Time	Area	Height	Area%
1	10.183	9501813	381942	50.578
2	13.621	9284620	252207	49.422
Total		18786433	634149	100.000

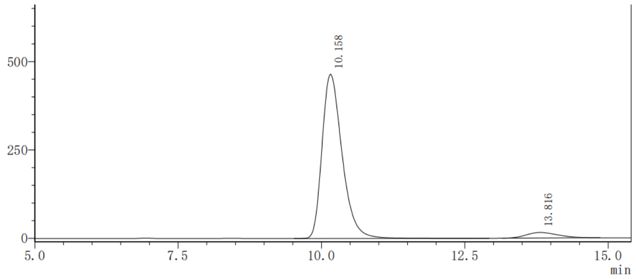

Peak	Ret. Time	Area	Height	Area%
1	10.158	11285491	464135	94.814
2	13.816	617319	15809	5.186
Total		11902811	479944	100.000

Supplementary Fig. 50 | Enantiomeric excess determination of product 48

(***S***)-5-(2-(1-((tert-butyldimethylsilyl)oxy)-2-methylpropan-2-yl)phenyl)benzo[*c*]phenanthridin-6(5H)-one(**49**)

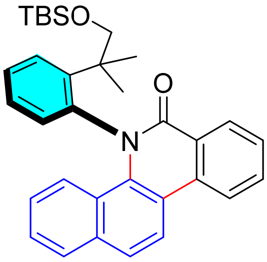

The title compound **49** was prepared according to **General Procedure C** and purified by preparative TLC in hexane/EtOAc = 10/1 as the eluent to afford a yellow solid (42 mg, 83% yield, 92% ee). mp: 128.3-129.4°C.

^{1}H NMR (600 MHz, Chloroform-*d*) δ 8.58 (dd, *J* = 7.9, 1.4 Hz, 1H), 8.42 (dd, *J* = 16.8, 8.5 Hz, 2H), 7.86 (t, *J* = 7.7 Hz, 1H), 7.83 – 7.75 (m, 3H), 7.64 (t, *J* = 7.5 Hz, 1H), 7.52 (t, *J* = 7.8 Hz, 1H), 7.39 – 7.31 (m, 2H), 7.17 (d, *J* = 9.1 Hz, 1H), 7.07 (d, *J* = 7.8 Hz, 1H), 7.00 (ddd, *J* = 8.8, 6.7, 1.5 Hz, 1H), 3.60 – 3.30 (dd, *J* = 24.0, 12.0 Hz, 2H), 1.14 (d, *J* = 11.7 Hz, 6H), 0.77 (s, 9H), -0.21 (d, *J* = 20.7 Hz, 6H).

^{13}C NMR (151 MHz, Chloroform-*d*) δ 164.3, 144.8, 141.1, 135.7, 135.6, 133.2, 131.6, 131.4, 129.2, 129.0, 128.7, 128.1, 128.1, 126.3, 126.2, 125.7, 125.4, 125.0, 122.7, 120.8, 71.5, 42.1, 26.0, 25.6, 25.5, 18.4, -5.6.

HRMS (ESI-TOF) calcd for $C_{33}H_{37}NNaO_2Si^+$ ([M+Na]$^+$): 530.2486, found: 530.2487.

$[\alpha]_D^{20}$ = 70.2 (c = 0.88, $CHCl_3$).

The ee value was determined by HPLC analysis on a IG column (n-hexane/i-PrOH = 80/20, flow = 1.1 mL/min, 254 nm), t = 6.0 min (major), t = 4.9 min (minor). The absolute stereochemistry was assigned by analogy to compound **55**.

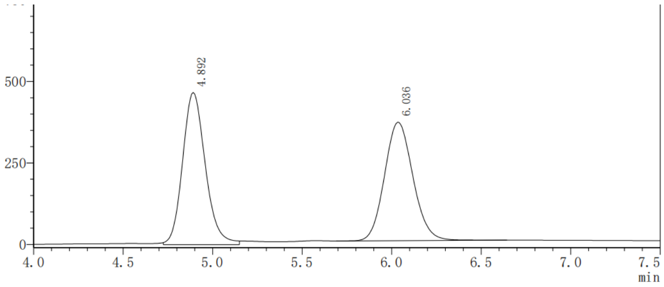

Peak	Ret. Time	Area	Height	Area%
1	4.892	4132619	467006	50.721
2	6.036	4015117	363440	49.279
Total		8147736	830446	100.000

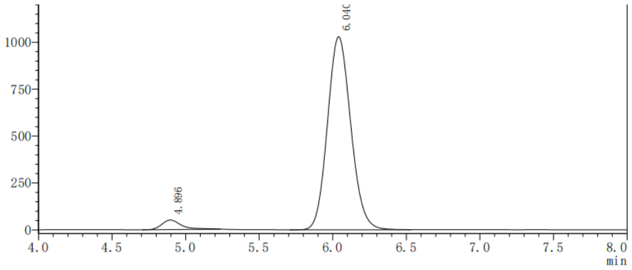

Peak	Ret. Time	Area	Height	Area%
1	4.896	469841	50489	3.965
2	6.040	11378671	1030302	96.035
Total		11848511	1080790	100.000

Supplementary Fig. 51 | Enantiomeric excess determination of product 49

(***S***)-5-(2-(2-((tert-butyldimethylsilyl)oxy)propan-2-yl)phenyl)benzo[*c*]phenanthridin-6(5H)-one(**50**)

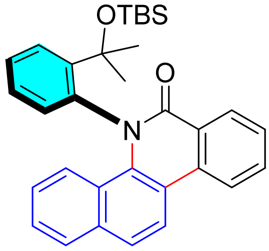

The title compound **50** was prepared according to **General Procedure C** and purified by preparative TLC in hexane/EtOAc = 10/1 as the eluent to afford a yellow oil (43 mg, 87% yield, 95% ee).
^{1}H NMR (600 MHz, Chloroform-*d*) δ 8.58 (dd, *J* = 7.9, 1.4 Hz, 1H), 8.43 (d, *J* = 8.3 Hz, 1H), 8.39 (d, *J* = 8.8 Hz, 1H), 8.06 (dd, *J* = 8.2, 1.5 Hz, 1H), 7.86 (ddd, *J* = 8.3, 7.1, 1.5 Hz, 1H), 7.81 (dd, *J* = 8.0, 1.4 Hz, 1H), 7.77 (d, *J* = 8.8 Hz, 1H), 7.64 (t, *J* = 7.5 Hz, 1H), 7.53 (t, *J* = 8.0 Hz, 1H), 7.36 (t, *J* = 7.4 Hz, 1H), 7.33 – 7.27 (m, 2H), 6.99 (ddd, *J* = 16.3, 8.3, 1.5 Hz, 2H), 1.53 (s, 3H), 1.24 (s, 3H), 0.87 (s, 9H), 0.05 (s, 3H), -0.06 (s, 3H).
^{13}C NMR (151 MHz, Chloroform-*d*) δ 163.9, 147.6, 138.6, 135.7, 135.4, 134.9, 133.2, 130.9, 130.4, 129.2, 128.9, 128.8, 128.6, 128.2, 126.3, 126.3, 125.7, 125.6, 125.1, 124.7, 122.7, 120.6, 116.8, 76.5, 32.2, 30.3, 26.1, 18.4, -1.9, -2.0.
HRMS (ESI-TOF) calcd for $C_{32}H_{35}NNaO_2Si^+$ ([M+Na]$^+$): 516.2329, found: 516.233.
$[\alpha]_D^{20}$ = 44.1 (c = 0.96, $CHCl_3$).
The ee value was determined by HPLC analysis on a IG column (n-hexane/i-PrOH = 80/20, flow = 1.1 mL/min, 254 nm), t = 7.1 min (major), t = 4.7 min (minor). The absolute stereochemistry was assigned by analogy to compound **55**.

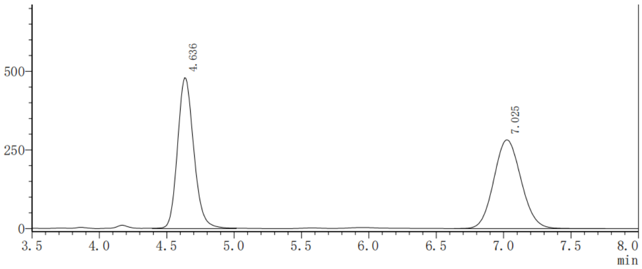

Peak	Ret. Time	Area	Height	Area%
1	4.636	4035182	480217	50.246
2	7.025	3995694	282579	49.754
Total		8030876	762796	100.000

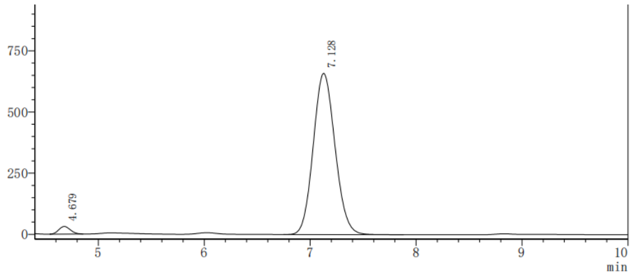

Peak	Ret. Time	Area	Height	Area%
1	4.679	236199	30975	2.442
2	7.128	9436525	658862	97.558
Total		9672724	689837	100.000

Supplementary Fig. 52 | Enantiomeric excess determination of product 50

(***S***)-5-(2-(1-hydroxy-2-methylpropan-2-yl)phenyl)benzo[*c*]phenanthridin-6(5H)-one(**51**)

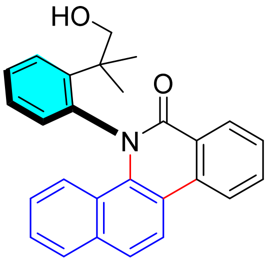

The title compound **51** was prepared according to **General Procedure C** and purified by preparative TLC in hexane/EtOAc = 3/1 as the eluent to afford a white foam (42 mg, 82% yield, 79% ee).

^{1}H NMR (600 MHz, Chloroform-*d*) δ 8.61 (d, *J* = 8.0 Hz, 1H), 8.46 (dd, *J* = 15.1, 8.4 Hz, 2H), 7.90 (t, *J* = 7.8 Hz, 1H), 7.86 (d, *J* = 8.1 Hz, 1H), 7.82 (dd, *J* = 8.9, 2.8 Hz, 2H), 7.66 (t, *J* = 7.6 Hz, 1H), 7.57 (t, *J* = 8.1 Hz, 1H), 7.40 (t, *J* = 7.5 Hz, 1H), 7.35 (t, *J* = 7.7 Hz, 1H), 7.25 (d, *J* = 9.6 Hz, 1H), 7.10 – 7.02 (m, 1H), 6.94 (d, *J* = 7.8 Hz, 1H), 3.67 – 3.57 (m, 2H), 2.56 (br, 1H), 1.39 (s, 3H), 1.08 (s, 3H).

^{13}C NMR (151 MHz, Chloroform-*d*) δ 165.4, 143.8, 142.2, 135.7, 134.9, 134.8, 133.7, 131.3, 130.8, 129.4, 129.3, 129.0, 128.5, 126.4, 126.0, 125.6, 125.5, 124.9, 124.5, 122.7, 120.8, 117.2, 74.3, 42.6, 29.3, 24.5.

HRMS (ESI-TOF) calcd for $C_{27}H_{23}NNaO_2^+$ ([M+Na]$^+$): 416.1621, found: 416.1623.

$[\alpha]_D^{20}$ = 43.7 (c = 0.91, $CHCl_3$).

The ee value was determined by HPLC analysis on a AD-H column (n-hexane/i-PrOH = 75/25, flow = 1.1 mL/min, 254 nm), t = 9.9 min (major), t = 9.2 min (minor). The absolute stereochemistry was assigned by analogy to compound **55**.

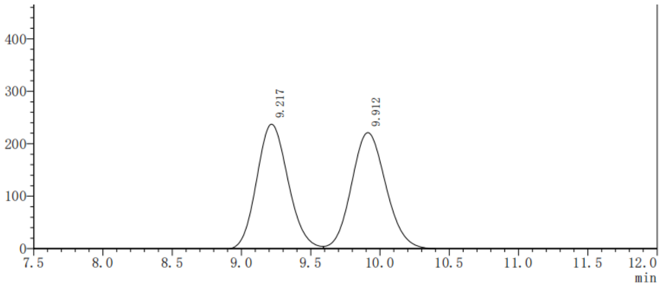

Peak	Ret. Time	Area	Height	Area%

1	9.217	3811367	239193	49.394
2	9.912	3904907	222872	50.606
Total		7716275	462065	100.000

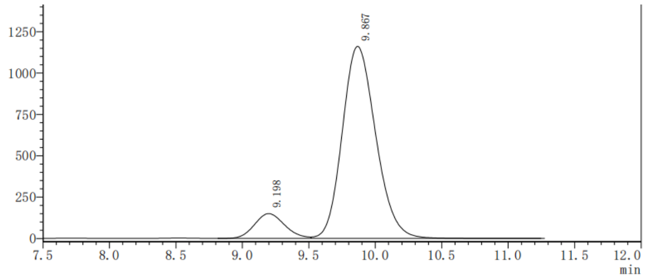

Peak	Ret. Time	Area	Height	Area%
1	9.198	2375833	150072	10.440
2	9.867	20382069	1161686	89.560
Total		22757902	1311758	100.000

Supplementary Fig. 53 | Enantiomeric excess determination of product 51

General Procedure for the Synthesis of diaxially chirality amide products

General Procedure E: To an oven-dried 10 mL Schlenk tube were added 1,5-diiodonaphthalene (0.05 mmol), amide **2** (0.1 mmol), $Pd_2(dba)_3$ (2.5 mg, 0.0025 mmol), **L25** (5.1 mg, 0.01 mmol), Ag_2SO_4 (31.2 mg, 0.1 mmol), Toluene (1.0 mL), H_2O (0.1 mL), 4ÅMS (50 mg), NBE (7.5 mg, 0.075 mmol). The mixture was stirred for 36 h at 80 °C. The resulting mixture was quenched by filtered through a celite pad and concentrated in *vacuo*. The residue was purified by preparative TLC to afford the product.

(***S***, ***S***)-7,15-bis(2-(tert-butyl)phenyl)-7,15-dihydrophenanthridino[4,3-*c*]phenanthridine-8,16 dione(**52**)

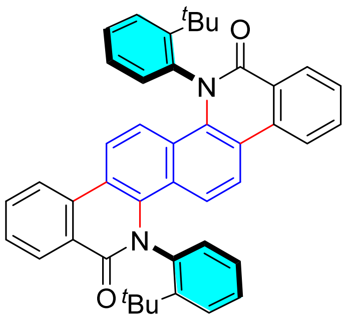

The title compound **52** was prepared according to **General Procedure E** and purified by preparative TLC in hexane/EtOAc = 5/1 as the eluent to afford a yellow foam (17 mg, 53% yield, 91.5:8.5 dr, 99% ee).

^{1}H NMR (400 MHz, Chloroform-*d*) δ 8.54 (dd, *J* = 7.9, 1.4 Hz, 2H), 8.27 (d, *J* = 8.4 Hz, 2H), 7.88 (d, *J* = 9.6 Hz, 2H), 7.79 (td, *J* = 7.7, 7.2, 1.5 Hz, 4H), 7.69 (d, *J* = 9.6 Hz, 2H), 7.62 (t, *J* = 7.5 Hz, 2H), 7.48 (td, *J* = 7.7, 1.5 Hz, 2H), 7.22 (td, *J* = 7.5, 1.5 Hz, 2H), 6.82 (dd, *J* = 7.8, 1.5 Hz, 2H), 1.39 (s, 18H).

^{13}C NMR (101 MHz, Chloroform-*d*) δ 164.3, 147.7, 140.7, 135.5, 133.8, 133.2, 130.7, 130.1, 129.2, 128.7, 128.6, 127.8, 126.3, 125.8, 122.6, 122.0, 118.7, 116.7, 37.1, 31.9.
HRMS (ESI-TOF) calcd for $C_{44}H_{38}N_2NaO_2^+$ ($[M+Na]^+$): 649.2825, found: 649.2829.
$[\alpha]_D^{20}$ = 88.0 (c = 0.89, $CHCl_3$).
The ee and dr value was determined by HPLC analysis on a AD-H column (n-hexane/i-PrOH = 90/10, flow = 0.8 mL/min, 254 nm), t = 11.4 min (major), t = 14.2 min (*meso*), t = 17.9 min (minor). The absolute stereochemistry was assigned by analogy to compound 55.

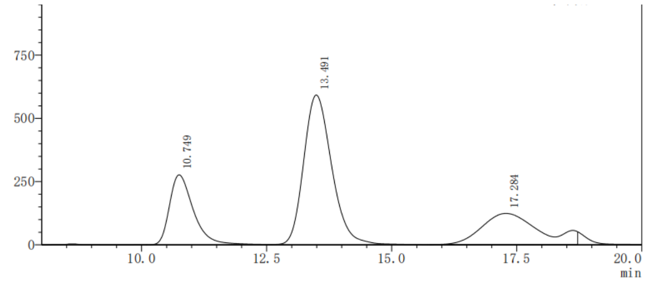

Peak	Ret. Time	Area	Height	Area%
1	10.749	9698498	279931	22.472
2	13.491	23595609	594837	54.672
3	17.284	9864618	123079	22.857
Total		43158725	997847	100.000

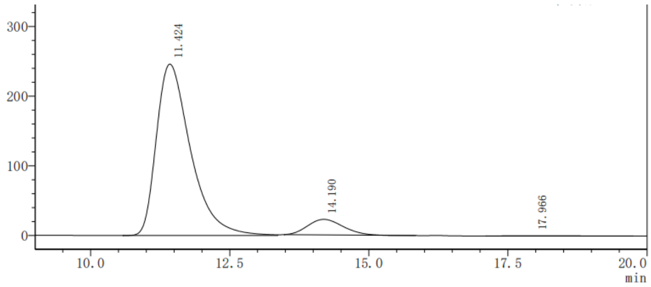

Peak	Ret. Time	Area	Height	Area%
1	11.424	10534885	246199	91.318
2	14.190	989570	22338	8.578
3	17.966	11973	169	0.104
Total		11536428	268707	100.000

Supplementary Fig. 54 | Enantiomeric excess determination of product 52

(***S***, ***S***)-7,15-bis(2-(2-((tert-butyldimethylsilyl)oxy)propan-2-yl)phenyl)-7,15-dihydrophenanthridino[4,3-*c*]phenanthridine-8,16-dione(**53**)

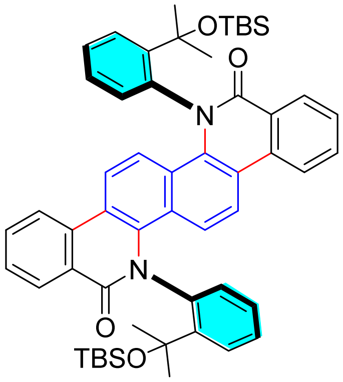

The title compound **53** was prepared according to **General Procedure E** and purified by preparative TLC in hexane/EtOAc = 5/1 as the eluent to afford a yellow foam (21 mg, 49% yield, 94% ee).

^{1}H NMR (400 MHz, Chloroform-*d*) δ 8.52 (dd, J = 8.0, 1.4 Hz, 2H), 8.18 (d, J = 8.2 Hz, 2H), 8.00 (dd, J = 8.2, 1.5 Hz, 2H), 7.85 – 7.73 (m, 6H), 7.61 (t, J = 7.5 Hz, 2H), 7.52 – 7.42 (m, 2H), 7.20 (td, J = 7.6, 1.5 Hz, 2H), 6.68 (dd, J = 7.9, 1.3 Hz, 2H), 1.76 (s, 6H), 1.61 (s, 6H), 0.89 (s, 18H), 0.19 (s, 6H), 0.04 (s, 6H).

^{13}C NMR (101 MHz, Chloroform-*d*) δ 163.8, 147.7, 139.1, 135.3, 133.9, 133.2, 130.8, 129.1, 128.9, 128.6, 128.5, 126.4, 125.8, 122.5, 122.5, 118.7, 116.8, 33.1, 29.8, 26.0, 18.4, -1.7, -1.8.

HRMS (ESI-TOF) calcd for $C_{54}H_{62}N_2NaO_4Si_2^+$ ($[M+Na]^+$): 881.4140, found: 881.4145.

$[\alpha]_D^{20}$ = 17.4 (c = 0.95, $CHCl_3$).

The ee value was determined by HPLC analysis on a IE column (n-hexane/i-PrOH = 80/20, flow = 1.1 mL/min, 254 nm), t = 11.9 min (major), t = 14.0 min (minor). The absolute stereochemistry was assigned by analogy to compound **55**.

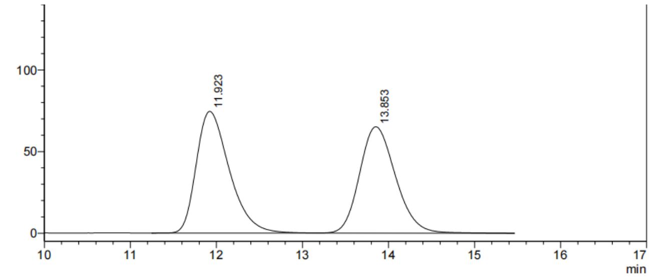

Peak	Ret. Time	Area	Height	Area%
1	11.923	1999367	74547	51.288
2	13.853	1898950	65166	48.712
Total		3898317	139713	100.000

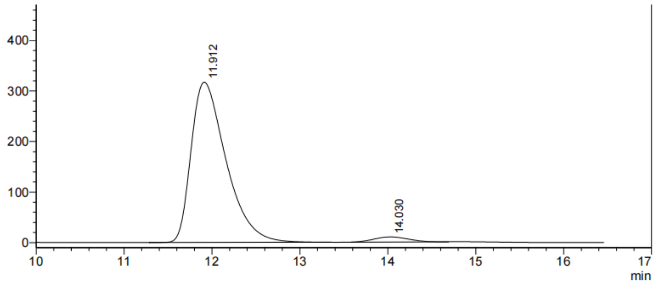

Peak	Ret. Time	Area	Height	Area%
1	11.912	8767657	316971	97.044

2	14.030	267035	9958	2.956
Total		9034691	326928	100.000

Supplementary Fig. 55 | Enantiomeric excess determination of product 53

(***meso***)-7,15-bis(2-(2-((tert-butyldimethylsilyl)oxy)propan-2-yl)phenyl)-7,15-dihydrophenanthridino[4,3-*c*]phenanthridine-8,16-dione(**53'**)

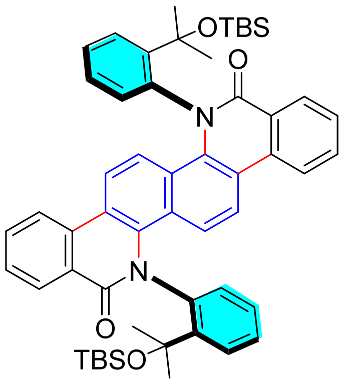

The title compound **53'** was prepared according to **General Procedure E** and purified by preparative TLC in hexane/EtOAc = 5/1 as the eluent to afford a yellow foam (4.6 mg, 11% yield).
^{1}H NMR (400 MHz, Chloroform-*d*) δ 8.54 (d, *J* = 7.9 Hz, 2H), 8.19 (d, *J* = 8.2 Hz, 2H), 7.93 (dd, *J* = 8.1, 1.5 Hz, 2H), 7.83 – 7.77 (m, 4H), 7.68 (d, *J* = 9.4 Hz, 2H), 7.62 (t, *J* = 7.5 Hz, 2H), 7.47 (t, *J* = 7.7 Hz, 2H), 7.32 – 7.27 (t, *J* = 7.7 Hz, 2H), 6.99 (d, *J* = 8.4 Hz, 2H), 1.39 (s, 6H), 1.18 (s, 6H), 0.82 (s, 9H), 0.03 (s, 6H), -0.05 (s, 6H).
^{13}C NMR (101 MHz, Chloroform-*d*) δ 163.6, 147.1, 138.4, 135.4, 133.7, 133.2, 130.9, 129.3, 128.6, 128.5, 127.9, 126.6, 126.0, 122.5, 122.2, 118.6, 116.7, 76.5, 31.4, 30.5, 26.0, 18.3, -1.8, -2.0.
HRMS (ESI-TOF) calcd for $C_{54}H_{62}N_2NaO_4Si_2^+$ ([M+Na]$^+$): 881.4140, found: 881.4139.

(***S***, ***S***)-7,15-bis(2-(1-((tert-butyldimethylsilyl)oxy)-2-methylpropan-2-yl)phenyl)-7,15-dihydrophenanthridino[4,3-*c*]phenanthridine-8,16-dione(**54**)

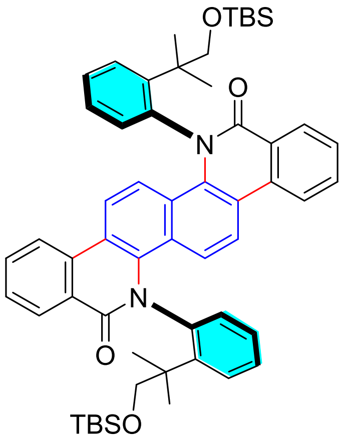

The title compound **54** was prepared according to **General Procedure E** and purified by preparative TLC in hexane/EtOAc = 6/1 as the eluent to afford a yellow foam (22 mg, 50% yield, 99% ee).
^{1}H NMR (400 MHz, Chloroform-*d*) δ 8.53 (dd, *J* = 8.0, 1.4 Hz, 2H), 8.24 (d, *J* = 8.3 Hz, 2H), 7.90 – 7.77 (m, 6H), 7.69 (d, *J* = 9.5 Hz, 2H), 7.65 – 7.58 (m, 2H), 7.47 (ddd, *J* = 8.4, 7.3, 1.5 Hz, 2H),

7.21 (td, J = 7.6, 1.4 Hz, 2H), 6.76 (dd, J = 7.8, 1.5 Hz, 2H), 3.90 – 3.73(dd, J = 30.0, 10.0 Hz, 4H), 1.41 (s, 6H), 1.33 (s, 6H), 0.84 (s, 18H), -0.04 (s, 6H), -0.08 (s, 6H).

^{13}C NMR (101 MHz, Chloroform-*d*) δ 164.3, 145.0, 141.7, 135.4, 133.9, 133.2, 131.2, 130.0, 129.1, 128.5, 128.4, 128.2, 126.2, 125.8, 122.6, 122.3, 118.7, 116.6, 71.8, 42.4, 26.5, 26.1, 25.0, 18.5, -5.3, -5.4.

HRMS (ESI-TOF) calcd for $C_{56}H_{66}N_2NaO_4Si_2^+$ ([M+Na]$^+$): 909.4453, found: 909.4453.

$[\alpha]_D^{20}$ = 26.4 (c = 1.0, $CHCl_3$).

The ee value was determined by HPLC analysis on a IA column (n-hexane/i-PrOH = 80/20, flow = 1.0 mL/min, 254 nm), t = 4.3 min (major), t = 5.3 min (minor). The absolute stereochemistry was assigned by analogy to compound **55**.

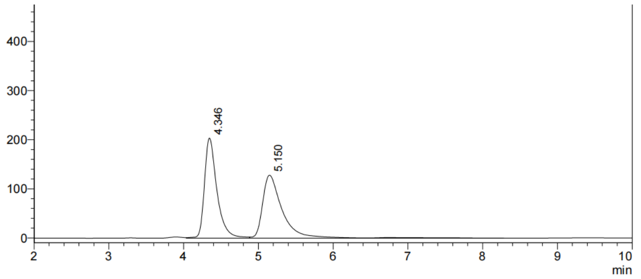

Peak	Ret. Time	Area	Height	Area%
1	4.346	2370522	203793	49.836
2	5.150	2386119	128329	50.164
Total		4756641	332122	100.000

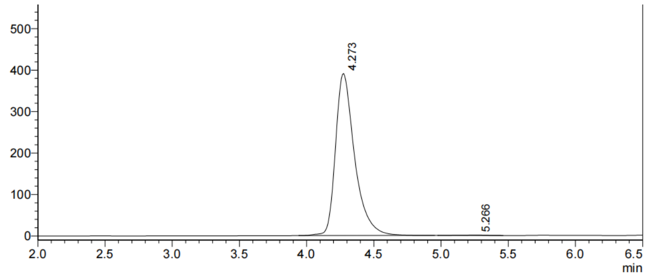

Peak	Ret. Time	Area	Height	Area%
1	4.273	3871437	390606	99.646
2	5.266	13735	974	0.354
Total		3885173	391580	100.000

Supplementary Fig. 56 | Enantiomeric excess determination of product 54

(***meso***)-7,15-bis(2-(1-((tert-butyldimethylsilyl)oxy)-2-methylpropan-2-yl)phenyl)-7,15-dihydrophenanthridino[4,3-*c*]phenanthridine-8,16-dione(**54'**)

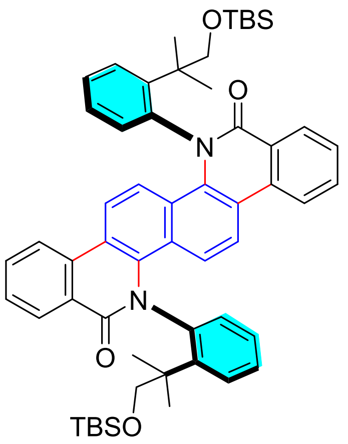

The title compound **54'** was prepared according to **General Procedure E** and purified by preparative TLC in hexane/EtOAc = 6/1 as the eluent to afford a yellow foam (16 mg, 37% yield).

^{1}**H NMR (400 MHz, Chloroform-*d*)** δ 8.54 (dd, *J* = 8.0, 1.4 Hz, 2H), 8.23 (d, *J* = 8.3 Hz, 2H), 7.89 – 7.73 (m, 6H), 7.62 (t, *J* = 7.5 Hz, 2H), 7.53 (d, *J* = 9.5 Hz, 2H), 7.48 (t, *J* = 7.7 Hz, 2H), 7.31 (t, *J* = 7.5 Hz, 2H), 7.04 (dd, *J* = 7.8, 1.5 Hz, 2H), 3.64 – 3.39 (dd, *J* = 36.0, 12.0 Hz, 4H), 1.08 (d, *J* = 10.5 Hz, 12H), 0.76 (s, 18H), -0.19 (d, *J* = 16.8 Hz, 12H).

^{13}**C NMR (101 MHz, Chloroform-*d*)** δ 164.1, 144.5, 140.9, 135.6, 133.8, 133.2, 131.6, 131.4, 129.2, 128.6, 128.5, 127.7, 126.6, 125.9, 122.6, 122.0, 118.7, 116.5, 71.4, 42.0, 26.0, 25.8, 25.1, 18.4, -5.5.

HRMS (ESI-TOF) calcd for $C_{56}H_{66}N_2NaO_4Si_2^+$ ($[M+Na]^+$): 909.4453, found: 909.4456.

2.4 Scale-up Preparation, Derivatization, and Application.

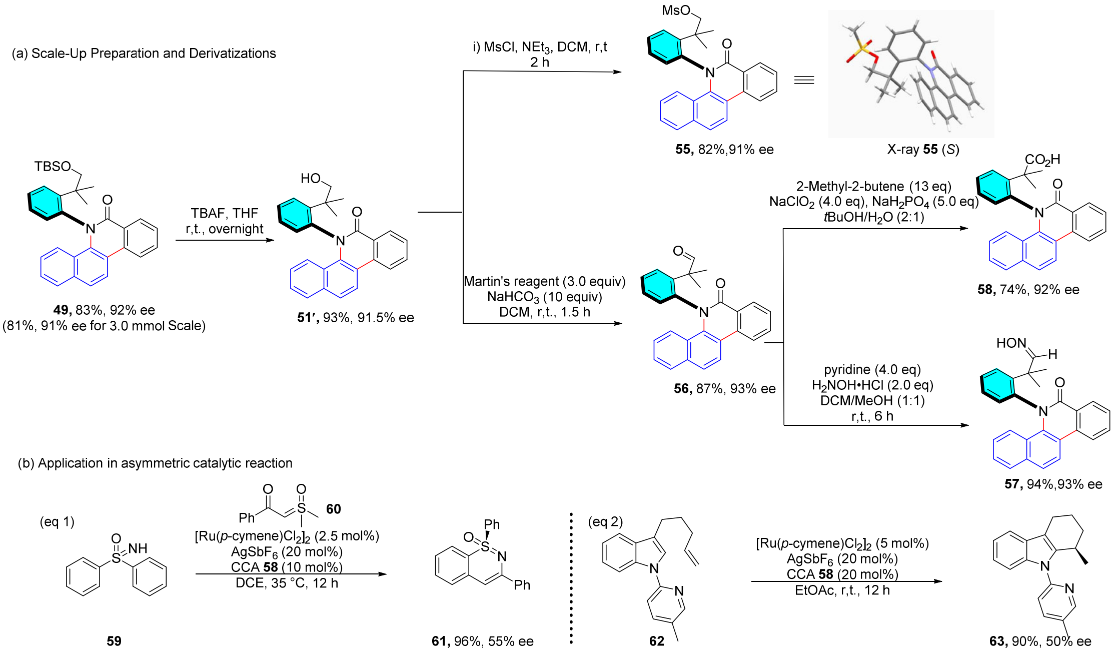

Supplementary Fig. 57 | Scale-Up Preparation and Derivatizations, and Application

Scale-up preparation of 49

To an oven-dried 250 mL Schlenk tube were added substrate **1** (4.5 mmol), amide **2m** (3.0 mmol), $Pd_2(dba)_3$ (147 mg, 0.15 mmol), **L26** (306 mg, 0.6 mmol), Ag_2SO_4 (1.869 g, 6 mmol), Toluene (60 mL), H_2O (6.0 mL), 4ÅMS (3.0 g), NBE (450 mg, 4.5 mmol). The mixture was stirred for 36 h at 80 °C. The resulting mixture was quenched by filtered through a celite pad and concentrated in *vacuo*. The residue was purified by column chromatography to afford the product **49** (1.23 g, 81% yield, 91% ee). The ee value was determined by chiral HPLC.

Derivatizations of 49

Synthesis of 51 (by desilylation of compound 49)

TBAF (5.0 mmol, 1 M in THF, 5.0 equiv) was added dropwise to a solution of **49** (507 mg, 1.0 mmol, 1.0 equiv) in THF (10 mL) at 0 ºC, then the mixture was warmed to r.t. and stirred for 8 h. Quenched with H_2O (10 mL), extracted with EtOAc, the combined organic layers were washed with brine, dried over Na_2SO_4, filtered and concentrated *in vacuo*. The residue was purified by column chromatography on silica gel gave the desired product **51** as a yellow foam (365 mg, 93% yield, 90% ee).

$[\alpha]_D^{20}$ = 65.7 (c = 0.77, $CHCl_3$).

The ee value was determined by HPLC analysis on a AD-H column (n-hexane/i-PrOH = 75/25, flow = 1.1 mL/min, 254 nm), t = 9.9 min (major), t = 9.2 min (minor). The absolute stereochemistry was assigned by analogy to compound **55**.

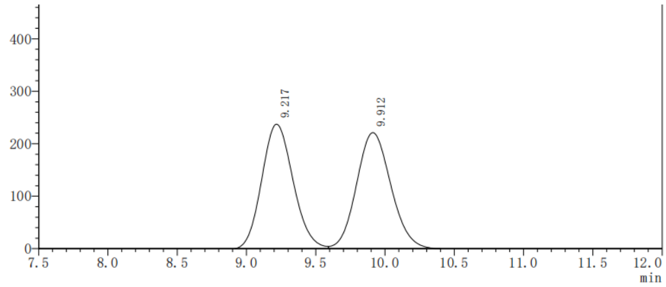

Peak	Ret. Time	Area	Height	Area%
1	9.217	3811367	239193	49.394
2	9.912	3904907	222872	50.606
Total		7716275	462065	100.000

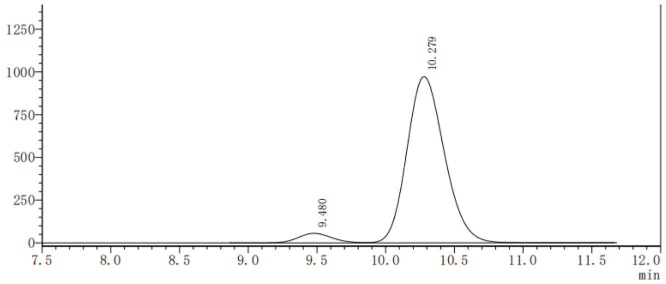

Peak	Ret. Time	Area	Height	Area%
1	9.480	954305	56086	4.918
2	10.279	18451256	972520	95.082

Total		19405561	1028606	100.000

Supplementary Fig. 58 | Enantiomeric excess determination of product 51 (by desilylation of compound 49)

Synthesis of 55

To an oven-dried 10 mL Schlenk tube were added **51'** (39 mg, 0.1 mmol), Et_3N (0.3 mmol), MsCl (0. 5 mmol), DCM (3 mL). The mixture was stirred for 2 h at 20 °C. The resulting mixture was quenched by filtered through a celite pad and concentrated in *vacuo*. The residue was purified by preparative TLC to afford the product **55** as a colorless solid (38 mg, 82% yield, 91% ee).

(***S***)-2-methyl-2-(2-(6-oxobenzo[*c*]phenanthridin-5(6H)-yl)phenyl)propyl methanesulfonate(**55**)

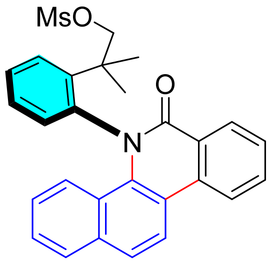

^{1}H NMR (600 MHz, Chloroform-*d*) δ 8.57 (d, *J* = 7.8 Hz, 1H), 8.48 – 8.39 (m, 2H), 7.90 (t, *J* = 8.0 Hz, 1H), 7.85 (d, *J* = 8.1 Hz, 1H), 7.81 (d, *J* = 8.5 Hz, 2H), 7.67 (t, *J* = 8.2 Hz, 1H), 7.56 (t, *J* = 8.3 Hz, 1H), 7.40 (t, *J* = 7.9 Hz, 1H), 7.34 (t, *J* = 8.3 Hz, 1H), 7.20 (d, *J* = 9.2 Hz, 1H), 7.07 (t, *J* = 8.0 Hz, 1H), 6.98 (d, *J* = 7.7 Hz, 1H), 4.43 (dd, *J* = 9.4, 2.9 Hz, 1H), 4.14 (dd, *J* = 9.4, 3.0 Hz, 1H), 2.74 (s, 3H), 1.40 (s, 3H), 1.25 (s, 3H).

^{13}C NMR (151 MHz, Chloroform-*d*) δ 164.4, 142.1, 141.6, 135.7, 135.0, 134.9, 133.5, 131.4, 131.1, 129.2, 129.2, 129.1, 129.1, 128.4, 126.4, 125.8, 125.6, 125.4, 125.2, 124.4, 122.8, 120.8, 117.0, 77.8, 40.4, 36.8, 26.9, 25.0.

HRMS (ESI-TOF) calcd for $C_{28}H_{25}NNaO_4S^+$ ($[M+Na]^+$): 494.1397, found: 494.1399.

$[\alpha]_D^{20}$ = 42.6 (c = 0.86, $CHCl_3$).

The ee value was determined by HPLC analysis on a IG column (n-hexane/i-PrOH = 50/50, flow = 1.2 mL/min, 254 nm), t = 19.2 min (major), t = 10.6 min (minor). The absolute stereochemistry was assigned by X-ray.

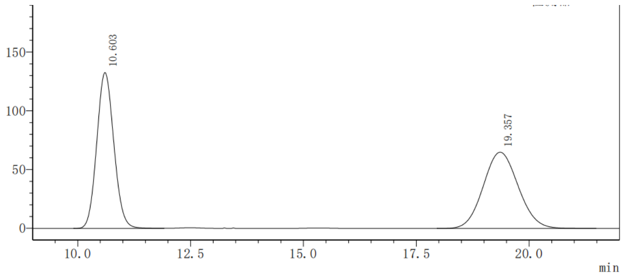

Peak	Ret. Time	Area	Height	Area%
1	10.603	3502359	132666	49.957
2	19.357	3508437	64907	50.043
Total		7010796	197573	100.000

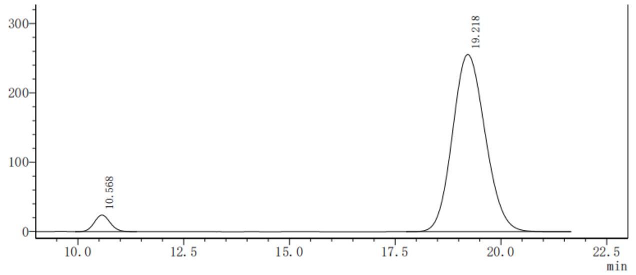

Peak	Ret. Time	Area	Height	Area%
1	10.568	619184	23825	4.301
2	19.218	13776688	255628	95.699
Total		14395872	279453	100.000

Supplementary Fig. 59 | Enantiomeric excess determination of product 55

Synthesis of 56

To an oven-dried 10 mL Schlenk tube were added **51'** (39 mg, 0.1 mmol), Martin's reagent (0.3 mmol), $NaHCO_3$ (1.0 mmol), DCM (5 mL) at 0 °C. Then the mixture was stirred for 1 h at 25 °C. The resulting mixture was quenched by filtered through a celite pad and concentrated in *vacuo*. The residue was purified by preparative TLC to afford the product **56** as a yellow foam (34 mg, 87% yield, 93% ee). The ee value was determined by chiral HPLC.

(***S***)-2-methyl-2-(2-(6-oxobenzo[*c*]phenanthridin-5(6H)-yl)phenyl)propanal(**56**)

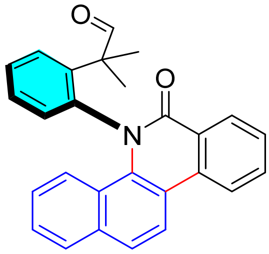

^{1}H NMR (600 MHz, Chloroform-*d*) δ 9.42 (s, 1H), 8.54 (d, *J* = 7.9 Hz, 1H), 8.42 (t, *J* = 9.1 Hz, 2H), 7.90 – 7.82 (m, 2H), 7.79 (d, *J* = 8.7 Hz, 1H), 7.67 – 7.58 (m, 3H), 7.46 – 7.40 (m, 1H), 7.37 (t, *J* = 7.4 Hz, 1H), 7.17 (d, *J* = 7.9 Hz, 1H), 7.12 (d, *J* = 9.2 Hz, 1H), 7.02 (t, *J* = 7.5 Hz, 1H), 1.37 (s, 3H), 1.17 (s, 3H).
^{13}C NMR (151 MHz, Chloroform-*d*) δ 201.2, 164.4, 141.7, 141.0, 135.6, 134.8, 134.7, 133.5, 131.4, 130.0, 129.6, 129.3, 129.2, 129.1, 128.4, 126.2, 125.5, 125.4, 125.3, 124.3, 122.8, 120.9, 117.3, 51.0, 23.6, 23.1.
HRMS (ESI-TOF) calcd for $C_{27}H_{21}NNaO_2^+$ ($[M+Na]^+$): 414.1465, found: 414.1467.
$[\alpha]_D^{20}$ = 124.2 (c = 0.83, $CHCl_3$).
The ee value was determined by HPLC analysis on a IG column (n-hexane/i-PrOH = 80/20, flow = 1.2 mL/min, 254 nm), t = 24.1 min (major), t = 21.9 min (minor). The absolute stereochemistry was assigned by analogy to compound **55**.

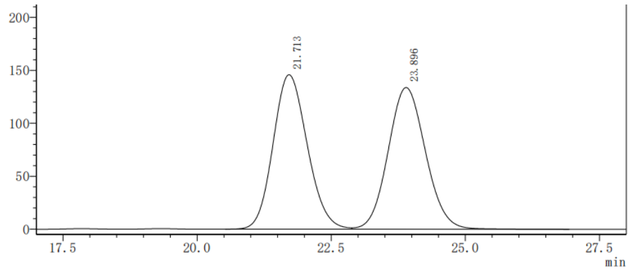

Peak	Ret. Time	Area	Height	Area%
1	21.713	6468737	145854	49.903
2	23.896	6493919	133753	50.097
Total		12962656	279607	100.000

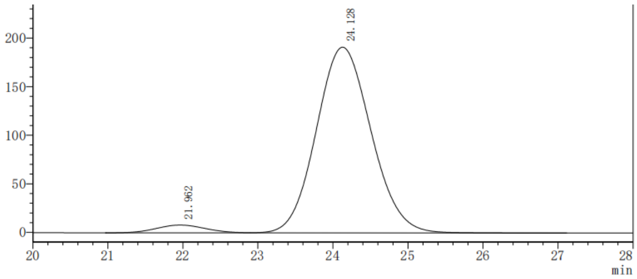

Peak	Ret. Time	Area	Height	Area%
1	21.962	368047	8055	3.649
2	24.128	9717614	191181	96.351
Total		10085661	199236	100.000

Supplementary Fig. 60 | Enantiomeric excess determination of product 56

Synthesis of 57

To an oven-dried 10 mL Schlenk tube were added **56** (39 mg, 0.1 mmol), pyridine (0.4 mmol), hydroxylamine hydrochloride (0.2 mmol), DCM/MeOH (1:1, 2.0 mL) at 25 °C. Then the mixture was stirred for 5 h. The resulting mixture was quenched by filtered through a celite pad and concentrated in *vacuo*. The residue was purified by preparative TLC to afford the product **57** as a white foam (38 mg, 94% yield, 93% ee).

(***S***, ***Z***)-2-methyl-2-(2-(6-oxobenzo[*c*]phenanthridin-5(6H)-yl)phenyl)propanal oxime(**57**)

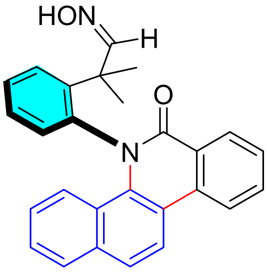

^{1}H NMR (600 MHz, Chloroform-*d*) δ 8.57 (d, *J* = 7.9 Hz, 1H), 8.40 (dd, *J* = 8.6, 6.0 Hz, 2H), 7.83 (d, *J* = 7.9 Hz, 2H), 7.77 (d, *J* = 8.7 Hz, 1H), 7.68 (d, *J* = 8.2 Hz, 1H), 7.61 (t, *J* = 7.5 Hz, 1H), 7.57

(t, J = 7.8 Hz, 1H), 7.37 (q, J = 7.1 Hz, 2H), 7.33 (s, 1H), 7.09 (dd, J = 8.7, 3.3 Hz, 2H), 7.00 (t, J = 8.0 Hz, 1H), 6.48 (s, 1H), 1.43 (s, 3H), 1.25 (s, 3H).

^{13}C NMR (151 MHz, Chloroform-*d*) δ 164.2, 157.1, 143.7, 140.7, 135.6, 135.0, 134.9, 133.2, 131.3, 130.0, 129.4, 129.2, 129.2, 128.8, 128.1, 126.2, 125.6, 125.4, 125.1, 124.5, 122.6, 120.9, 117.1, 41.8, 27.2, 27.2.

HRMS (ESI-TOF) calcd for $C_{27}H_{22}N_2NaO_2^+$ ([M+Na]$^+$): 429.1573, found: 429.1573.

$[\alpha]_D^{20}$ = 156.2 (c = 0.89, $CHCl_3$).

The ee value was determined by HPLC analysis on a AD-H column (n-hexane/i-PrOH = 80/20, flow = 1.2 mL/min, 254 nm), t = 13.1 min (major), t = 21.8 min (minor). The absolute stereochemistry was assigned by analogy to compound **55**.

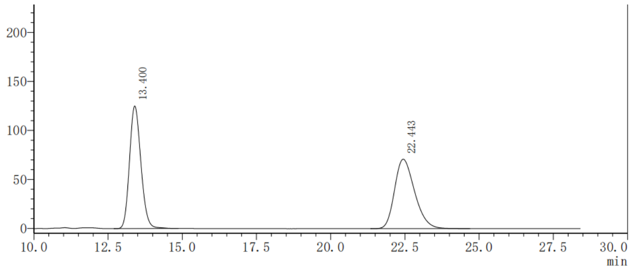

Peak	Ret. Time	Area	Height	Area%
1	13.400	3355017	125224	50.140
2	22.443	3336222	70874	49.860
Total		6691238	196098	100.000

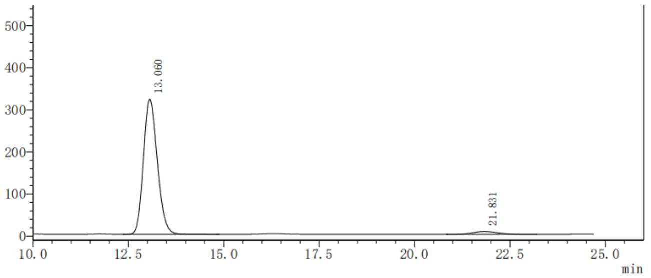

Peak	Ret. Time	Area	Height	Area%
1	13.060	8145172	320871	96.390
2	21.831	305010	6692	3.610
Total		8450182	327563	100.000

Supplementary Fig. 61 | Enantiomeric excess determination of product 57

Synthesis of 58

To an oven-dried 10 mL Schlenk tube was added **56** (0.1 mmol), *t*-BuOH (3 mL), and a solution of 2-methylbut-2-ene (13.0 equiv.), $NaClO_2$ (3.7 equiv.), NaH_2PO_4 (5.0 equiv.) in H_2O (1mL). The mixture was stirred for 2 h under room temperature. The resulting mixture was directly concentrated, and the residue was purified by preparative TLC to afford the product **58**. **58** was obtained as an off white solid (30 mg, 74% yield, 92% ee).

(***S***)-2-methyl-2-(2-(6-oxobenzo[*c*]phenanthridin-5(6H)-yl)phenyl)propanoic acid(**58**)

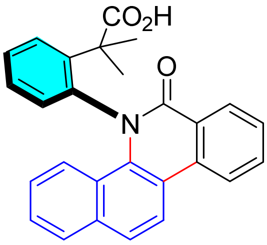

$[\alpha]_D^{20}$ = -93.9 (c = 1.08, $CHCl_3$). mp: 206.5-208 °C.

^{1}H NMR (400 MHz, Chloroform-*d*) δ 8.63 (dd, *J* = 8.0, 1.4 Hz, 1H), 8.50 (d, *J* = 8.3 Hz, 1H), 8.46 (d, *J* = 8.9 Hz, 1H), 7.99 – 7.86 (m, 3H), 7.76 – 7.66 (m, 2H), 7.62 (d, *J* = 9.1 Hz, 1H), 7.51 (t, *J* = 7.6 Hz, 1H), 7.45 (t, *J* = 7.4 Hz, 1H), 7.30 – 7.21 (m, 1H), 7.14 (ddd, *J* = 8.7, 6.8, 1.5 Hz, 1H), 6.71 (dd, *J* = 8.0, 1.4 Hz, 1H), 1.77 (s, 3H), 1.62 (s, 3H).

^{13}C NMR (101 MHz, Chloroform-*d*) δ 177.0, 166.0, 141.4, 141.0, 135.6, 135.1, 134.4, 133.9, 131.5, 129.7, 129.7, 129.6, 129.4, 128.9, 126.7, 126.4, 125.9, 125.3, 124.4, 124.3, 122.8, 120.4, 118.1, 49.6, 30.0, 24.3.

HRMS (ESI-TOF) calcd for $C_{27}H_{21}NNaO_3^+$ ($[M+Na]^+$): 430.1414, found: 430.1415.

The ee value was speculated by the compound **58 '**(methylation product of **58)**, HPLC analysis on an AD-H column (n-hexane/i-PrOH = 80/20, flow = 1.1 mL/min, 254 nm), t = 16.9 min (major), t = 9.2 min (minor). The absolute stereochemistry was assigned by analogy to compound **55**.

(***S***)-methyl 2-methyl-2-(2-(6-oxobenzo[*c*]phenanthridin-5(6H)-yl)phenyl)propanoate(**58′**)

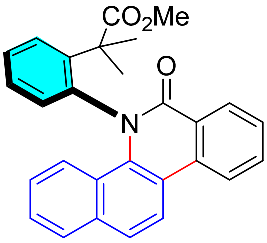

^{1}H NMR (400 MHz, Chloroform-*d*) δ 8.56 (dd, *J* = 7.8, 1.5 Hz, 1H), 8.40 (dd, *J* = 11.6, 8.6 Hz, 2H), 7.89 – 7.74 (m, 3H), 7.66 – 7.60 (m, 2H), 7.53 (ddd, *J* = 8.2, 7.2, 1.5 Hz, 1H), 7.37 (tdd, *J* = 7.6, 6.5, 1.2 Hz, 2H), 7.30 (d, *J* = 8.3 Hz, 1H), 7.07 – 6.95 (m, 2H), 3.12 (s, 3H), 1.52 (s, 3H), 1.48 (s, 3H).

^{13}C NMR (101 MHz, Chloroform-*d*) δ 176.8, 164.1, 142.5, 140.7, 135.6, 135.2, 134.8, 133.3, 131.3, 129.8, 129.3, 129.1, 128.7, 128.7, 128.2, 126.7, 126.2, 125.5, 125.1, 125.0, 124.5, 122.7, 120.7, 117.0, 51.9, 47.5, 27.7, 26.3.

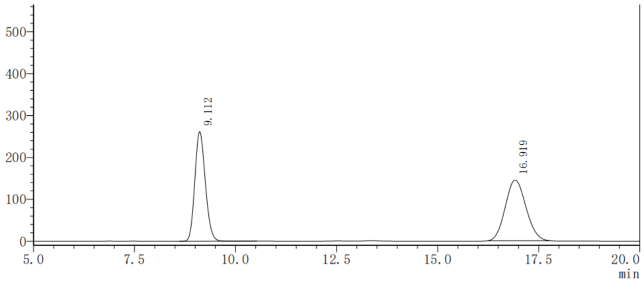

Peak	Ret. Time	Area	Height	Area%
1	9.112	4737777	261466	47.986
2	16.919	5135431	145030	52.014
Total		9873208	406495	100.000

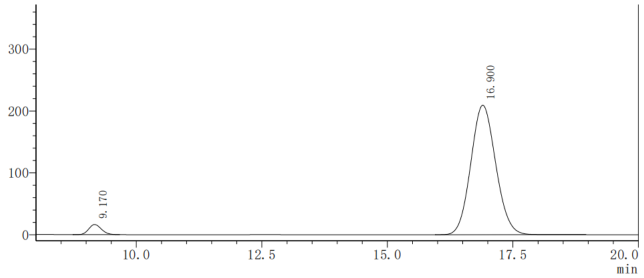

Peak	Ret. Time	Area	Height	Area%
1	9.170	286081	16386	3.795
2	16.900	7251965	209455	96.205
Total		7538045	225841	100.000

Supplementary Fig. 62 | Enantiomeric excess determination of product 58'

Application of chiral acid 58

The procedure for the synthesis of **61** and **63** refer to the known literature[4,5].

To a 20 mL Schlenk tube was added sulfoximines **59** (0.10 mmol), sulfoxonium ylides (0.15 mmol), chiral acid **58** (4.0 mg, 10 mol%), $AgSbF_6$ (6.9 mg, 20 mmol%), [(*p*cymene)$RuCl_2$]$_2$ (1.5 mg, 2.5 mol%) and DCE (2.0 mL). The reaction flask was degassed three times with N_2. Then stirred at 35 °C for 12 h. After cooling to room temperature, the mixture was diluted with ethyl acetate, filtrated through celite. After concentration, the resulting residue was purified by preparative TLC using hexane/EtOAc as the eluent to afford the desired product **61** as a yellow foam (30 mg, 96% yield, 55% ee).
The ee value was determined by HPLC analysis on a AS-H column (n-hexane/i-PrOH = 80/20, flow = 1.2 mL/min, 254 nm), t = 19.9 min (major), t = 12.3 min (minor).

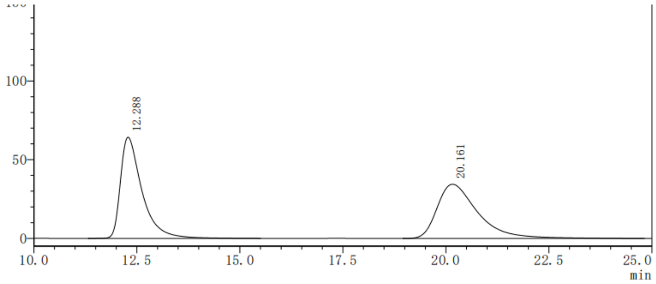

Peak	Ret. Time	Area	Height	Area%
1	12.288	2340055	64293	49.659
2	20.161	2372204	34445	50.341
Total		4712260	98737	100.000

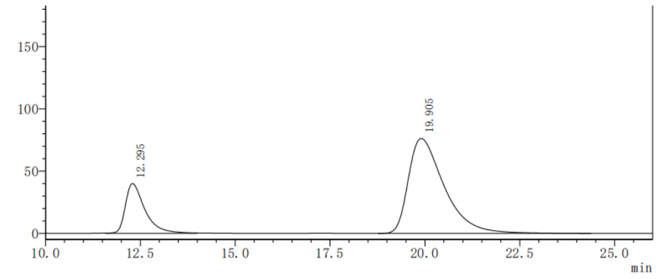

Peak	Ret. Time	Area	Height	Area%
1	12.295	1447907	40071	22.418
2	19.905	5010767	76289	77.582
Total		6458674	116360	100.000

Supplementary Fig. 63 | Enantiomeric excess determination of product 61

(***S***)-1,3-diphenylbenzo[*e*][1,2]thiazine 1-oxide(**61**)

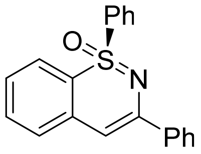

^{1}H NMR (600 MHz, Chloroform-*d*) δ 8.04 – 7.98 (m, 4H), 7.65 (t, *J* = 7.6 Hz, 1H), 7.58 (t, *J* = 7.4 Hz, 2H), 7.49 (t, *J* = 7.8 Hz, 1H), 7.43 (dd, *J* = 18.6, 8.8 Hz, 3H), 7.37 (d, *J* = 7.3 Hz, 1H), 7.33 (d, *J* = 8.2 Hz, 1H), 7.23 (t, *J* = 7.2 Hz, 1H), 6.83 (s, 1H).

Ruthenium(II)-Catalyzed Asymmetric C−H Alkylation for the synthesis of Tetrahydrocarbazoles

A suspension of indole **62** (0.1 mmol, 1.0 equiv), $[RuCl_2(p\text{-cymene})]_2$ (3.0 mg, 0.005 mmol), $AgSbF_6$ (6.9 mg, 0.02 mmol), chiral acid **58** (8.1 mg, 0.02 mmol) in EtOAc (1.0 mL) were stirred at 25 °C for 12 h. The reaction mixture was diluted with EtOAc (2.0 mL) and the solvent was removed *in vacuo*. The crude mixture was purified by column chromatography on silica gel to afford the desired product **63** as a colorless oil (25 mg, 90% yield, 50% ee).

The ee value was determined by HPLC analysis on a IG column (n-hexane/i-PrOH = 92/8, flow = 1.0 mL/min, 254 nm), t = 7.3 min (major), t = 6.6 min (minor).

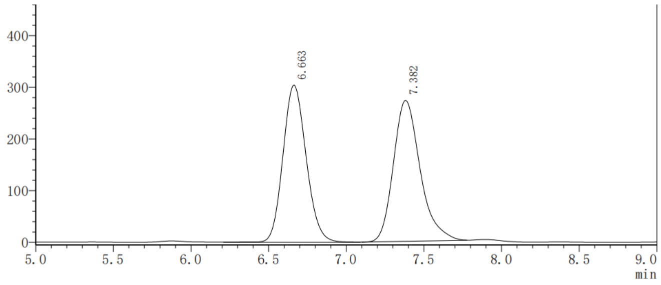

Peak	Ret. Time	Area	Height	Area%
1	6.663	3171344	303754	49.299
2	7.382	3261582	272581	50.701
Total		6432926	576335	100.000

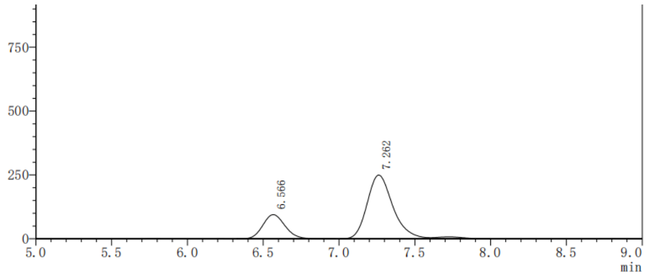

Peak	Ret. Time	Area	Height	Area%
1	6.566	978401	95232	24.314
2	7.262	3045553	249467	75.686
Total		4023954	344699	100.000

Supplementary Fig. 64 | Enantiomeric excess determination of product 63

(***R***)-1-methyl-9-(5-methylpyridin-2-yl)-2,3,4,9-tetrahydro-1H-carbazole(**63**)

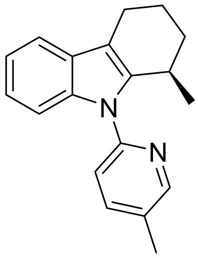

^{1}H NMR (600 MHz, Chloroform-*d*) δ 8.45 (s, 1H), 7.67 (dd, *J* = 8.2, 2.4 Hz, 1H), 7.50 (dd, *J* = 5.7, 3.2 Hz, 1H), 7.34 (d, *J* = 7.7 Hz, 2H), 7.13 (dt, *J* = 6.3, 3.6 Hz, 2H), 3.48 (m, 1H), 2.86 – 2.66 (m, 2H), 2.43 (s, 3H), 2.11 (tt, *J* = 12.7, 9.8, 4.6 Hz, 1H), 1.97 – 1.84 (m, 2H), 1.65 (m, 1H), 0.84 (d, *J* = 6.9 Hz, 3H).

2.5 X-ray Crystallographic Data

A single crystal of **55** suitable for X-ray crystallography (CDCC: 2249376 (**55**)) was obtained by crystallization via evaporation from its chloroform solution. The structure is of publishable quality (no A-alerts and B-alerts, see CIF/checkCIF) with a R1 value of 4.16%.

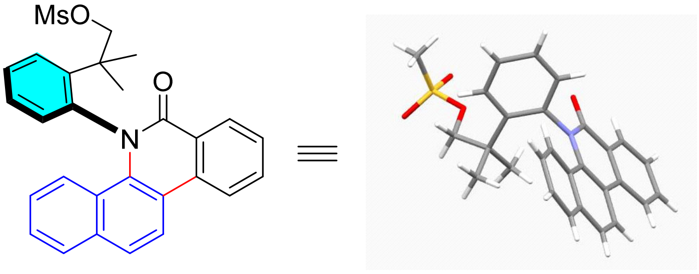

Supplementary Fig. 65 | X-Ray crystallographic data of 55. Thermal ellipsoids are shown at the 30% level.

Supplementary Table 4 | Crystal data and structure refinement for 55.

Bond precision:	C-C = 0.0030 A		Wavelength=1.34139
Cell:	a=9.4766(3)	b=11.4929(4)	c=10.8760(4)
	alpha=90	beta=107.443(1)	gamma=90
Temperature:	170 K		

	Calculated	Reported
Volume	1130.07(7)	1130.07(7)
Space group	P 21	P 1 21 1
Hall group	P 2yb	P 2yb
Moiety formula	C28 H25 N O4 S	C28 H25 N O4 S
Sum formula	C28 H25 N O4 S	C28 H25 N O4 S
Mr	471.55	471.55
Dx, g cm-3	1.386	1.386
Z	2	2
Mu (mm-1)	1.025	1.025
F000	496.0	496.0
F000’	497.65	
h, k, lmax	12,14,14	12,14,14
Nref	5197[2730]	4653
Tmin, Tmax	0.873, 0.903	0.645, 0.752
Tmin’	0.630	

Correction method= # Reported T Limits: Tmin=0.645 Tmax=0.752

AbsCorr = MULTI–SCAN

Data completeness= 1.70/0.90 Theta(max)= 60.633

R(reflections)= 0.0416(4585) wR2(reflections)= 0.0966(4653)

S = 1.133 Npar= 310

Flack parameter 0.067(7)

2.6 Enantiomerization Barrier Determination of 3

The enantiomerization barrier, corresponding to the barrier to rotation for the following atropisomers, was obtained by kinetic of racemization of an enantiomer.[6] The slope of the firstorder kinetic line gives the racemization constant ($k_{racemization}$ = 2 x $k_{enantiomerization}$). Eyring equation gives the enantiomerization barrier ($\Delta G^{\ddagger}_{enaniomerization}$) from enantiomerisation constant ($k_{enantiomerization}$), R = Gas constant = 8.31451 J.K^{-1}.mol^{-1}, h = Planck constant = 6.62608 x 10^{-34} J.s and k_B = Boltzmann constant = 1.38066 x 10^{-23} J.K^{-1}.

$$\Delta G^{\ddagger}_{enaniomerization} = RT_1 \ln \frac{k_B T_1}{hk_{enantiomerization}}$$

The half-life time ($t_{1/2}$), given below, is at the temperature used for the kinetic. Reactions were conducted at 1 mg/mL concentration in N_2-filled sealed tubes to minimize air oxidation. Enantiomeric ratios were determined by HPLC.

Racemization of 3 in *i*-PrOH at 150 °C

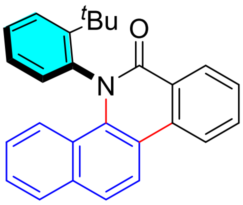

3

Supplementary Table 5 | Rotational barrier measurement data for product 3.

Time (second)	Enantiomeric Excess (ee)	First Order Racemization $\ln(ee_0/ee_t)$
0	91.325	0
1860	87.574	0.038375
3720	85.25	0.065271
5520	82.524	0.09777
7320	78.98	0.141665
9180	77.692	0.158107
10740	74.352	0.202049
12480	70.678	0.252725
14580	67.348	0.300986
16440	64.164	0.349417
20040	59.918	0.417883

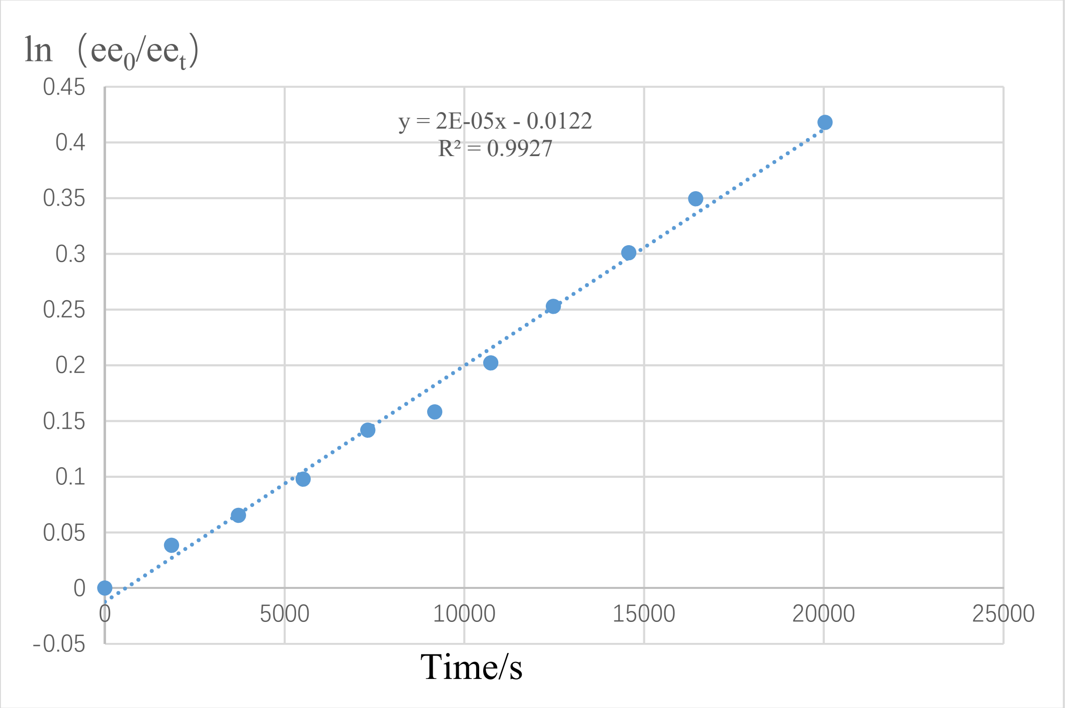

Supplementary Fig. 66 | Plot for the rotational barrier measurement of compound 3.

$k_{racemization}$ (150 °C) = 2×10^{-5} s^{-1}

$k_{enantiomerization}$ (150 °C) = 1×10^{-5} s^{-1}

$\Delta G^{\ddagger}$ $_{enaniomerization}$ = 145.3 KJ/mol = 34.7 kcal/mol

$t_{1/2}$ (150 °C) = 8.48 hours

2.7 Optical properties

Uv-vis Spectra and Fluorescence Spectra

Solutions of **21, 33, 52, 53,** and **54** (20 μM in DCM), 10 mm × 10 mm quartz cell.

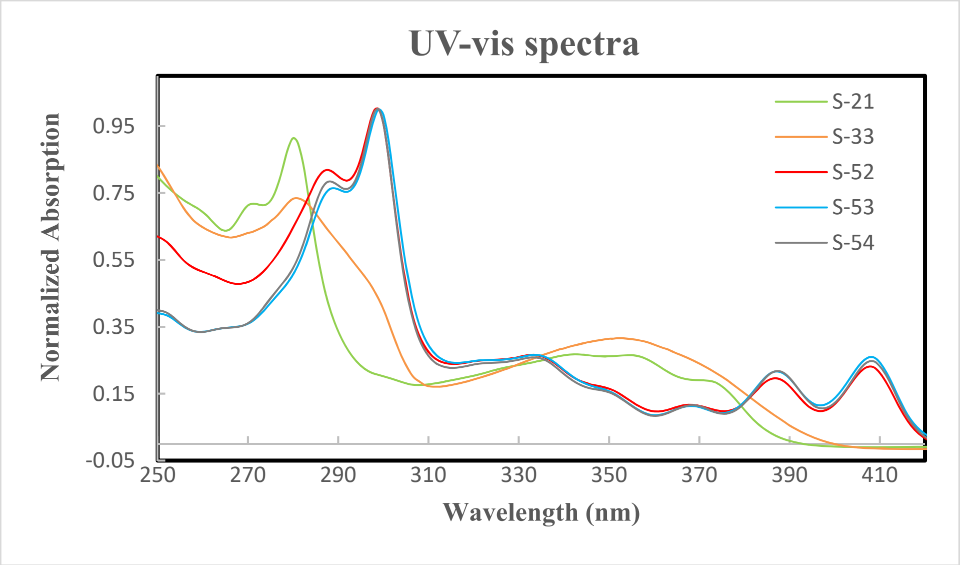

Supplementary Fig. 67 | UV-vis spectra of 21, 33, 52, 53, and 54.

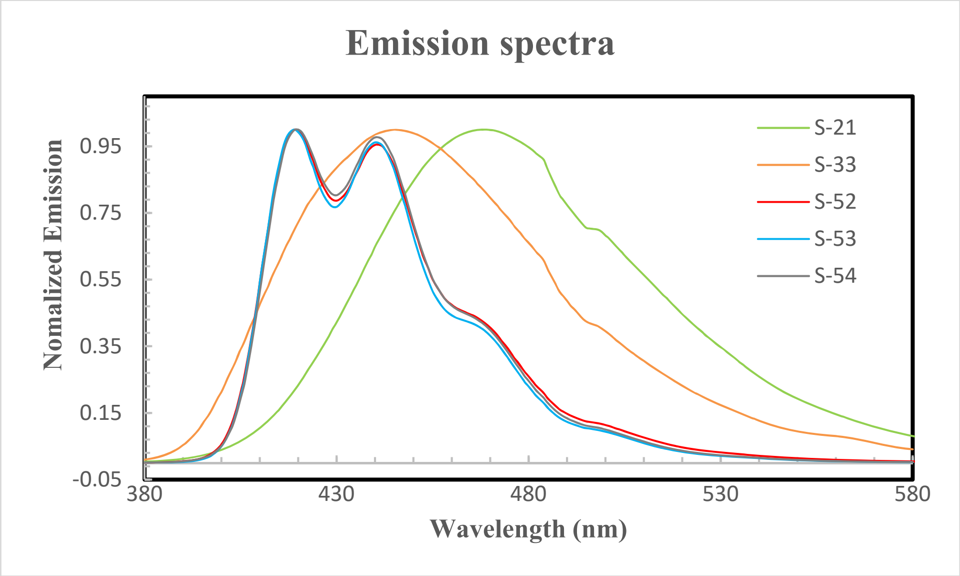

Supplementary Fig. 68 | Emission spectra of 21, 33, 52, 53, and 54.

Supplementary Table 6 | Absorption Maxima, Emission Maxima, and Stokes Shifts of 21, 33, 52, 53, and 54.

Compound	λ_{abs} (nm)	λ_{em} (nm)	Stokes Shift (nm)
**21**	280, 342, 354	469	189
**33**	280, 352	445	165
**52**	288, 299, 333	419, 441	120

**53**	289, 299, 333	419, 440	120
**54**	288, 299, 333	420, 440	121

Circular polarized luminescence (CPL) spectra

Solutions of **52**, **53**, and **54** (1.0 mM in DCM), 10 mm × 10 mm quartz cell.

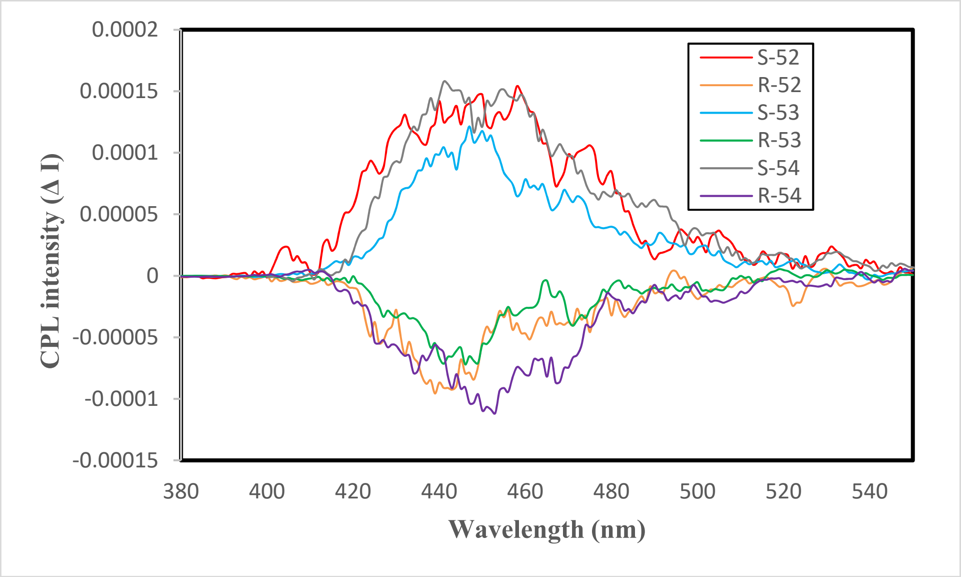

Supplementary Fig. 69 | Circular Polarized Luminescence (CPL) Spectra of 52, 53, 54, and their enantiomers.

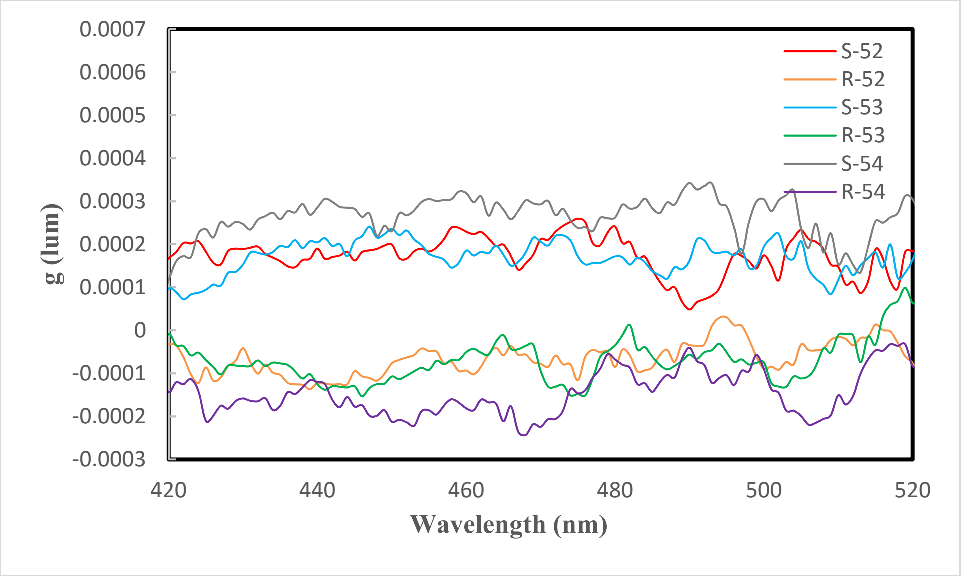

Supplementary Fig. 70 | g_{lum} values–wavelength curve for 52, 53, 54, and their enantiomers.

Supplementary Table 7 | Asymmetric Factors of 52, 53, 54, and their enantiomers.

Compound	Excitation wavelength (nm)	g_{lum} (range) (420 ~ 450 nm)
**52**	300	1.8×10^{-4}
**52-ent**	300	-9.8×10^{-5}
**53**	300	2.2×10^{-4}

**53-ent**	300	-1.3×10^{-4}
**54**	300	2.2×10^{-4}
**54-ent**	300	-2.0×10^{-4}

For each compound, the corresponding g_{lum} value in the table is the average of g_{lum} values within the giving range of wavelength indicated in the parentheses.

Photoluminescence Quantum Yield (PLQY)

The PLQY was measured in an integrating sphere. The sample solution (20 µM inDCM about 2 mL) was contained in a 10×10 mm quartz cell with a long neck.

Supplementary Table 8 | Photoluminescence quantum yield (PLQY) of 21, 33, 52, 53, and 54.

Compound	Excitation wavelength (nm)	Integral interval of excitation light (nm)	Integral interval of emission light (nm)	PLQY
**21**	280	270 ~ 296	350 ~ 580	0.97
**33**	280	270 ~ 296	350 ~ 580	0.40
**52**	300	286 ~ 318	380 ~ 600	0.47
**53**	300	286 ~ 318	380 ~ 600	0.47
**54**	300	286 ~ 318	380 ~ 600	0.51

2.8 Unsuccessful substrates

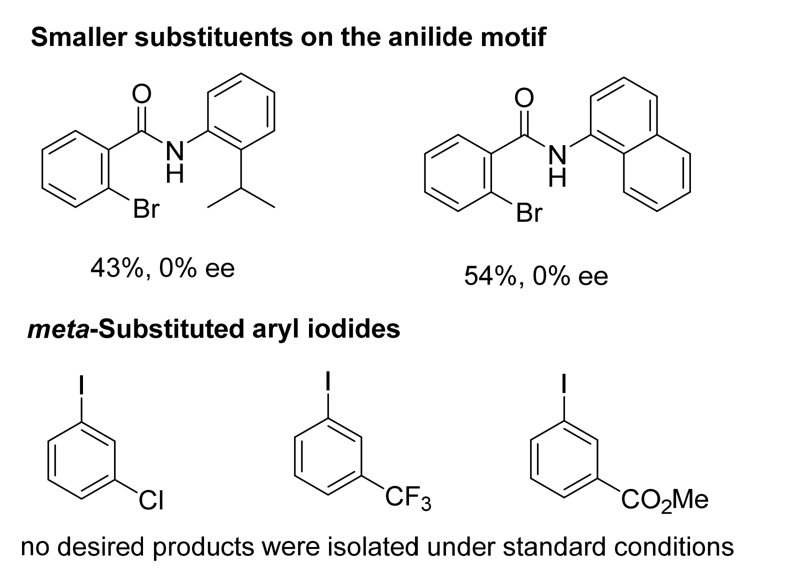

Supplementary Fig. 71 | Unsuccessful substrates

5-(2-isopropylphenyl)benzo[c]phenanthridin-6(5H)-one (**64**)

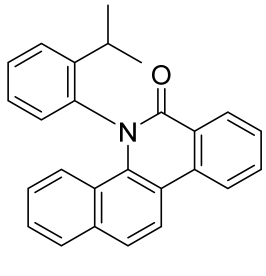

The compound was prepared according to **General Procedure C** and purified by preparative TLC in hexane/EtOAc = 6/1 as the eluent to afford a colorless oil (15 mg, 43% yield, 0% ee).

^{1}H NMR (600 MHz, Chloroform-*d*) δ 8.59 (d, *J* = 6.5 Hz, 1H), 8.43 (d, *J* = 8.3 Hz,1H), 8.39 (d, *J* = 8.8 Hz, 1H), 7.89 – 7.85 (m, 1H), 7.81 (d, *J* = 6.7 Hz, 1H), 7.77 (d, *J* = 8.8 Hz, 1H), 7.64 (t, *J* = 7.5 Hz, 1H), 7.52 (q, *J* = 4.6, 3.4 Hz, 2H), 7.40 – 7.31 (m, 2H), 7.28 – 7.19 (m, 2H), 6.99 (ddd, *J* = 8.6, 6.7, 1.5 Hz, 1H), 2.70 (hept, *J* = 6.9 Hz, 1H), 1.16 (d, *J* = 6.8 Hz, 3H), 0.79 (d, *J* = 6.8 Hz, 3H).

^{13}C NMR (151 MHz, Chloroform-*d*) δ 163.3, 146.5, 140.6, 135.5, 135.4, 134.8, 133.2, 129.5, 129.3, 129.3, 128.8, 128.1, 127.5, 127.2, 126.3, 125.9, 125.7, 125.1, 124.9, 124.4, 122.6, 120.6, 116.6, 28.5, 24.1, 22.9.

HRMS (ESI-TOF) calcd for $C_{26}H_{22}NO^{+}$ ($[M+H]^{+}$): 364.1696, found: 364.1698.

The ee value was determined by HPLC analysis on a AD column (n-hexane/i-PrOH = 85/15, flow = 1.1 mL/min, 254 nm), t = 7.0 min (major), t = 8.0 min (minor).

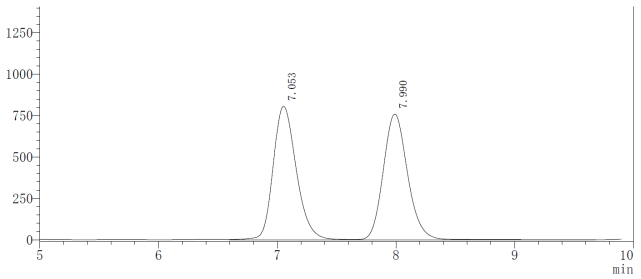

Peak	Ret. Time	Area	Height	Area%
1	7.053	10905267	805987	50.356
2	7.990	10751123	758647	49.644
Total		21656390	1564635	100.000

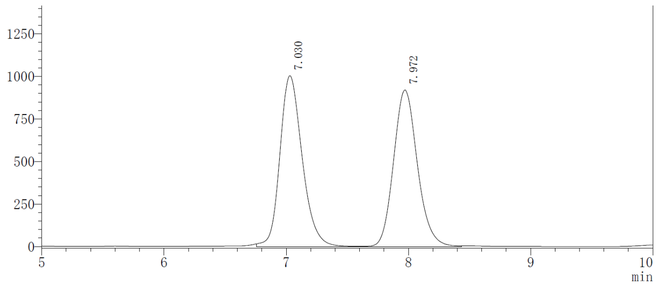

Peak	Ret. Time	Area	Height	Area%
1	7.030	12678462	1004054	50.502
2	7.972	12426212	920427	49.498
Total		25104674	1924480	100.000

Supplementary Fig. 72 | Enantiomeric excess determination of product 64

5-(naphthalen-1-yl)benzo[c]phenanthridin-6(5H)-one (**65**)

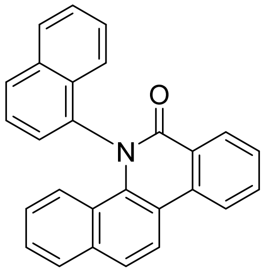

The compound was prepared according to **General Procedure D** and purified by preparative TLC in hexane/EtOAc = 6/1 as the eluent to afford a colorless oil (20 mg, 54% yield, 0% ee).

^{1}H NMR (600 MHz, Chloroform-*d*) δ 8.58 (d, *J* = 7.9 Hz, 1H), 8.47 (d, *J* = 8.3 Hz, 1H), 8.42 (d, *J* = 8.8 Hz, 1H), 7.99 (t, *J* = 7.9 Hz, 2H), 7.92 – 7.84 (m, 2H), 7.79 (t, *J* = 9.0 Hz, 2H), 7.65 (t, *J* = 7.5 Hz, 1H), 7.55 (ddd, *J* = 8.1, 6.8, 1.2 Hz, 1H), 7.52 – 7.45 (m, 2H), 7.28 – 7.24 (m, 1H), 7.23 (d, *J* = 7.1 Hz, 1H), 7.08 (d, *J* = 9.0 Hz, 1H), 6.75 (ddd, *J* = 8.6, 6.8, 1.5 Hz, 1H).

^{13}C NMR (151 MHz, Chloroform-*d*) δ 163.6, 140.6, 136.0, 135.2, 134.9, 134.4, 133.4, 131.8, 129.4, 129.1, 128.7, 128.6, 128.3, 127.6, 126.7, 126.4, 126.2, 125.9, 125.7, 125.5, 125.3, 125.1, 124.4, 123.8, 122.6, 120.4, 117.3.

HRMS (ESI-TOF) calcd for $C_{27}H_{18}NO^{+}$ ($[M+H]^{+}$): 372.1383, found: 372.1383.

The ee value was determined by HPLC analysis on a AD column (n-hexane/i-PrOH = 85/15, flow = 1.1 mL/min, 254 nm), t = 20.8 min (major), t = 26.7 min (minor).

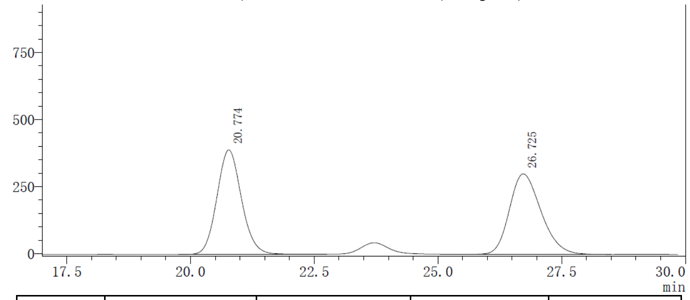

Peak	Ret. Time	Area	Height	Area%
1	20.774	13496088	389209	50.279
2	26.725	13346353	299862	49.721
Total		26842442	689071	100.000

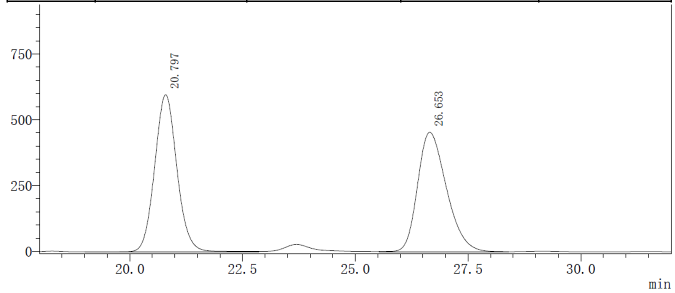

Peak	Ret. Time	Area	Height	Area%
1	20.797	20513833	596648	50.327
2	26.653	20246949	453804	49.673
Total		40760782	1050452	100.000

Supplementary Fig. 73 | Enantiomeric excess determination of product 65

3. NMR Spectra

1b

^{1}H NMR (400 MHz, Chloroform-*d*)

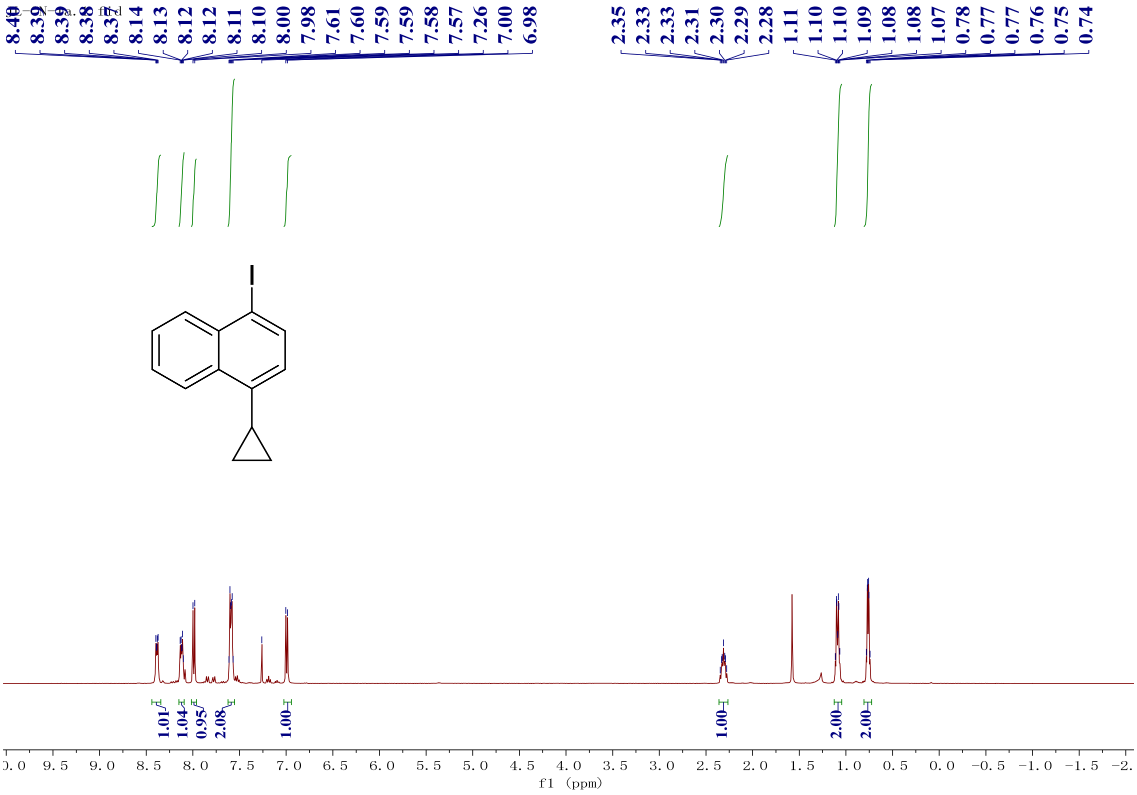

^{13}C NMR (101 MHz, Chloroform-*d*)

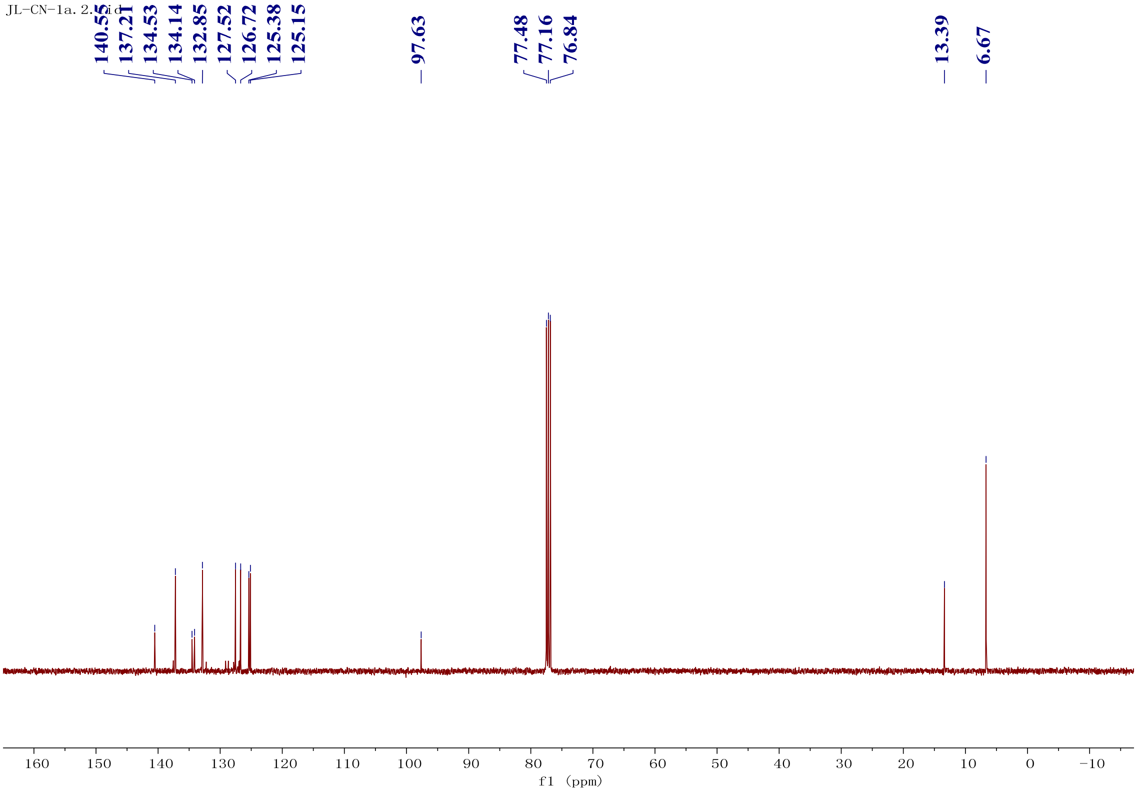

1c

^{1}H NMR (400 MHz, Chloroform-*d*)

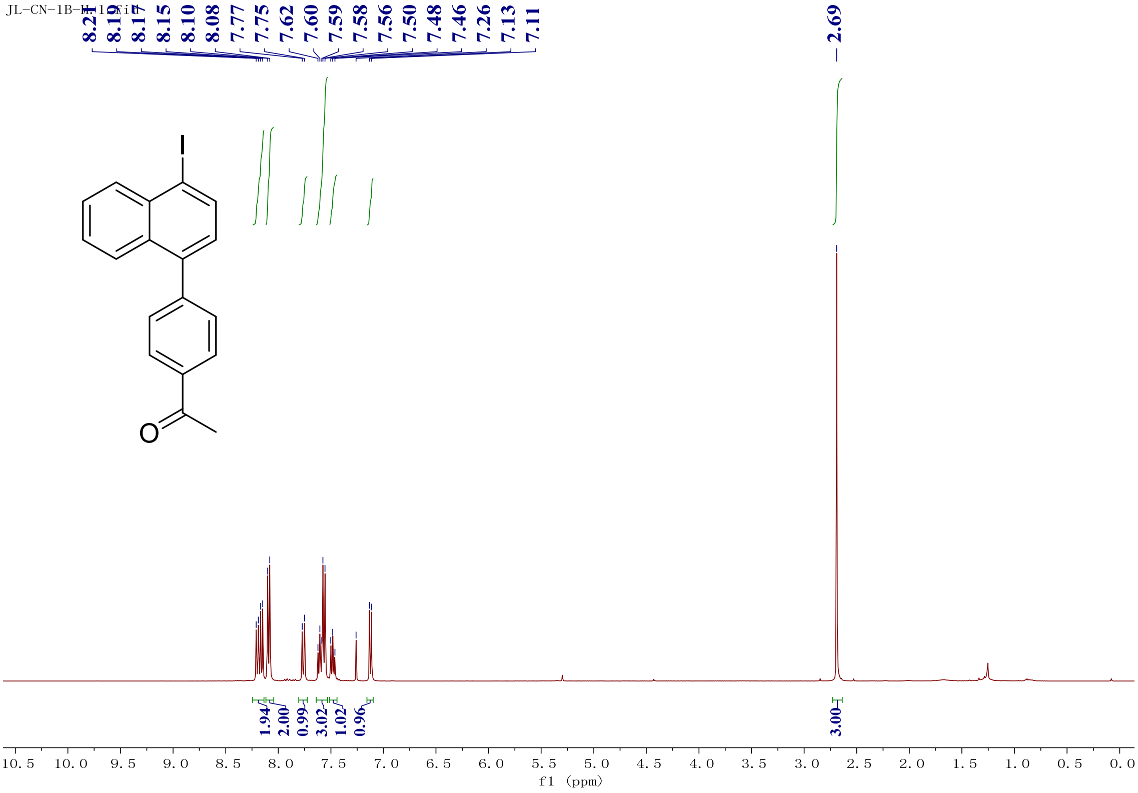

^{13}C NMR (101 MHz, Chloroform-*d*)

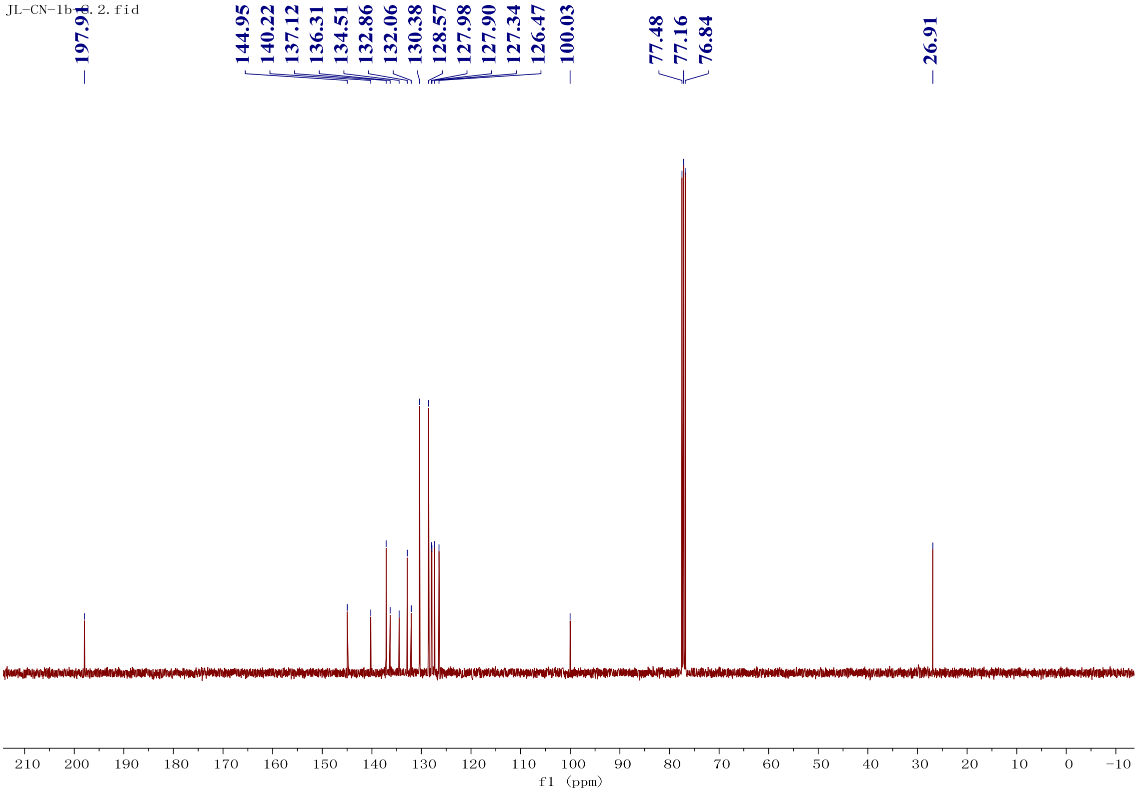

1d

^{1}H NMR (400 MHz, Chloroform-*d*)

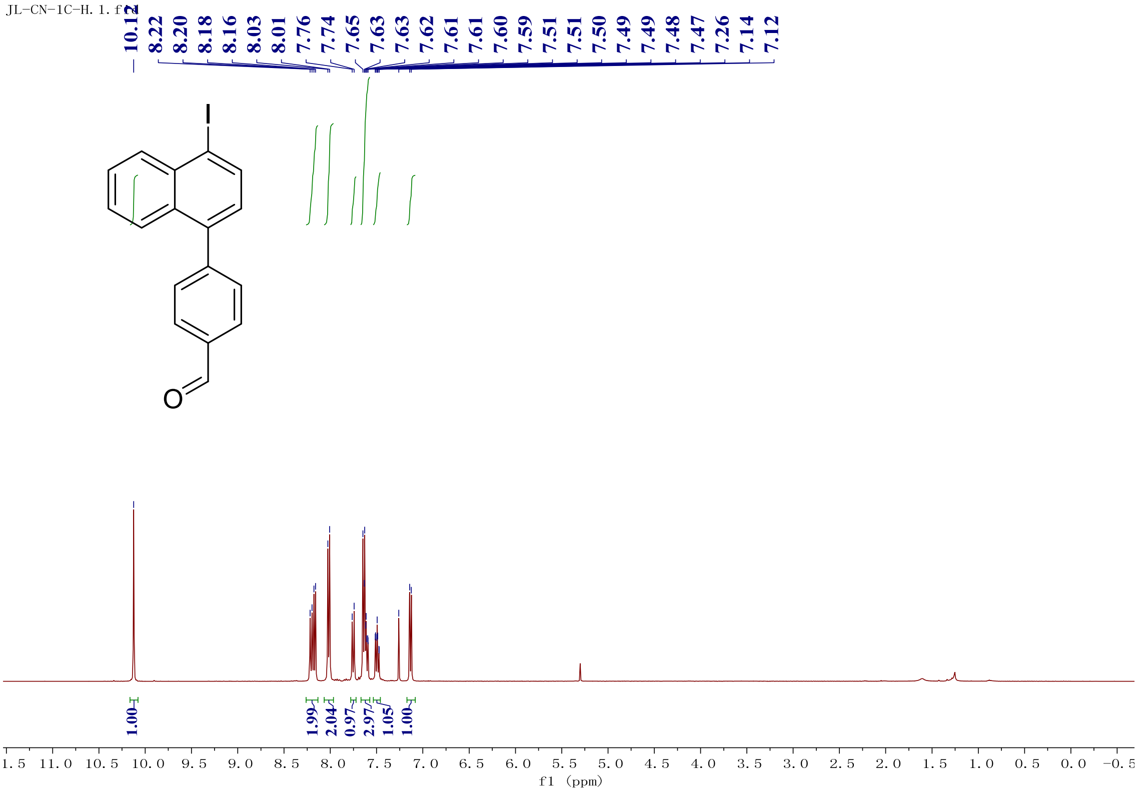

^{13}C NMR (101 MHz, Chloroform-*d*)

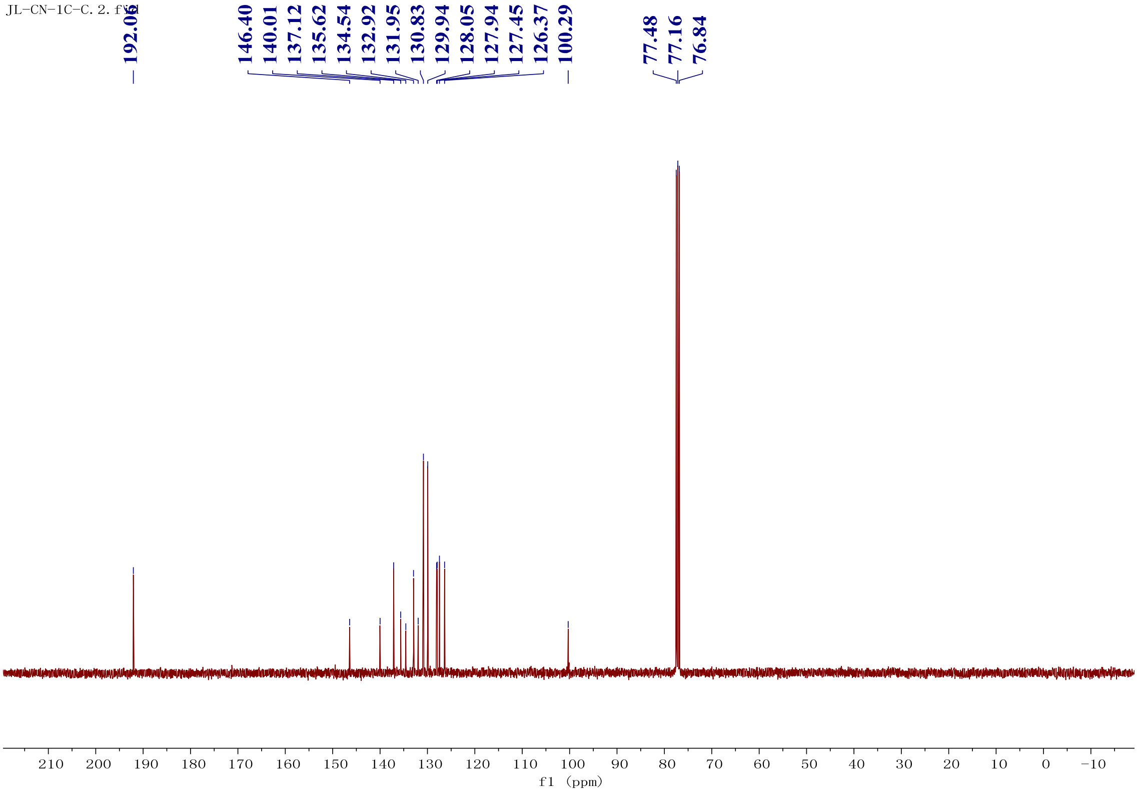

1e

^{1}H NMR (400 MHz, Chloroform-*d*)

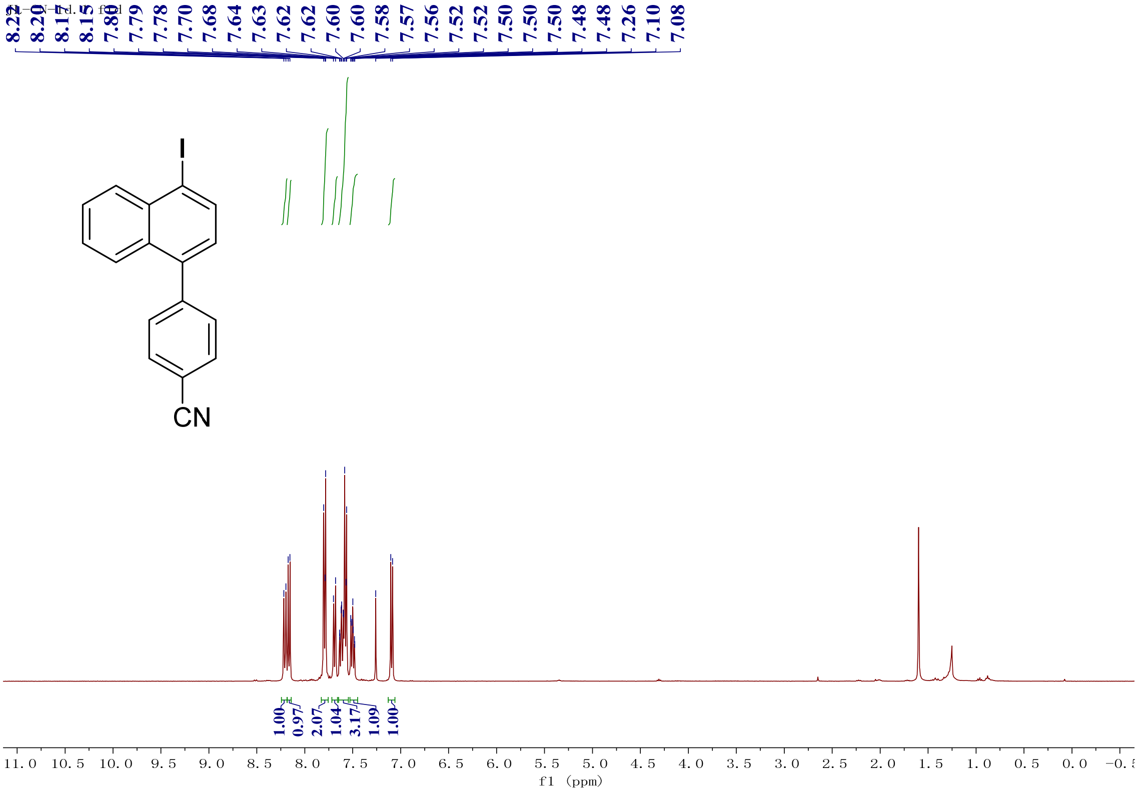

^{13}C NMR (101 MHz, Chloroform-*d*)

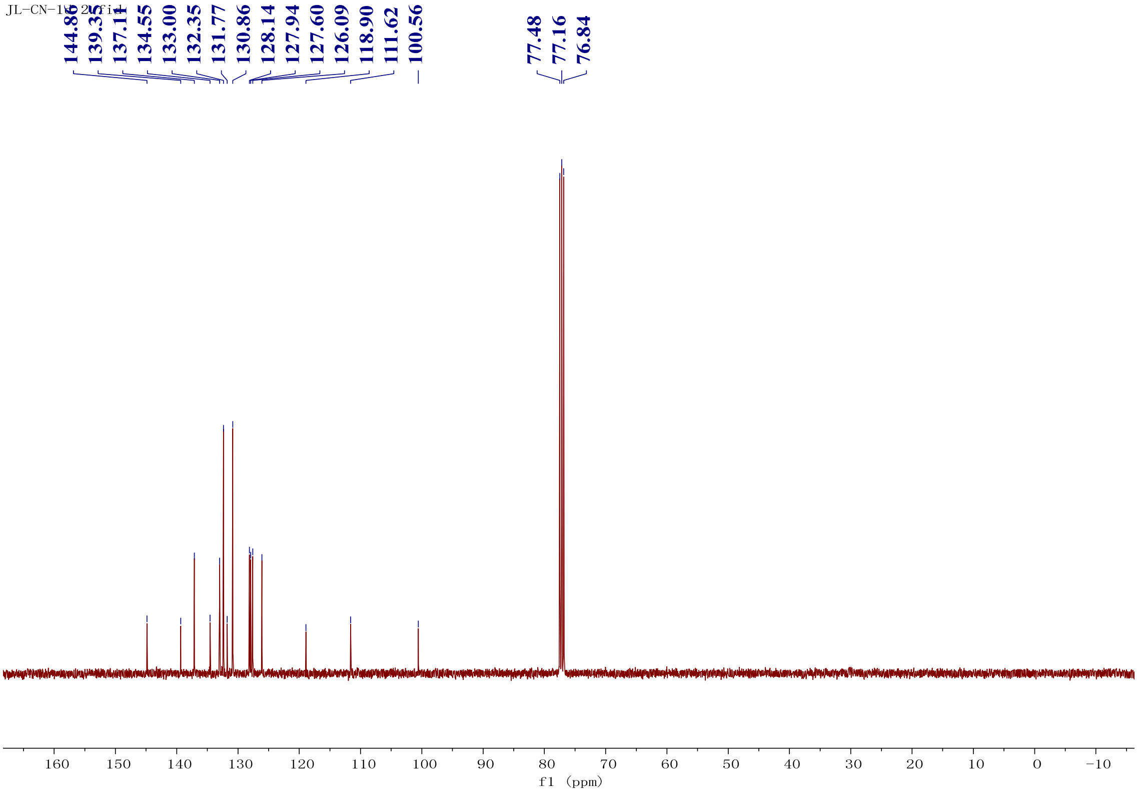

1f

^{1}H NMR (400 MHz, Chloroform-*d*)

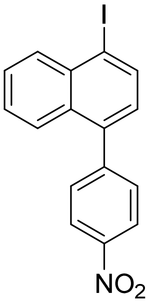

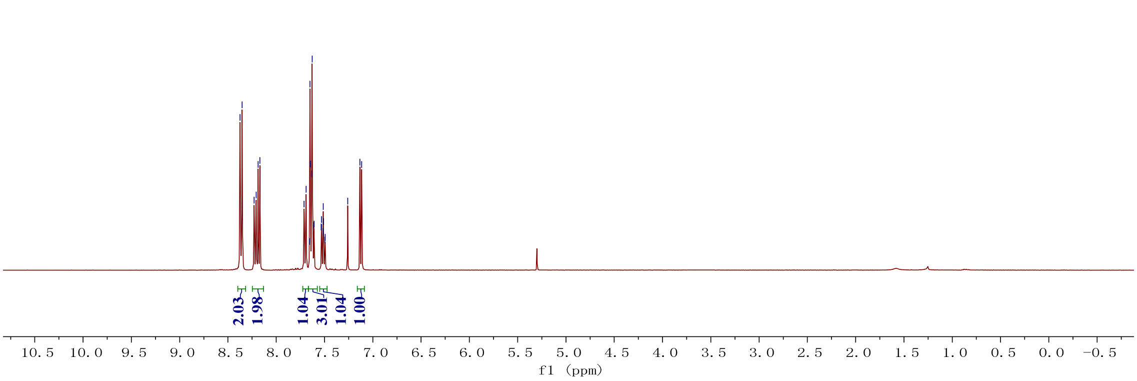

^{13}C NMR (101 MHz, Chloroform-*d*)

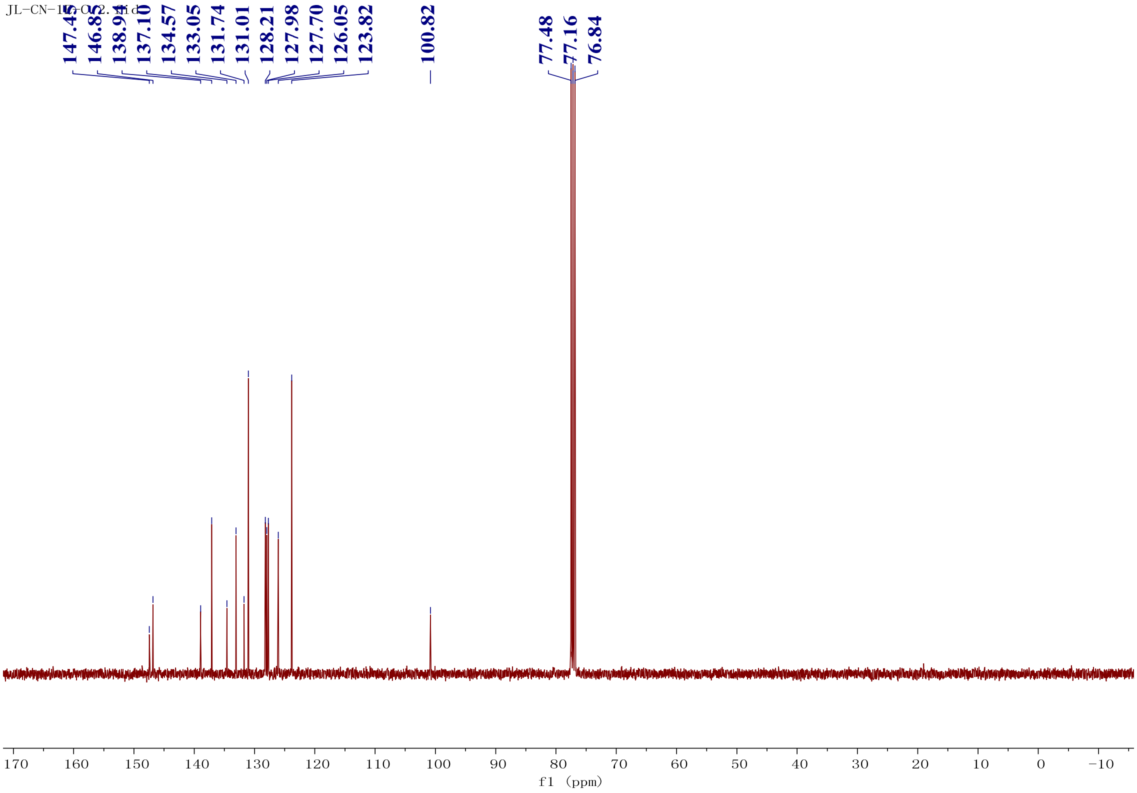

1g

^{1}H NMR (400 MHz, Chloroform-*d*)

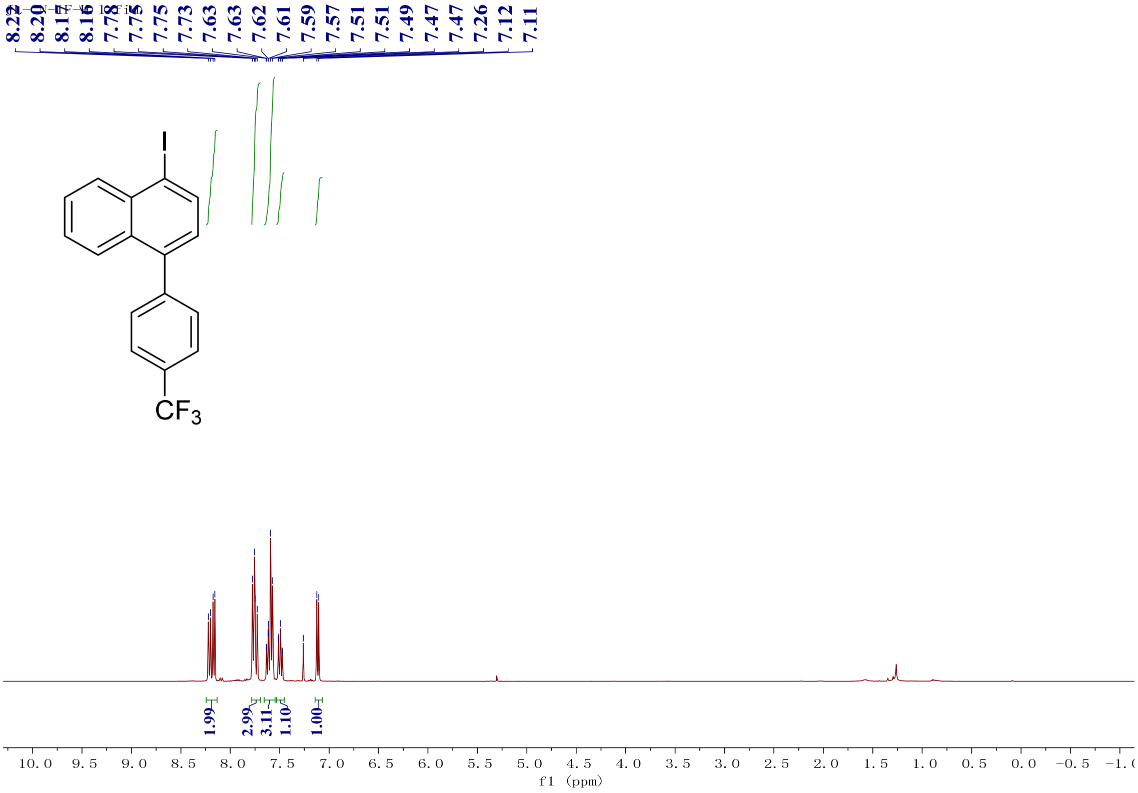

^{13}C NMR (101 MHz, Chloroform-*d*)

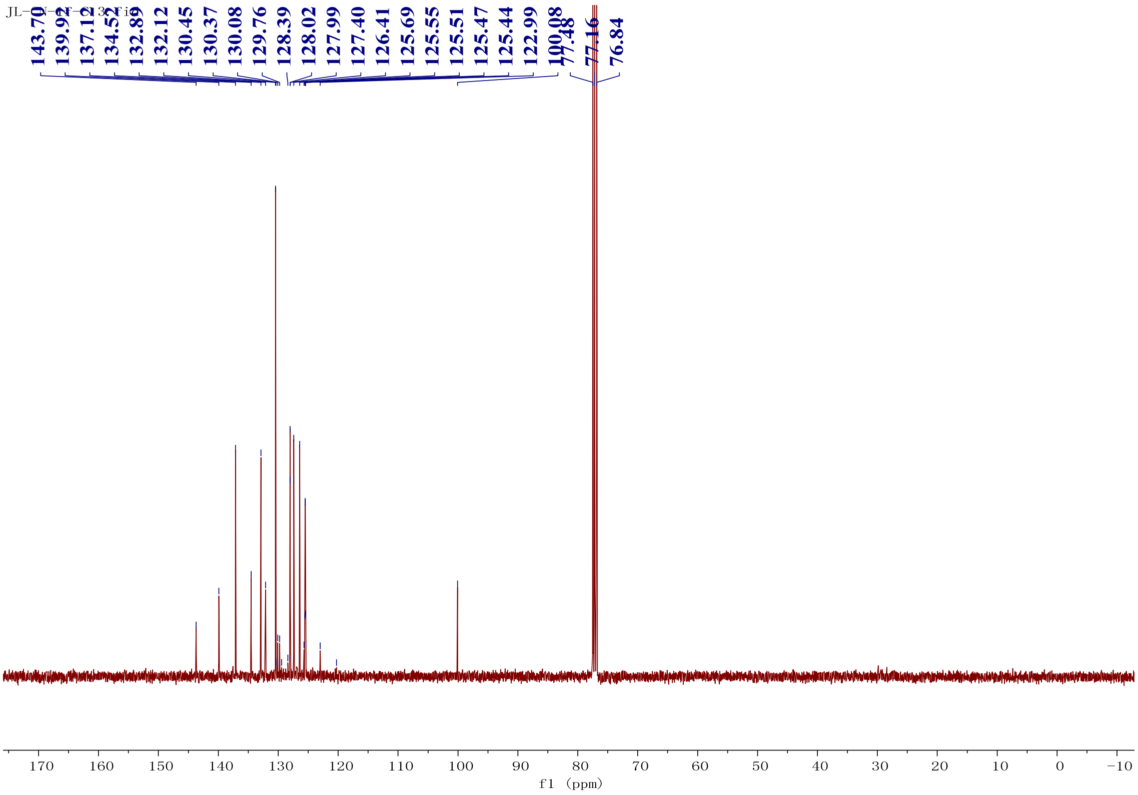

^{19}F NMR (376 MHz, Chloroform-*d*)

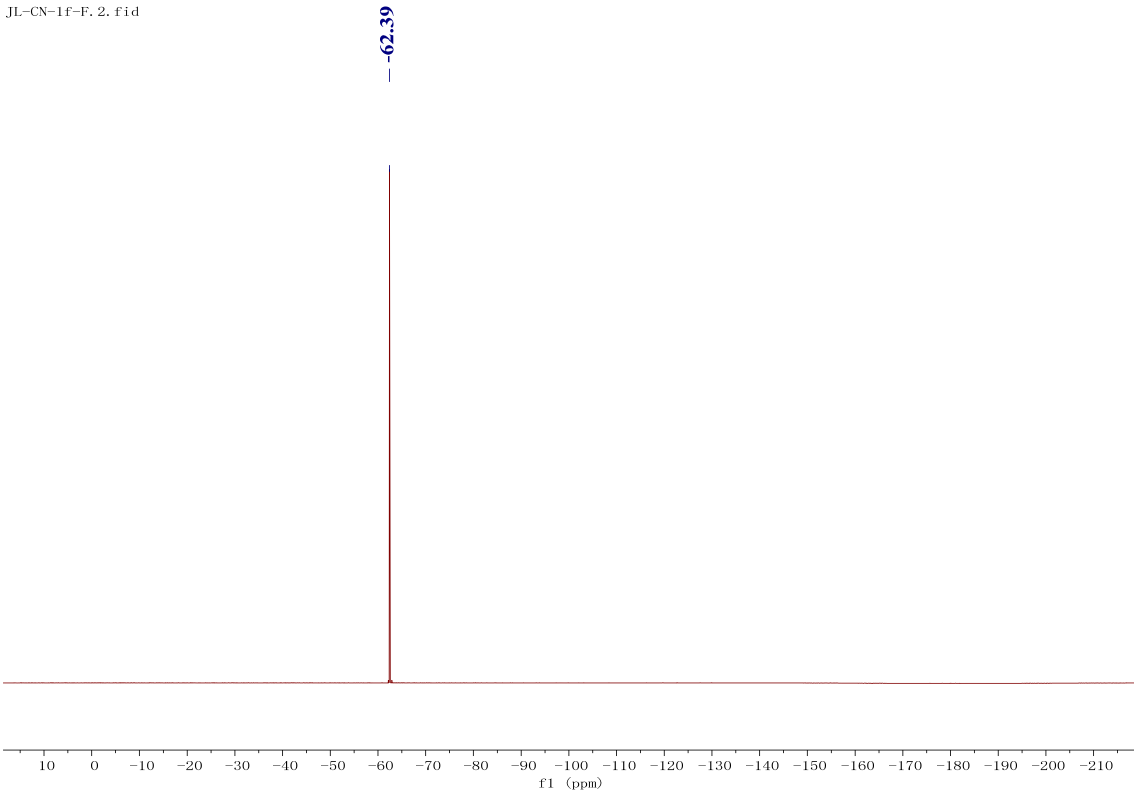

1h

^{1}H NMR (400 MHz, Chloroform-*d*)

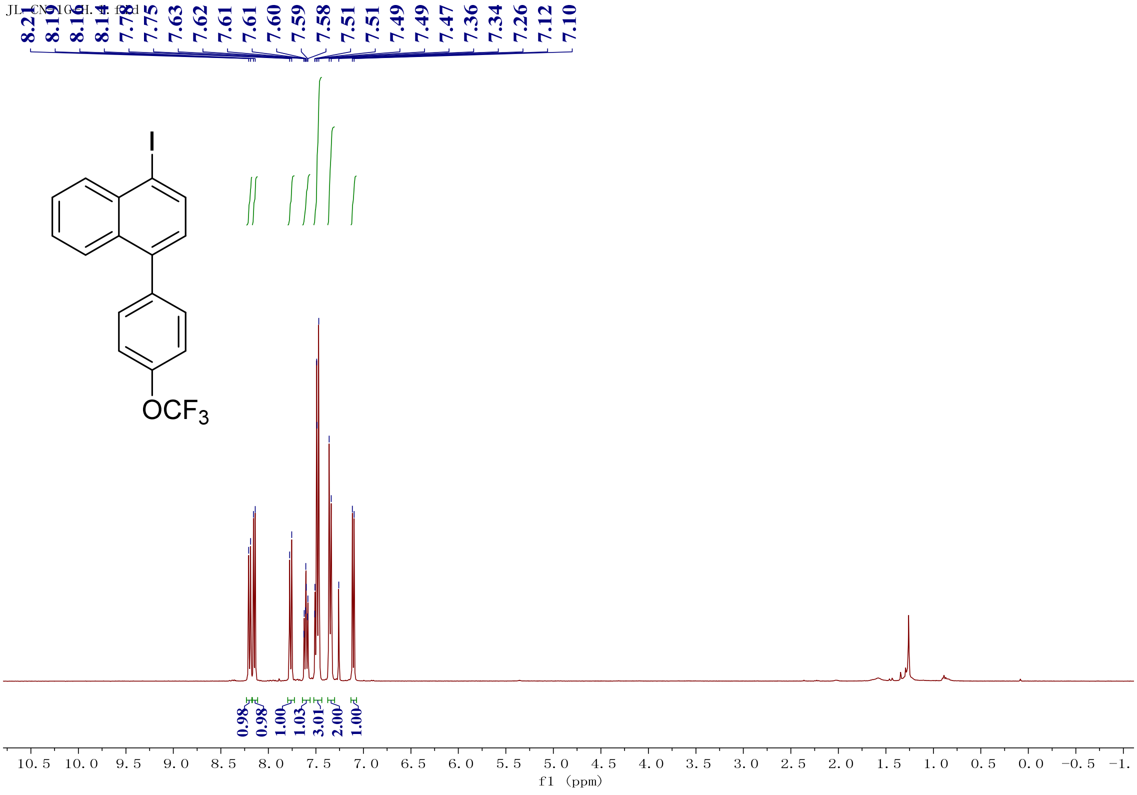

^{13}C NMR (101 MHz, Chloroform-*d*)

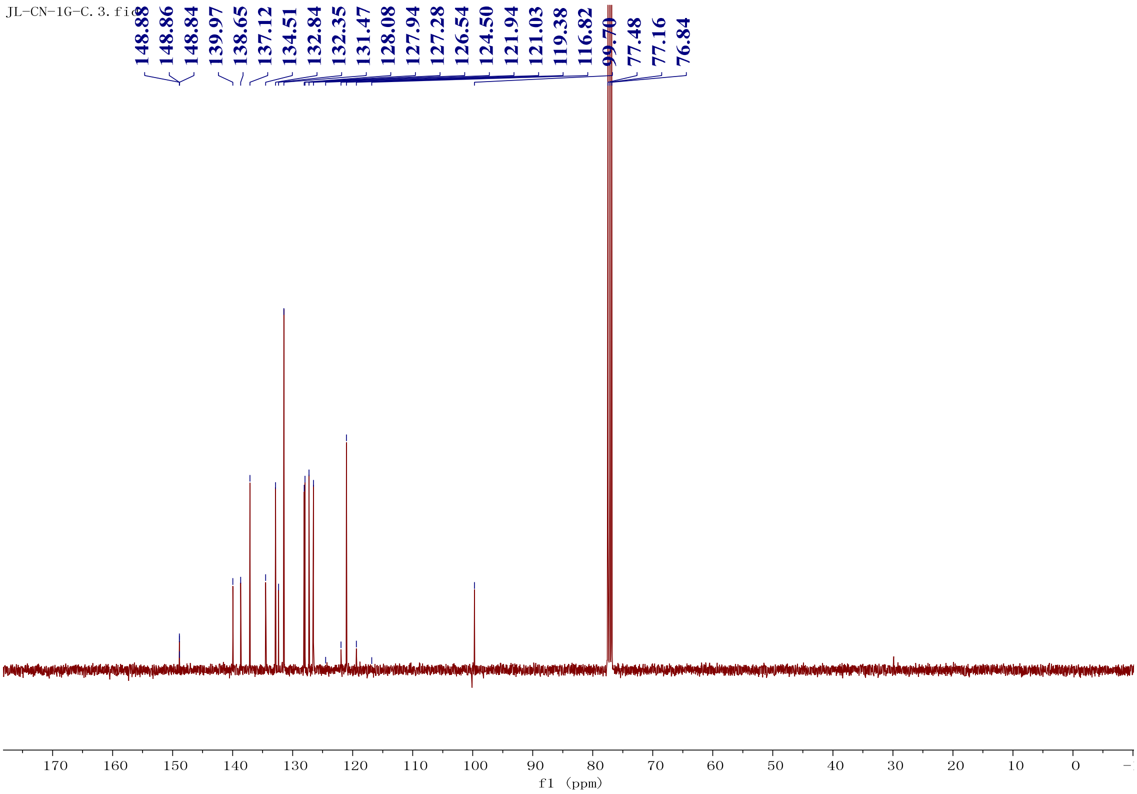

^{19}F NMR (376 MHz, Chloroform-*d*)

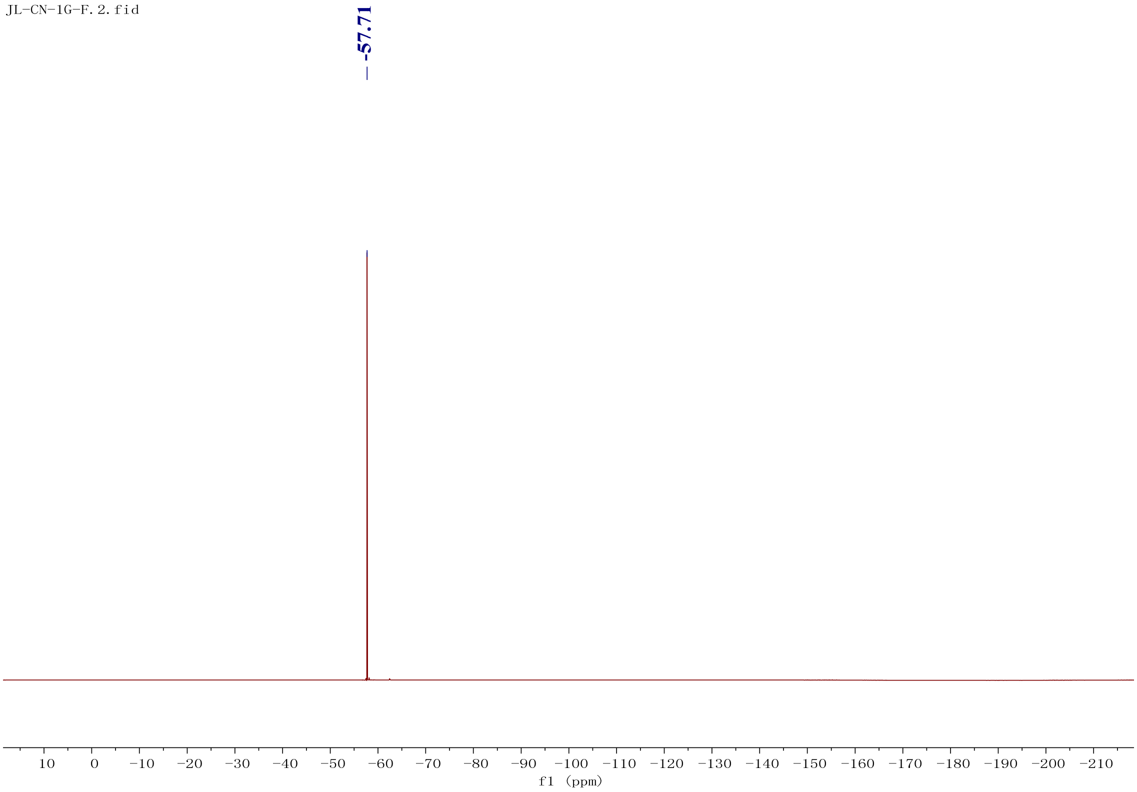

1i

^{1}H NMR (400 MHz, Chloroform-*d*)

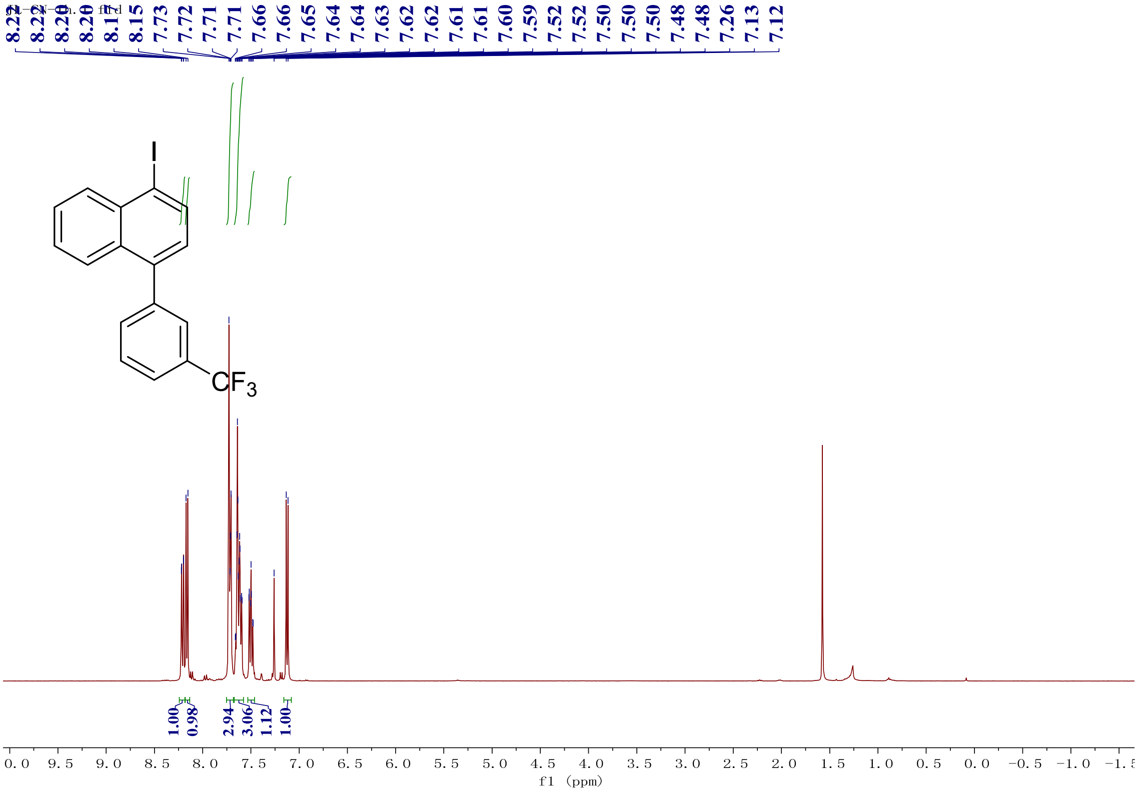

^{13}C NMR (101 MHz, Chloroform-*d*)

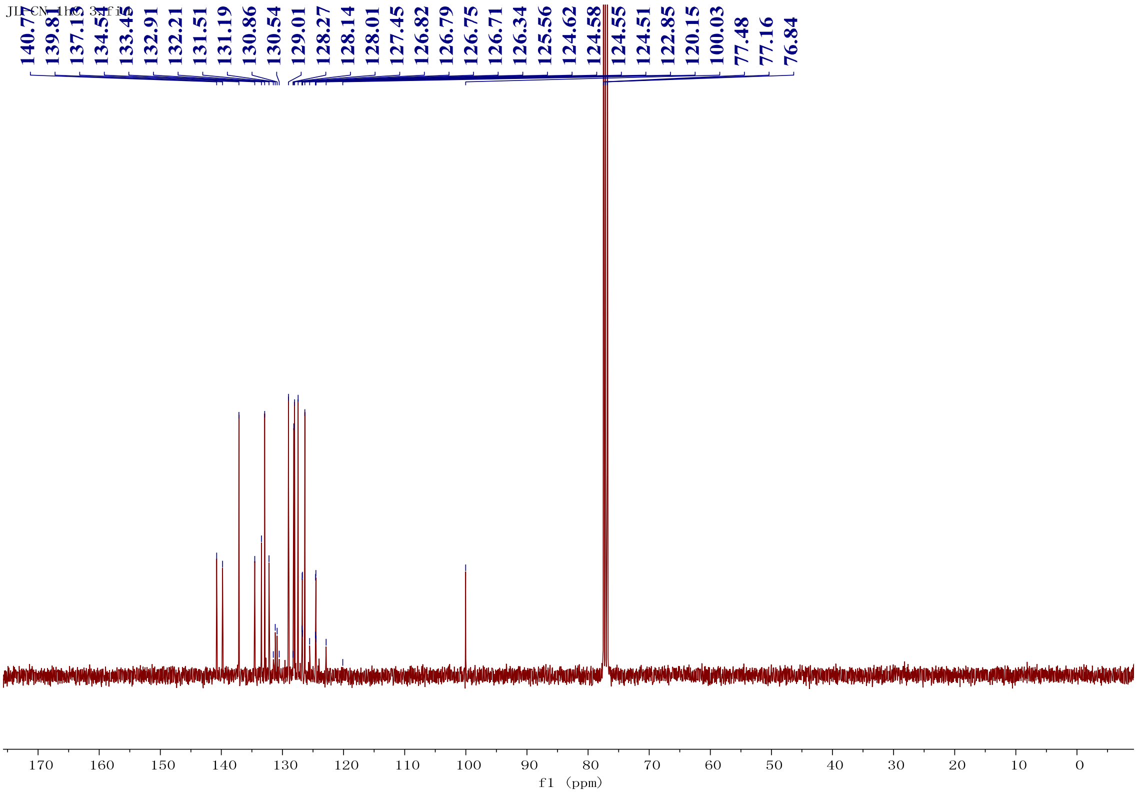

^{19}F NMR (376 MHz, Chloroform-*d*)

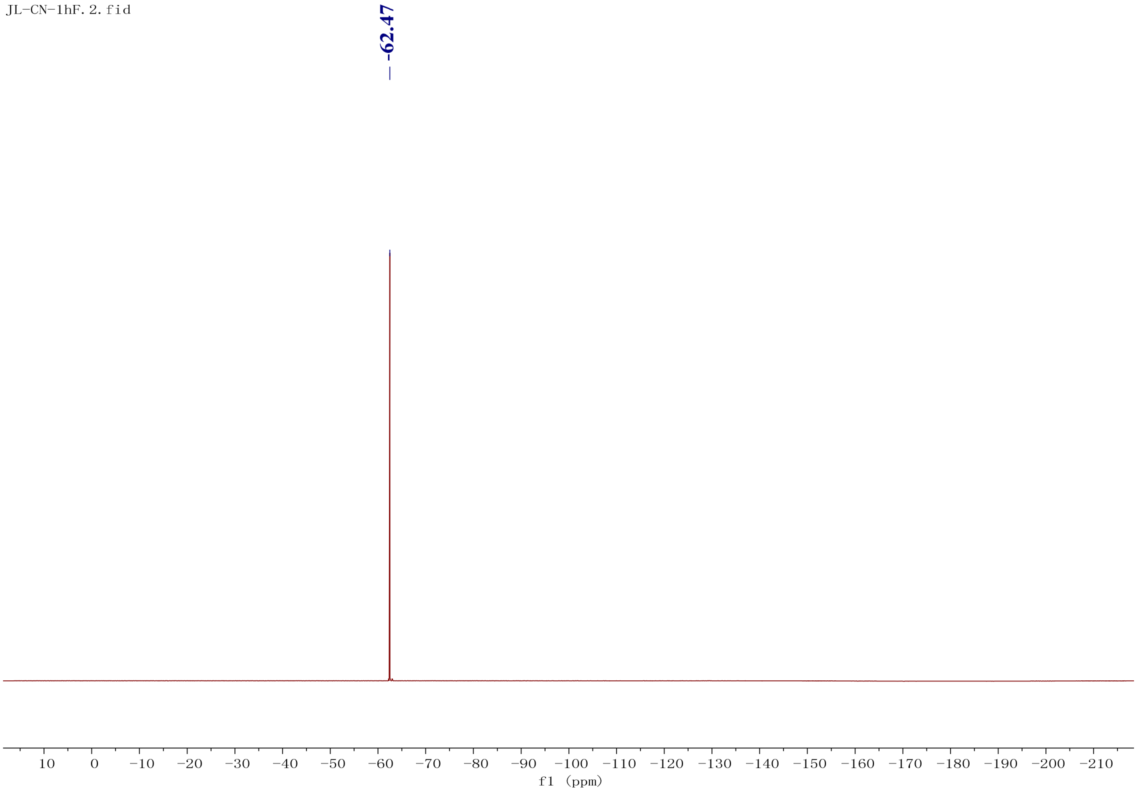

1j

^{1}H NMR (400 MHz, Chloroform-*d*)

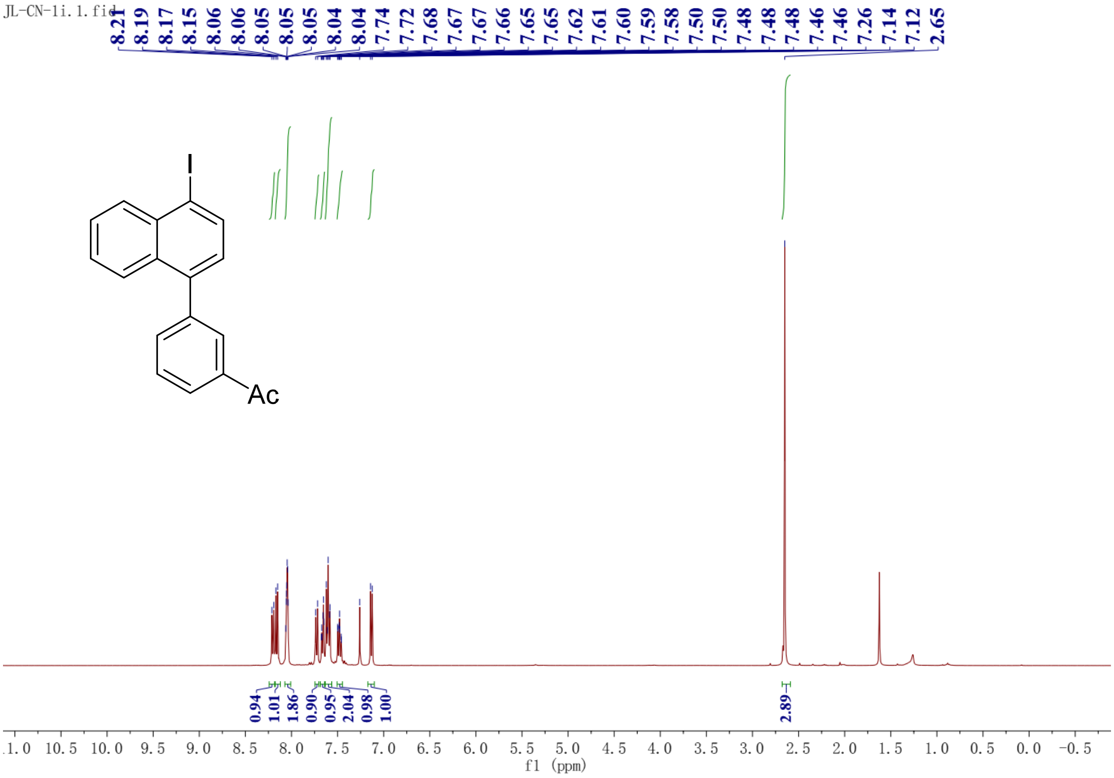

^{13}C NMR (101 MHz, Chloroform-*d*)

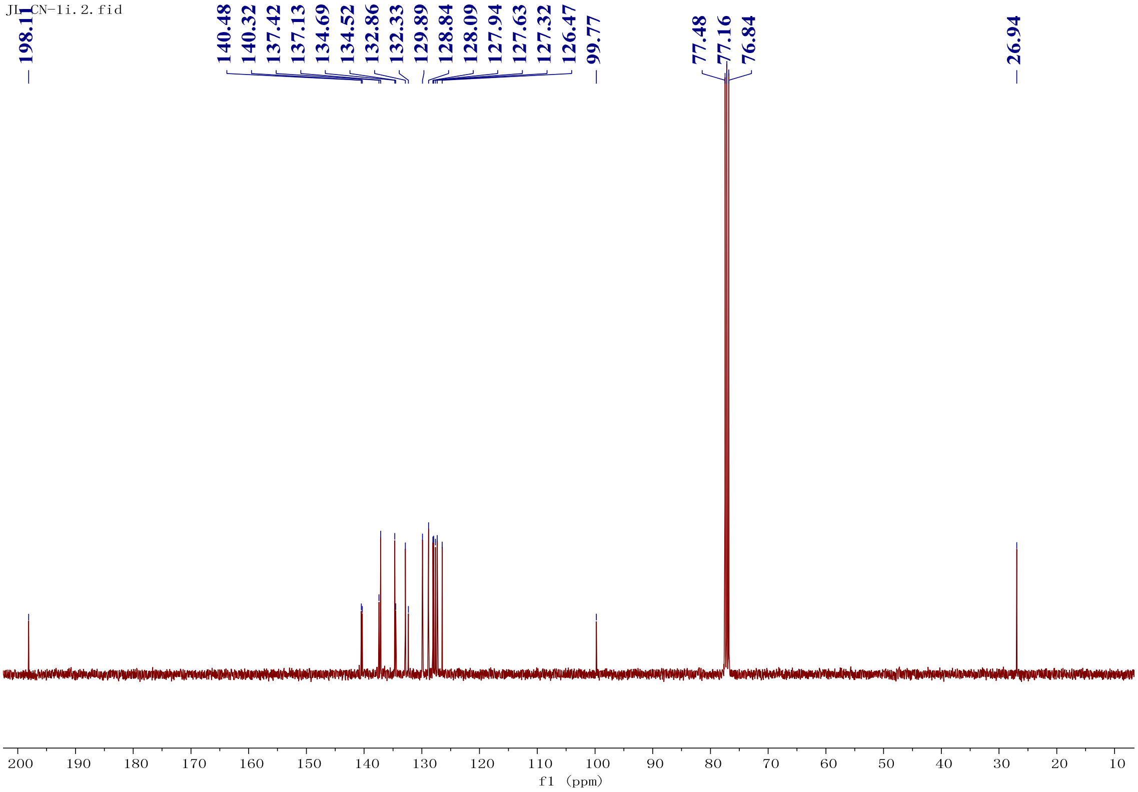

1k

^{1}H NMR (400 MHz, Chloroform-*d*)

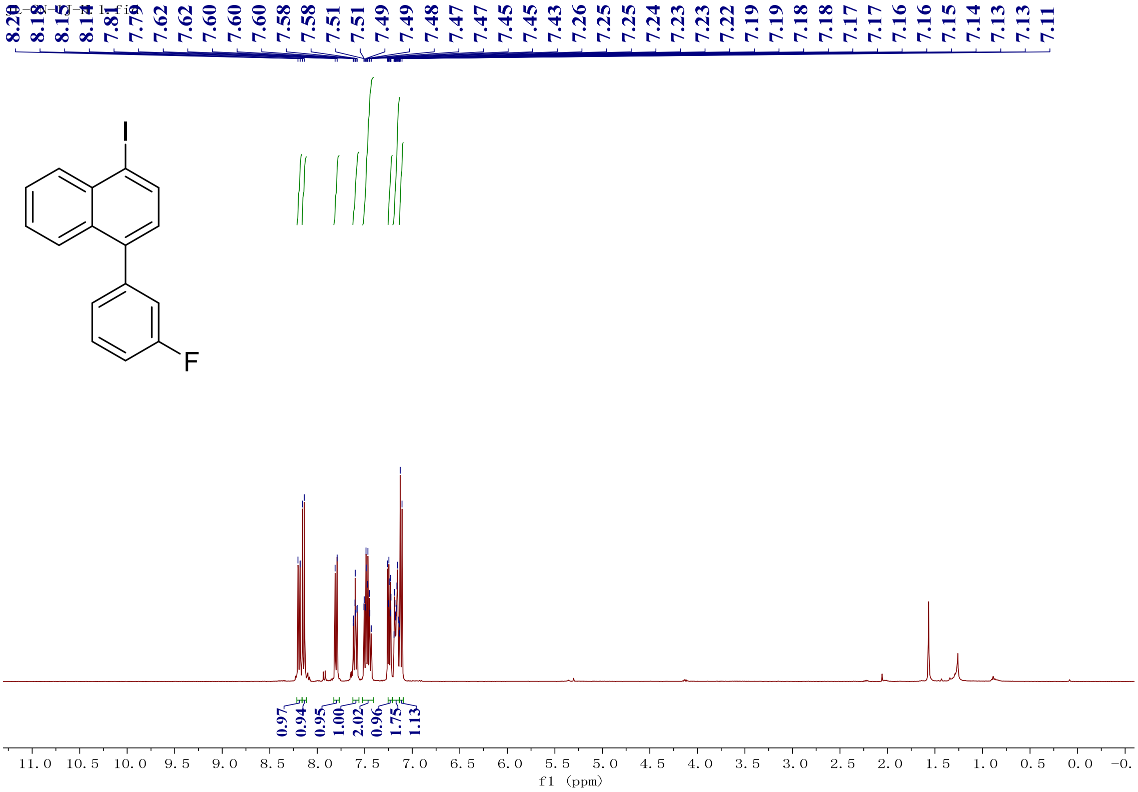

^{13}C NMR (101 MHz, Chloroform-*d*)

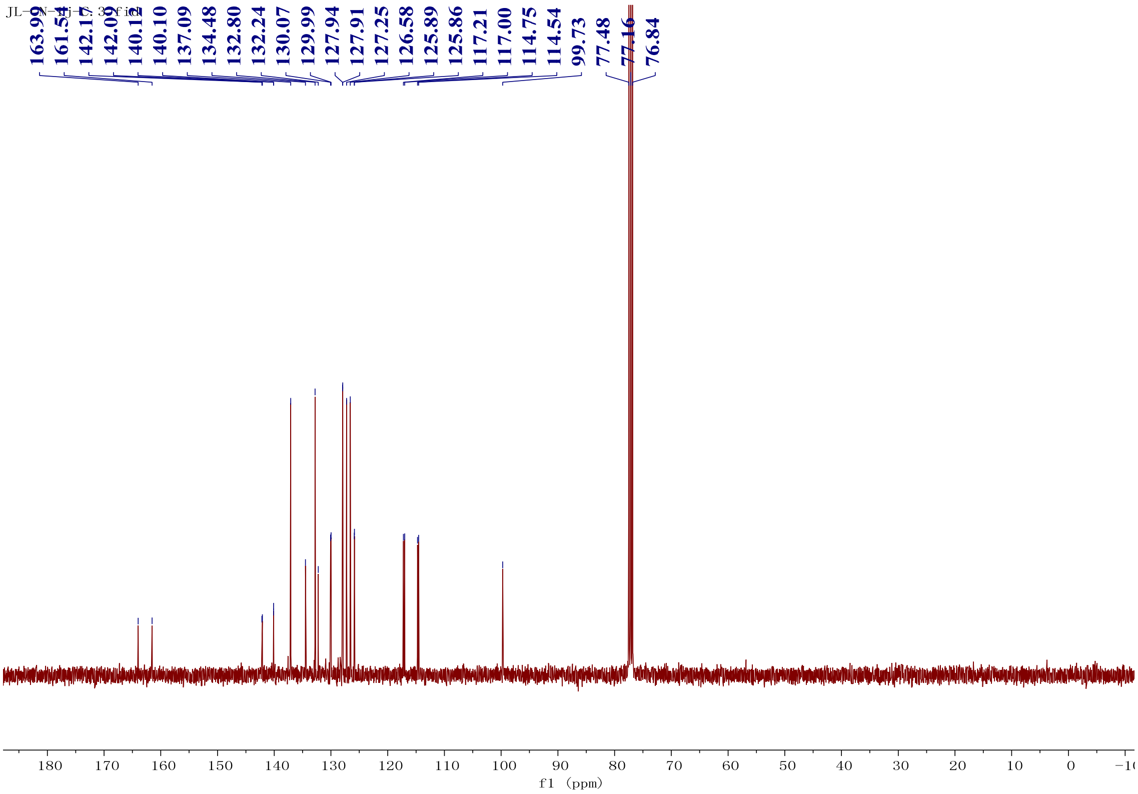

^{19}F NMR (376 MHz, Chloroform-*d*)

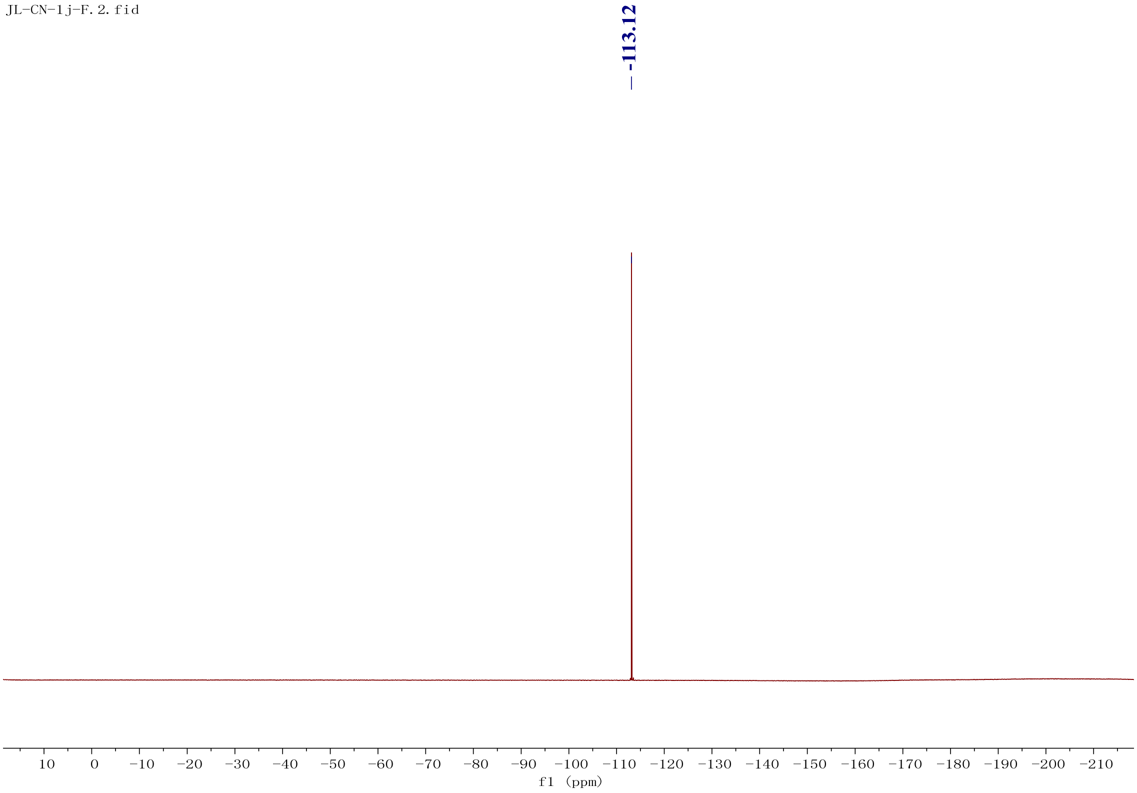

1l

^{1}H NMR (400 MHz, Chloroform-*d*)

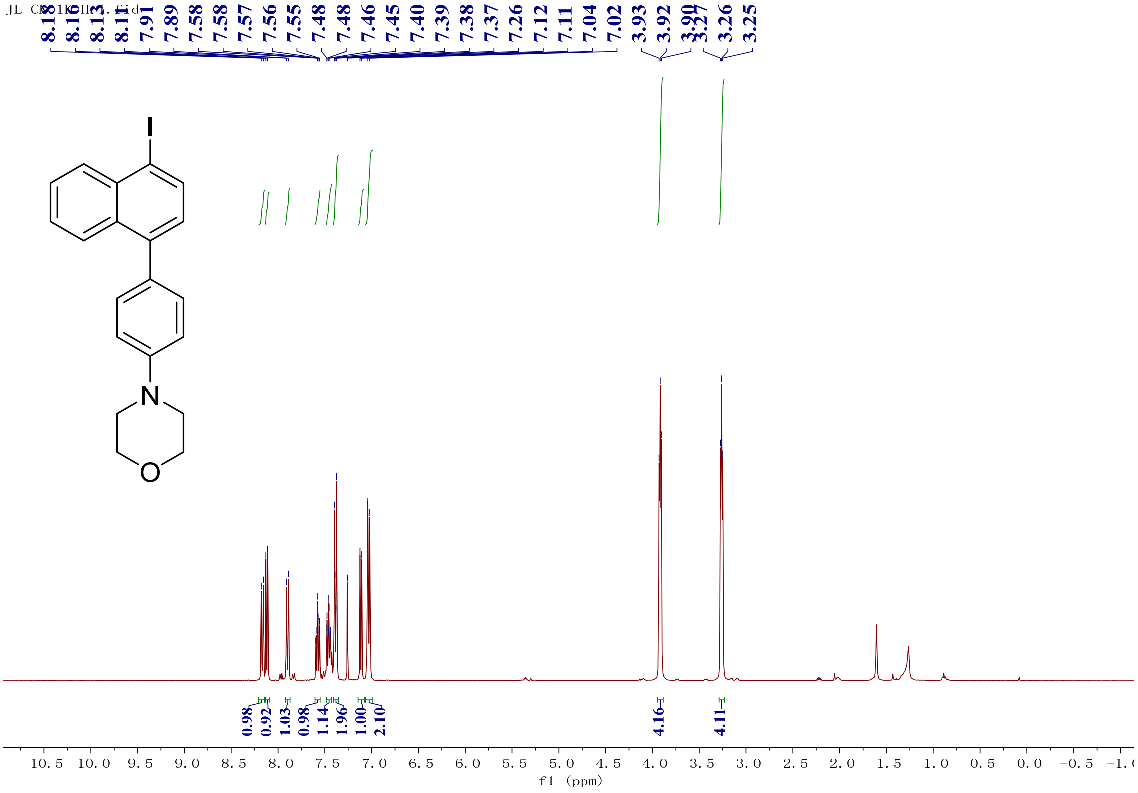

^{13}C NMR (101 MHz, Chloroform-*d*)

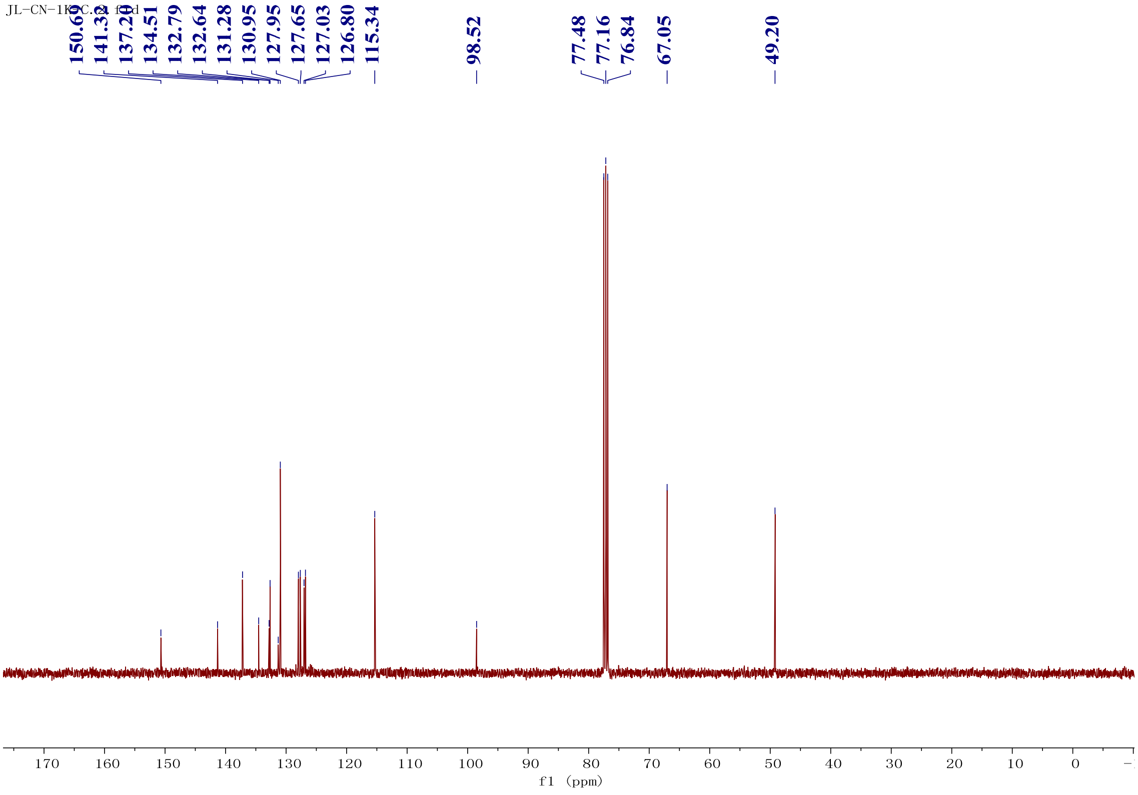

1m

^{1}H NMR (400 MHz, Chloroform-*d*)

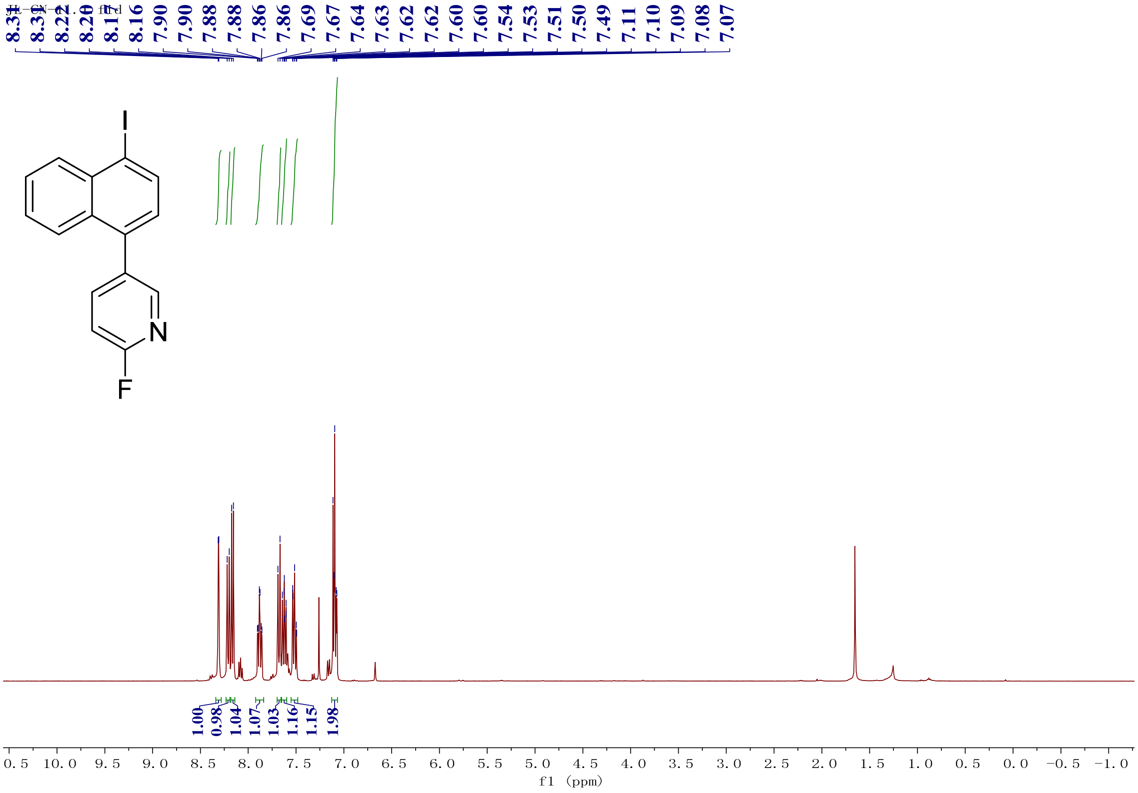

^{13}C NMR (101 MHz, Chloroform-*d*)

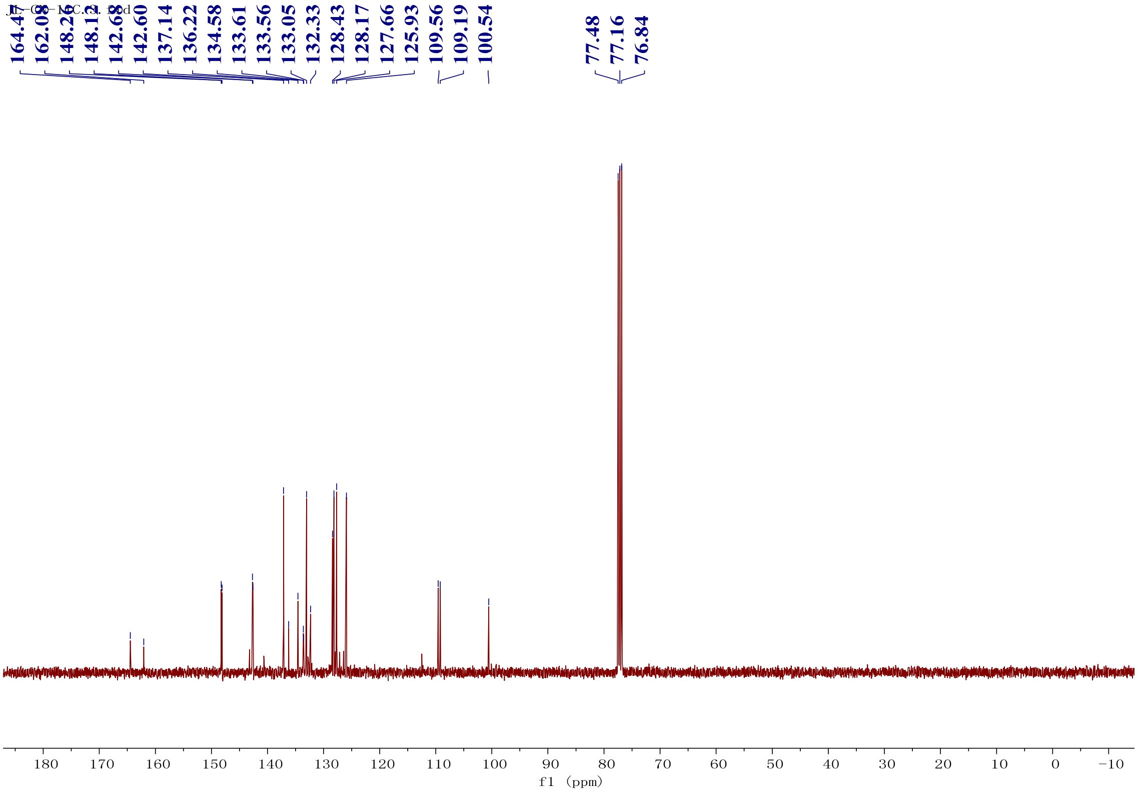

^{19}F NMR (376 MHz, Chloroform-*d*)

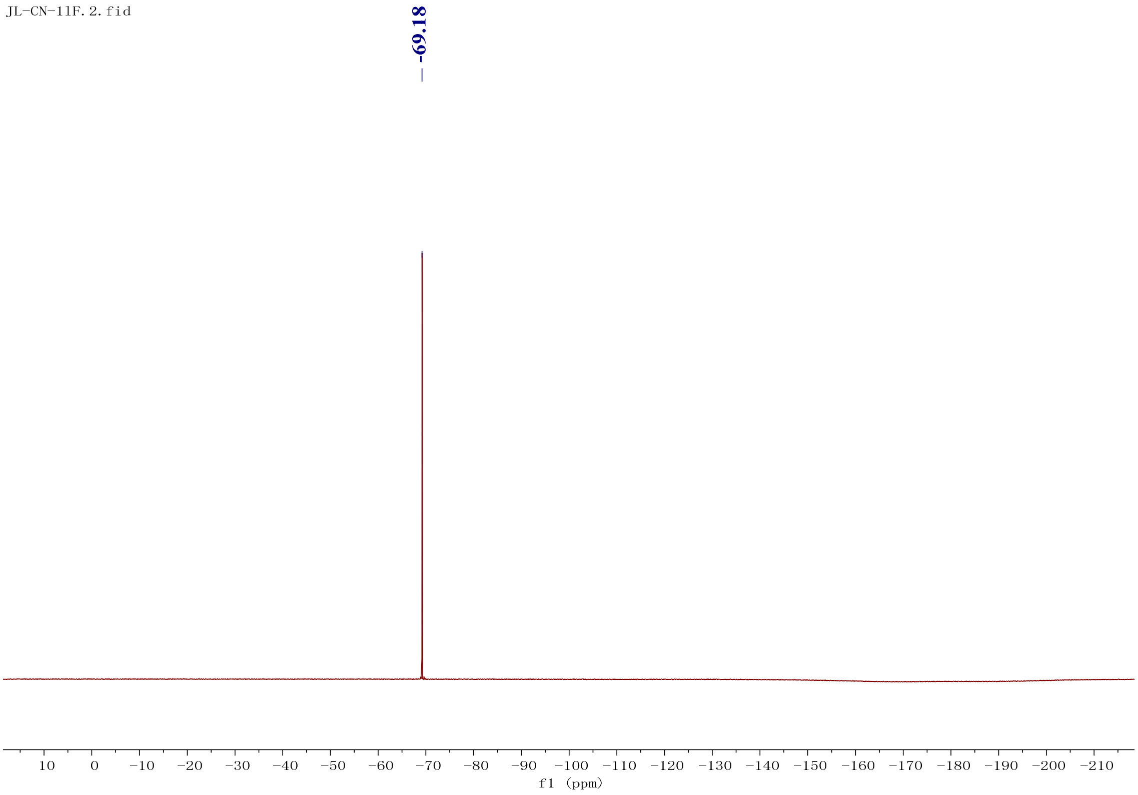

1n

^{1}H NMR (400 MHz, Chloroform-*d*)

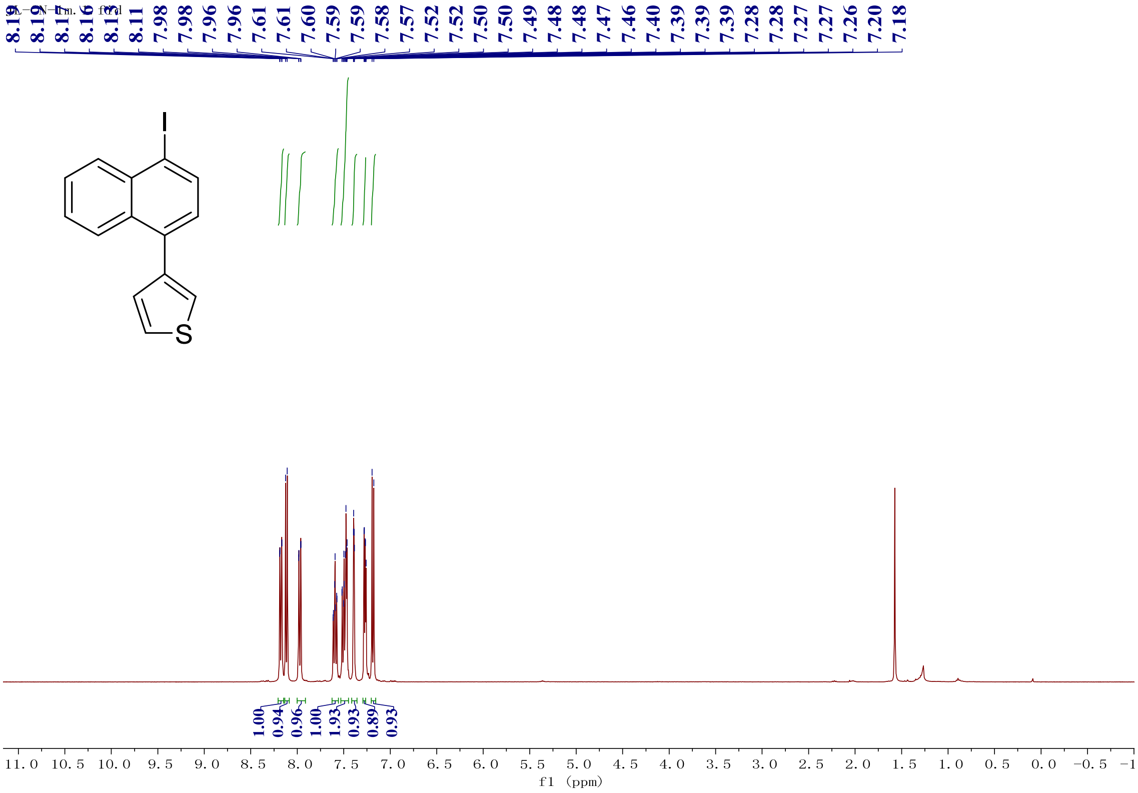

^{13}C NMR (101 MHz, Chloroform-*d*)

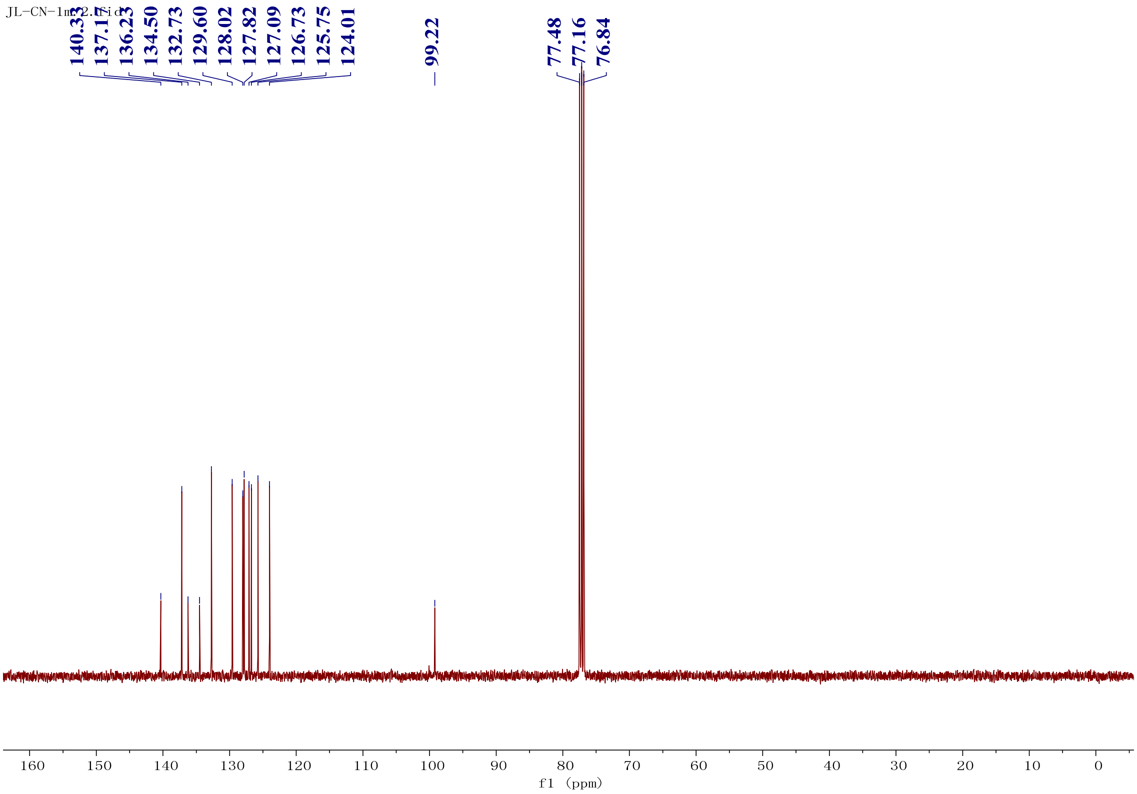

1o

^{1}H NMR (400 MHz, Chloroform-*d*)

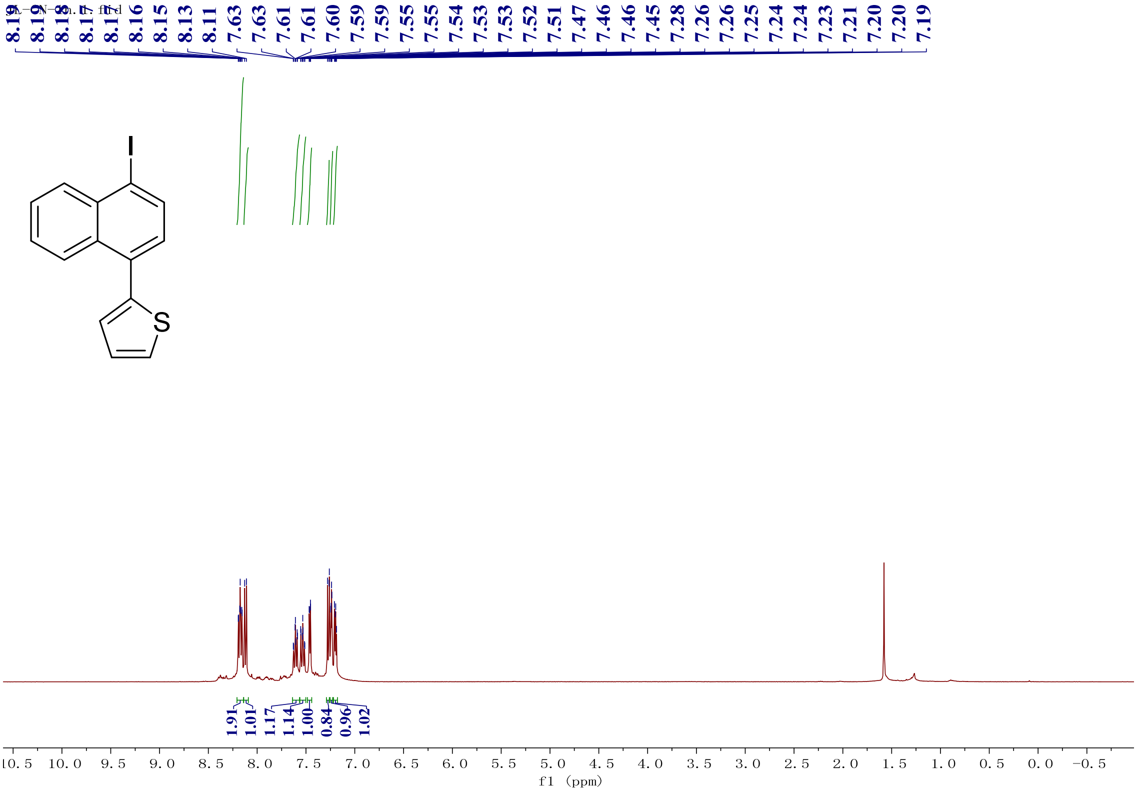

^{13}C NMR (101 MHz, Chloroform-*d*)

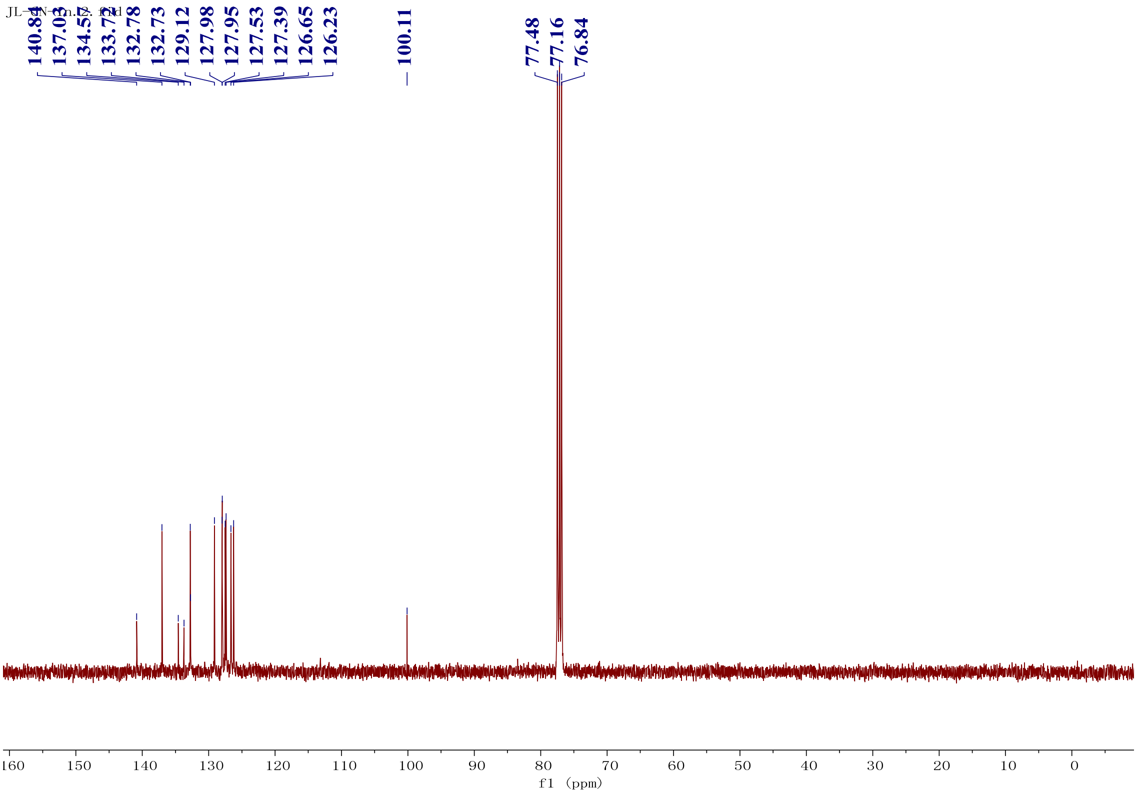

1p

^{1}H NMR (400 MHz, Chloroform-*d*)

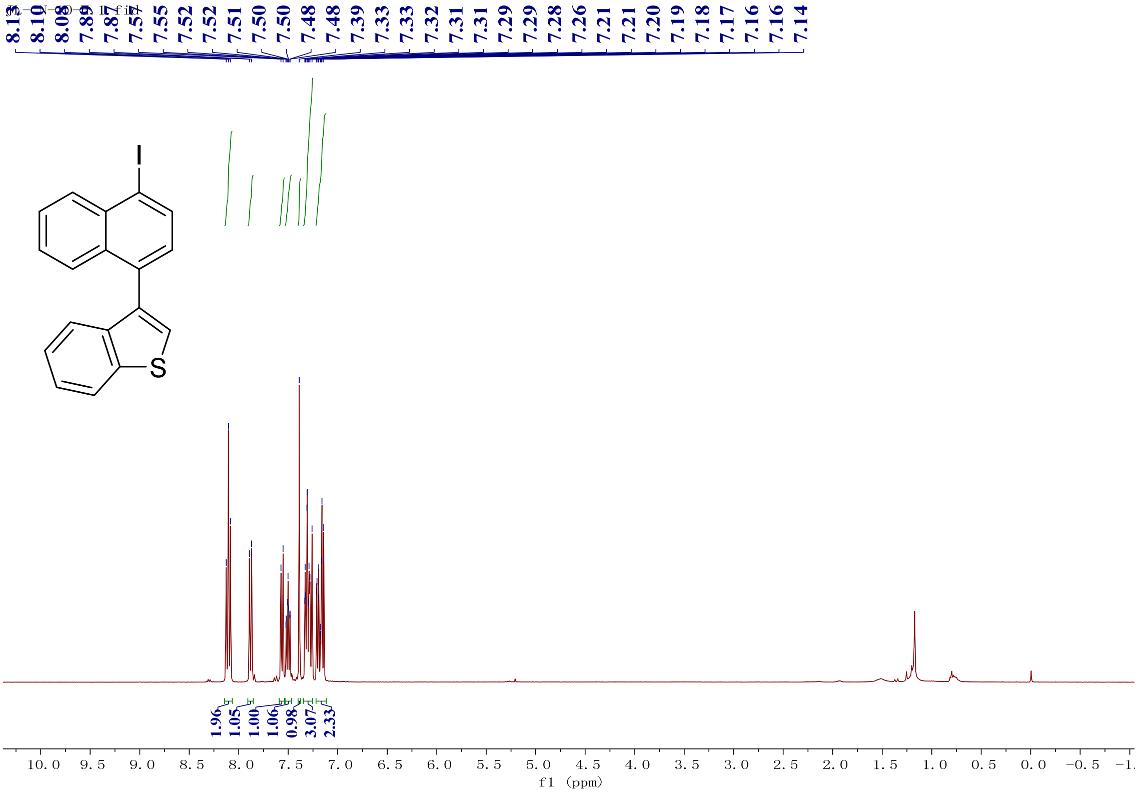

^{13}C NMR (101 MHz, Chloroform-*d*)

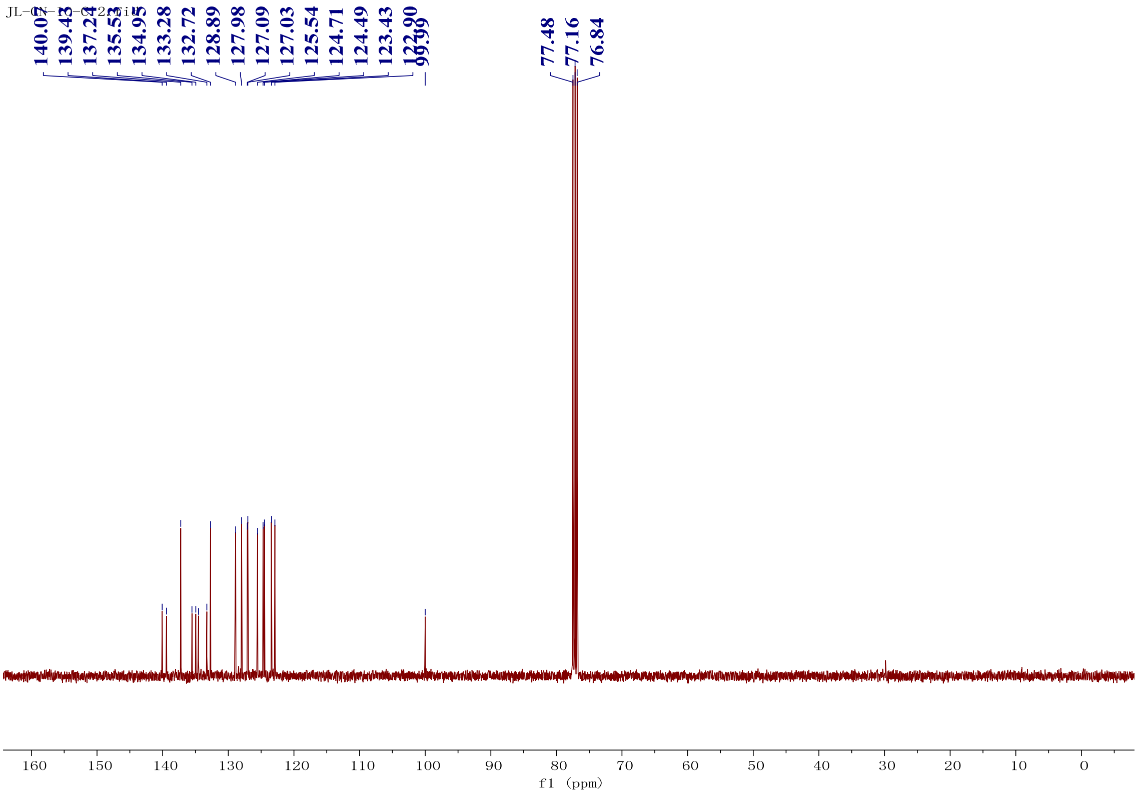

1q

^{1}H NMR (400 MHz, Chloroform-*d*)

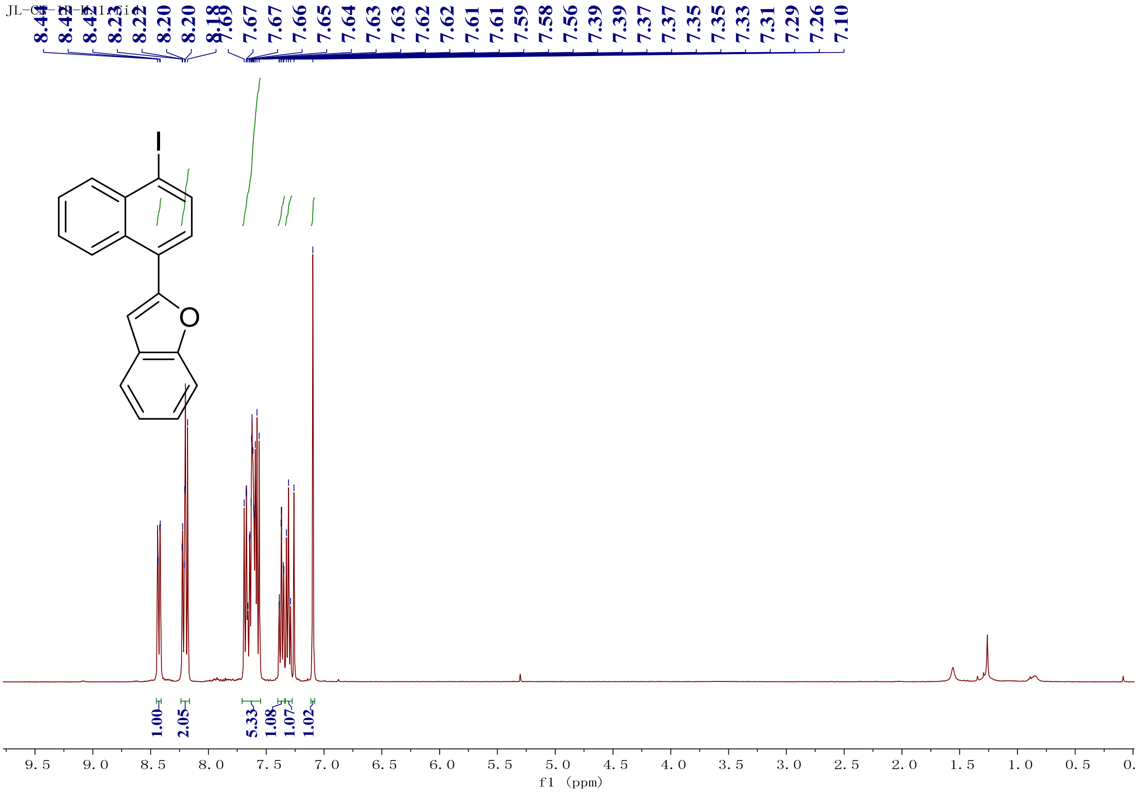

^{13}C NMR (101 MHz, Chloroform-*d*)

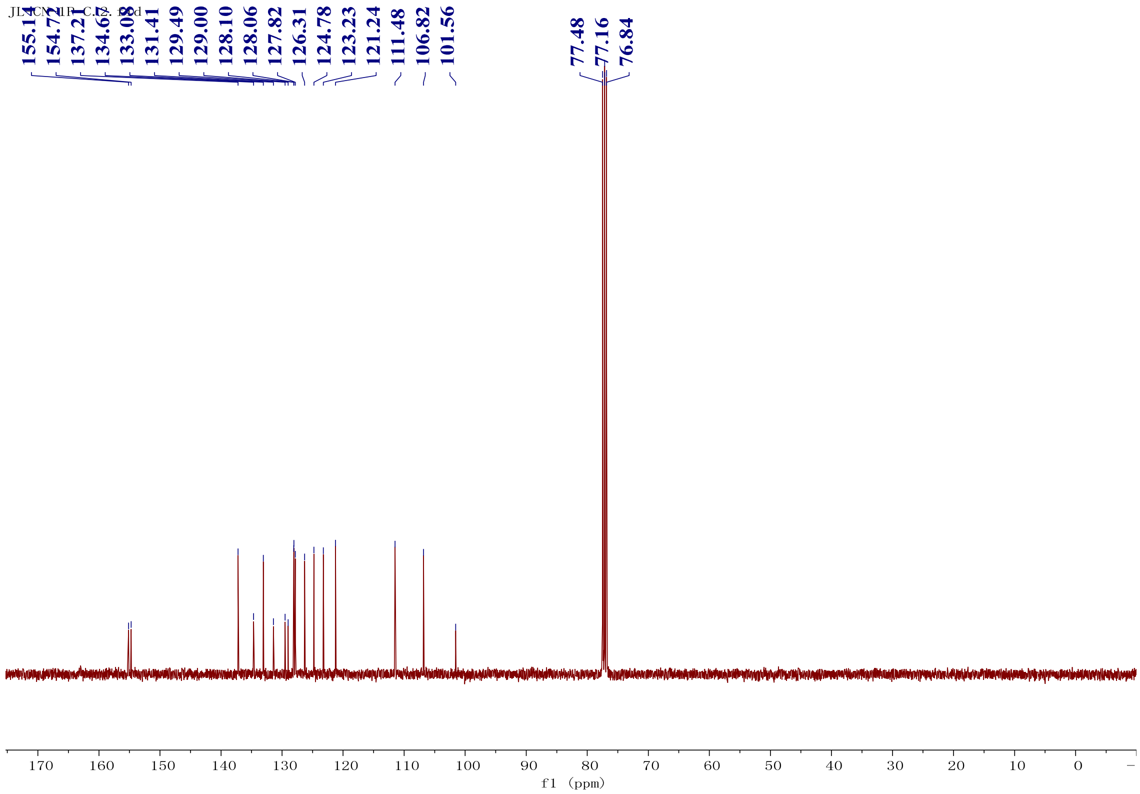

1r

^{1}H NMR (400 MHz, Chloroform-*d*)

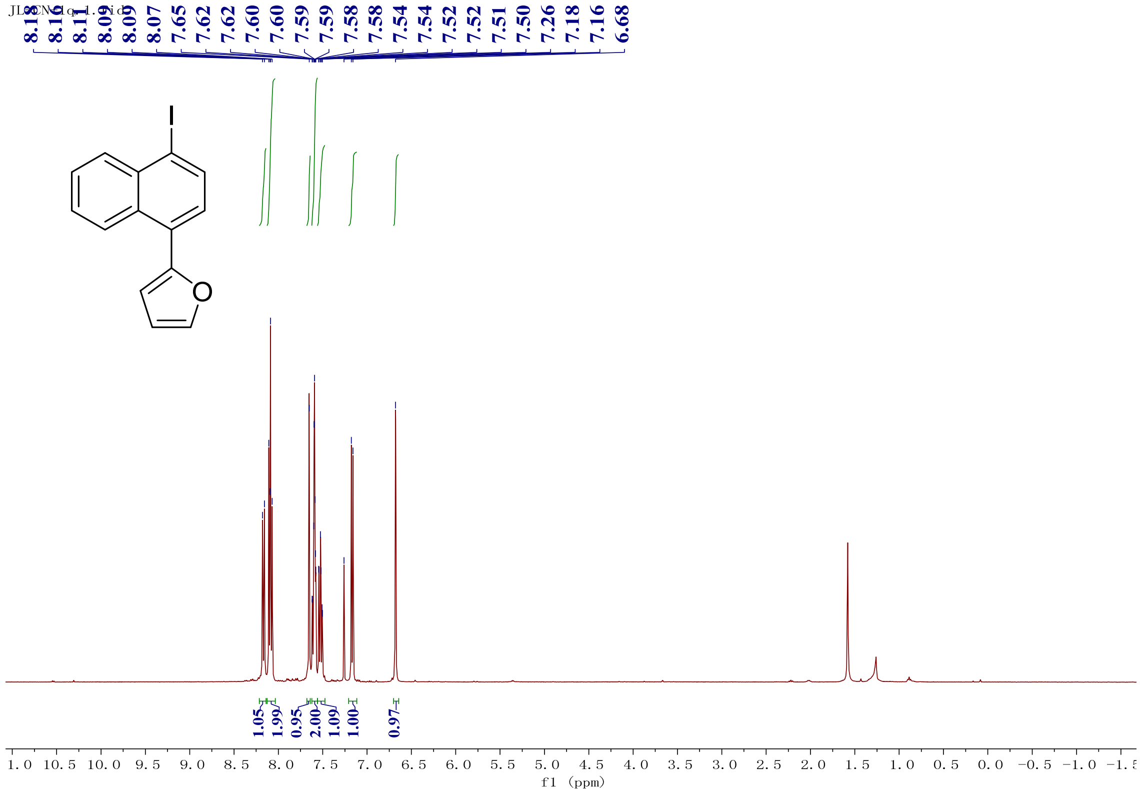

^{13}C NMR (101 MHz, Chloroform-*d*)

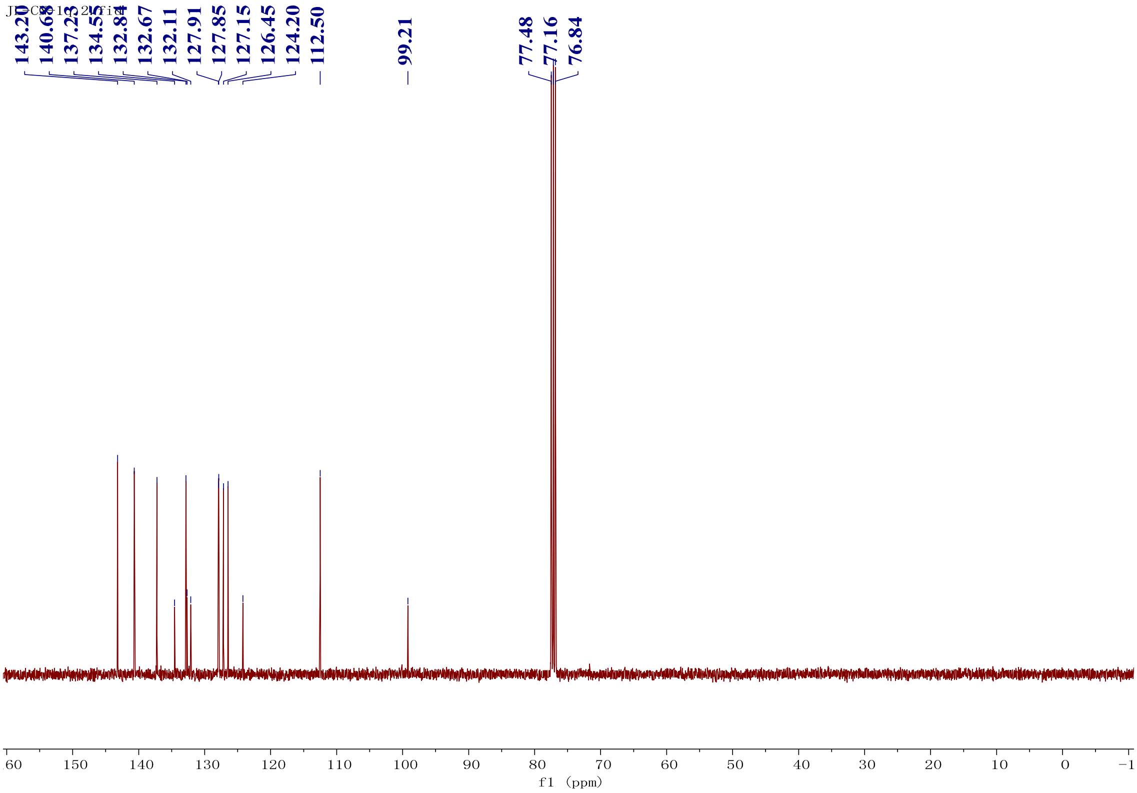

1s

^{1}H NMR (400 MHz, Chloroform-*d*)

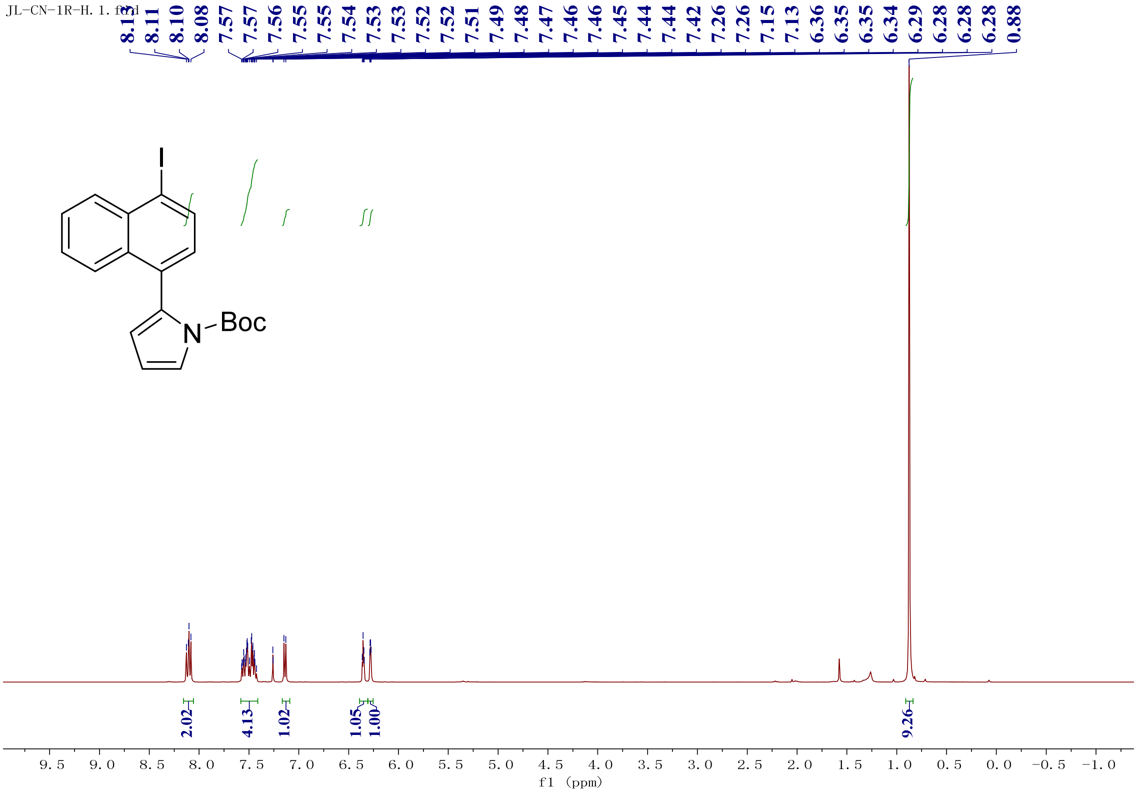

^{13}C NMR (101 MHz, Chloroform-*d*)

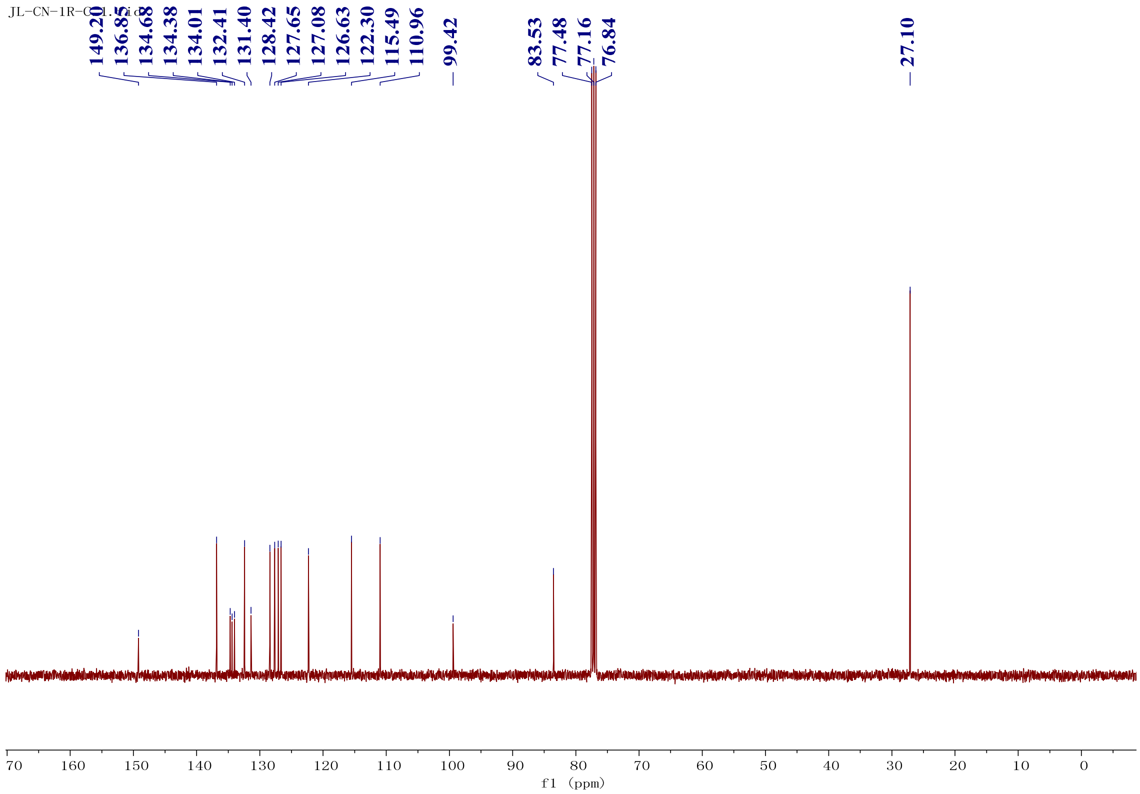

1t

^{1}H NMR (400 MHz, Chloroform-*d*)

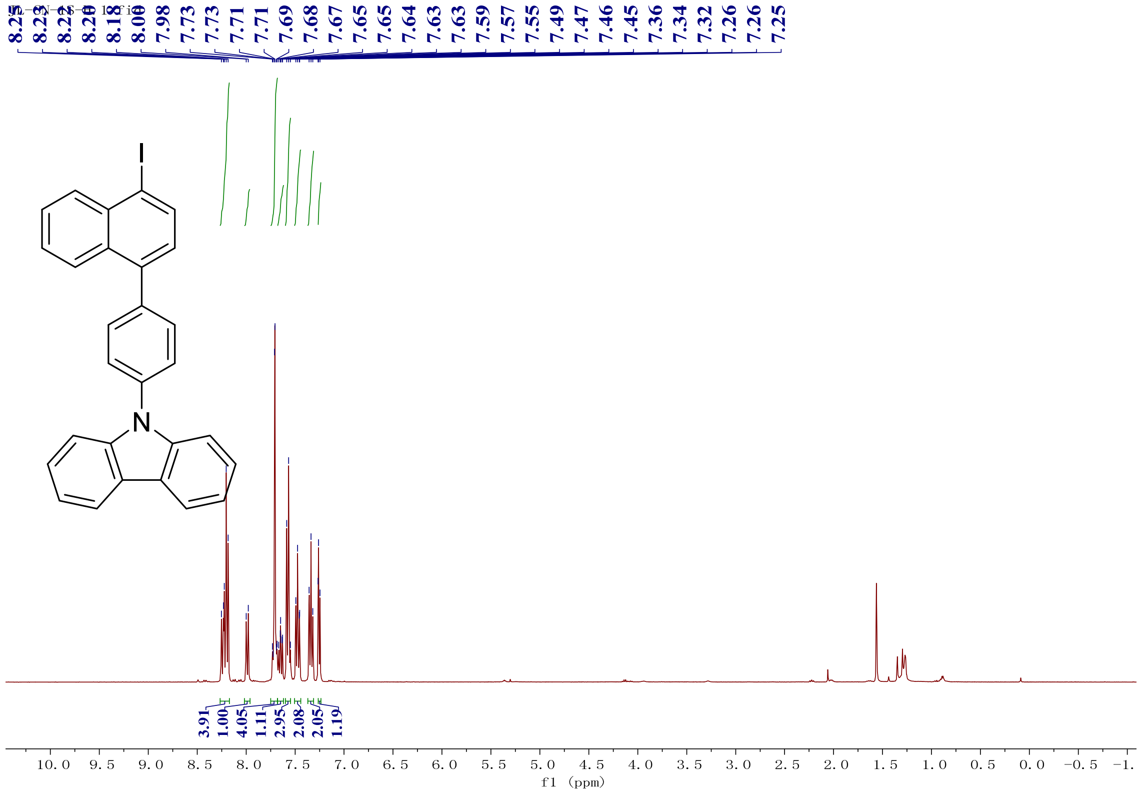

^{13}C NMR (101 MHz, Chloroform-*d*)

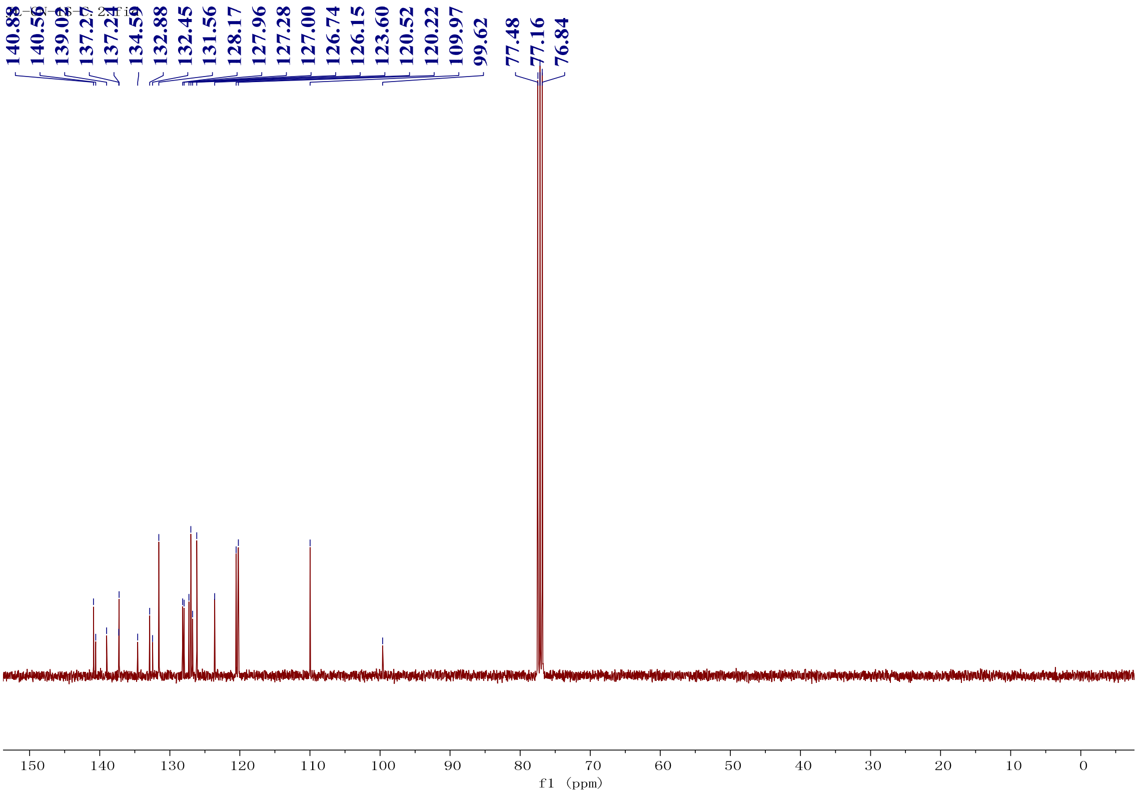

1u

^{1}H NMR (400 MHz, Chloroform-*d*)

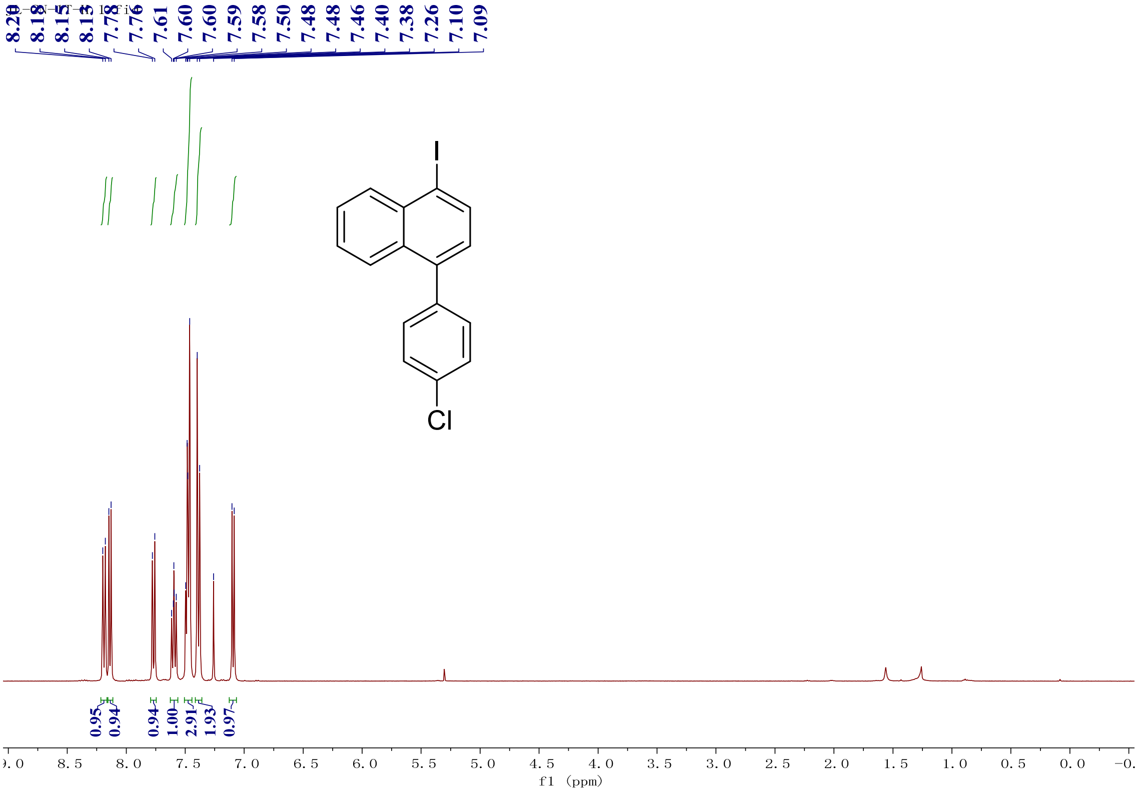

^{13}C NMR (101 MHz, Chloroform-*d*)

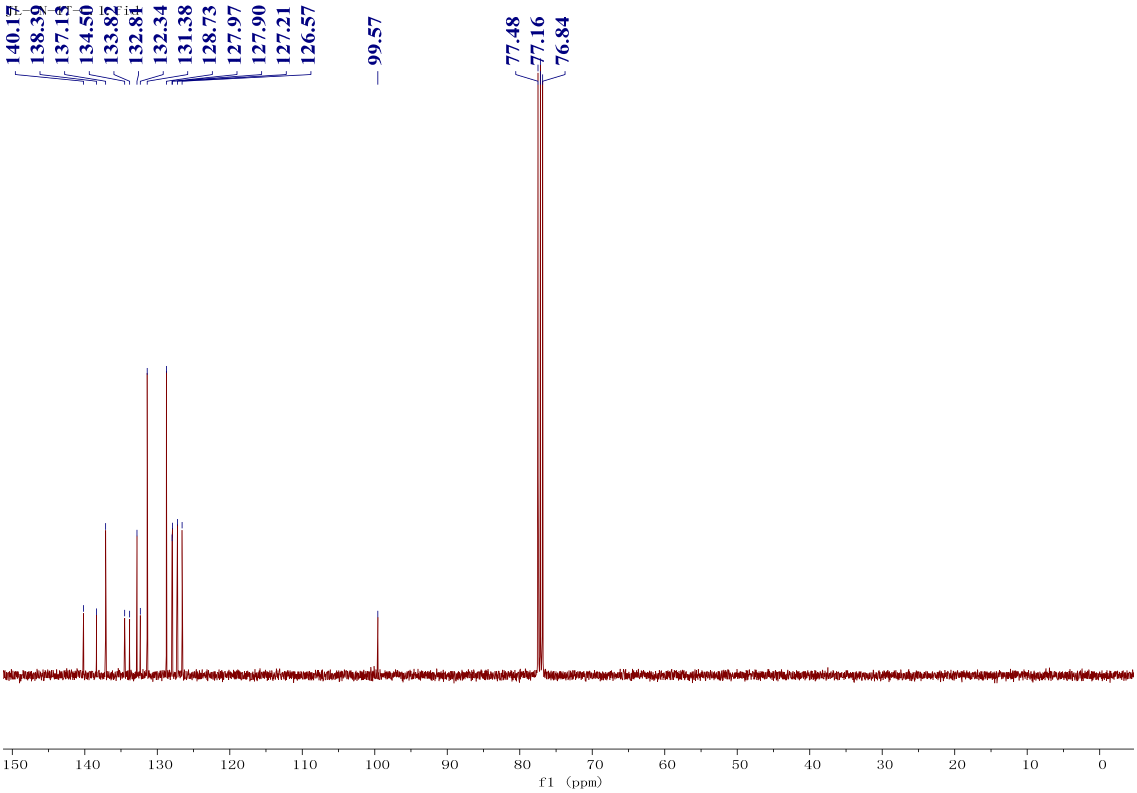

1v

^{1}H NMR (400 MHz, Chloroform-*d*)

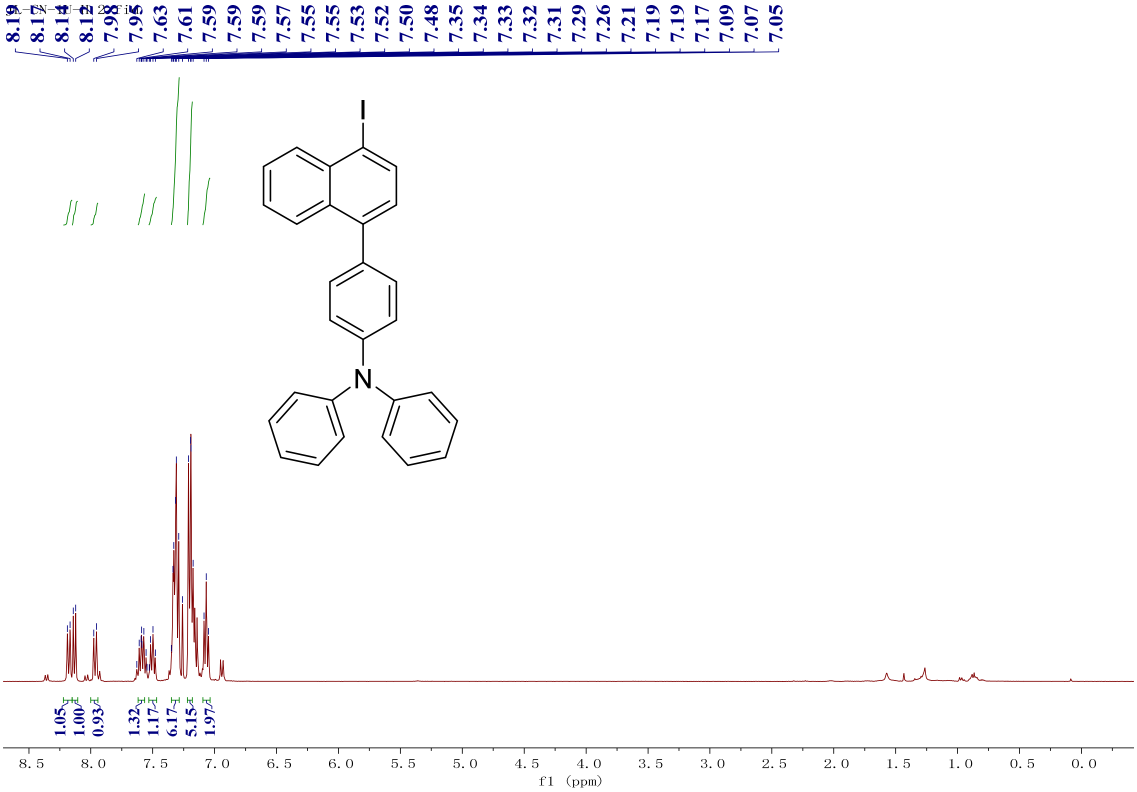

^{13}C NMR (101 MHz, Chloroform-*d*)

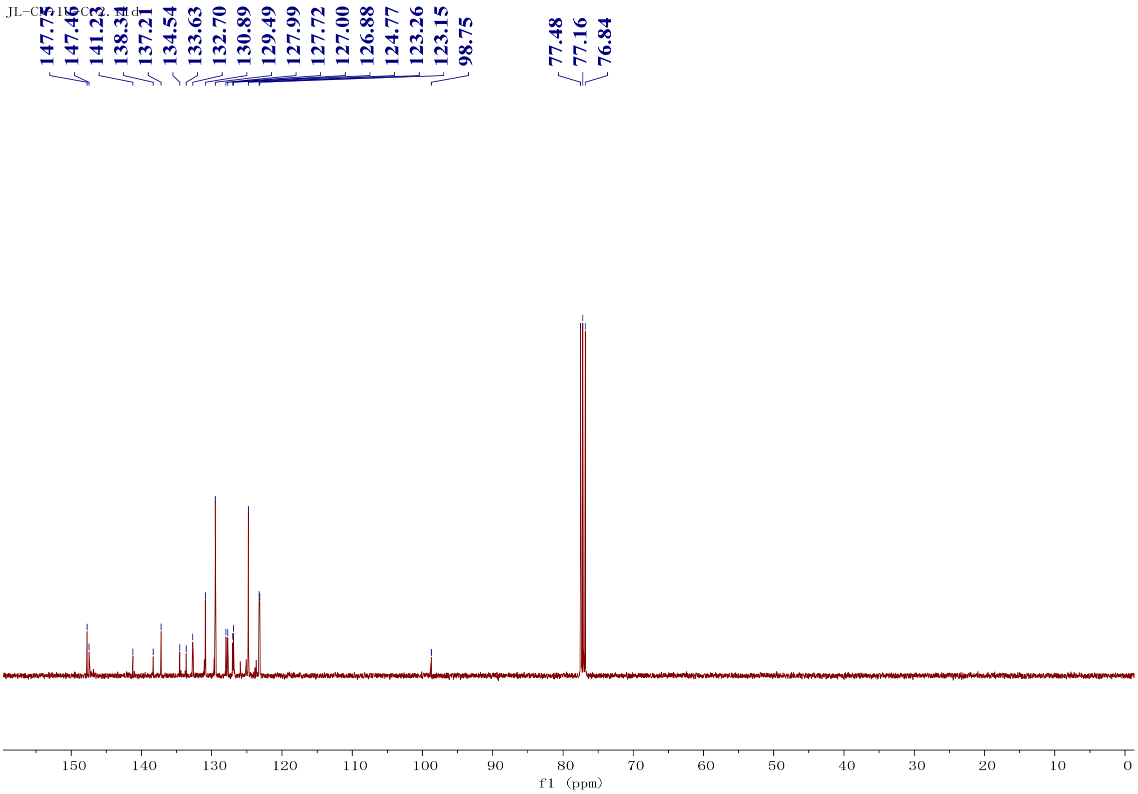

2a

^{1}H NMR (400 MHz, Chloroform-*d*)

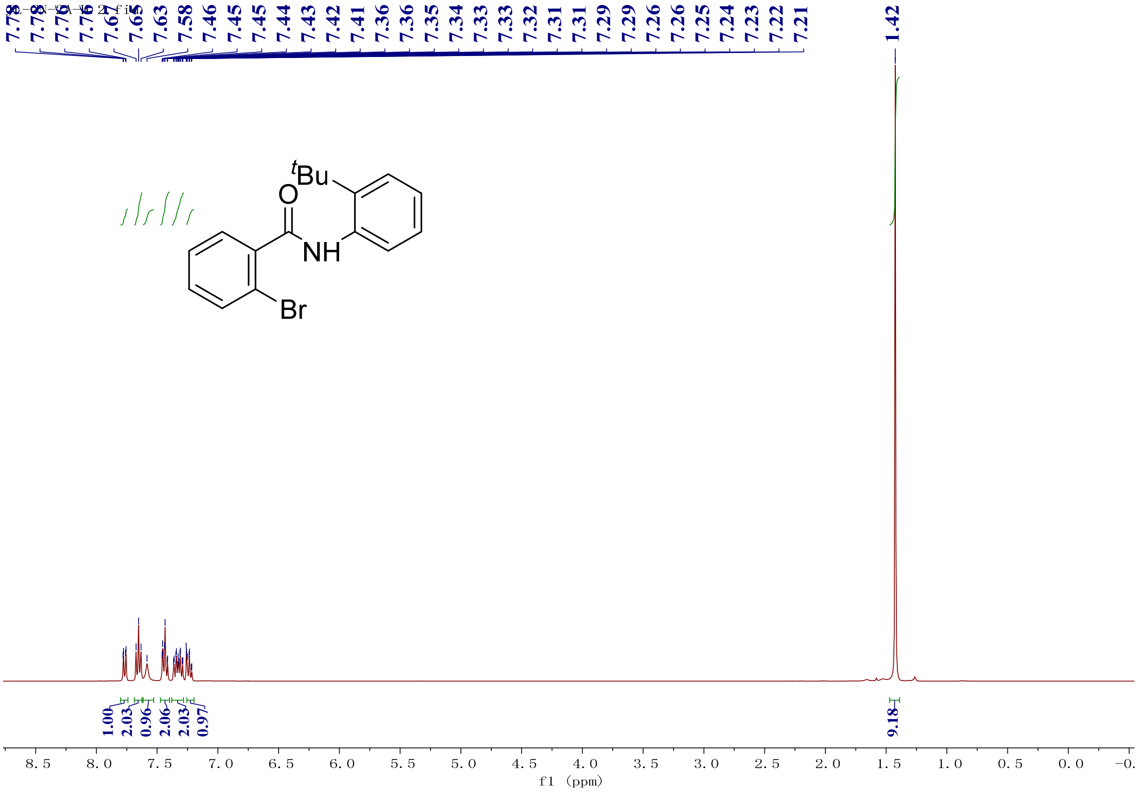

^{13}C NMR (101 MHz, Chloroform-*d*)

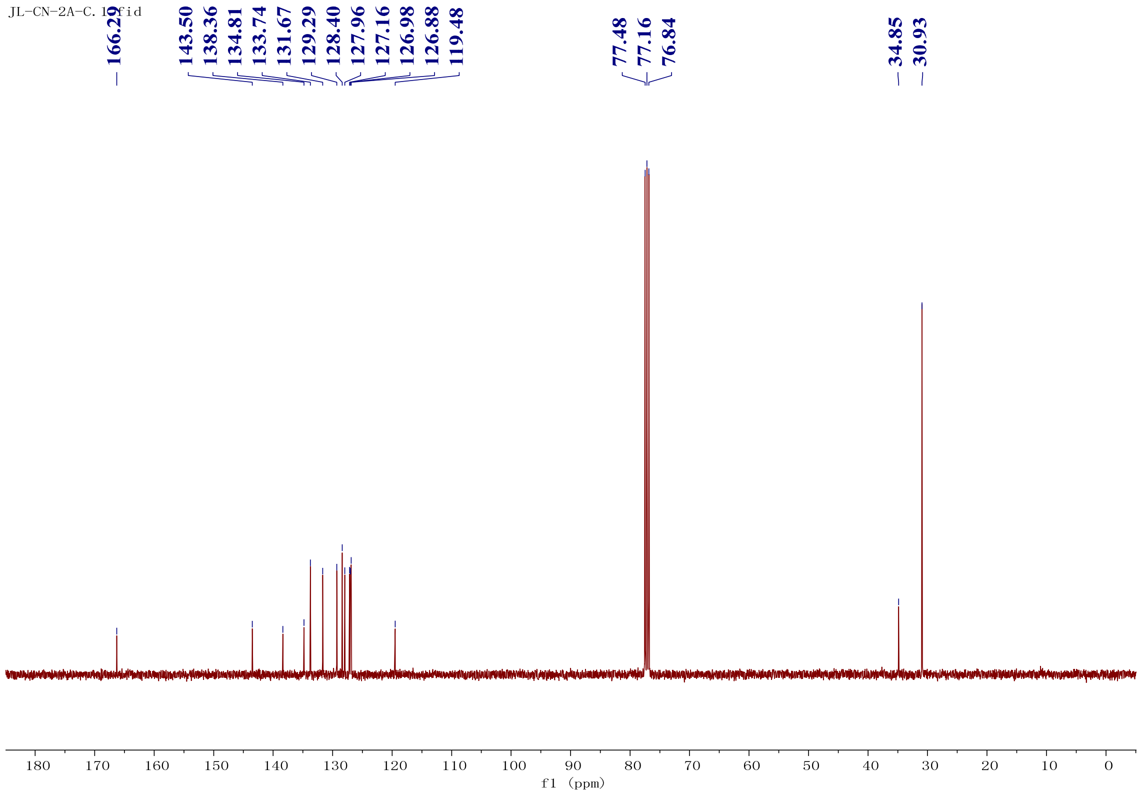

2b

^{1}H NMR (400 MHz, Chloroform-*d*)

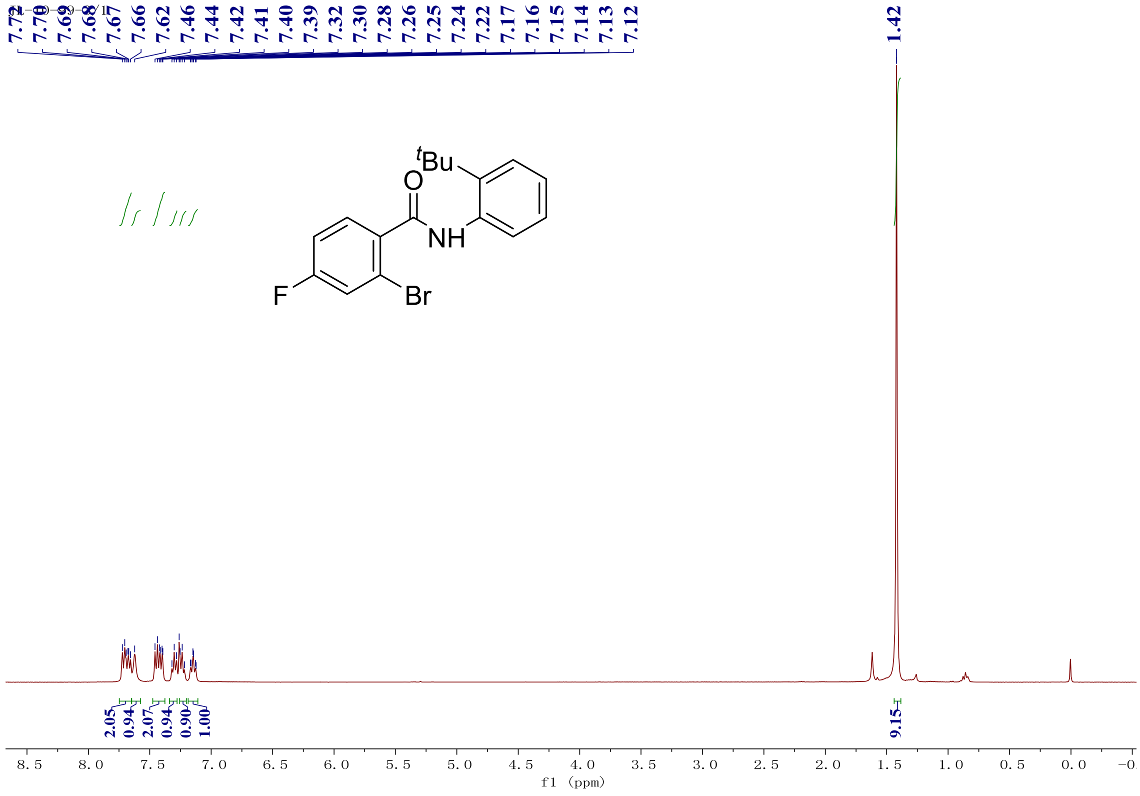

^{13}C NMR (101 MHz, Chloroform-*d*)

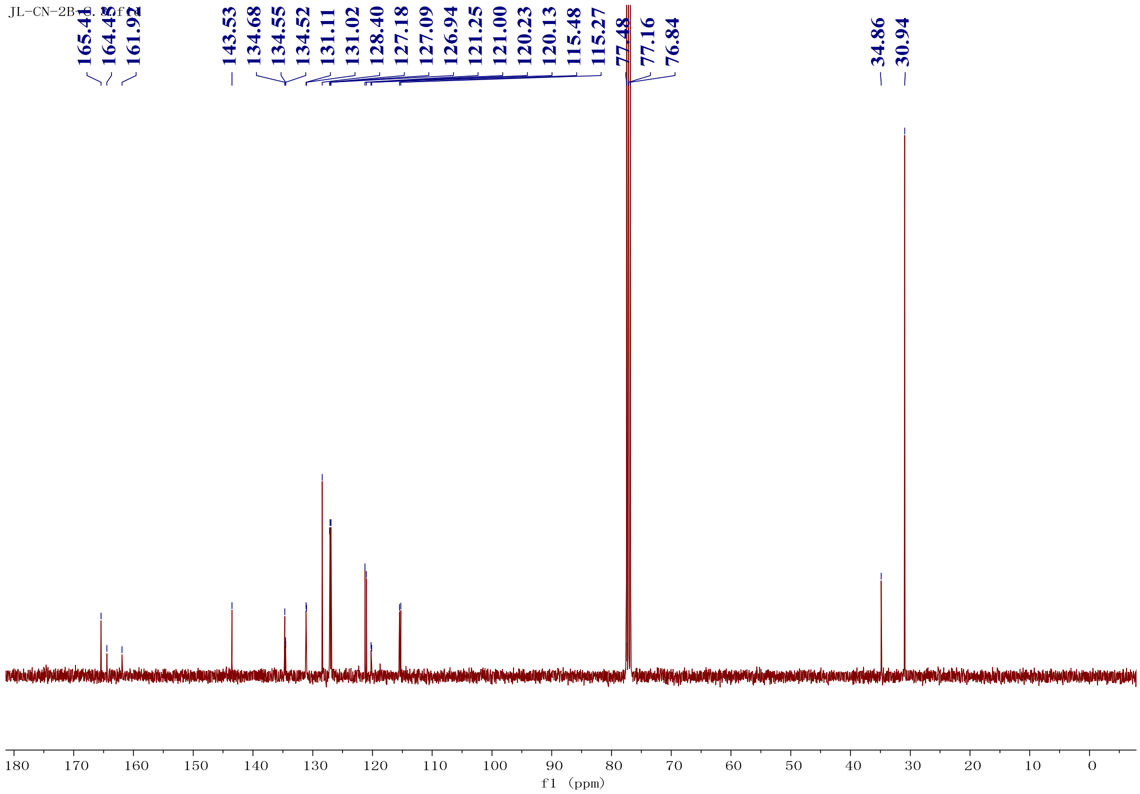

^{19}F NMR (376 MHz, Chloroform-*d*)

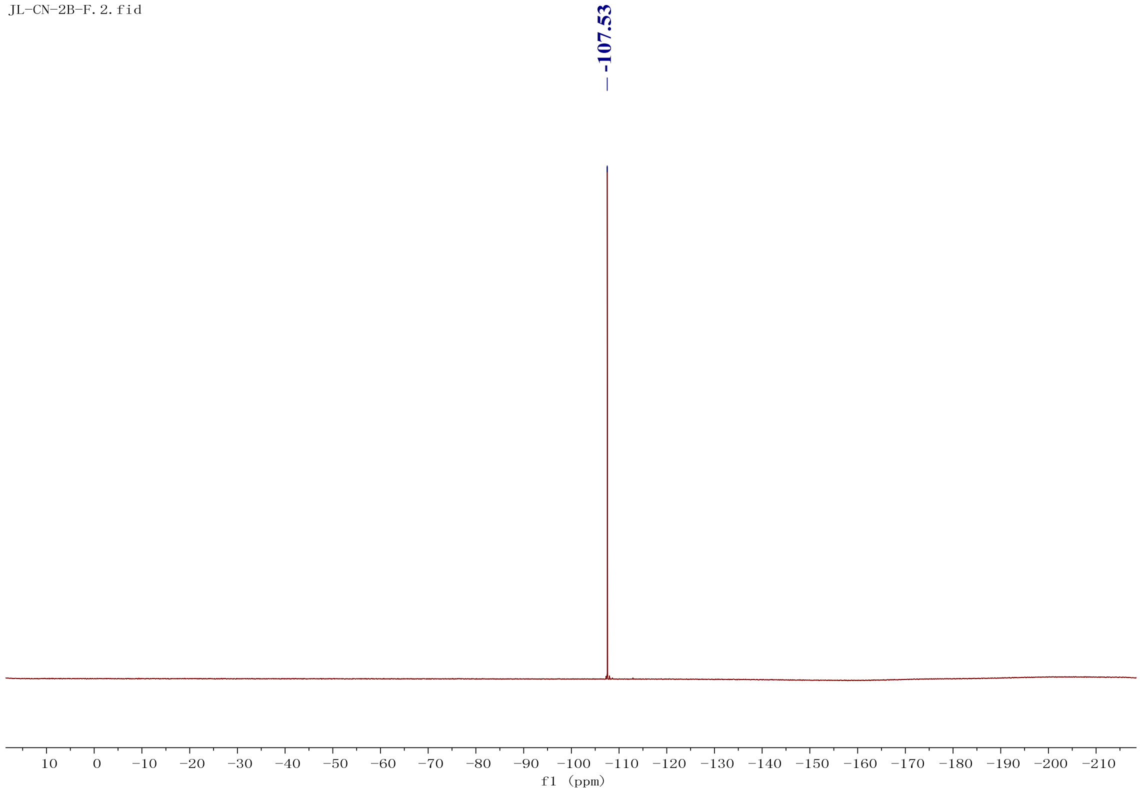

2c

^{1}H NMR (400 MHz, Chloroform-*d*)

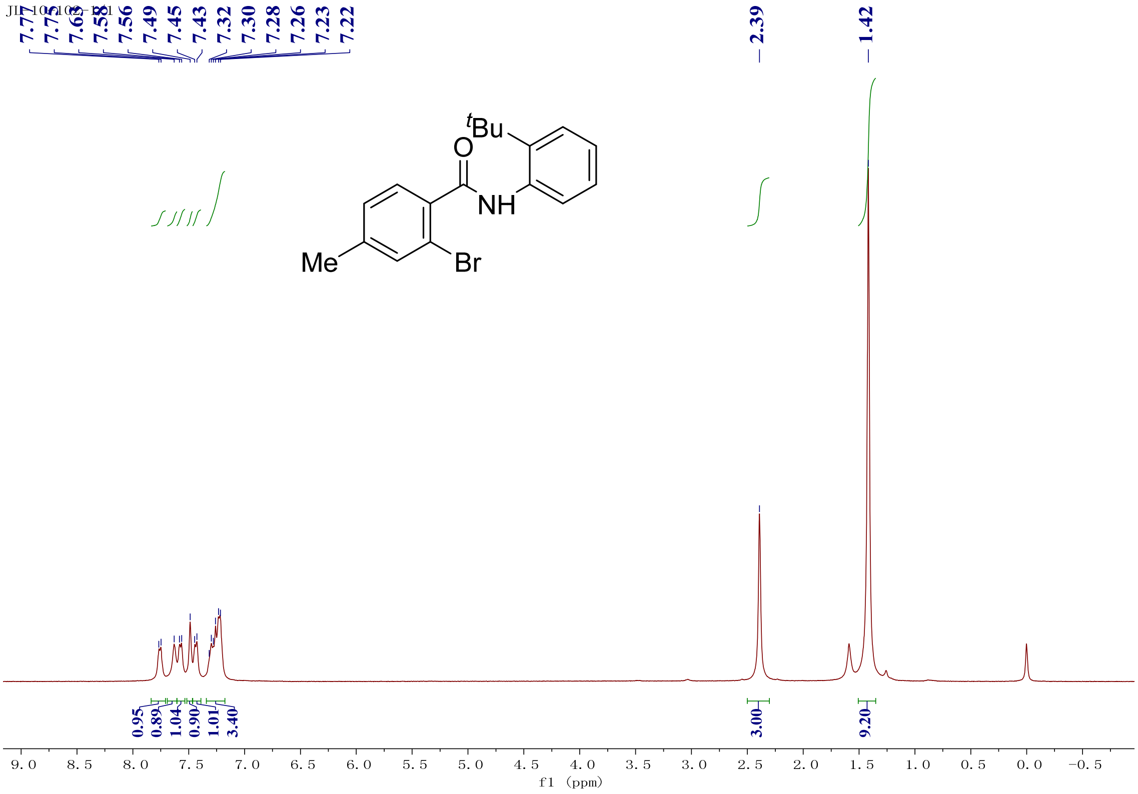

^{13}C NMR (101 MHz, Chloroform-*d*)

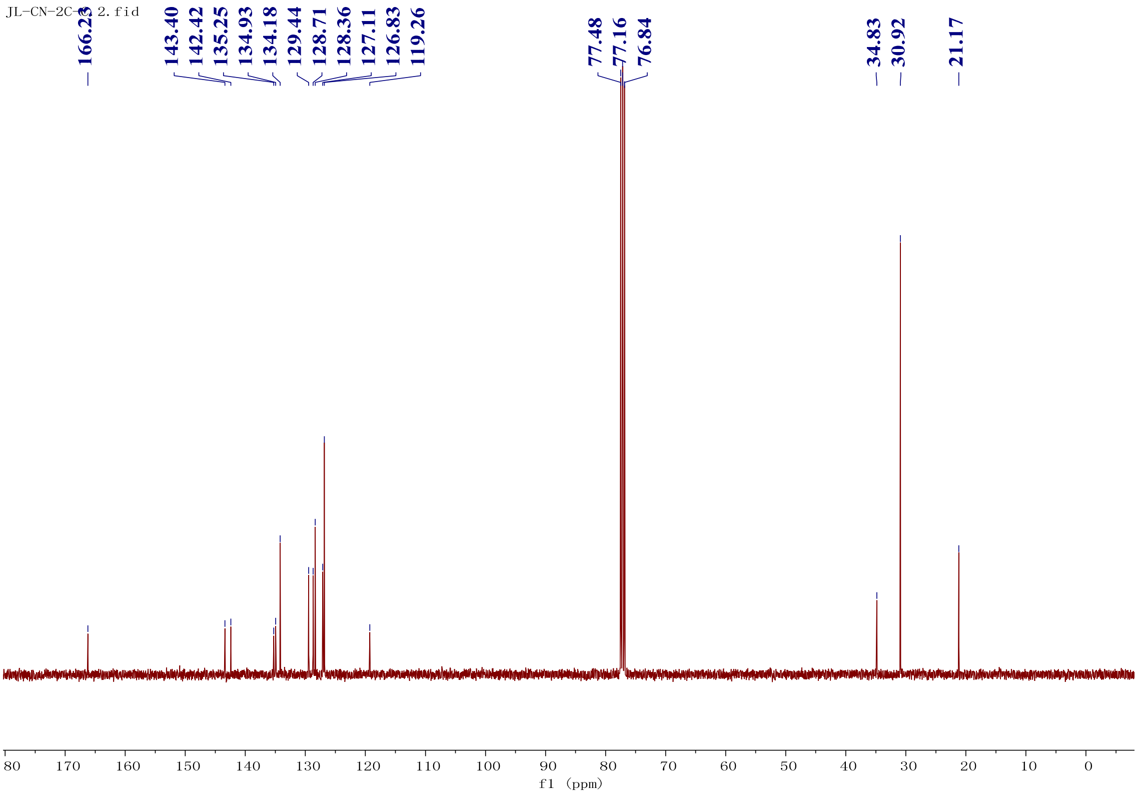

2d

^{1}H NMR (400 MHz, Chloroform-*d*)

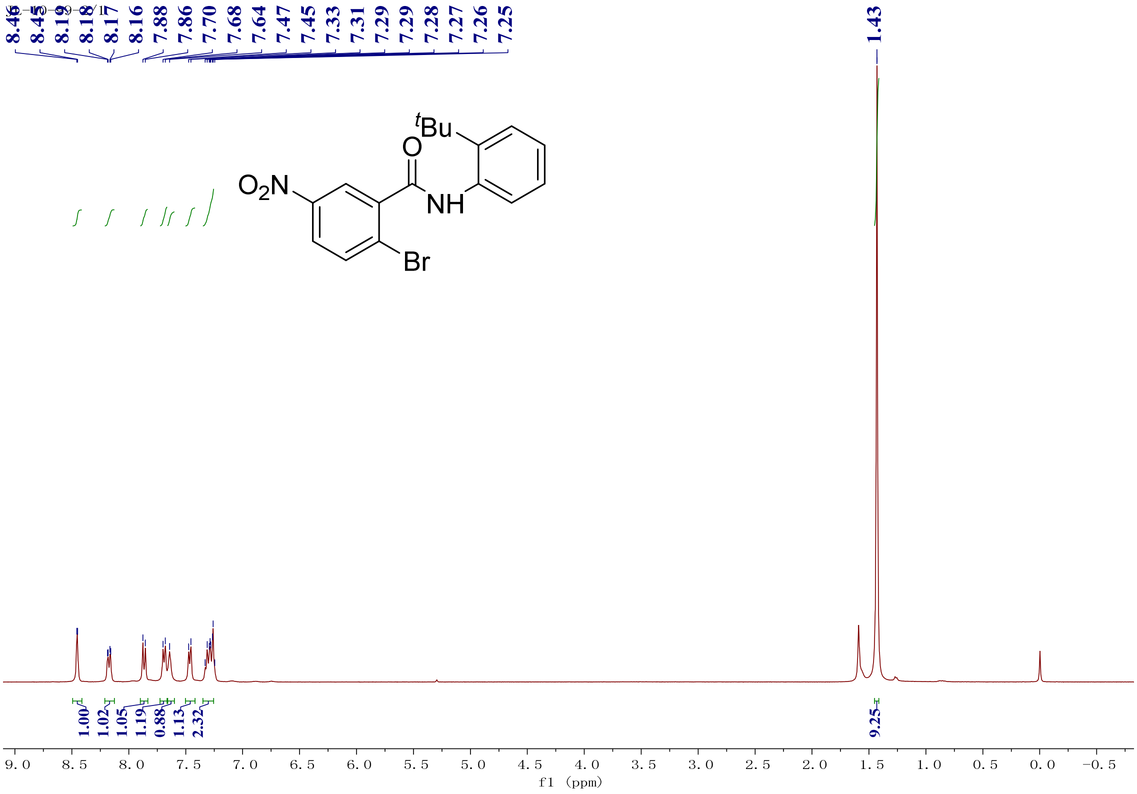

^{13}C NMR (101 MHz, Chloroform-*d*)

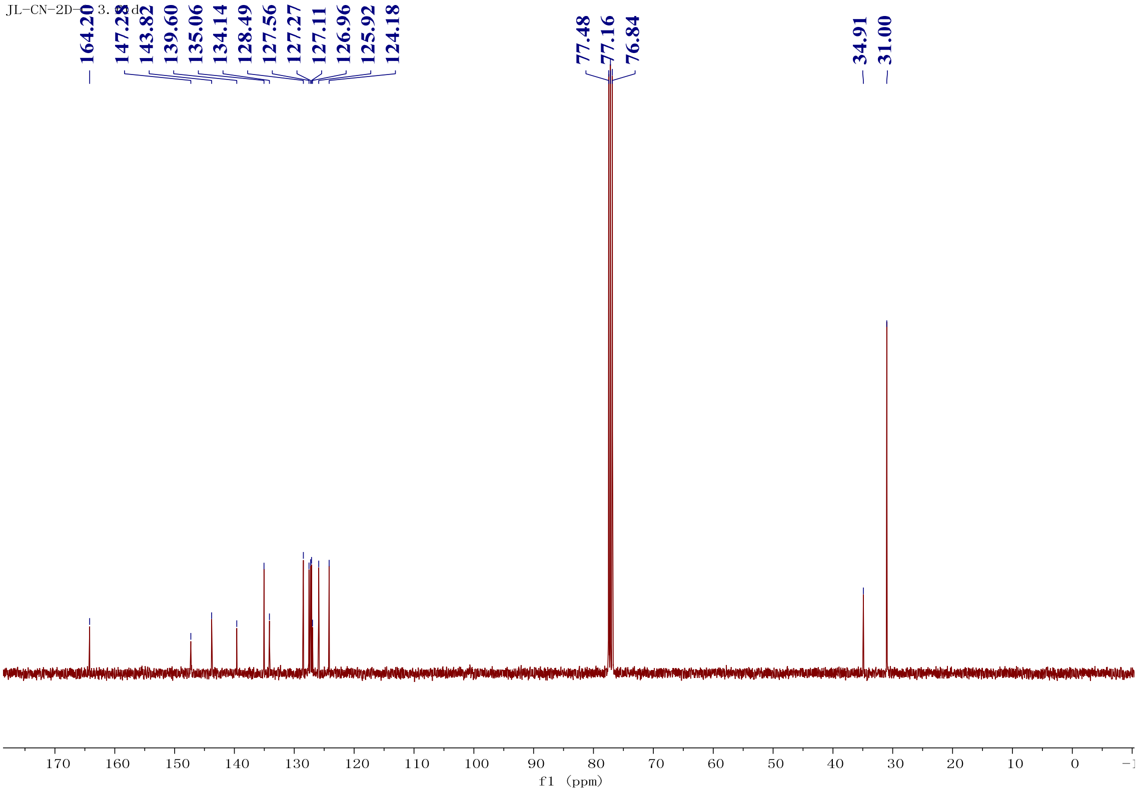

2e

^{1}H NMR (400 MHz, Chloroform-*d*)

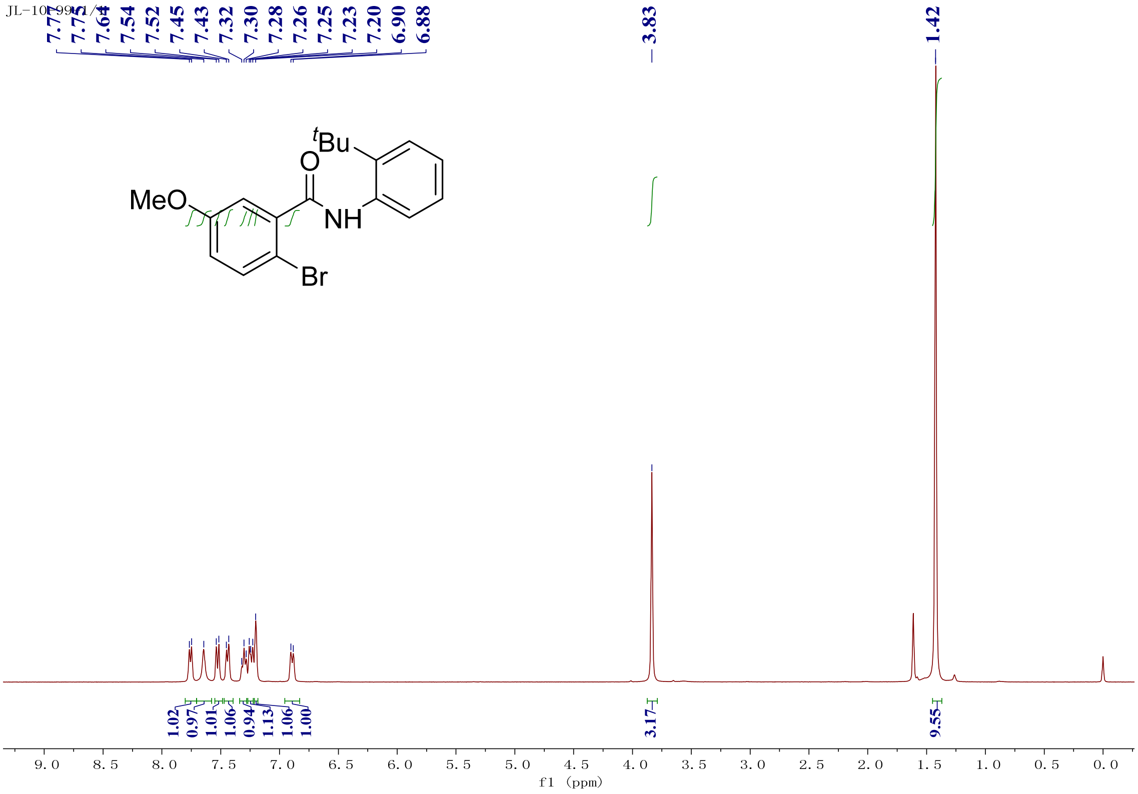

^{13}C NMR (101 MHz, Chloroform-*d*)

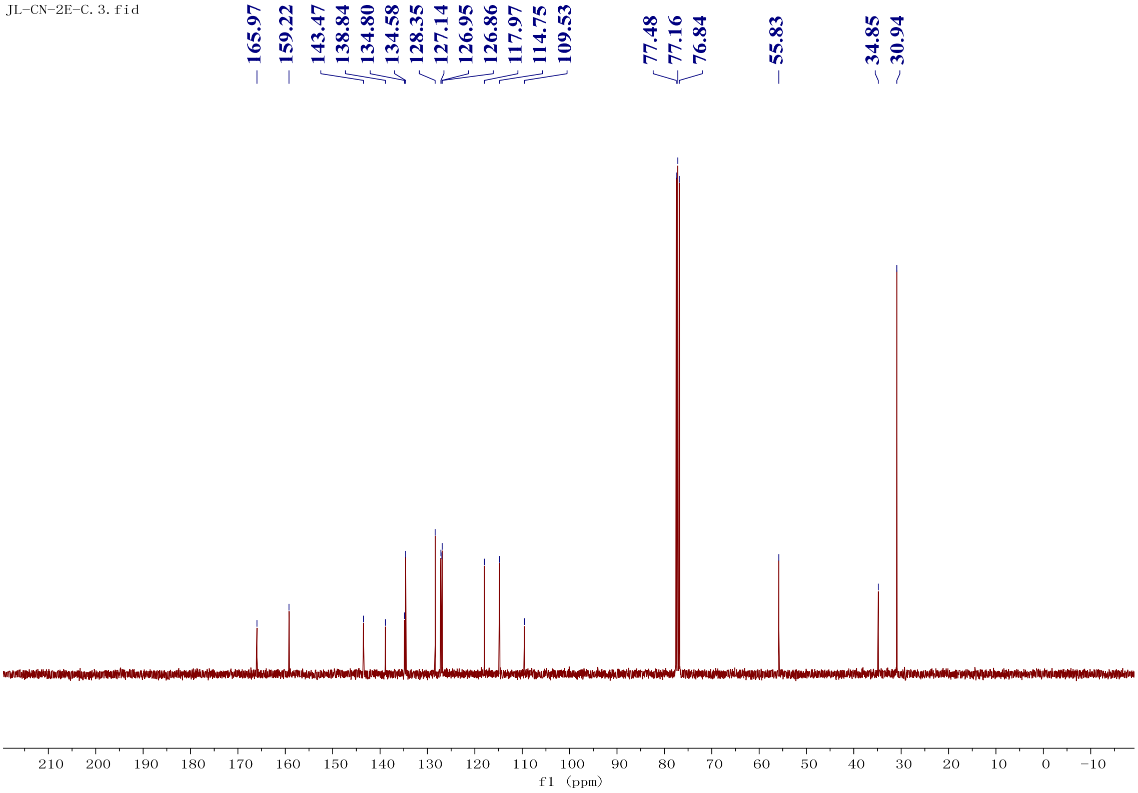

2f

^{1}H NMR (400 MHz, Chloroform-*d*)

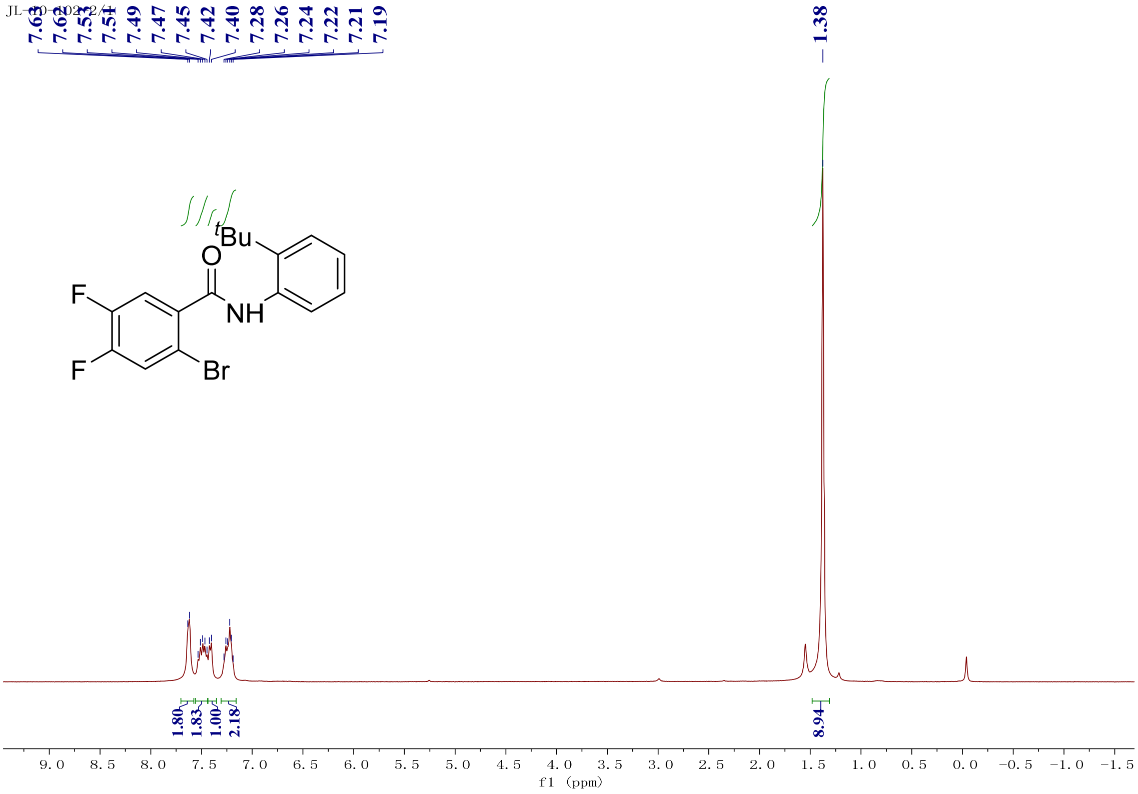

^{13}C NMR (101 MHz, Chloroform-*d*)

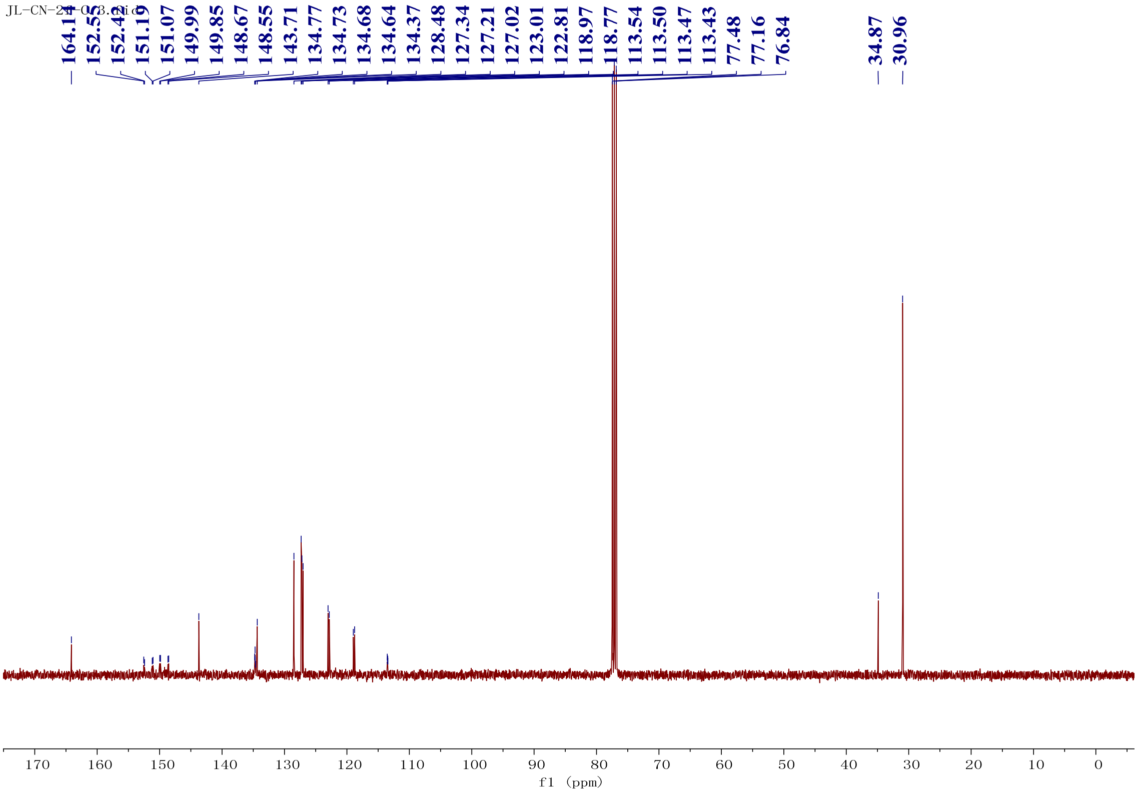

^{19}F NMR (376 MHz, Chloroform-*d*)

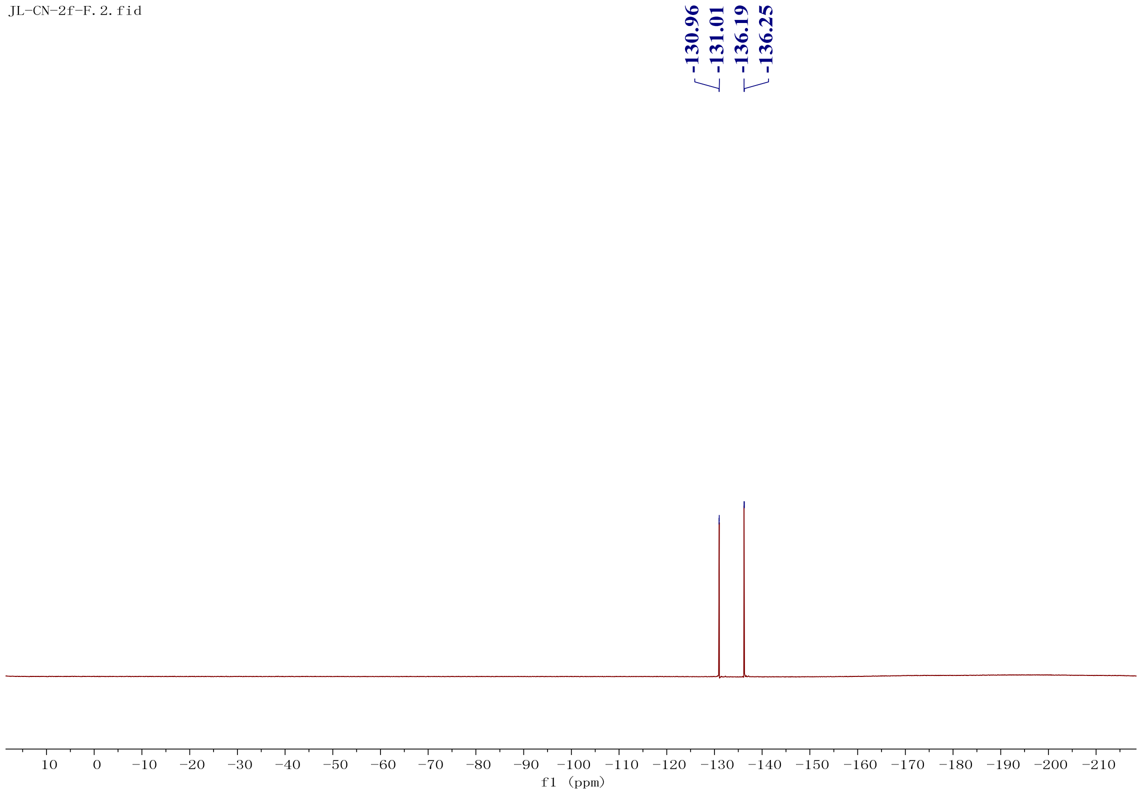

2g

^{1}H NMR (400 MHz, Chloroform-*d*)

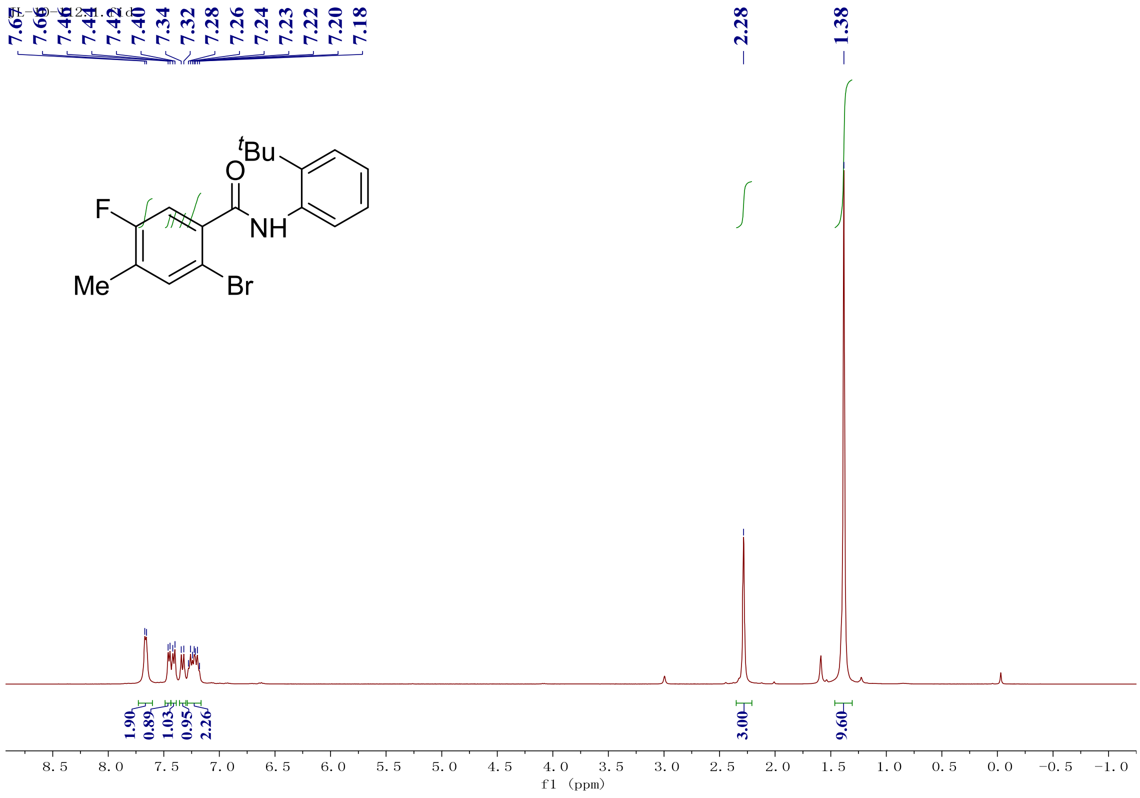

^{13}C NMR (101 MHz, Chloroform-*d*)

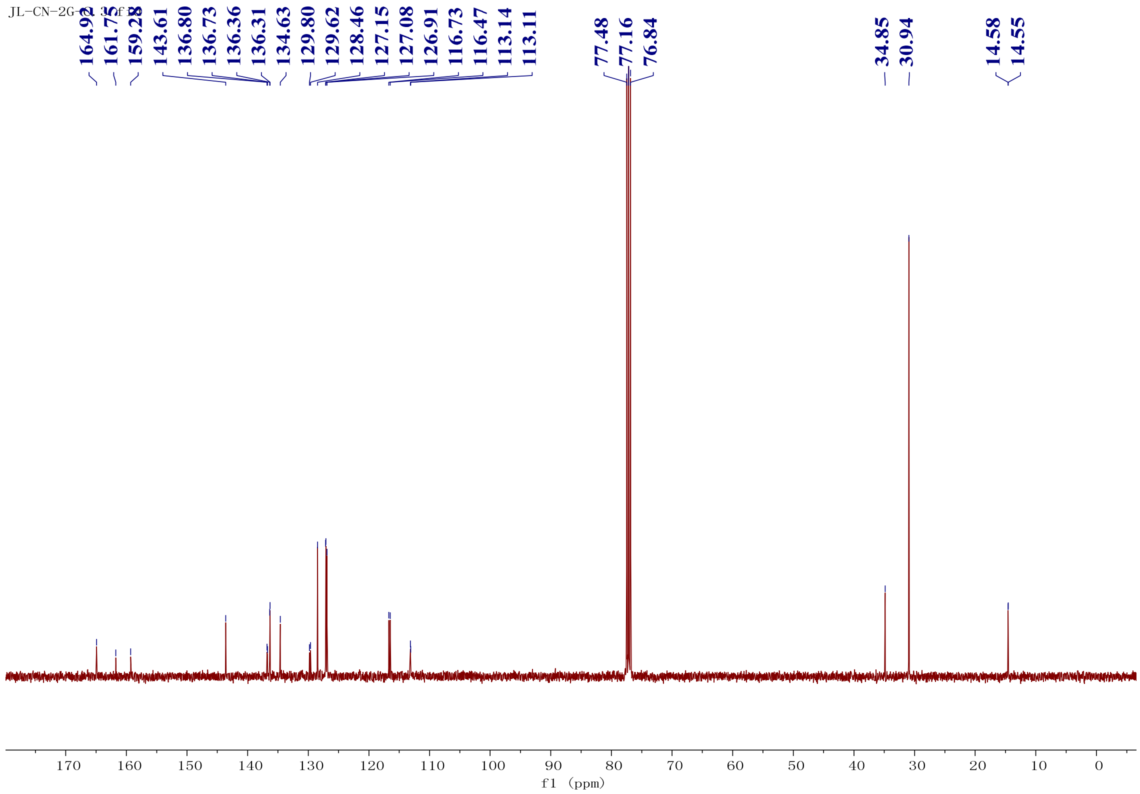

^{19}F NMR (376 MHz, Chloroform-*d*)

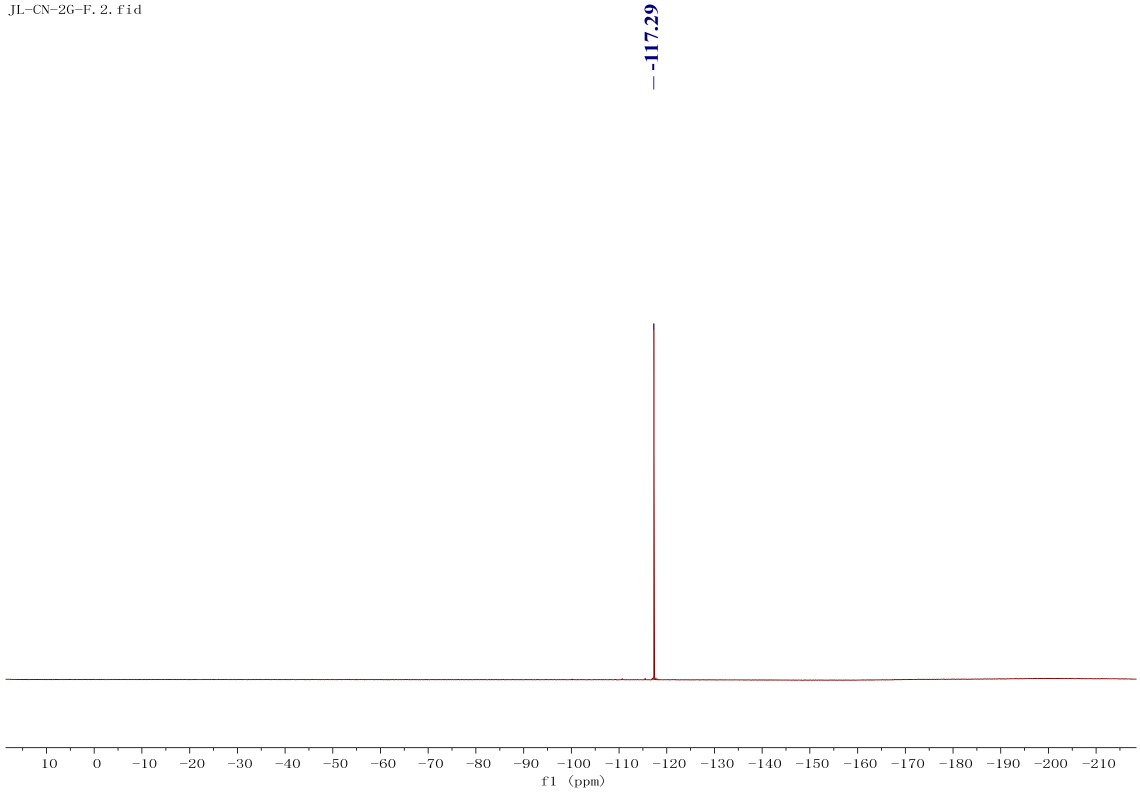

2h

^{1}H NMR (400 MHz, Chloroform-*d*)

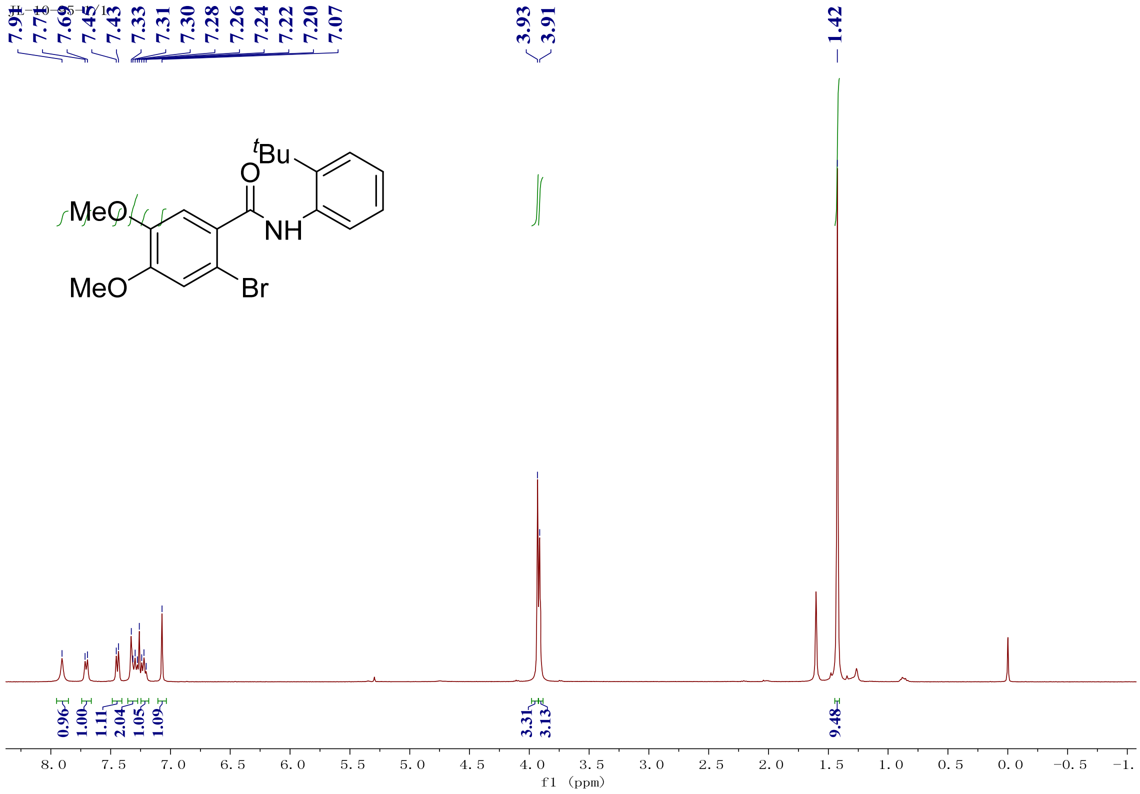

^{13}C NMR (101 MHz, Chloroform-*d*)

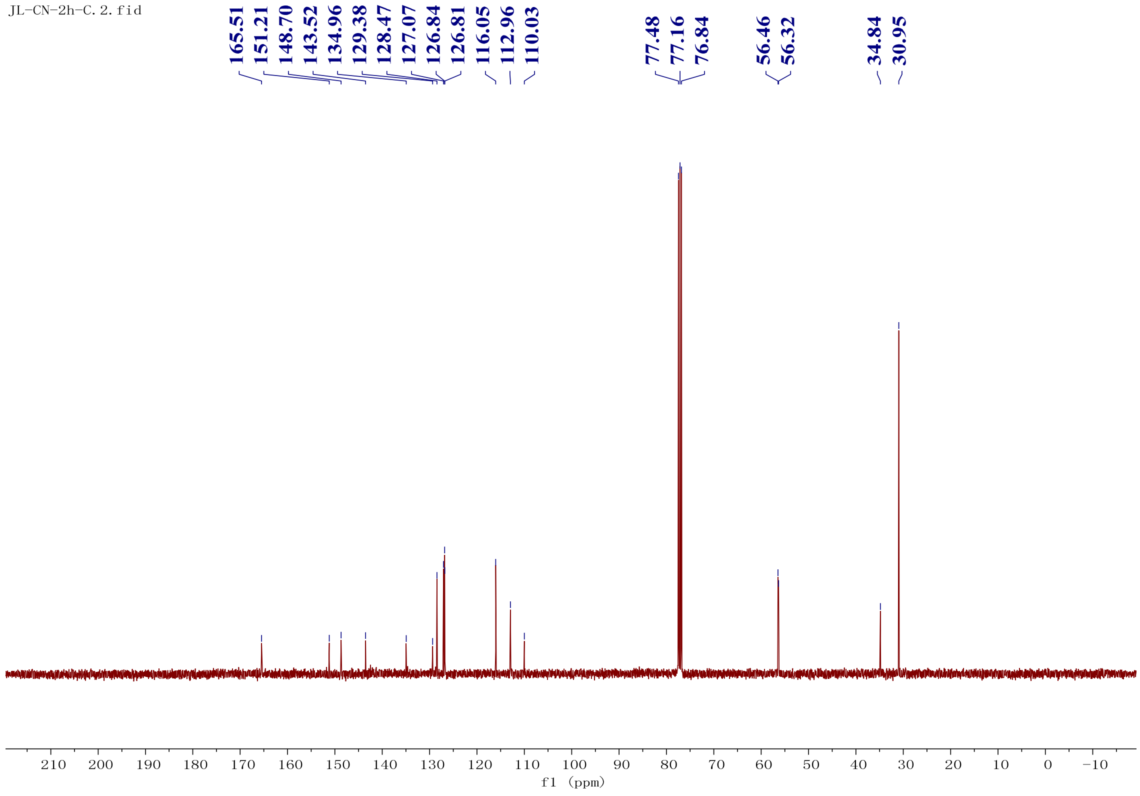

2i

^{1}H NMR (400 MHz, Chloroform-*d*)

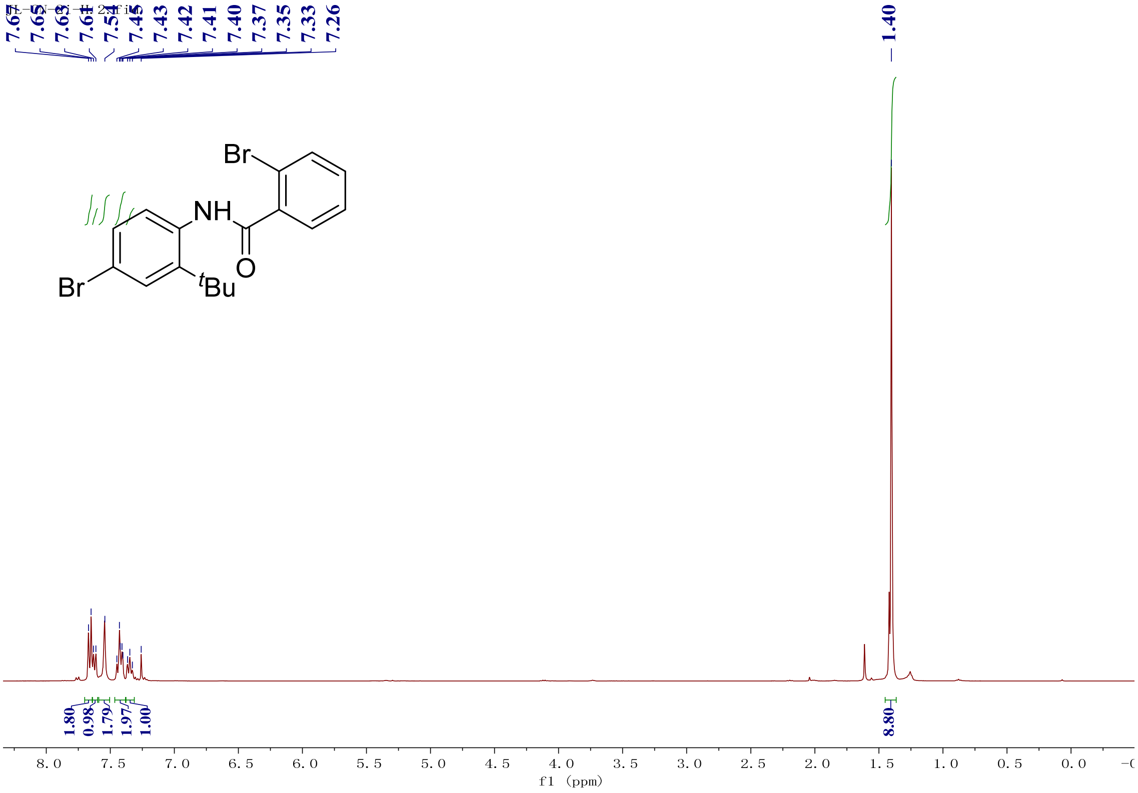

^{13}C NMR (101 MHz, Chloroform-*d*)

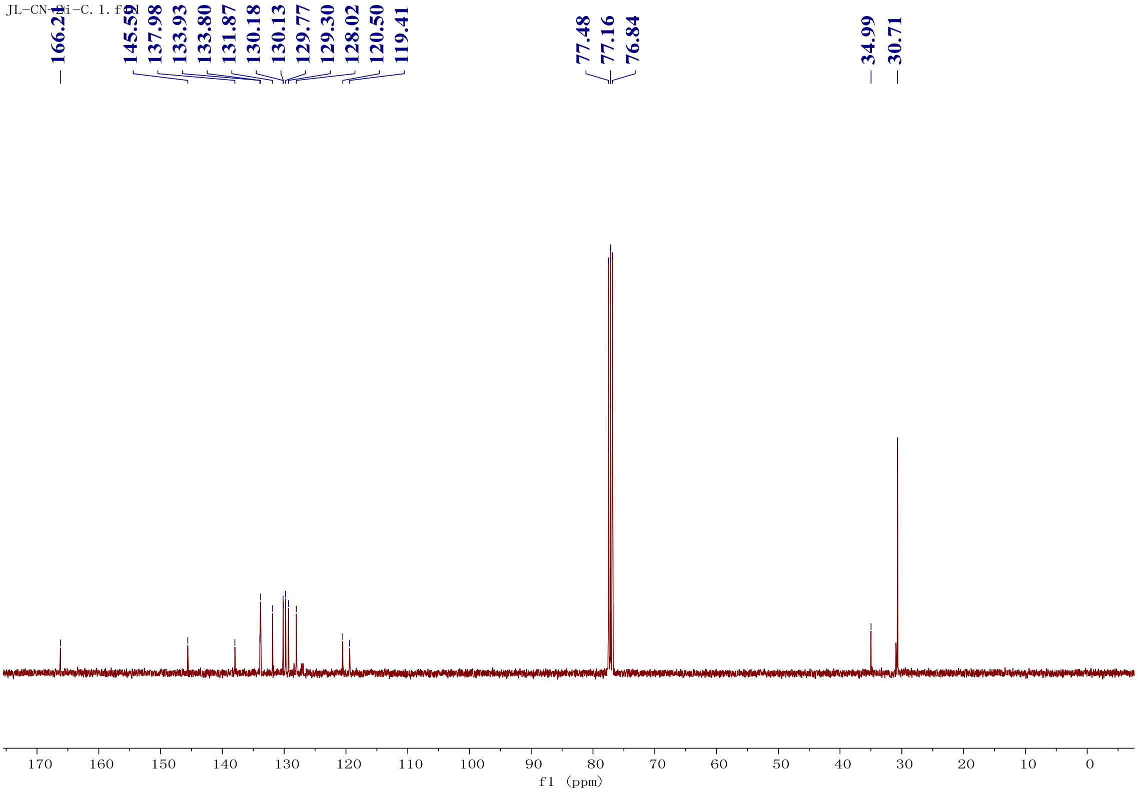

2j

^{1}H NMR (400 MHz, Chloroform-*d*)

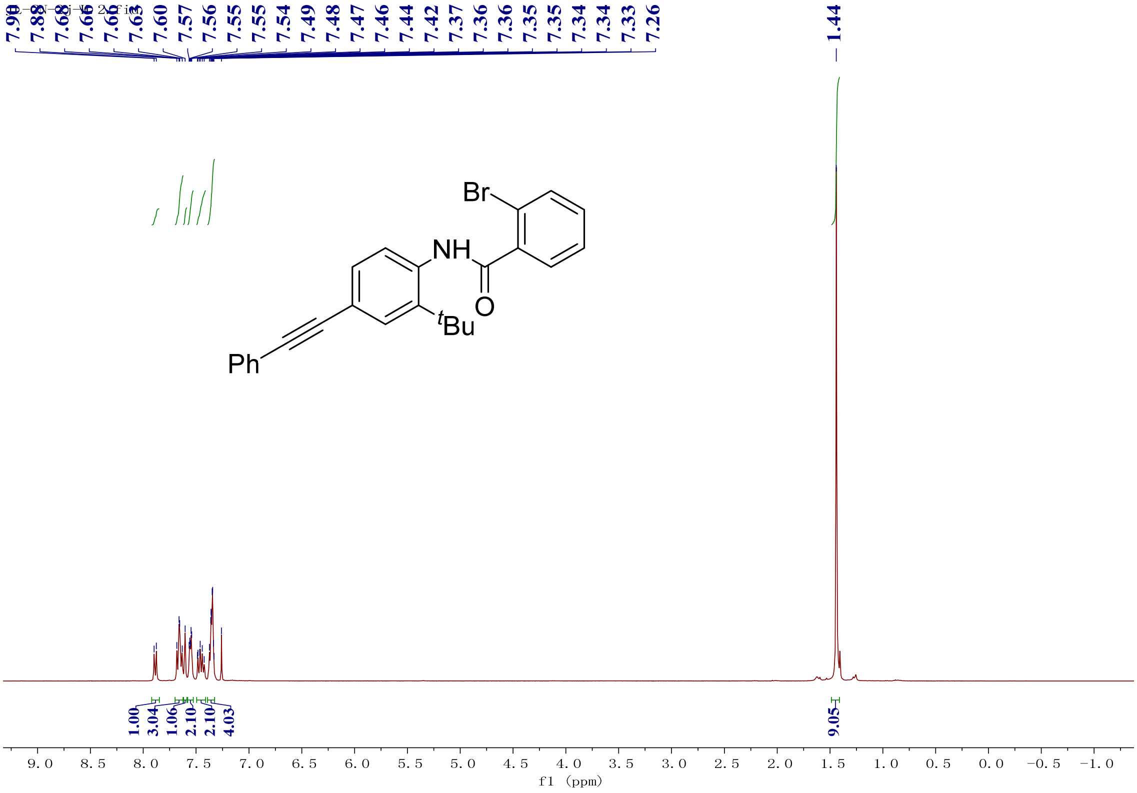

^{13}C NMR (101 MHz, Chloroform-*d*)

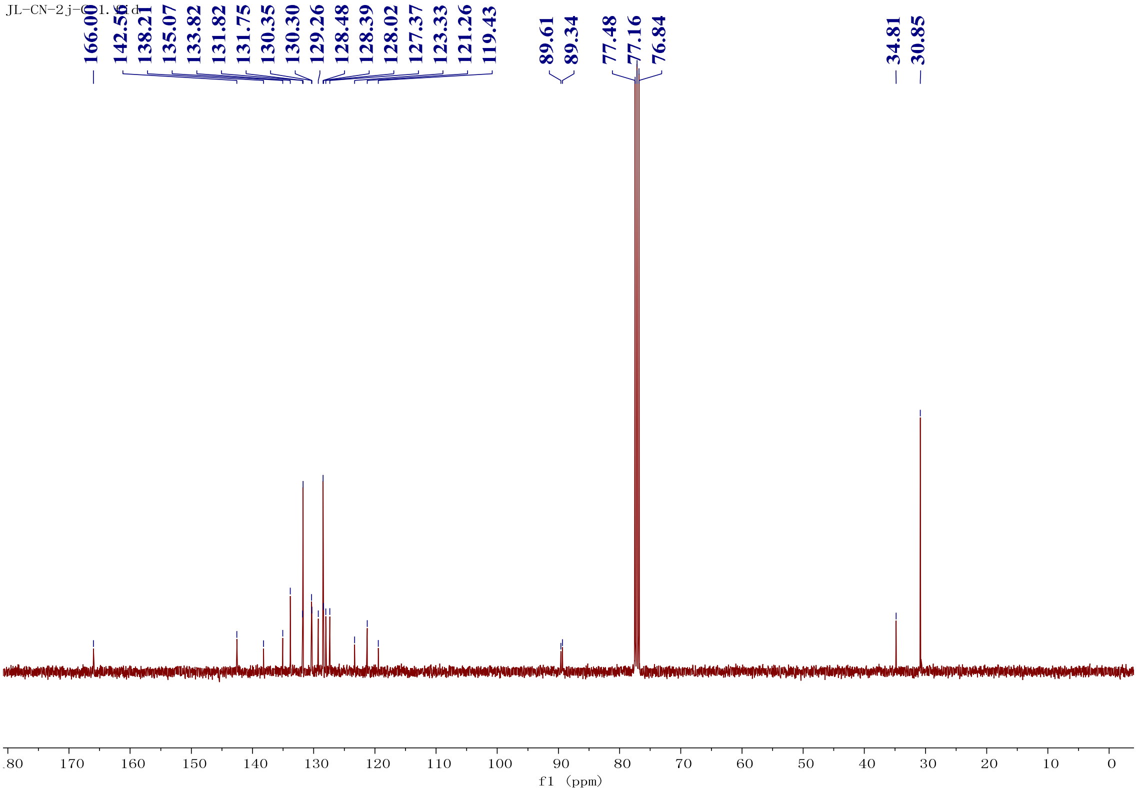

2k

^{1}H NMR (400 MHz, Chloroform-*d*)

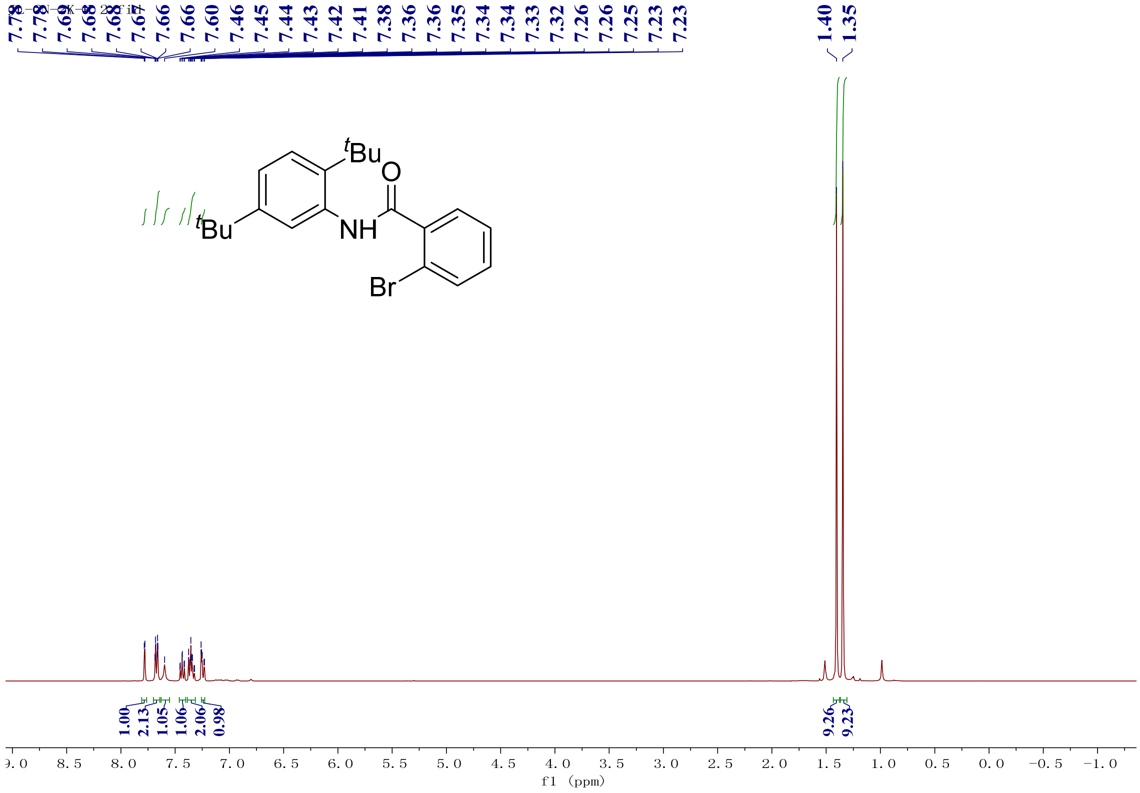

^{13}C NMR (101 MHz, Chloroform-*d*)

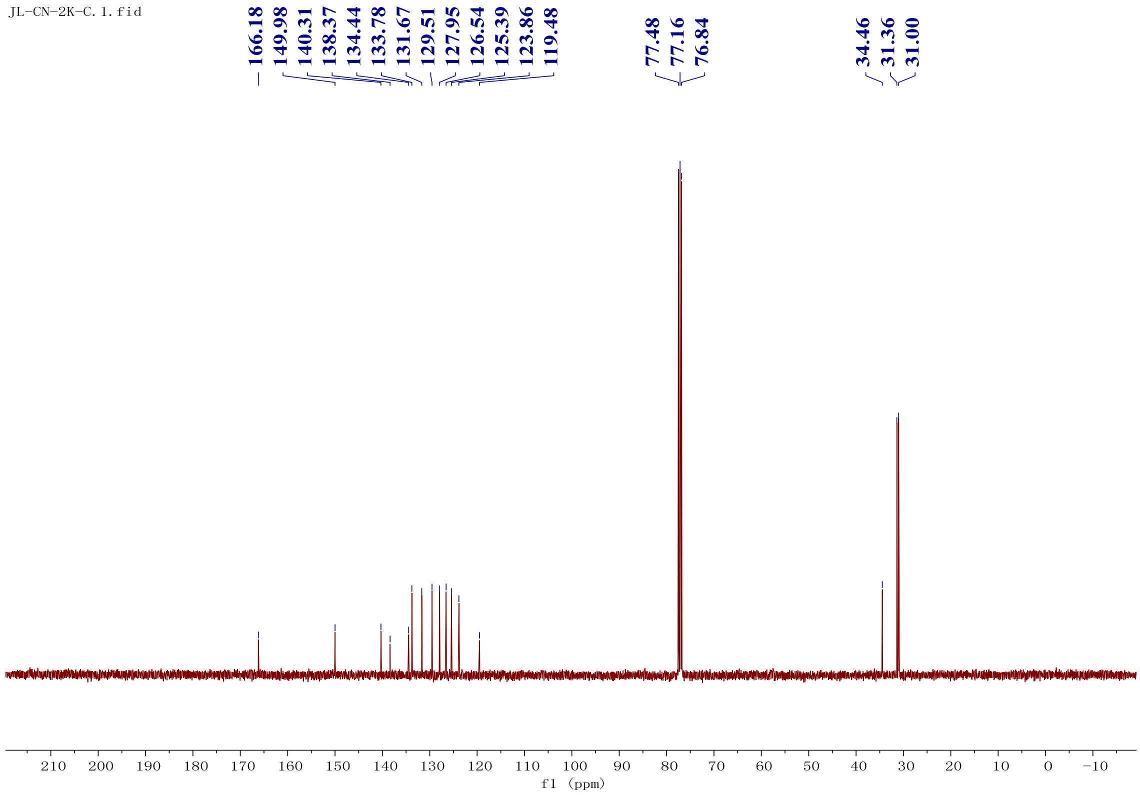

2l

^{1}H NMR (400 MHz, Chloroform-*d*)

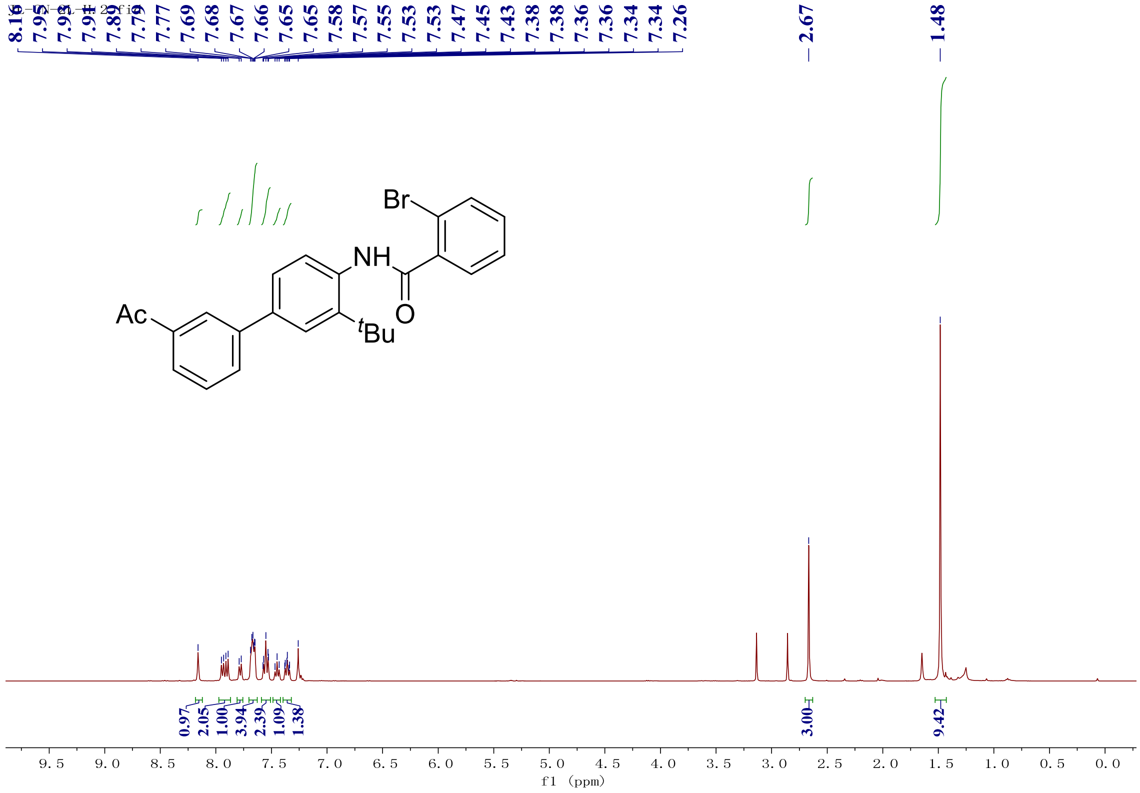

^{13}C NMR (101 MHz, Chloroform-*d*)

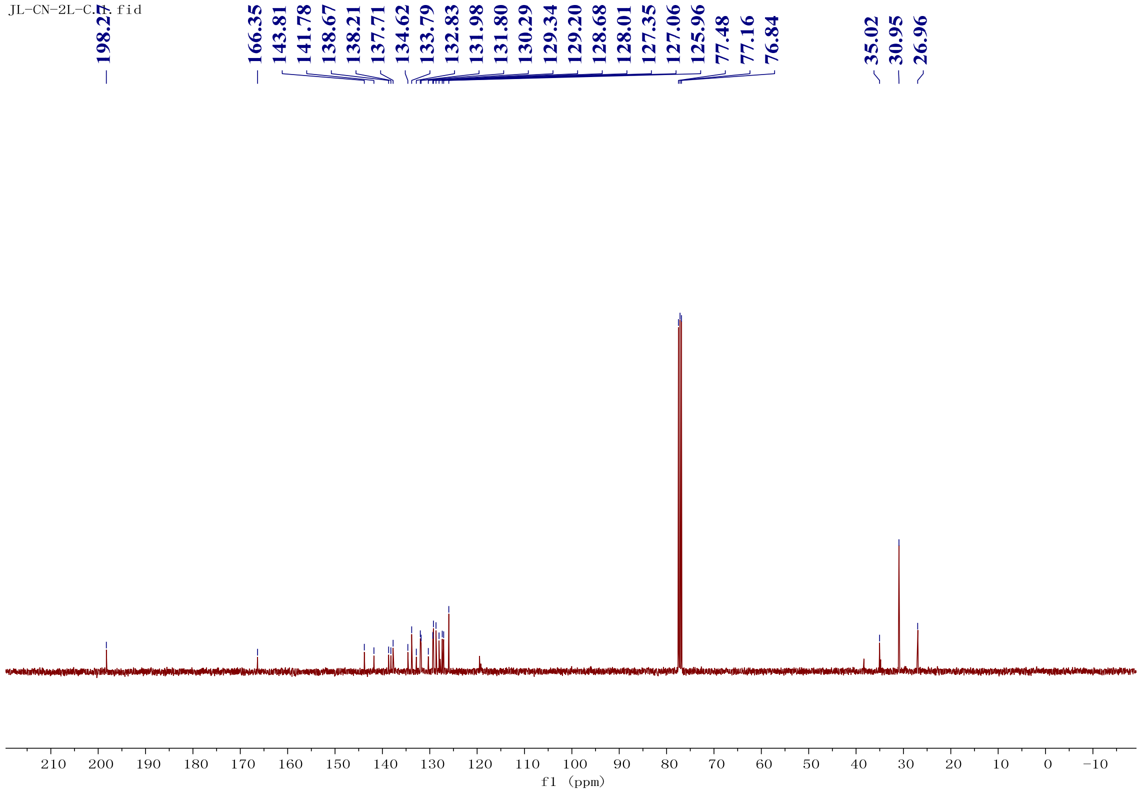

2m

^{1}H NMR (400 MHz, Chloroform-*d*)

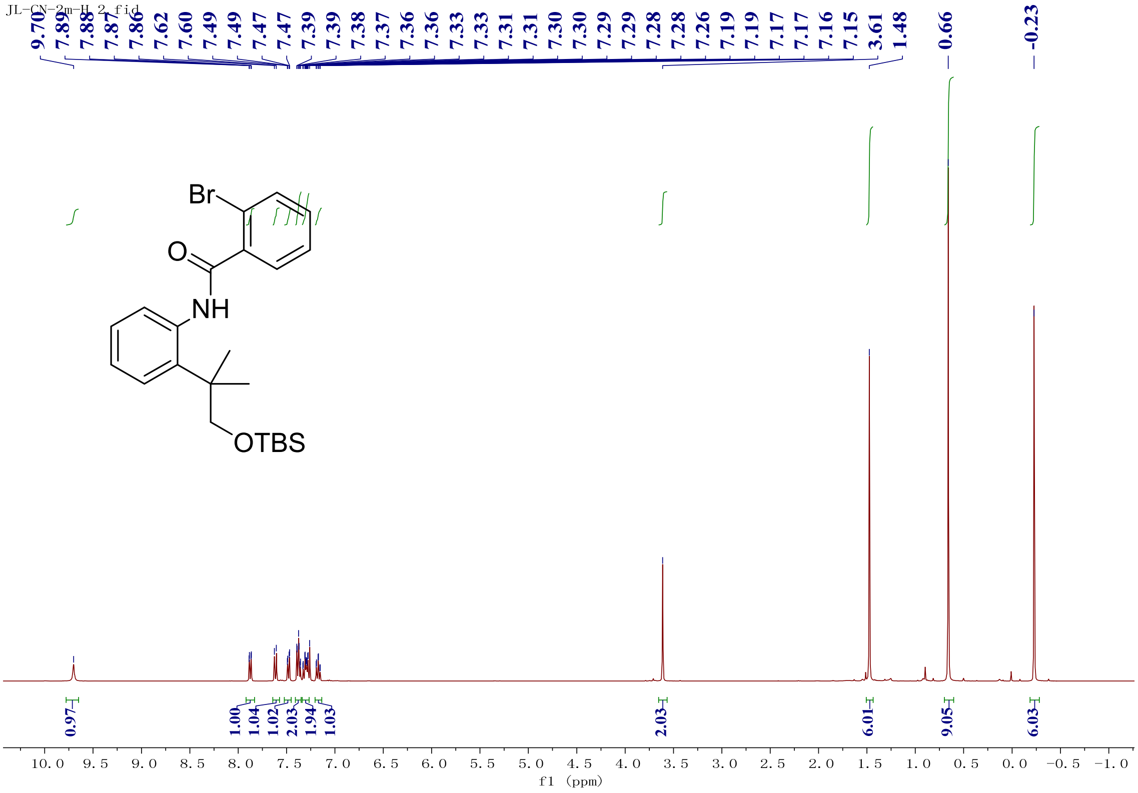

^{13}C NMR (101 MHz, Chloroform-*d*)

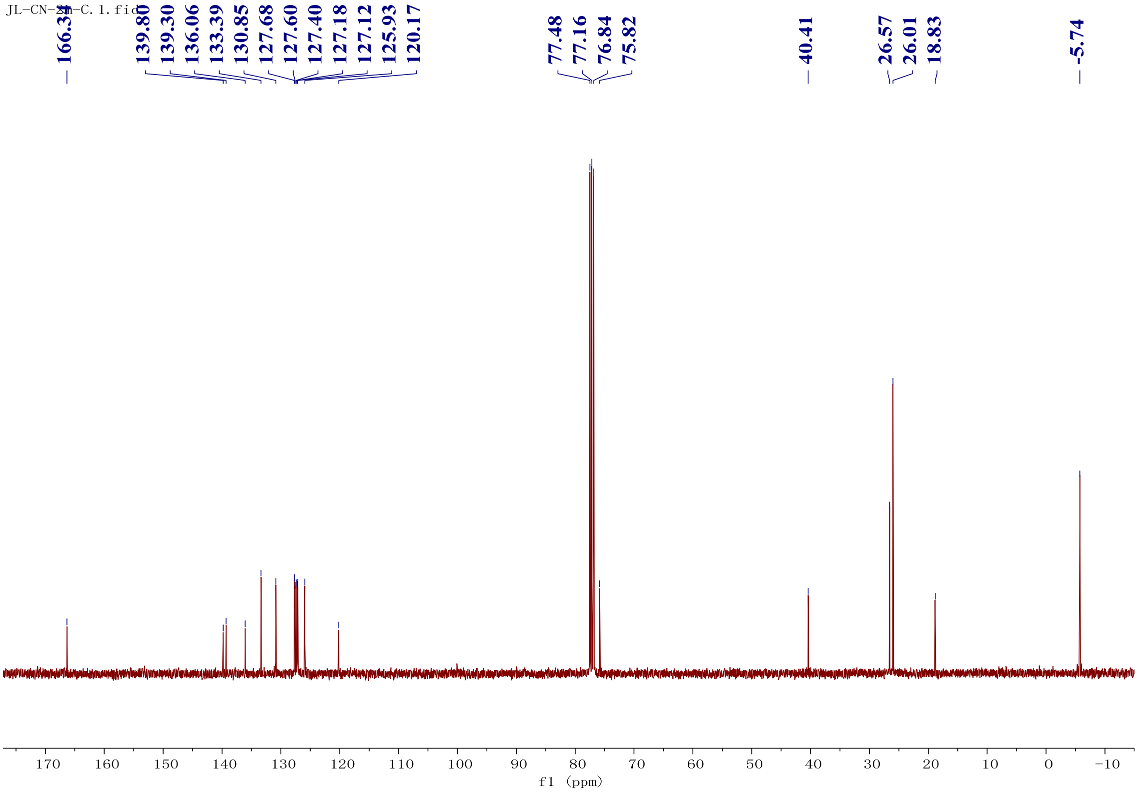

2n

^{1}H NMR (400 MHz, Chloroform-*d*)

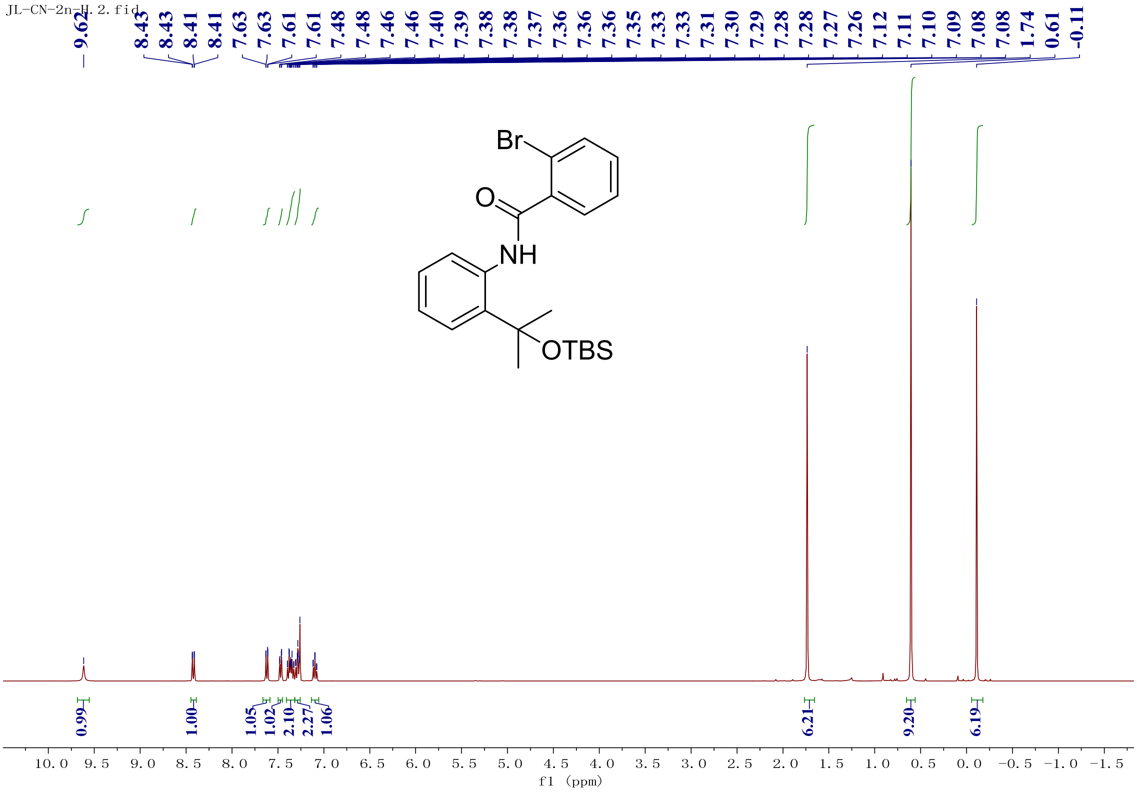

^{13}C NMR (101 MHz, Chloroform-*d*)

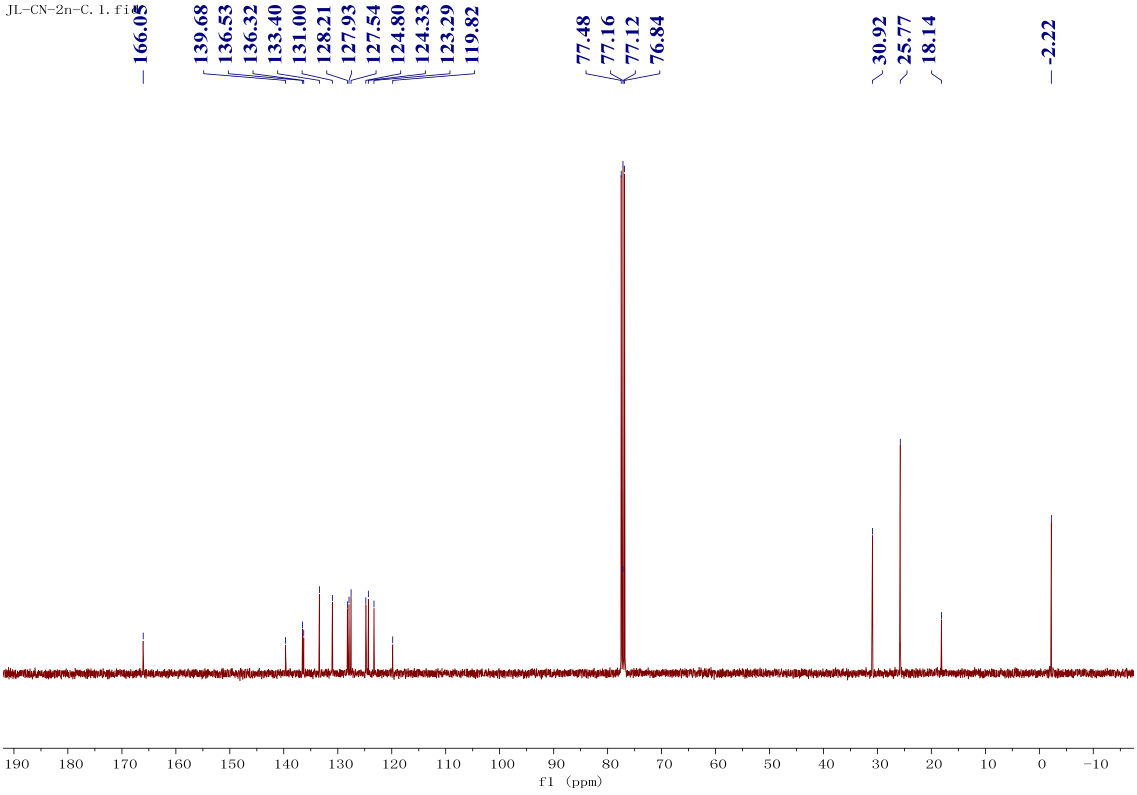

2o

^{1}H NMR (400 MHz, Chloroform-*d*)

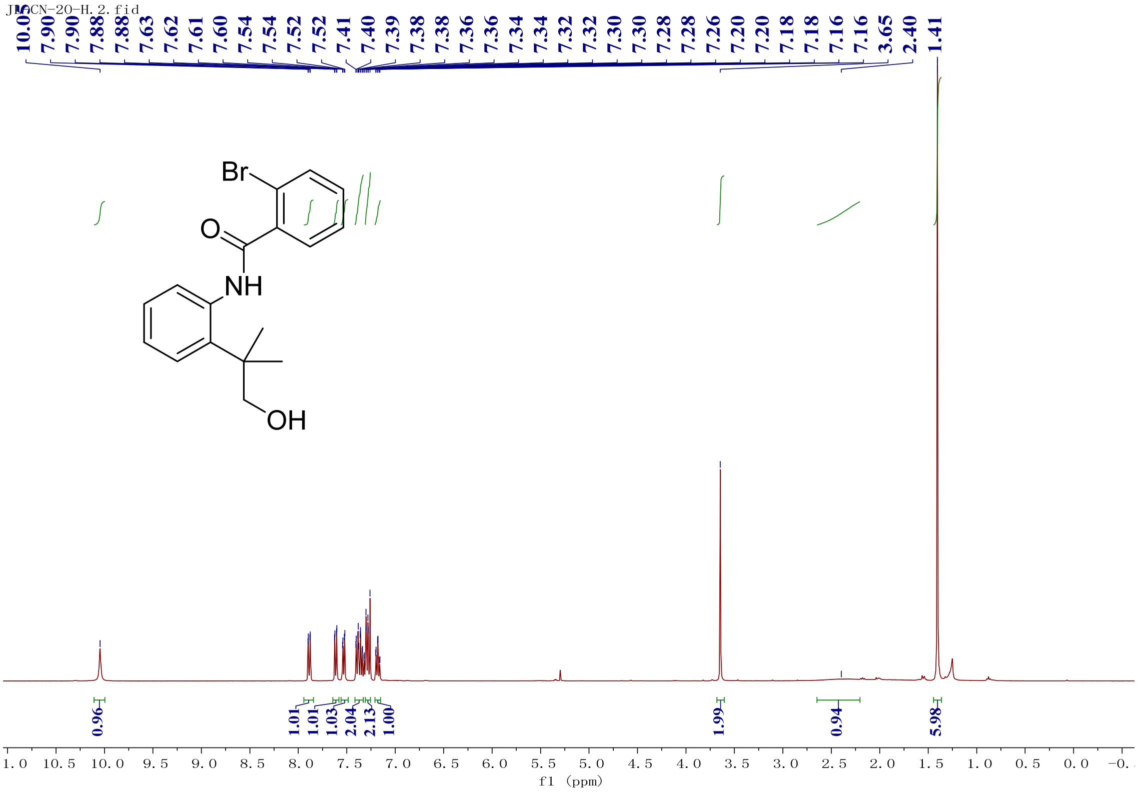

^{13}C NMR (101 MHz, Chloroform-*d*)

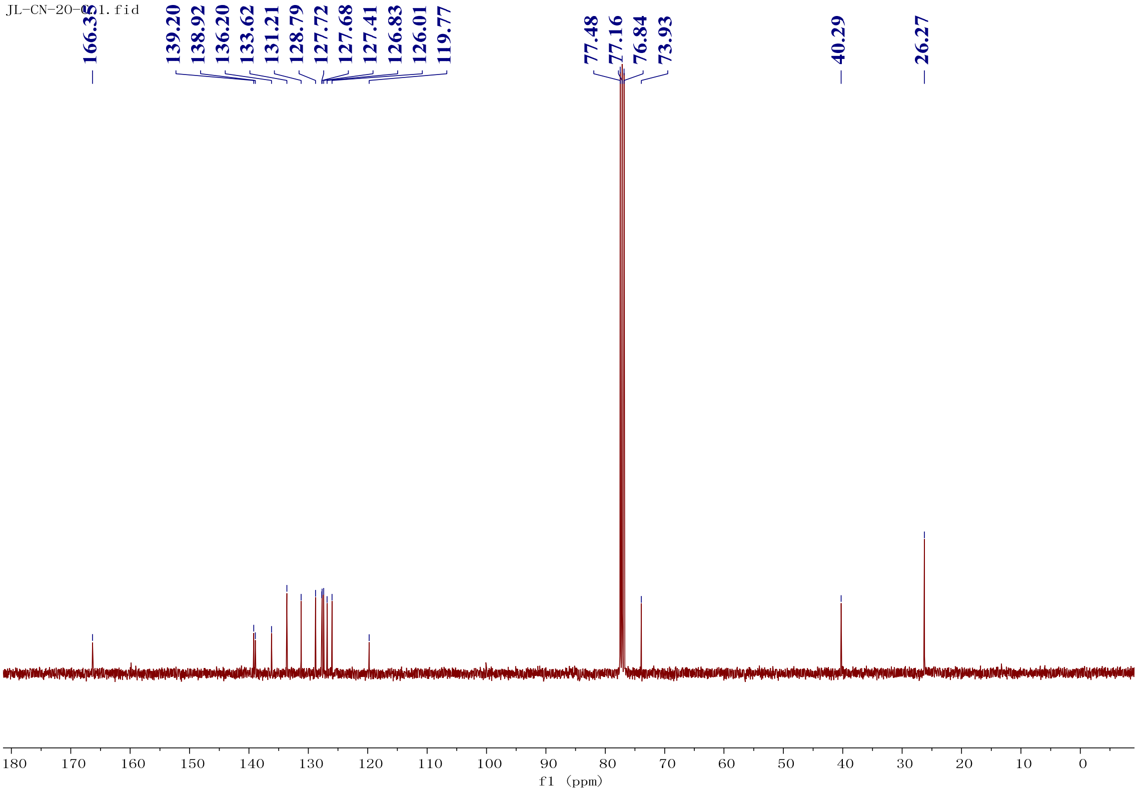

2p

^{1}H NMR (400 MHz, Chloroform-*d*)

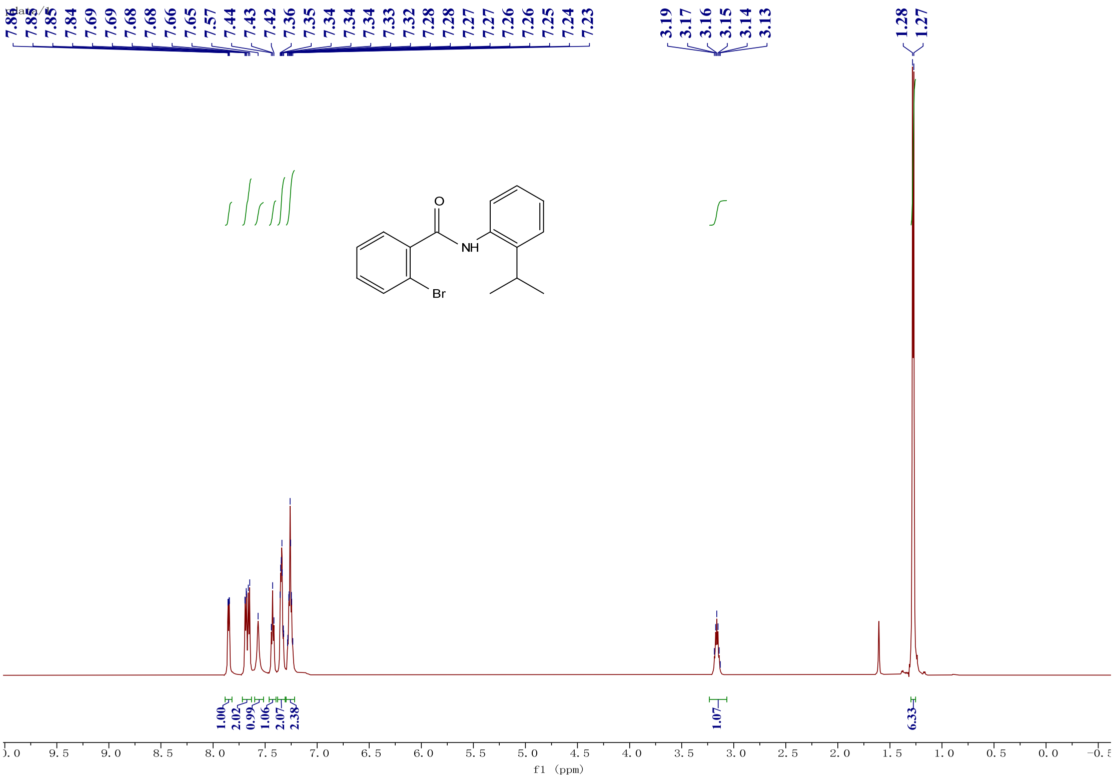

^{13}C NMR (151 MHz, Chloroform-*d*)

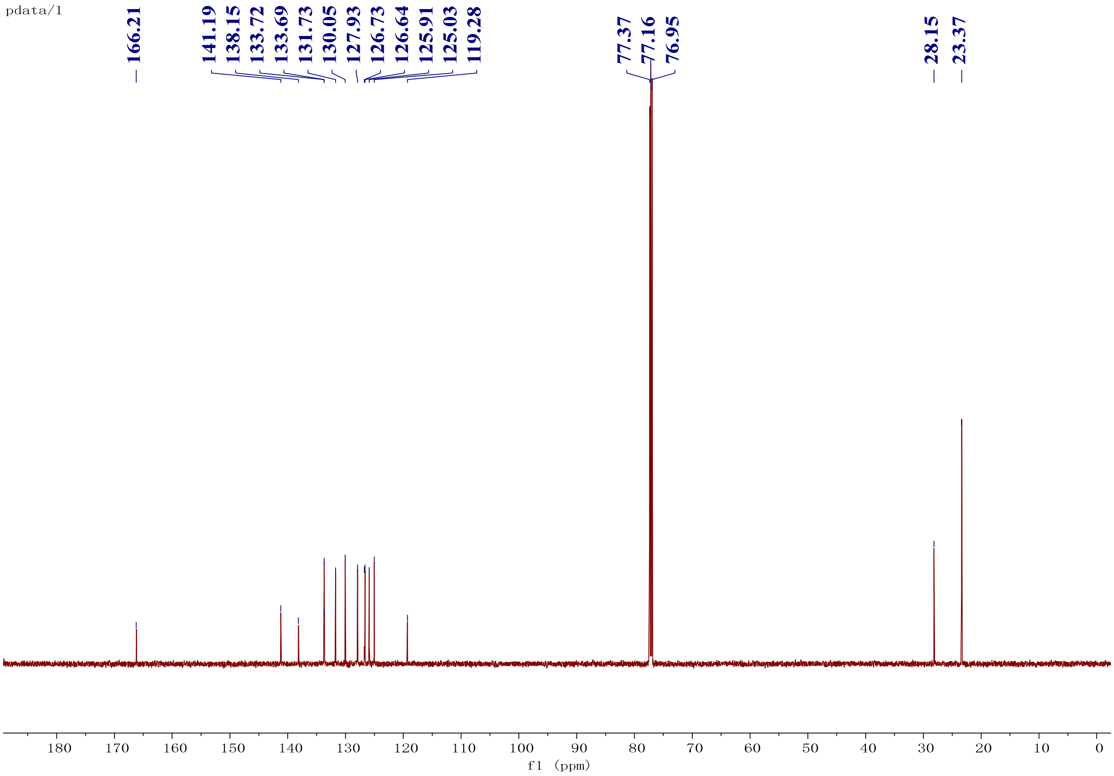

2q

^{1}H NMR (600 MHz, DMSO-d_6)

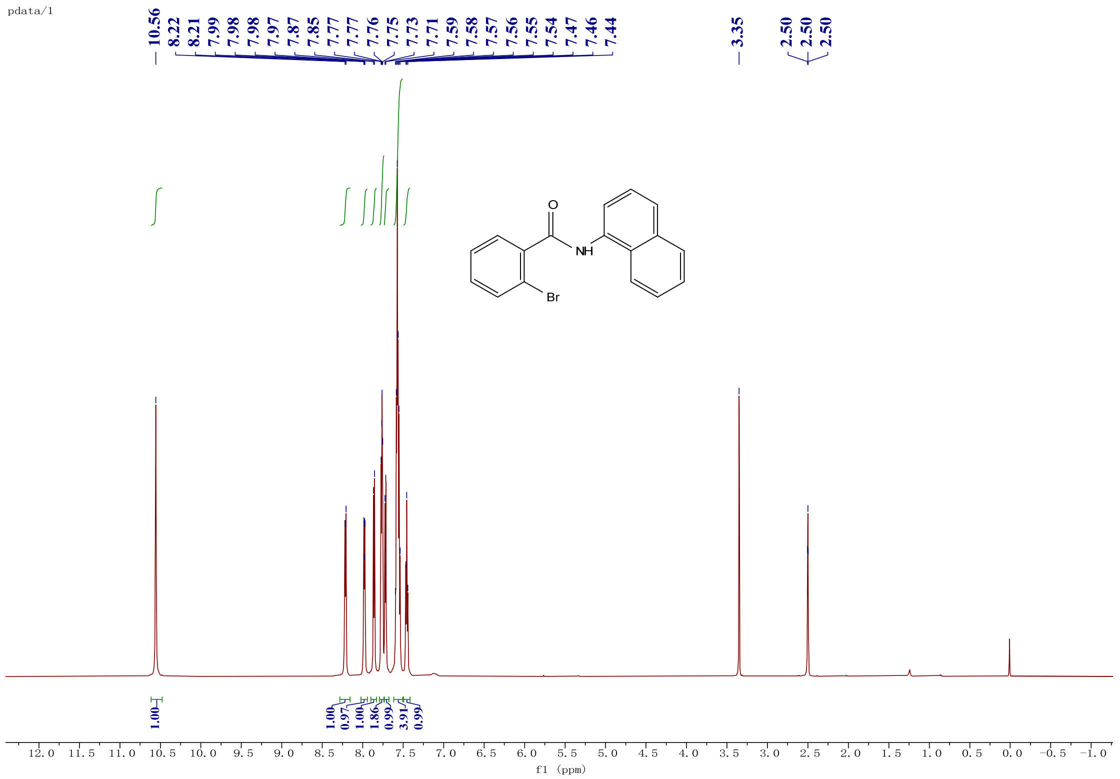

^{13}C NMR (151 MHz, DMSO-d_6)

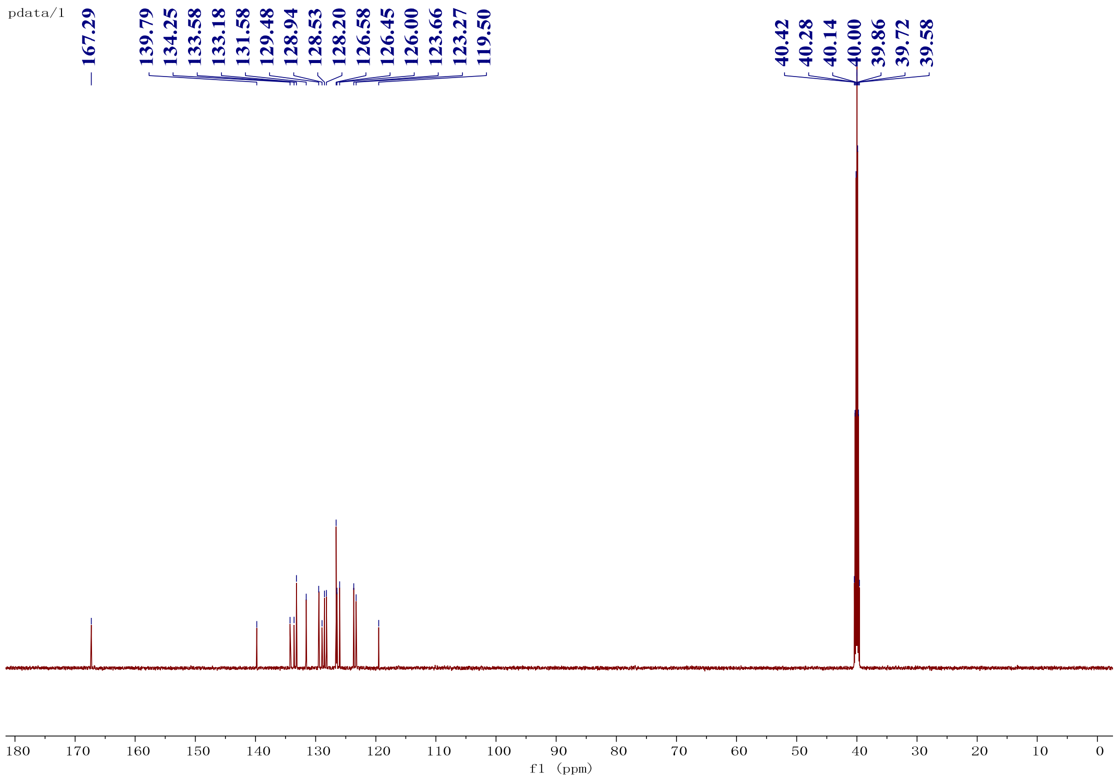

3

^{1}H NMR (600 MHz, Chloroform-*d*)

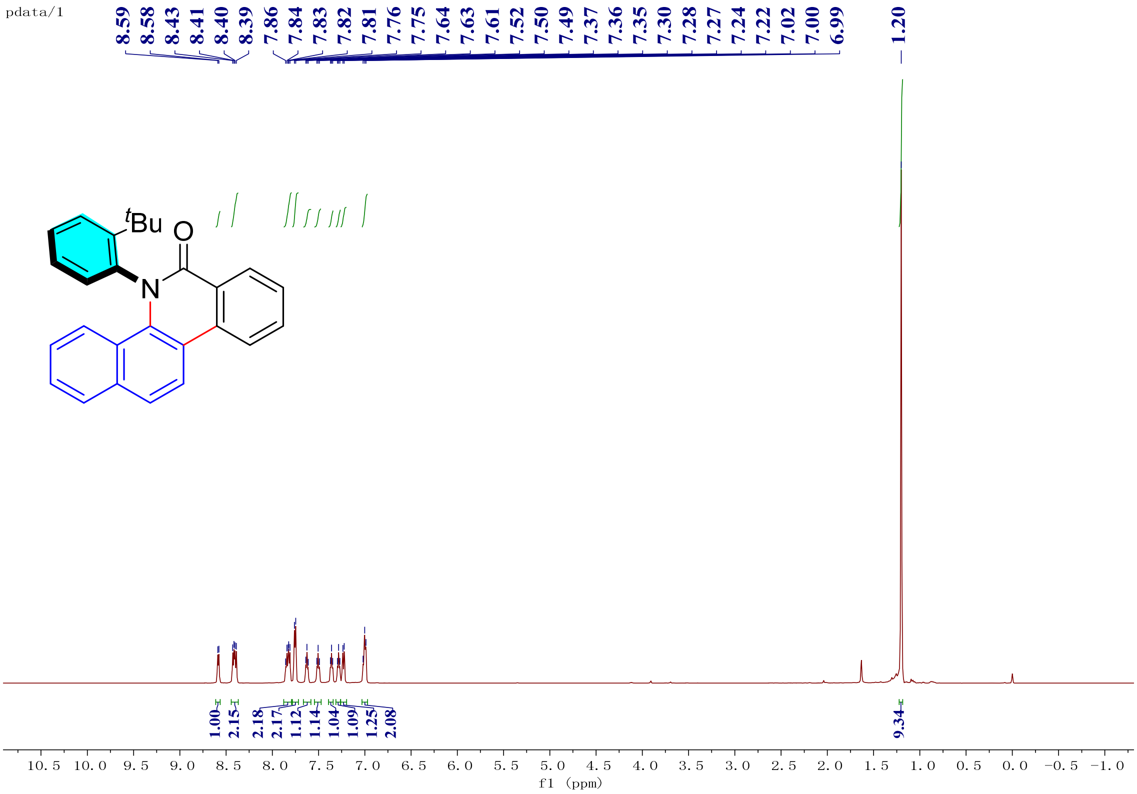

^{13}C NMR (151 MHz, Chloroform-*d*)

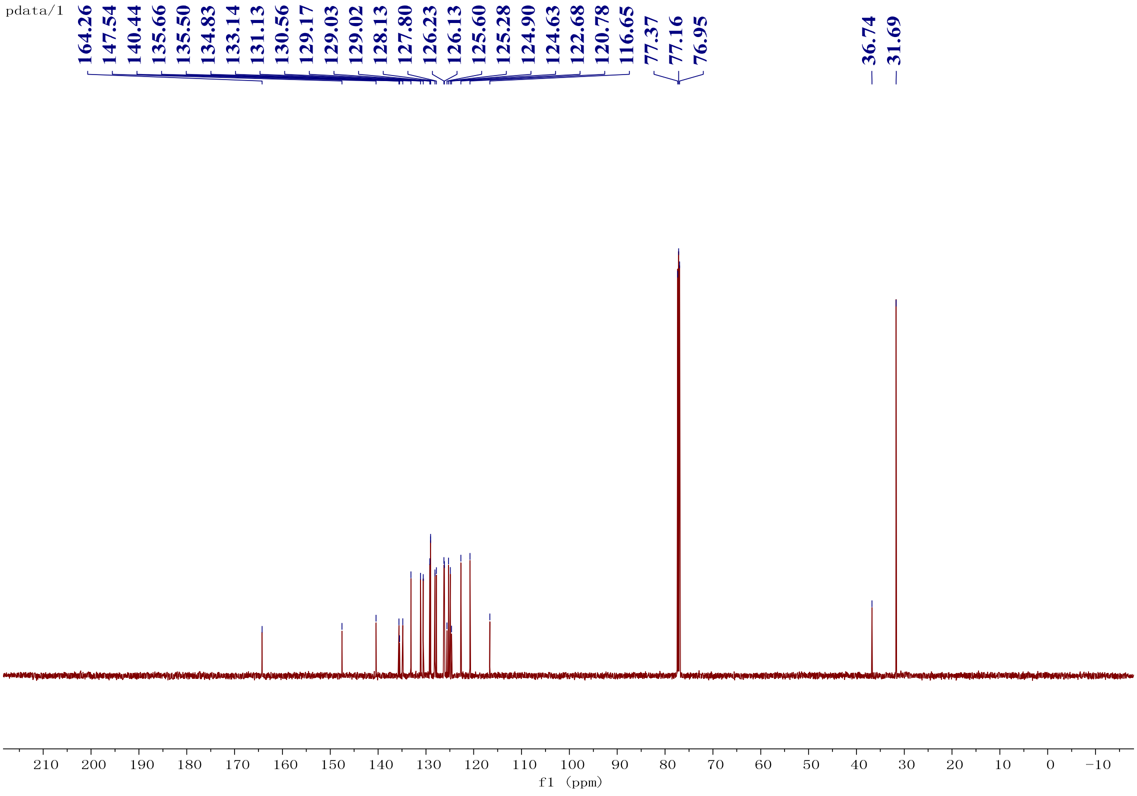

4

^{1}H NMR (600 MHz, Chloroform-*d*)

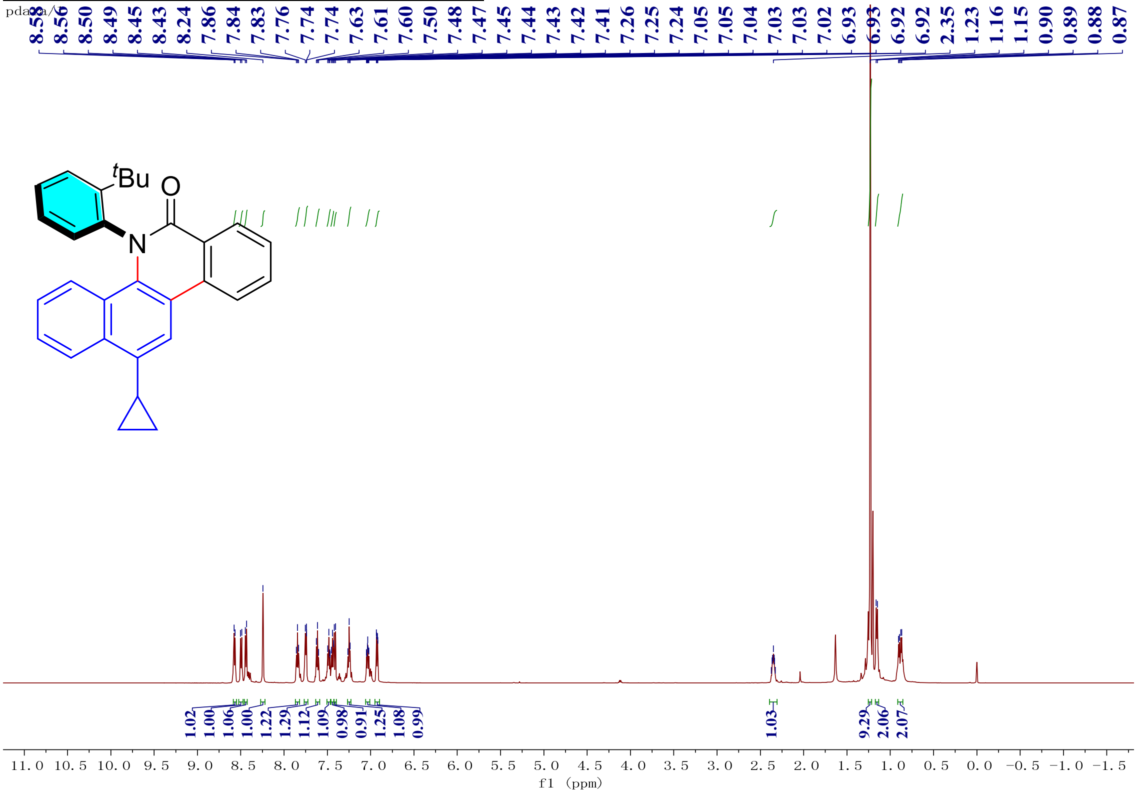

^{13}C NMR (151 MHz, Chloroform-*d*)

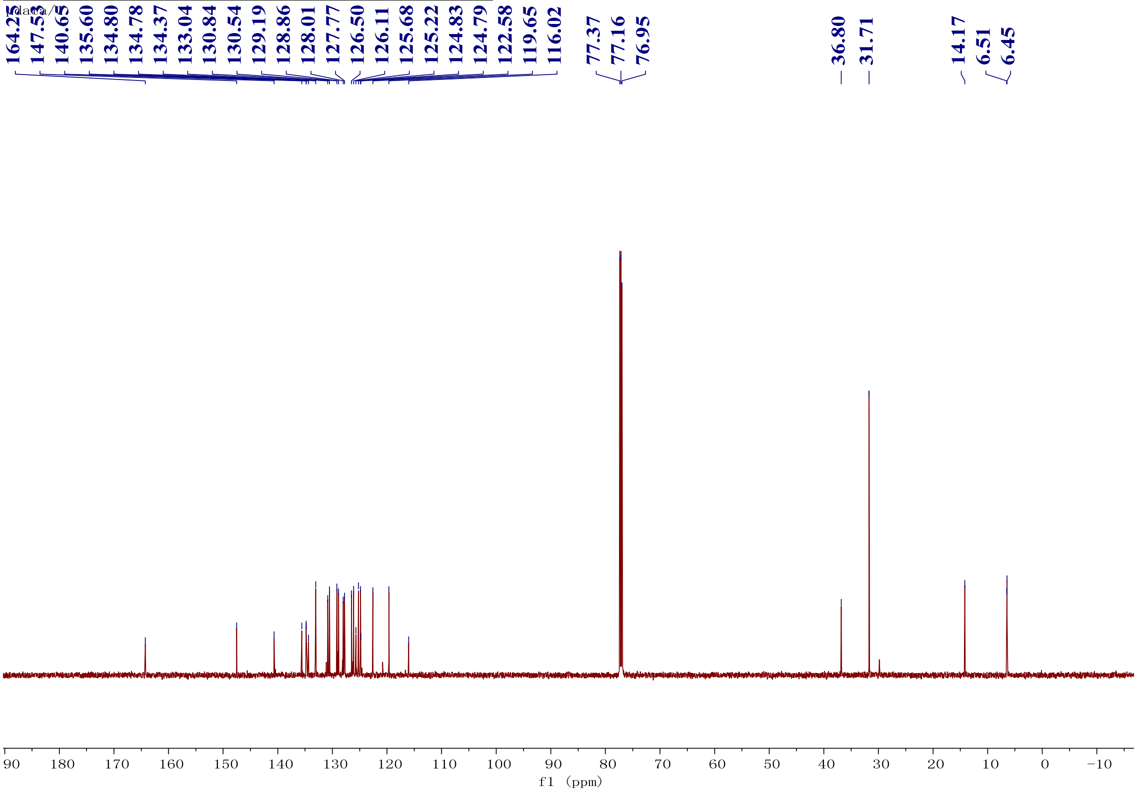

5

^{1}H NMR (600 MHz, Chloroform-*d*)

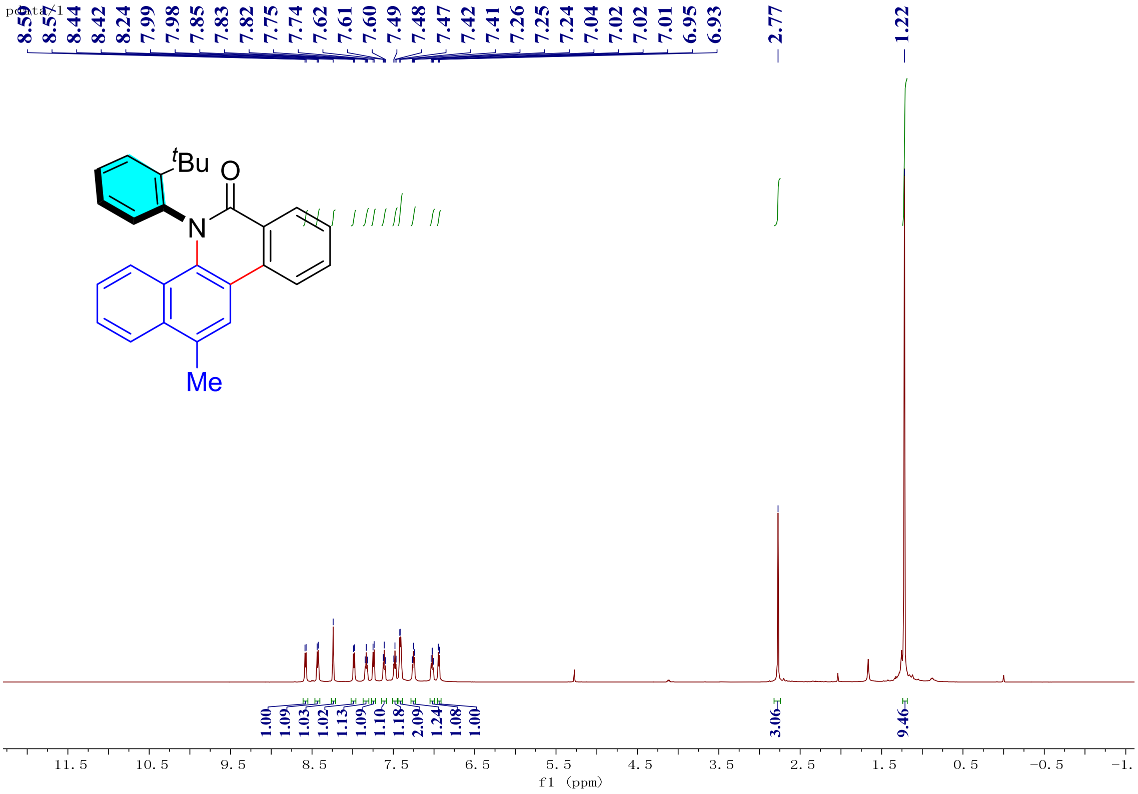

^{13}C NMR (151 MHz, Chloroform-*d*)

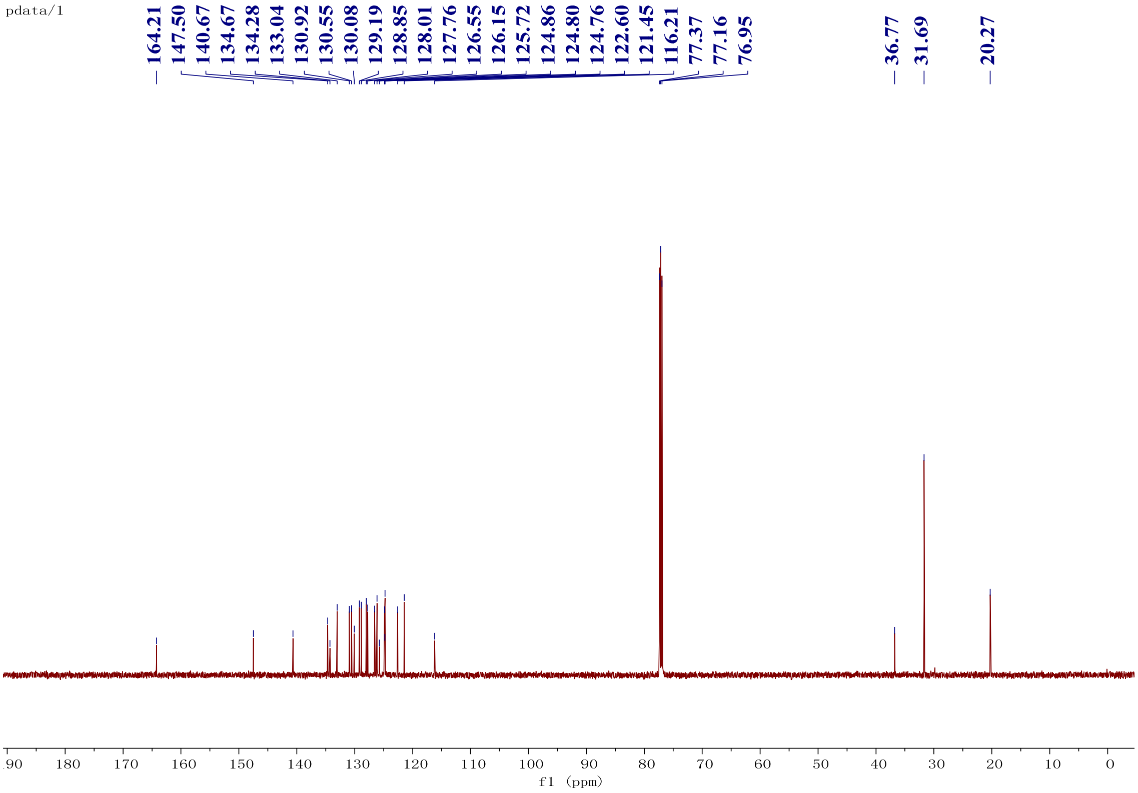

6

^{1}H NMR (600 MHz, Chloroform-*d*)

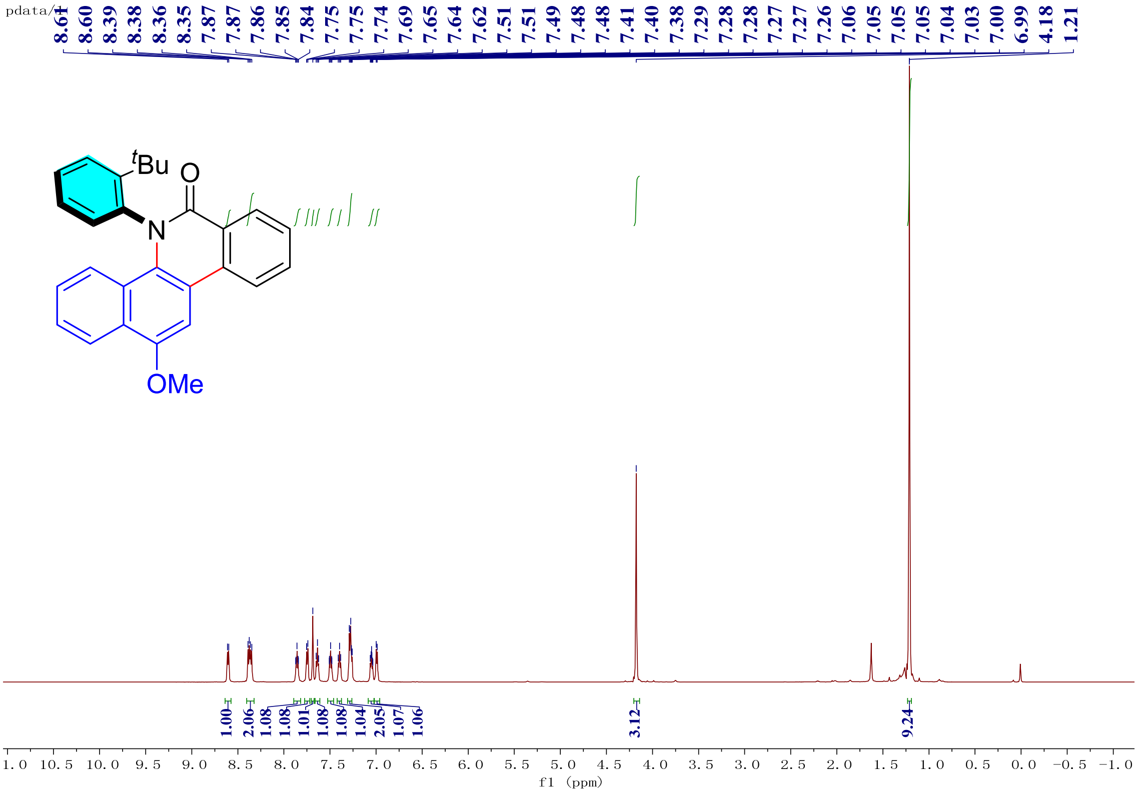

^{13}C NMR (151 MHz, Chloroform-*d*)

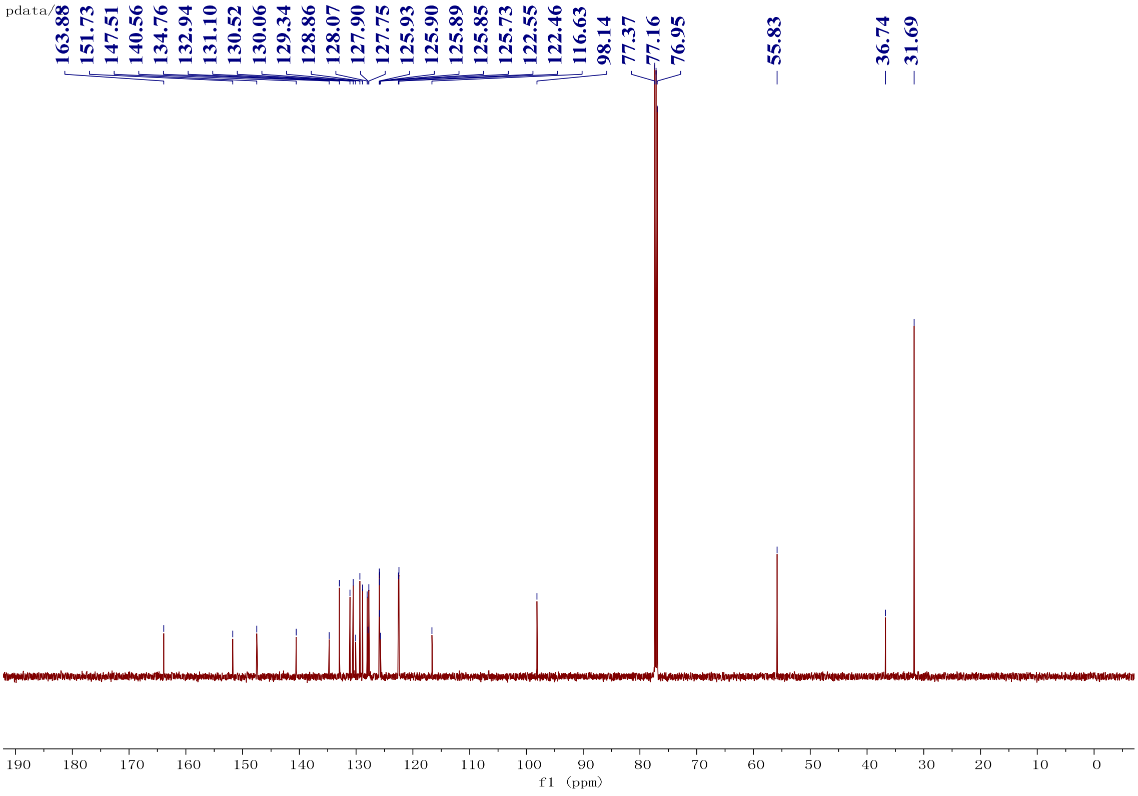

7

^{1}H NMR (600 MHz, Chloroform-*d*)

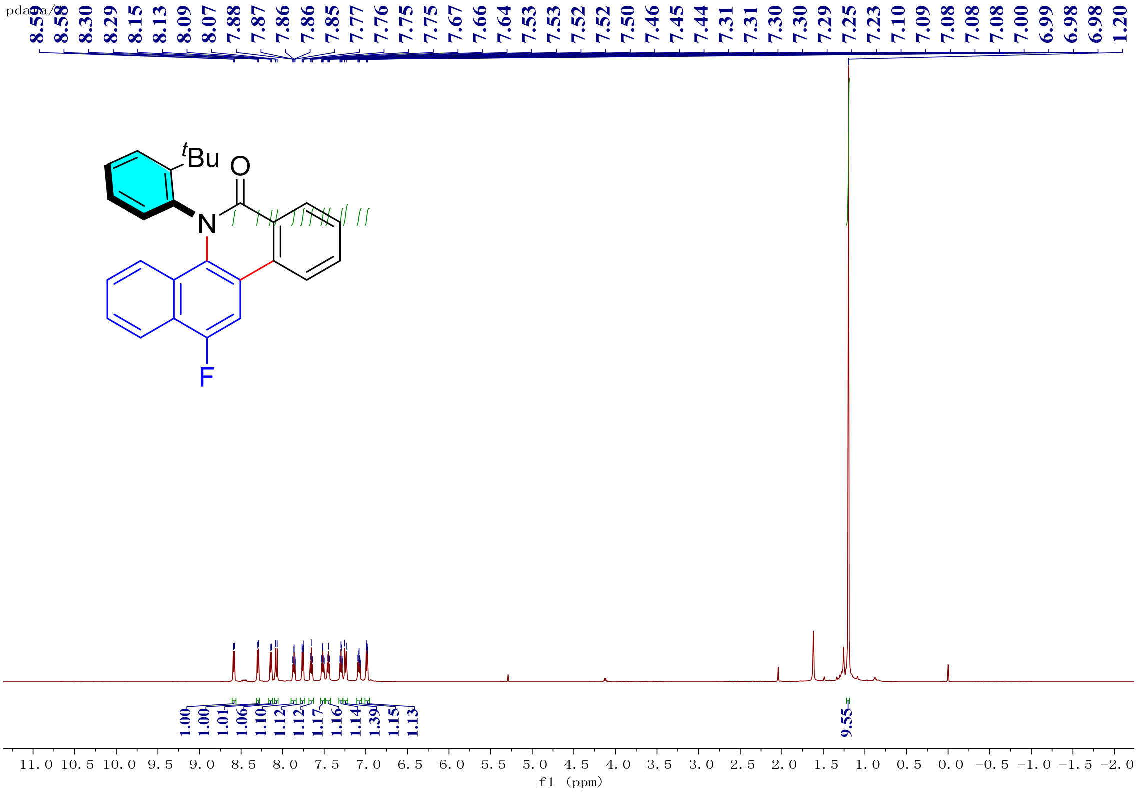

^{13}C NMR (151 MHz, Chloroform-*d*)

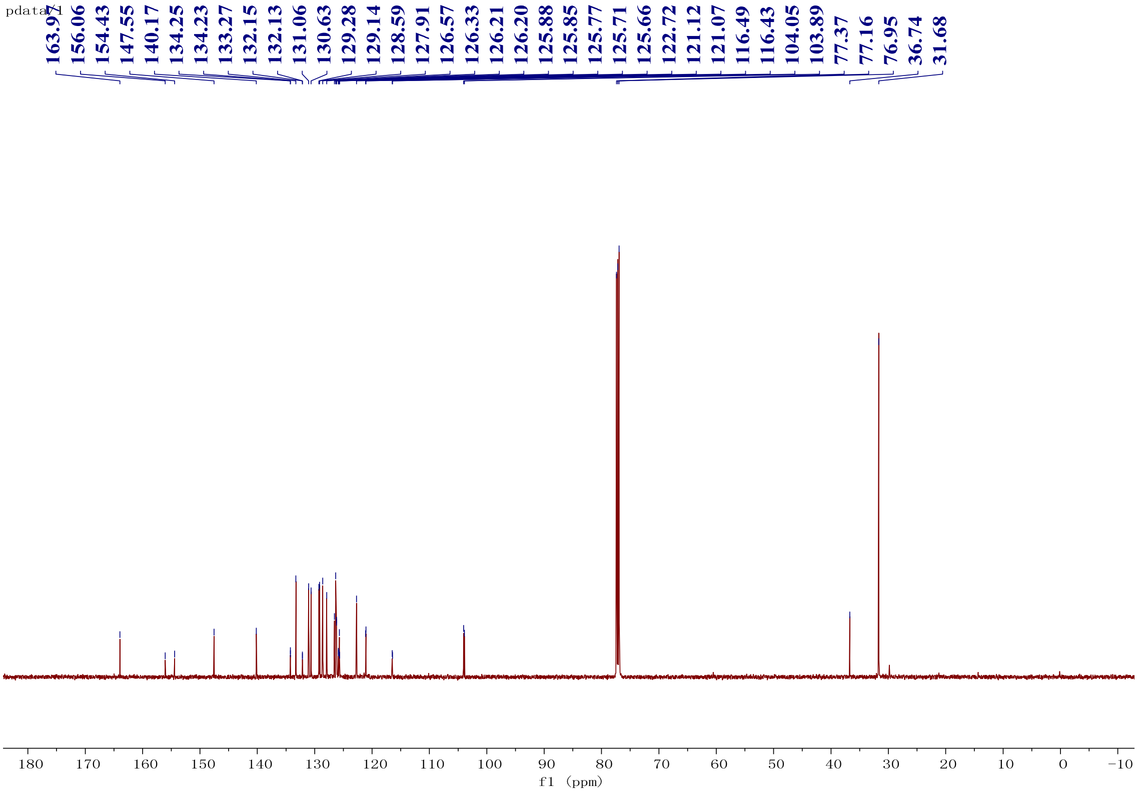

^{19}F NMR (565 MHz, Chloroform-*d*)

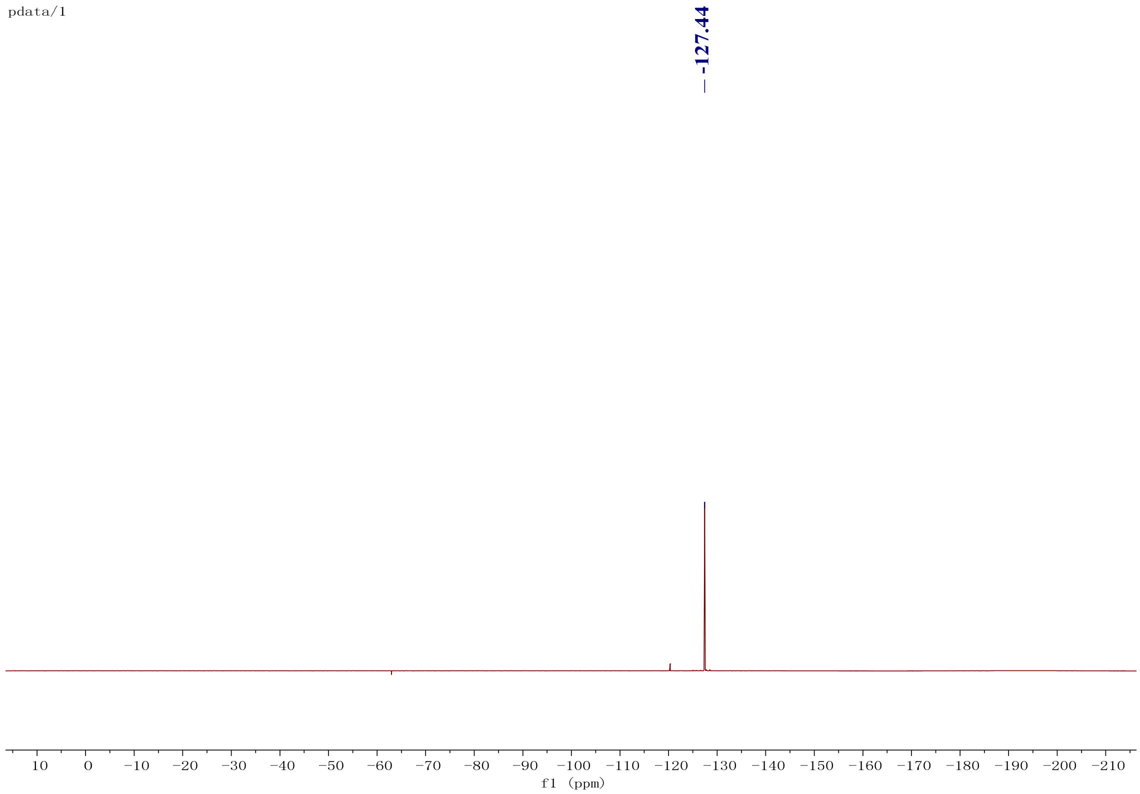

8

^{1}H NMR (600 MHz, Chloroform-*d*)

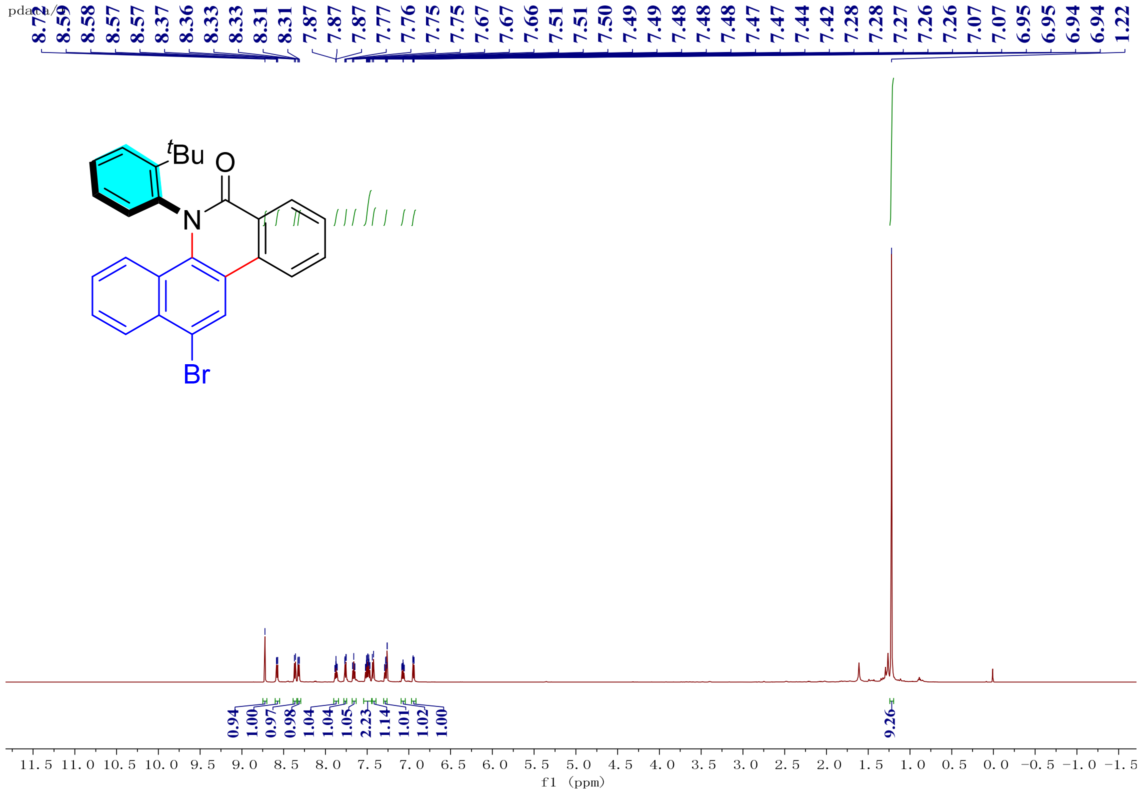

^{13}C NMR (151 MHz, Chloroform-*d*)

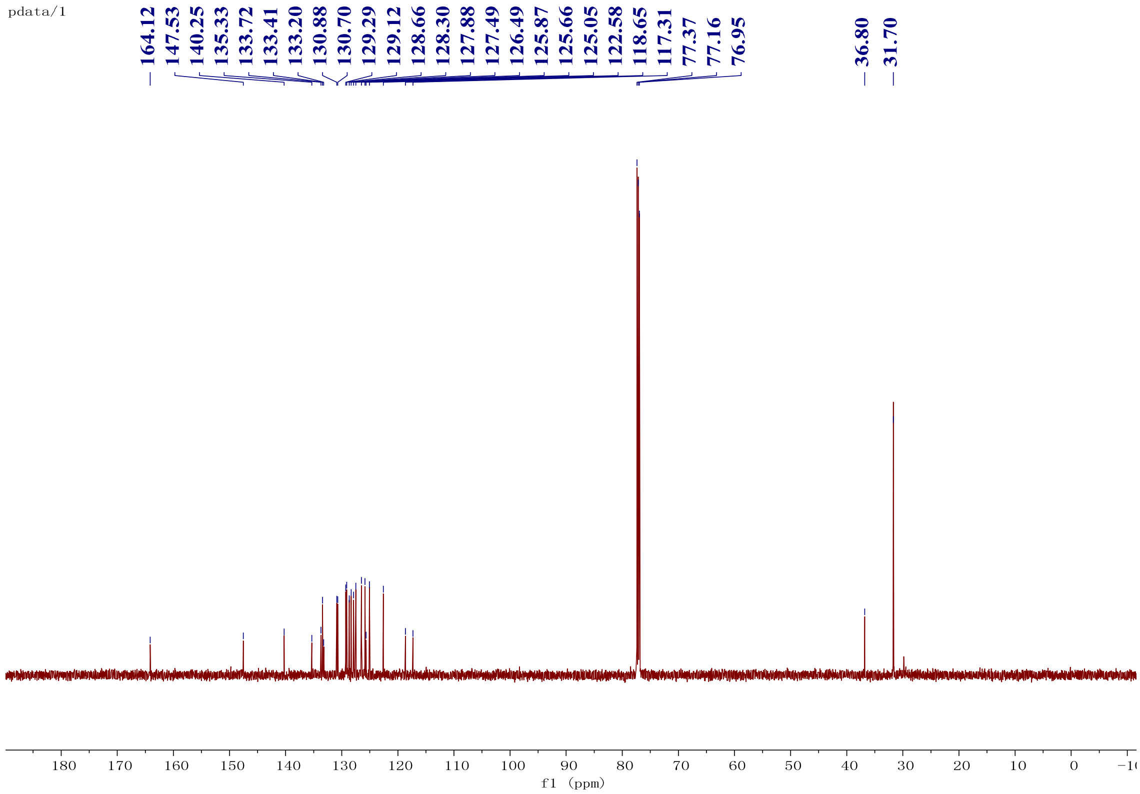

9

^{1}H NMR (600 MHz, Chloroform-*d*)

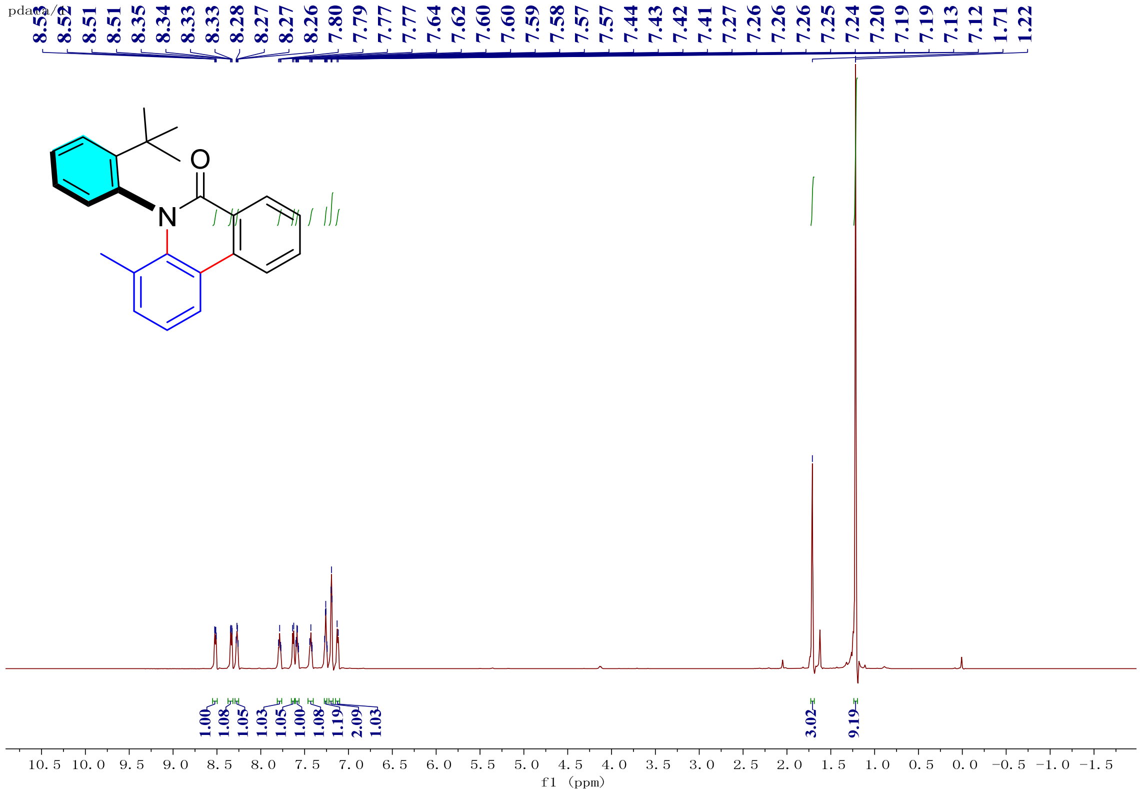

^{13}C NMR (151 MHz, Chloroform-*d*)

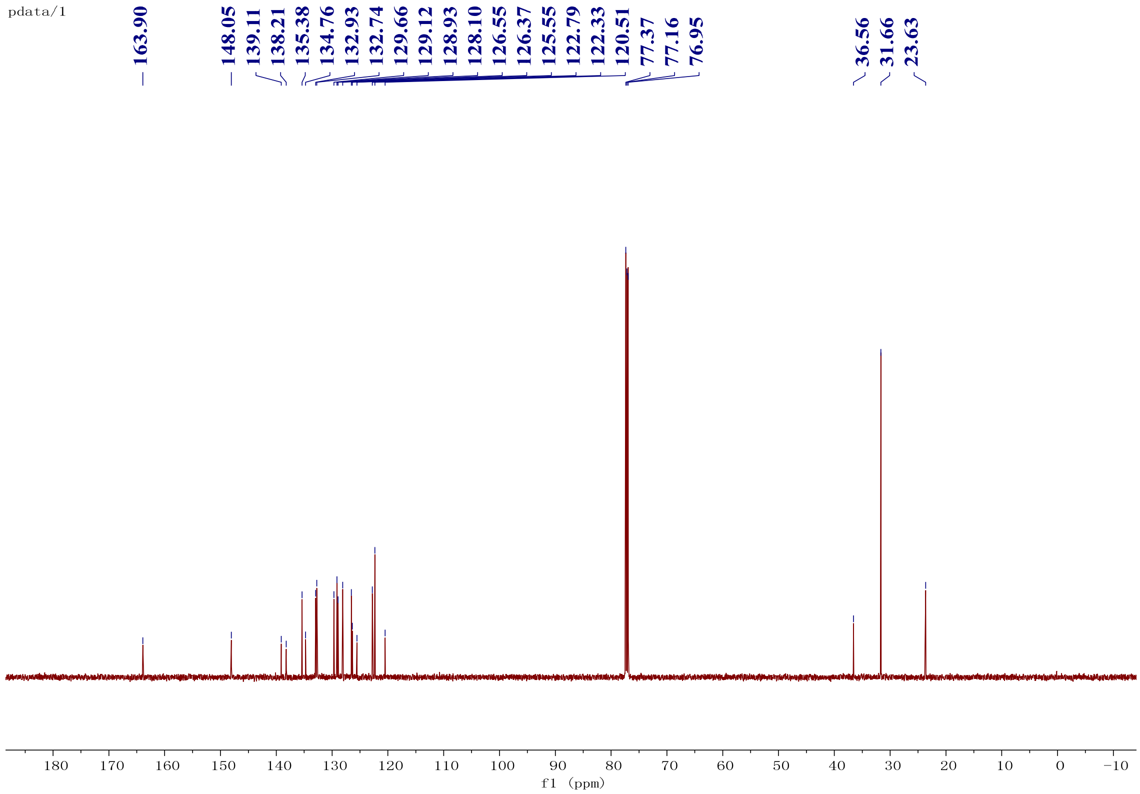

10

^{1}H NMR (400 MHz, Chloroform-*d*)

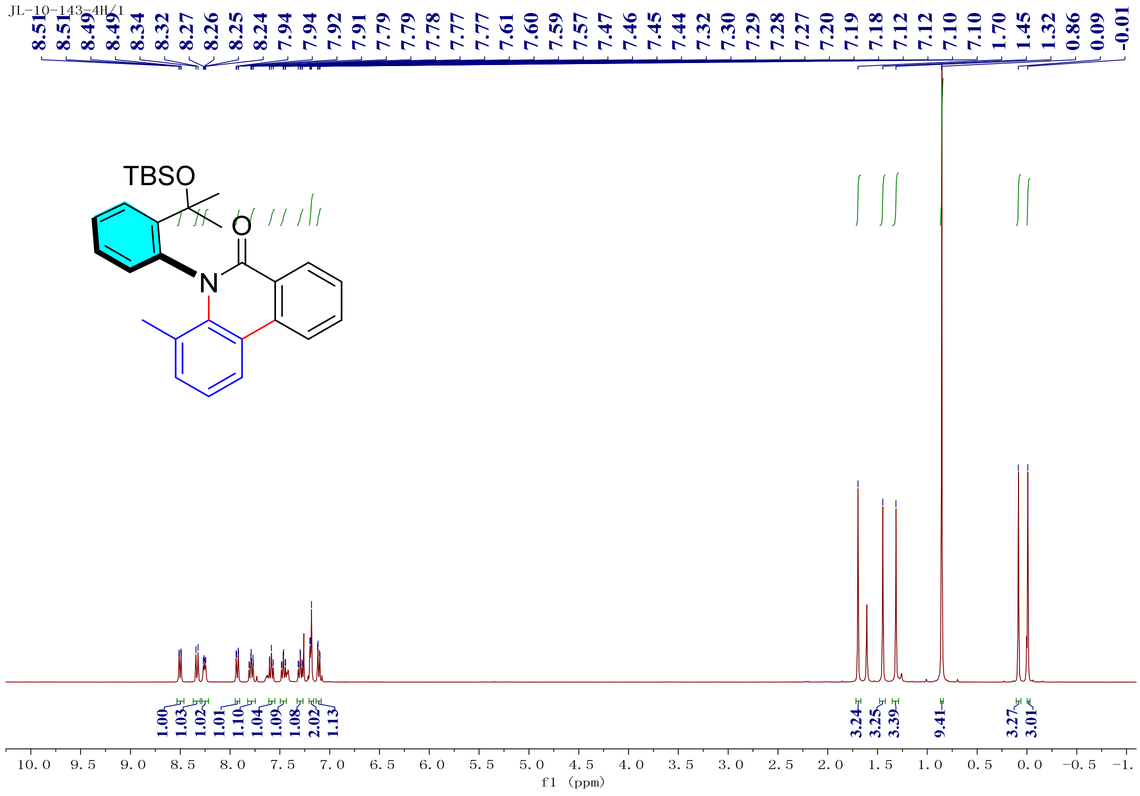

^{13}C NMR (101 MHz, Chloroform-*d*)

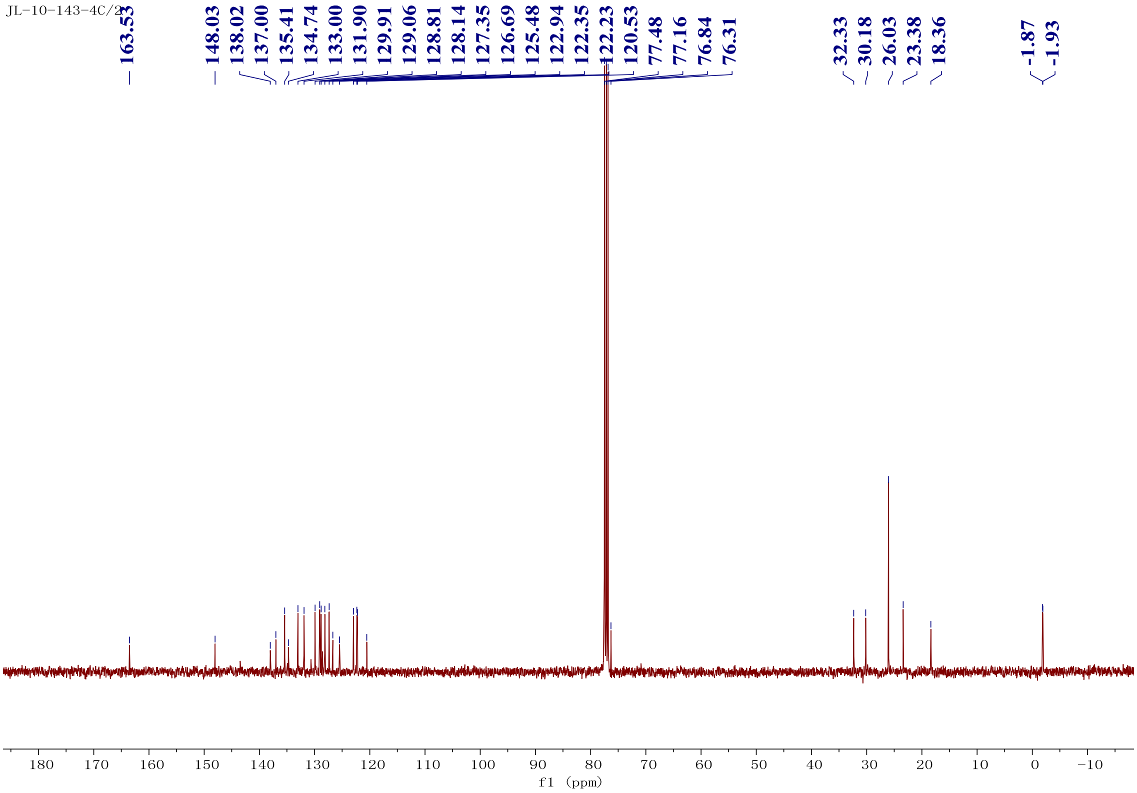

11

^{1}H NMR (400 MHz, Chloroform-*d*)

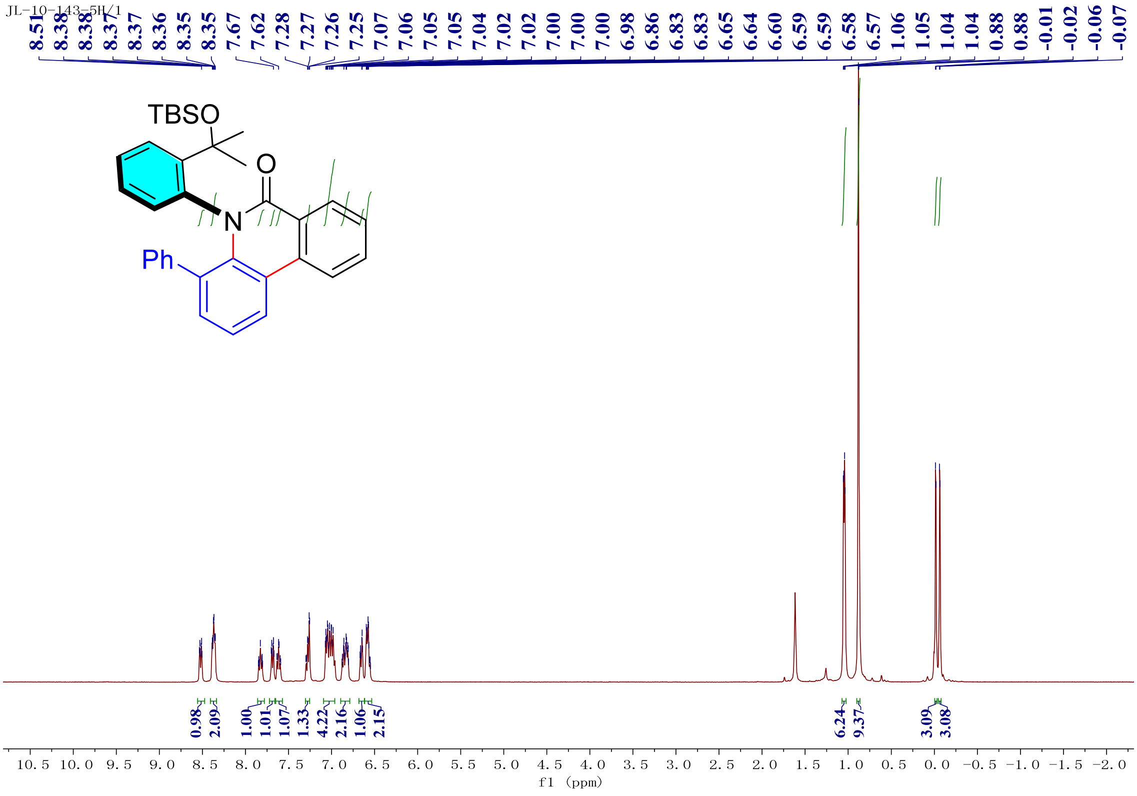

^{13}C NMR (101 MHz, Chloroform-*d*)

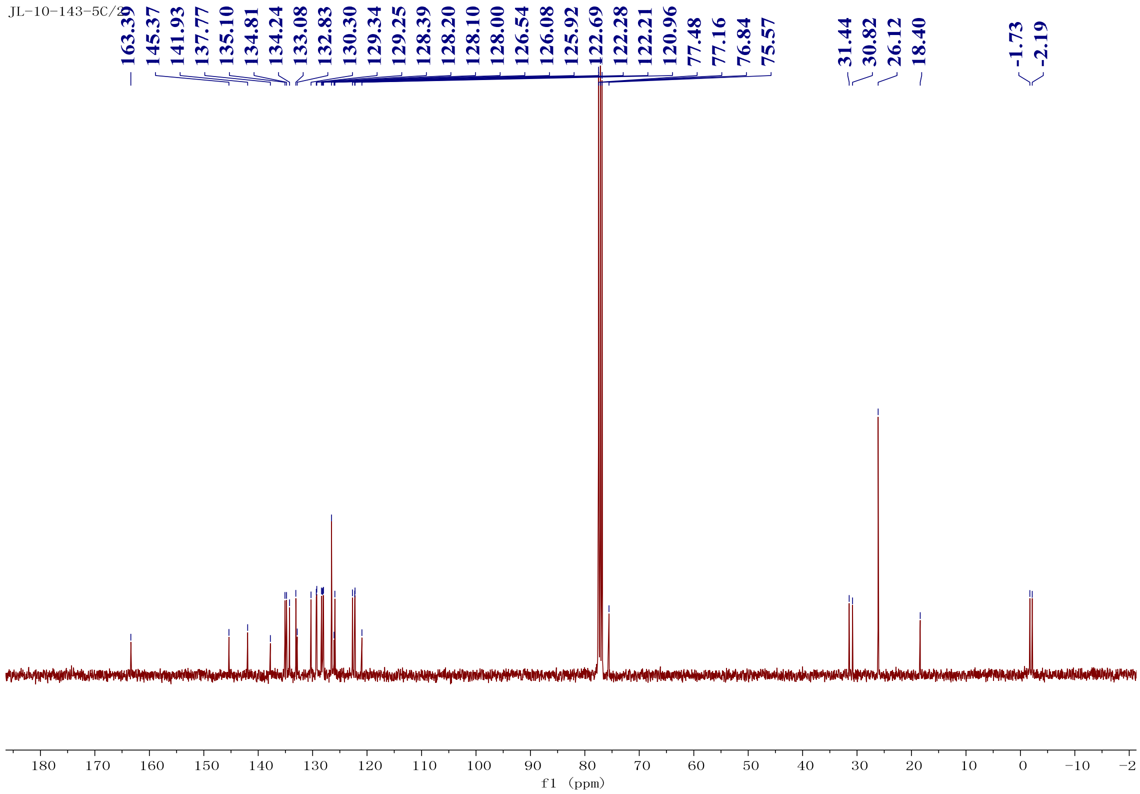

12

^{1}H NMR (400 MHz, Chloroform-*d*)

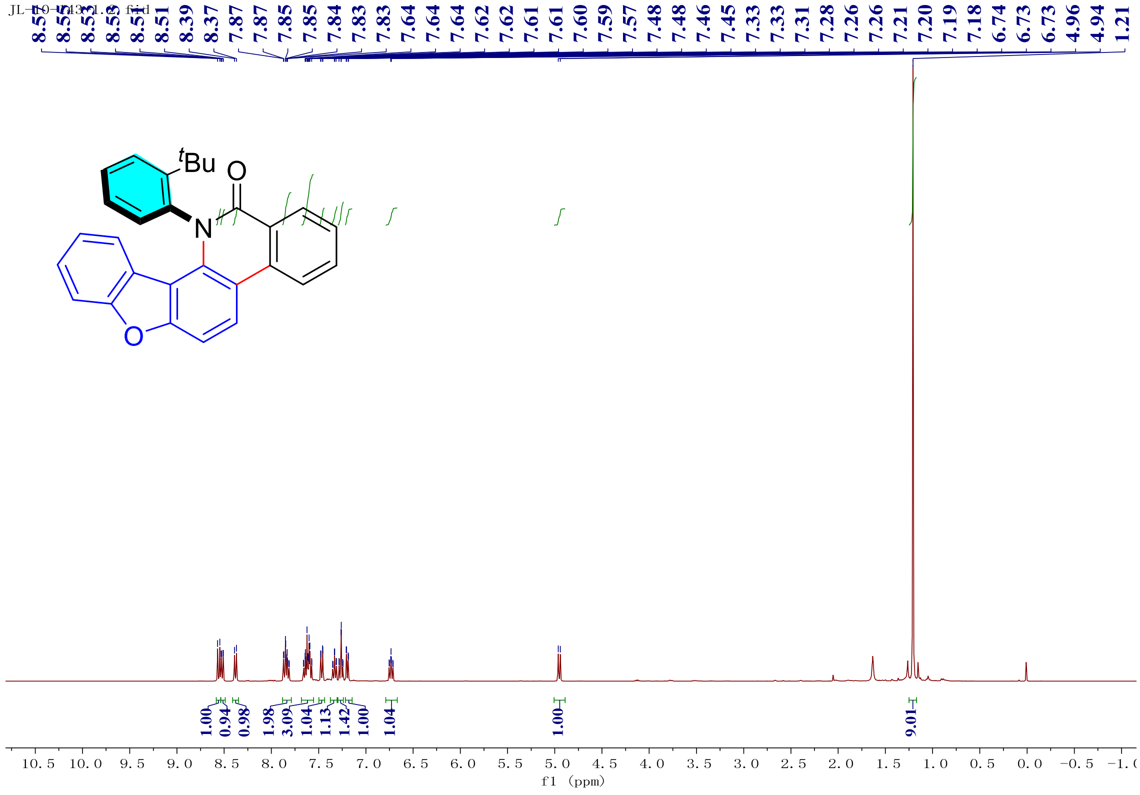

^{13}C NMR (101 MHz, Chloroform-*d*)

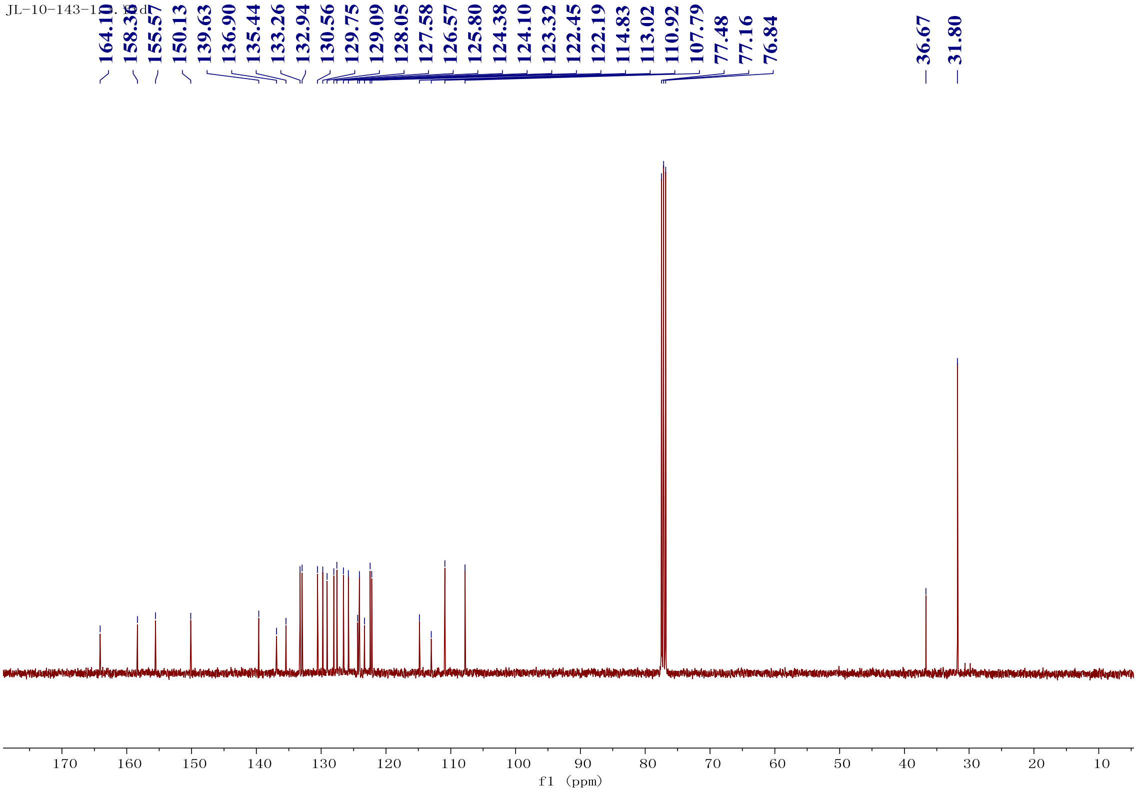

13

^{1}H NMR (600 MHz, Chloroform-*d*)

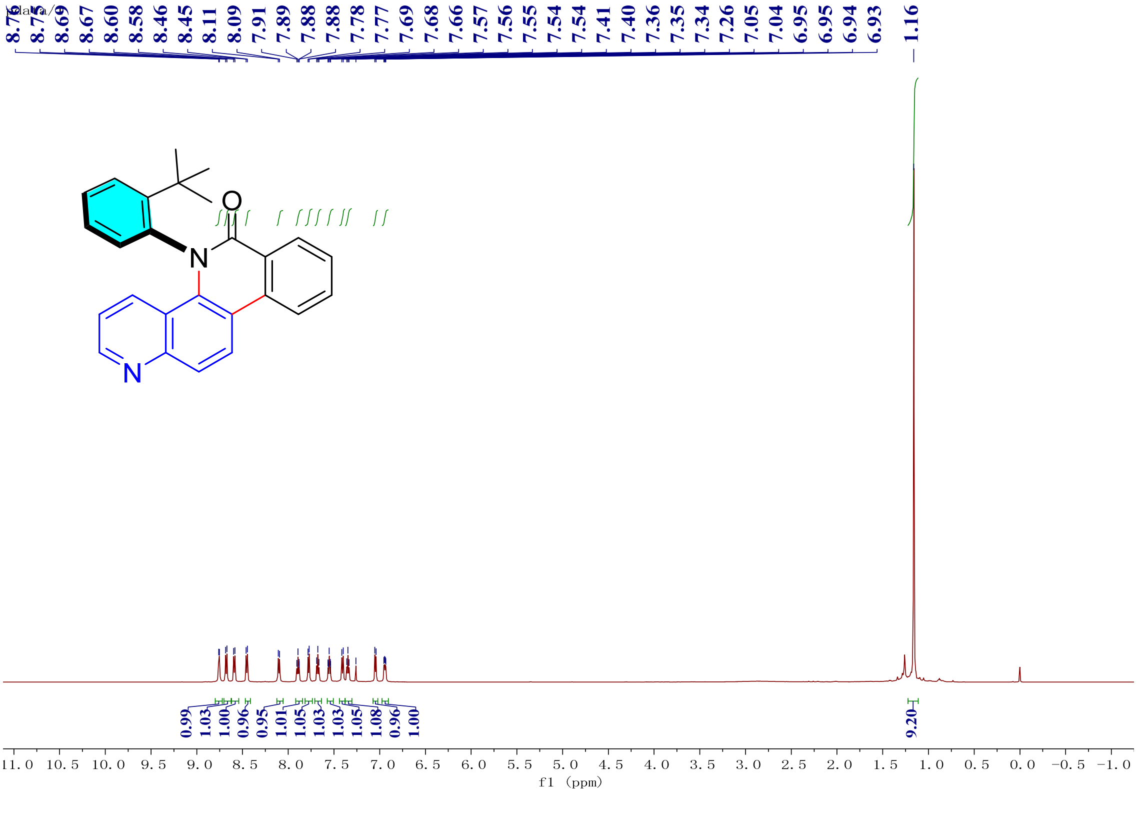

^{13}C NMR (151 MHz, Chloroform-*d*)

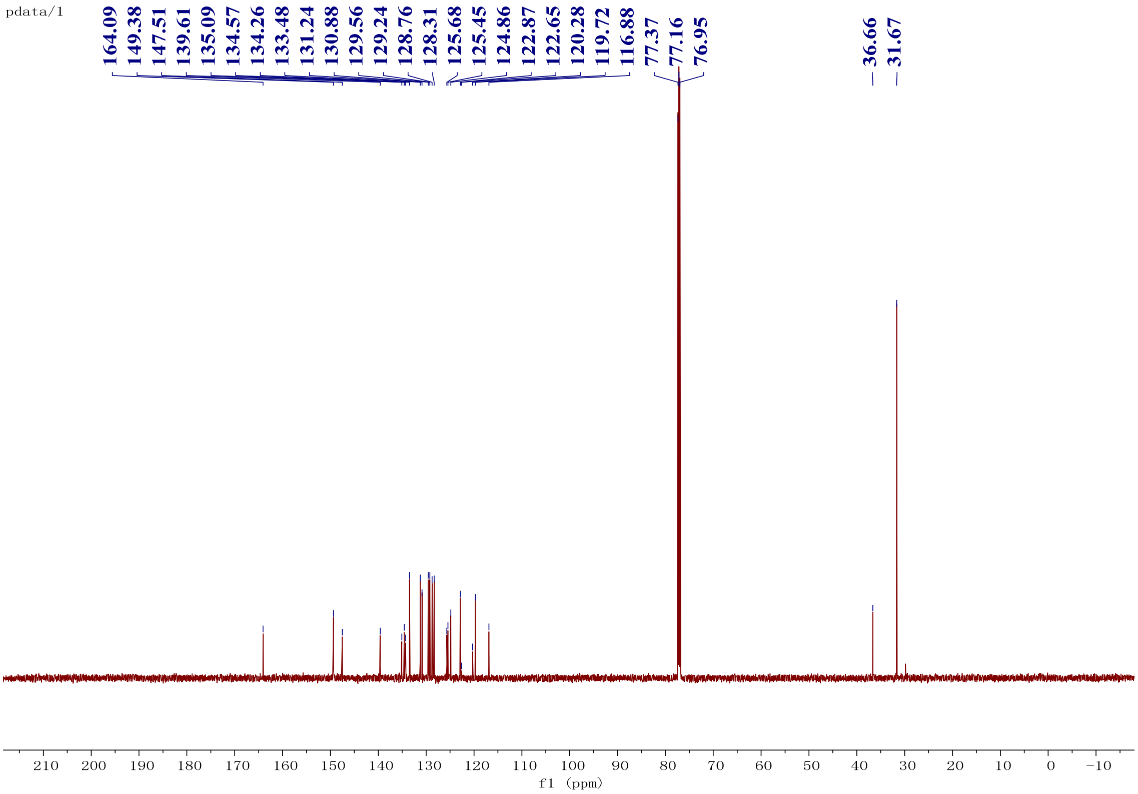

14

^{1}H NMR (400 MHz, Chloroform-*d*)

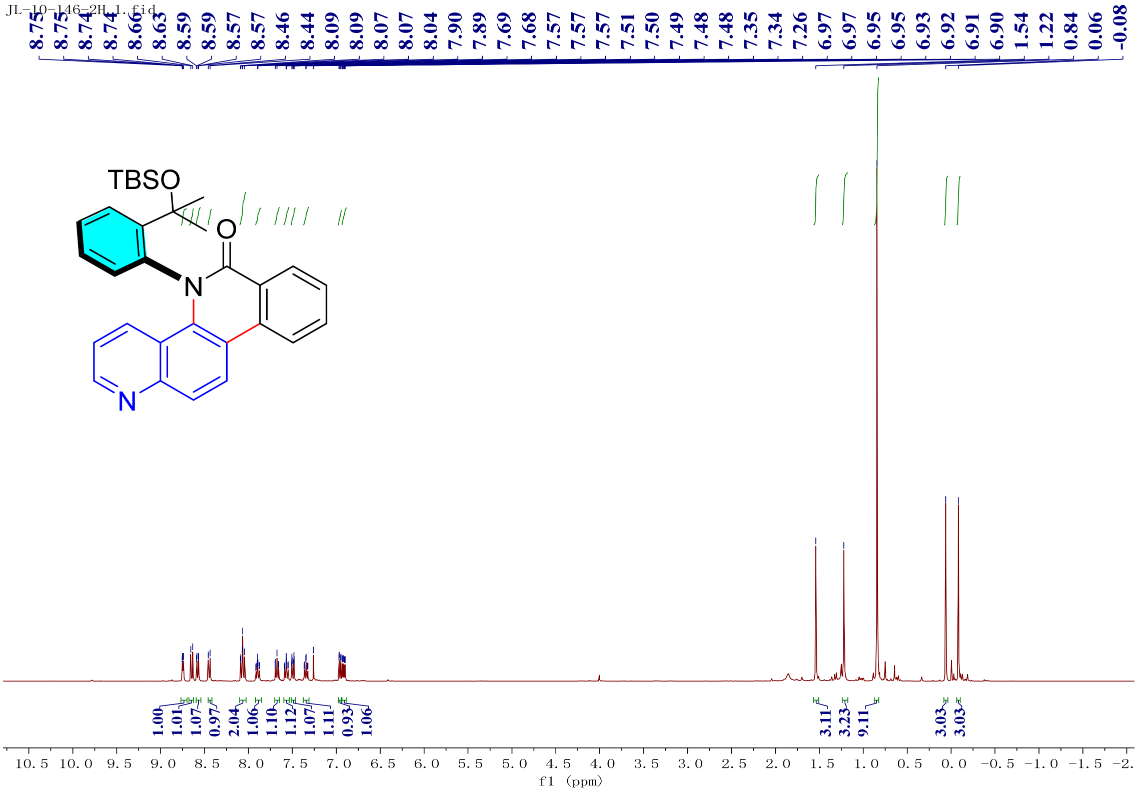

^{13}C NMR (101 MHz, Chloroform-*d*)

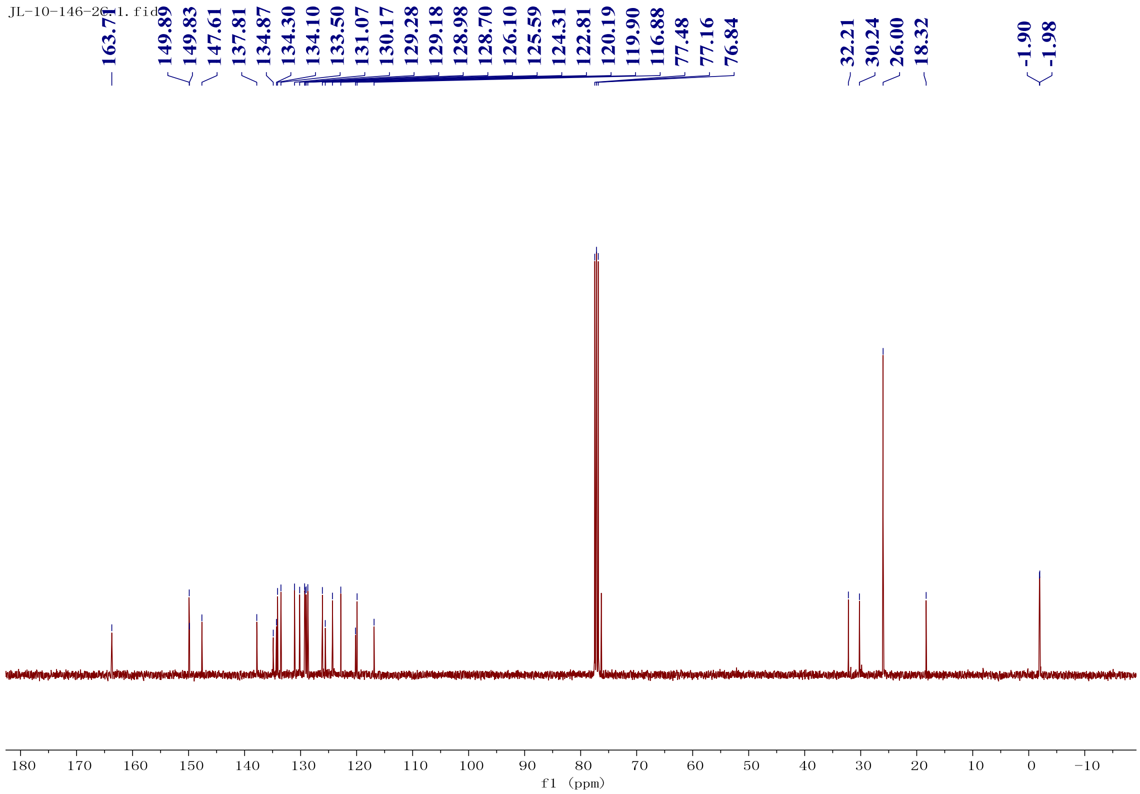

15

^{1}H NMR (600 MHz, Chloroform-*d*)

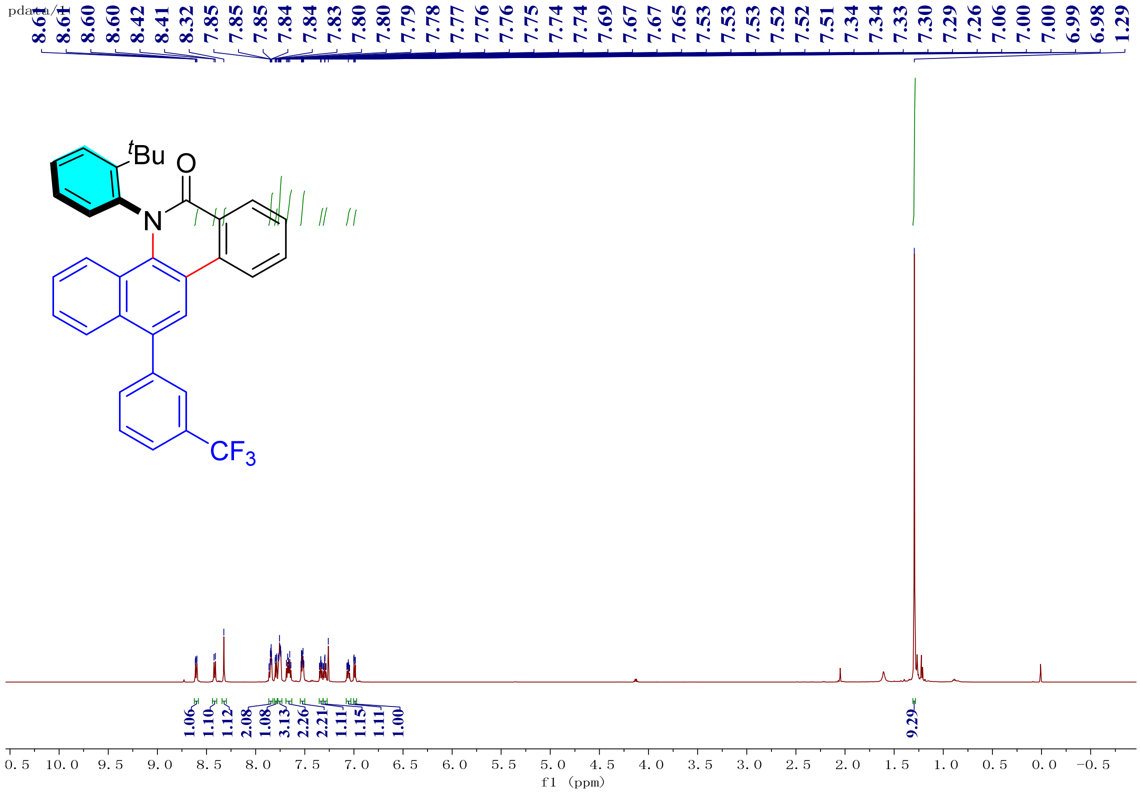

^{13}C NMR (151 MHz, Chloroform-*d*)

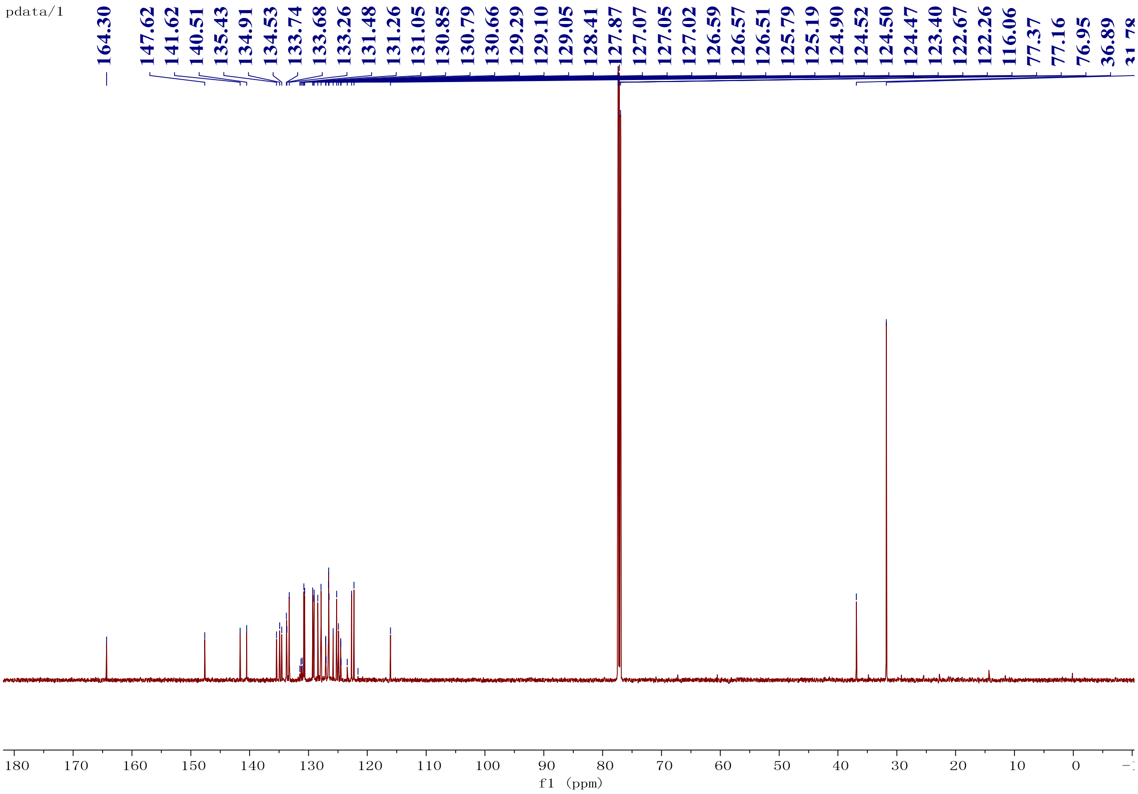

^{19}F NMR (565 MHz, Chloroform-*d*)

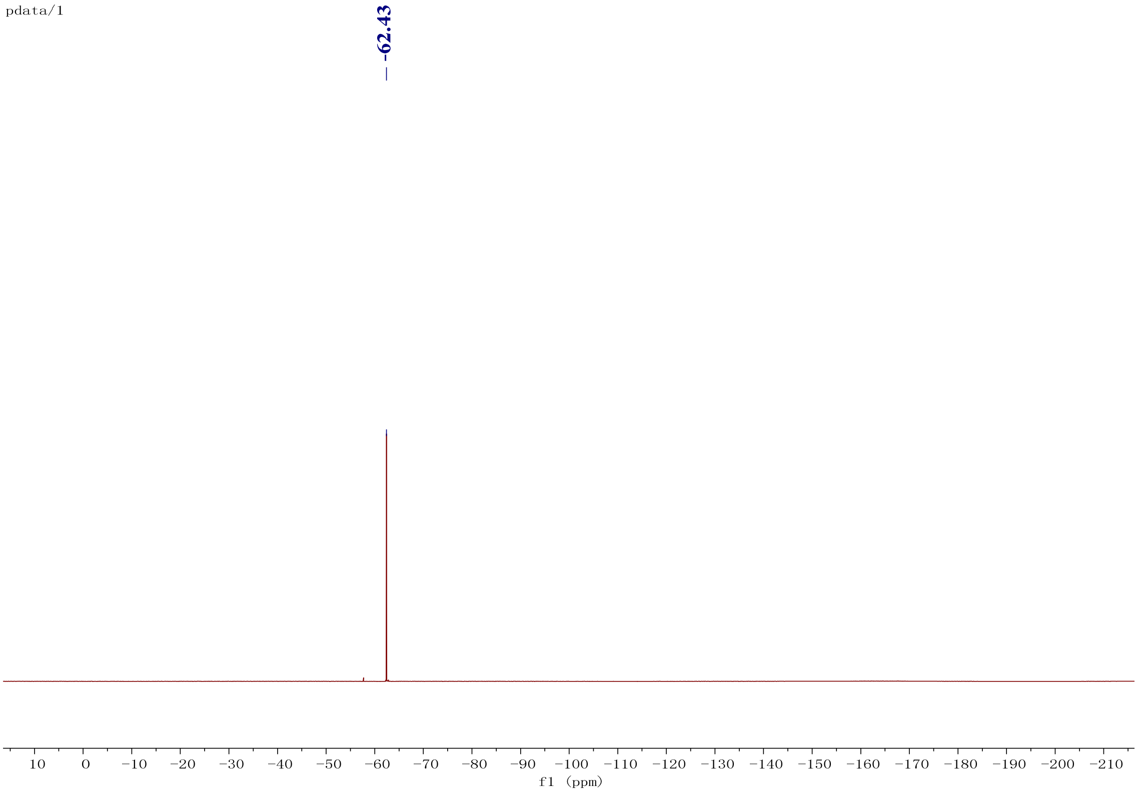

16

^{1}H NMR (600 MHz, Chloroform-*d*)

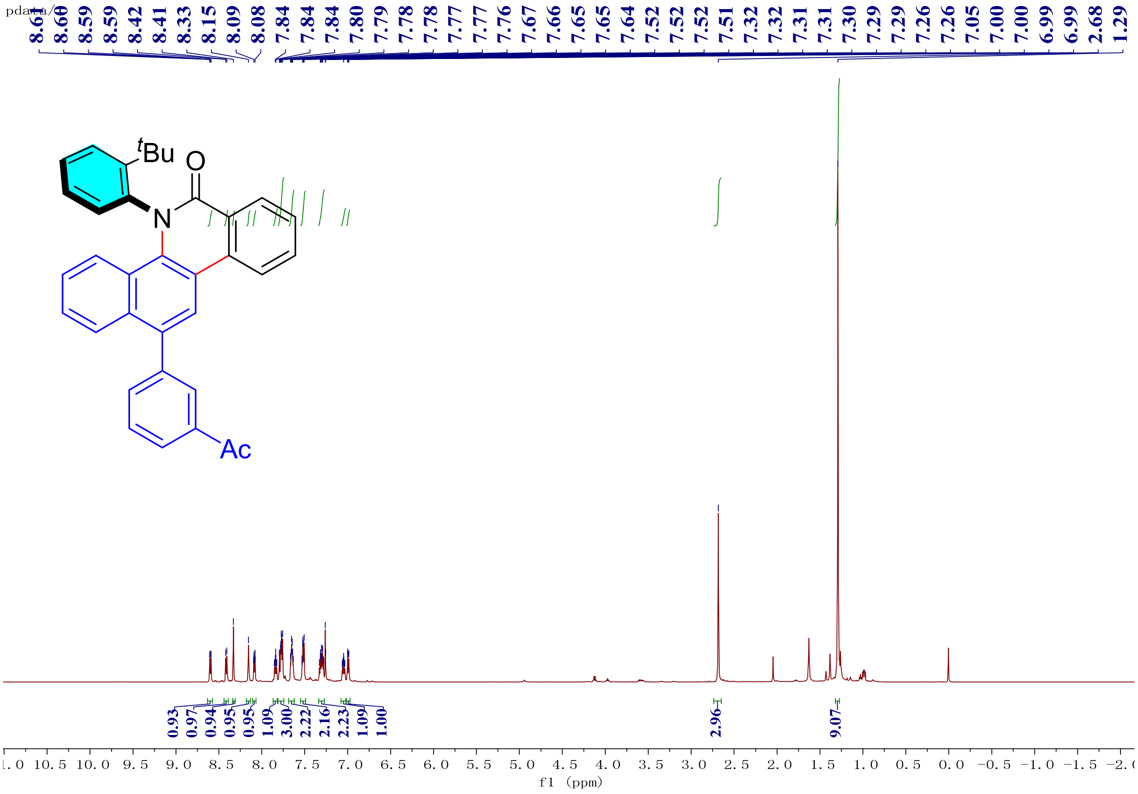

^{13}C NMR (151 MHz, Chloroform-*d*)

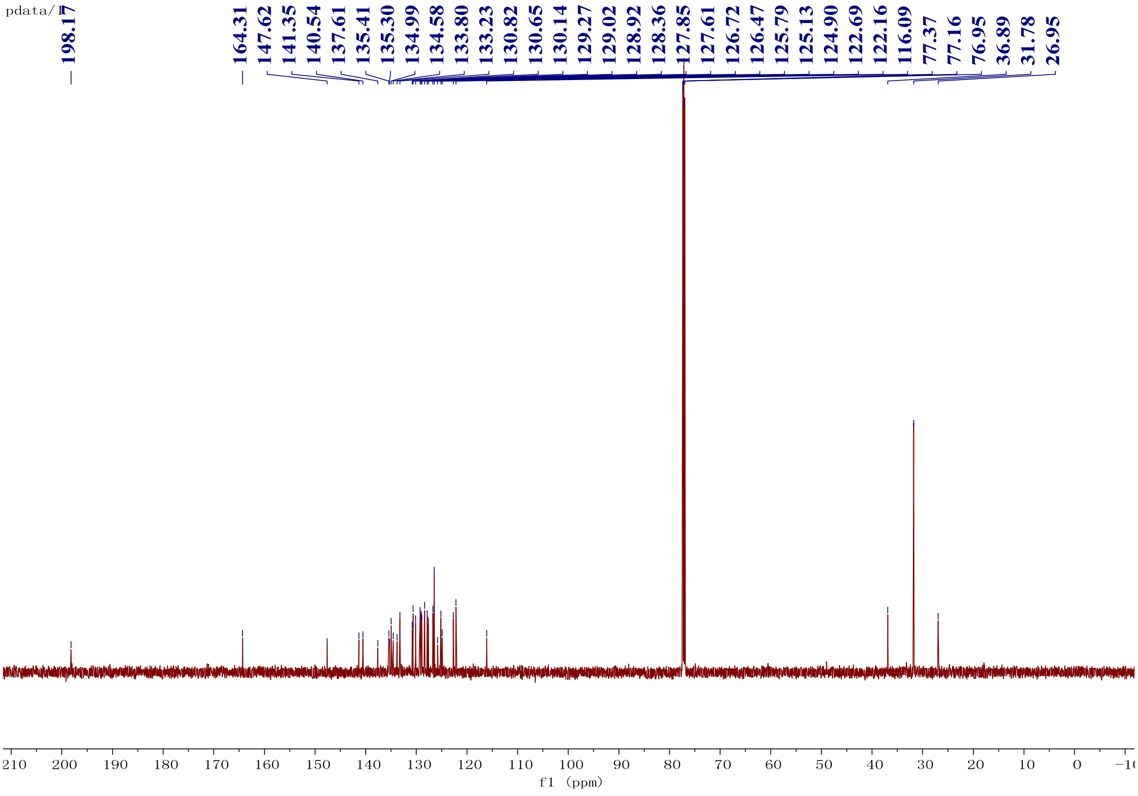

17

^{1}H NMR (600 MHz, Chloroform-*d*)

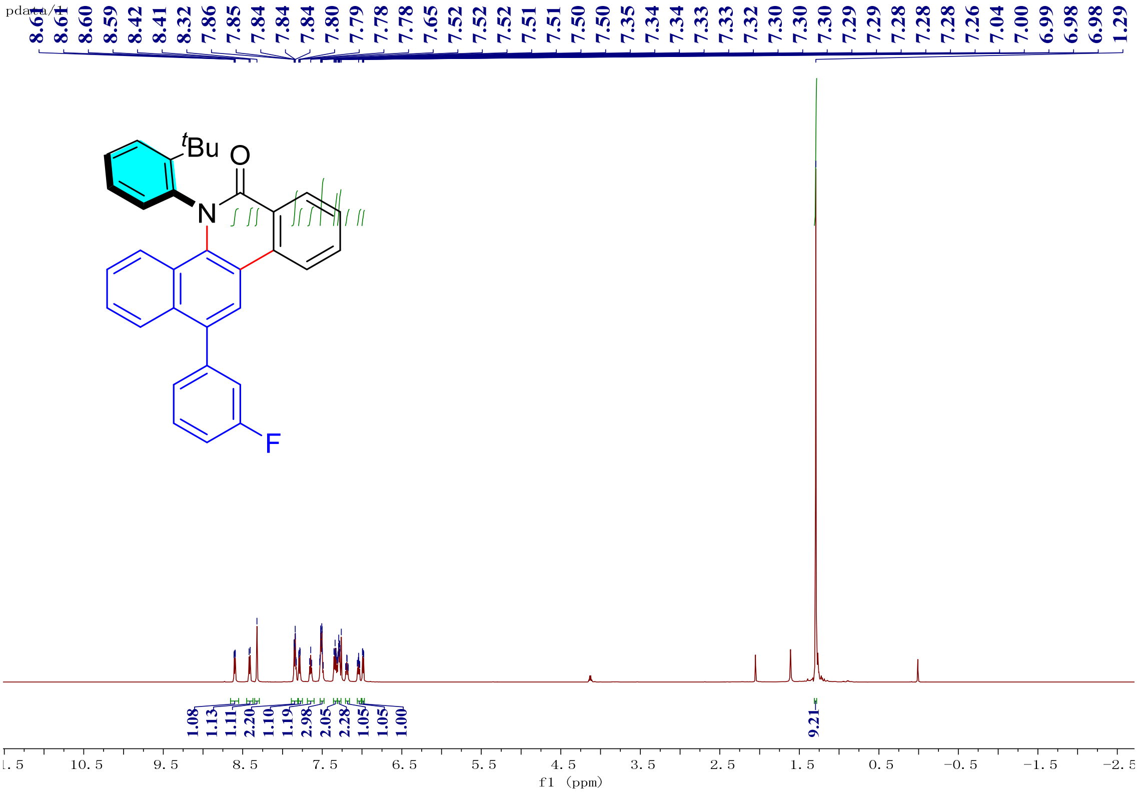

^{13}C NMR (151 MHz, Chloroform-*d*)

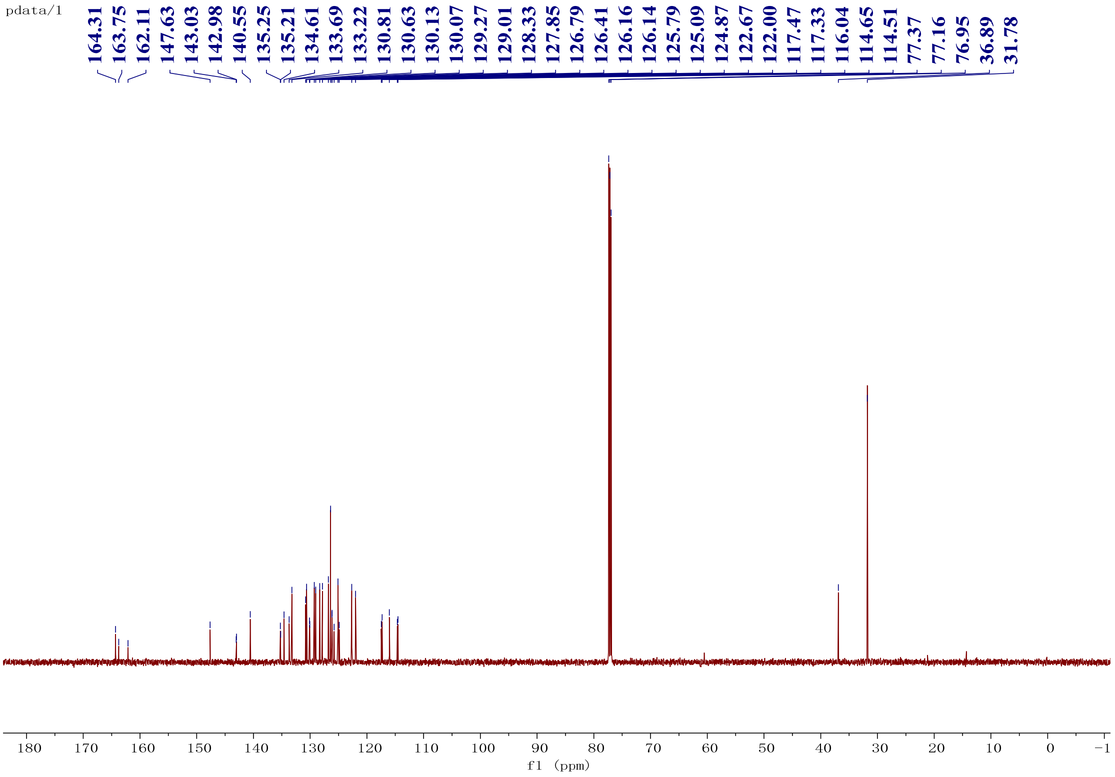

^{19}F NMR (565 MHz, Chloroform-*d*)

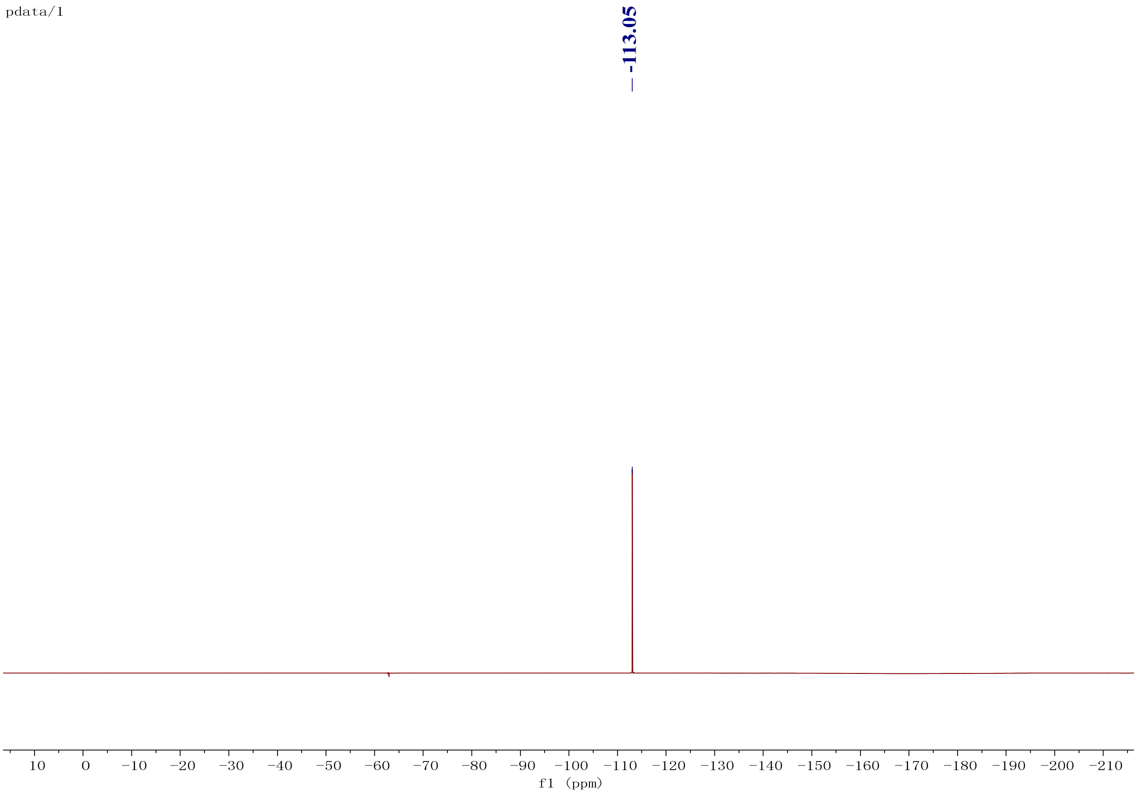

18

^{1}H NMR (600 MHz, Chloroform-*d*)

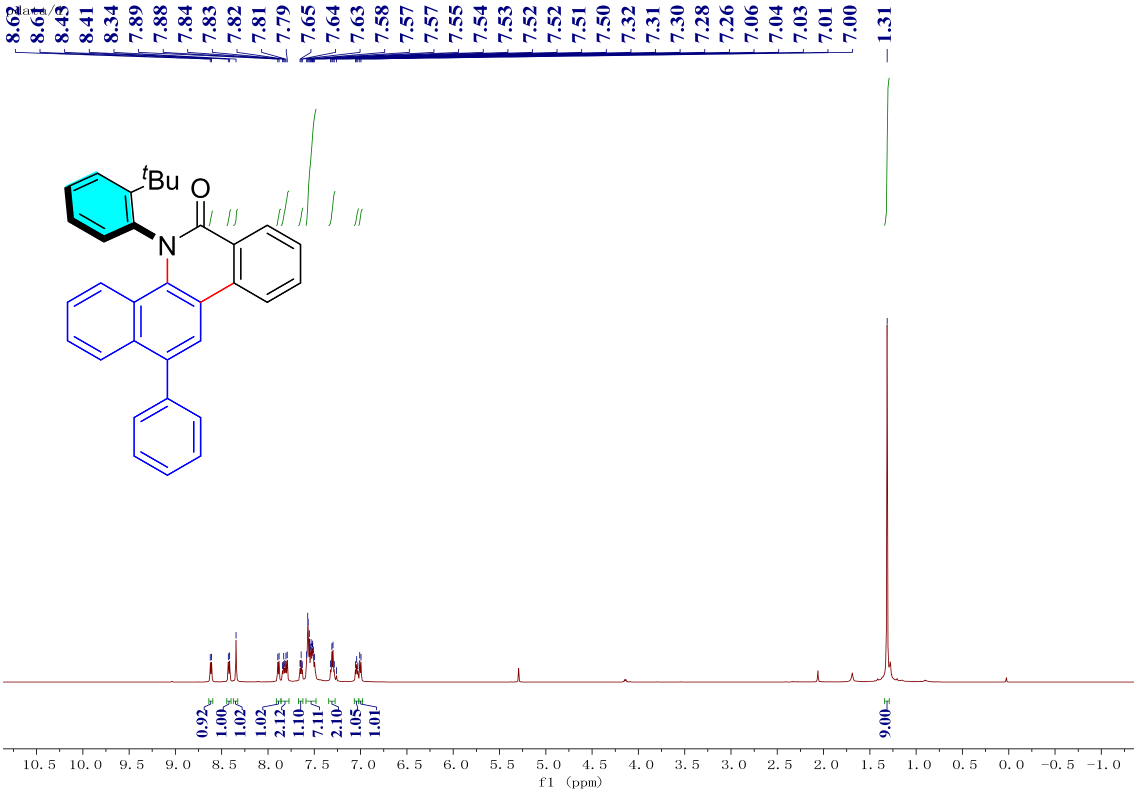

^{13}C NMR (151 MHz, Chloroform-*d*)

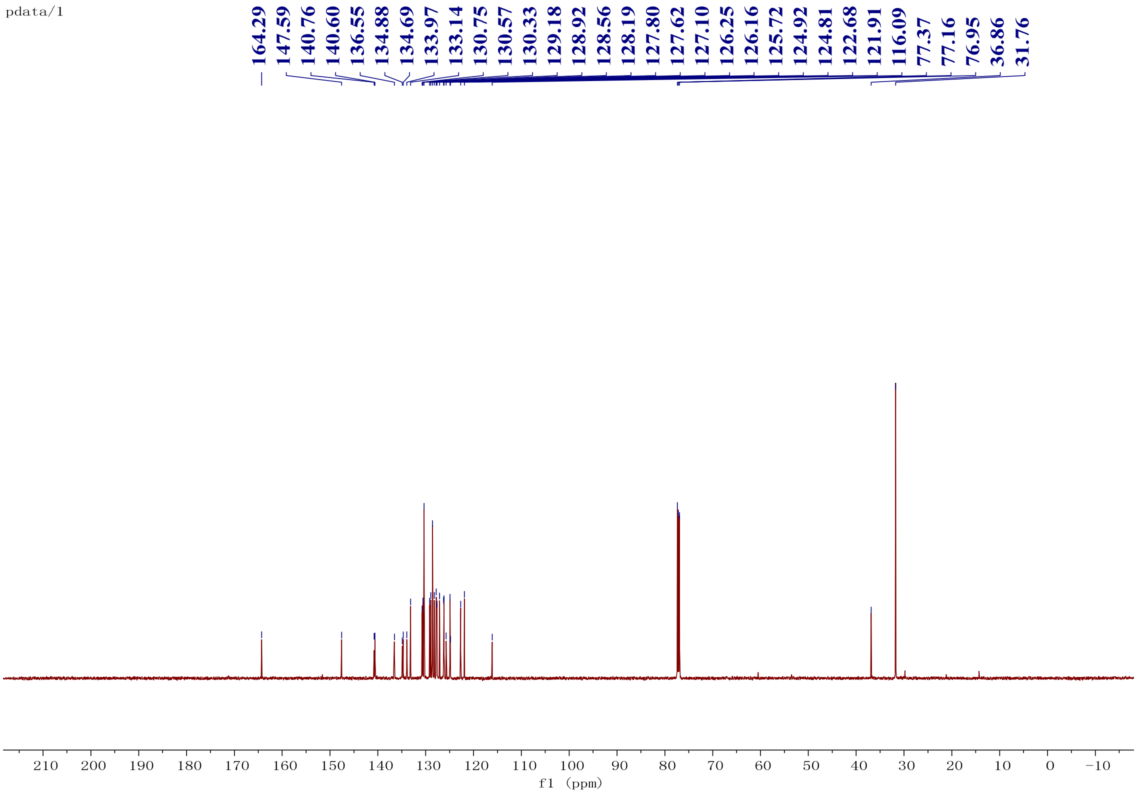

19

^{1}H NMR (600 MHz, Chloroform-*d*)

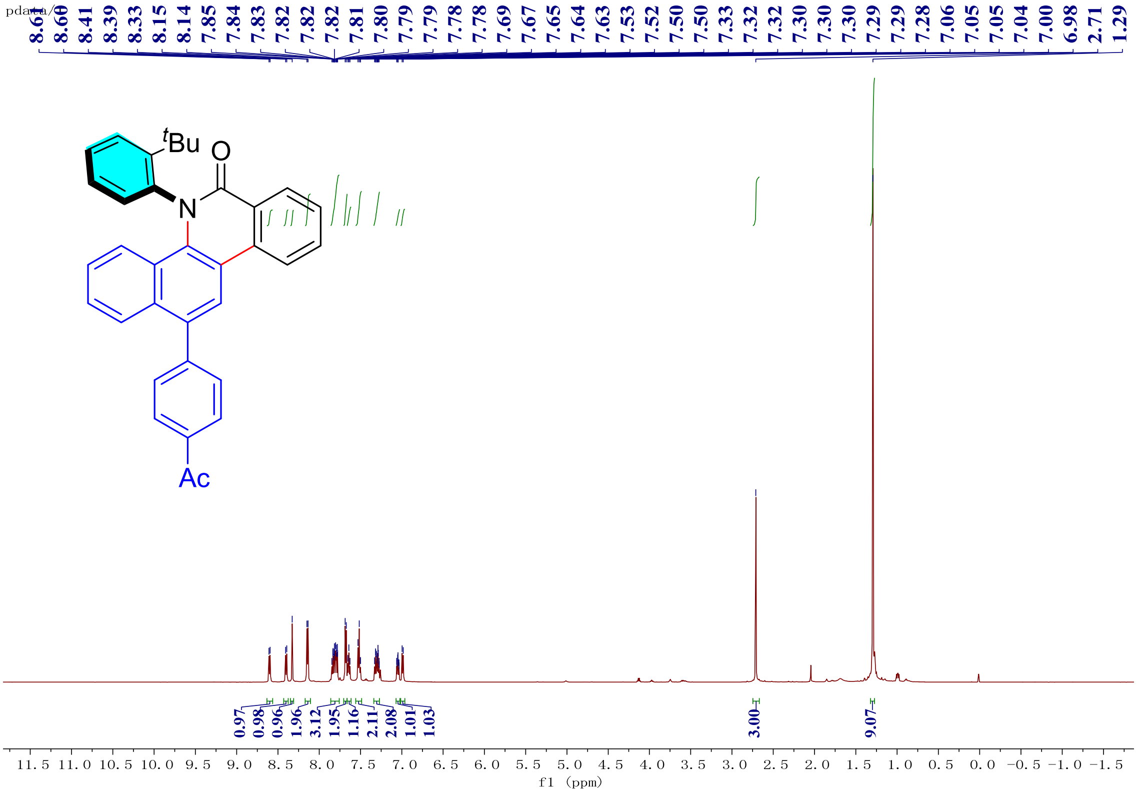

^{13}C NMR (151 MHz, Chloroform-*d*)

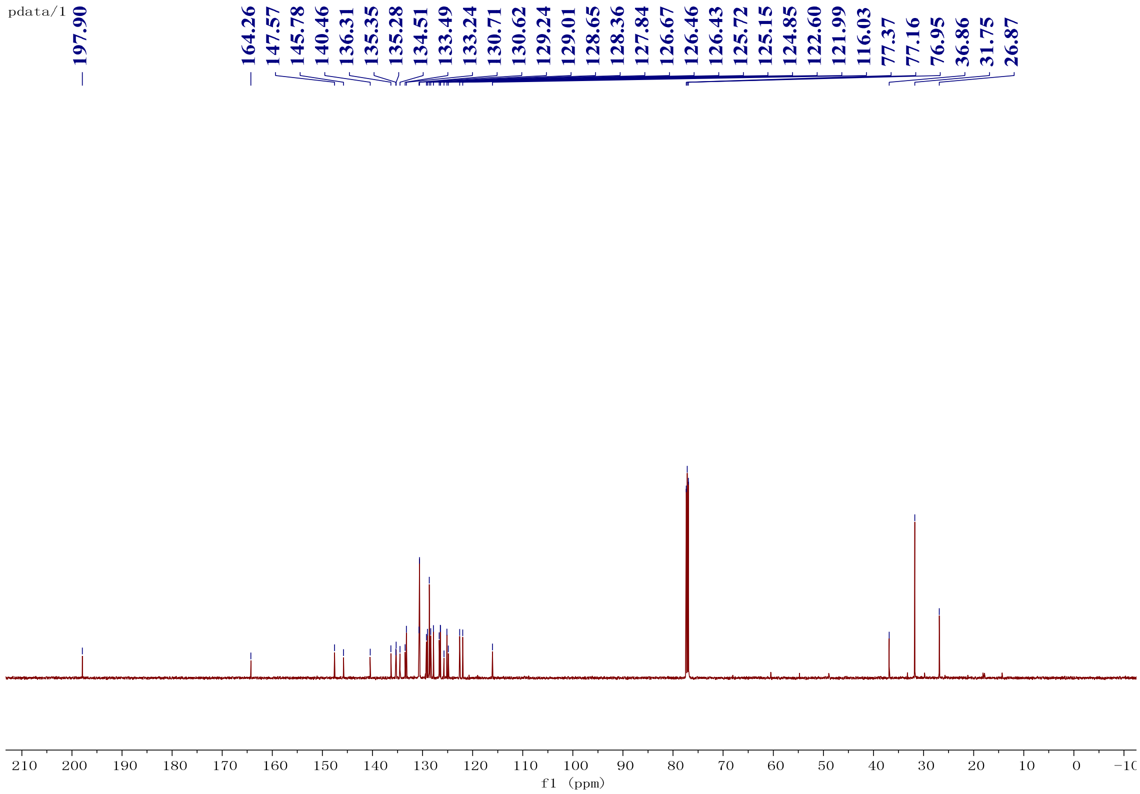

20

^{1}H NMR (600 MHz, Chloroform-*d*)

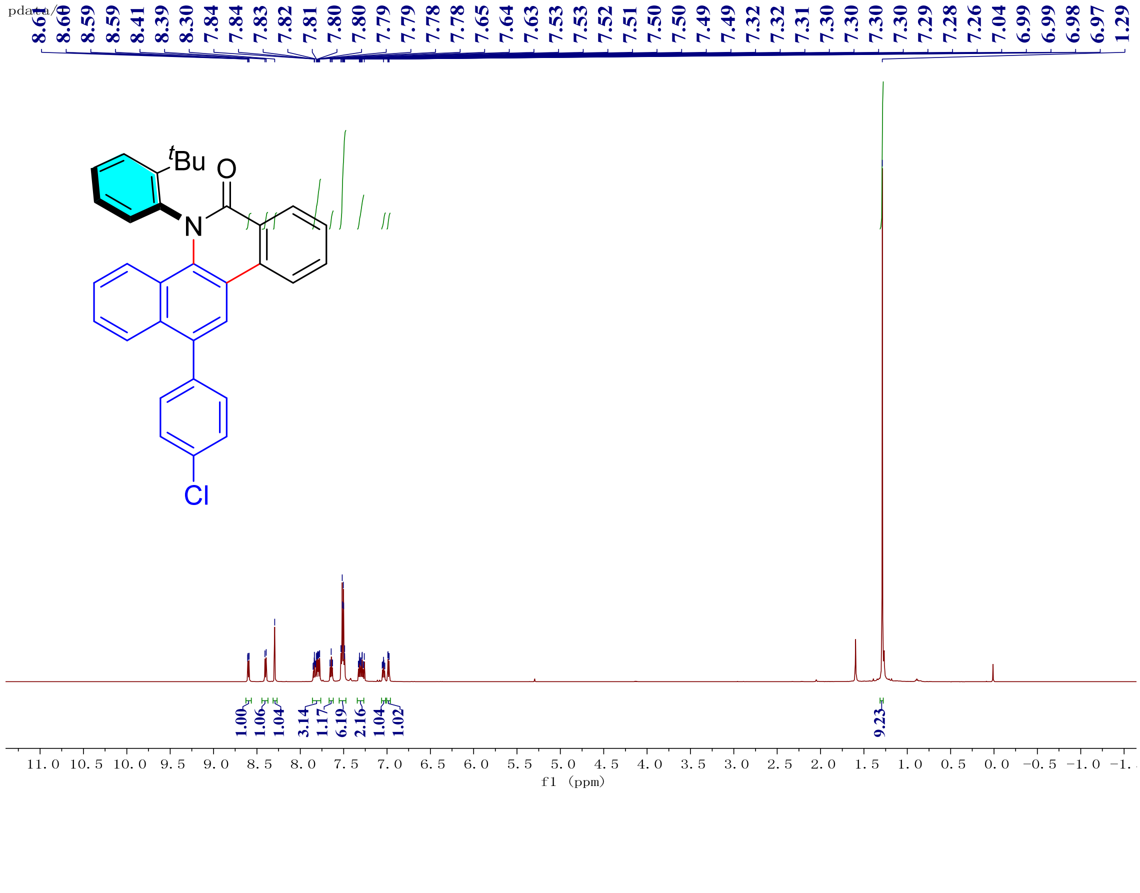

^{13}C NMR (151 MHz, Chloroform-*d*)

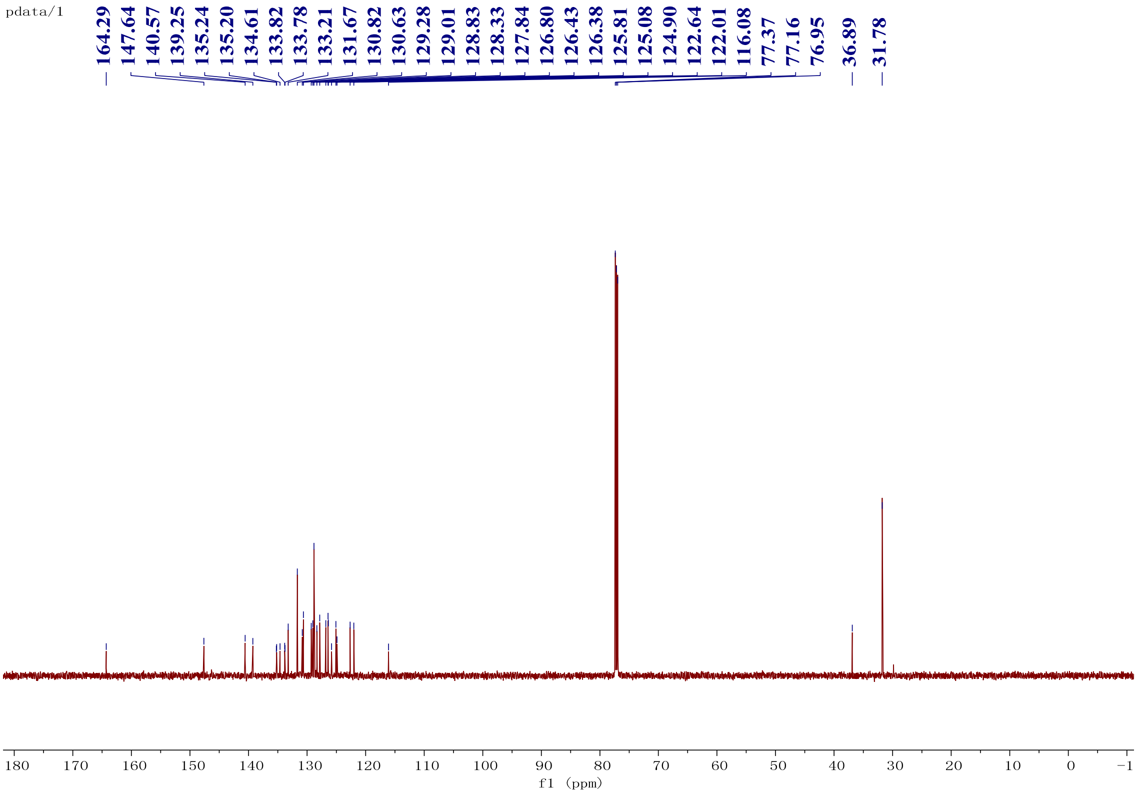

21

^{1}H NMR (600 MHz, Chloroform-*d*)

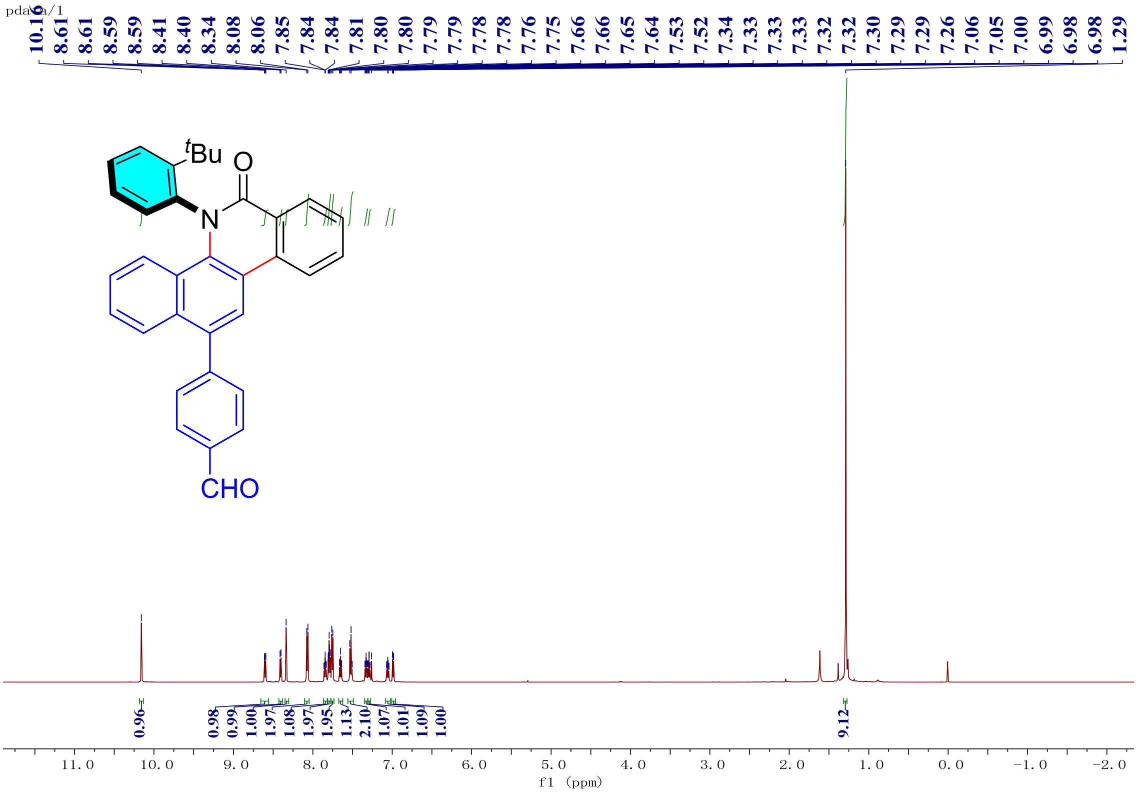

^{13}C NMR (151 MHz, Chloroform-*d*)

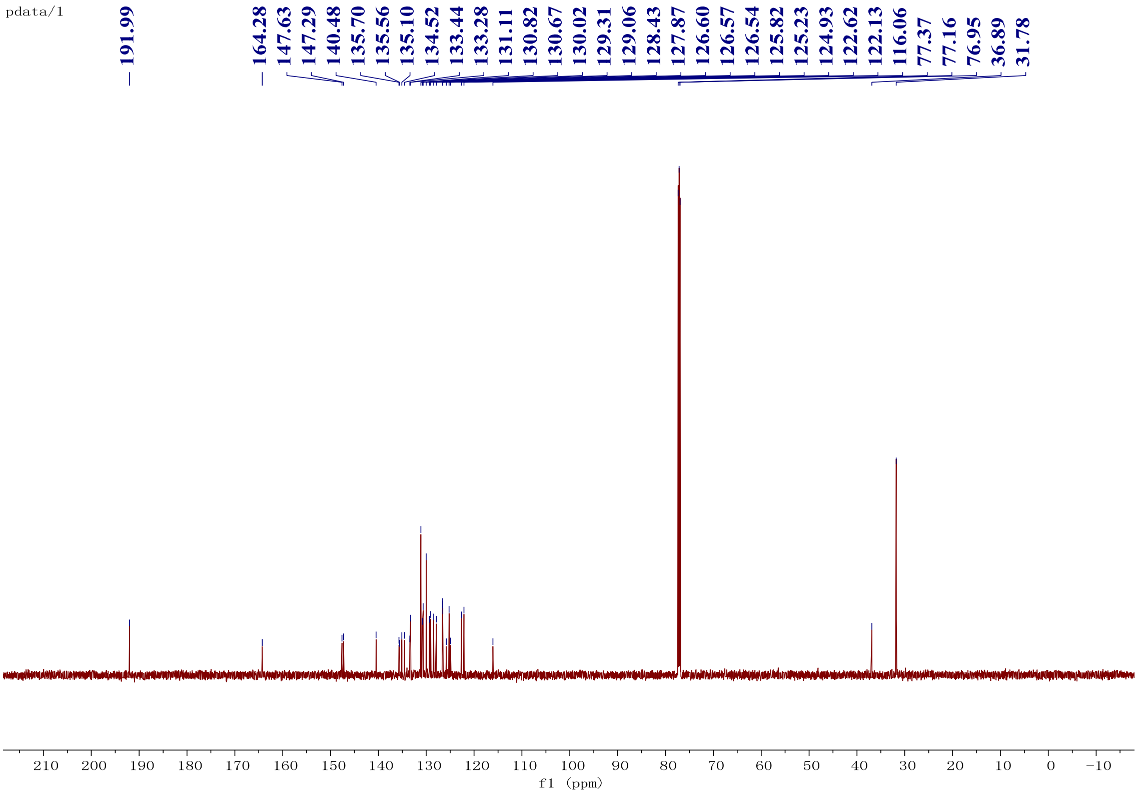

22

^{1}H NMR (600 MHz, Chloroform-*d*)

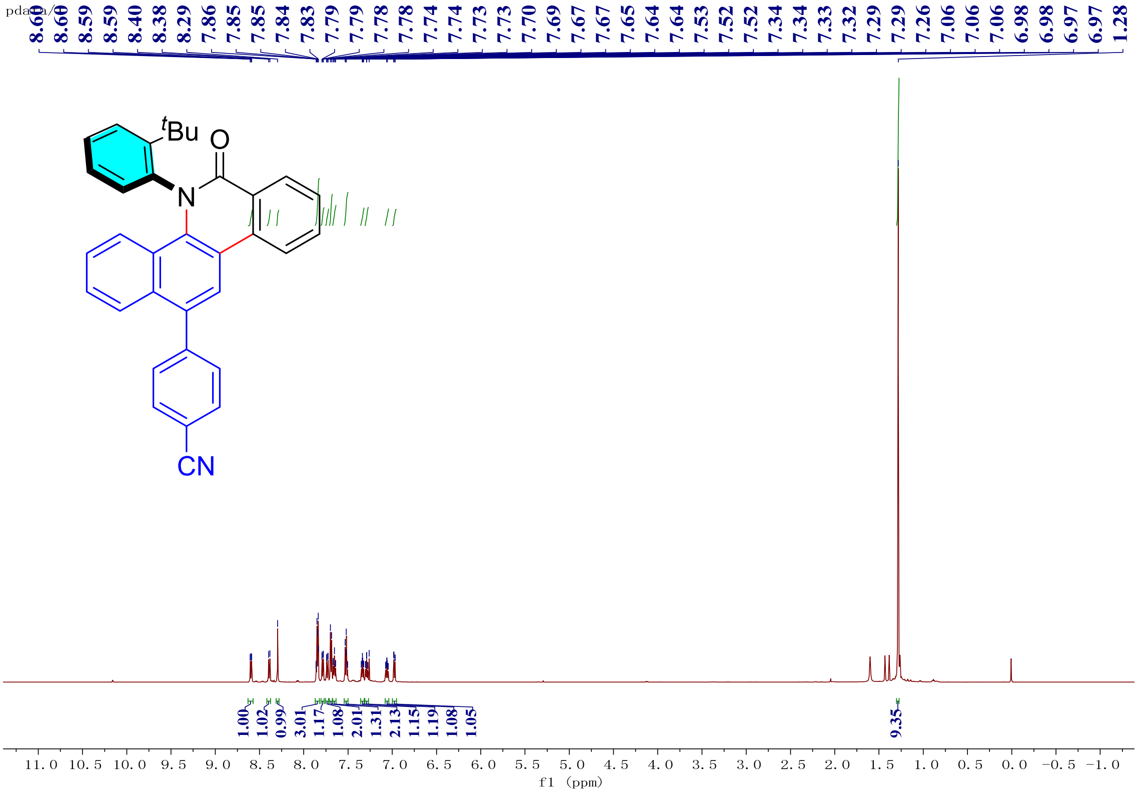

^{13}C NMR (151 MHz, Chloroform-*d*)

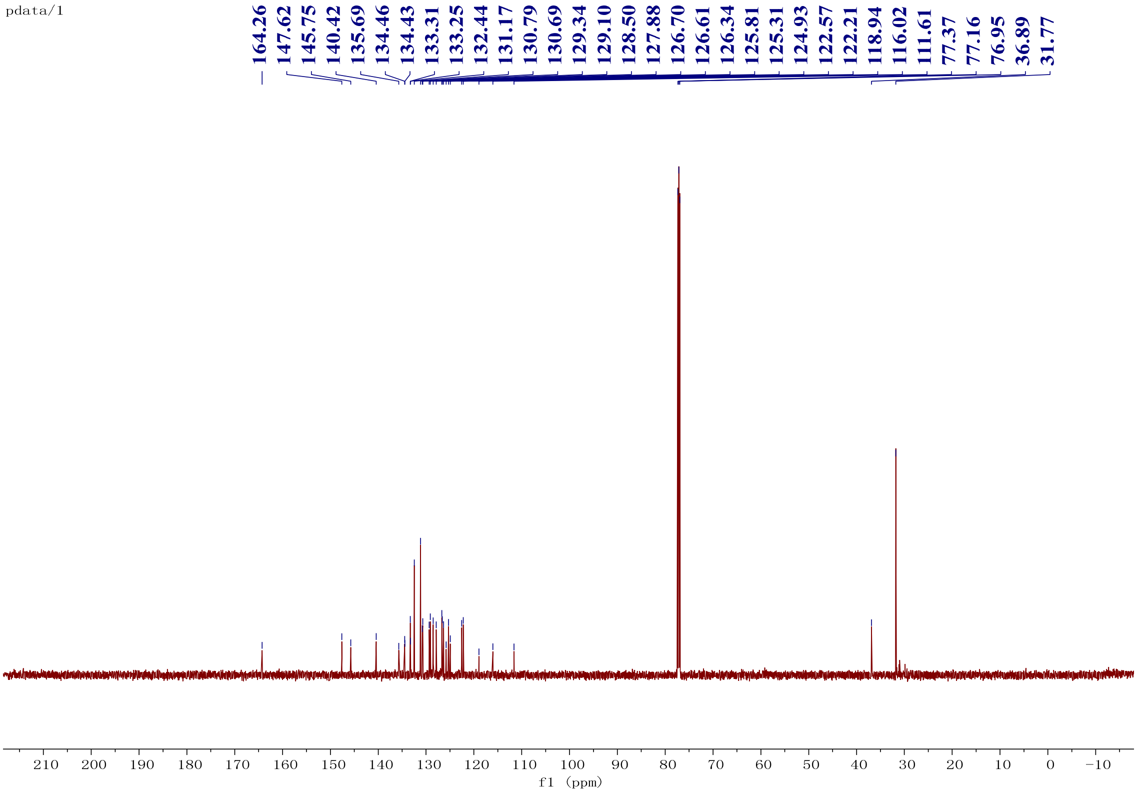

23

^{1}H NMR (600 MHz, Chloroform-*d*)

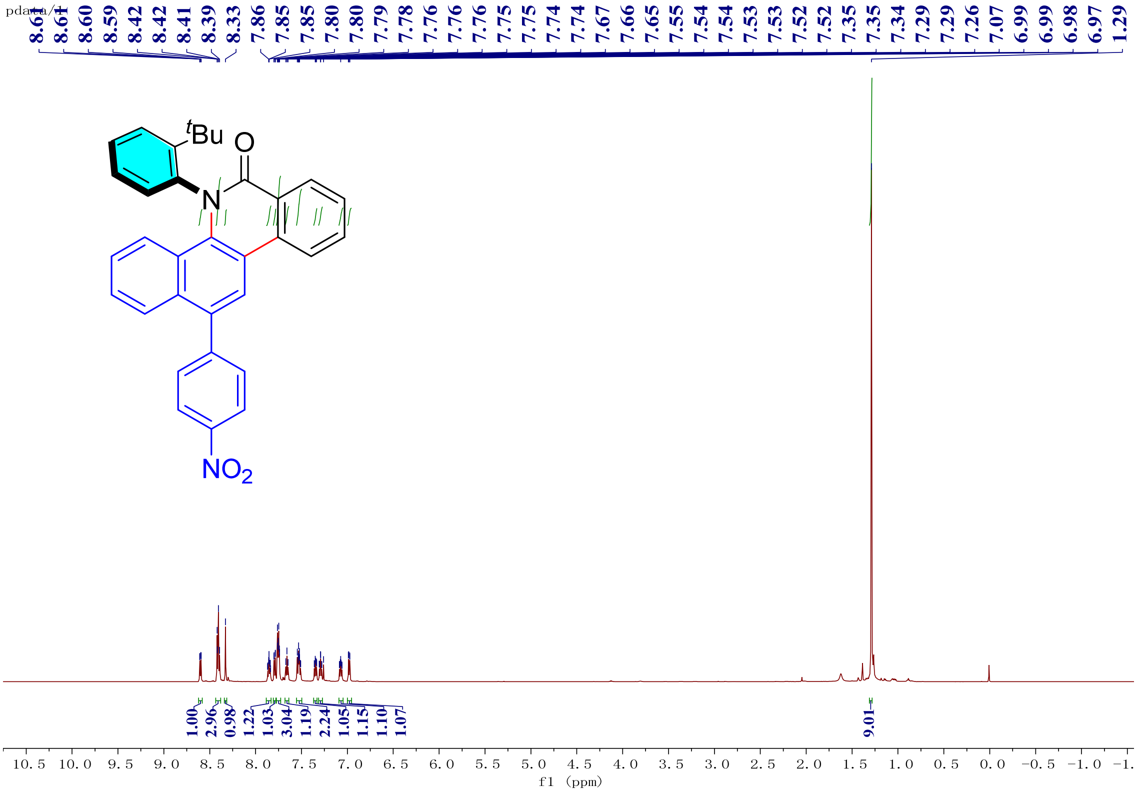

^{13}C NMR (151 MHz, Chloroform-*d*)

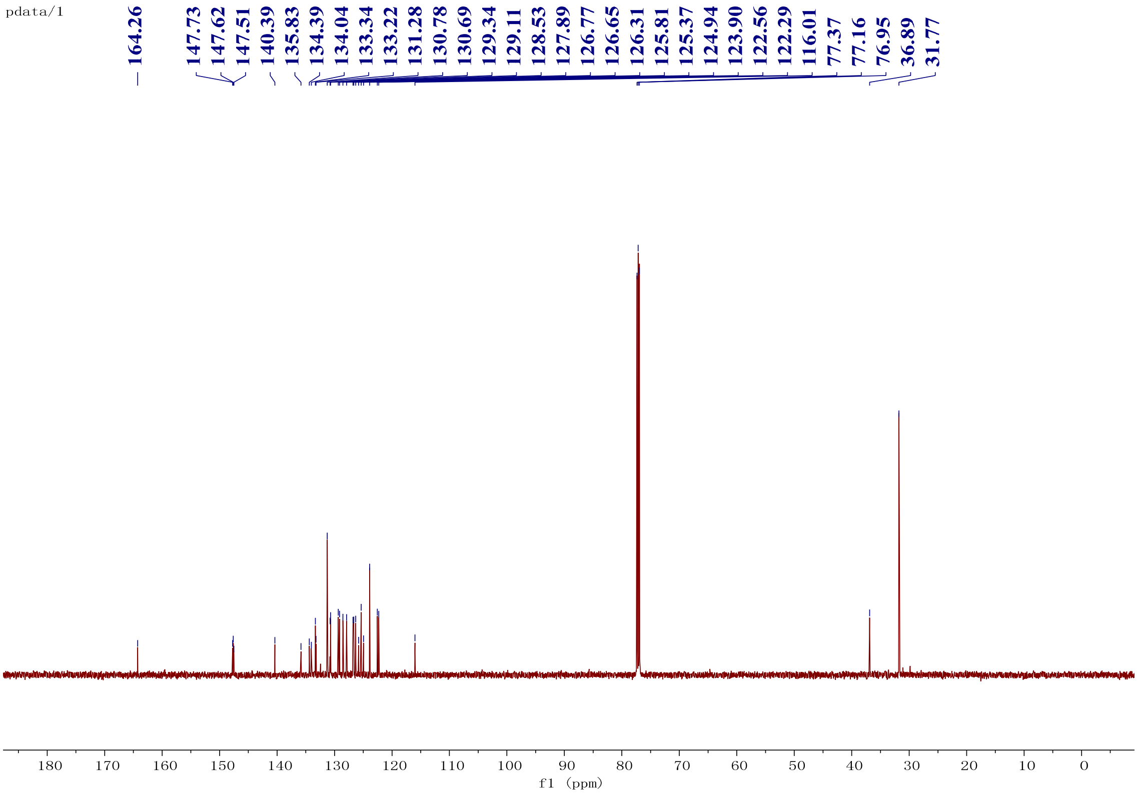

24

^{1}H NMR (600 MHz, Chloroform-*d*)

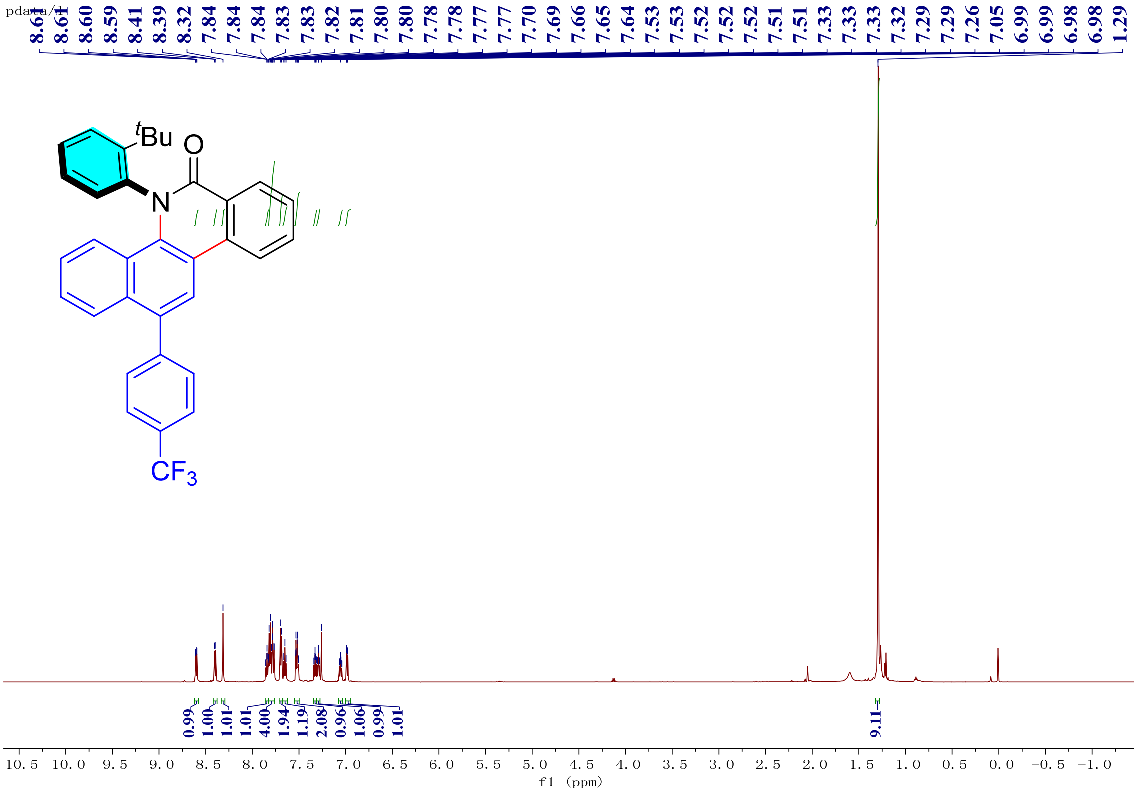

^{13}C NMR (151 MHz, Chloroform-*d*)

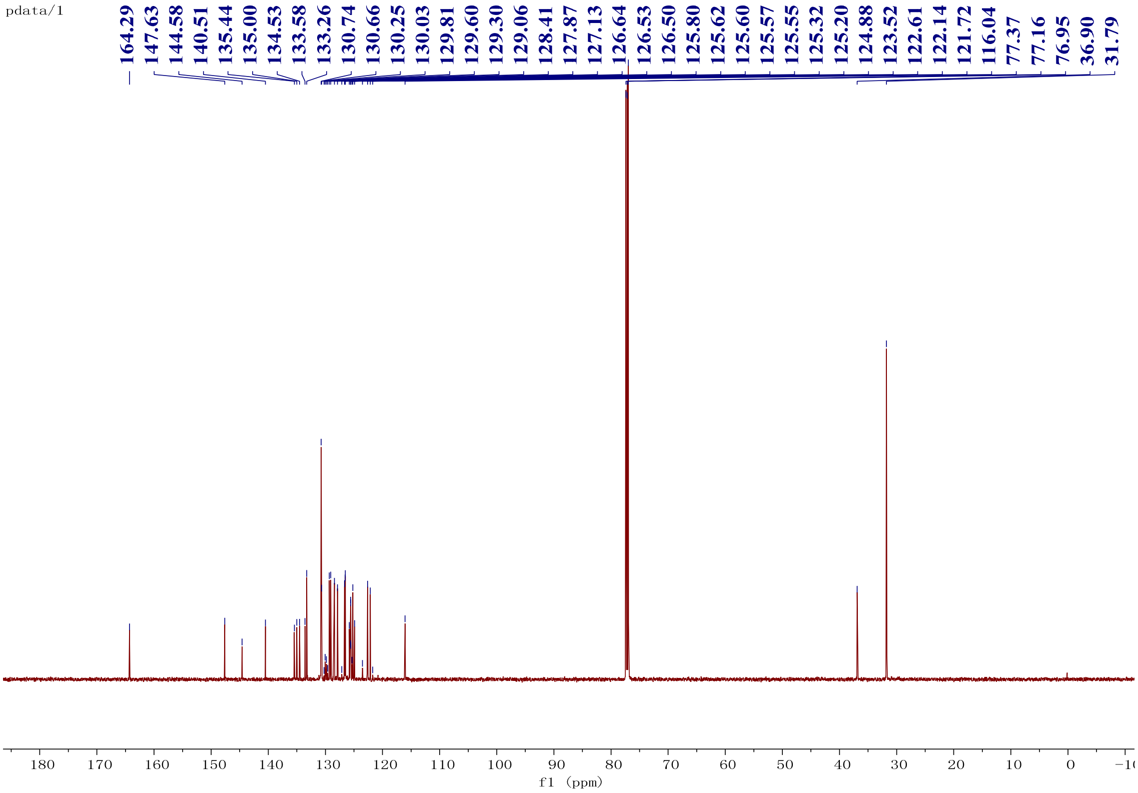

^{19}F NMR (565 MHz, Chloroform-*d*)

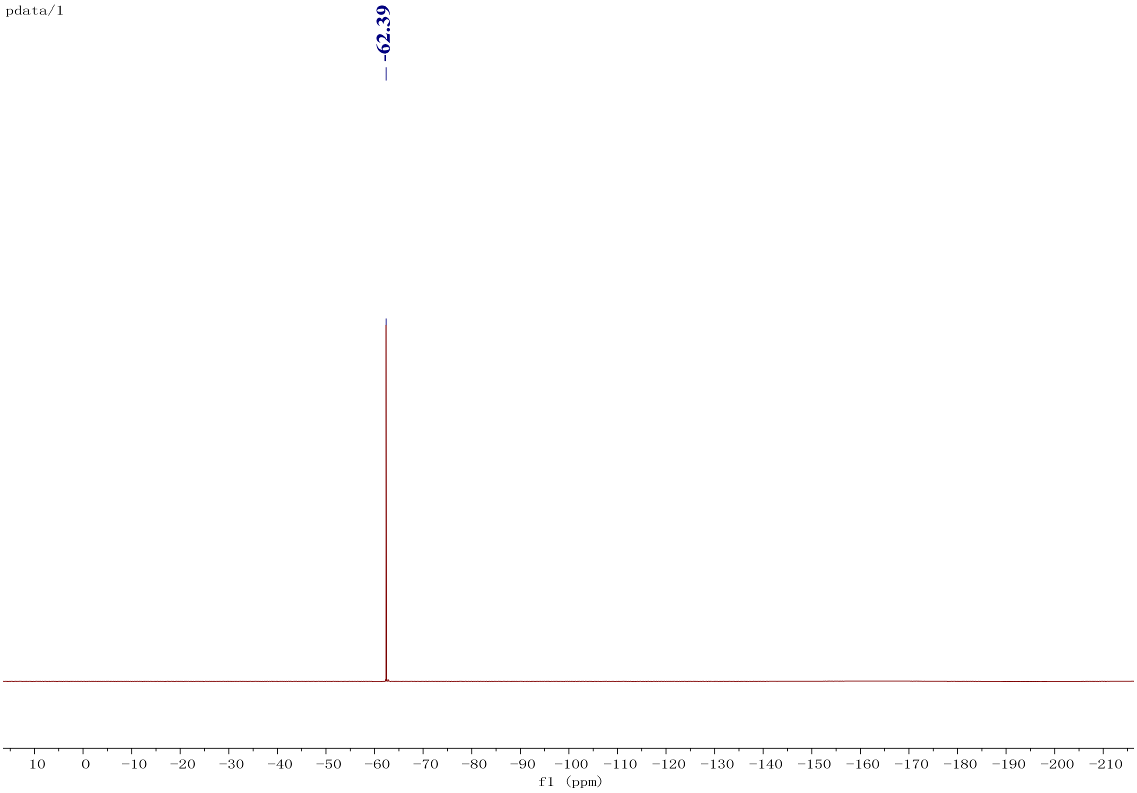

25

^{1}H NMR (600 MHz, Chloroform-*d*)

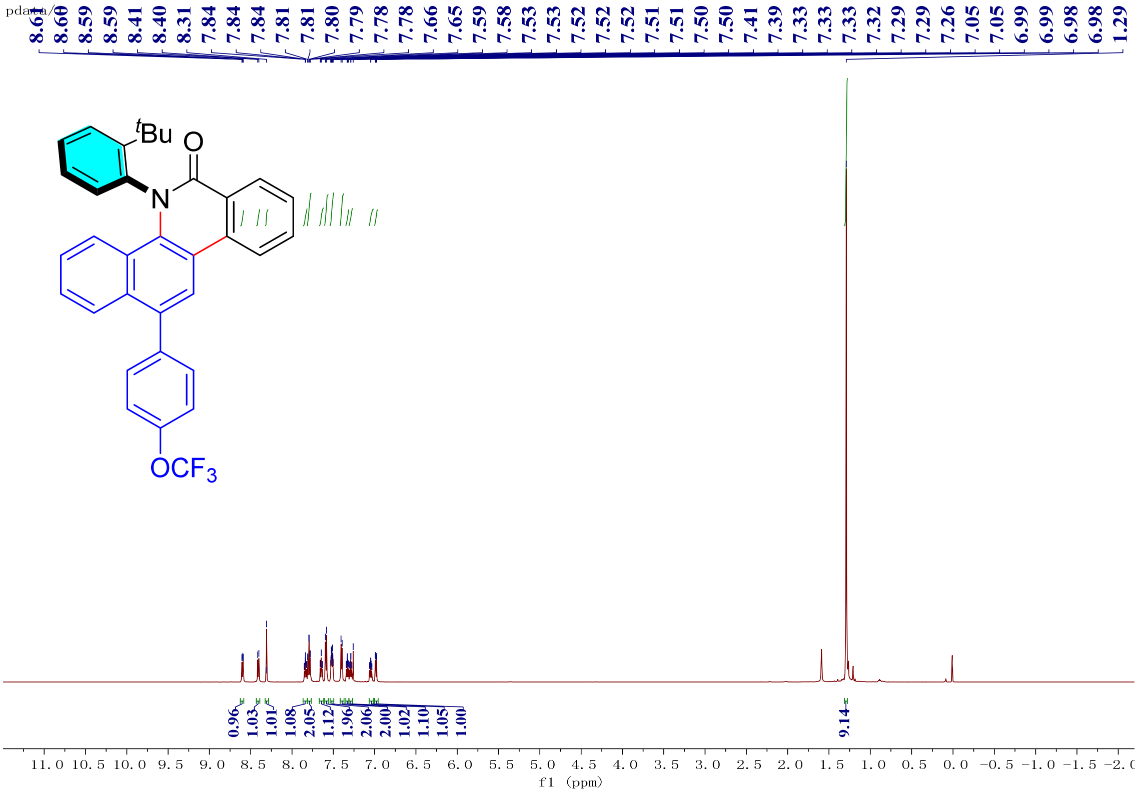

^{13}C NMR (151 MHz, Chloroform-*d*)

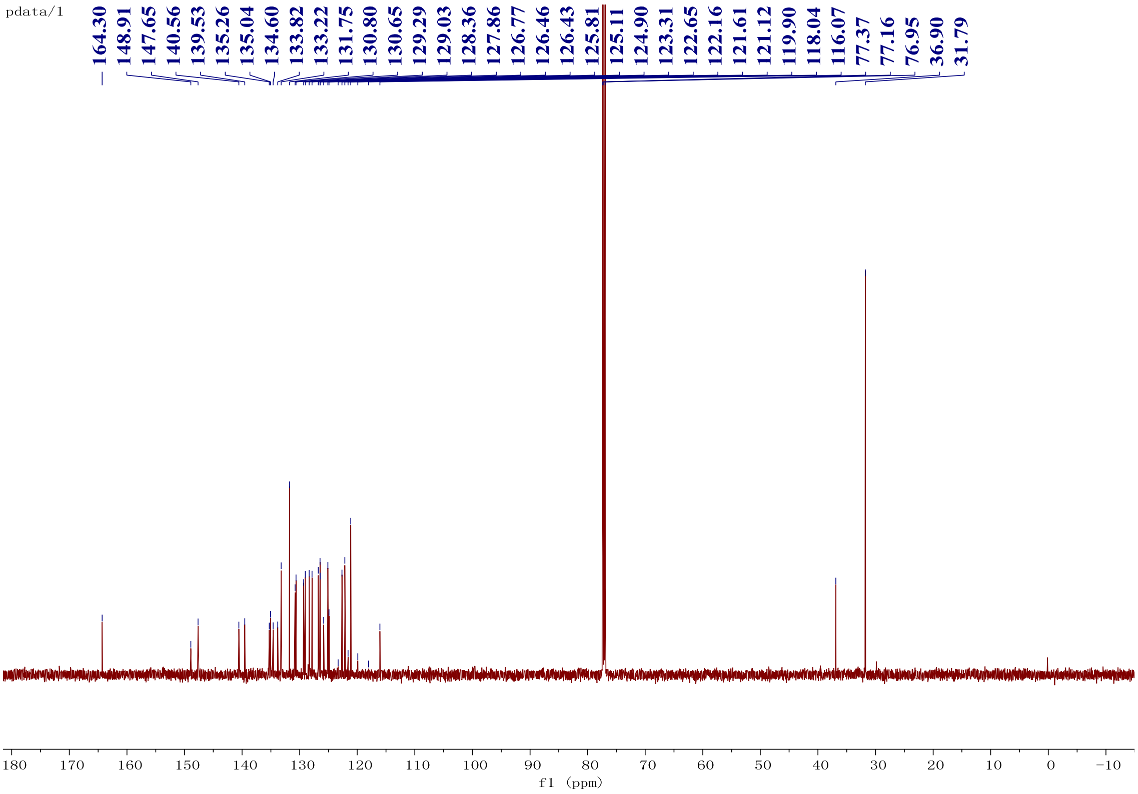

^{19}F NMR (565 MHz, Chloroform-*d*)

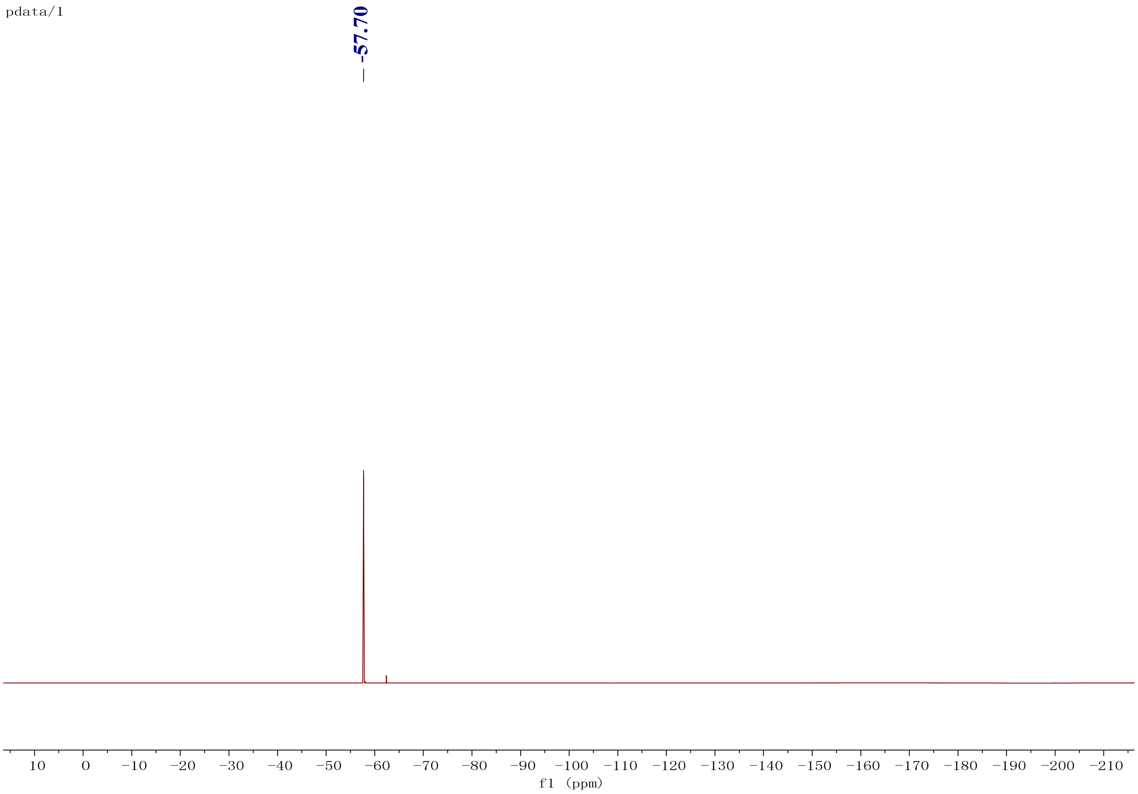

26

^{1}H NMR (600 MHz, Chloroform-*d*)

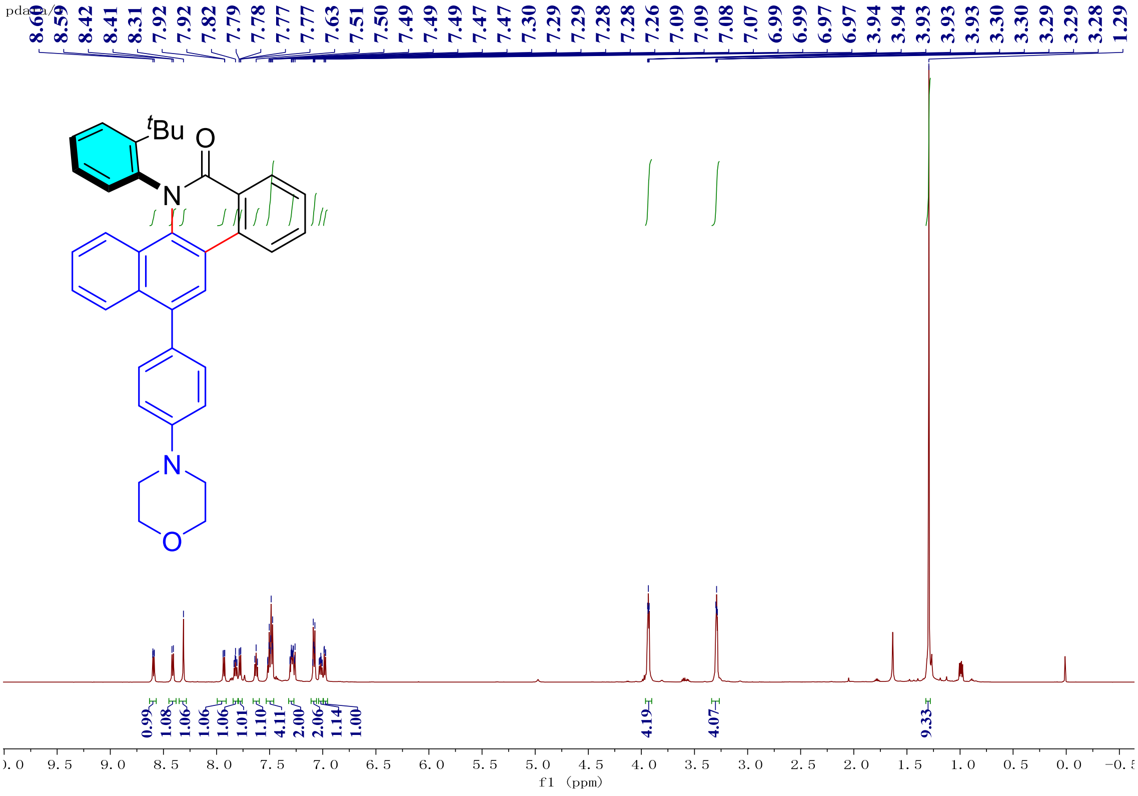

^{13}C NMR (151 MHz, Chloroform-*d*)

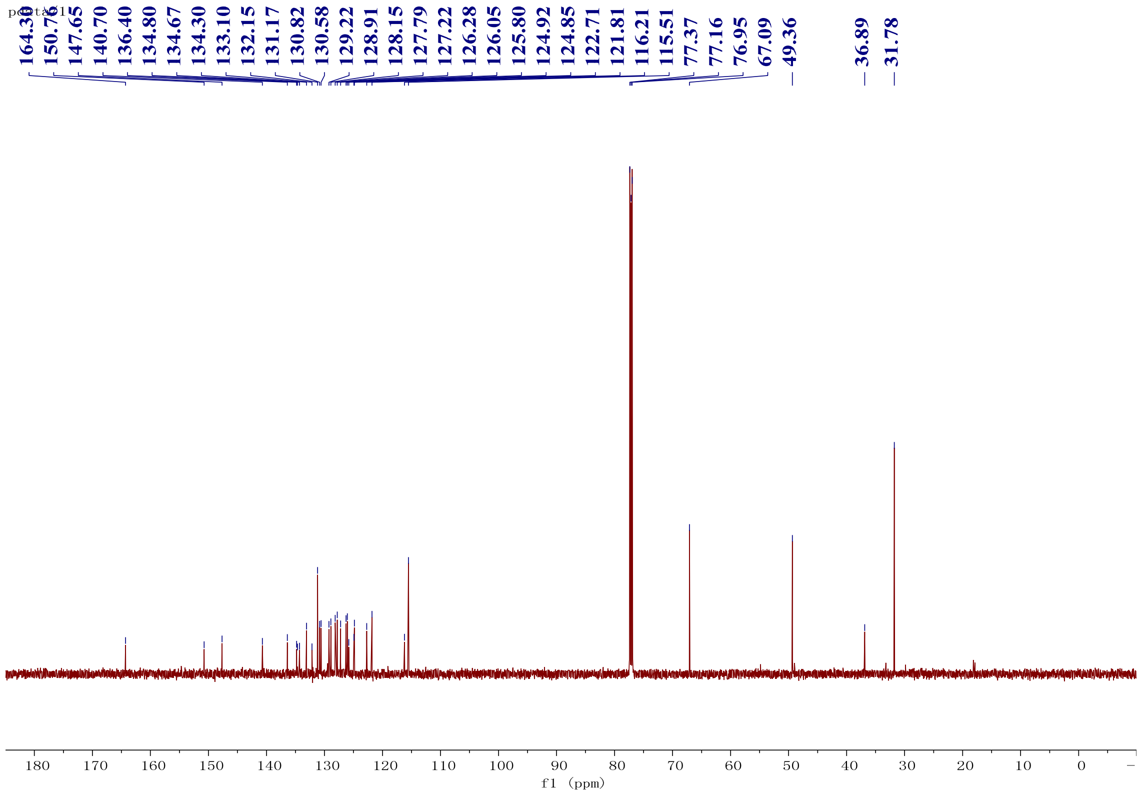

27

^{1}H NMR (400 MHz, Chloroform-*d*)

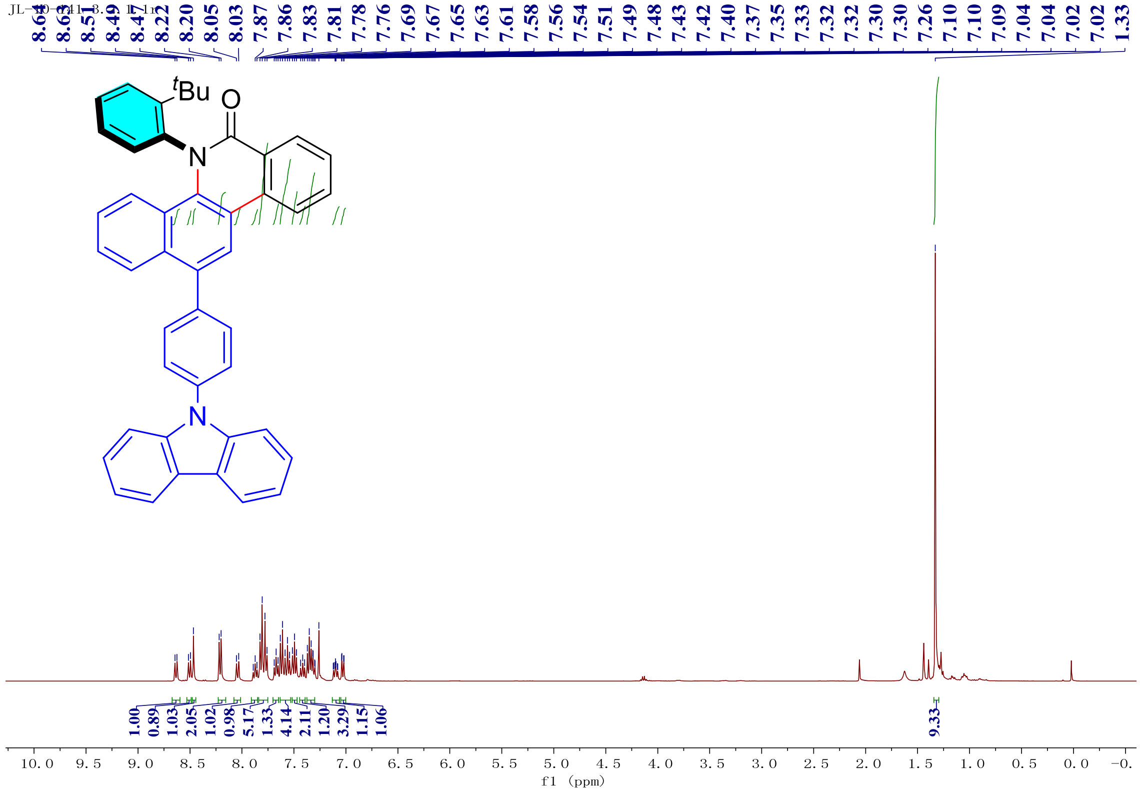

^{13}C NMR (101 MHz, Chloroform-*d*)

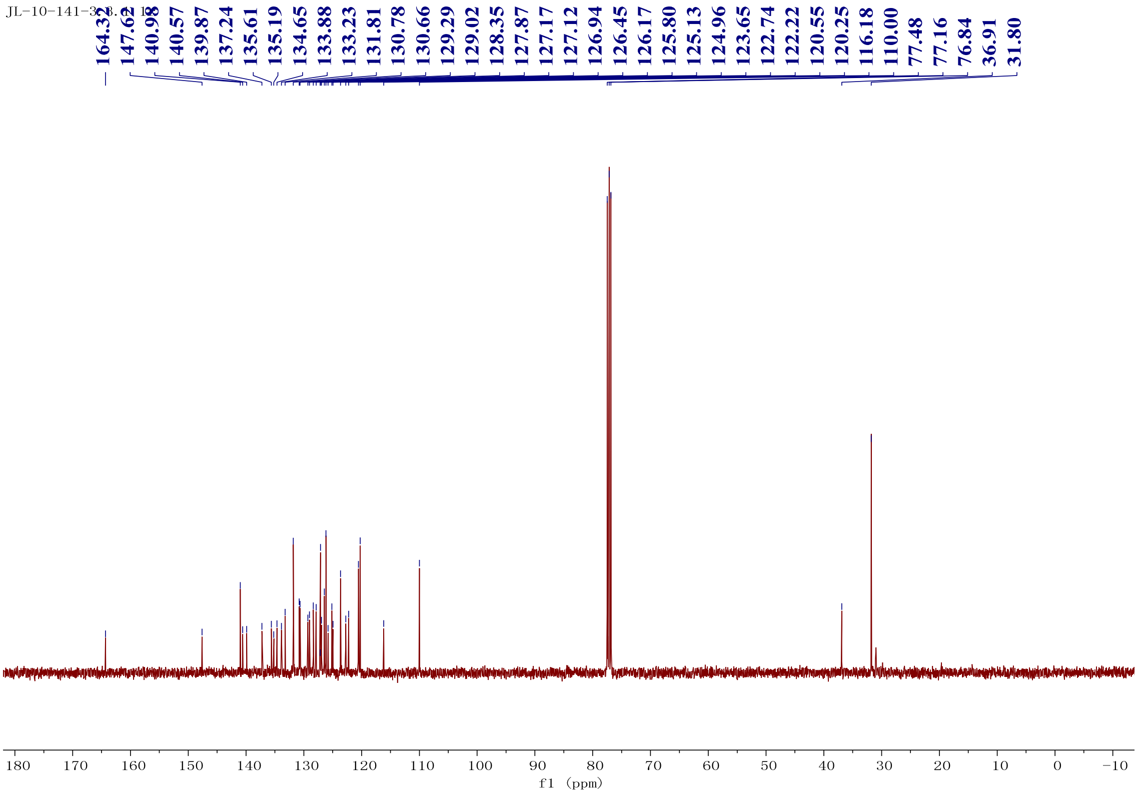

28

^{1}H NMR (400 MHz, Chloroform-*d*)

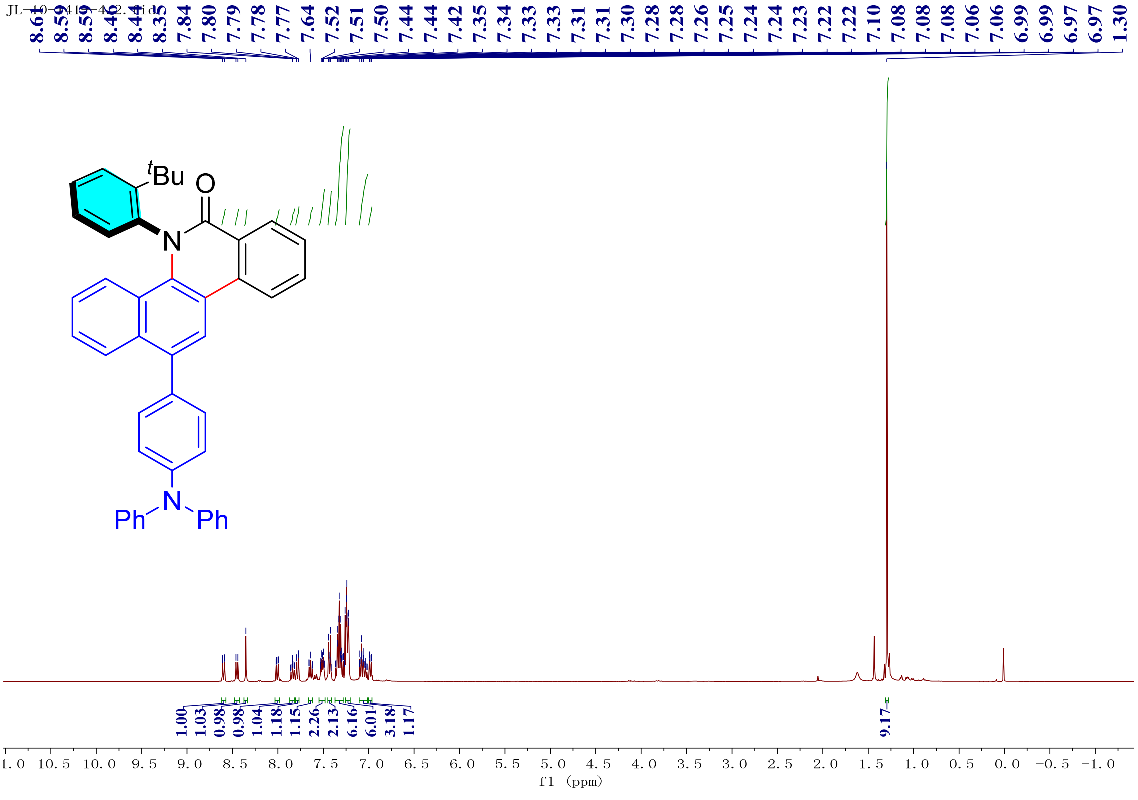

^{13}C NMR (101 MHz, Chloroform-*d*)

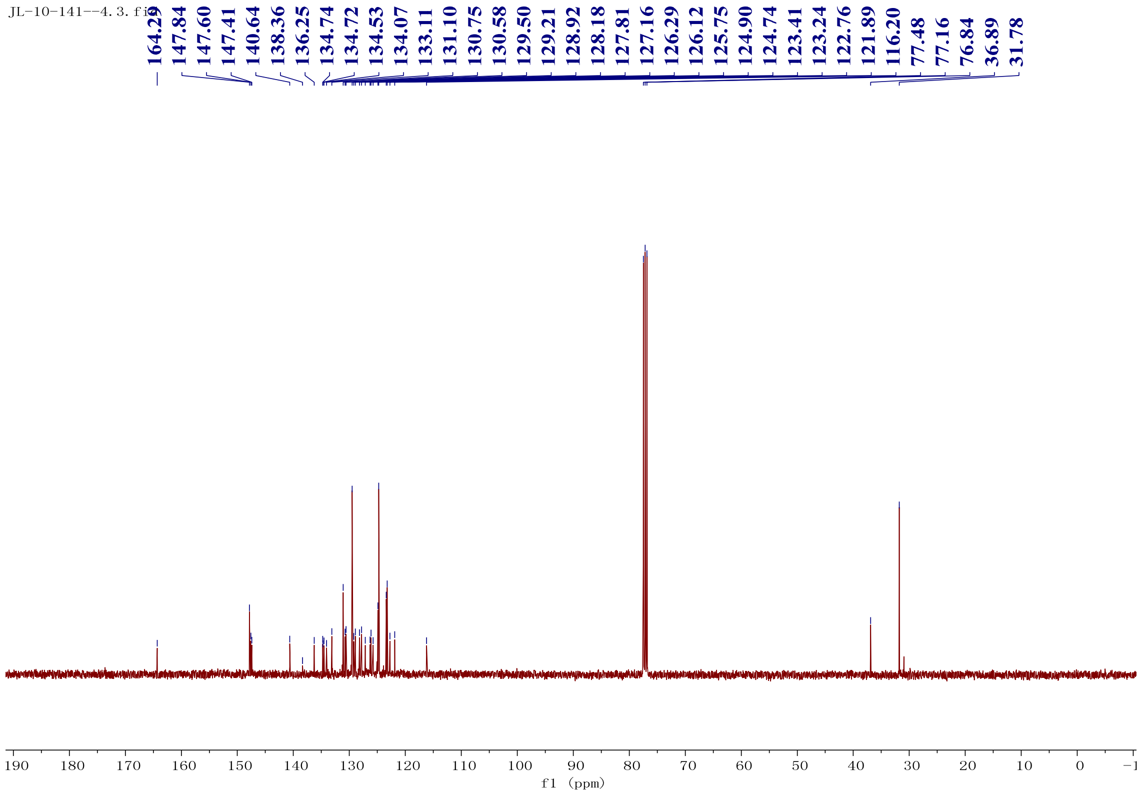

29

^{1}H NMR (600 MHz, Chloroform-*d*)

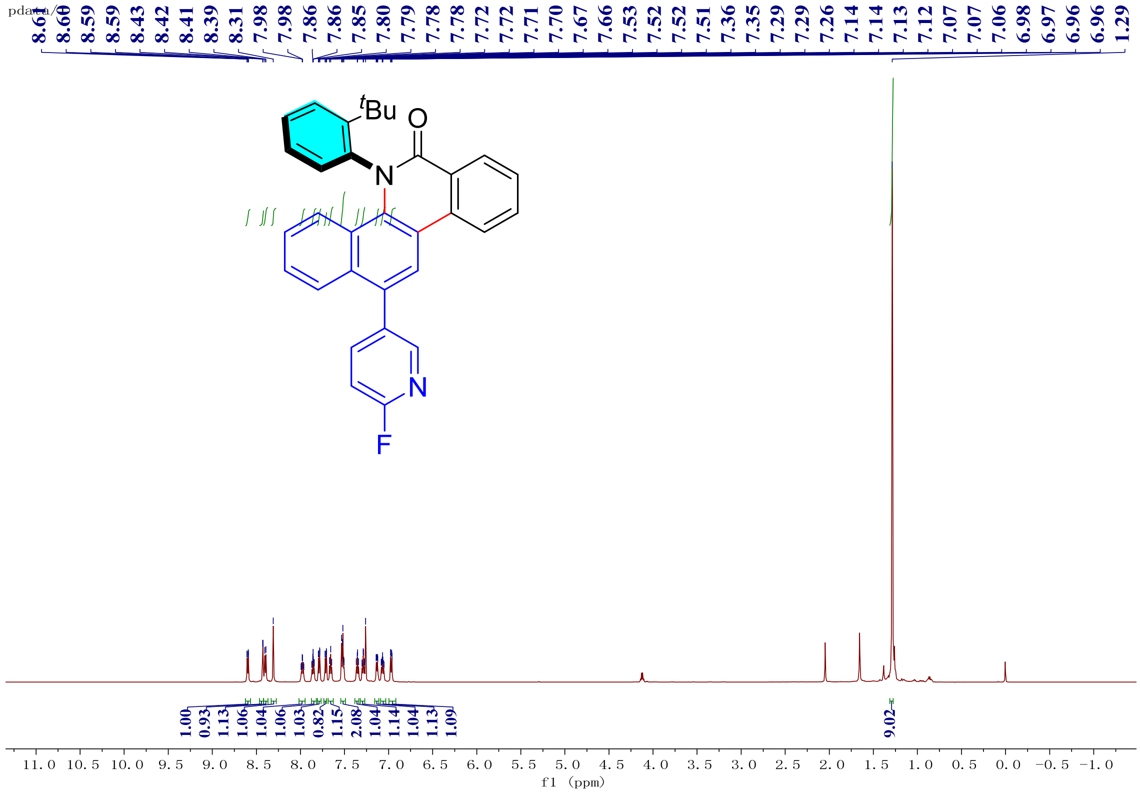

^{13}C NMR (151 MHz, Chloroform-*d*)

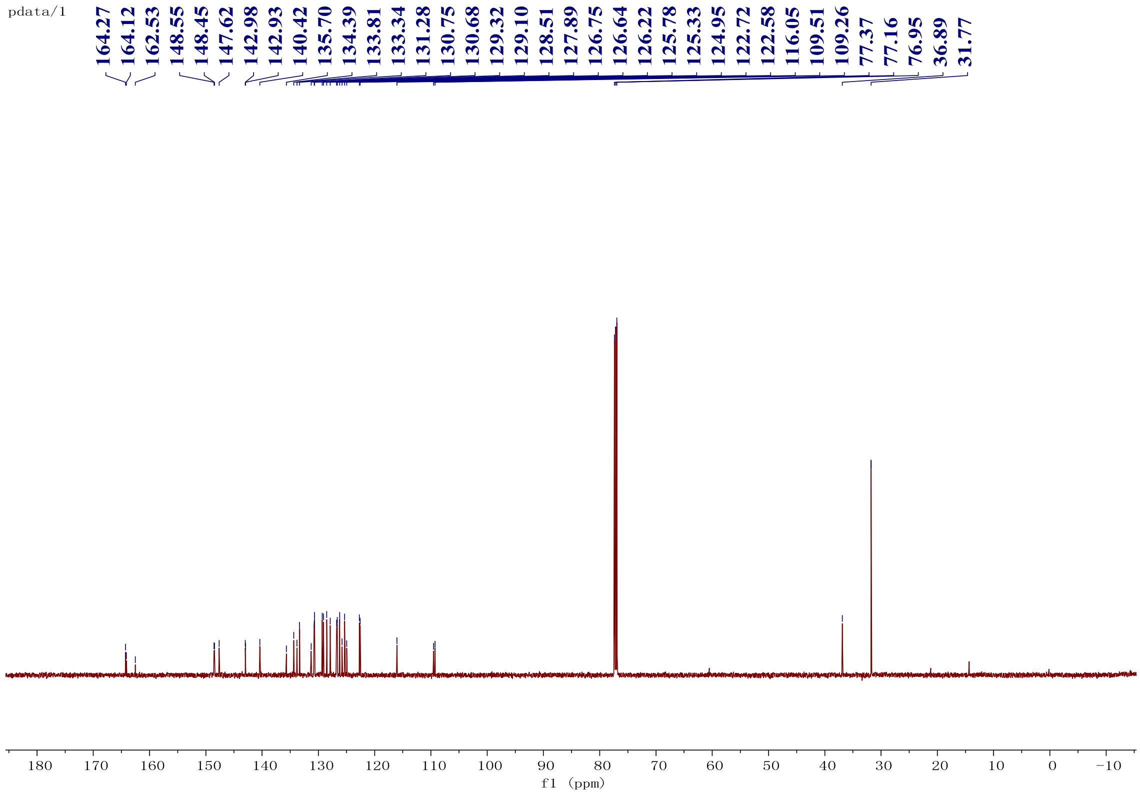

^{19}F NMR (565 MHz, Chloroform-*d*)

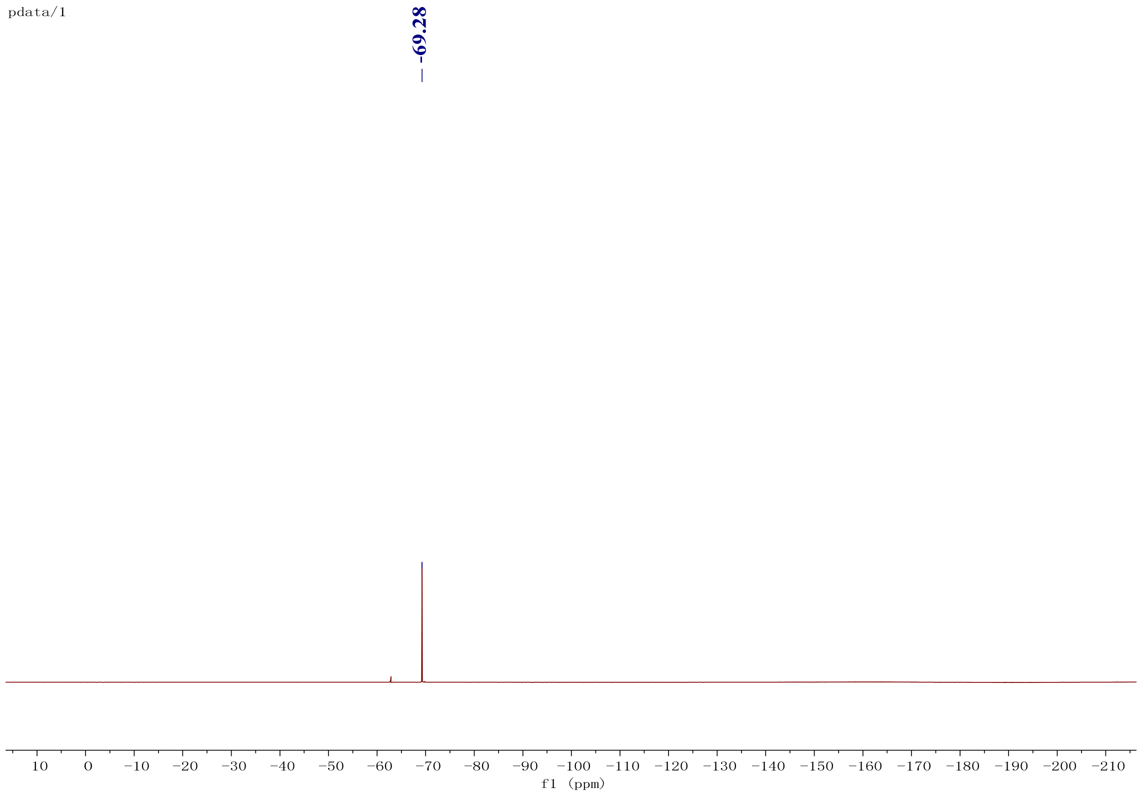

30

^{1}H NMR (600 MHz, Chloroform-*d*)

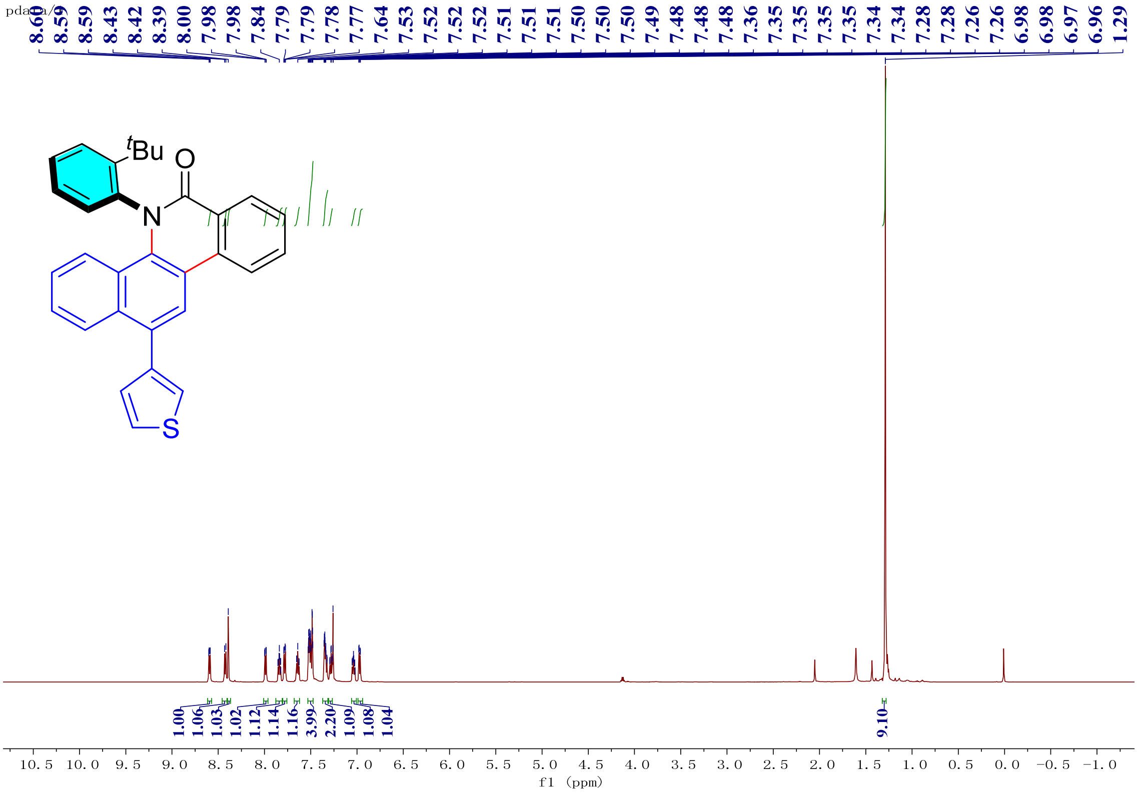

^{13}C NMR (151 MHz, Chloroform-*d*)

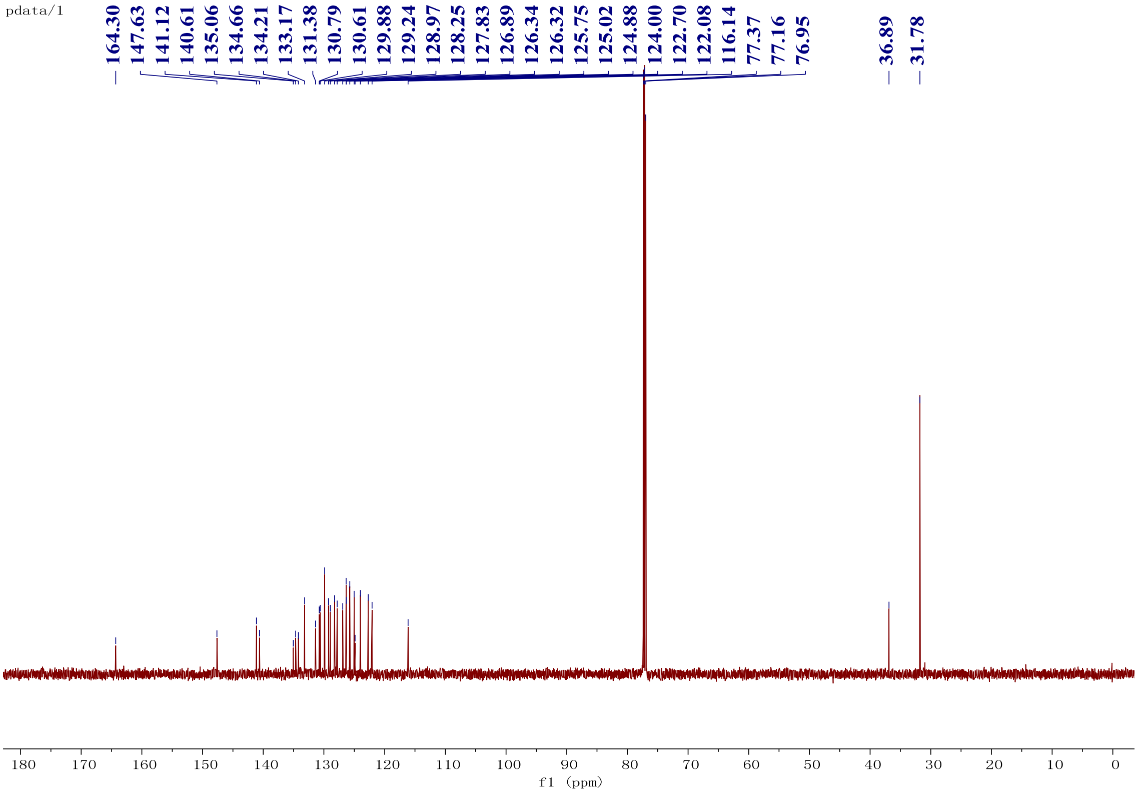

31

^{1}H NMR (600 MHz, Chloroform-*d*)

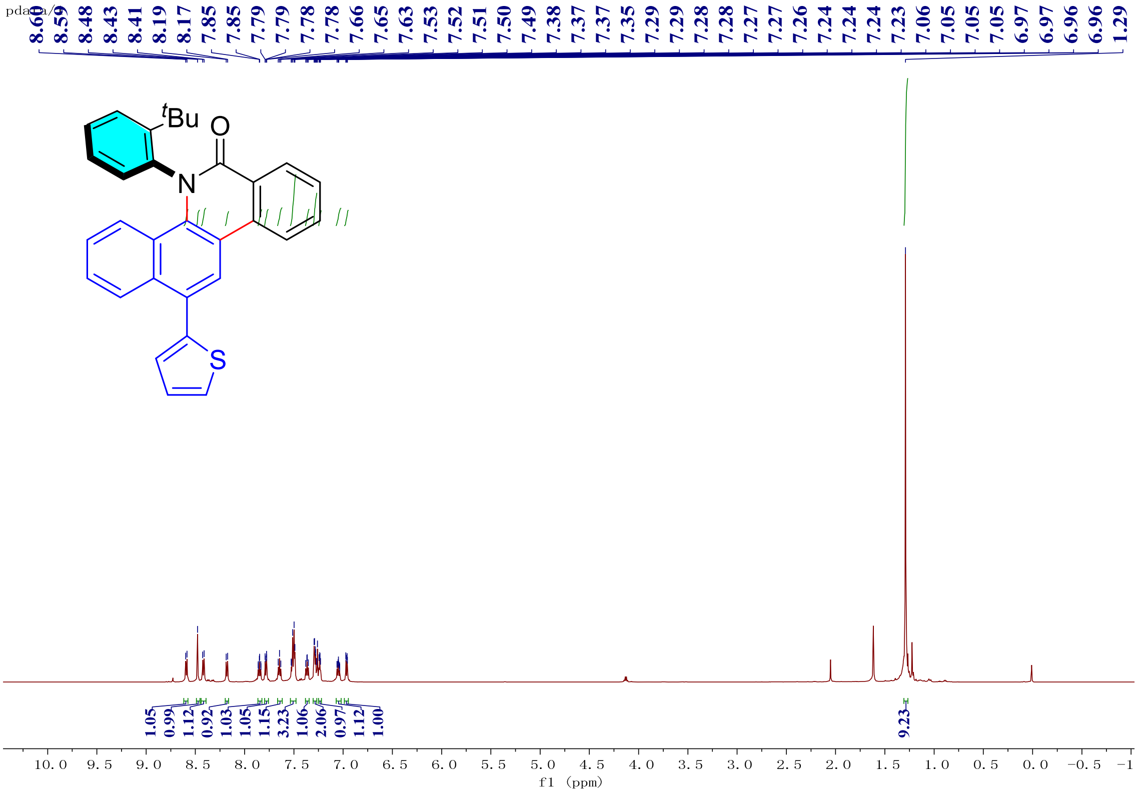

^{13}C NMR (151 MHz, Chloroform-*d*)

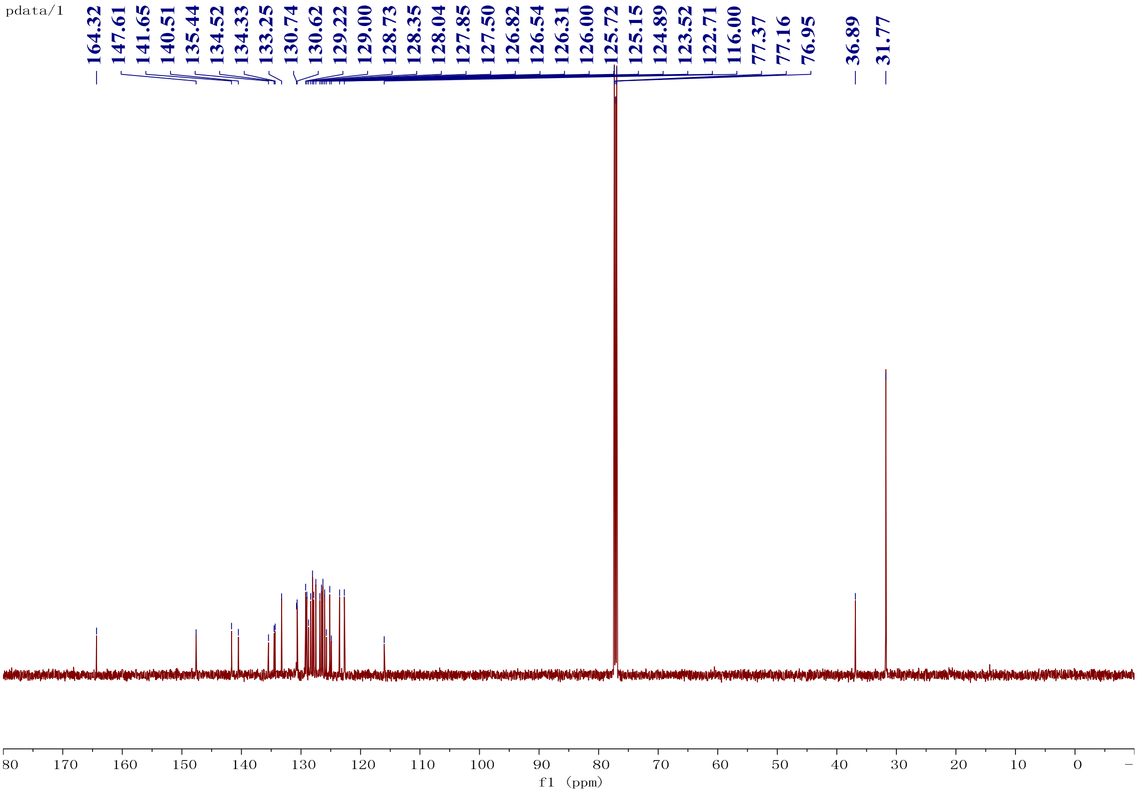

32

^{1}H NMR (400 MHz, Chloroform-*d*)

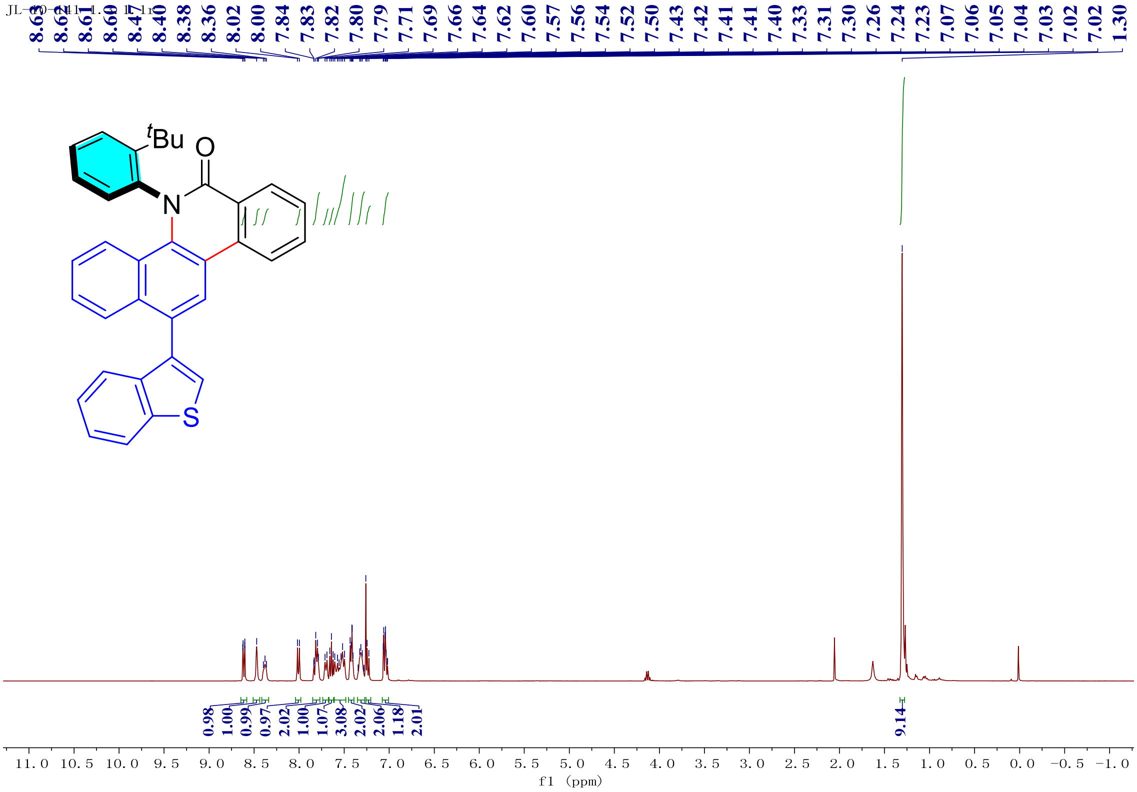

^{13}C NMR (101 MHz, Chloroform-*d*)

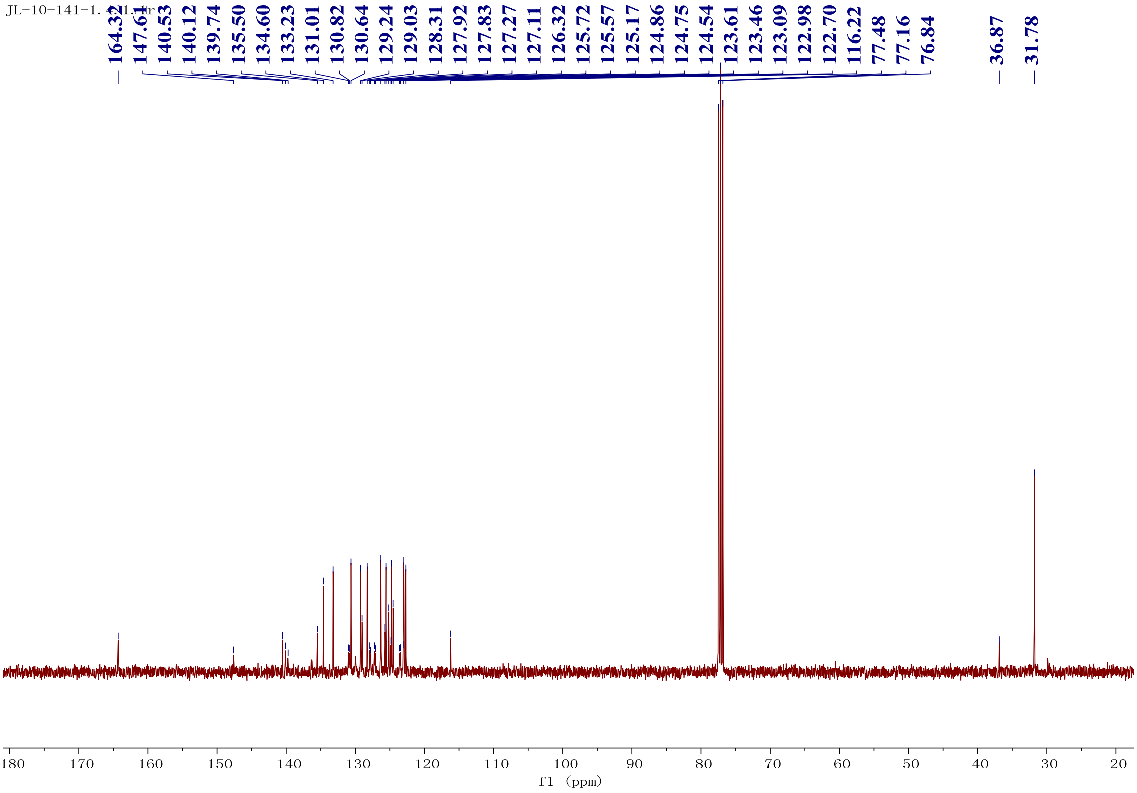

33

^{1}H NMR (600 MHz, Chloroform-*d*)

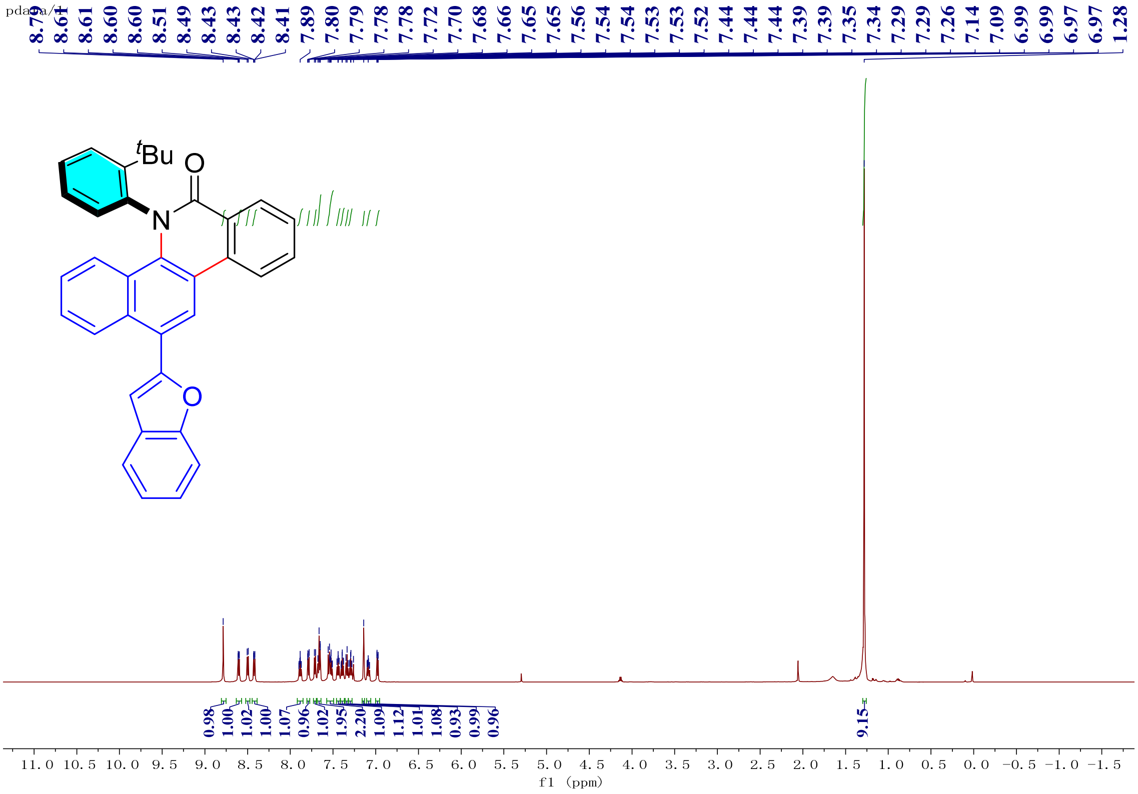

^{13}C NMR (151 MHz, Chloroform-*d*)

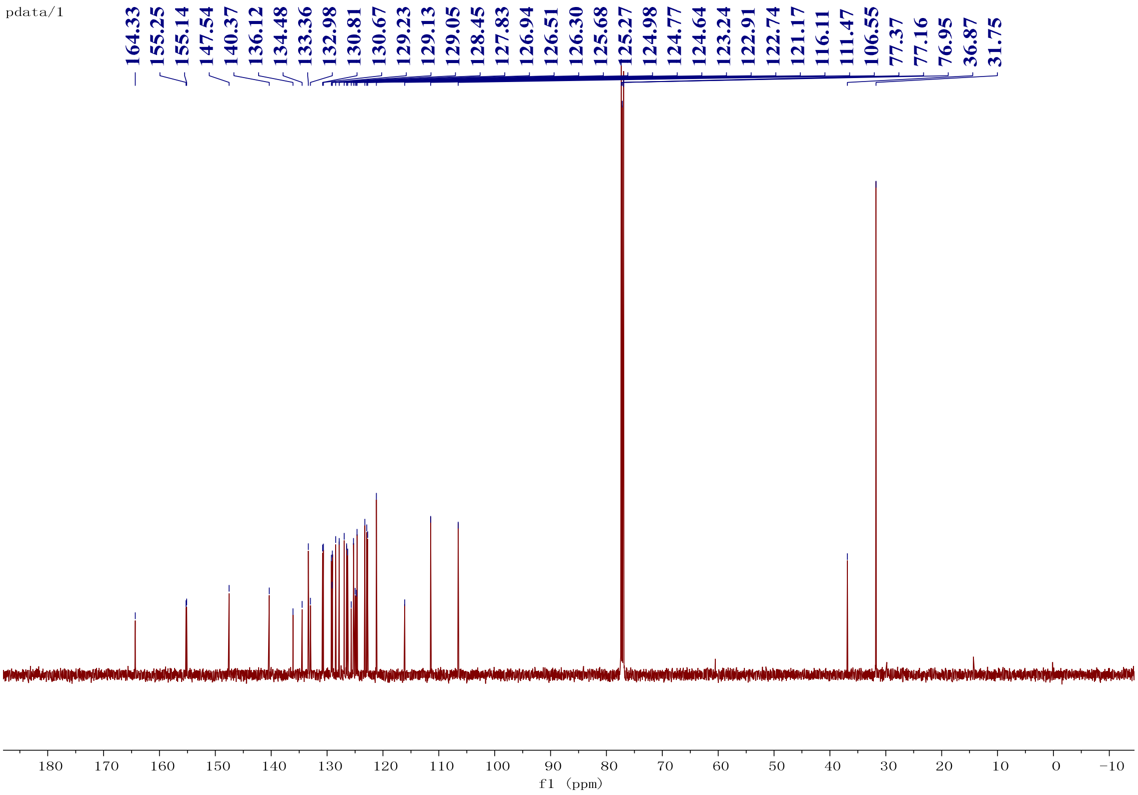

34

^{1}H NMR (600 MHz, Chloroform-*d*)

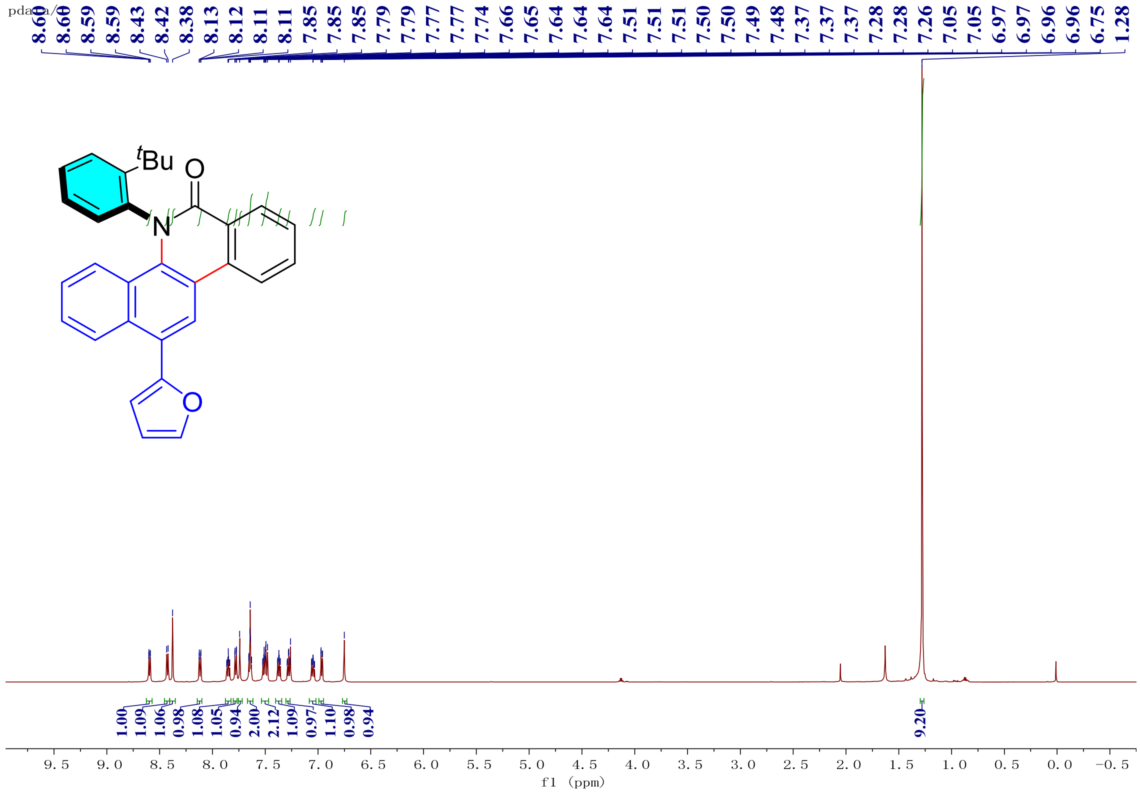

^{13}C NMR (151 MHz, Chloroform-*d*)

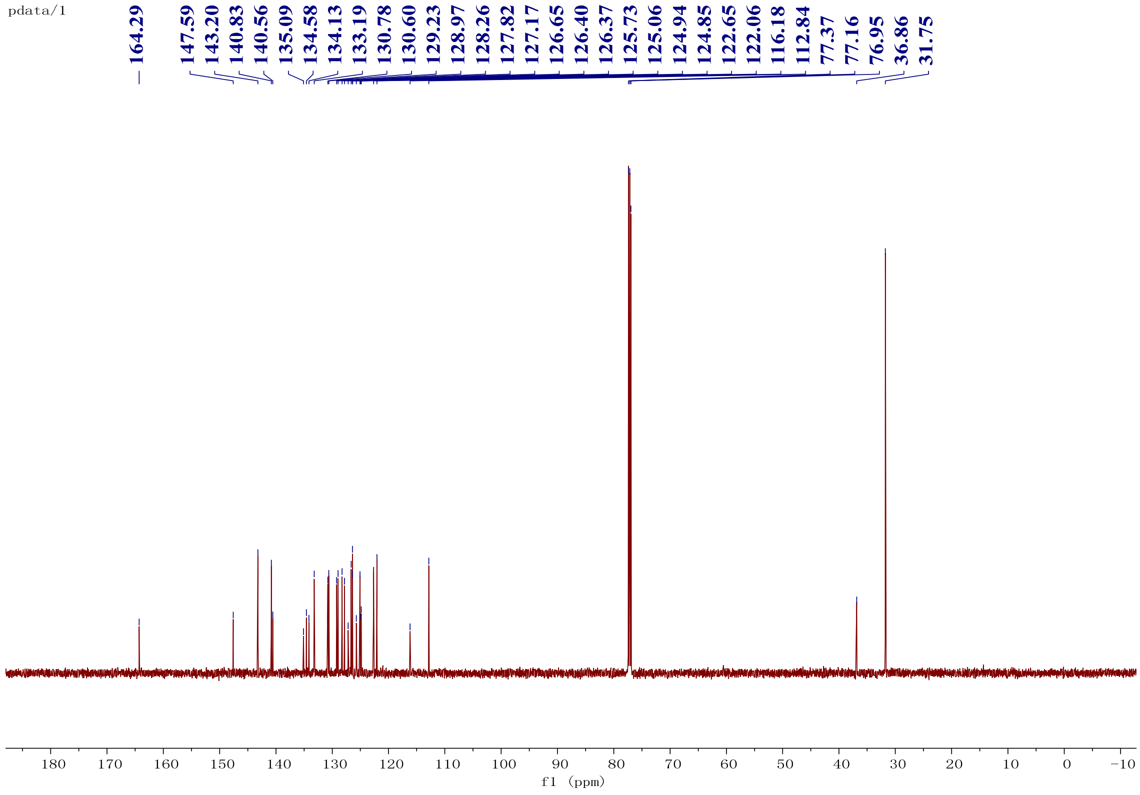

35

^{1}H NMR (400 MHz, Chloroform-*d*)

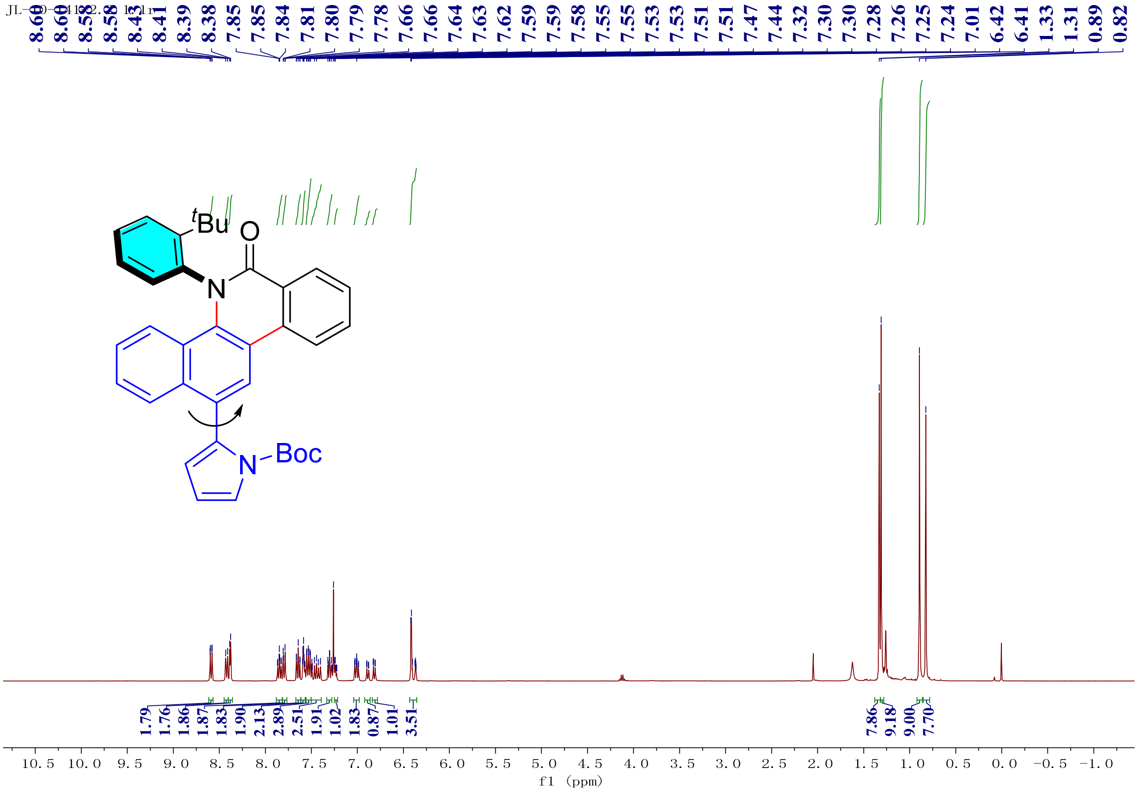

^{13}C NMR (101 MHz, Chloroform-*d*)

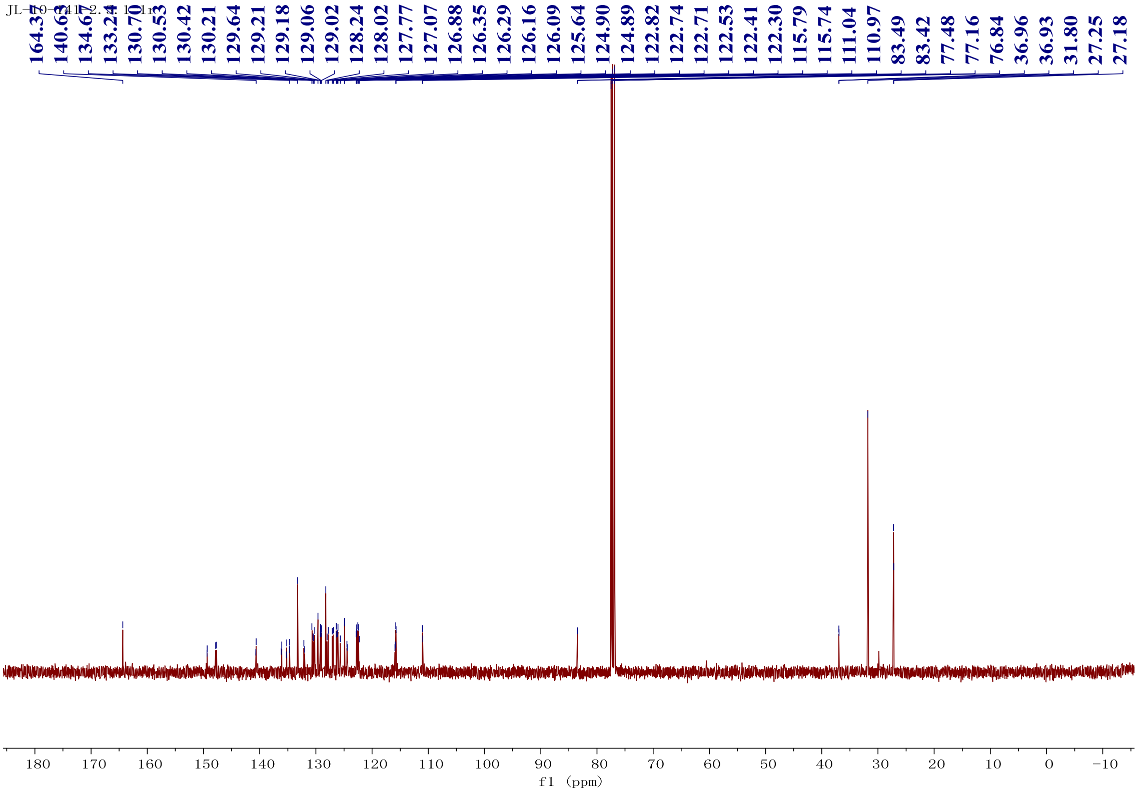

35′

^{1}H NMR (400 MHz, Chloroform-*d*)

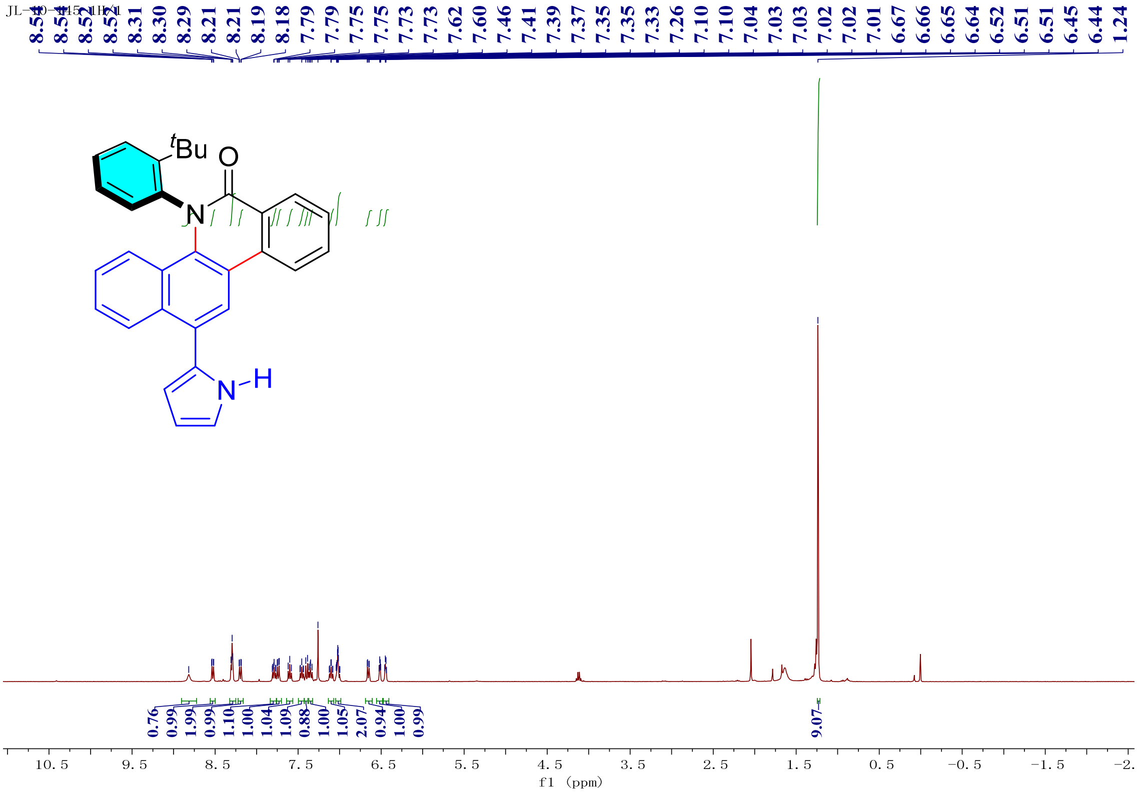

^{13}C NMR (101 MHz, Chloroform-*d*)

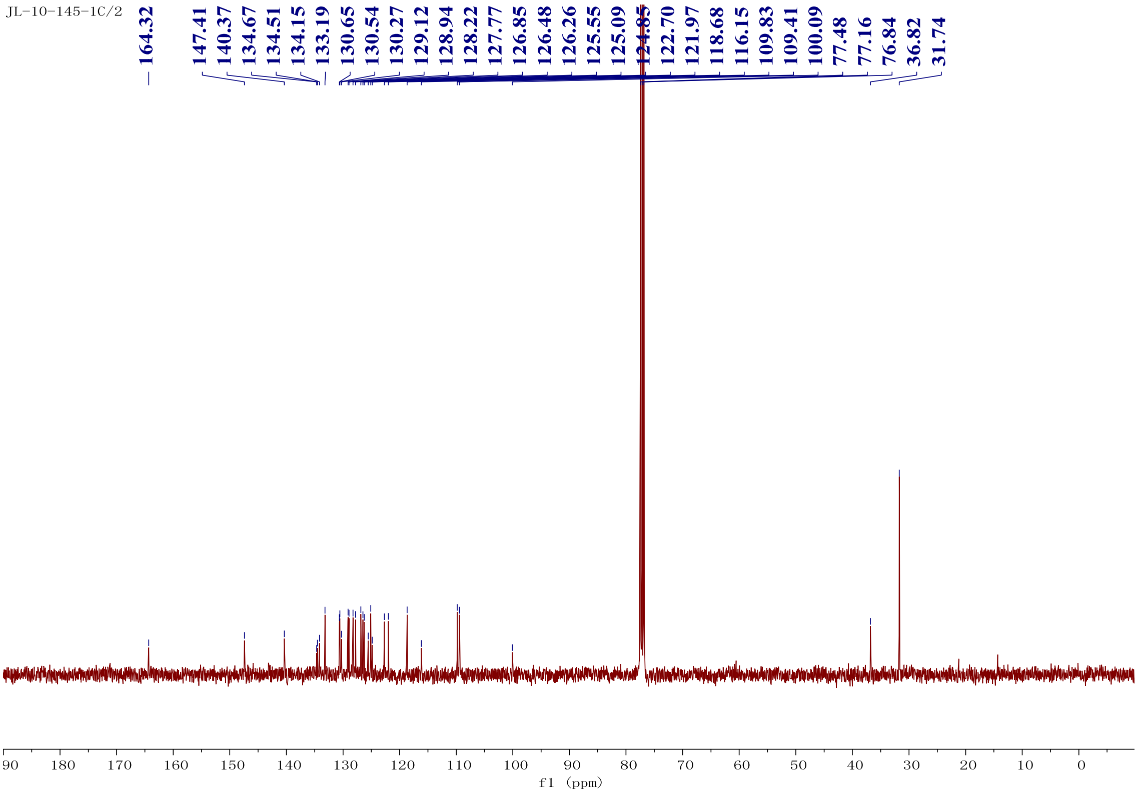

36

^{1}H NMR (400 MHz, Chloroform-*d*)

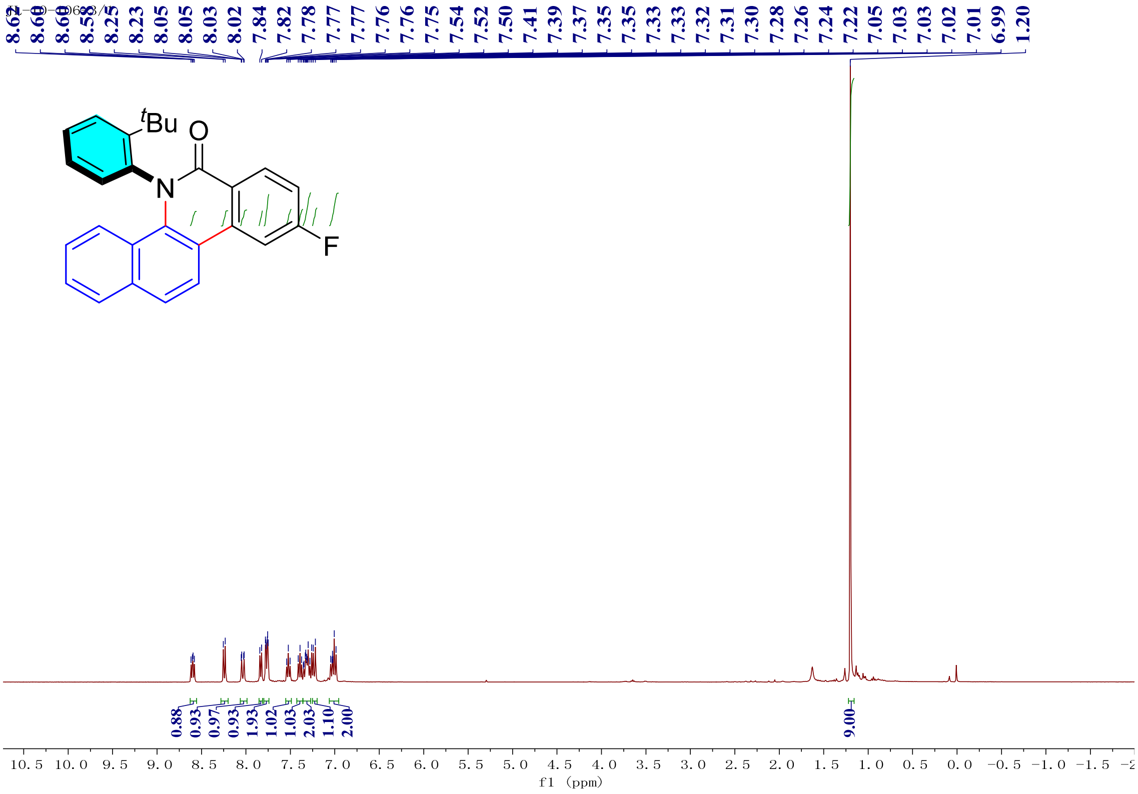

^{13}C NMR (101 MHz, Chloroform-*d*)

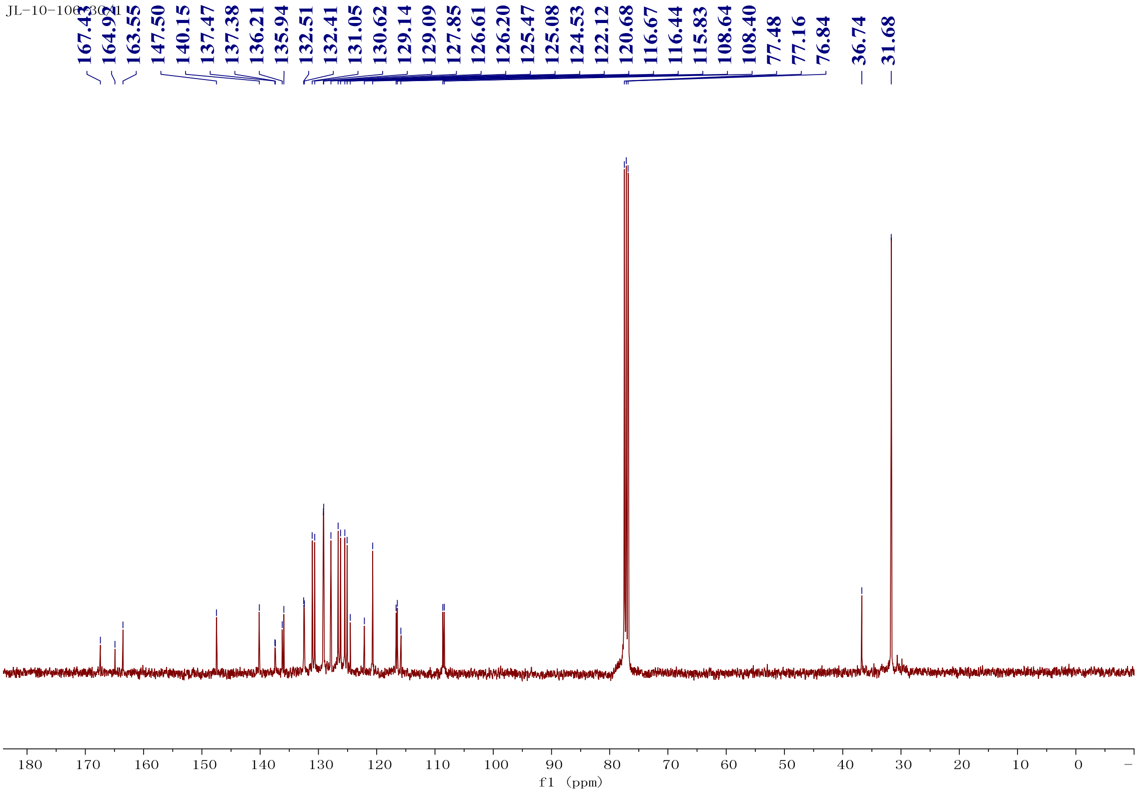

^{19}F NMR (376 MHz, Chloroform-*d*)

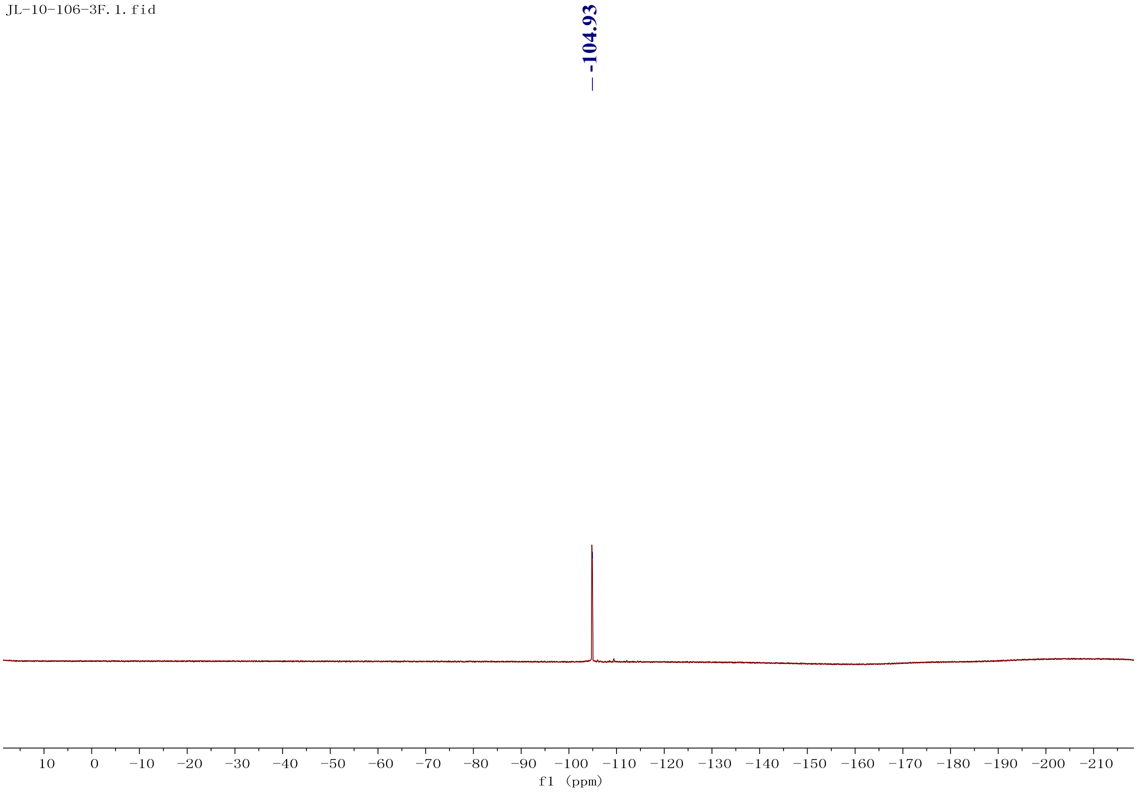

37

^{1}H NMR (400 MHz, Chloroform-*d*)

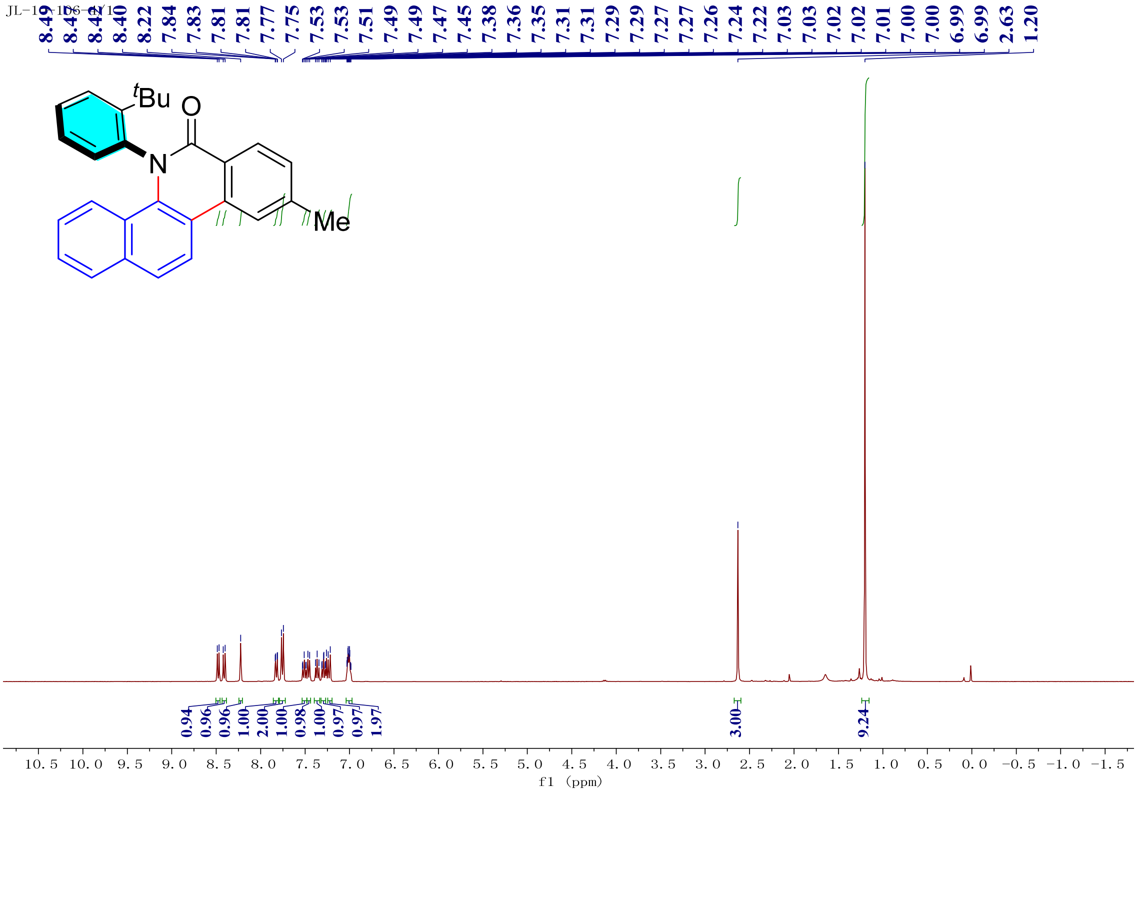

^{13}C NMR (101 MHz, Chloroform-*d*)

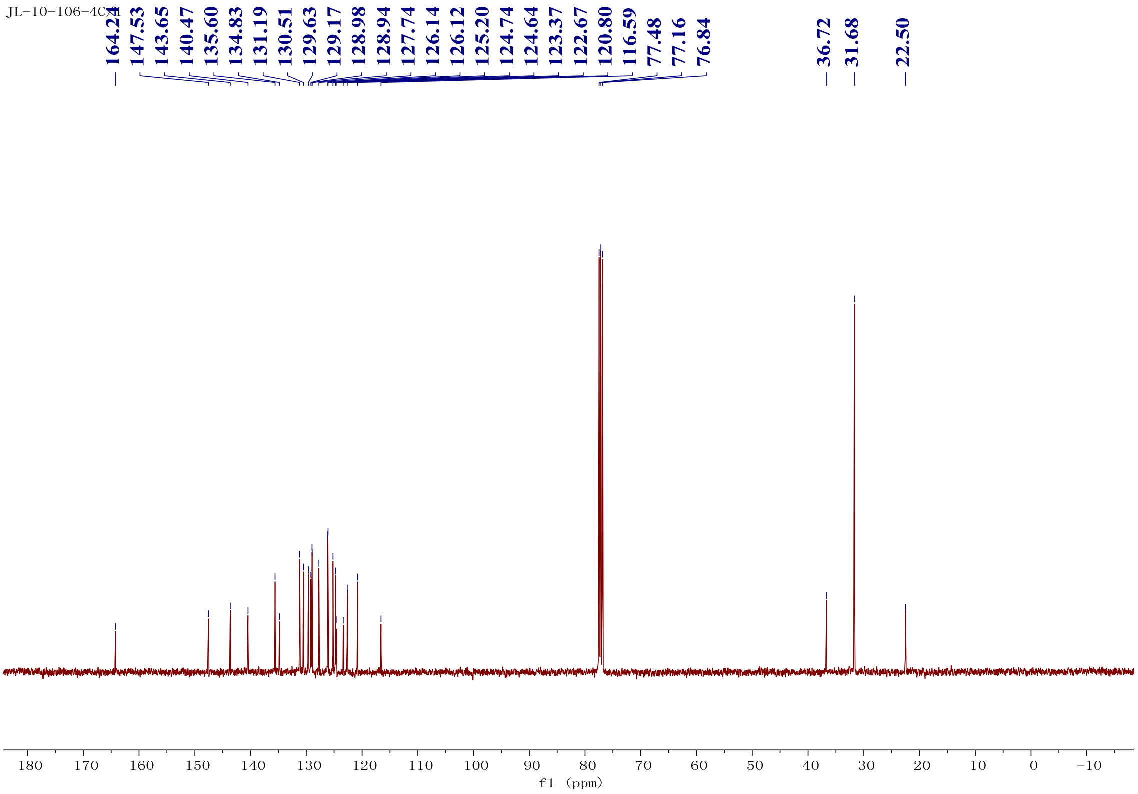

38

^{1}H NMR (400 MHz, Chloroform-*d*)

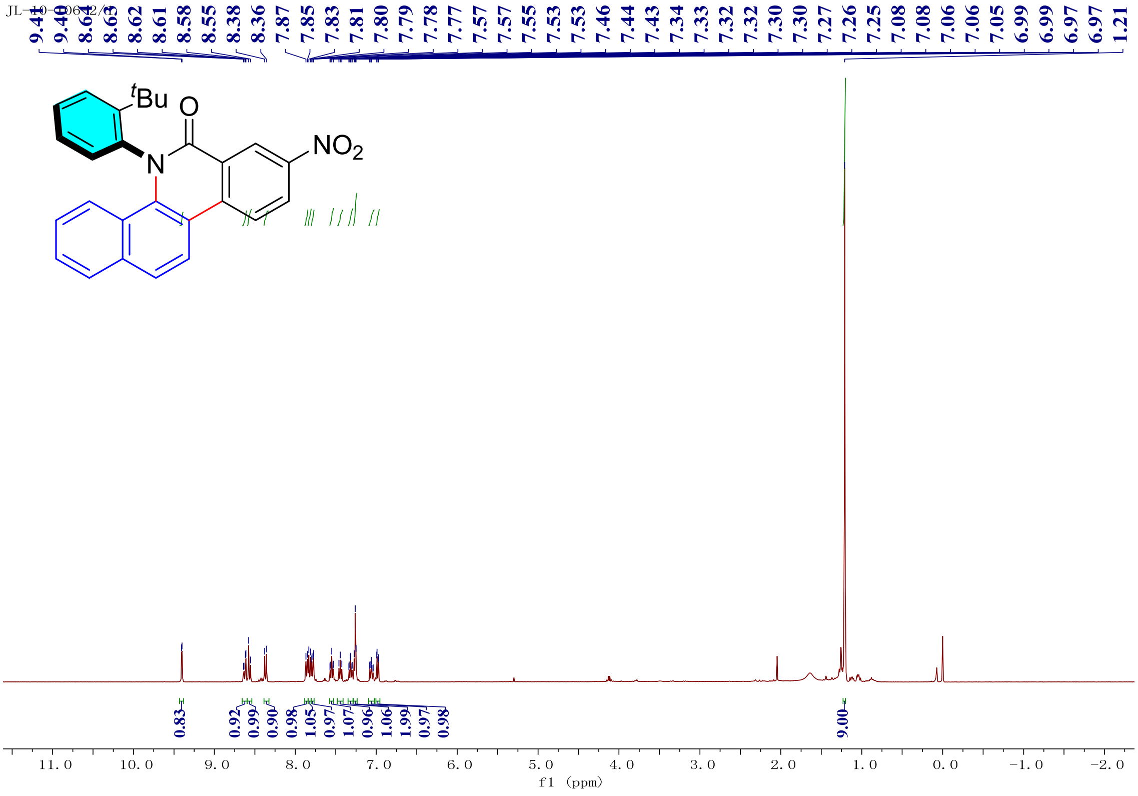

^{13}C NMR (101 MHz, Chloroform-*d*)

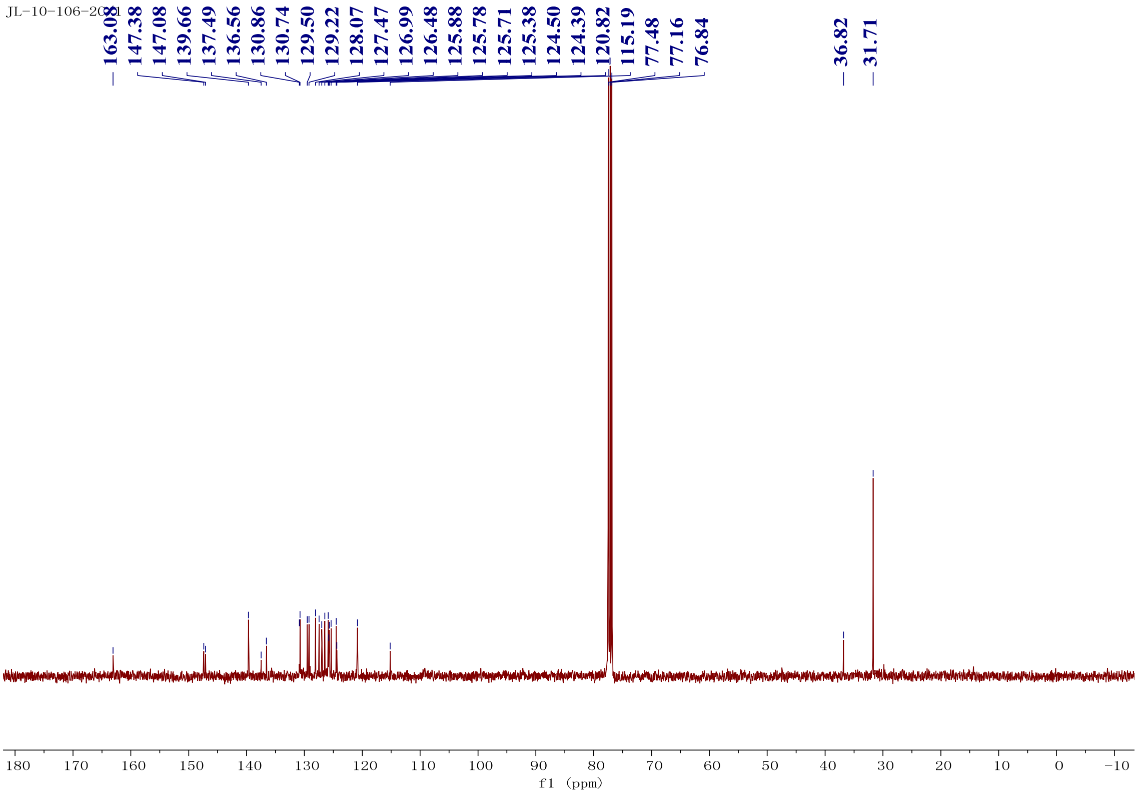

39

^{1}H NMR (600 MHz, Chloroform-*d*)

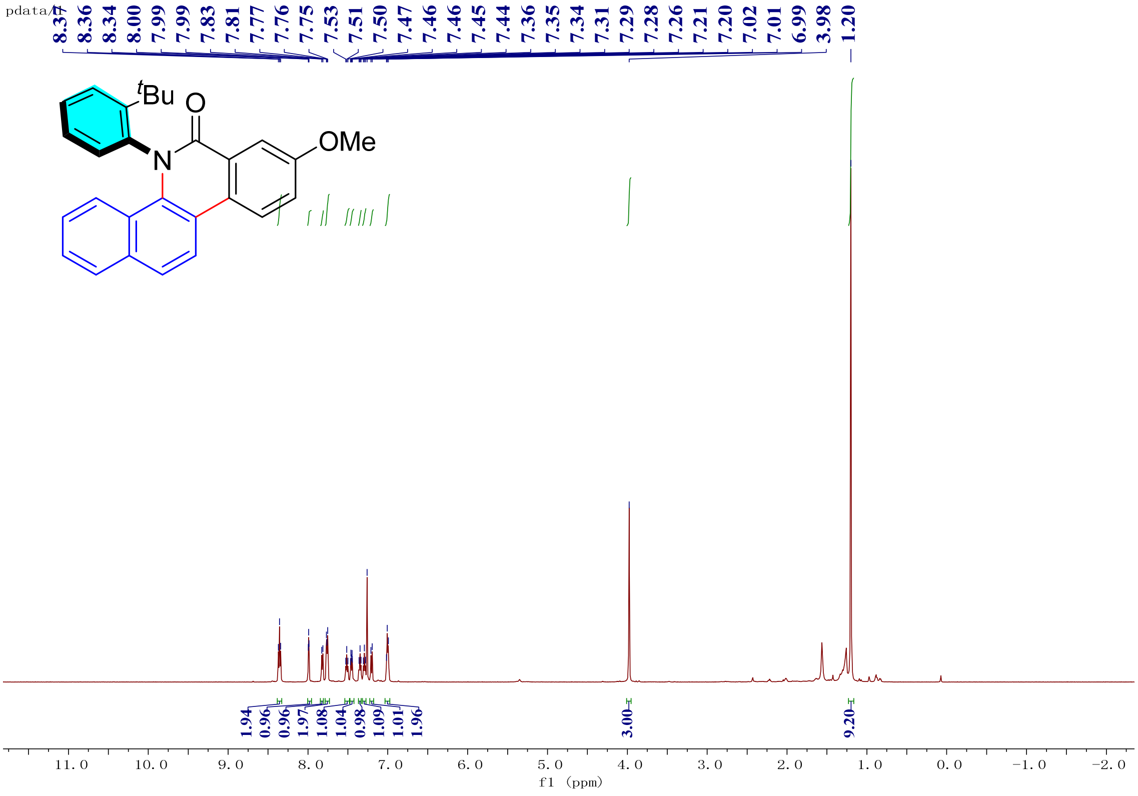

^{13}C NMR (151 MHz, Chloroform-*d*)

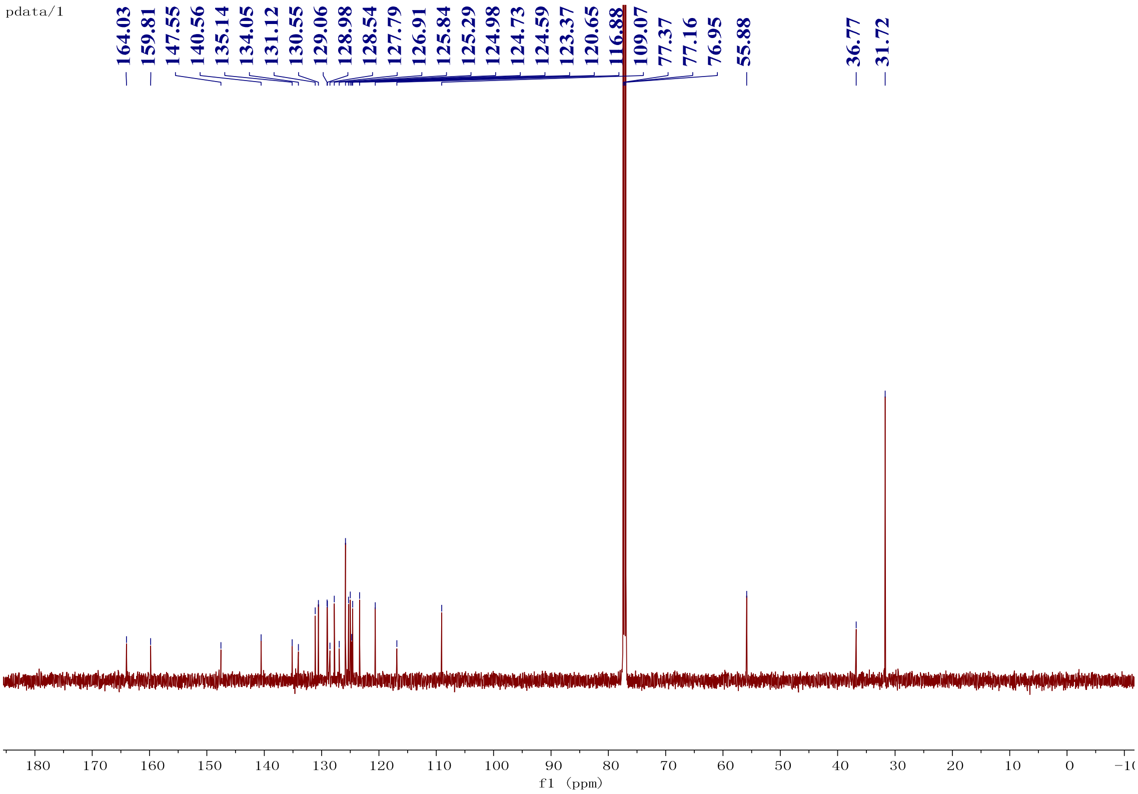

40

^{1}H NMR (400 MHz, Chloroform-*d*)

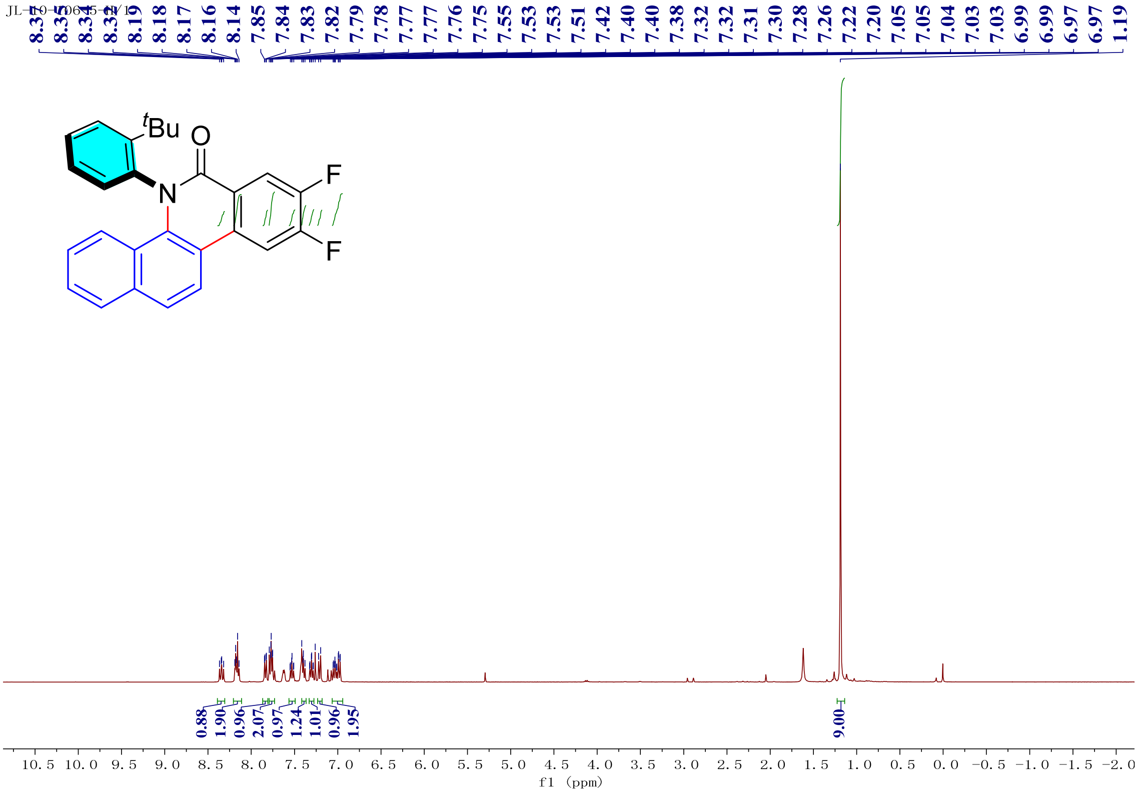

^{13}C NMR (101 MHz, Chloroform-*d*)

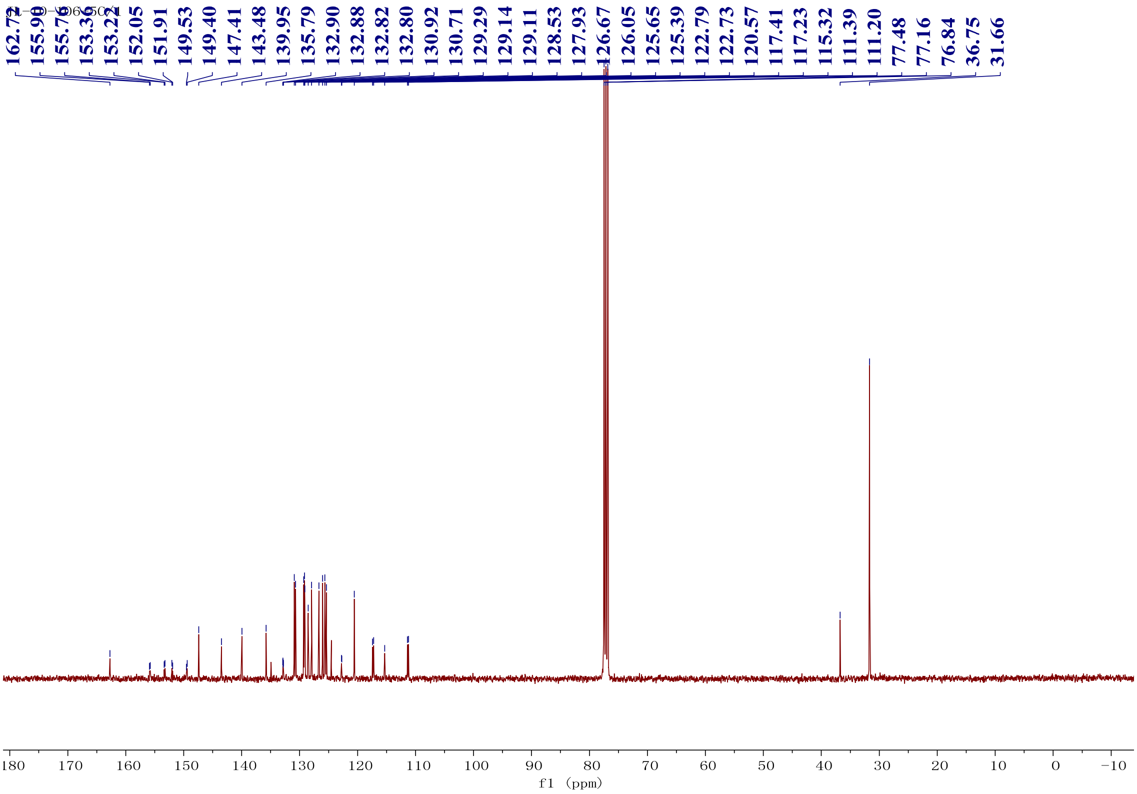

^{19}F NMR (376 MHz, Chloroform-*d*)

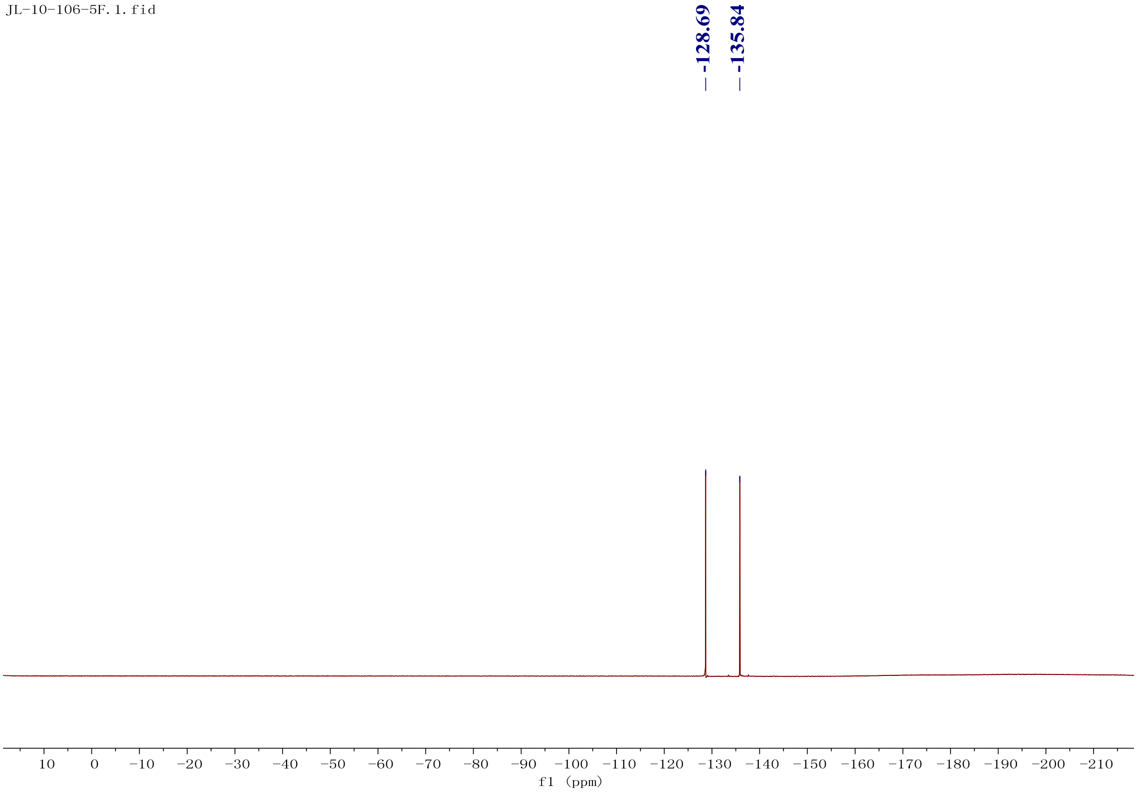

41

<u>**^{1}H NMR (600 MHz, Chloroform-*d*)**</u>

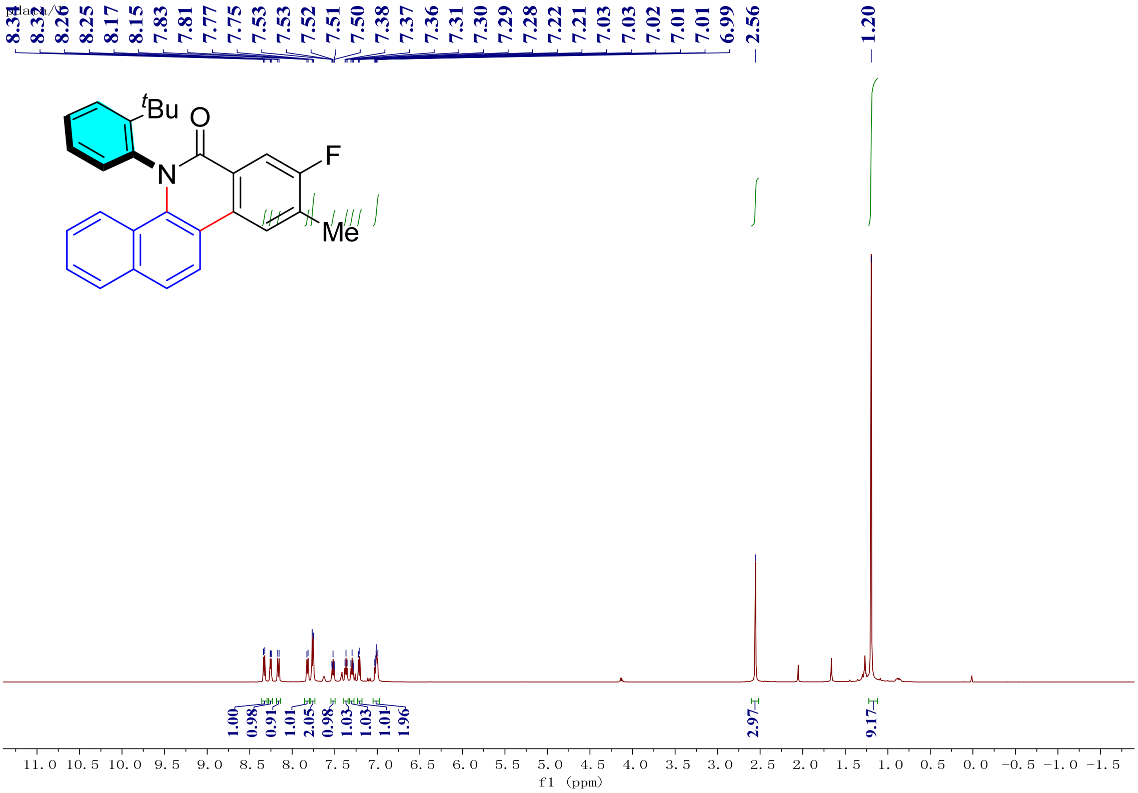

<u>**^{13}C NMR (151 MHz, Chloroform-*d*)**</u>

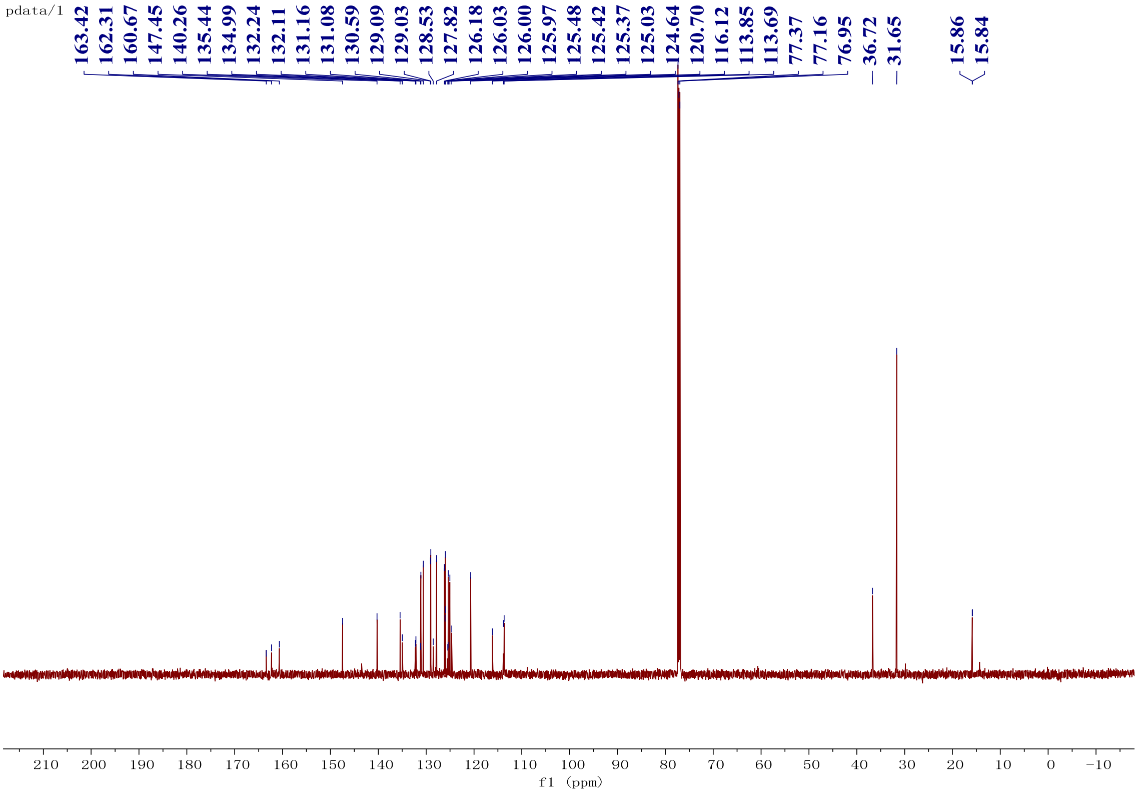

^{19}F NMR (565 MHz, Chloroform-*d*)

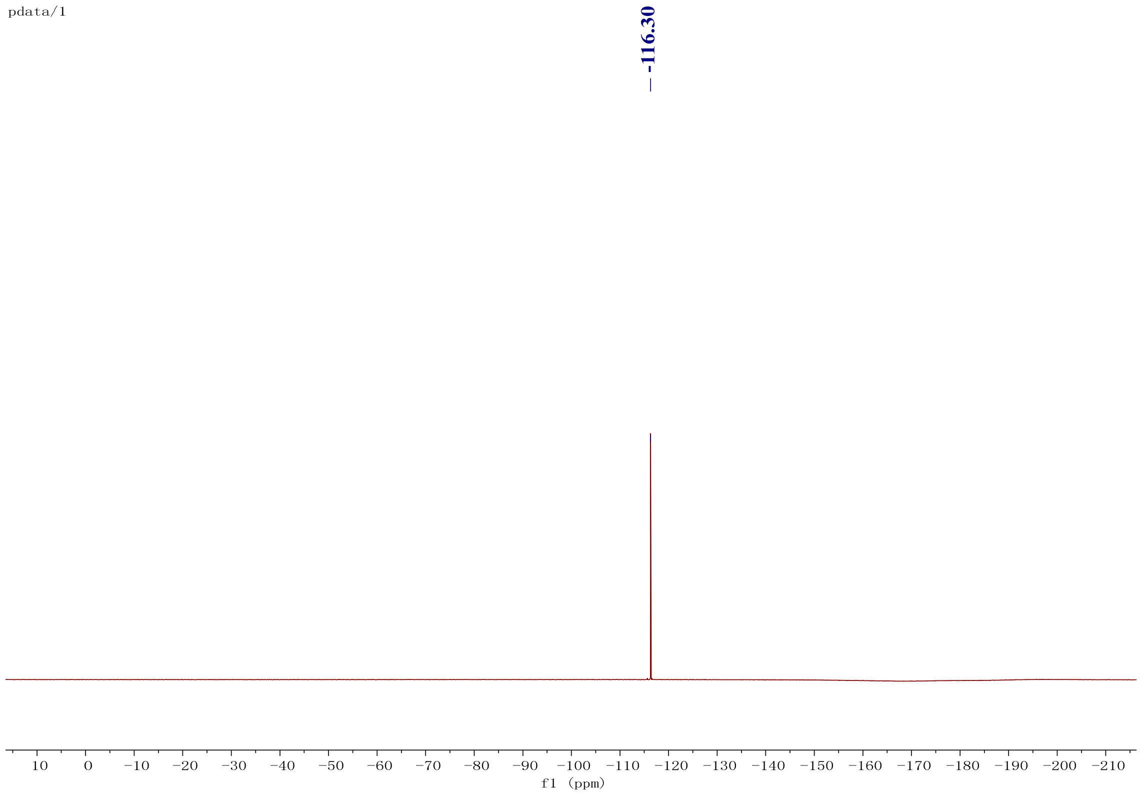

42

^{1}H NMR (600 MHz, Chloroform-*d*)

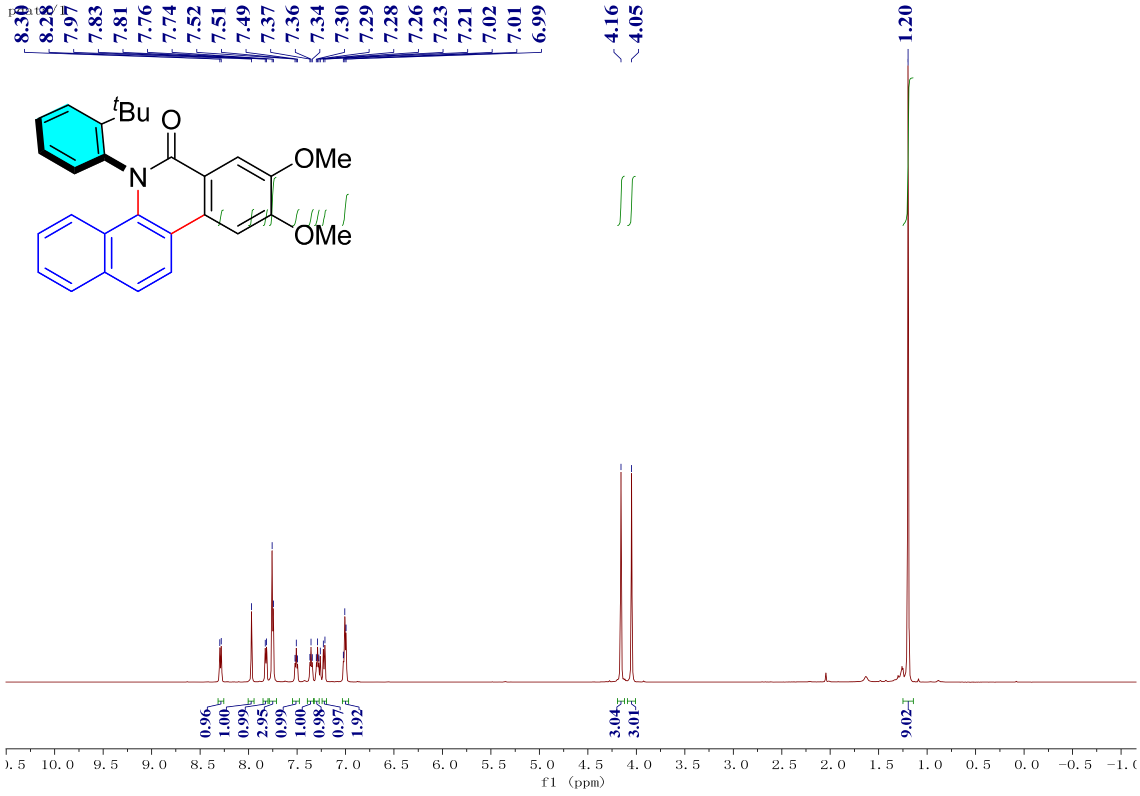

^{13}C NMR (151 MHz, Chloroform-*d*)

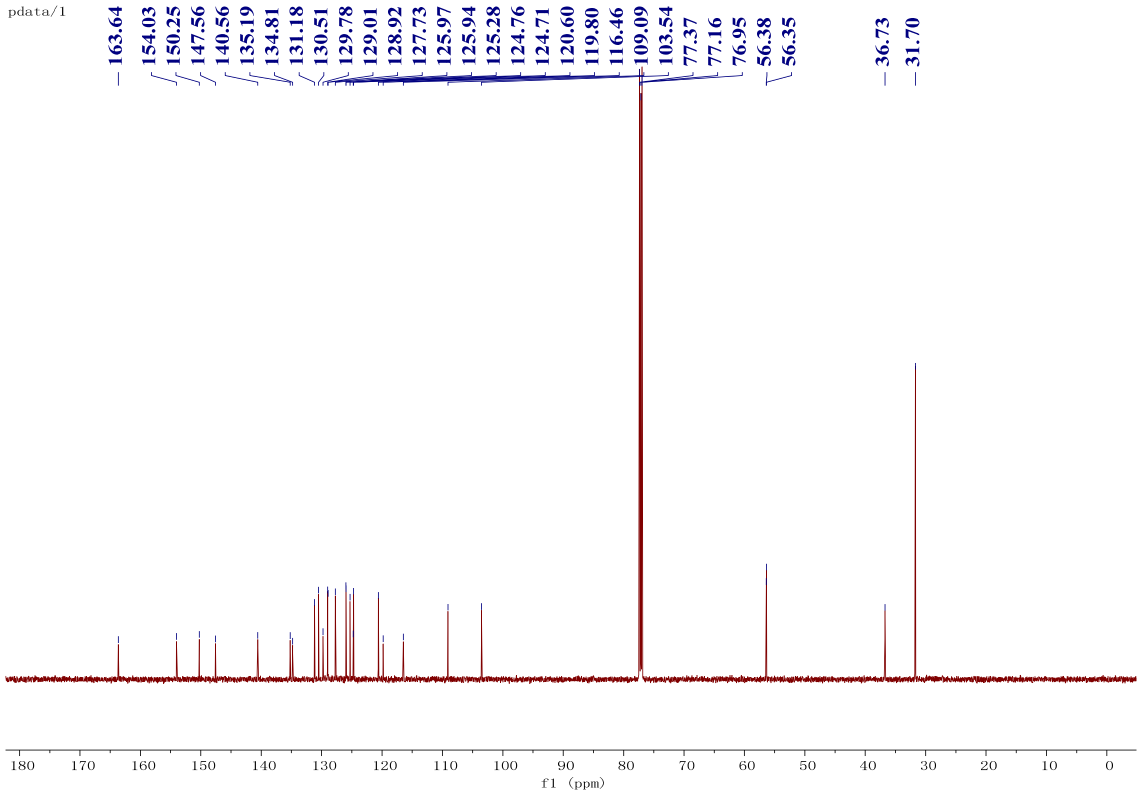

43

^{1}H NMR (600 MHz, Chloroform-*d*)

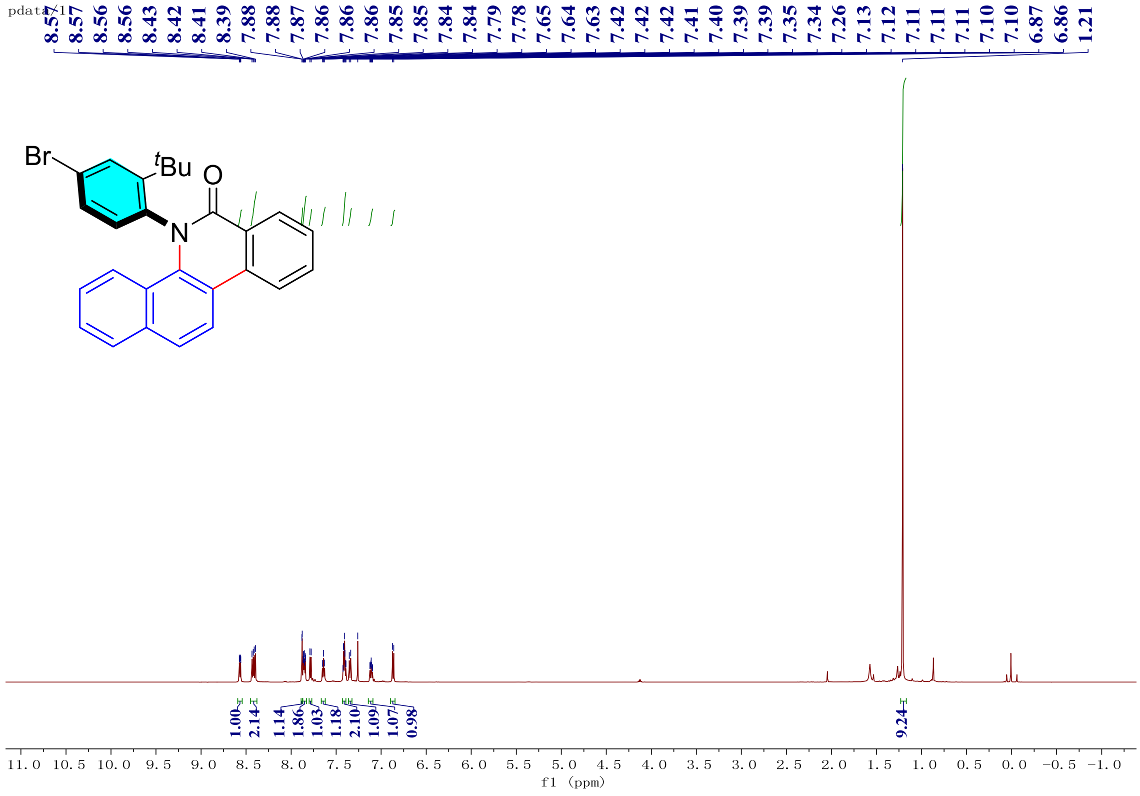

^{13}C NMR (151 MHz, Chloroform-*d*)

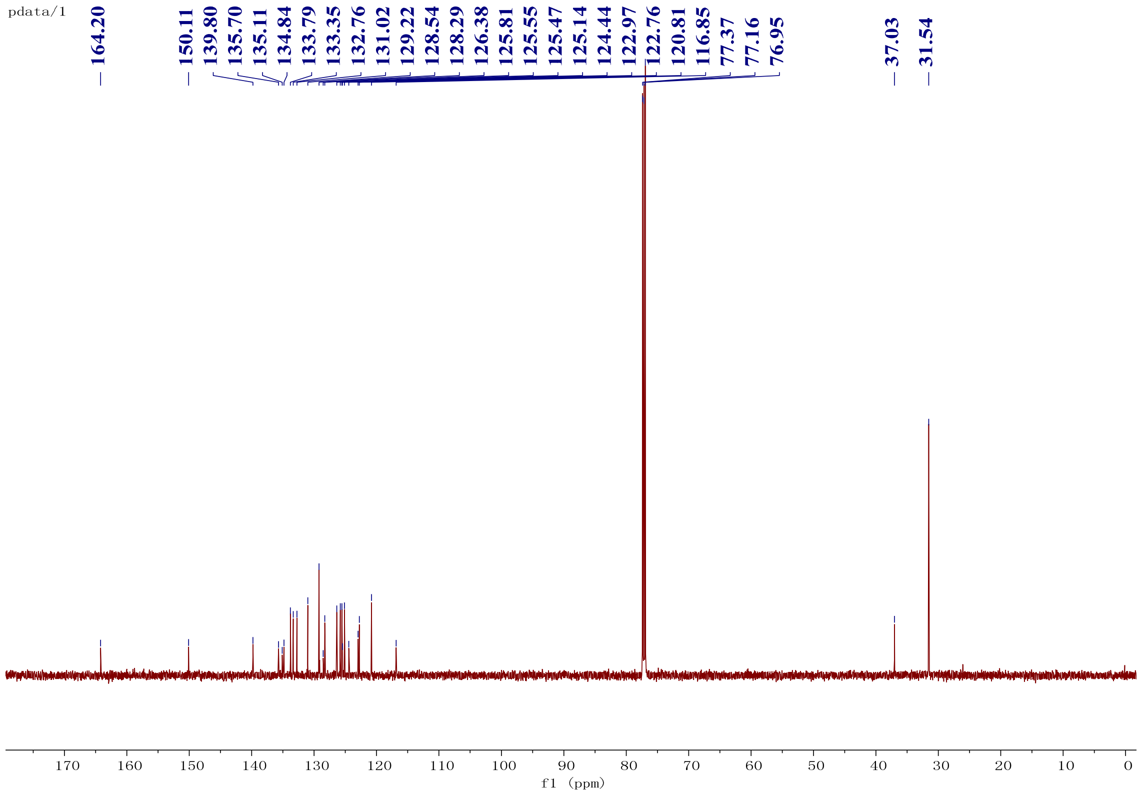

44

^{1}H NMR (600 MHz, Chloroform-*d*)

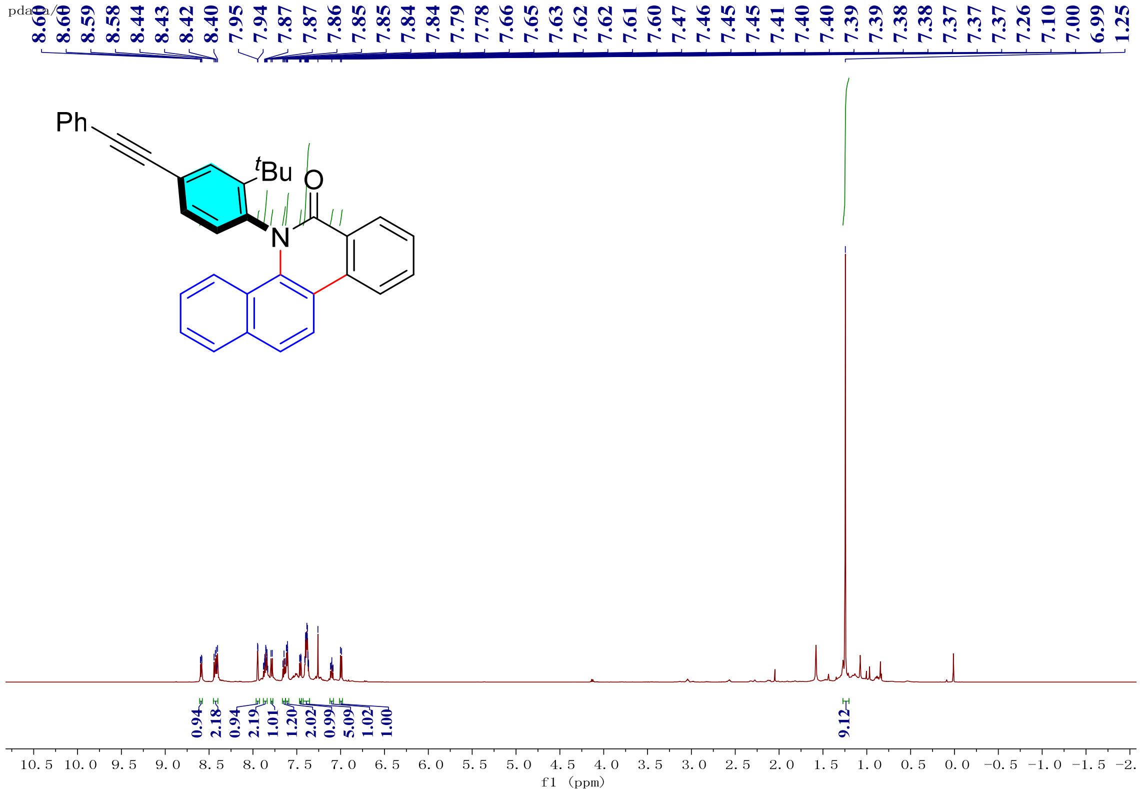

^{13}C NMR (151 MHz, Chloroform-*d*)

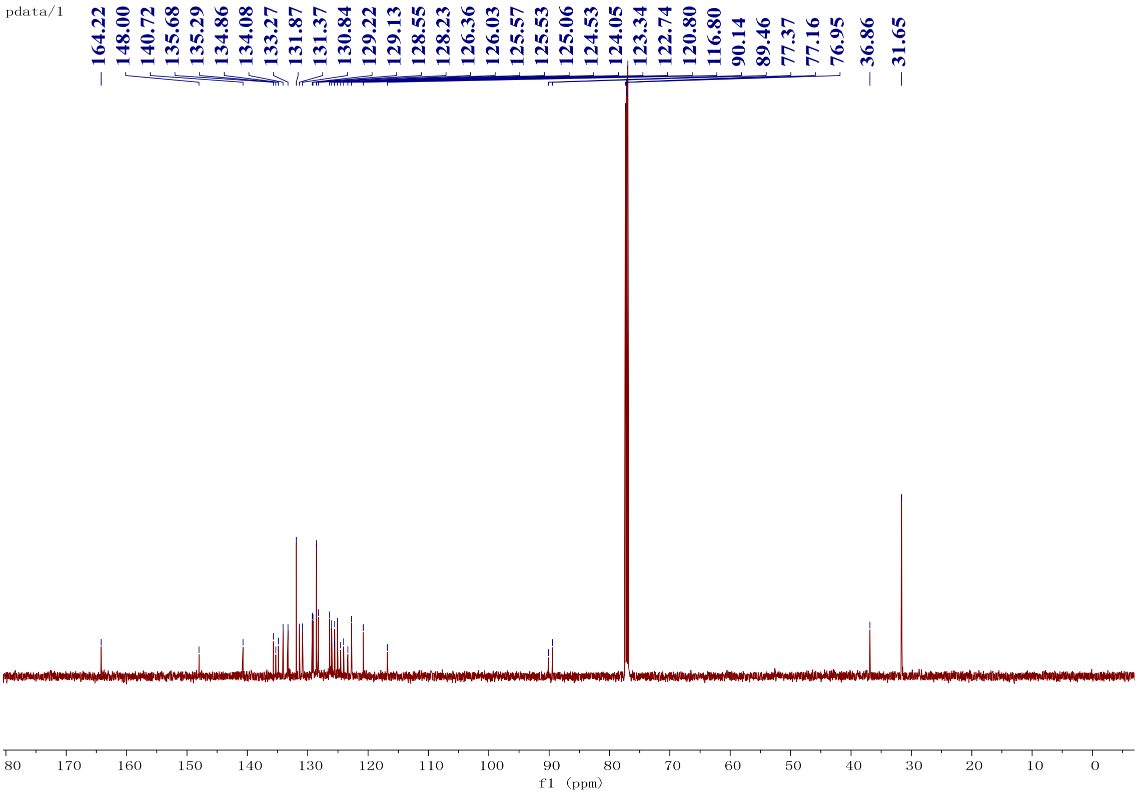

45

^{1}H NMR (600 MHz, Chloroform-*d*)

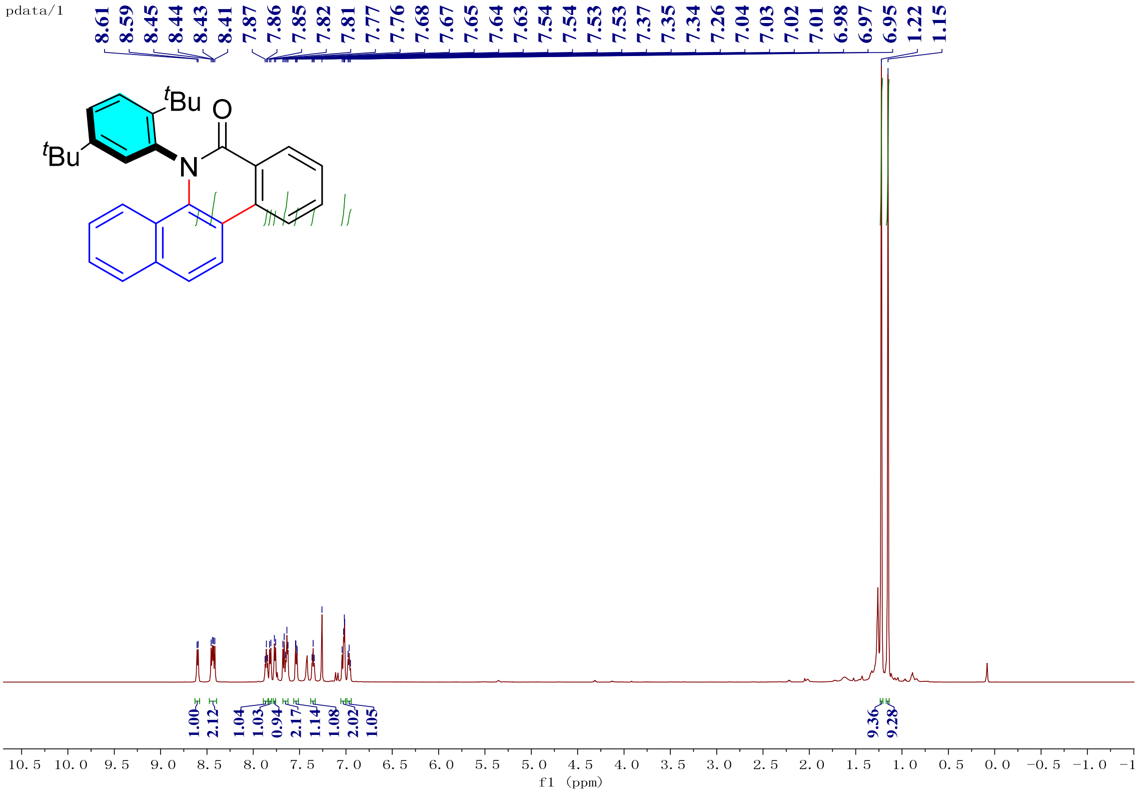

^{13}C NMR (151 MHz, Chloroform-*d*)

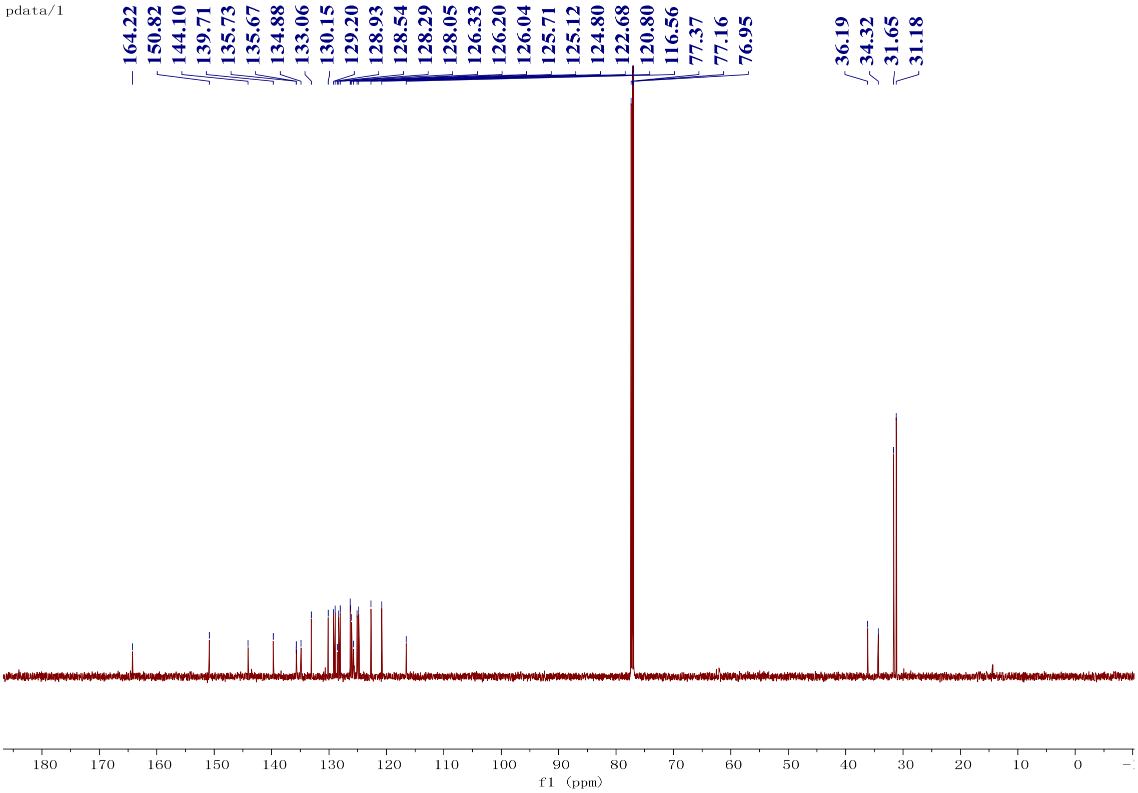

46

^{1}H NMR (600 MHz, Chloroform-*d*)

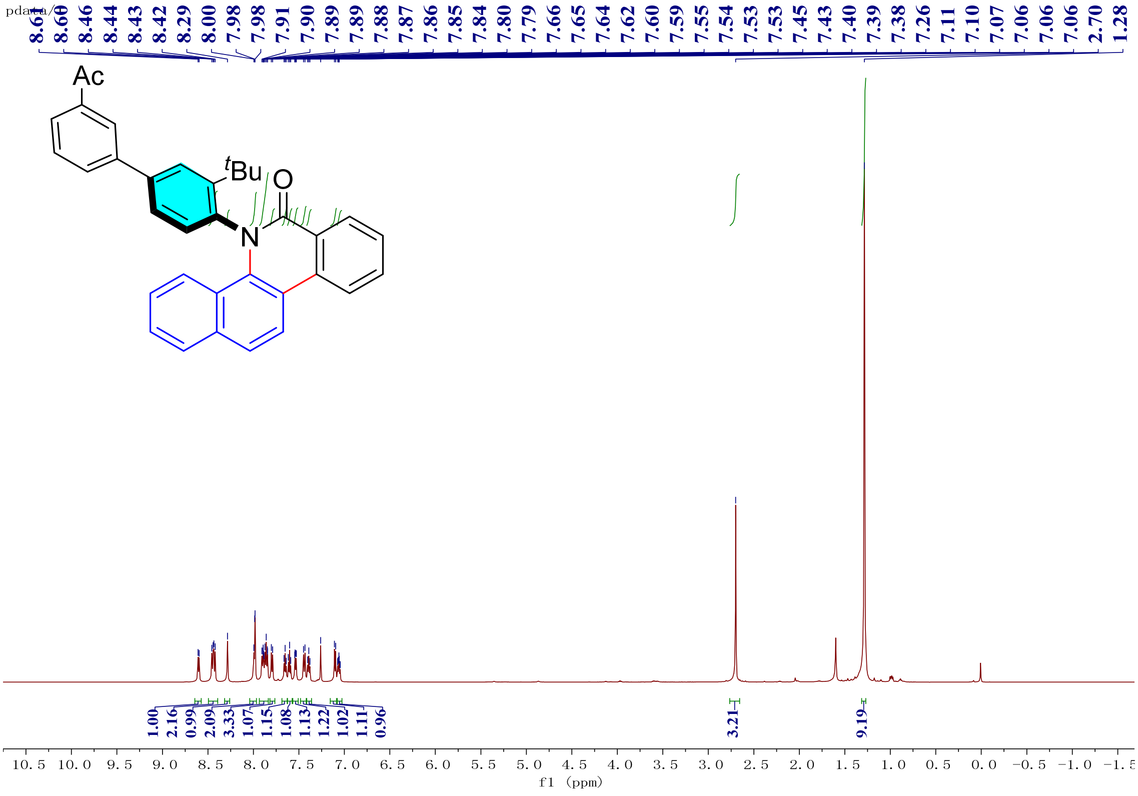

^{13}C NMR (151 MHz, Chloroform-*d*)

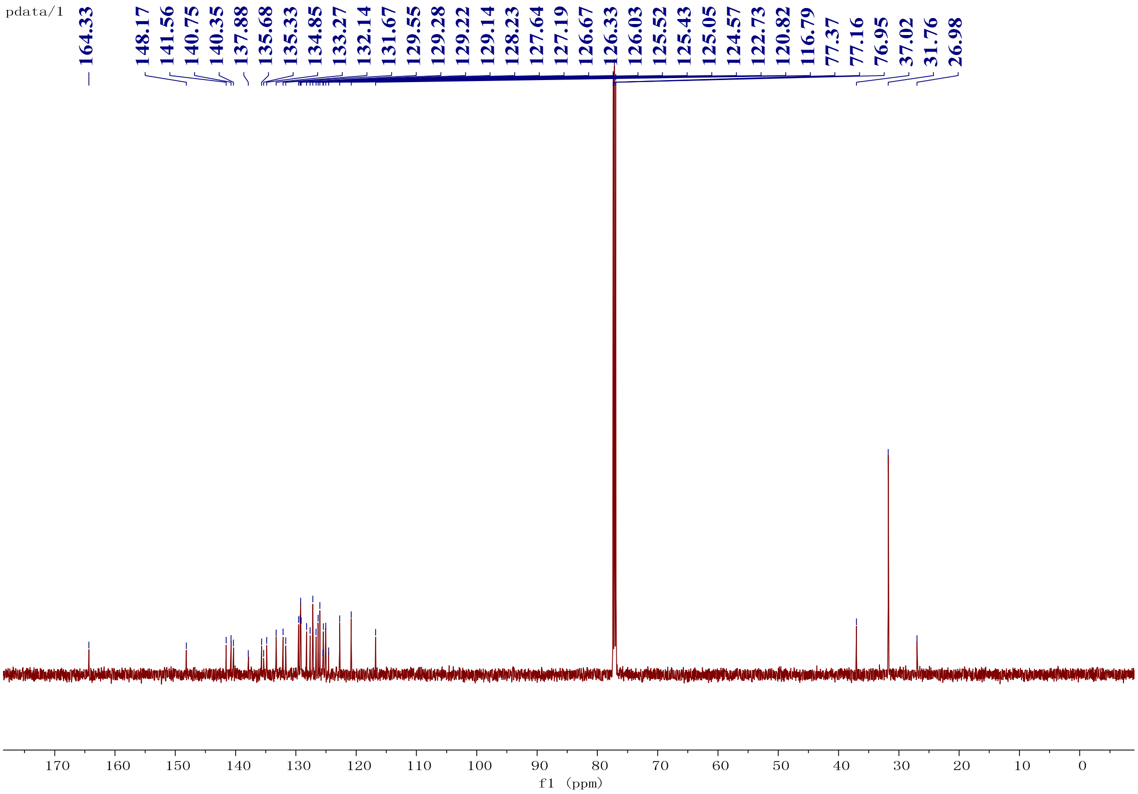

47

^{1}H NMR (600 MHz, Chloroform-*d*)

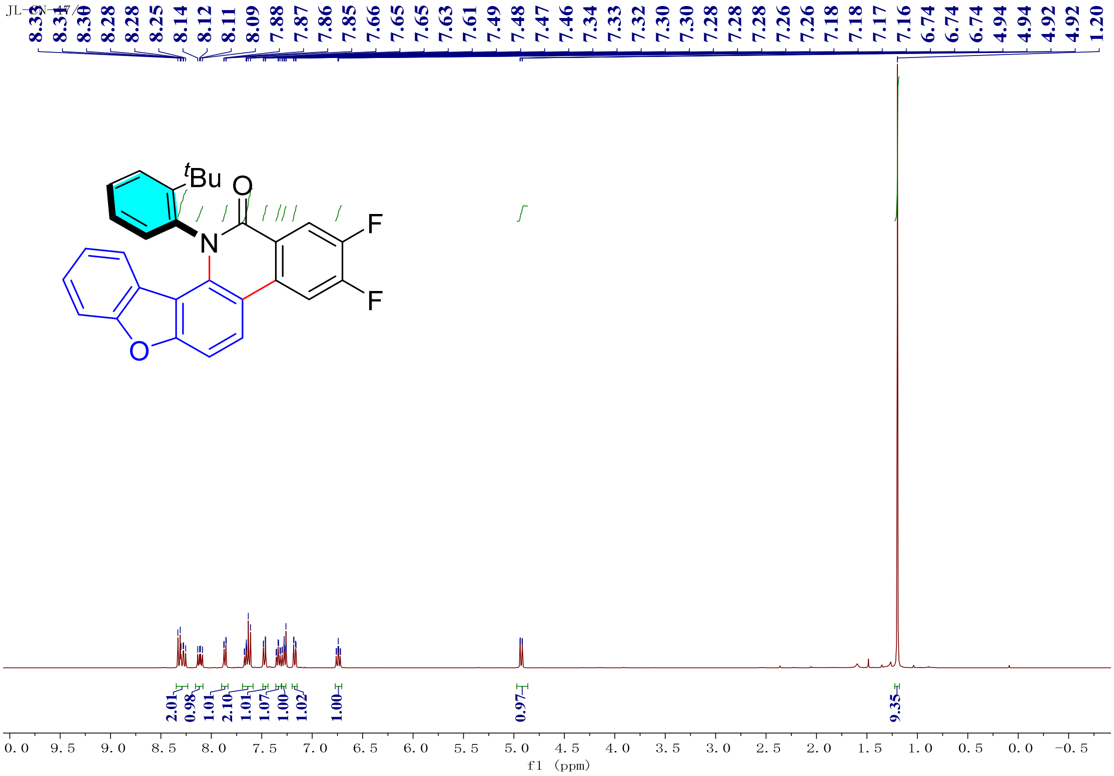

^{13}C NMR (151 MHz, Chloroform-*d*)

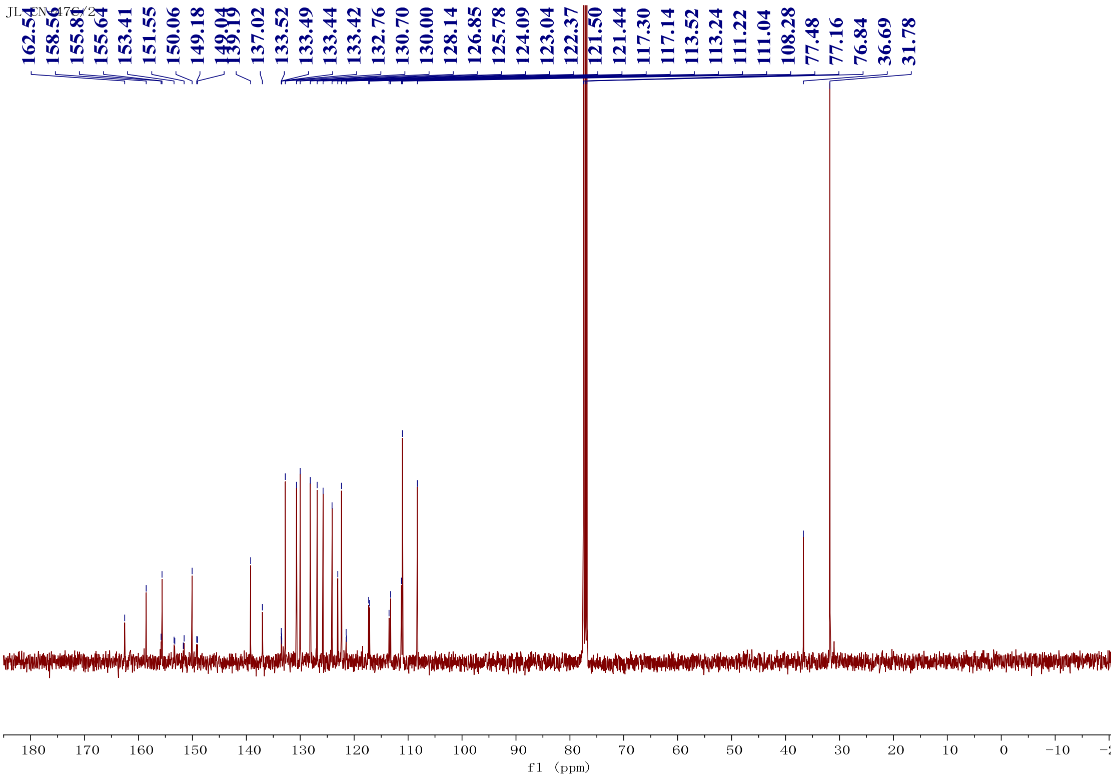

^{19}F NMR (376 MHz, Chloroform-*d*)

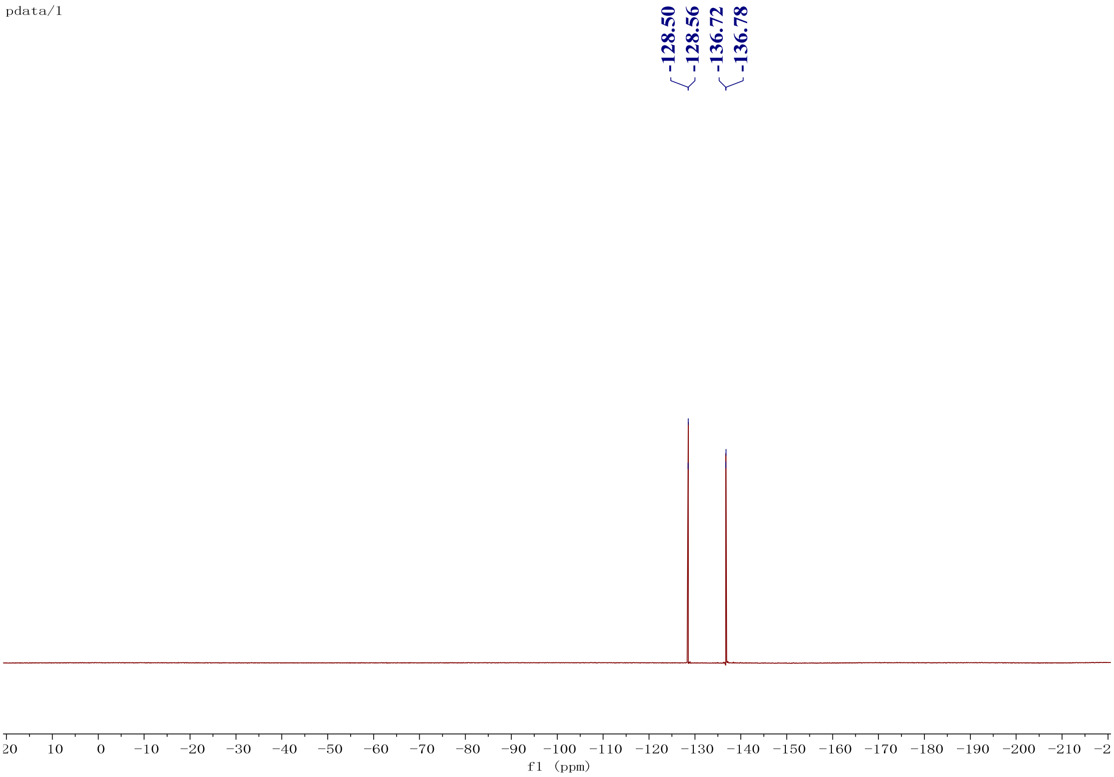

48

^{1}H NMR (600 MHz, Chloroform-*d*)

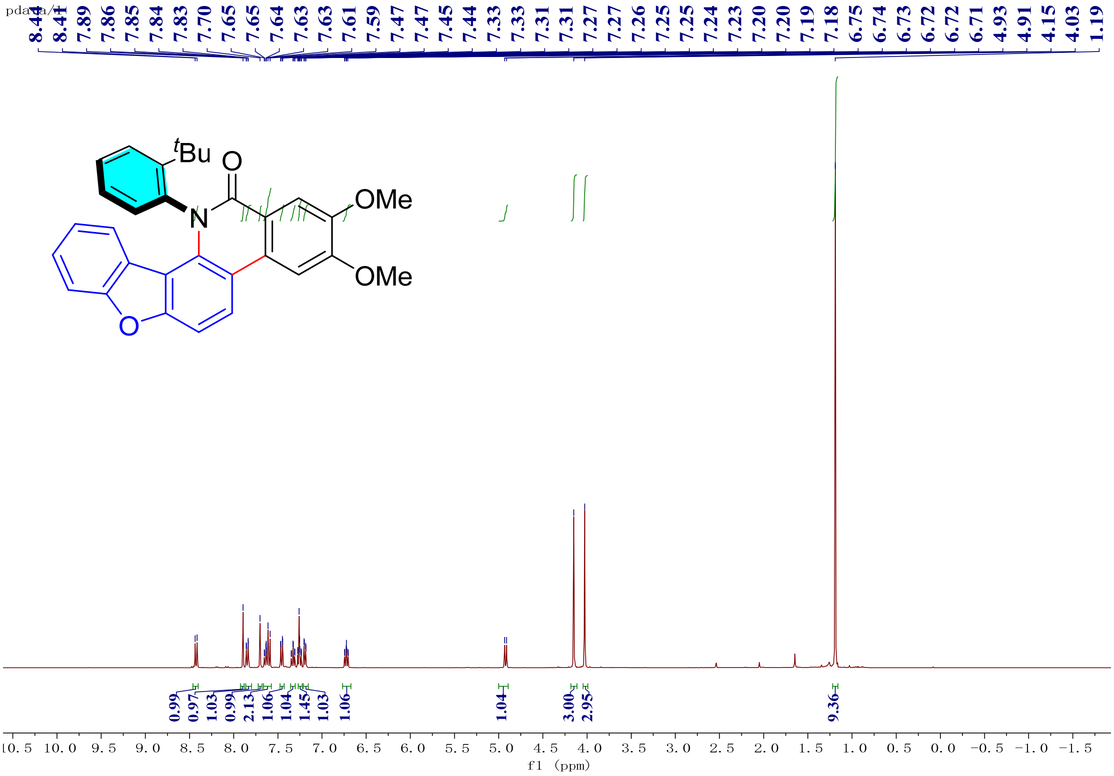

^{13}C NMR (151 MHz, Chloroform-*d*)

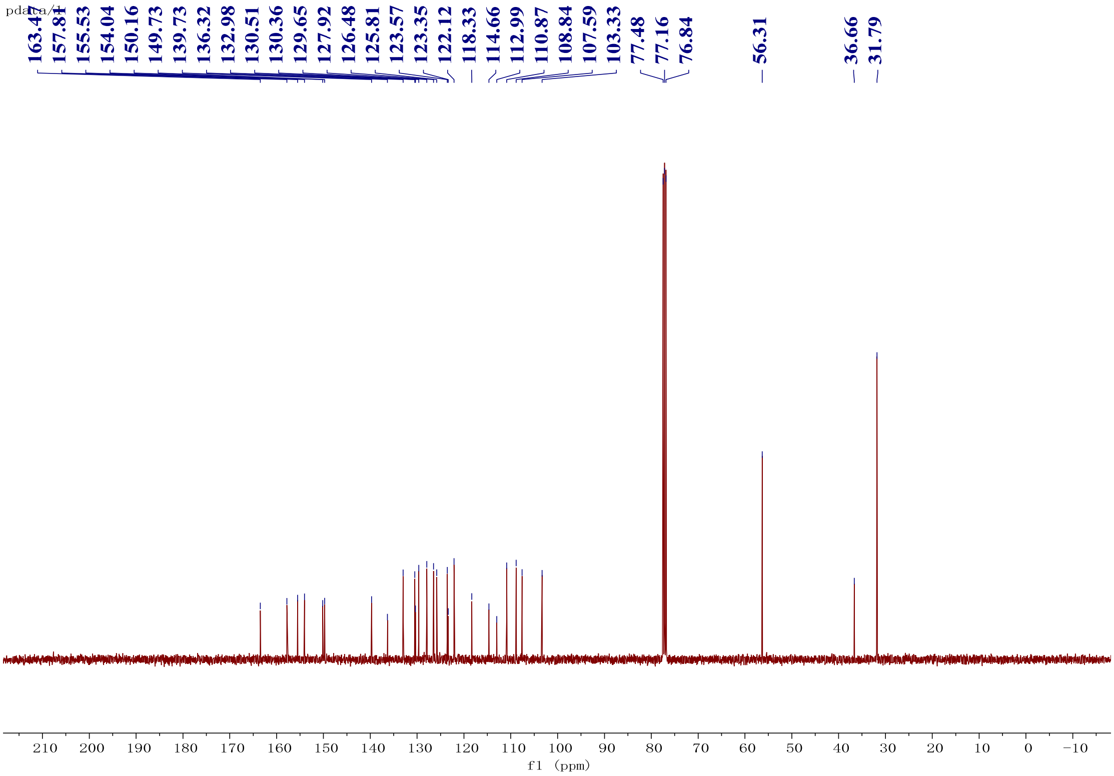

49

^{1}H NMR (600 MHz, Chloroform-*d*)

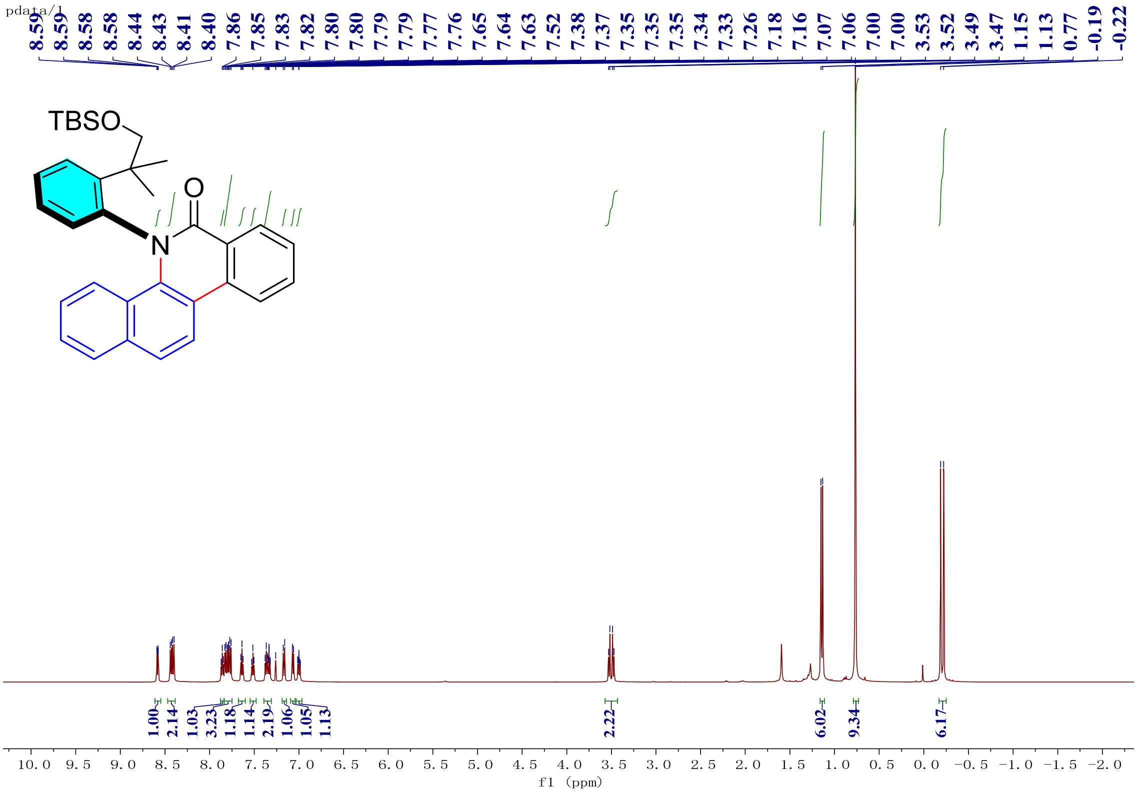

^{13}C NMR (151 MHz, Chloroform-*d*)

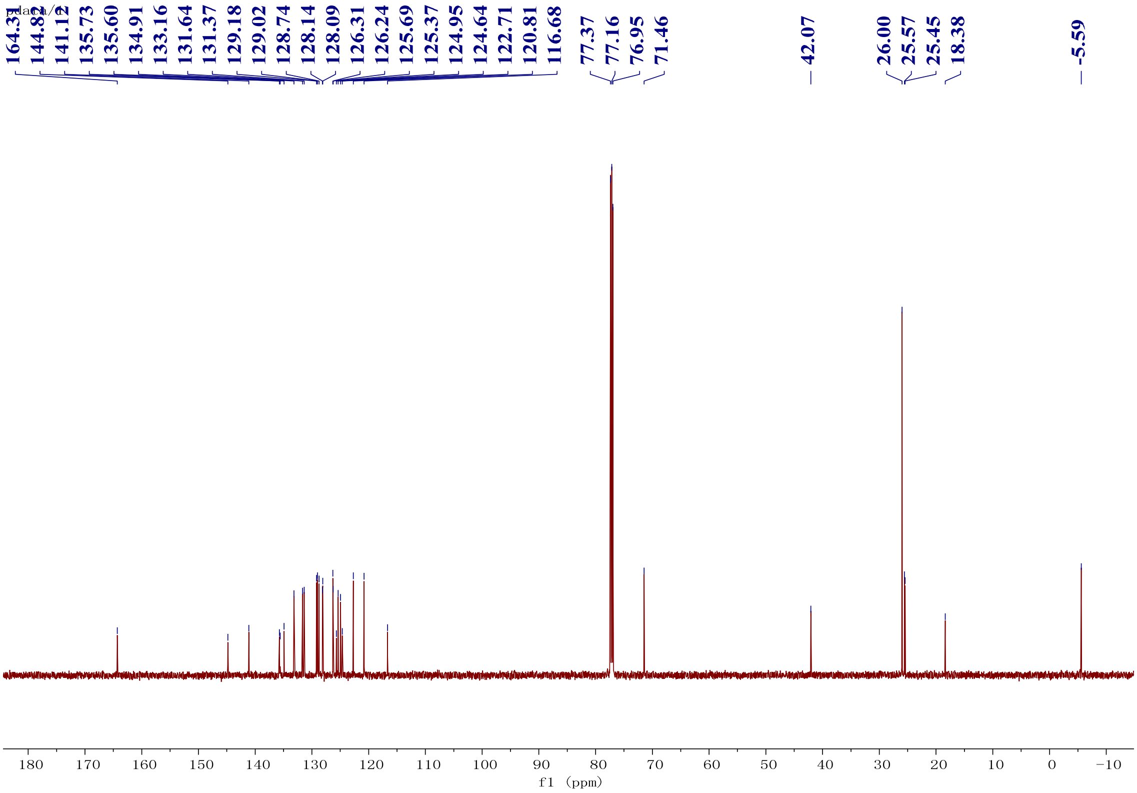

50

^{1}H NMR (600 MHz, Chloroform-*d*)

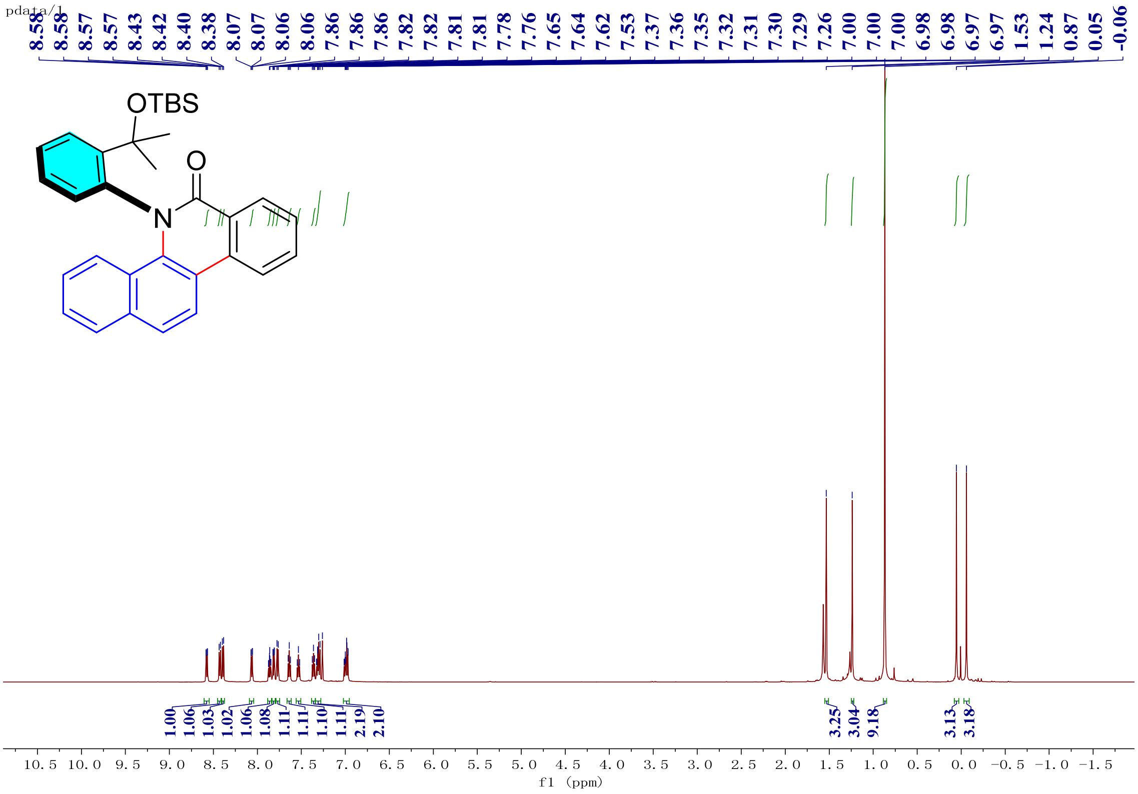

^{13}C NMR (151 MHz, Chloroform-*d*)

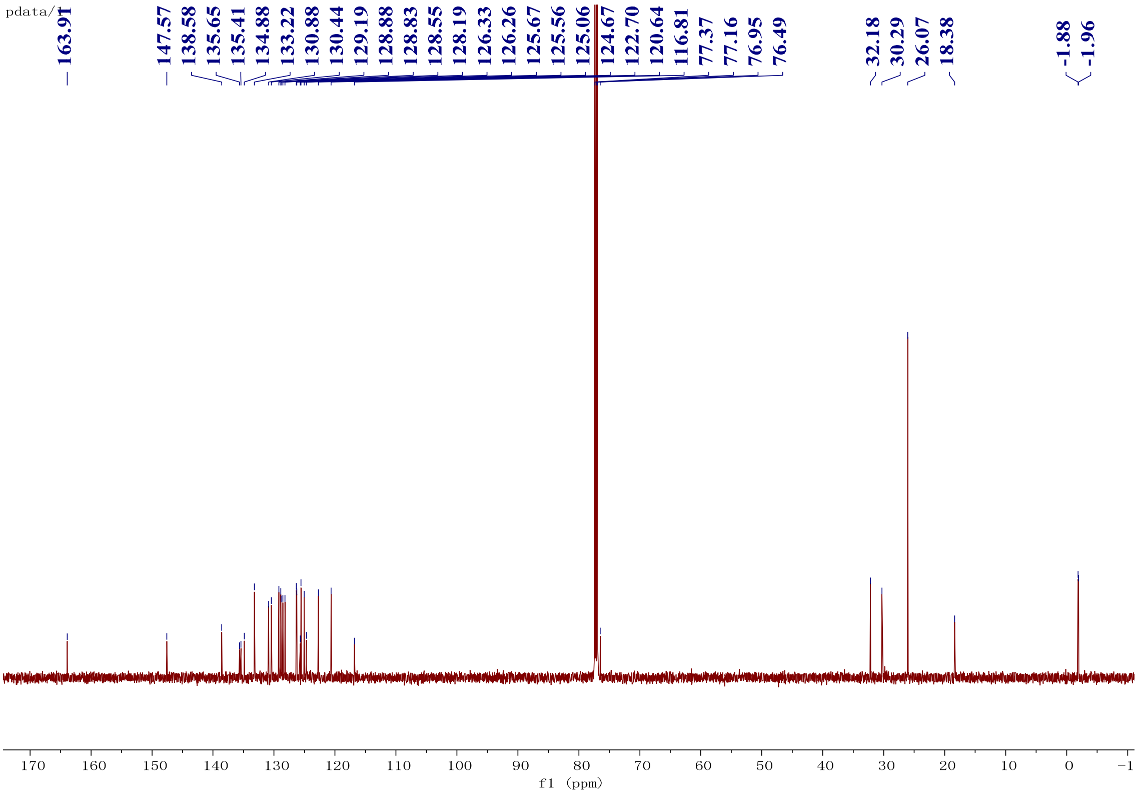

51

^{1}H NMR (600 MHz, Chloroform-*d*)

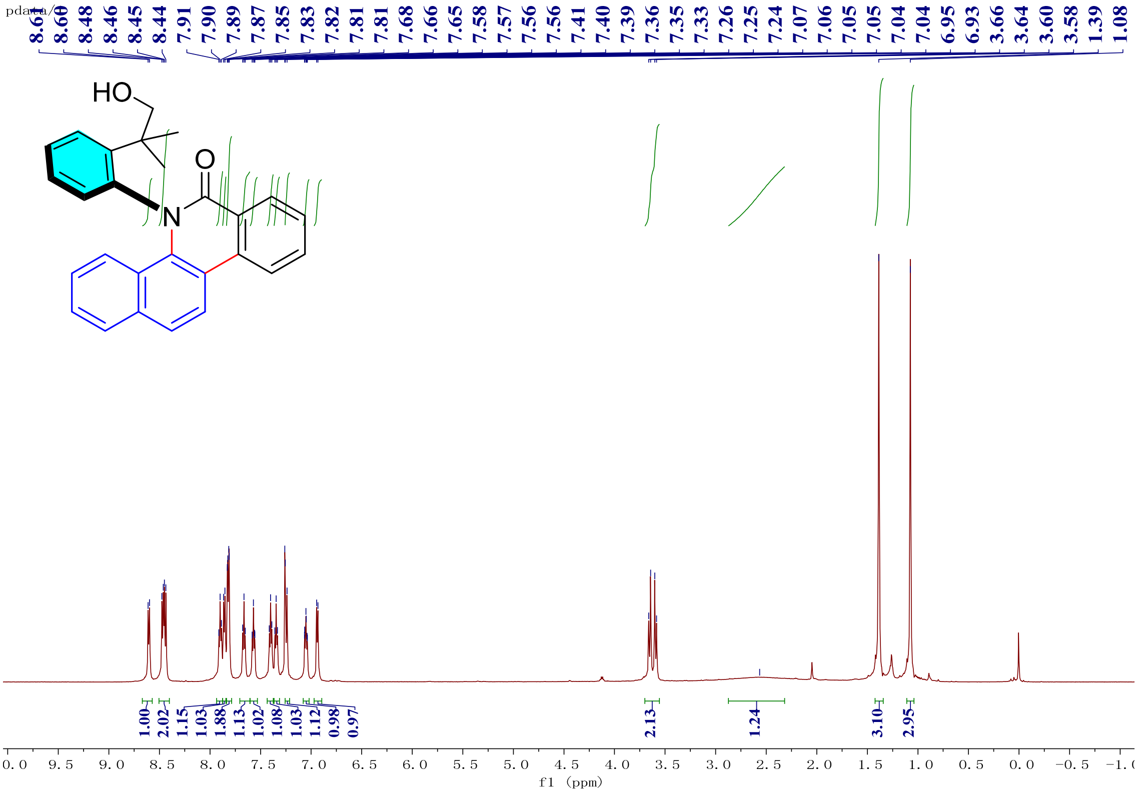

^{13}C NMR (151 MHz, Chloroform-*d*)

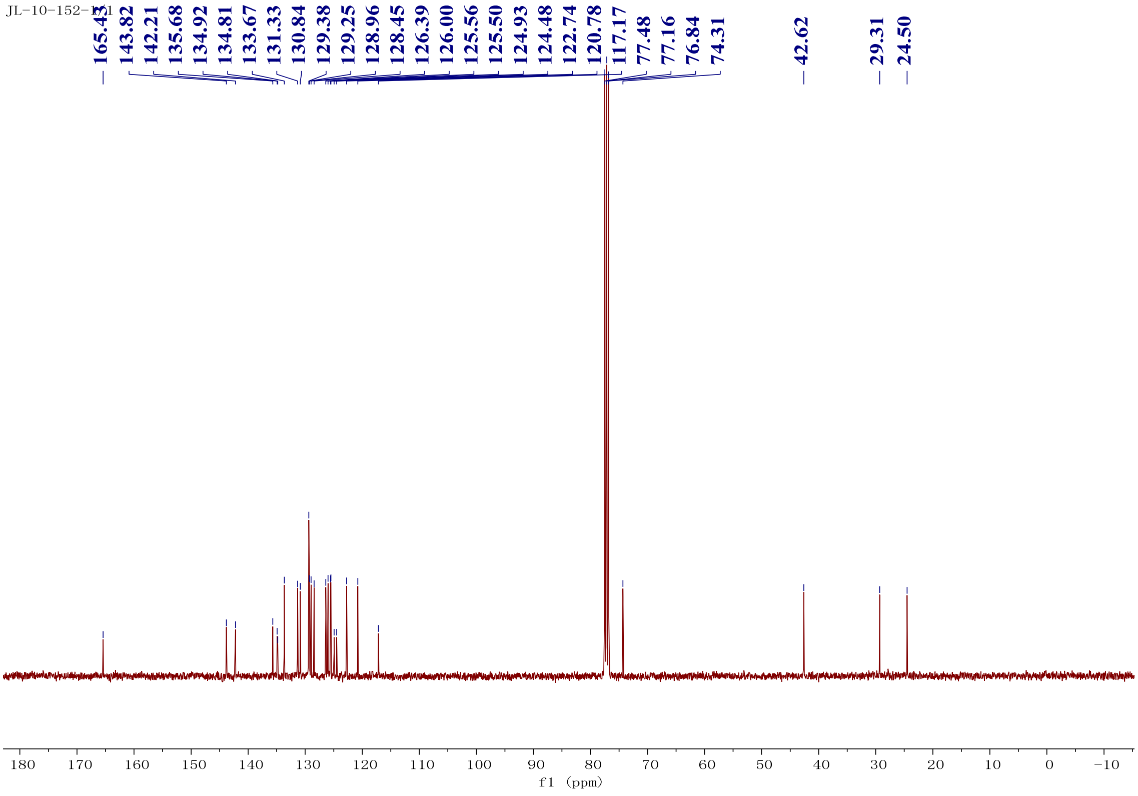

52

^{1}H NMR (400 MHz, Chloroform-*d*)

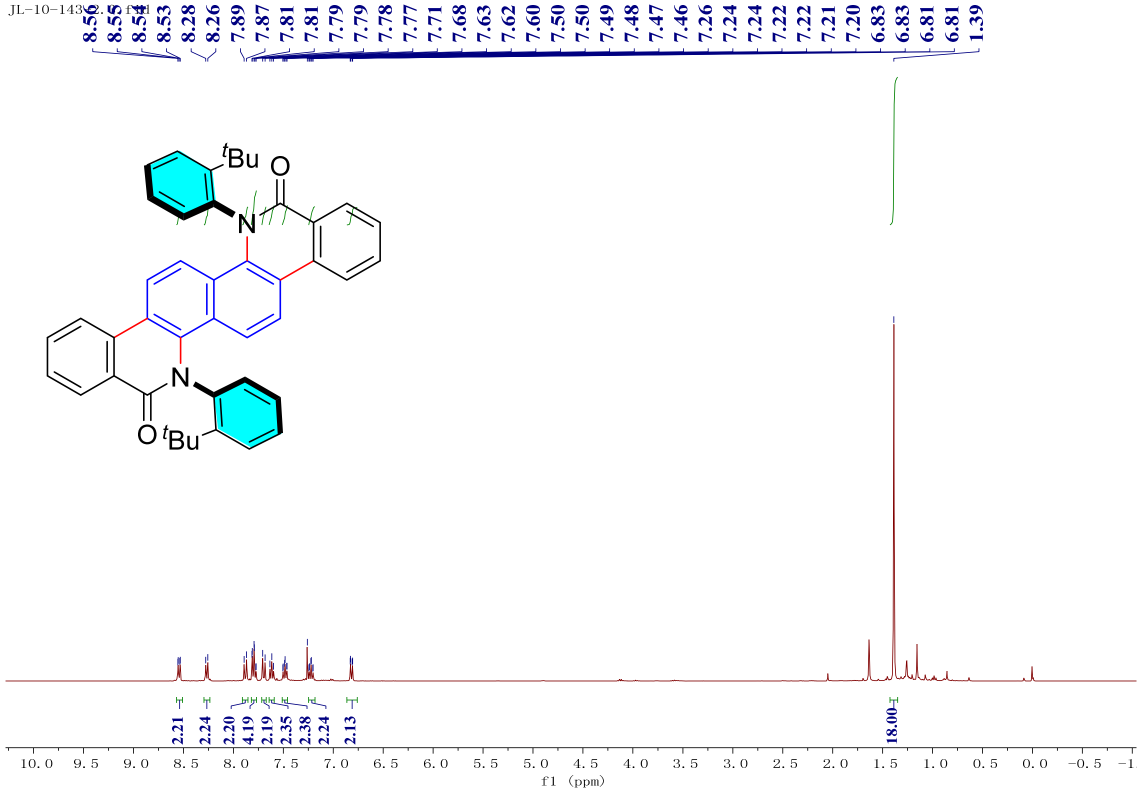

^{13}C NMR (101 MHz, Chloroform-*d*)

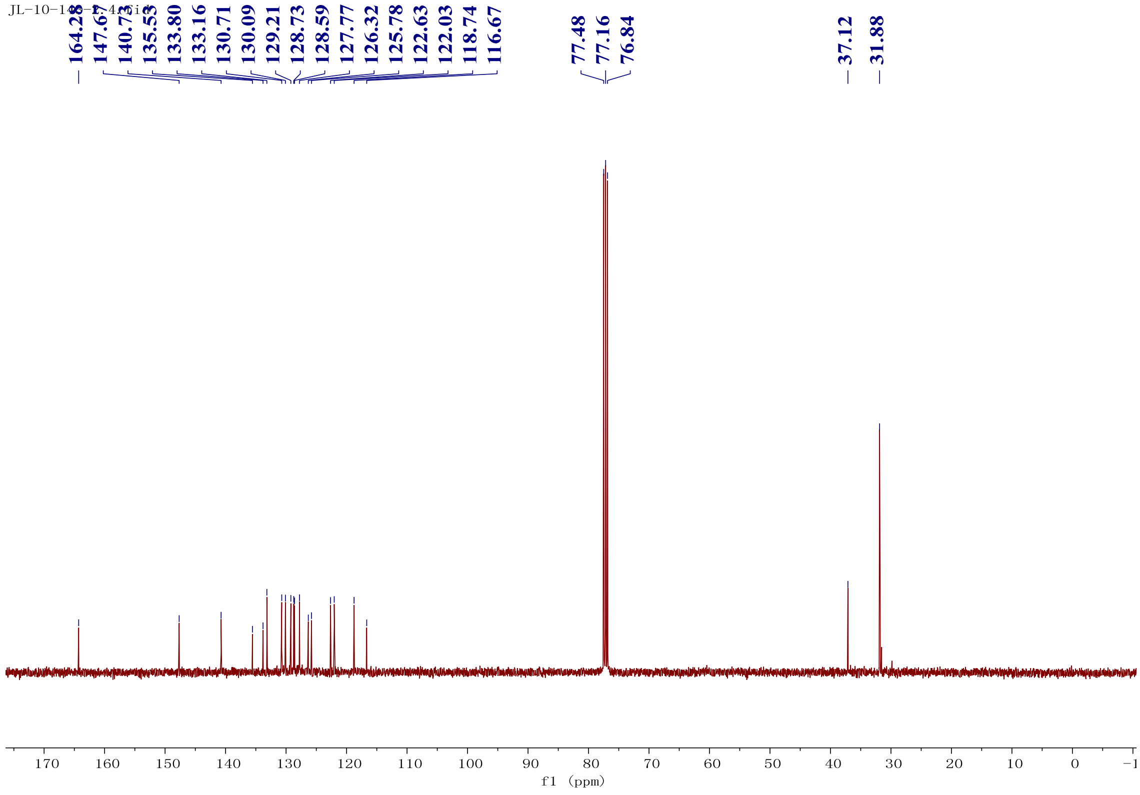

53

^{1}H NMR (400 MHz, Chloroform-*d*)

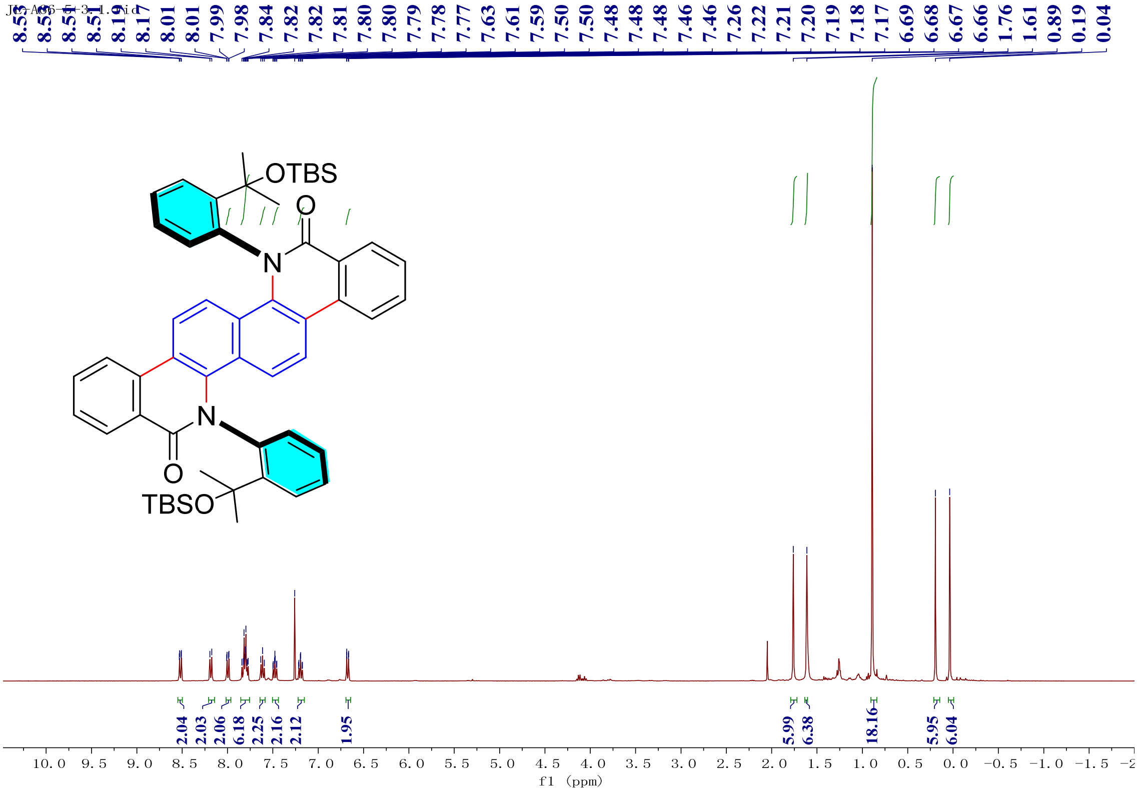

^{13}C NMR (101 MHz, Chloroform-*d*)

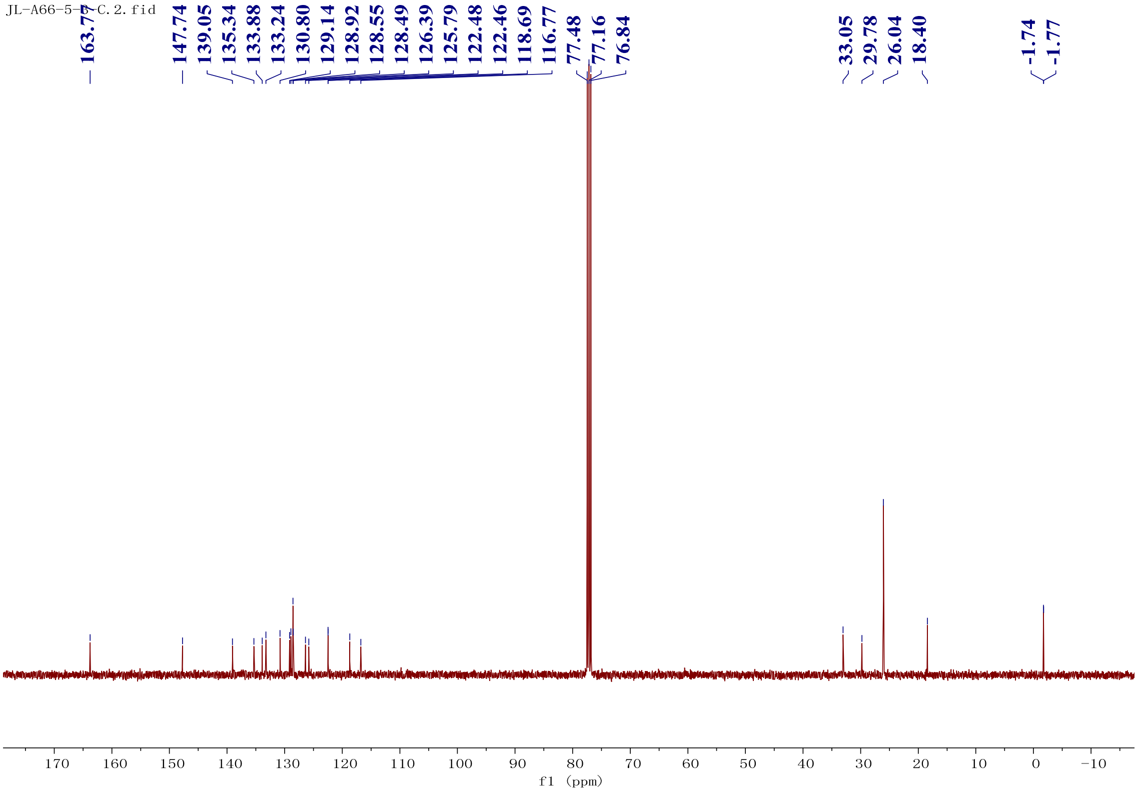

53′

^{1}H NMR (400 MHz, Chloroform-*d*)

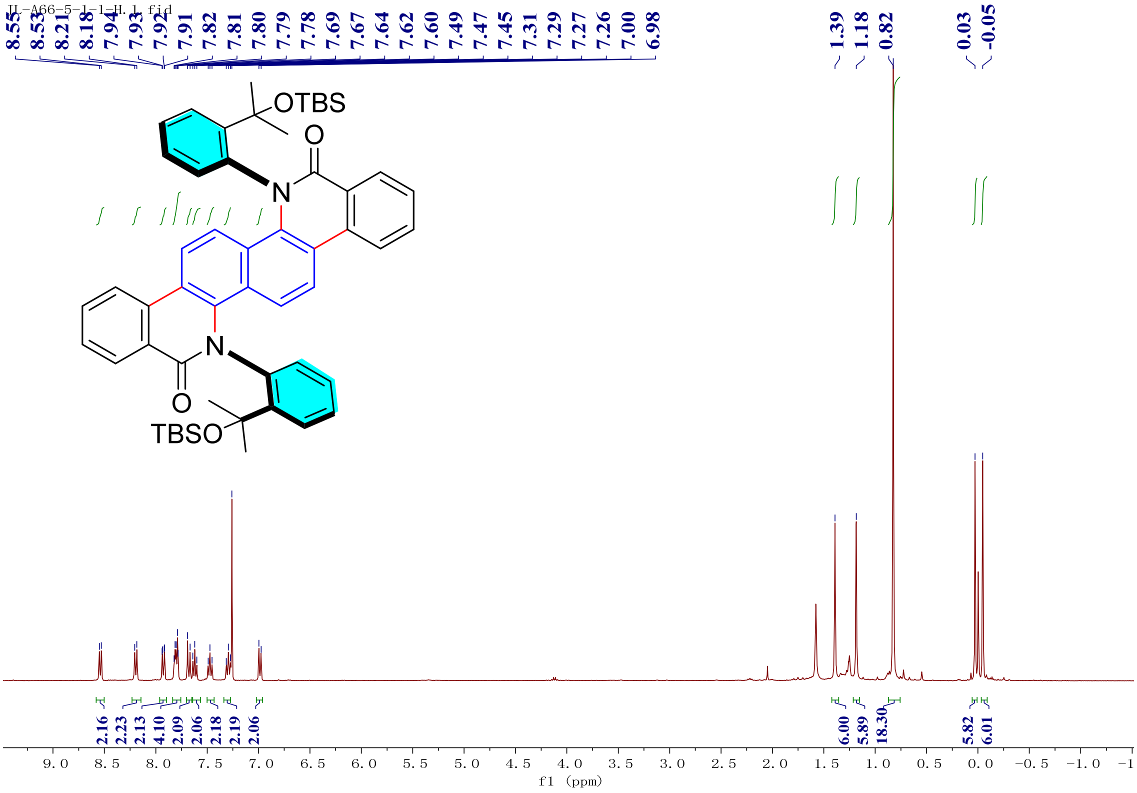

^{13}C NMR (101 MHz, Chloroform-*d*)

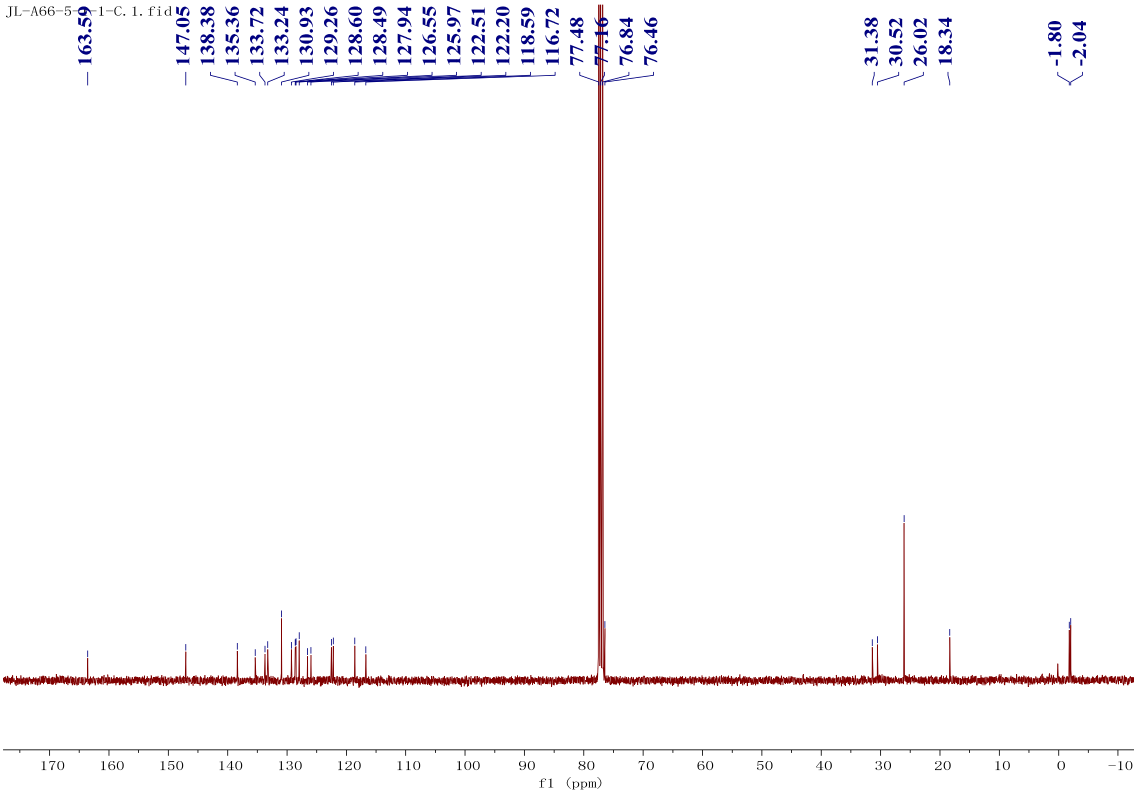

54

^{1}H NMR (400 MHz, Chloroform-*d*)

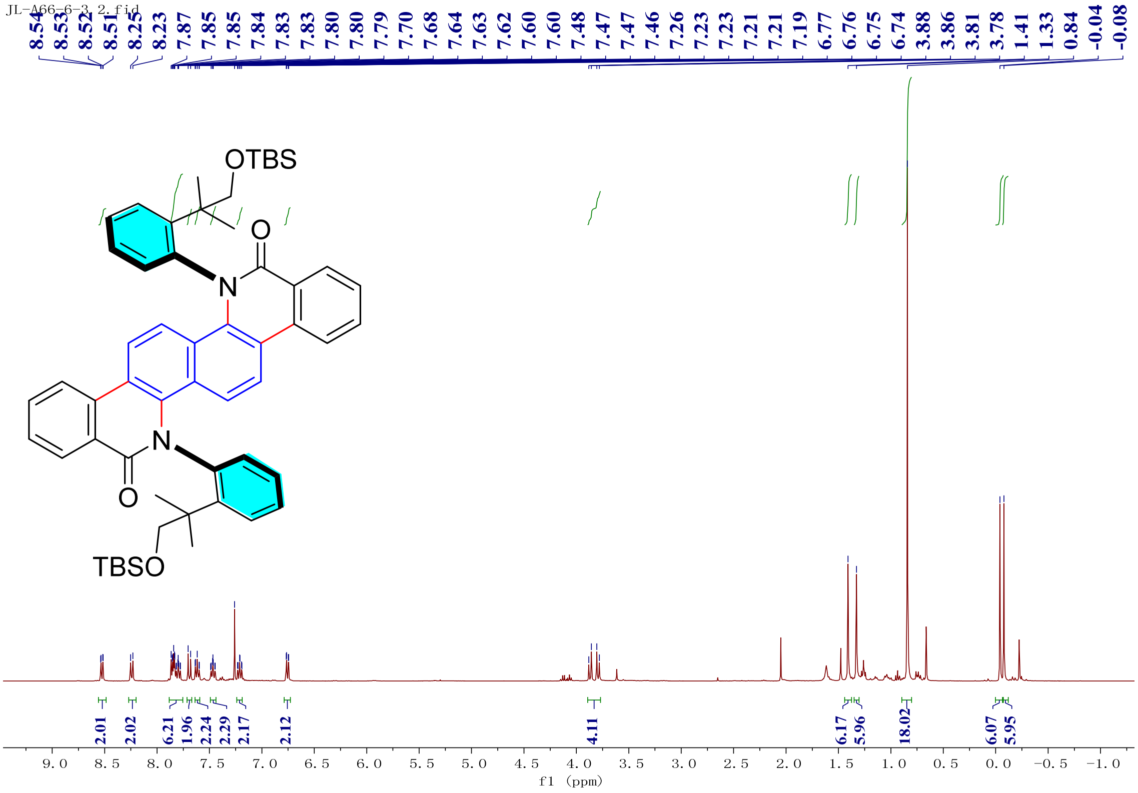

^{13}C NMR (101 MHz, Chloroform-*d*)

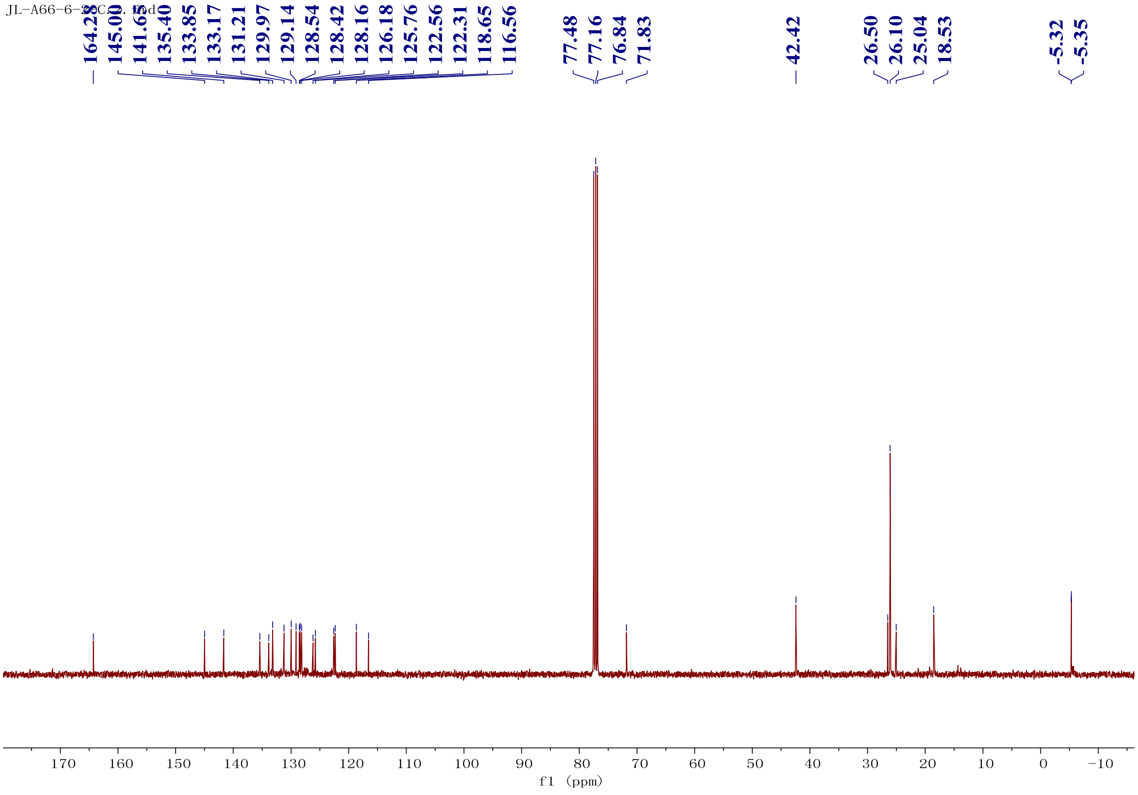

54′

^{1}H NMR (400 MHz, Chloroform-*d*)

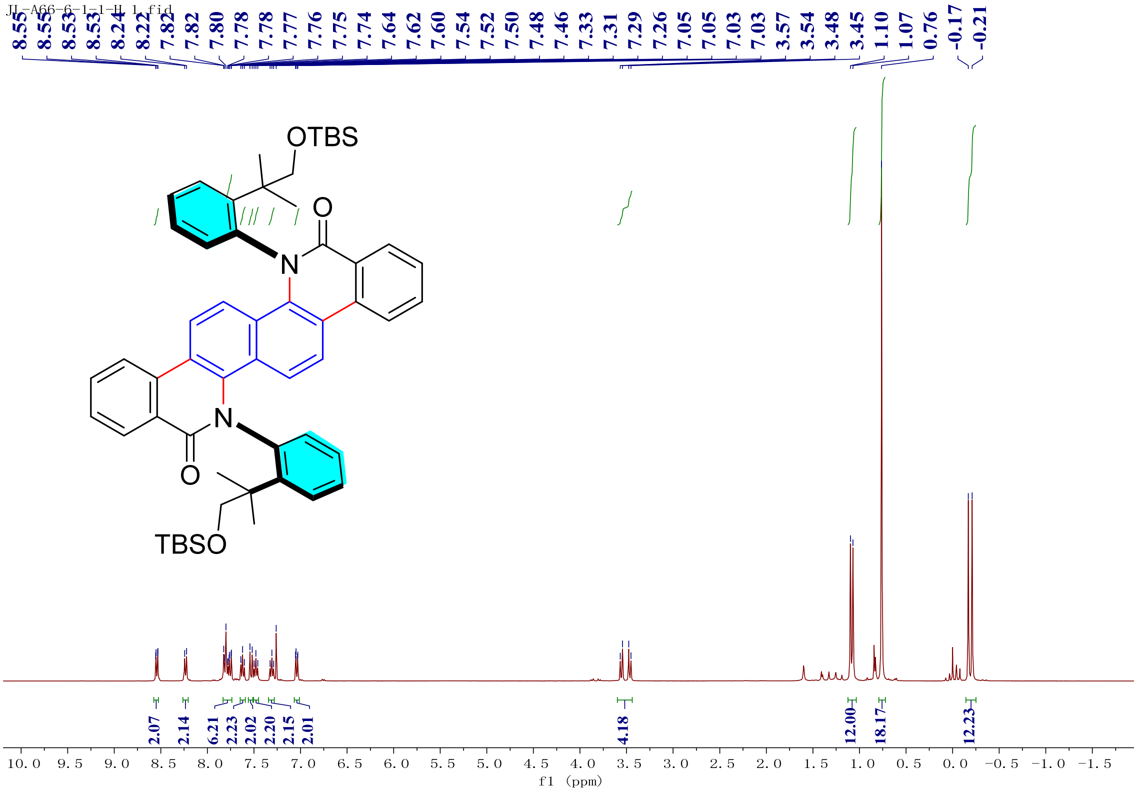

^{13}C NMR (101 MHz, Chloroform-*d*)

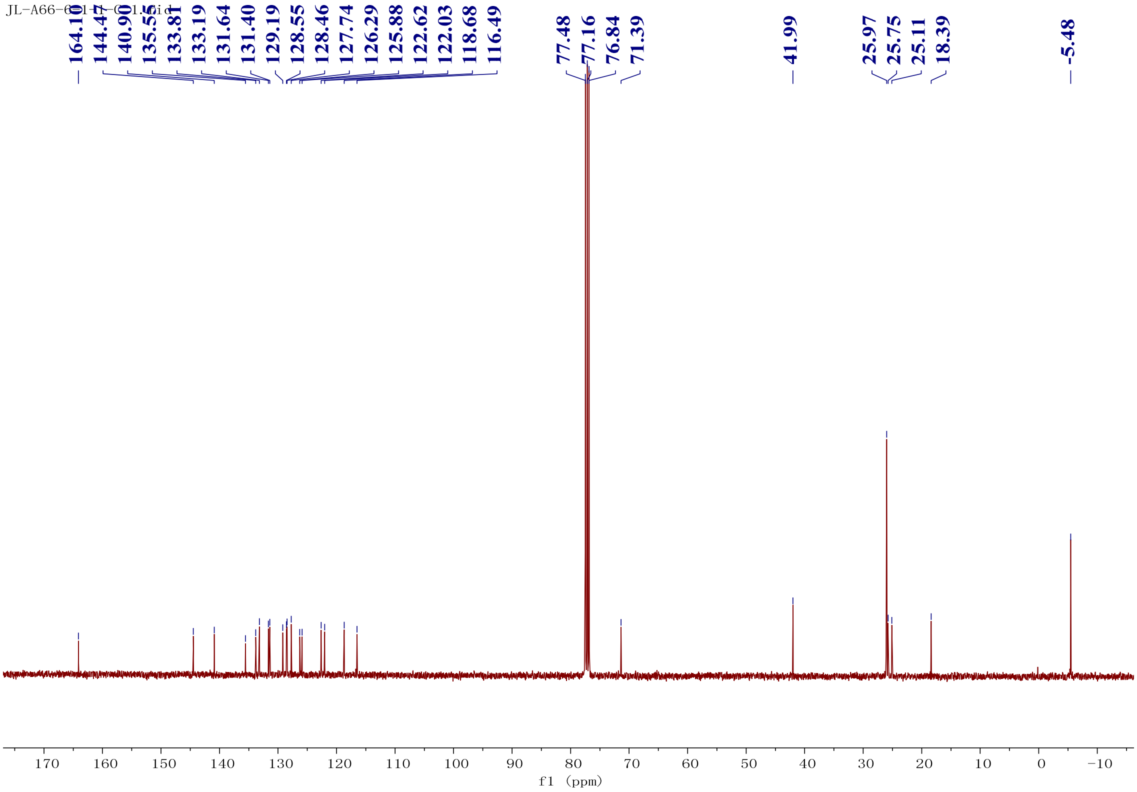

55

^{1}H NMR (600 MHz, Chloroform-*d*)

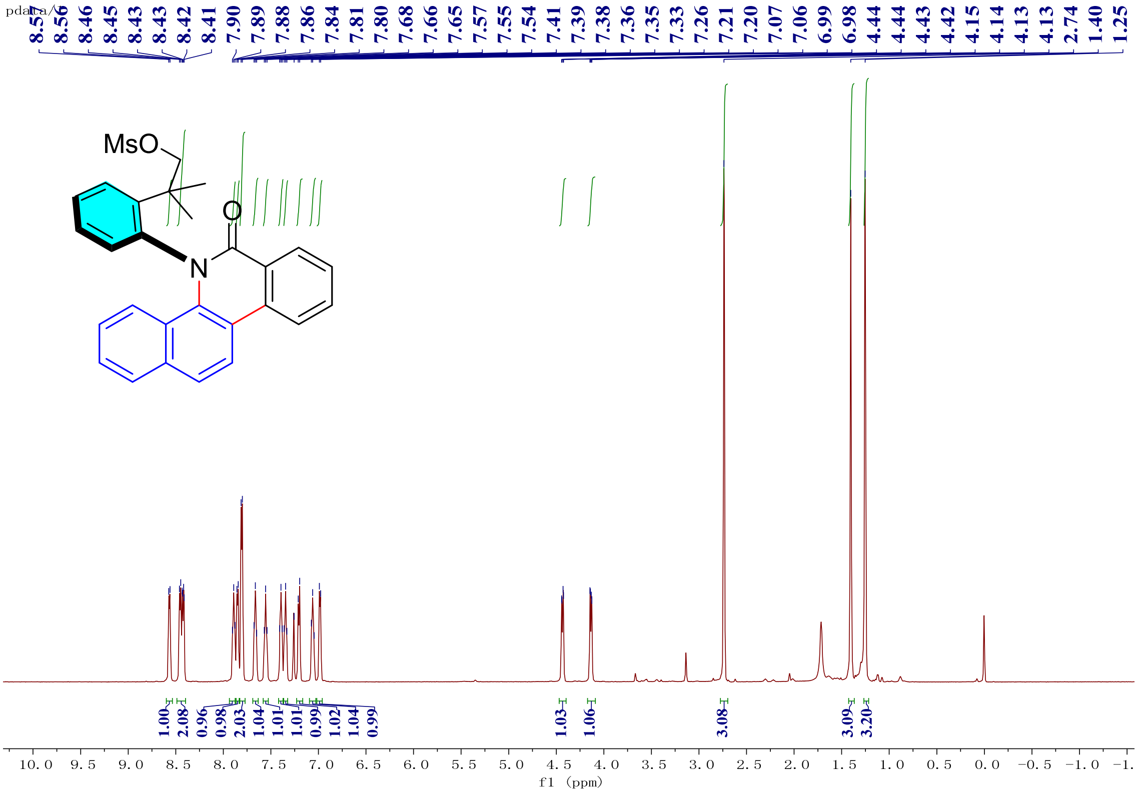

^{13}C NMR (151 MHz, Chloroform-*d*)

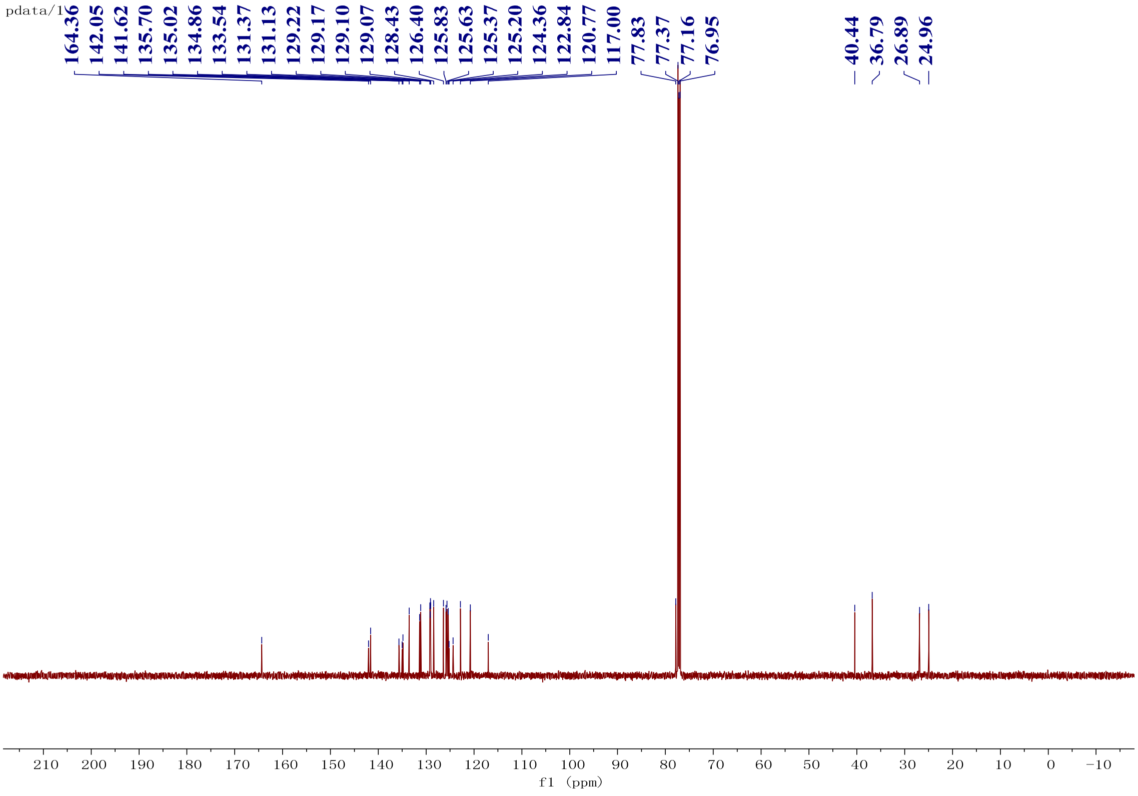

56

^{1}H NMR (600 MHz, Chloroform-*d*)

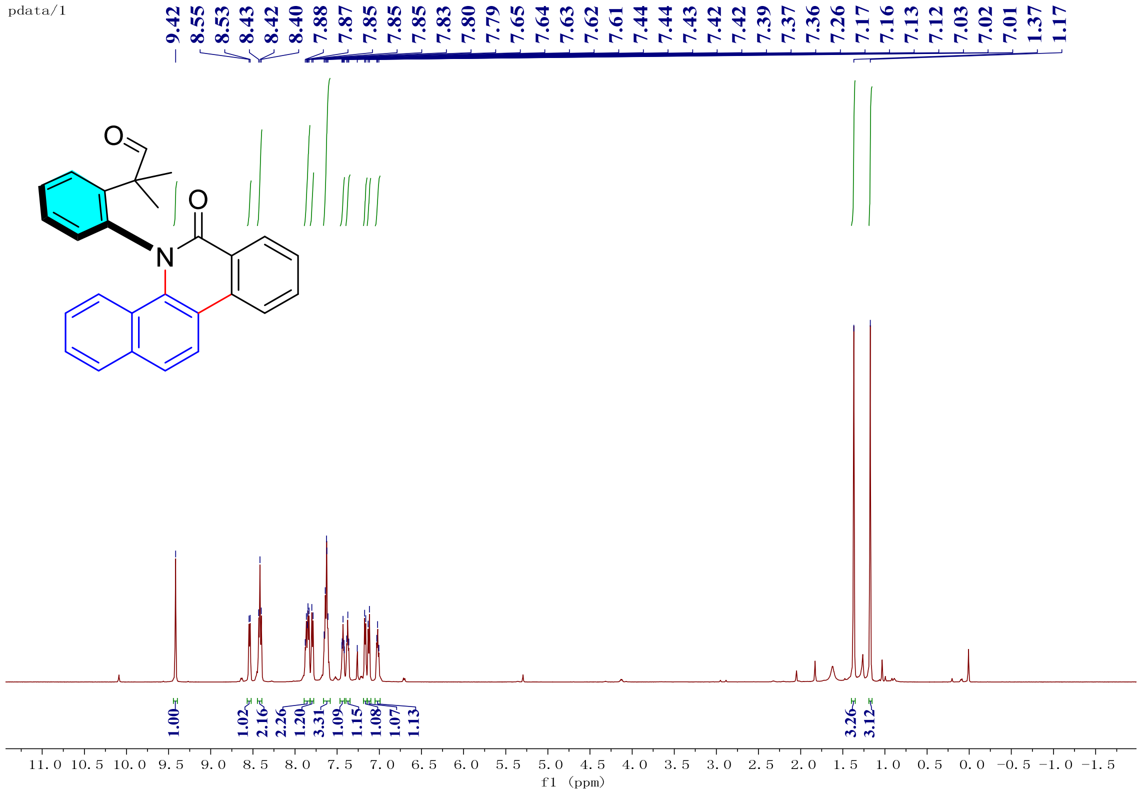

^{13}C NMR (151 MHz, Chloroform-*d*)

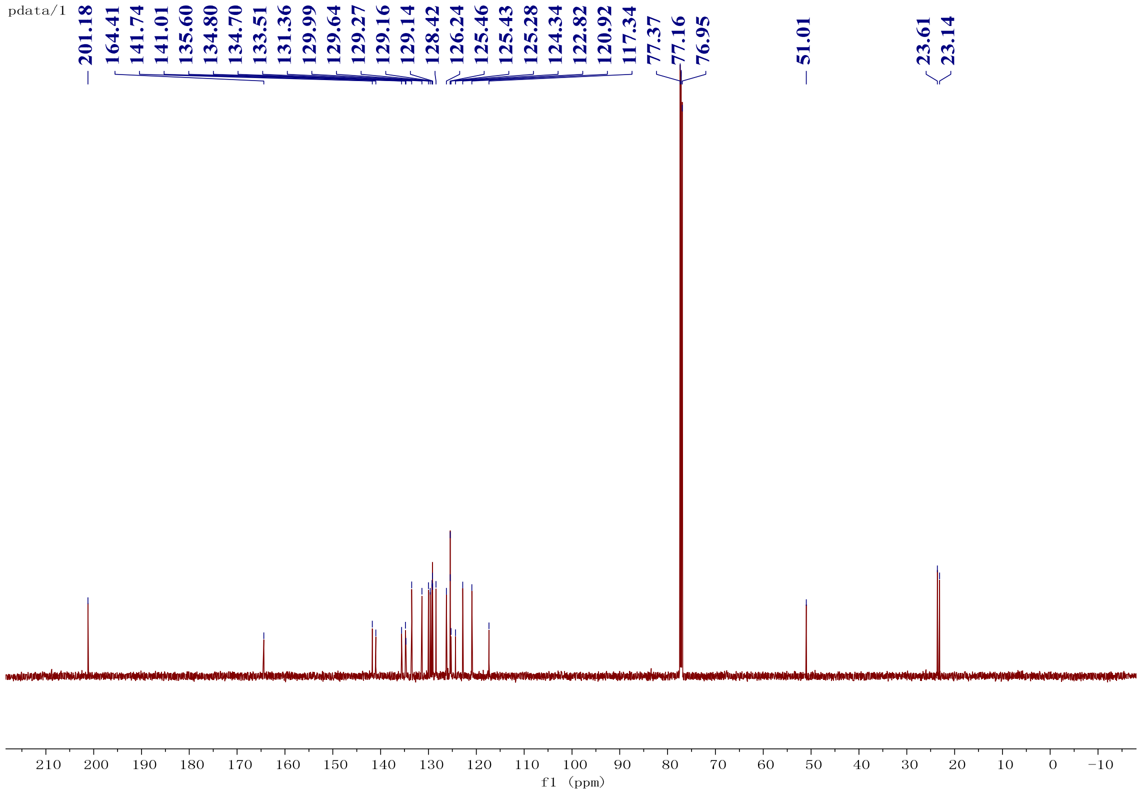

57

^{1}H NMR (600 MHz, Chloroform-*d*)

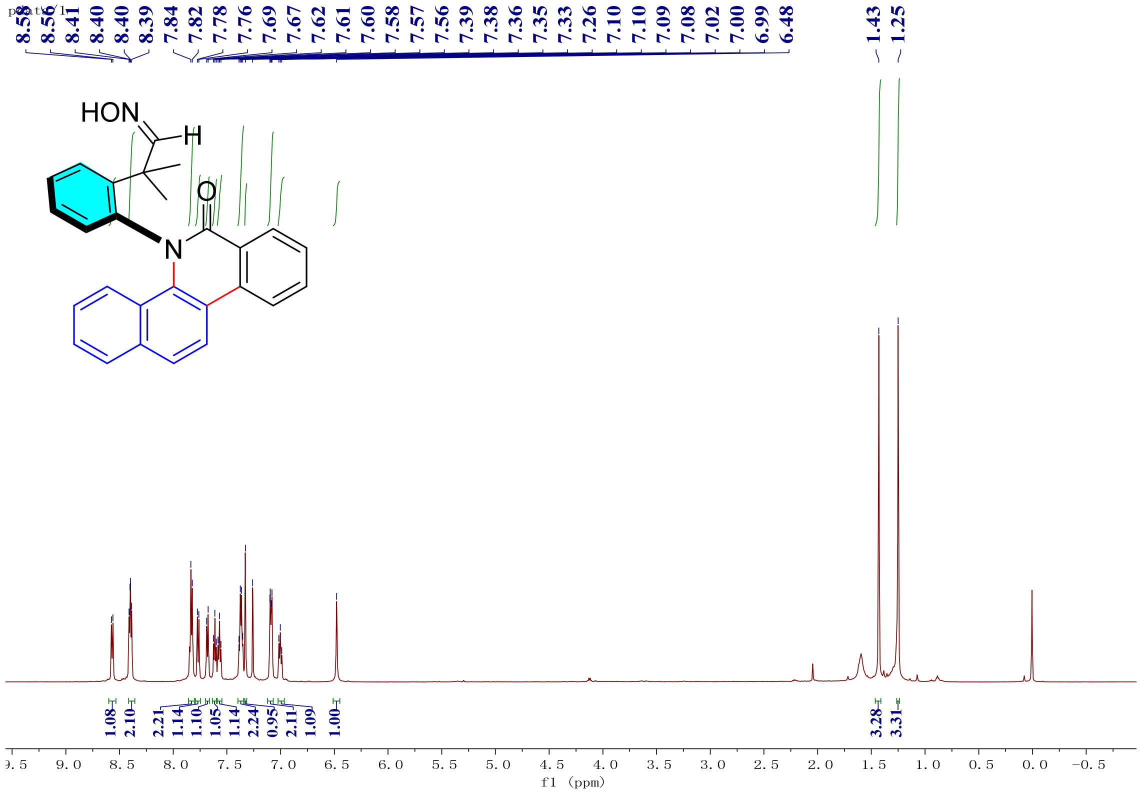

^{13}C NMR (151 MHz, Chloroform-*d*)

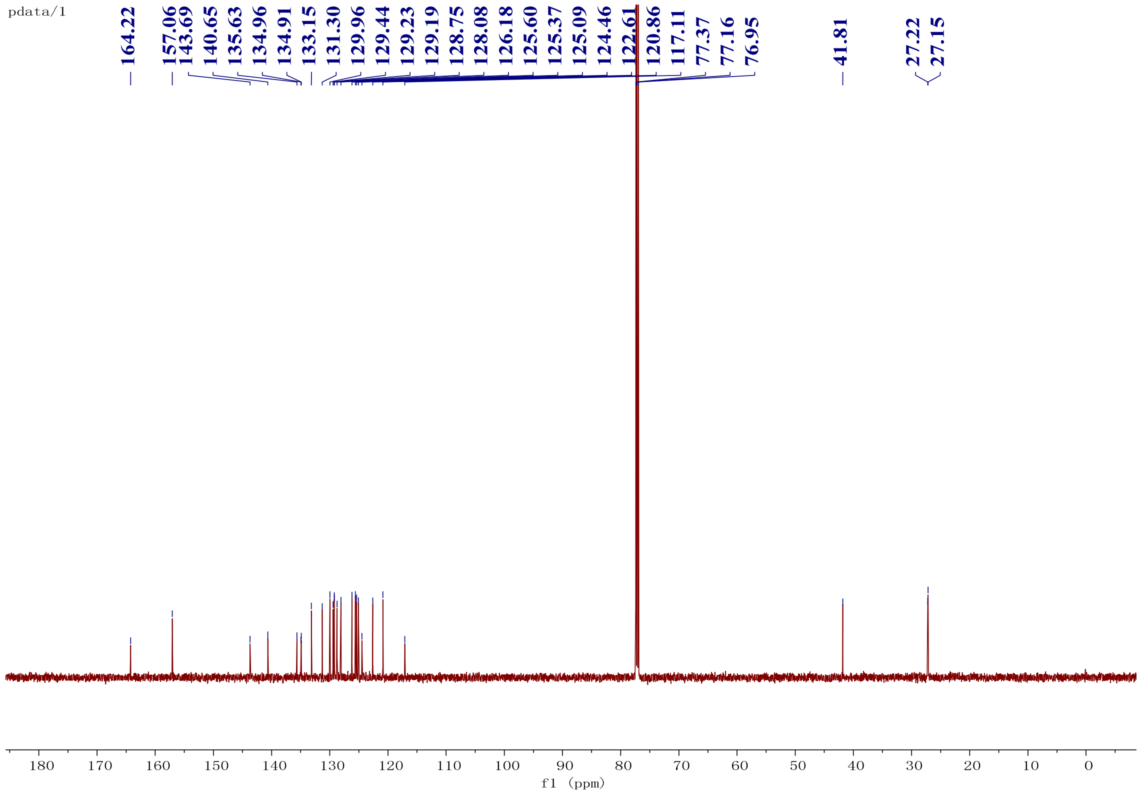

58

^{1}H NMR (400 MHz, Chloroform-*d*)

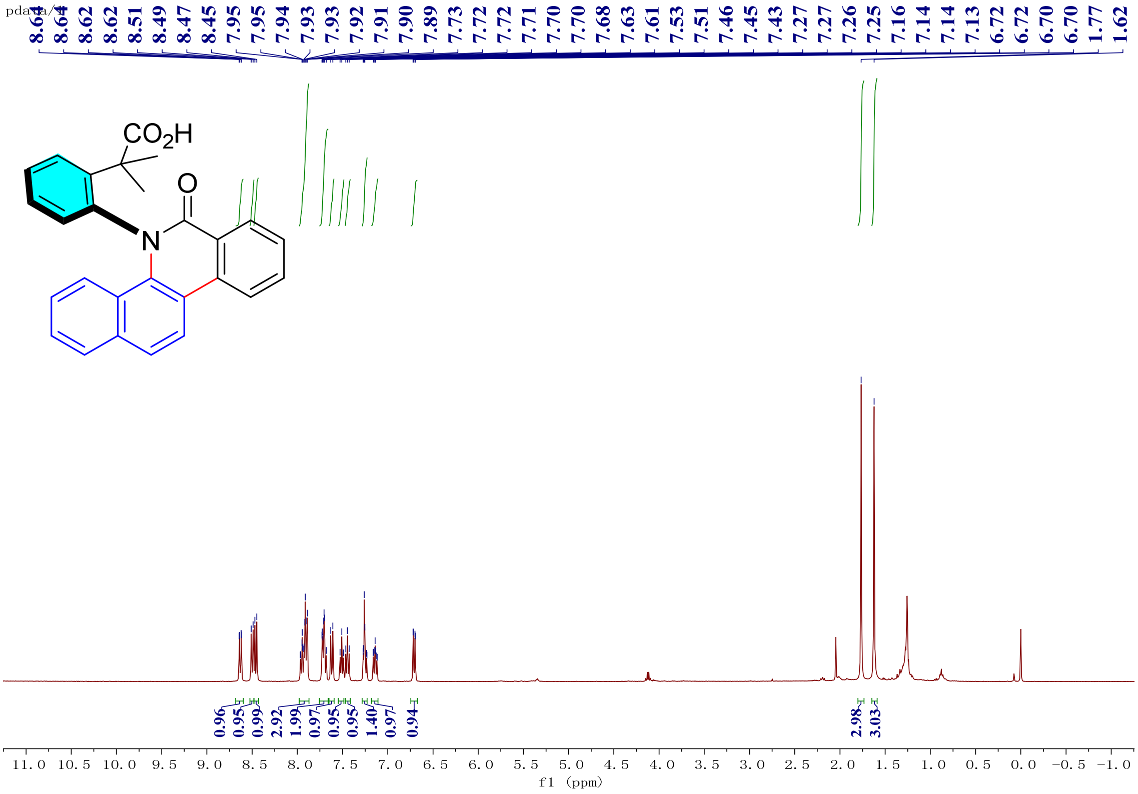

^{13}C NMR (101 MHz, Chloroform-*d*)

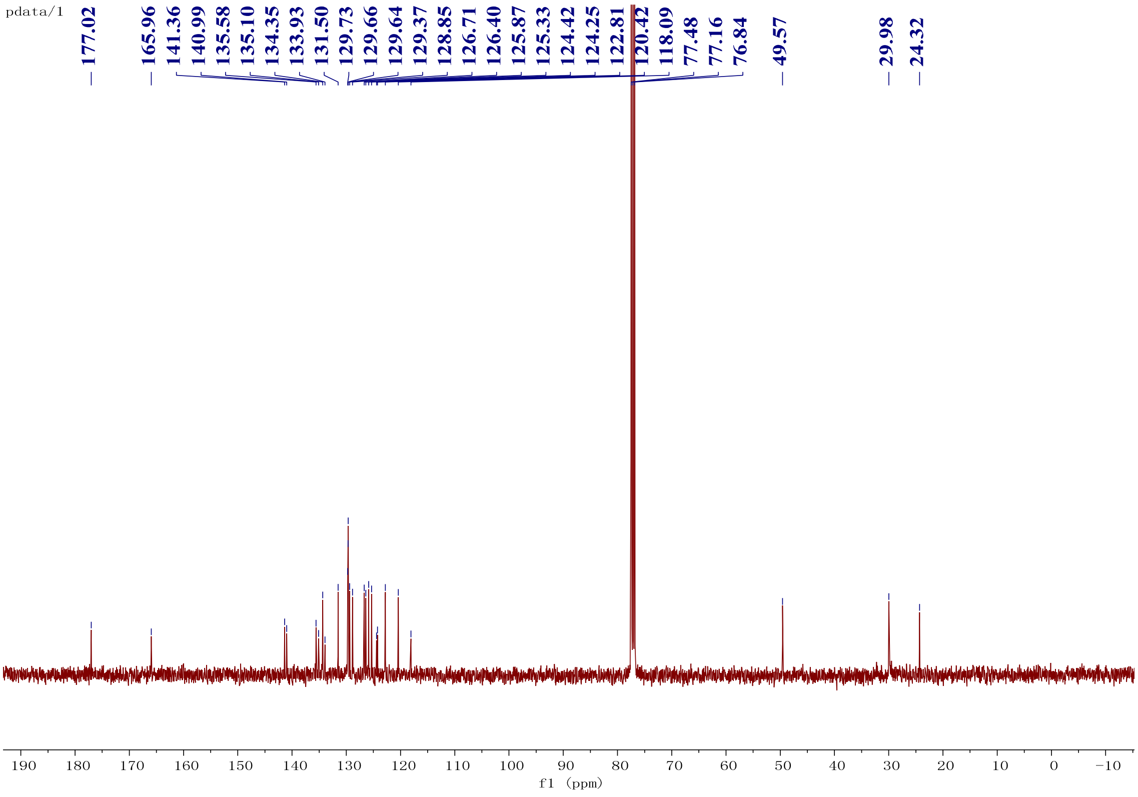

58′

^{1}H NMR (400 MHz, Chloroform-*d*)

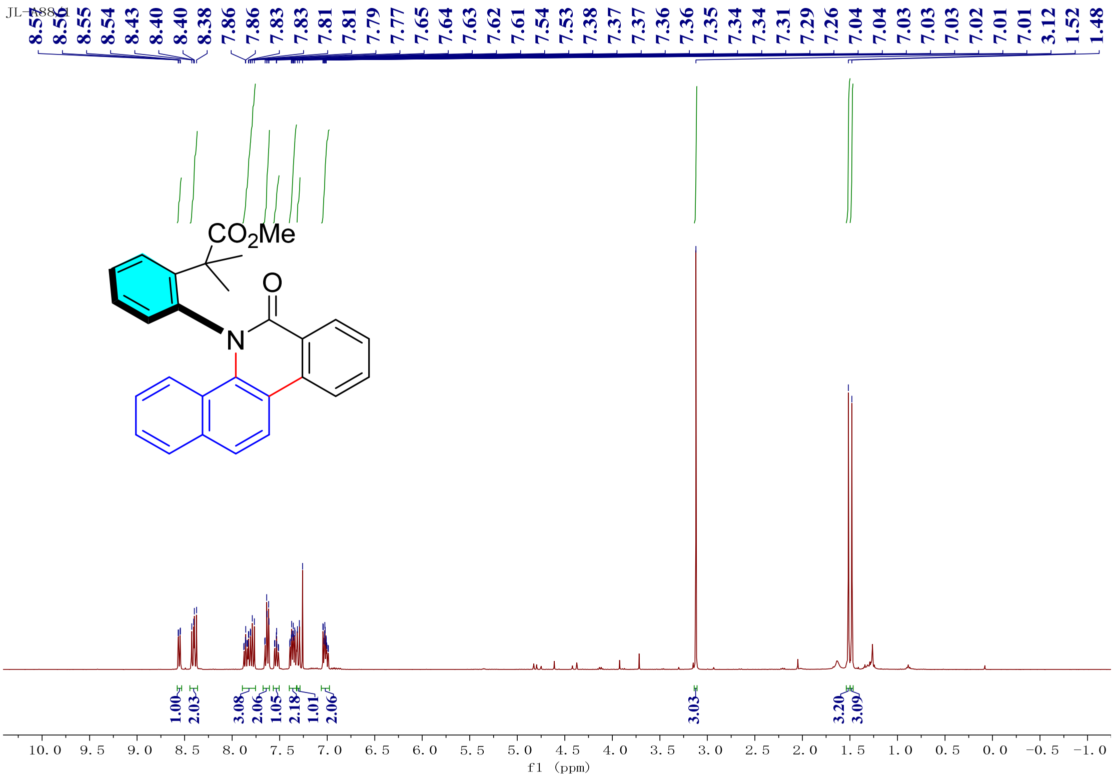

^{13}C NMR (101 MHz, Chloroform-*d*)

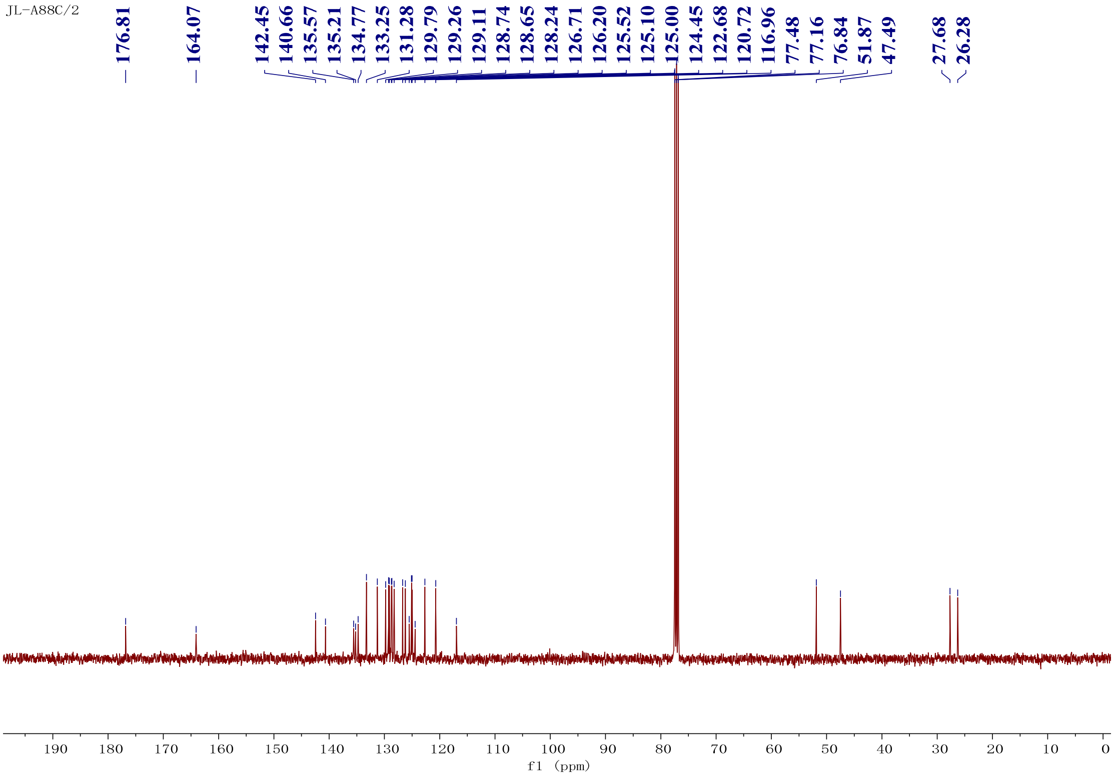

61

^{1}H NMR (600 MHz, Chloroform-*d*)

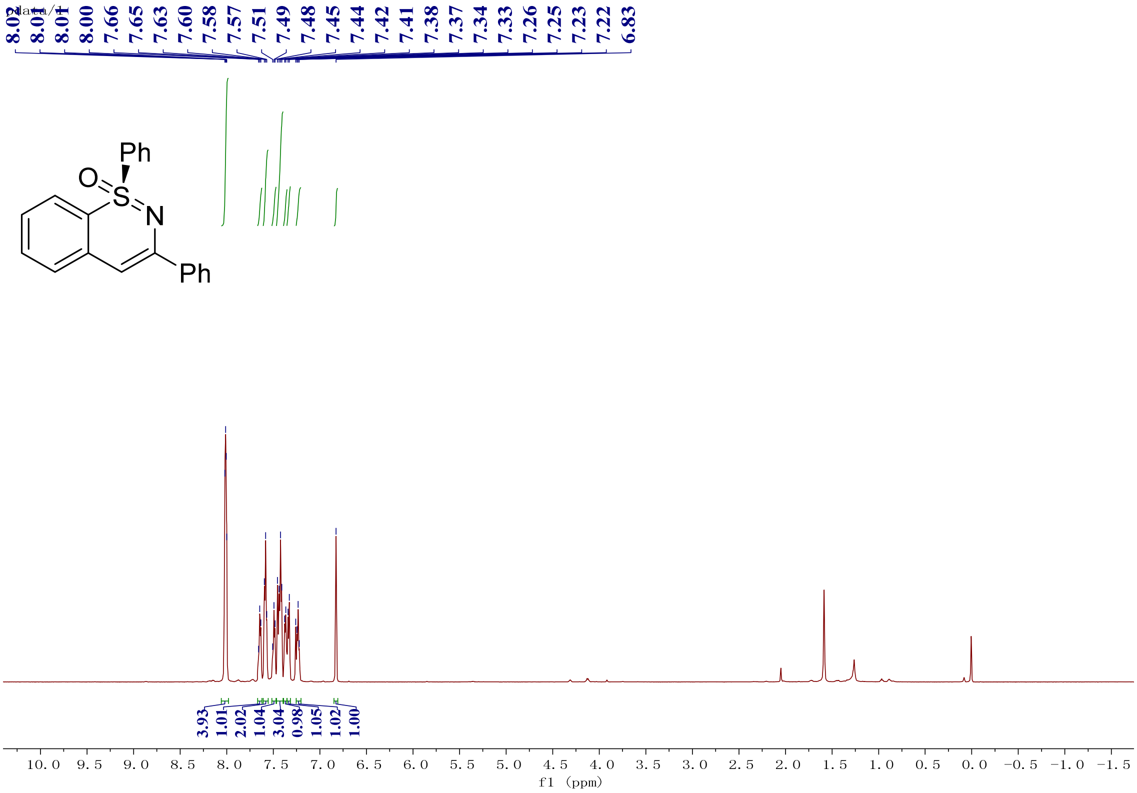

63

^{1}H NMR (600 MHz, Chloroform-*d*)

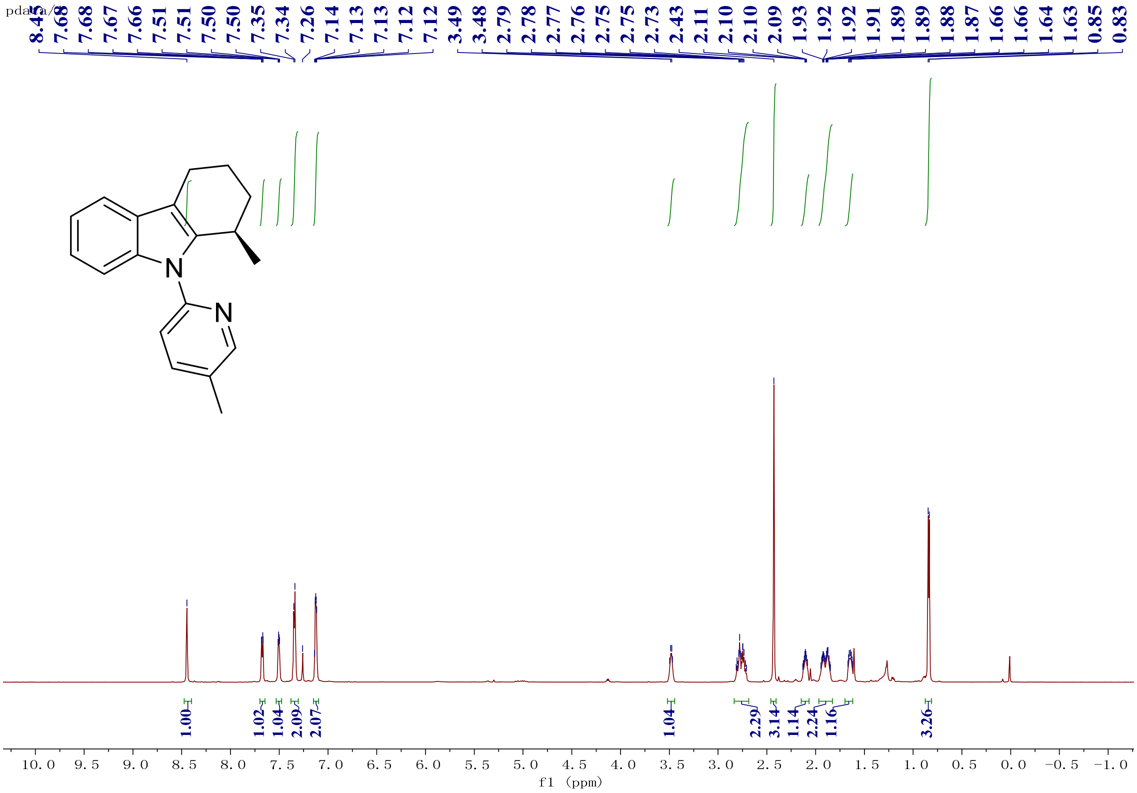

64

^{1}H NMR (600 MHz, Chloroform-*d*)

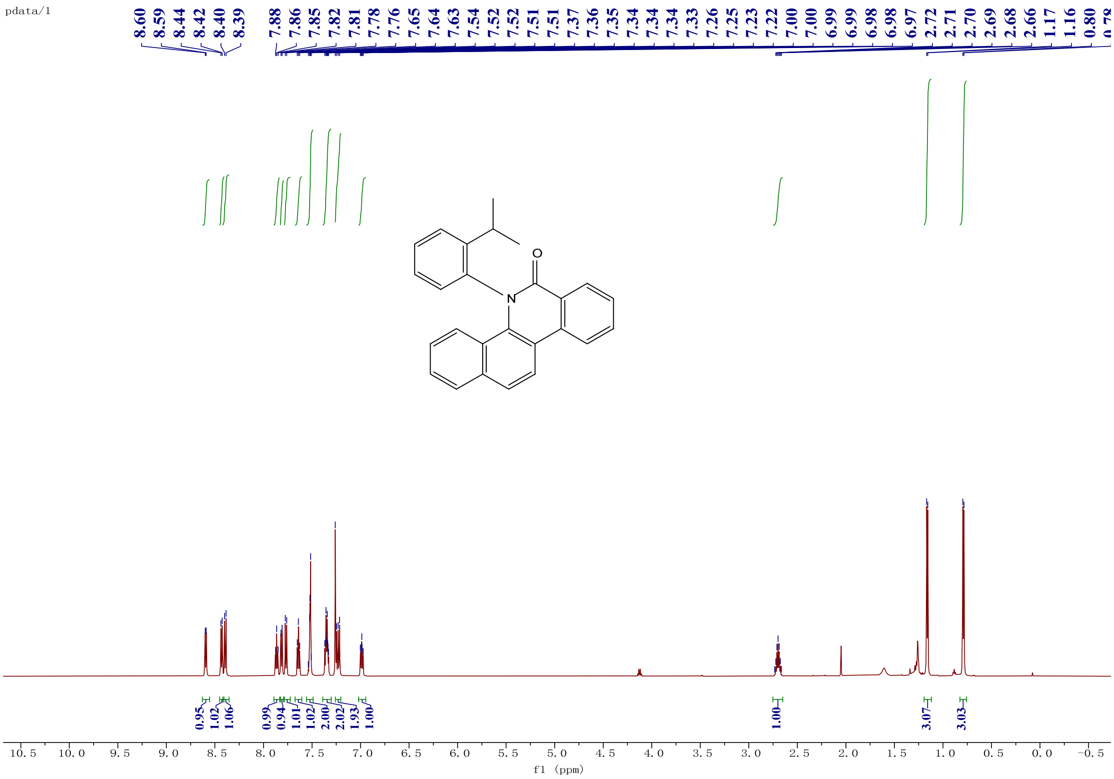

^{13}C NMR (151 MHz, Chloroform-*d*)

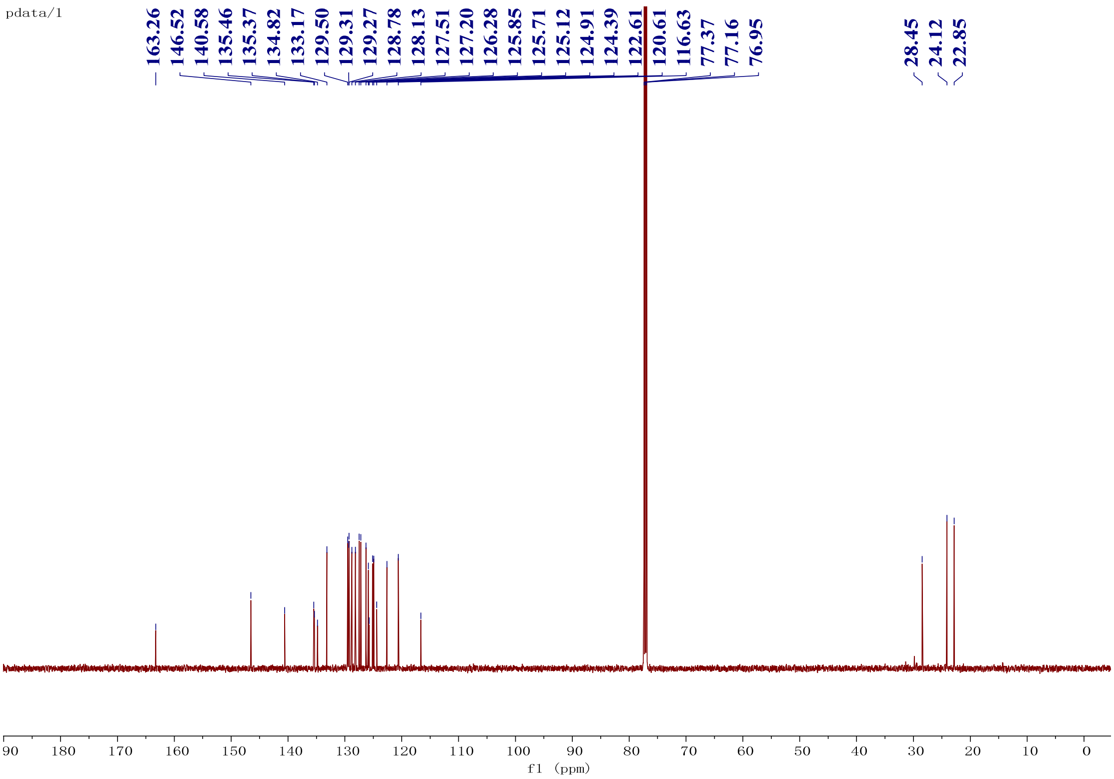

65

^{1}H NMR (600 MHz, Chloroform-*d*)

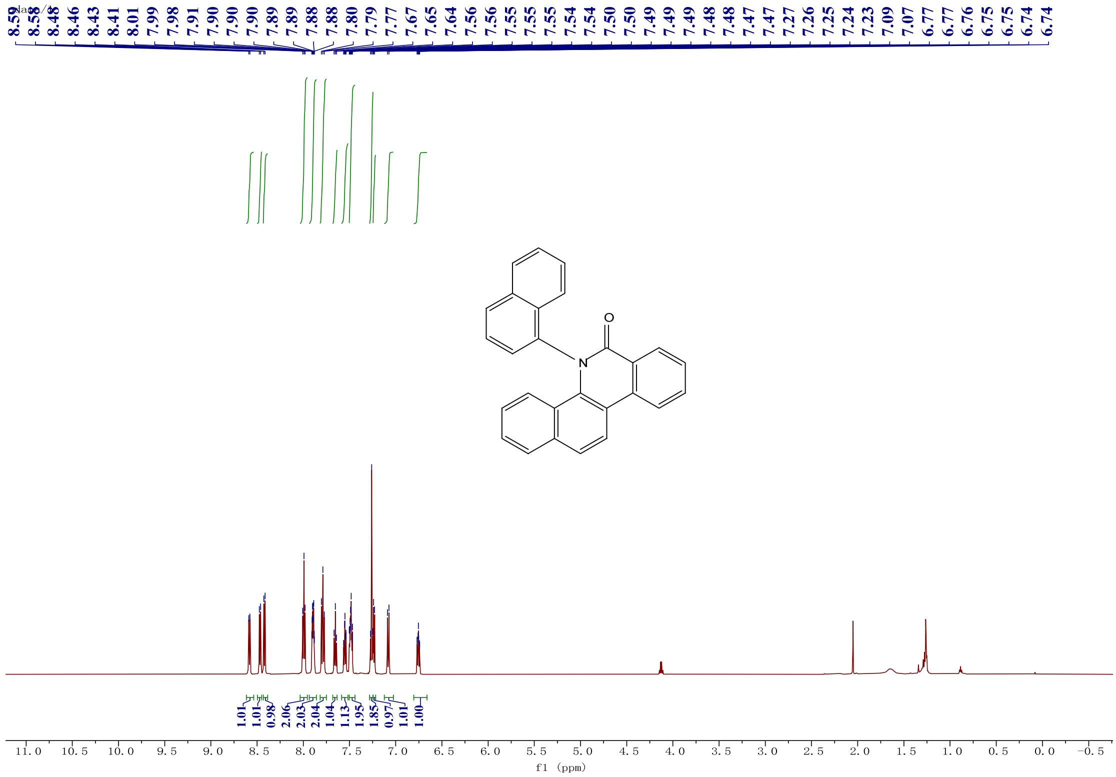

^{13}C NMR (151 MHz, Chloroform-*d*)

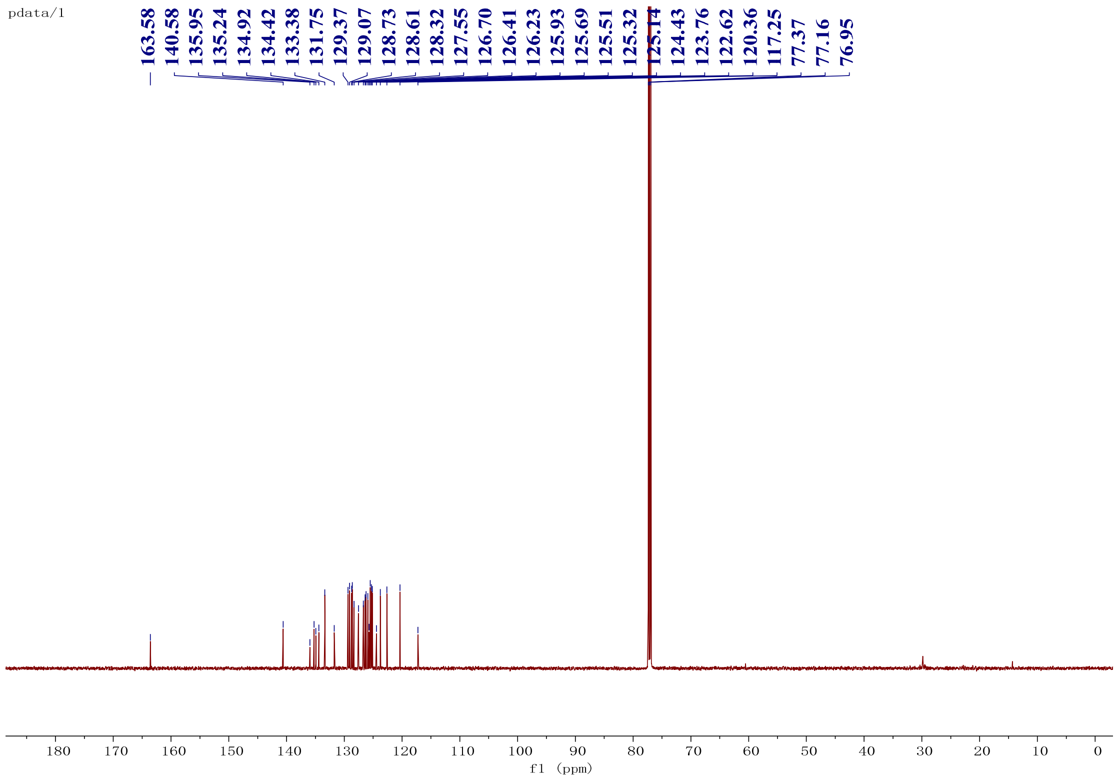

4. References

[1] Cheng, X.-K.; Li, T.-T.; Liu, Y.-T.; Lu, Z. Stereo-and Enantioselective Benzylic C−H alkenylation via Photoredox/Nickel Dual Catalysis. *ACS Catal.* **11**, 11059-11065 (2021).

[2] Liu, X. *et al.* A Robust Microfluidic Device for the Synthesis and Crystal Growth of Organometallic Polymers with Highly Organized Structures. *Angew. Chem. Int. Ed*. **54**, 1846-1850 (2015).

[3] Liu, Z.-S. *et al.* An axial-to-axial chirality transfer strategy for atroposelective construction of C–N axial chirality. *Chem* **7**, 1917-1932 (2021).

[4] Zhou, T. *et al.* Effiffifficient Synthesis of Sulfur-Stereogenic Sulfoximines via Ru(II)-Catalyzed Enantioselective C−H Functionalization Enabled by Chiral Carboxylic Acid. *J. Am. Chem. Soc.* **143**, 6810-6816 (2021).

[5] Dhawa, U.; Connon, R.; Oliveira, J. C. A.; Steinbock, R.; Ackermann, L. Enantioselective Ruthenium-Catalyzed C−H Alkylations by a Chiral Carboxylic Acid with Attractive Dispersive Interactions. *Org. Lett.* **23**, 2760-2765 (2021).

[6] Raut, V. S. *et al.* Enantioselective Syntheses of Furan Atropisomers by an Oxidative Central-to-Axial Chirality Conversion Strategy. *J. Am. Chem. Soc*. **139**, 2140-2143 (2017).
